# Retrospective genomic analysis of sorghum adaptation to temperate-zone grain production

**DOI:** 10.1186/gb-2013-14-6-r68

**Published:** 2013-06-26

**Authors:** Carrie S Thurber, Justin M Ma, Race H Higgins, Patrick J Brown

**Affiliations:** 1 Energy Biosciences Institute, University of Illinois, Urbana, IL, USA; 2 Department of Crop Sciences, University of Illinois, Urbana, IL, USA

**Keywords:** Genotyping-by-sequencing, introgression, photoperiod, flowering time, dwarfism

## Abstract

**Background:**

Sorghum is a tropical C_4 _cereal that recently adapted to temperate latitudes and mechanized grain harvest through selection for dwarfism and photoperiod-insensitivity. Quantitative trait loci for these traits have been introgressed from a dwarf temperate donor into hundreds of diverse sorghum landraces to yield the Sorghum Conversion lines. Here, we report the first comprehensive genomic analysis of the molecular changes underlying this adaptation.

**Results:**

We apply genotyping-by-sequencing to 1,160 Sorghum Conversion lines and their exotic progenitors, and map donor introgressions in each Sorghum Conversion line. Many Sorghum Conversion lines carry unexpected haplotypes not found in either presumed parent. Genome-wide mapping of introgression frequencies reveals three genomic regions necessary for temperate adaptation across all Sorghum Conversion lines, containing the *Dw1, Dw2*, and *Dw3 *loci on chromosomes 9, 6, and 7 respectively. Association mapping of plant height and flowering time in Sorghum Conversion lines detects significant associations in the *Dw1 *but not the *Dw2 *or *Dw3 *regions. Subpopulation-specific introgression mapping suggests that chromosome 6 contains at least four loci required for temperate adaptation in different sorghum genetic backgrounds. The *Dw1 *region fractionates into separate quantitative trait loci for plant height and flowering time.

**Conclusions:**

Generating Sorghum Conversion lines has been accompanied by substantial unintended gene flow. Sorghum adaptation to temperate-zone grain production involves a small number of genomic regions, each containing multiple linked loci for plant height and flowering time. Further characterization of these loci will accelerate the adaptation of sorghum and related grasses to new production systems for food and fuel.

## Background

Cereals have been selected by humans for thousands of years, first during their domestication from wild grasses and subsequently for increased yield, uniformity, and adaptation to new environments and management practices [1-3]. Specific molecular pathways have recently proven useful for cereal adaptation to modern, high-input agriculture. For example, the Green Revolution exploited allelic variation in the gibberellin pathway in wheat and rice to produce semi-dwarf cultivars with increased harvest index and improved resistance to lodging [4-7]. Similar phenotypic changes occurred during the creation of dwarf grain sorghum suitable for mechanized harvest at temperate latitudes. Understanding the genetic control of these changes is critical for the efficient transfer of useful alleles, both between tropical and temperate growing regions and between breeding programs for different end uses.

Sorghum is the fifth most important cereal crop worldwide [8] and is widely grown in temperate regions, but was domesticated in the African tropics [9]. Temperate adaptation for grain production in sorghum requires photoperiod-insensitivity, for early maturity, and dwarfism, both of which involve at least four major loci [10]. Of the major maturity loci (*Ma1-Ma6*), *Ma1 *has been identified as PRR37 [11] and *Ma3 *as Phytochrome B [12]. Of the major dwarfing loci (*Dw1-Dw4*), *Dw3 *has been identified as PGP1/PGP19, an auxin transporter orthologous to maize *brachytic2 *[13]. *Dw2 *and *Dw1 *are uncloned, with the former closely-linked to *Ma1 *[14] and the latter mapping to chromosome 9 [15,16].

**Figure 1:**
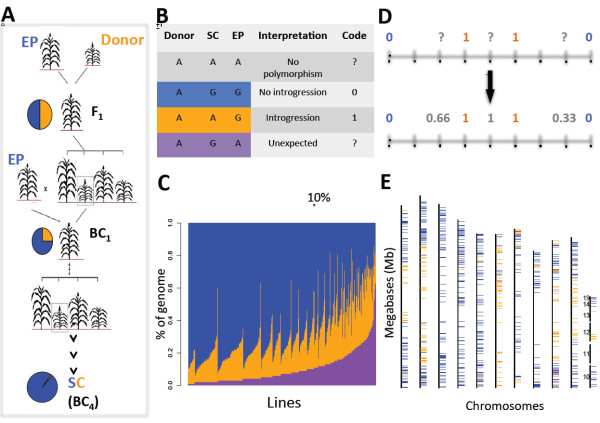
**Molecular analysis of the SC Program**. (**A**) Backcrossing scheme used to create SC lines from EP lines and an elite donor. Four generations of backcrossing were completed, with selection during each F_2 _generation for short, photoperiod-insensitive plants. (**B**) Interpretation of molecular data from donor, SC, and EP lines. SC alleles shared with either the donor or EP lines indicate that introgression has occurred (orange) or not occurred (blue), respectively. SC alleles not found in either parent are unexpected (purple) and were treated as missing data. (**C**) Genome content of 580 SC lines. Each vertical bar represents a single SC line. Bars are ordered by the percentage of unexpected genotypes. The solid black vertical line indicates a cutoff of 10% unexpected genotypes. (**D**) Missing and unexpected introgression scores (question marks) were assigned values based on the mean of each flanking marker weighted by its physical distance. (**E**) A representative example of the introgression maps created for each SC line. The 10 sorghum chromosomes are shown from left to right. The 11th column displays unanchored contigs in the sorghum genome. Long-range linkage disequilibrium in SC lines was exploited to place these contigs on the sorghum physical map.

The oligogenic control of these important agronomic traits in sorghum was exploited through a backcross breeding scheme known as the Sorghum Conversion Program (SCP) [17]. Mutations for photoperiod-sensitivity and dwarfism had previously arisen spontaneously in temperate regions of Africa, Asia, and the southern US, and were already being used for grain sorghum production. However, the genetic base of US grain sorghum remained very narrow. During the SCP, genomic regions conferring early maturity and dwarfing were introgressed from an elite donor into approximately 800 exotic sorghum accessions representing the breadth of genetic diversity in sorghum. The resulting SC lines are closely related to their Exotic Progenitor (EP) lines, but differ dramatically in plant height and flowering time due to the presence of donor introgressions (Figure 1A). The elite donor, BTx406, carries recessive alleles for photoperiod-insensitivity and dwarfism at *Ma1 *and *Dw1-Dw3*, respectively [17], so these loci are expected to show a high frequency of donor introgression in SC lines. Klein *et al. *[14] previously mapped introgressions on chromosome 6 in a subset of SC lines and showed that several of them contain vast introgressed tracts around the linked *Ma1-Dw2 *loci. However, the genetic architecture of temperate adaptation in the SC lines (the number and linkage of loci as well as their frequencies in different subpopulations) has not been systematically studied on a genome-wide basis. This information can be used both to identify the underlying targets of the SCP and to help guide more efficient, marker-directed conversion of exotic sorghums to temperate-adapted varieties.

In this study, we use genotyping-by-sequencing (GBS) [18,19] to generate genome-wide single nucleotide polymorphism (SNP) data for 580 pairs of EP and SC lines, for a total of 1,160 sorghum inbreds. We then employ a novel introgression mapping approach to identify loci required for temperate adaptation, and validate our results using both phenotype-genotype association and population differentiation (F_st_) analyses.

## Results and discussion

### Genotyping-by-sequencing of SC lines and their exotic progenitors

To map elite donor introgressions in SC lines, we genotyped 580 pairs of SC and their corresponding EP lines (Additional File 1) at 54,034 SNPs using GBS. Briefly, we constructed reduced-representation DNA libraries using pairs of restriction enzymes [18], sequenced them in 96-plexes on the Illumina HiSeq, and processed the data using the TASSEL GBS pipeline [20]. We found that combining two separate double digests nearly doubled the number of SNPs called per sample (Additional File 2). The full dataset contained 0.3% heterozygous genotypes. Partial imputation using the TASSEL GBS pipeline reduced the proportion of missing genotypes from 66% to 23%.

Three different seed sources of the elite donor line, BTx406, were used to construct 28 different genomic libraries. Three of these libraries originating from a single seed source of BTx406 showed low concordance and were removed from subsequent analyses (Additional File 3). This low concordance was likely due to laboratory error as it was confined to libraries prepared on a single day. The remaining 25 libraries from the elite donor contained clear, homozygous majority calls for 53,037 SNPs. The elimination of approximately 7,000 SNPs in complete linkage disequilibrium with another SNP less than 64 basepairs (bp) away resulted in a dataset of 46,137 SNPs for calling introgressions.

Each trio of homozygous genotypes for a given SNP across a SC line, its corresponding EP line, and the elite donor has four possible outcomes (Figure 1B), most common of which is a lack of polymorphism. Of the three remaining polymorphic combinations, shared genotypes between a SC line and its EP line provide evidence that introgression has not occurred, whereas shared genotypes between a SC line and the donor provide evidence that introgression has occurred. The fourth possibility is unexpected: a SC line has a genotype not found in either of its parents. Unexpected genotypes could result from laboratory error (mix-up or cross-contamination of seed or DNA samples in our laboratory), historical error during the SCP (pollen contamination or error in pedigree records), or uncharacterized heterozygosity and/or genetic drift during the maintenance of the EP, SC, or donor lines. We used the proportion of unexpected genotypes as a quality-control filter to prune both markers and individuals. First, we discarded 75 markers with >20% unexpected genotypes, of which 55 were on chromosome 6 and 44 were found between 30 Mb and 43 Mb on chromosome 6, a region that includes *Ma1 *and likely includes *Dw2 *[14]. A possible explanation for the high proportion of unexpected genotypes in this region is that certain sources of the elite donor BTx406 used during the SCP differed from our BTx406 consensus genotype in this region. In support of this hypothesis, we note that the seed source of BTx406 derived from Lubbock, TX, very close to where the SCP was carried out, is heterozygous for many of the markers on chromosome 6 that were discarded due to having >20% unexpected genotypes. Second, we discarded 190 SC-EP pairs with >10% unexpected genotypes. The distribution of unexpected genotypes in some SC lines is clustered (for example, SC1104; Additional File 4), suggesting that genomic segments from a temperate donor other than BTx406 were introgressed. In other SC lines the unexpected genotypes are scattered, suggesting that genetic drift may have occurred between the EP line that was used as a recurrent parent and the EP line that was genotyped. For the 16 SC-EP pairs that have >33% unexpected genotypes, a clerical error of some kind - during transcription of pedigree records, seed packet labels, or DNA plates - is most likely. For the remainder of our analysis, we retained a set of 390 SC-EP pairs with <10% unexpected genotypes (Figure 1C), genotyped at 46,062 markers (Additional File 5).

### Inferring elite donor introgressions in SC lines

Introgression maps were generated for each SC line (Figure 1E; Additional File 4). The long-range linkage disequilibrium in the SC lines was exploited to map unanchored contigs in the sorghum genome (Additional File 6). After setting non-polymorphic and unexpected genotypes as missing, missing data were inferred using flanking markers (Figure 1D). Introgression frequency was then calculated for each marker as the proportion of the 390 SC lines carrying a BTx406 introgression. The theoretical expectation of introgression frequency after four backcrosses in the absence of selection is roughly 3%. The standard deviation of this value in individual SC lines, in a species with 10 chromosomes and a map length of roughly 16 Morgans, is also roughly 3% [21], so that the introgression frequency in a sample of 390 SC lines is expected to range from 2% to 4% in the absence of selection. Because our dataset contains a substantial proportion of missing data, introgressions that are very small and very rare may be missed entirely. However, we find that every chromosome contains regions with introgression frequencies >4%, indicating linkage to a target of selection during the SCP.

### Three genomic regions are associated with temperate adaptation in sorghum

**Figure 2:**
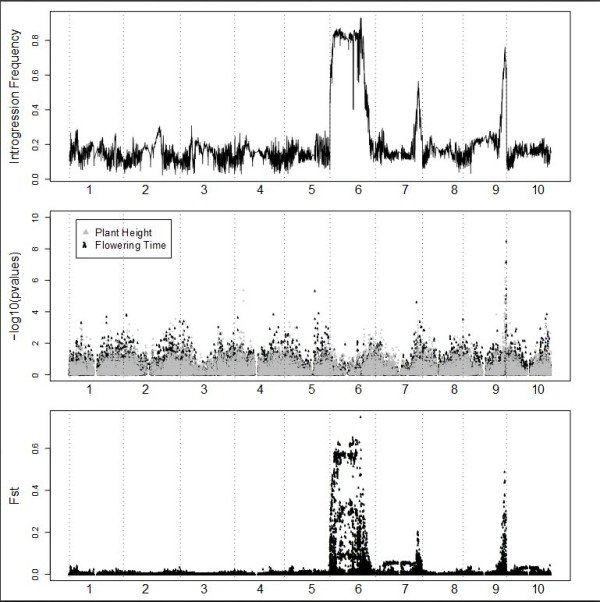
**Genome-wide analysis of temperate adaptation in sorghum**. The x axis in each panel represents physical distance along the ten sorghum chromosomes. The top panel shows introgression frequency in a set of 390 SC-EP pairs with <10% unexpected genotypes (see Methods for calculation). The middle panel shows phenotypic associations with plant height and flowering time in the full set of 580 genotyped SC lines. The bottom panel shows population differentiation (F_st_) between the full sets of 580 SC lines and 580 EP lines.

Three regions of the sorghum genome show pronounced peaks in introgression frequency in the SC lines (Figure 2; top panel), suggesting that these regions are nearly indispensable for adaptation to temperate grain production. We then used two methods to validate the introgression mapping results. First, we assessed functional variation for plant height and flowering time in SC lines by performing association mapping for these traits in the complete set of 580 genotyped SC lines (Figure 2; middle panel). EP lines were not included because most do not flower at temperate latitudes. Significant phenotypic associations were found in the *Dw1 *but not the *Dw2 *or *Dw3 *genomic regions. Second, to ensure that the introgression mapping results were not unduly affected by unexpected genotypes, we calculated F_st _between the complete sets of 580 genotyped SC lines and 580 EP lines and found that regions of high F_st _mirror the regions of high introgression frequency almost exactly (Figure 2; bottom panel). Unlike introgression frequency, F_st _makes no assumptions about the pedigrees of the SC lines.

### The cloned *Dw3 *locus on chromosome 7 is tagged using three different methods

**Figure 3:**
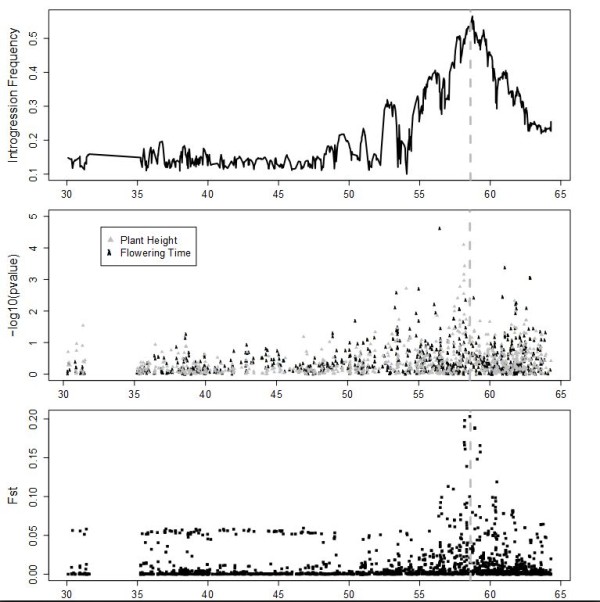
**Introgression frequency, phenotypic associations, and population differentiation in the *Dw3 *region on sorghum chromosome 7**. Panels are the same as in Figure 2. The location of *Dw3 *at 58.6 Mb is shown with a vertical dashed gray line.

Chromosome 7, which contains the known, cloned target *Dw3 *at 58.6 Mb, has a peak introgression frequency at 58.7 Mb, a peak F_st _at 58.6 Mb, and a peak plant height association at 58.2 Mb that is not quite significant at *P *<0.05 following a Bonferonni correction (Figure 3). Since the causal mutation in *Dw3 *is a copy number variant (CNV) that is unstable and may have arisen quite recently [13], our dataset may not contain linked SNPs in high linkage disequilibrium with the causal CNV. Several regions on either side of the *Dw3 *locus show local peaks in both introgression frequency and F_st_, and co-localize with weak signals of flowering time association.

### The *Dw1 *region on chromosome 9 fractionates into linked QTL

**Figure 4:**
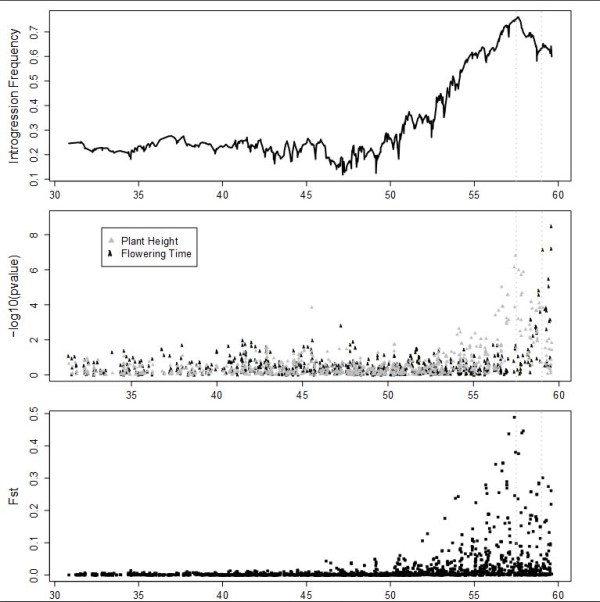
**Introgression frequency, phenotypic associations, and population differentiation in the *Dw1 *region on sorghum chromosome 9**. Panels are the same as in Figures 2 and 3. The locations of putative QTL for plant height and maturity are shown with vertical dashed gray lines.

Chromosome 9, which contains the uncloned *Dw1 *locus, has a peak introgression frequency at 57.6 Mb, a peak F_st _at 57.4 Mb, and a peak plant height association at 57.5 Mb, in close agreement with previous results (Figure 4) [15,16]. A separate cluster of SNPs in the *Dw1 *region associates with flowering time, with a peak at 59.6 Mb. The most significant SNPs for plant height and flowering time are not in significant linkage disequilibrium with each other (r^2 ^= 0.15) and align with two distinct peaks in both introgression frequency and F_st_, strongly suggesting that the *Dw1 *region contains separate loci for plant height and flowering time.

### Chromosome 6 retains little functional variation in SC lines

**Figure 5:**
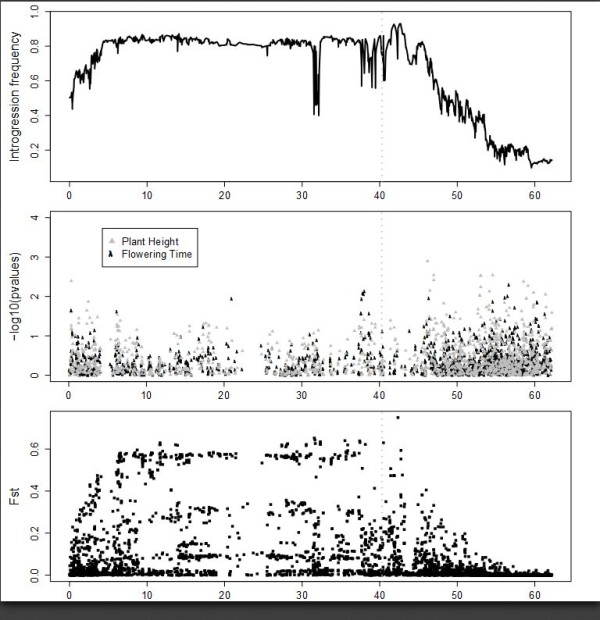
**Introgression frequency, phenotypic associations, and population differentiation on sorghum chromosome 6**. Panels are the same as in Figures 2 to 4. The location of *Ma1 *at 40.3 Mb is shown with a vertical dashed gray line.

Chromosome 6 displays a high introgression frequency and high F_st _across most of its length, even though the known targets on this chromosome are tightly linked: *Ma1 *at 40.3 Mb, and the uncloned *Dw2 *locus several Mb away (Figure 5). The peak introgression frequency and peak F_st _on chromosome 6 apparently correspond to *Dw2 *and not *Ma1 *(Additional File 7), possibly because several independent recessive *ma1 *alleles already exist in the EP lines (R. Klein, personal communication). The choppiness of the introgression frequency between 30 Mb and 43 Mb correlates with a very high proportion of unexpected genotypes in this region, which could result from the existence of an additional, uncharacterized *ma1-dw2 *haplotype in the elite donor. There are no significant phenotypic associations on chromosome 6, suggesting that elite donor introgressions have removed most functional variation for plant height and flowering time on this chromosome in SC lines. Consistent with previous studies reporting a limited number of chromosome 6 haplotypes in SC lines [14,15], we observe the maintenance of high introgression frequency across most of the chromosome, which could be attributed to either a large number of targeted loci or to limited recombination between a few targets. Targets could result from direct selection for plant height and flowering time and/or indirect selection for vigor and adaptation to climatic and soil variation. Regardless of the biological explanation, decreased variation on chromosome 6 is a concern for temperate sorghum breeding. Of the 35 major-effect genes mapped in sorghum as of 2010 [22], seven map to chromosome 6 and four (*d, gc, P, Rs_1_*) have been associated with resistance to biotic stresses including ergot, grain mold, and shoot fly [23-25]. Exotic alleles at these and other unidentified linked loci are at low frequency in SC lines, yet may be useful in future breeding efforts.

### Identification of subpopulation-specific introgression targets

**Figure 6:**
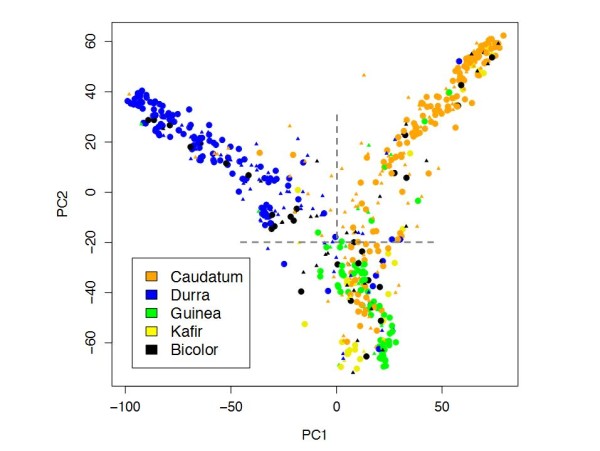
**Sorghum racial identity and subpopulation structure**. PCA plot of the 580 exotic progenitor (EP) lines genotyped in this study. Each dot represents an EP line, colored according to its morphologically-defined race. Larger circles and smaller triangles represent EP lines with more and fewer than 10% unexpected genotypes, respectively. The grey dashed lines indicate the criteria used to assign EP lines to genetic groups for subpopulation-specific introgression mapping.

Sorghum is a crop with strong population sub-division and apparently multiple domestication events [26]. Therefore, we calculated introgression frequencies separately in three subpopulations corresponding to the caudatum (C; *n *= 137), durra (D; *n *= 131), and guinea/kafir (GK; *n *= 122) racial groups. Subpopulations were defined based on genetic criteria in the EP lines (see Methods), which closely match traditional morphological classification (Figure 6). Similar results were obtained when subpopulations are defined based on genetic criteria in the SC lines with or without the three major introgression regions included (Additional File 8). The significance of introgression frequency differences between subpopulations was assessed using permutation (see Methods). We identified multiple subpopulation-specific introgression targets on every chromosome (Additional File 9). Most dramatically, a target at approximately 1 Mb on chromosome 6 is specific to the GK group. In addition to the linked *Ma1-Dw2 *loci and this GK-specific locus, the presence of at least one additional locus on chromosome 6 is necessary to explain the maintenance of high introgression frequency across the chromosome in SC lines of caudatum and durra origin. Introgression frequencies in regions linked to both *Dw1 *and *Dw3 *also vary significantly by subpopulation. Although differences in recombination between subpopulations could theoretically account for such differences, several of these regions also contain phenotypic associations with plant height and flowering time in SC lines, suggesting that they result from subpopulation-specific targets of the SCP. Similarly, a phenotypic association with flowering time at 41.9 Mb on chromosome 5 overlaps with a GK-specific introgression peak (Figure 2, Additional Files 9 and 10). Additional subpopulation-specific targets in regions unlinked to *Dw1, Dw2*, and *Dw3 *that do not overlap with significant phenotypic associations could contain loci for other agronomic traits selected for during the conversion process, including disease resistance, lack of seed dormancy, and overall vigor under temperate conditions.

## Conclusions

The molecular analysis of parents and progeny provides the opportunity for pedigree verification. Our results show that almost one-third of SC lines contain a substantial proportion of unexpected genotypes (>10% of informative markers). We used three complementary approaches - introgression mapping, association mapping, and population differentiation (F_st_) - to characterize the genetic architecture of adaptation to temperate-zone grain production in sorghum. Our novel introgression mapping strategy exploited recombination and selection previously imposed by plant breeders to map three major genomic regions, one of which no longer harbors functional variation in temperate-adapted SC lines. Association mapping confirmed that the *Dw1 *region contains separate QTL for plant height and flowering time. Significant differences in introgression frequency between subpopulations strongly suggest the existence of additional uncharacterized loci that affect plant height and flowering time in sorghum.

Linkage disequilibrium between at least four targeted loci on chromosome 6 has led to the introgression of a single elite haplotype across most of this chromosome in the majority of lines examined. Chromosome 6 contains roughly 10% of sorghum genes, for which very little functional diversity has been exploited for temperate sorghum breeding. This lack of diversity undoubtedly limits adaptive potential, especially for complex traits including resistance to abiotic and/or biotic stress. Increasing gene flow and recombination between tropical and temperate sorghum varieties and haplotypes will help unlock the genetic potential of this stress-tolerant crop to meet our rising demand for food, feed, and fuel in an era of increasing climatic volatility.

## Methods

### Plant materials, DNA extraction, and quantification

Seed for SC lines was obtained from the USDA-ARS Cropping Systems Research Laboratory (Lubbock, TX, USA) and seed for EP lines was obtained from the National Plant Germplasm System (NPGS [27]). Information on the geographic origins and morphological racial classification of each SC line were obtained from Texas A&M University (Additional File 1). Three independent seed sources of the elite donor BTx406 were obtained from the NPGS (PI 656020), the USDA-Cropping Systems Research Laboratory, and Texas A&M University. Genomic DNA was extracted from etiolated seedlings approximately 3 days after germination using a modified CTAB protocol [28] and quantified using PicoGreen (Invitrogen, NY, USA).

### SNP library creation

Libraries were prepared using a protocol modified from Poland *et al*. 2012 [18]. Genomic DNA (approximately 250 ng) was double digested with either *PstI*-HF and *BfaI *or *PstI*-HF and *HinP1I *at 37°C for 2 h with heat inactivation at 80°C for 20 min. Digested DNA was ligated to two separate adapters using T4 ligase with 1mM ATP. The first adapter contains the Illumina forward sequencing primer, one of 96 unique barcodes, and the *PstI *overhang. The second adapter contains the Illumina reverse sequencing primer and the overhang for either *BfaI *or *HinP1I*. The full list of adapters is shown in Additional File 11. Ligation reactions were held at 25°C for 2 h followed by heat inactivation at 65°C for 20 min. Pooled DNA from 96 barcoded libraries was cleaned using a 2:1 ratio of AmpureXP Beads (Beckman Coulter, CA, USA) to DNA solution using a Magnetic Particle Concentrator (Invitrogen, NY, USA) with two washes in 95% ethanol and resuspension in elution buffer (EB; 10mM Tris). Cleaned DNA pools were amplified using Illumina primers in a 2X PhusionHF Master Mix (New England Biolabs, MA, USA) with cycler conditions as follows: 98°C 30 s, 15 cycles (98°C 10 s, 68°C 30 s, 72°C 30 s), 72°C 5 min. Samples were run on agarose gels to confirm the presence of a genomic smear and cleaned a second time with AMPure beads. Amplified DNA sizes and relative concentrations were assessed using an Agilent Bioanalyzer 2100 and Agilent DNA1000 Kit (Agilent Technologies Inc., CA, USA) and PicoGreen. The two separately digested samples were combined in equimolar concentrations and diluted to 10 nM in library buffer (EB + 0.05% Tween-20) and submitted to the W.M. Keck Center at the University of Illinois for single-end sequencing on the Illumina HiSeq2000. The Keck Center performed an additional qPCR assay on each library to adjust concentrations before sequencing.

### Genotype data analysis

SNPs were called from Illumina fastq files using the TASSEL GBS pipeline [20]. Only 64 bp tags present at least 10 times in the dataset were considered. Alignment was performed using BWA [29] with the default settings. Inbred lines and SNPs with >95% missing data were discarded. SNPs were not filtered by minor allele frequency, as rare SNPs are especially useful for inferring introgression events between pairs of lines (Figure 1B). Heterozygous genotypes accounted for 0.3% of the total dataset. Partial imputation using the TASSEL GBS pipeline reduced the proportion of missing data from approximately 66% to approximately 20%. For the association and F_st _analyses, the remaining missing data were imputed using BEAGLE. This yielded substantially fewer unexpected genotypes than direct imputation using BEAGLE without prior partial imputation (data not shown).

### Mapping unanchored contigs in the sorghum genome

We defined a set of 213 SNPs from 31 unanchored contigs that had at least 20 introgression calls and an introgression frequency of at least 10%, and calculated linkage disequilibrium (r^2^) between introgression scores in the 213 unanchored SNPs and our complete set of 46,062 SNPs with introgression scores in the 390 SC-EP pairs that were placed on the sorghum physical map (V1.0 [30]). Most (181) of the unanchored SNPs mapped uniquely to a single chromosome, with a mean of 8.4 mapped SNPs tied for the highest r^2 ^across a mean physical distance of 9.1 Mb (Additional File 6).

### Calculation of introgression scores and frequencies

For each SNP, an introgression was scored as either present (1), when a genotype was shared between the SC line and the donor line, or absent (0), when a genotype was shared between the SC line and its EP line. Missing data for presence/ absence of introgressions were inferred as the mean of each flanking marker weighted by its physical distance (Figure 1D). Missing data proximal and distal to the first and last informative markers on a chromosome, respectively, were assigned the value of the closest informative marker. Once missing data were imputed, introgression frequencies were calculated at each SNP as the percentage of SC lines with an introgression.

### Subpopulation assignment and permutations

Principal component analysis (PCA) was performed in EP lines in R [31] using the prcomp() function and a dataset of 22,203 SNPs with minor allele frequencies >10% in the set of 1,160 SC and EP lines (580 pairs). EP lines were assigned to subpopulations using values for PC1 and PC2 as follows: (1) lines with PC2 <-20 were assigned to the guinea/kafir (GK) group; (2) lines with PC2 >-20 and PC1 >0 were assigned to the caudatum (C) group; (3) lines with PC2 >-20 and PC1 <0 were assigned to the durra (D) group. Introgressed regions excluded from the analysis in Additional File 8 were defined as locations <55 Mb on chromosome 6, >50 Mb on chromosome 7, and >50 Mb on chromosome 9. Significance of subpopulation differences in introgression frequency was assessed by randomly assigning SC lines to subpopulations of equivalent size (137, 131, and 122 individuals) and calculating introgression frequencies across the three permuted subpopulations. For each permutation, the maximum range of introgression frequencies across the three subpopulations was recorded for each chromosome. Two hundred permutations were performed and α was set to 0.05.

### Phenotypic data and association mapping

The 580 genotyped SC lines were grown in 6 m plots with 0.76 m row spacing in Urbana, IL in the summers of 2011 and 2012 and phenotyped for plant height and flowering time. Plant height was measured as the distance (cm) from the ground to the penultimate or 'pre-flag' leaf on one representative plant per row. Flowering time was measured as the time (days from planting) at which 50% of the plants in the row had initiated anthesis. Phenotypic data from each year were normalized and the mean normalized value across all years was used for association mapping. The GAPIT package in R [32] was used to conduct marker-trait associations using the default parameters. Markers included all SNPs discovered in this study with minor allele frequencies ≥10%. Missing SNP data were imputed using BEAGLE.

### Data availability

Raw genotyping-by-sequencing read data have been deposited in the Sequenced Read Archive [SRA: SRP022956]. Introgression scores have been included in a table as Additional File 12.

## Abbreviations

EP: exotic progenitor; GBS: genotyping-by-sequencing; SC: sorghum conversion; SNP: single nucleotide polymorphism.

## Competing interests

The authors declare that they have no competing interests.

## Authors' contributions

PB conceived the project idea. CT, JM, RH, and PB performed data collection and analysis. PB and CT wrote the manuscript.

## Supplementary Material

Additional File 1 **Table S1. SC and EP lines used in this study**. Principal components analysis in the EP lines was used to assign SC-EP pairs to subpopulations. Plant height and flowering time phenotypes used for association mapping in the SC lines are also provided. Click here for file

Additional File 2 **Figure S1. Enzyme effects on SNP output**. Combining two double digests (*PstI*-HF/*HinP1I *and *PstI*-HF/*BfaI*) nearly doubles the number of SNPs called per sample over one double digest (*Pst1*-HF/*HinP1I*). Click here for file

Additional File 3 **Figure S2. Principal Component Analysis (PCA) of BTx406 seed source libraries**. Twenty-eight libraries were created for BTx406 seed from three different sources (GRIN, Cornell, and Lubbock). The three outlier libraries from the GRIN collection were removed due to low concordance. Click here for file

Additional File 4 **Figure S3. Introgression maps for 390 SC lines**. Click here for file

Additional File 5 **Table S2. Number and percentage of introgressed, unexpected, and informative markers for each SC-EP pair**. Click here for file

Additional File 6 Table S3. Physical map positions of unanchored SNPs Click here for file

Additional File 7 **Figure S4**. Introgression frequency, phenotypic associations, and population differentiation in the *Ma1-Dw2 *region on sorghum chromosome 6. Panels are the same as in Figures 3 to 6. The locations of *Ma1 *at 40.3 Mb is shown with a vertical dashed gray line. Click here for file

Additional File 8 **Figure S5**. PCA of SC lines with and without SNPs in the three major introgressed regions. Click here for file

Additional File 9 **Figure S6**. Subpopulation-specific introgression frequencies. Click here for file

Additional File 10 **Table S4**. Phenotypic associations with plant height and flowering time in 580 SC lines. Click here for file

Additional File 11 **Table S5**. List of barcoded adapters used in library preparation. Click here for file

Additional File 12 **Table S6**. Raw introgression scores. Click here for file
