# Supplementary figures and images for: Retrospective genomic analysis of sorghum adaptation to temperate-zone grain production
Source: Genome Biol. 2013 Jun 26;14(6):R68. doi: 10.1186/gb-2013-14-6-r68 (PMC3706989; doi:10.1186/gb-2013-14-6-r68)

## Enzyme Effects on SNP Output

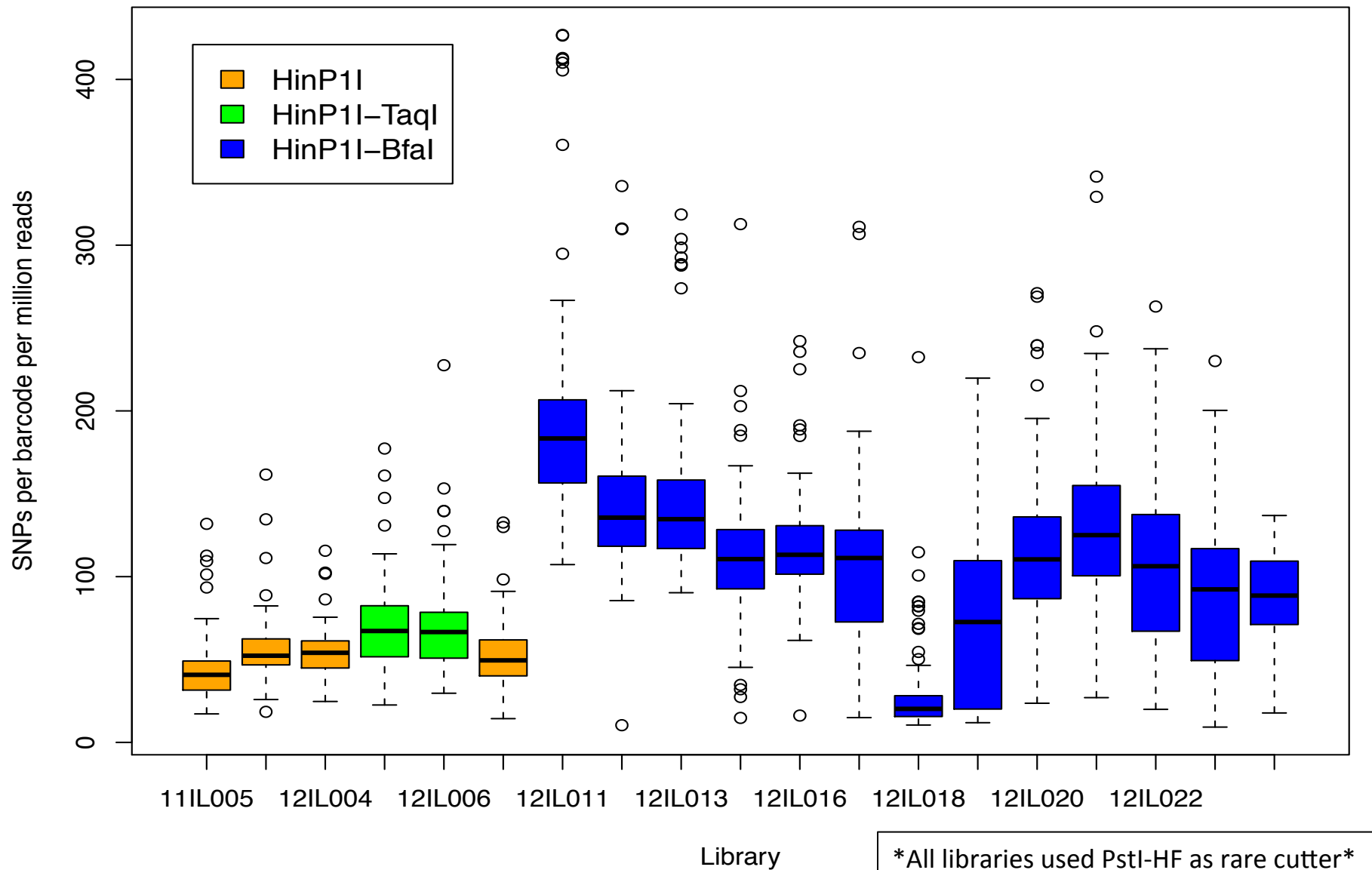

\*All libraries used PstI-HF as rare cutter\*

Supplement: Additional File 2 — Figure S1. Enzyme effects on SNP output. Combining two double digests (PstI-HF/HinP1I and PstI-HF/BfaI) nearly doubles the number of SNPs called per sample over one double digest (Pst1-HF/HinP1I). [file gb-2013-14-6-r68-S2.PDF]

## 20 BTx406 wells: 7 GRIN, 6 IGD, 7 Woodfin

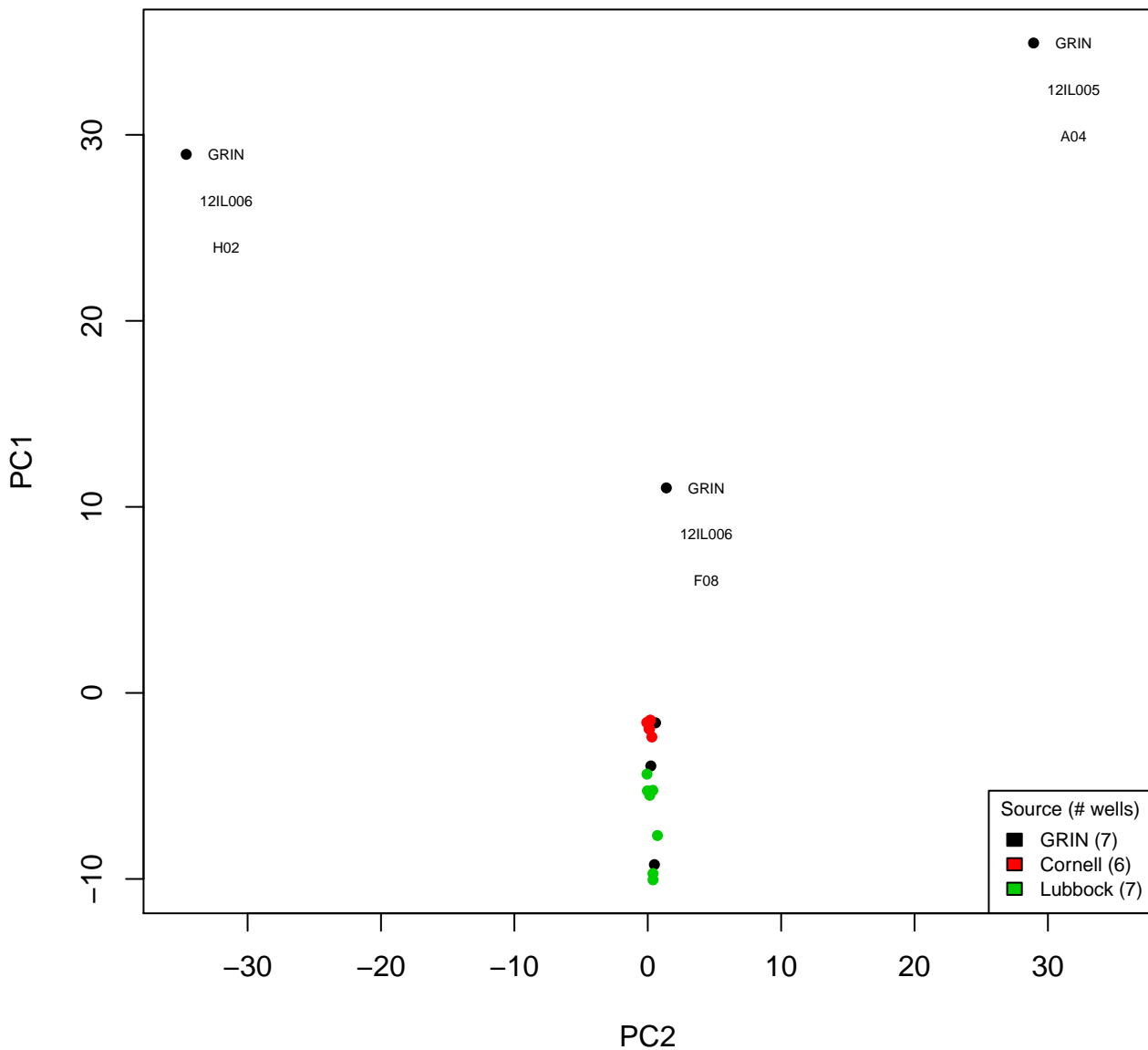

Supplement: Additional File 3 — Figure S2. Principal Component Analysis (PCA) of BTx406 seed source libraries. Twenty-eight libraries were created for BTx406 seed from three different sources (GRIN, Cornell, and Lubbock). The three outlier libraries from the GRIN collection were removed due to low concordance. [file gb-2013-14-6-r68-S3.PDF]

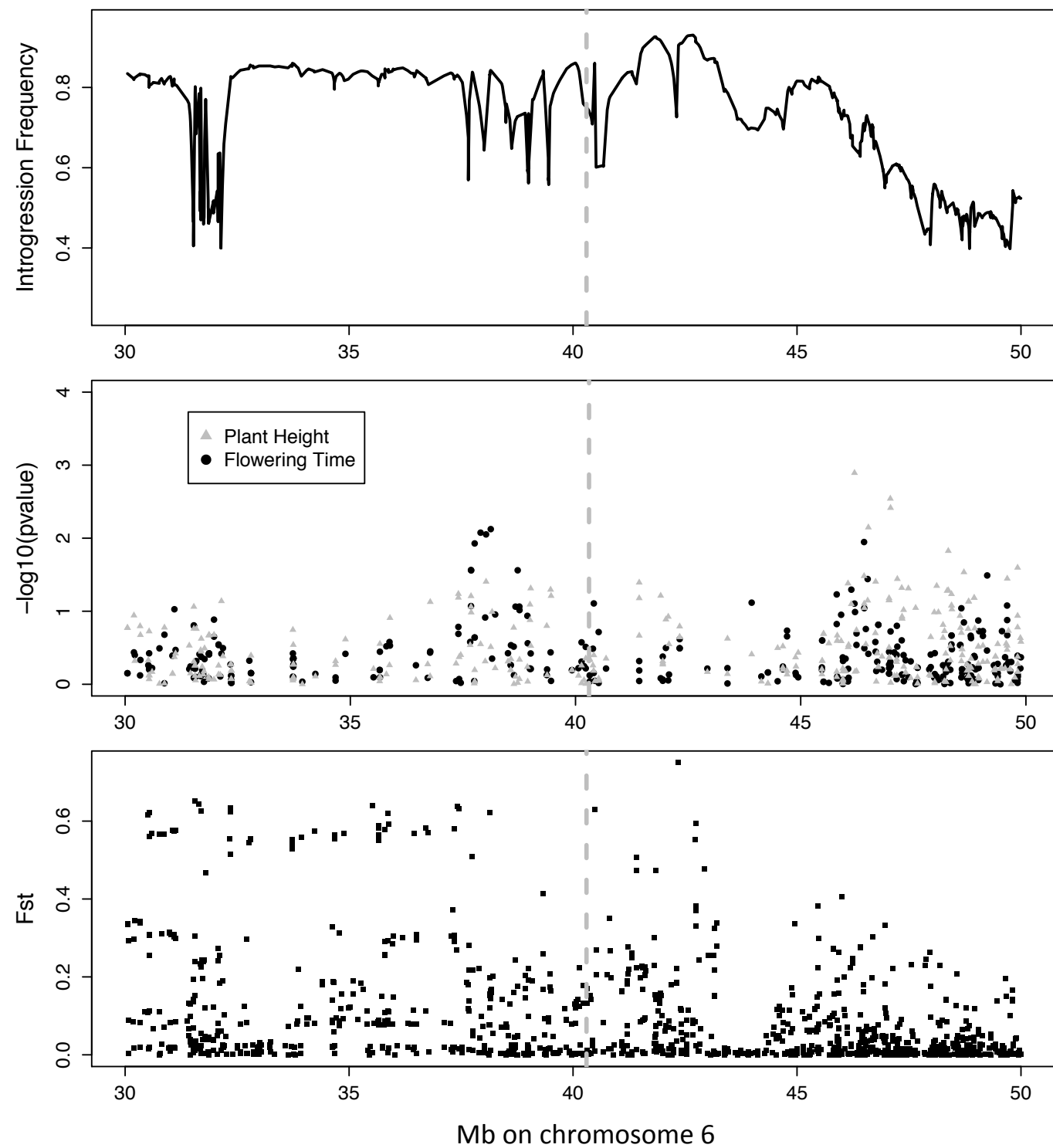

Supplement: Additional File 7 — Figure S4. Introgression frequency, phenotypic associations, and population differentiation in the Ma1-Dw2 region on sorghum chromosome 6. Panels are the same as in Figures 3 to 6. The locations of Ma1 at 40.3 Mb is shown with a vertical dashed gray line. [file gb-2013-14-6-r68-S7.PDF]

SC lines,  
whole genome

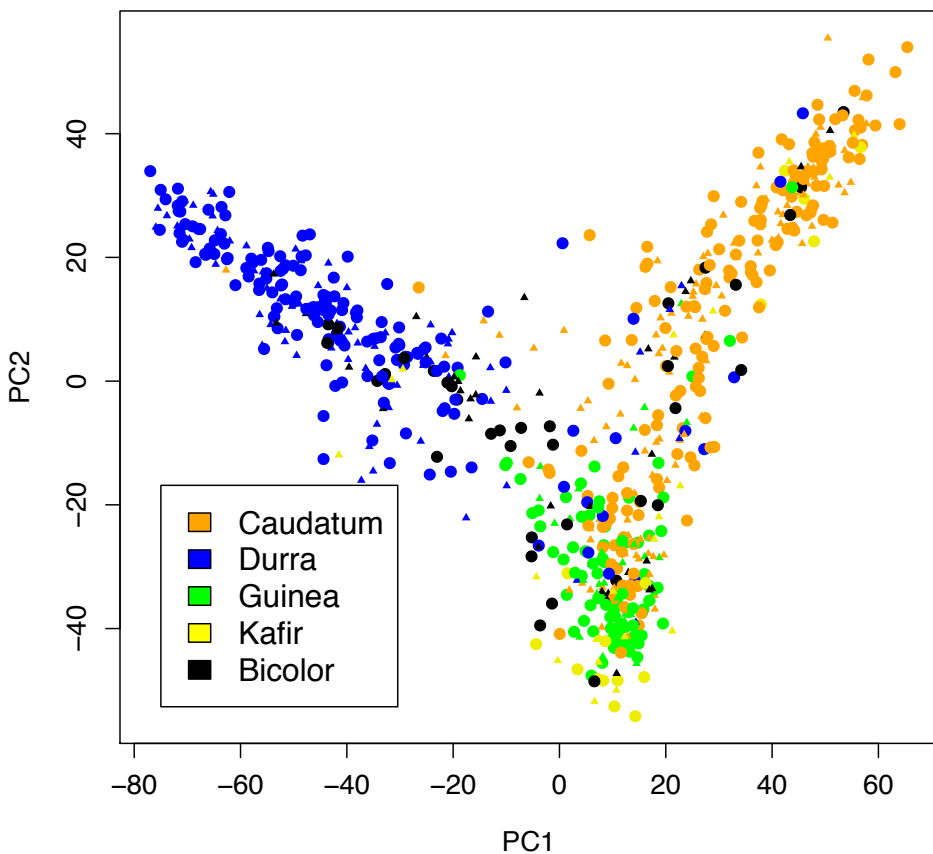

SC lines,  
excluding introgressed regions

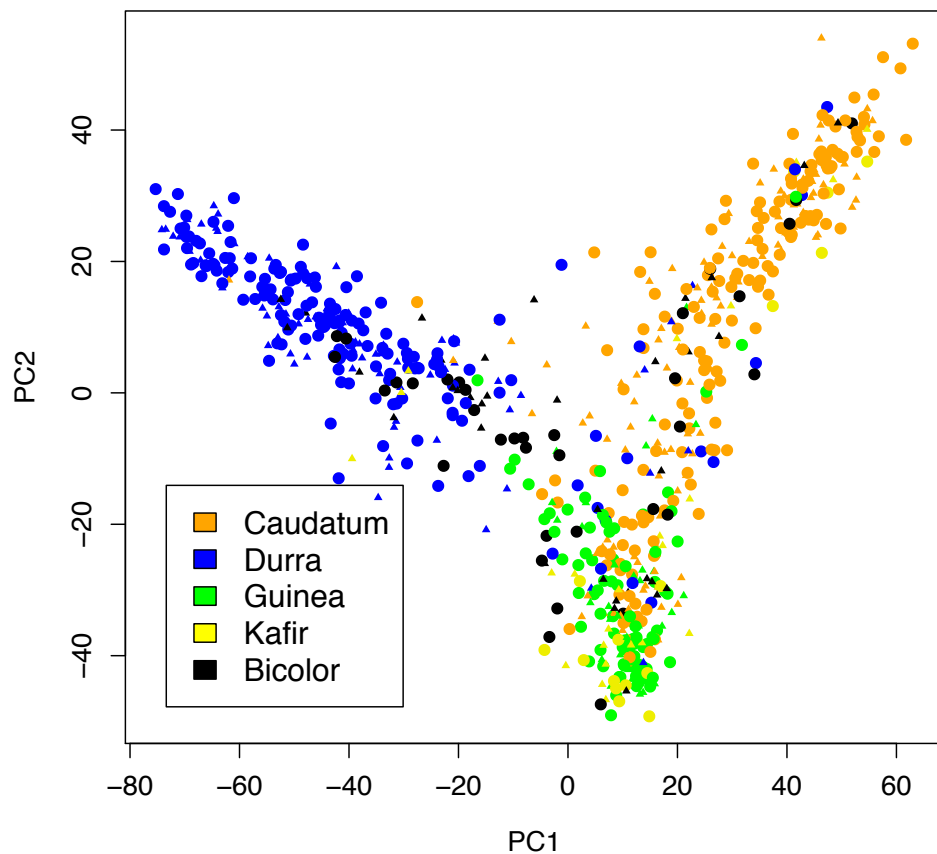

Supplement: Additional File 8 — Figure S5. PCA of SC lines with and without SNPs in the three major introgressed regions. [file gb-2013-14-6-r68-S8.PDF]
