# Supplementary material for: Retrospective genomic analysis of sorghum adaptation to temperate-zone grain production
Source: Genome Biol. 2013 Jun 26;14(6):R68. doi: 10.1186/gb-2013-14-6-r68 (PMC3706989; doi:10.1186/gb-2013-14-6-r68)
Supplement: Additional File 4 — Figure S3. Introgression maps for 390 SC lines. [file gb-2013-14-6-r68-S4.ZIP › AdditionalFile4_FigureS3.pdf]

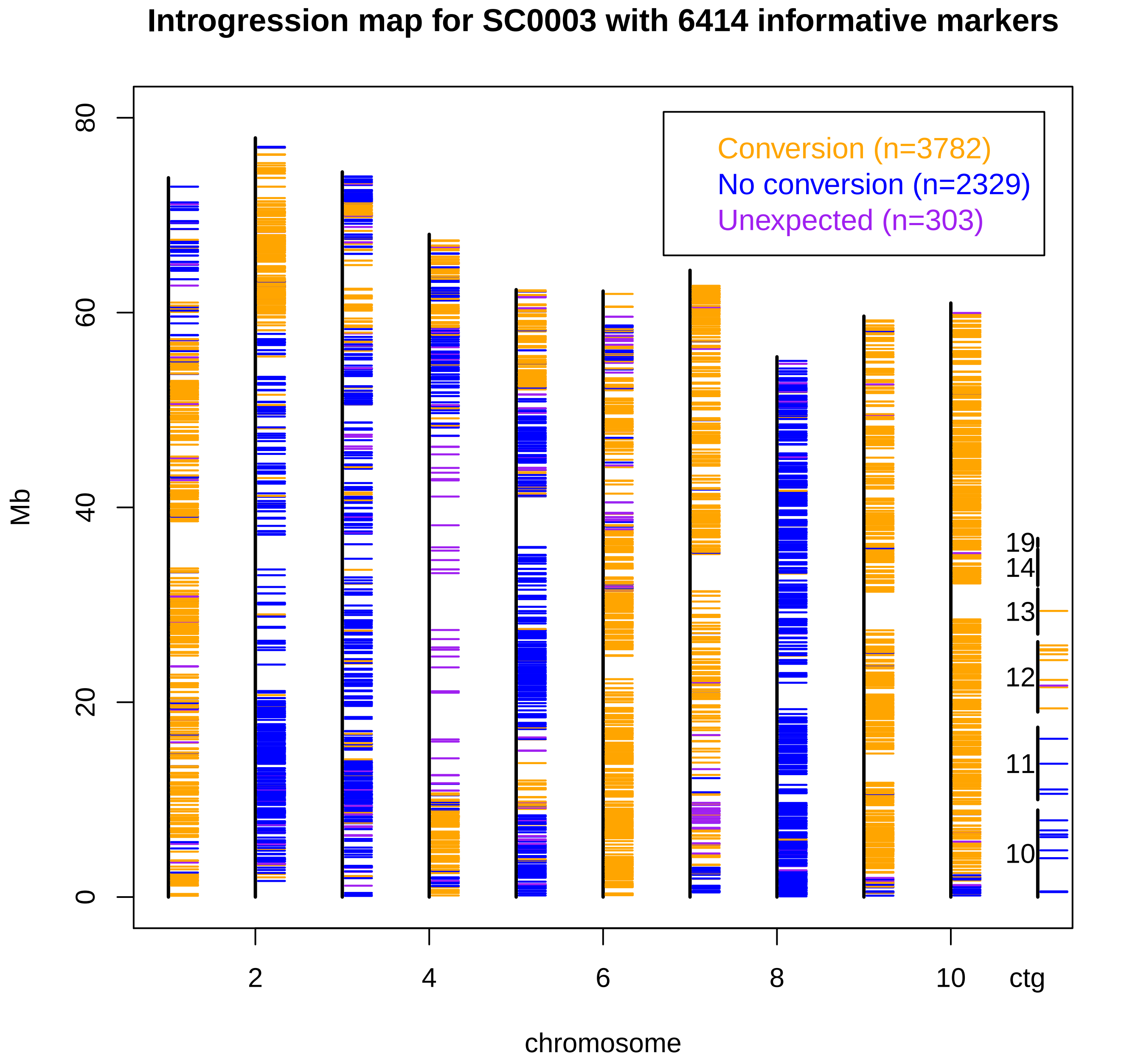

Introgression map for SC0003 with 6414 informative markers
Conversion (n=3782)
No conversion (n=2329)
Unexpected (n=303)
Mb
0
20
40
60
80
chromosome
2
4
6
8
10
ctg
19
14
13
12
11
10

Introgression map for SC0006 with 8018 informative markers

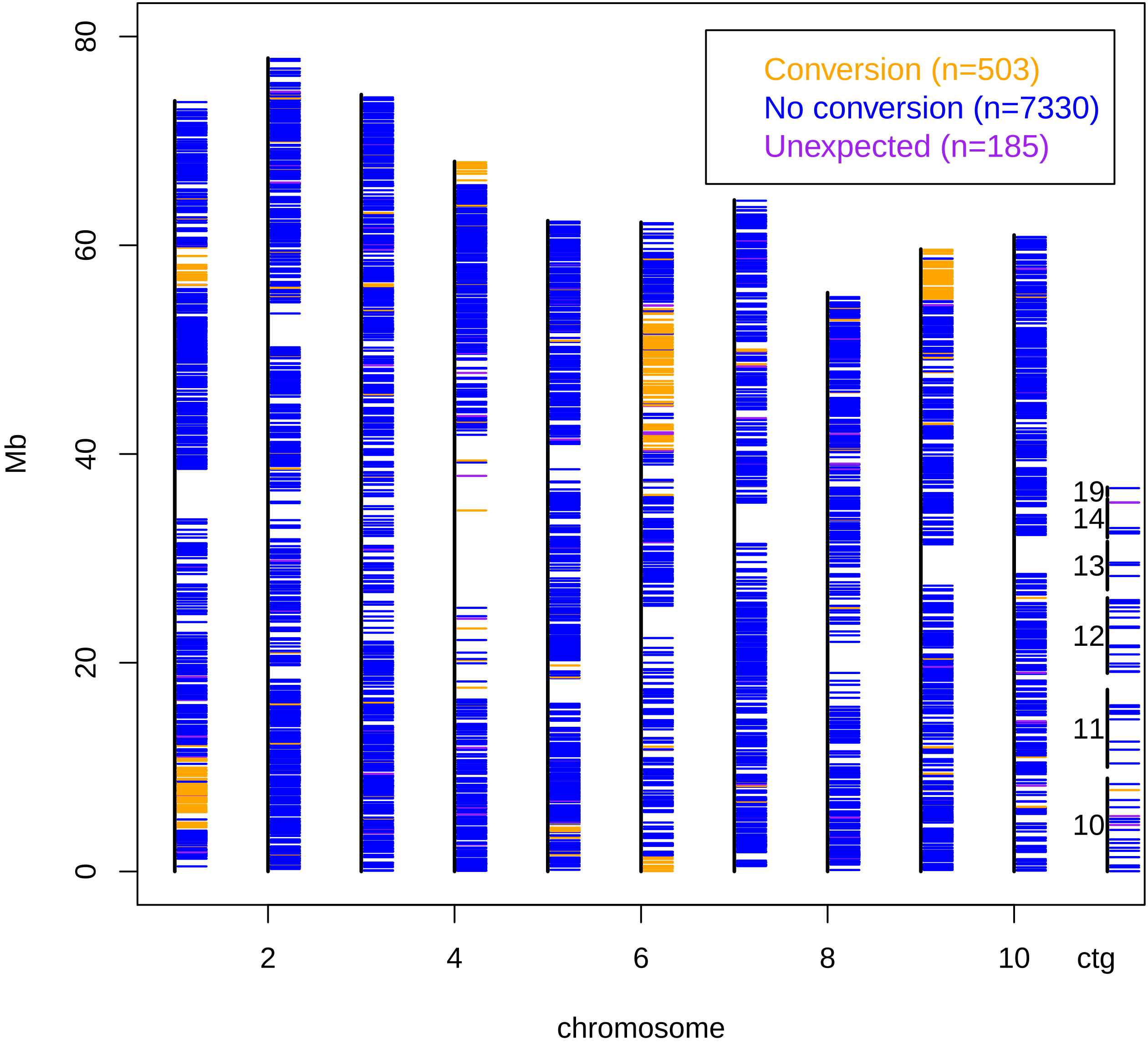

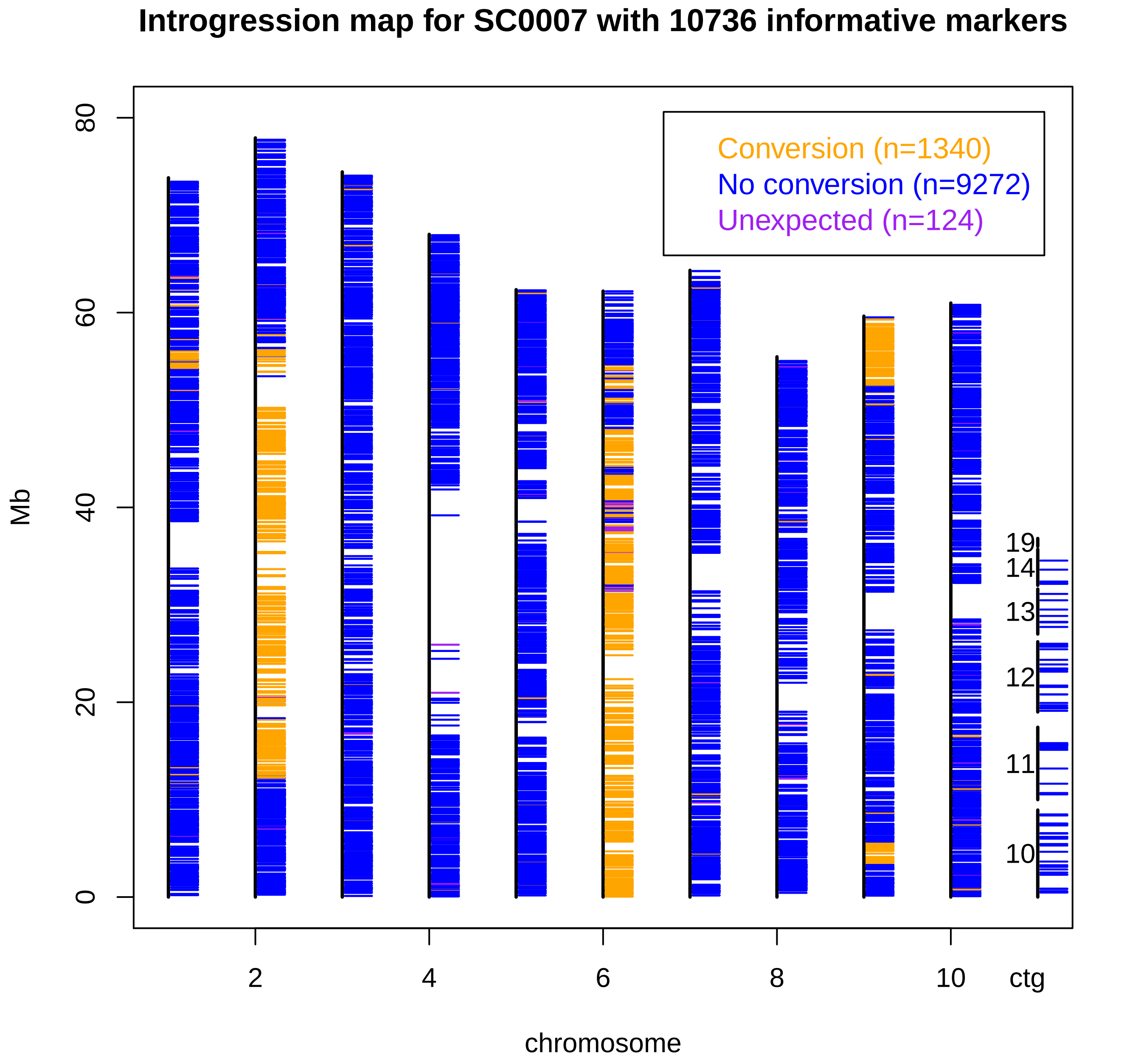

Introgression map for SC0007 with 10736 informative markers
Conversion (n=1340)
No conversion (n=9272)
Unexpected (n=124)
Mb
0
20
40
60
80
2
4
6
8
10
ctg
19
14
13
12
11
10
chromosome

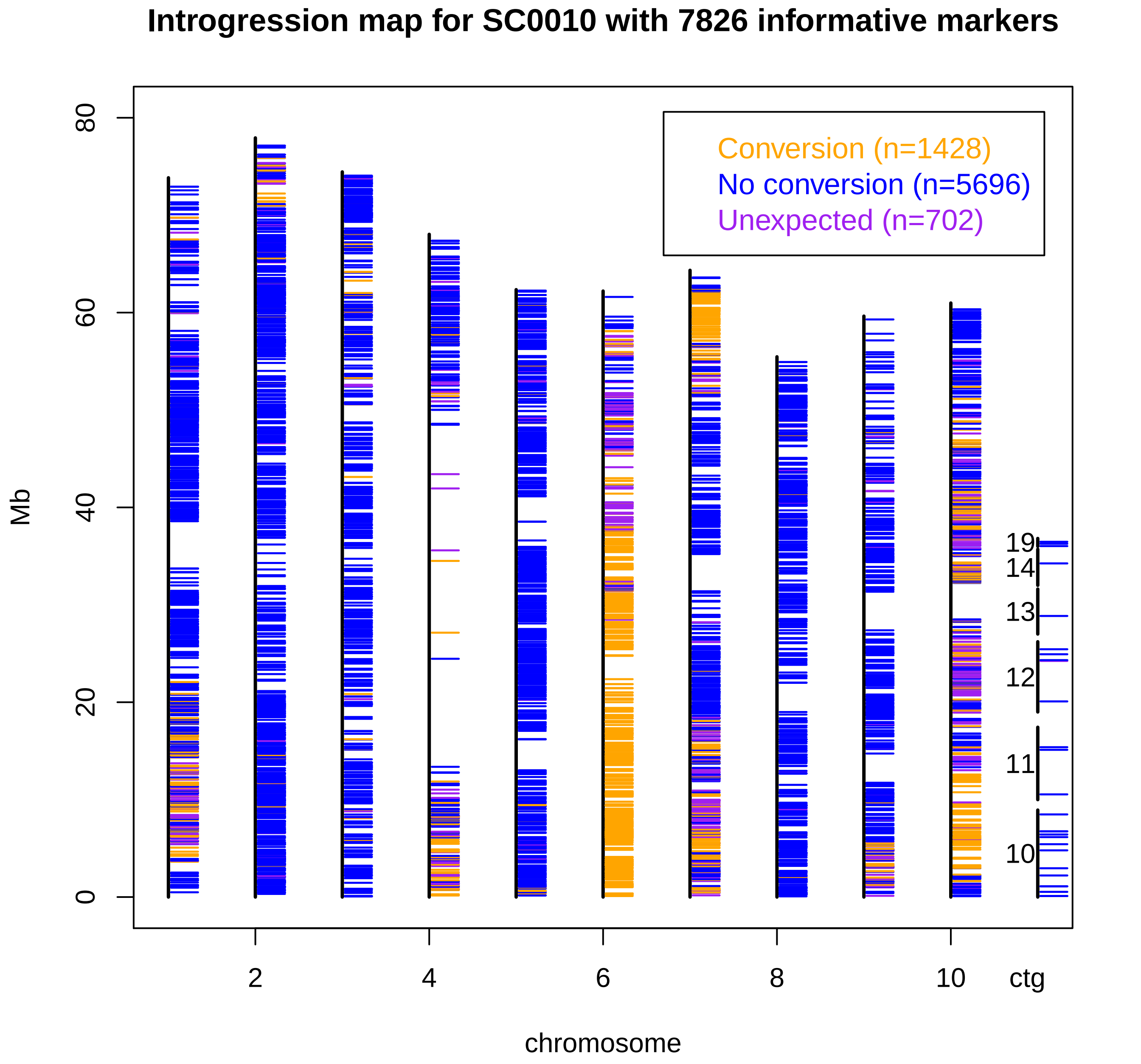
Introgression map for SC0010 with 7826 informative markers
Conversion (n=1428)
No conversion (n=5696)
Unexpected (n=702)
Mb
0
20
40
60
80
chromosome
2
4
6
8
10
ctg
19
14
13
12
11
10

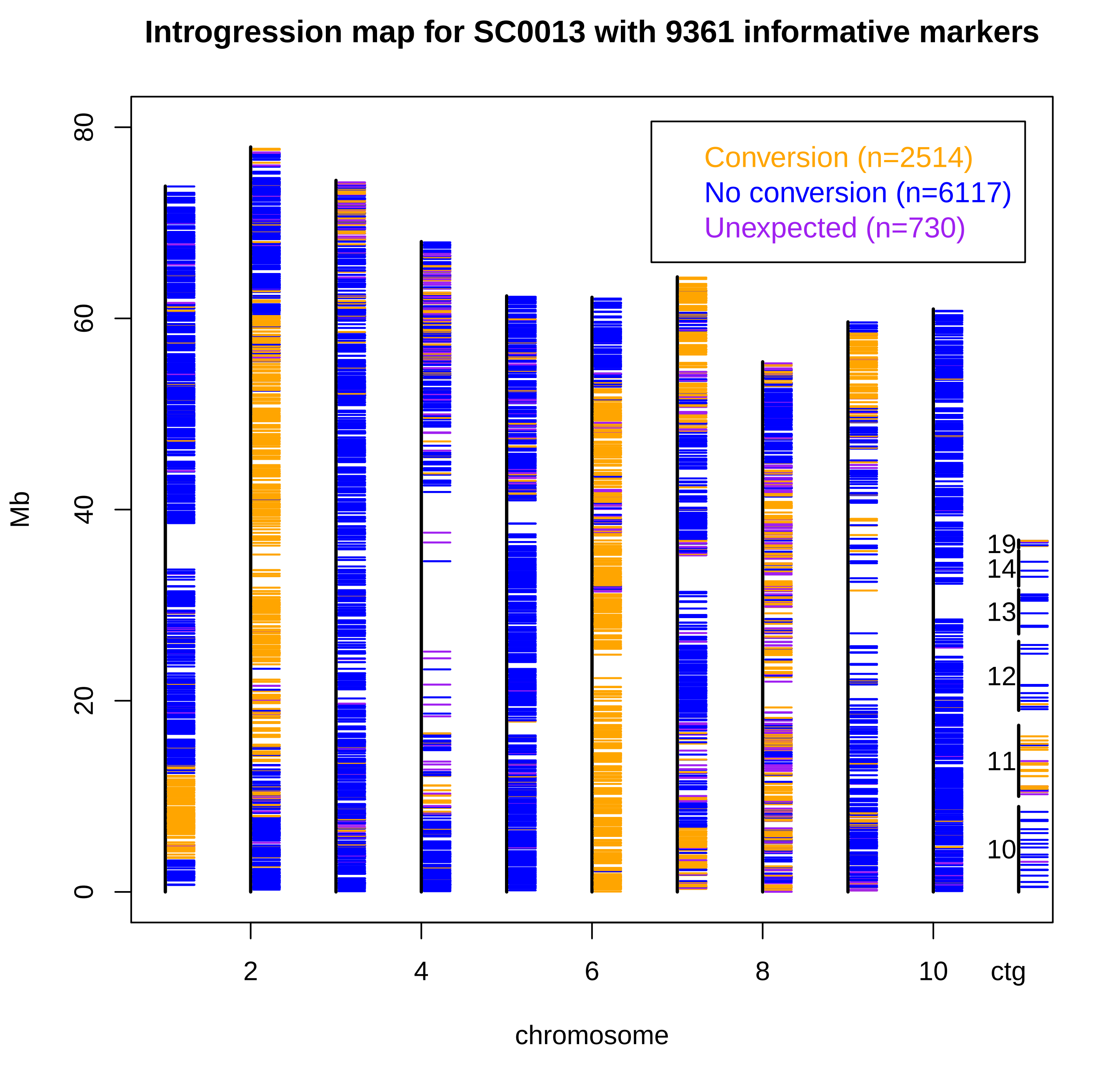

Introgression map for SC0013 with 9361 informative markers
Conversion (n=2514)
No conversion (n=6117)
Unexpected (n=730)
Mb
0
20
40
60
80
chromosome
2
4
6
8
10
ctg
19
14
13
12
11
10

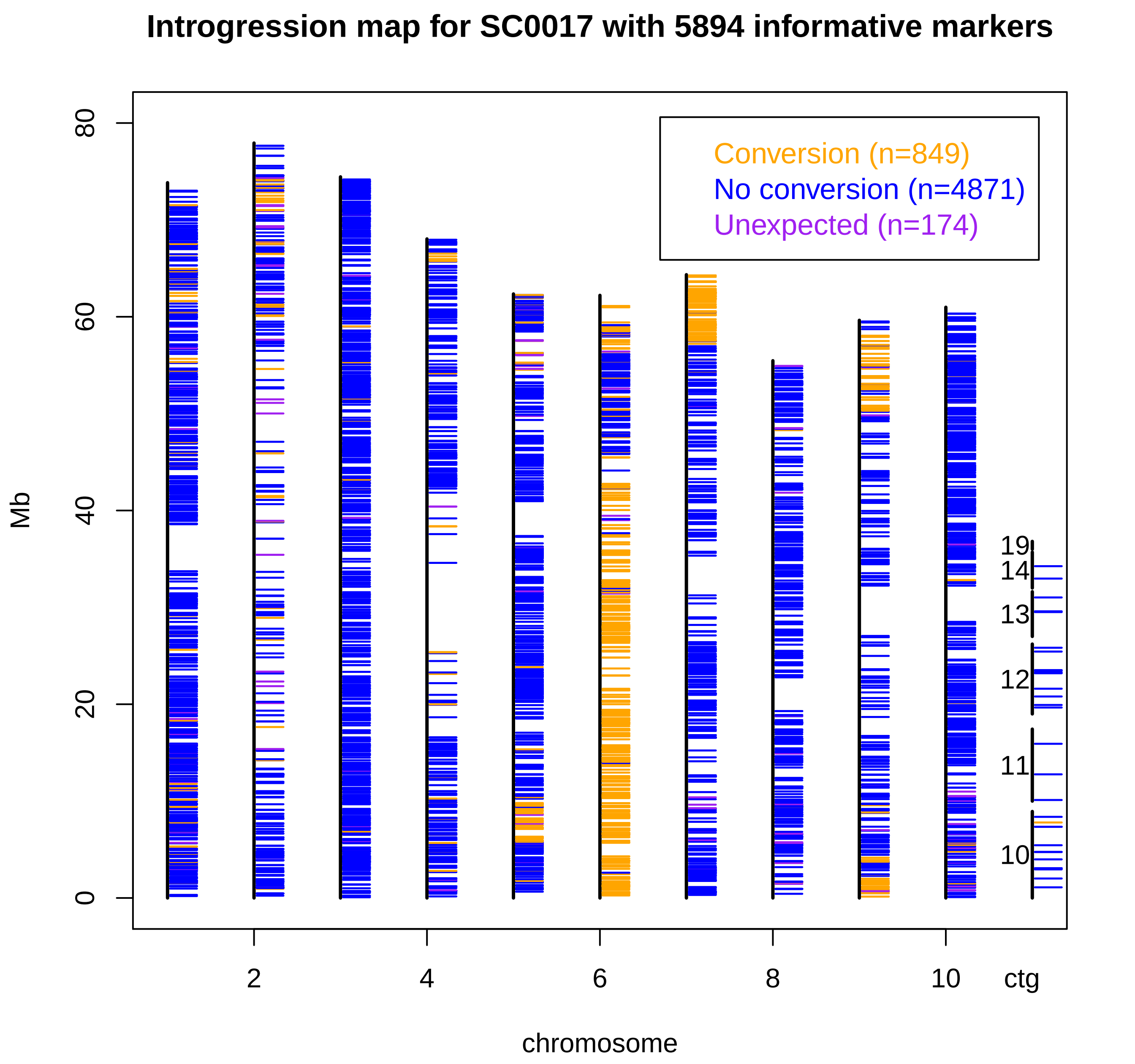
Introgression map for SC0017 with 5894 informative markers
Conversion (n=849)
No conversion (n=4871)
Unexpected (n=174)
Mb
0
20
40
60
80
chromosome
2
4
6
8
10
ctg
19
14
13
12
11
10

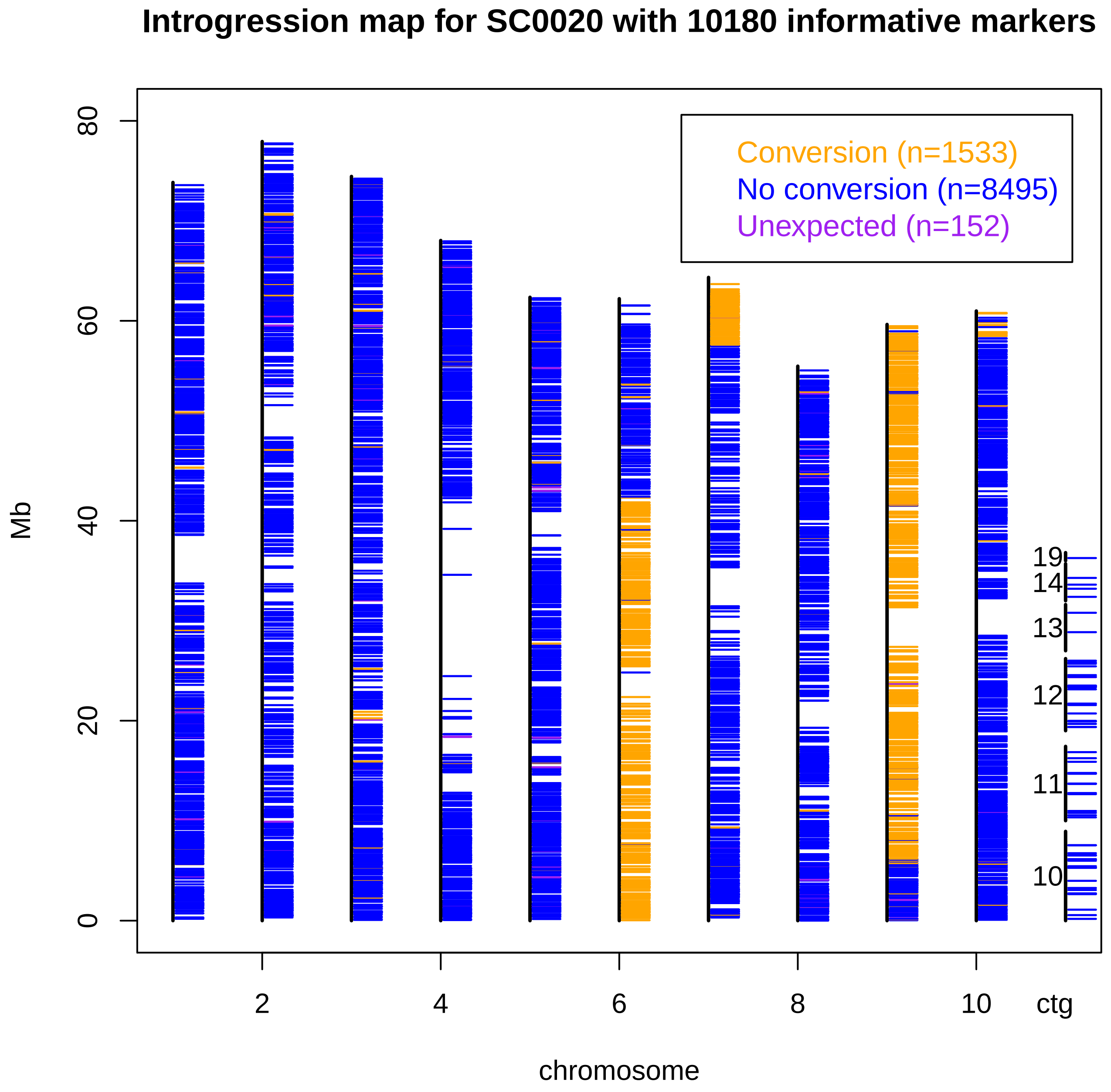

Introgression map for SC0020 with 10180 informative markers
Conversion (n=1533)
No conversion (n=8495)
Unexpected (n=152)
Mb
0
20
40
60
80
2
4
6
8
10
ctg
19
14
13
12
11
10
chromosome

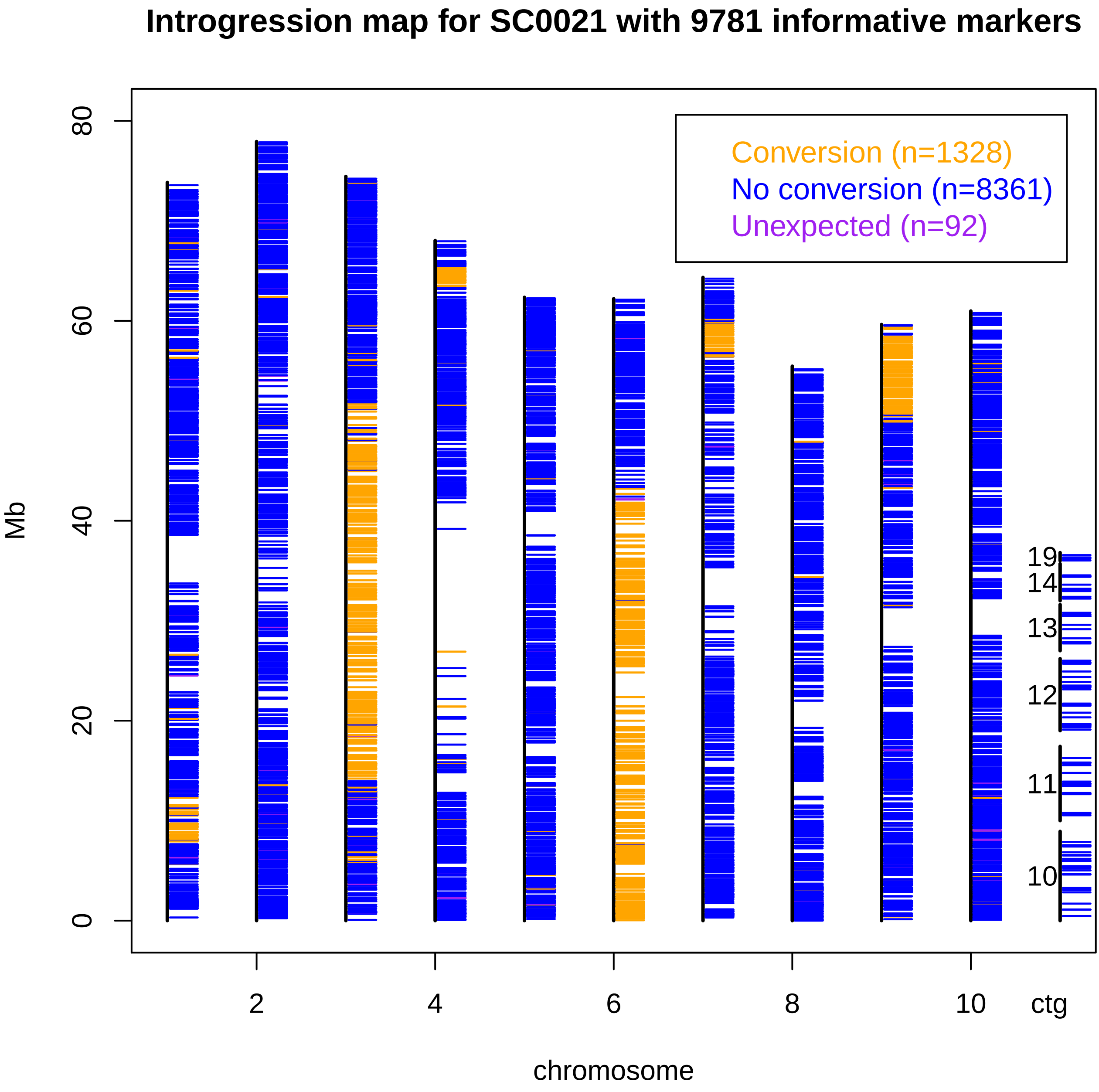
Introgression map for SC0021 with 9781 informative markers
Conversion (n=1328)
No conversion (n=8361)
Unexpected (n=92)
Mb
0
20
40
60
80
chromosome
2
4
6
8
10
ctg
19
14
13
12
11
10

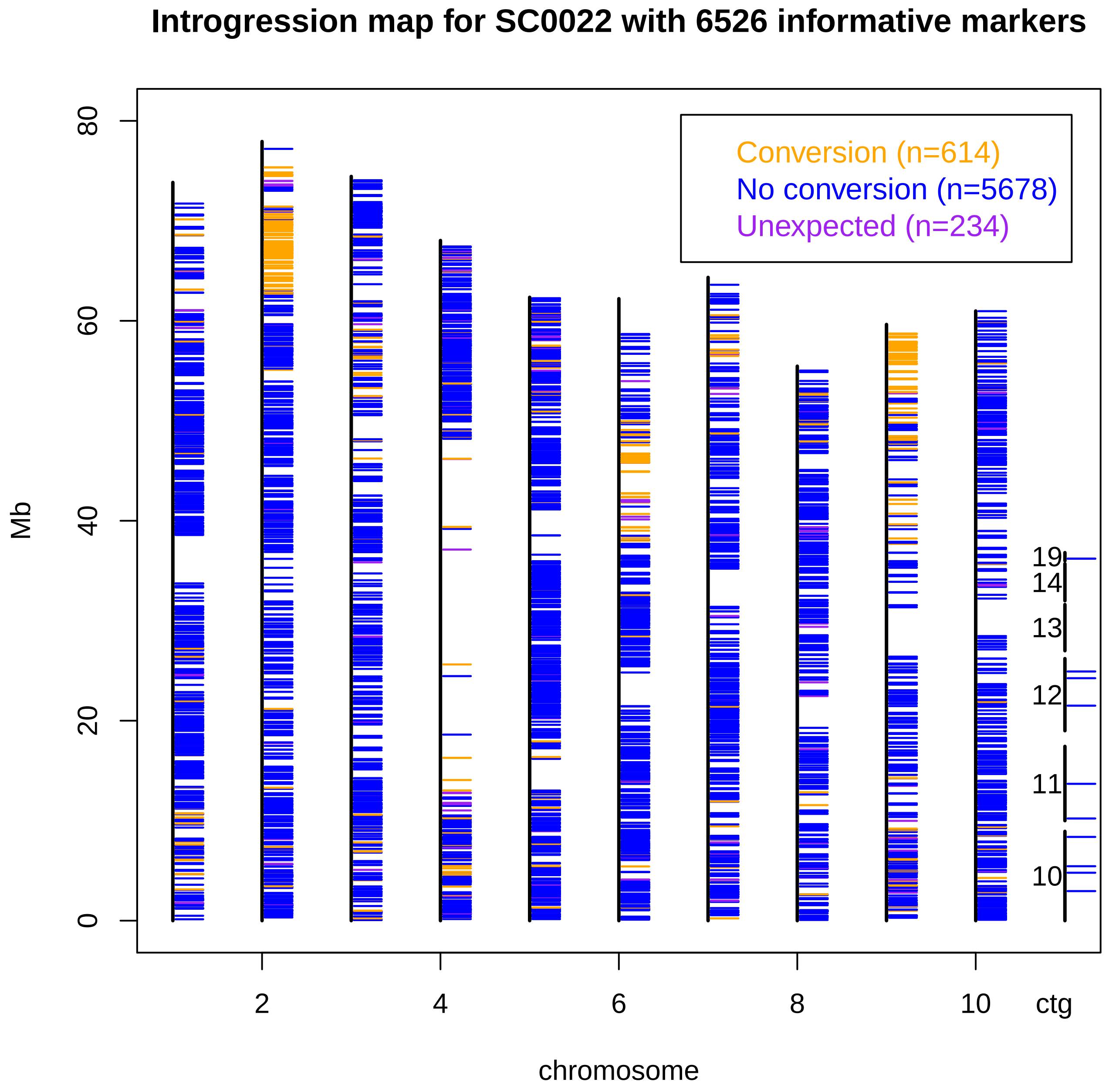

Introgression map for SC0022 with 6526 informative markers
Conversion (n=614)
No conversion (n=5678)
Unexpected (n=234)
Mb
0
20
40
60
80
chromosome
2
4
6
8
10
ctg
19
14
13
12
11
10

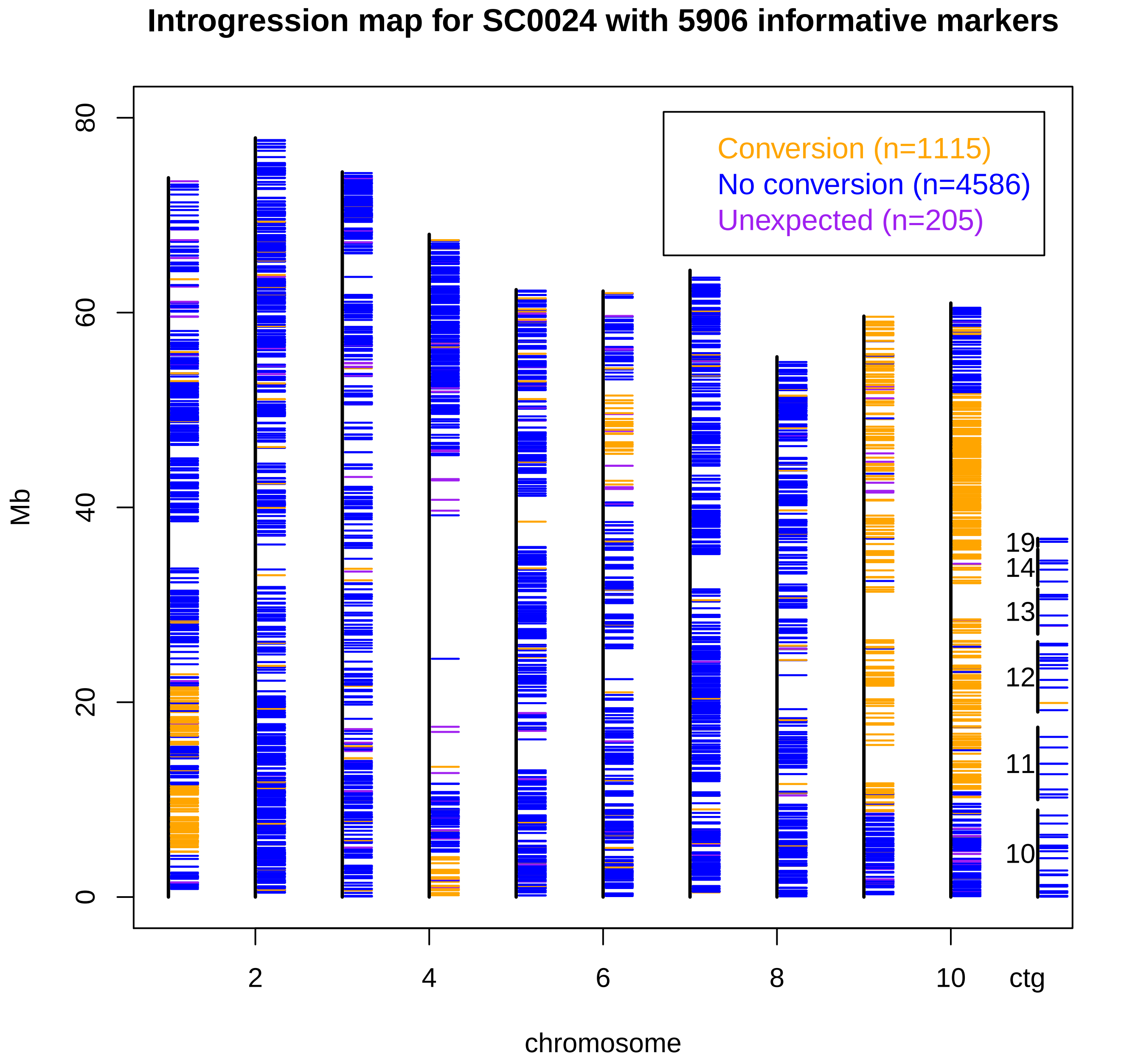

Introgression map for SC0024 with 5906 informative markers
Conversion (n=1115)
No conversion (n=4586)
Unexpected (n=205)
Mb
0
20
40
60
80
2
4
6
8
10
ctg
19
14
13
12
11
10
chromosome

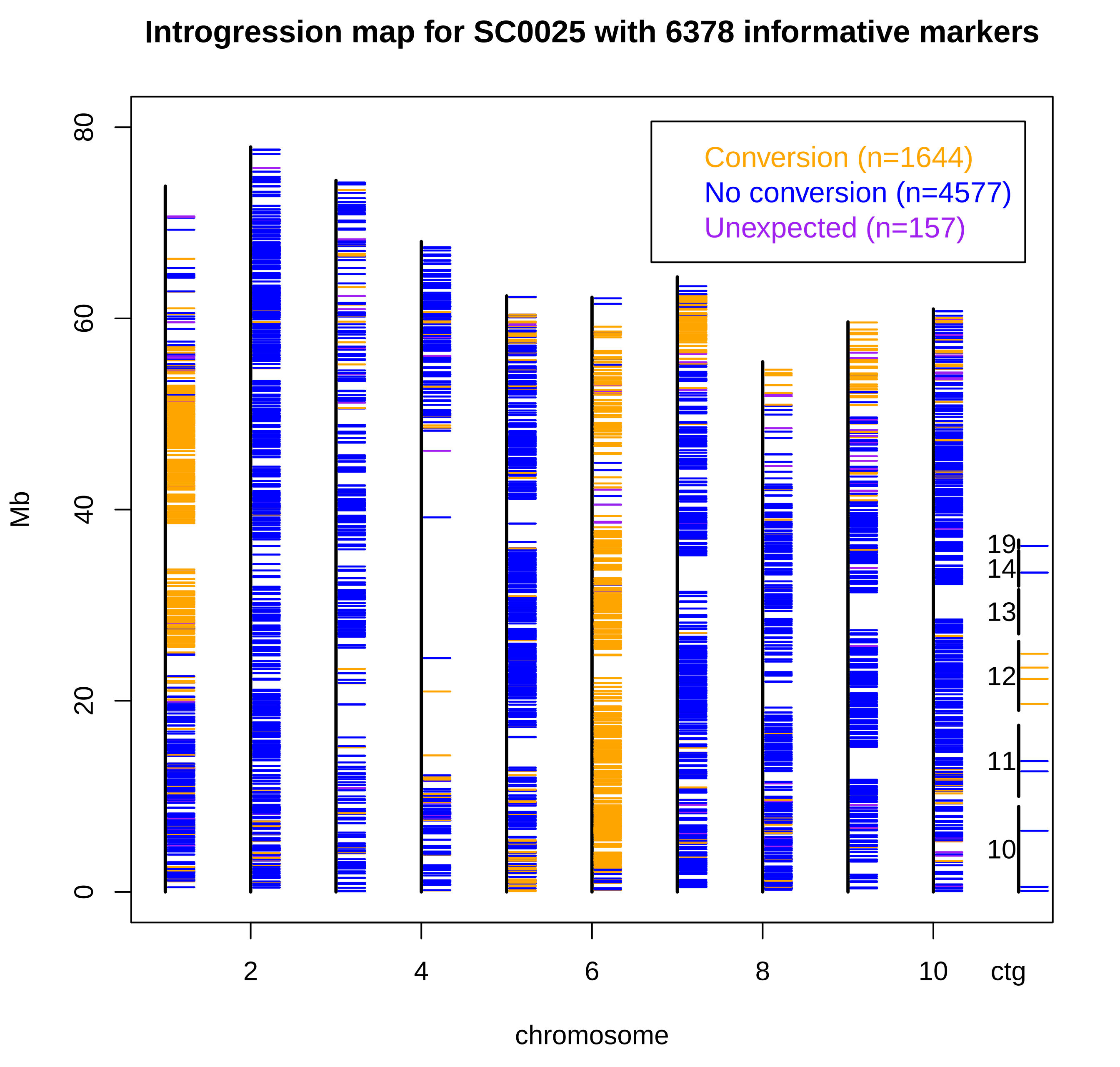

Introgression map for SC0025 with 6378 informative markers
Conversion (n=1644)
No conversion (n=4577)
Unexpected (n=157)
Mb
0
20
40
60
80
chromosome
2
4
6
8
10
ctg
19
14
13
12
11
10

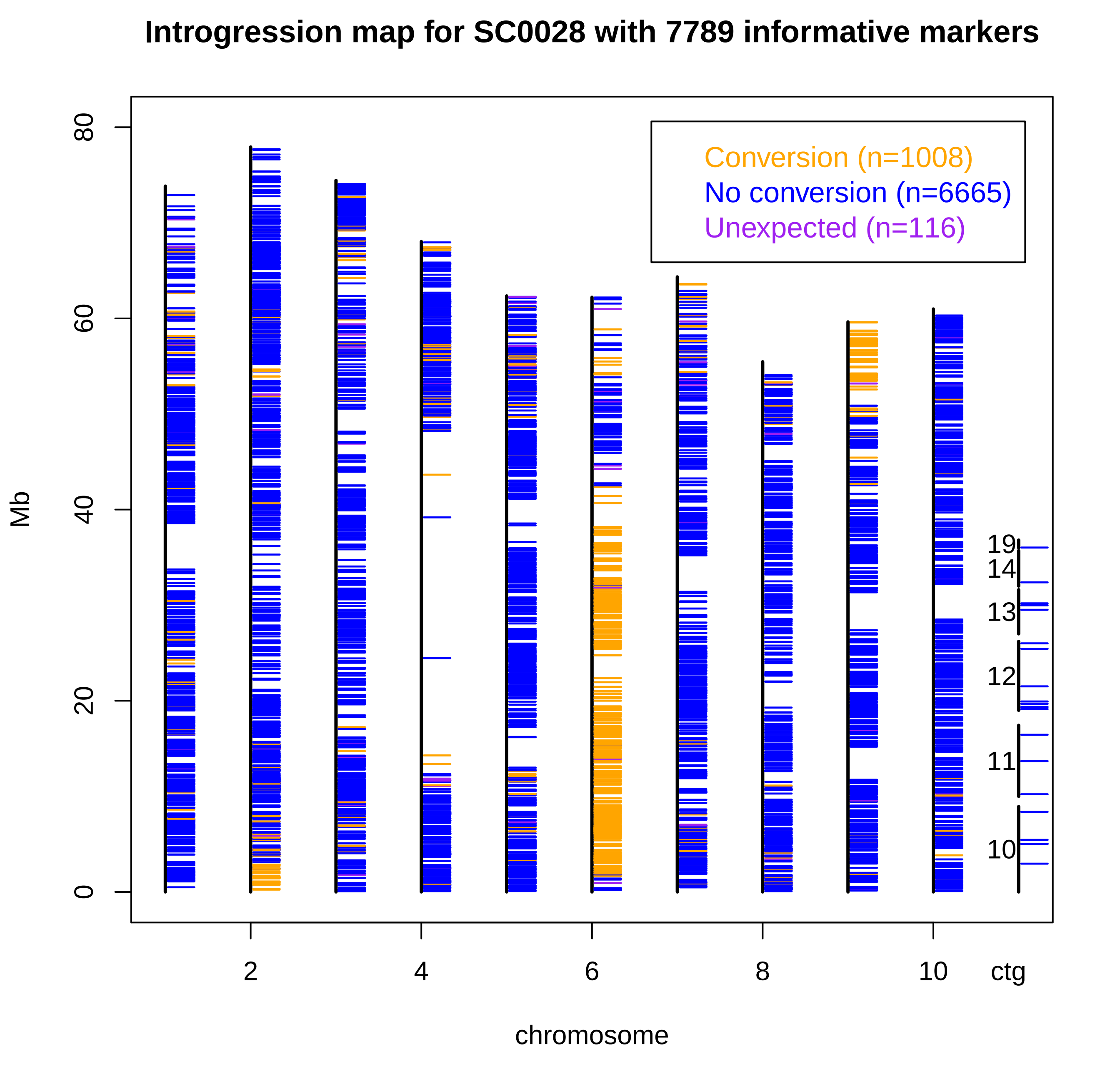
Introgression map for SC0028 with 7789 informative markers
Conversion (n=1008)
No conversion (n=6665)
Unexpected (n=116)
Mb
0
20
40
60
80
chromosome
2
4
6
8
10
ctg
19
14
13
12
11
10

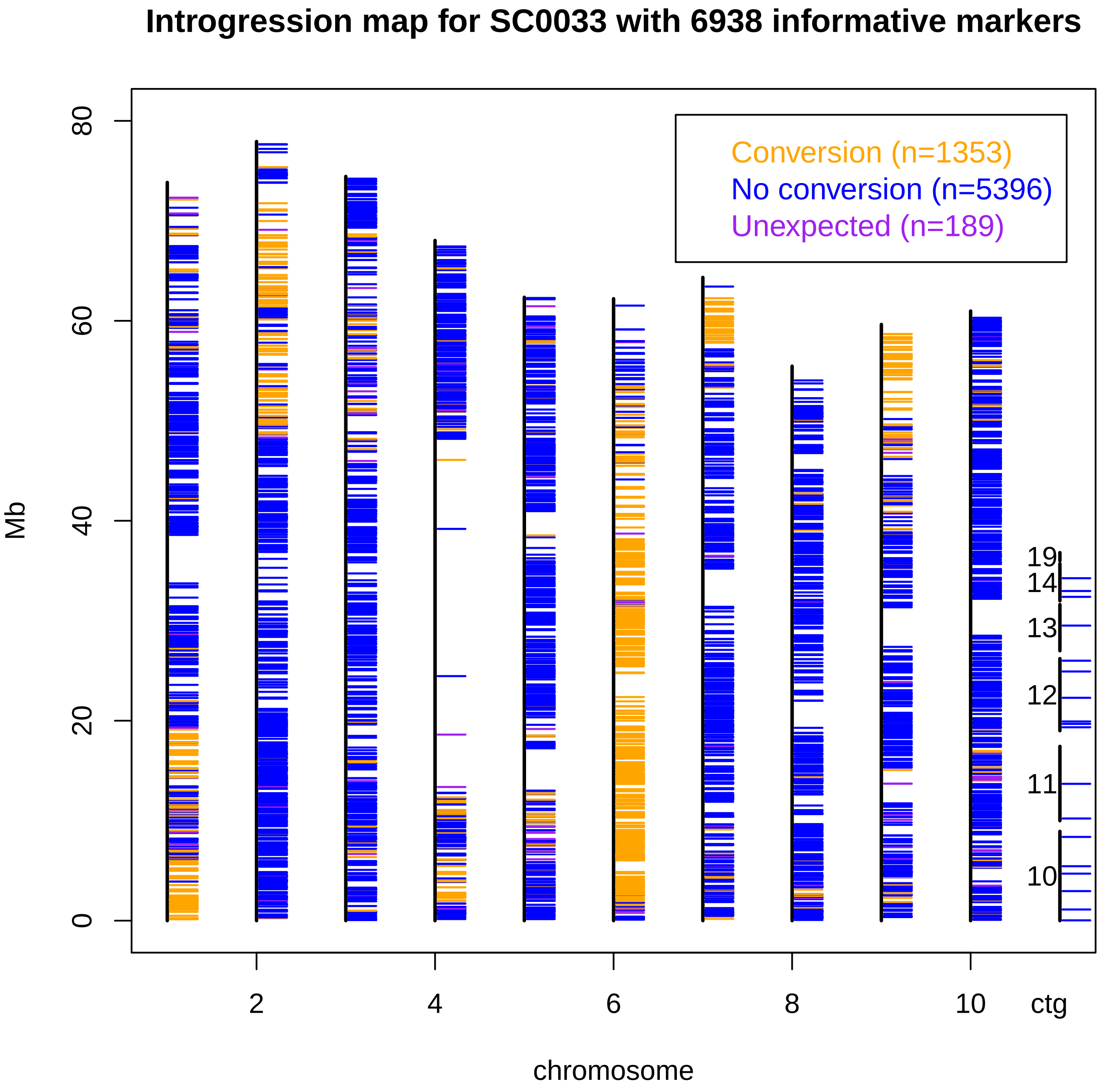
Introgression map for SC0033 with 6938 informative markers
Conversion (n=1353)
No conversion (n=5396)
Unexpected (n=189)
Mb
0
20
40
60
80
2
4
6
8
10
ctg
19
14
13
12
11
10
chromosome

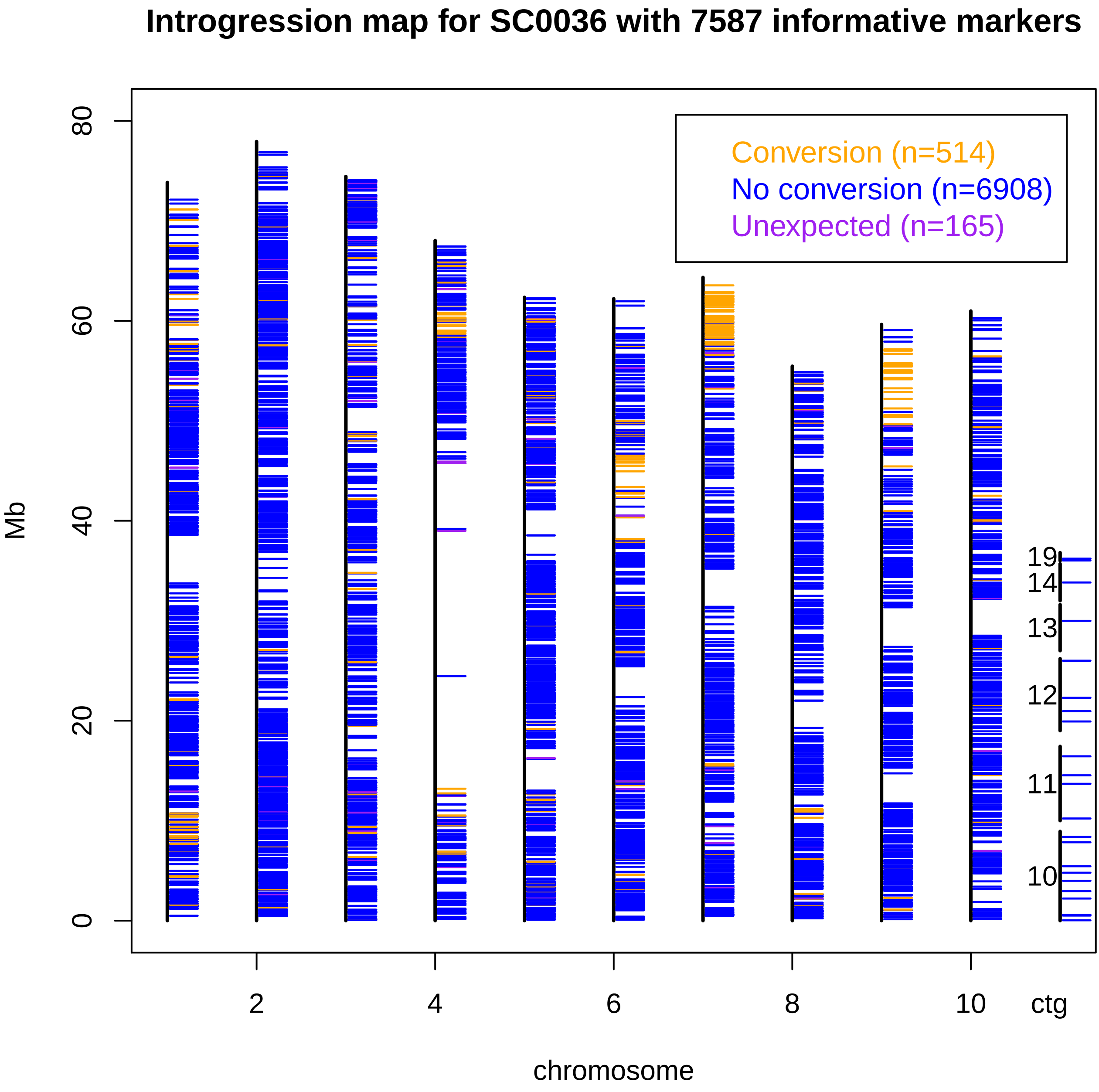

Introgression map for SC0036 with 7587 informative markers
Conversion (n=514)
No conversion (n=6908)
Unexpected (n=165)
Mb
0
20
40
60
80
chromosome
2
4
6
8
10
ctg
19
14
13
12
11
10

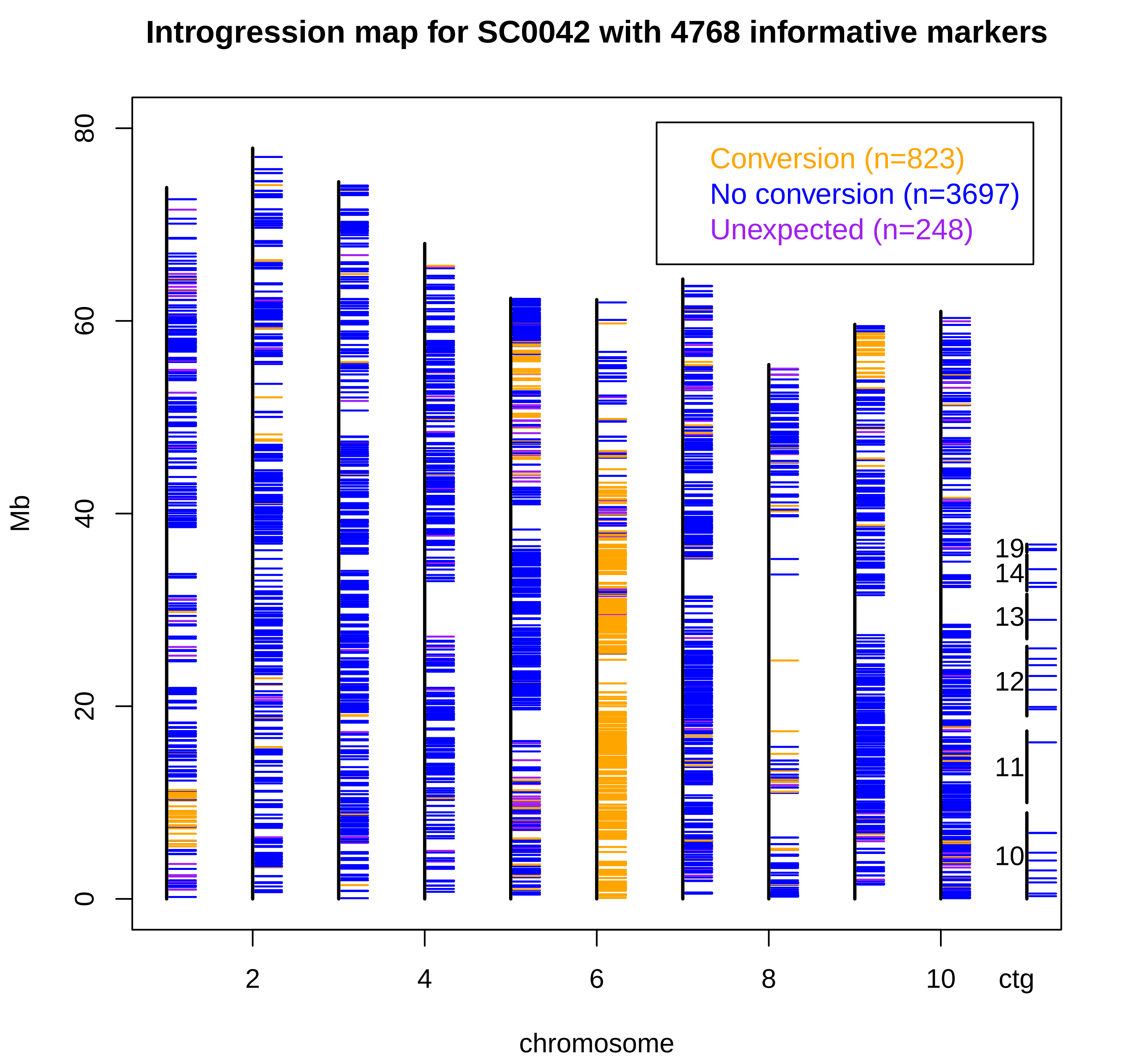

Introgression map for SC0042 with 4768 informative markers
Conversion (n=823)
No conversion (n=3697)
Unexpected (n=248)
Mb
0
20
40
60
80
chromosome
2
4
6
8
10
ctg
19
14
13
12
11
10

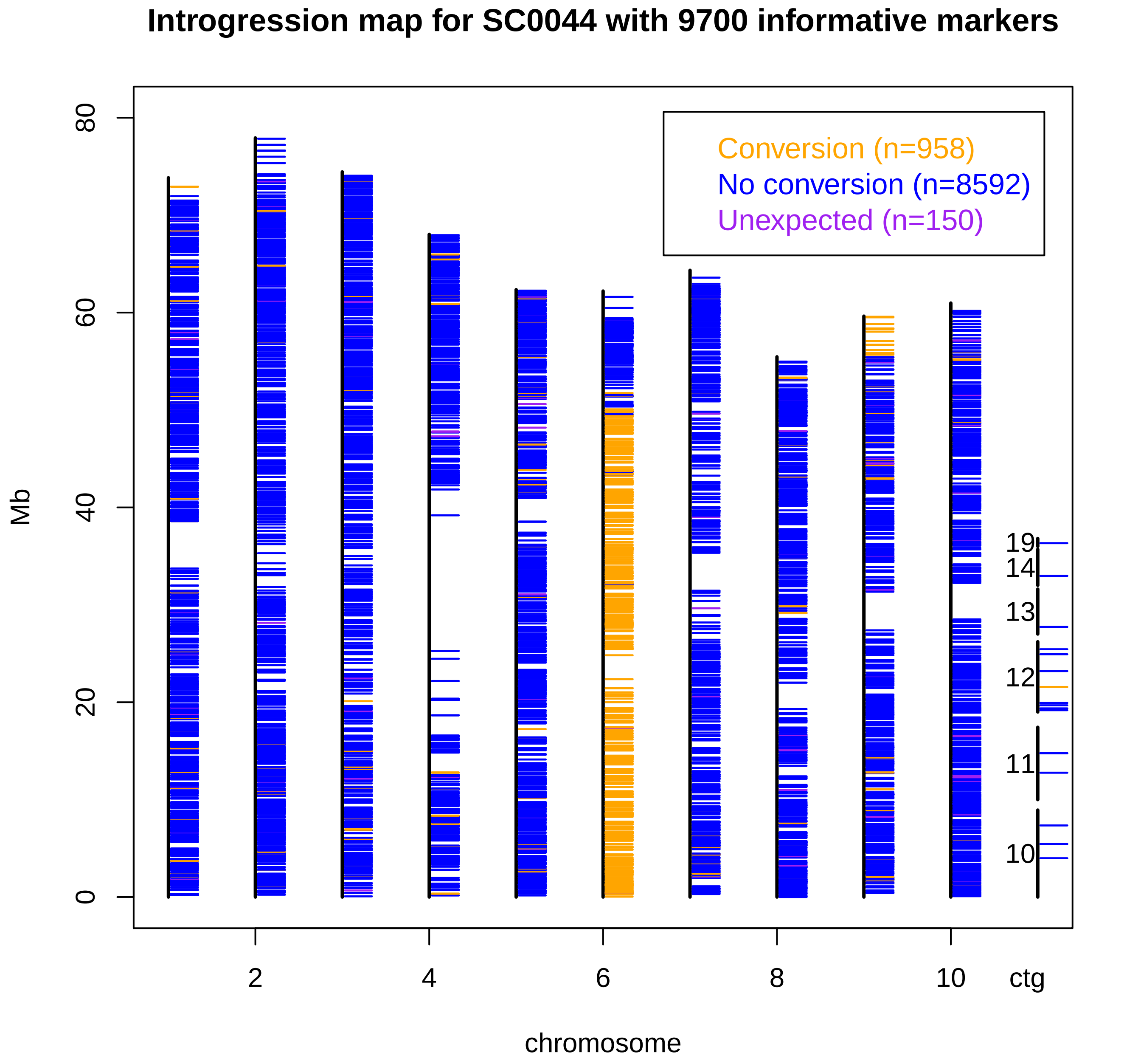

Introgression map for SC0044 with 9700 informative markers
Conversion (n=958)
No conversion (n=8592)
Unexpected (n=150)
Mb
0
20
40
60
80
chromosome
2
4
6
8
10
ctg
19
14
13
12
11
10

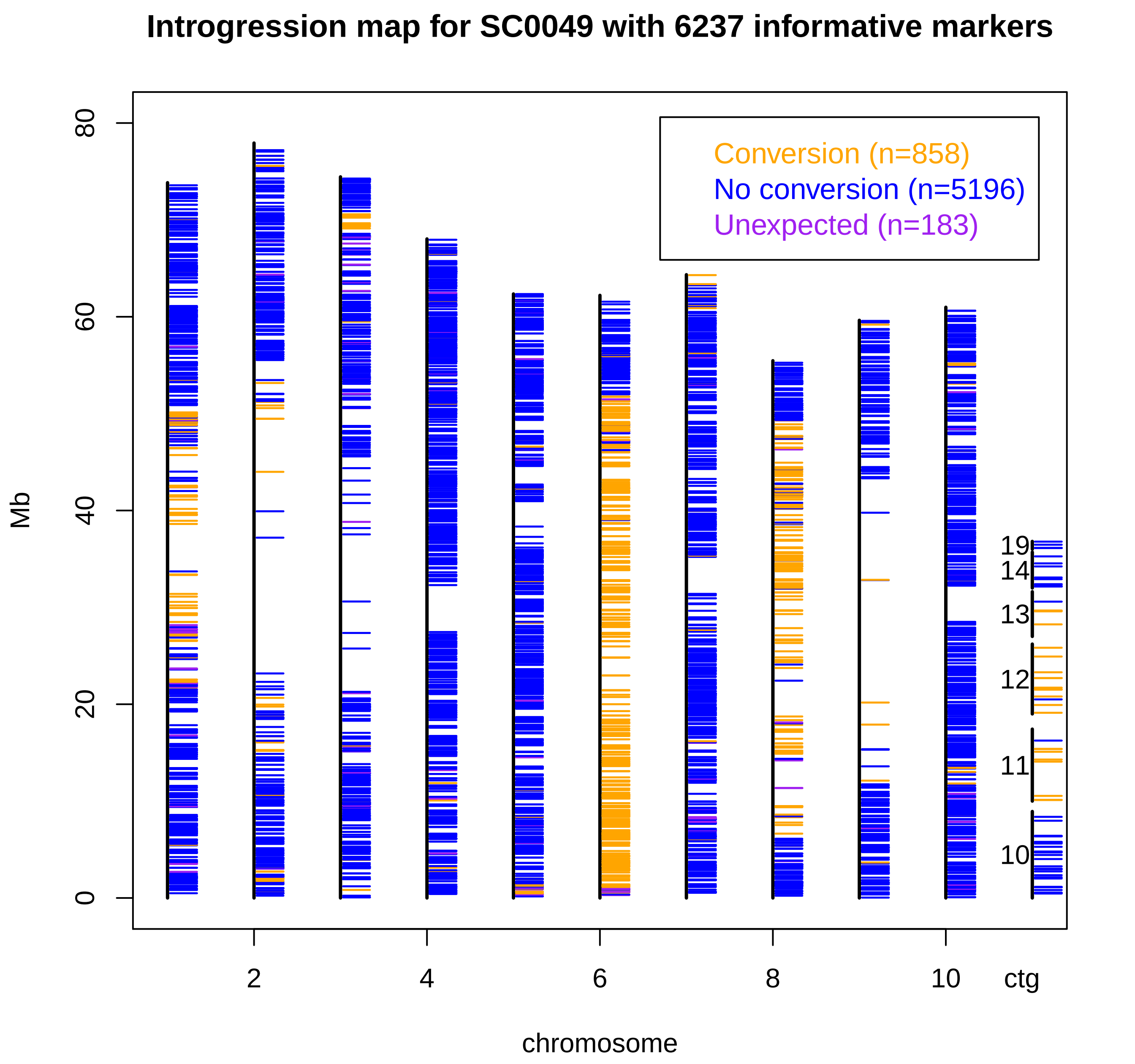

Introgression map for SC0049 with 6237 informative markers
Conversion (n=858)
No conversion (n=5196)
Unexpected (n=183)
Mb
0
20
40
60
80
2
4
6
8
10
ctg
19
14
13
12
11
10
chromosome

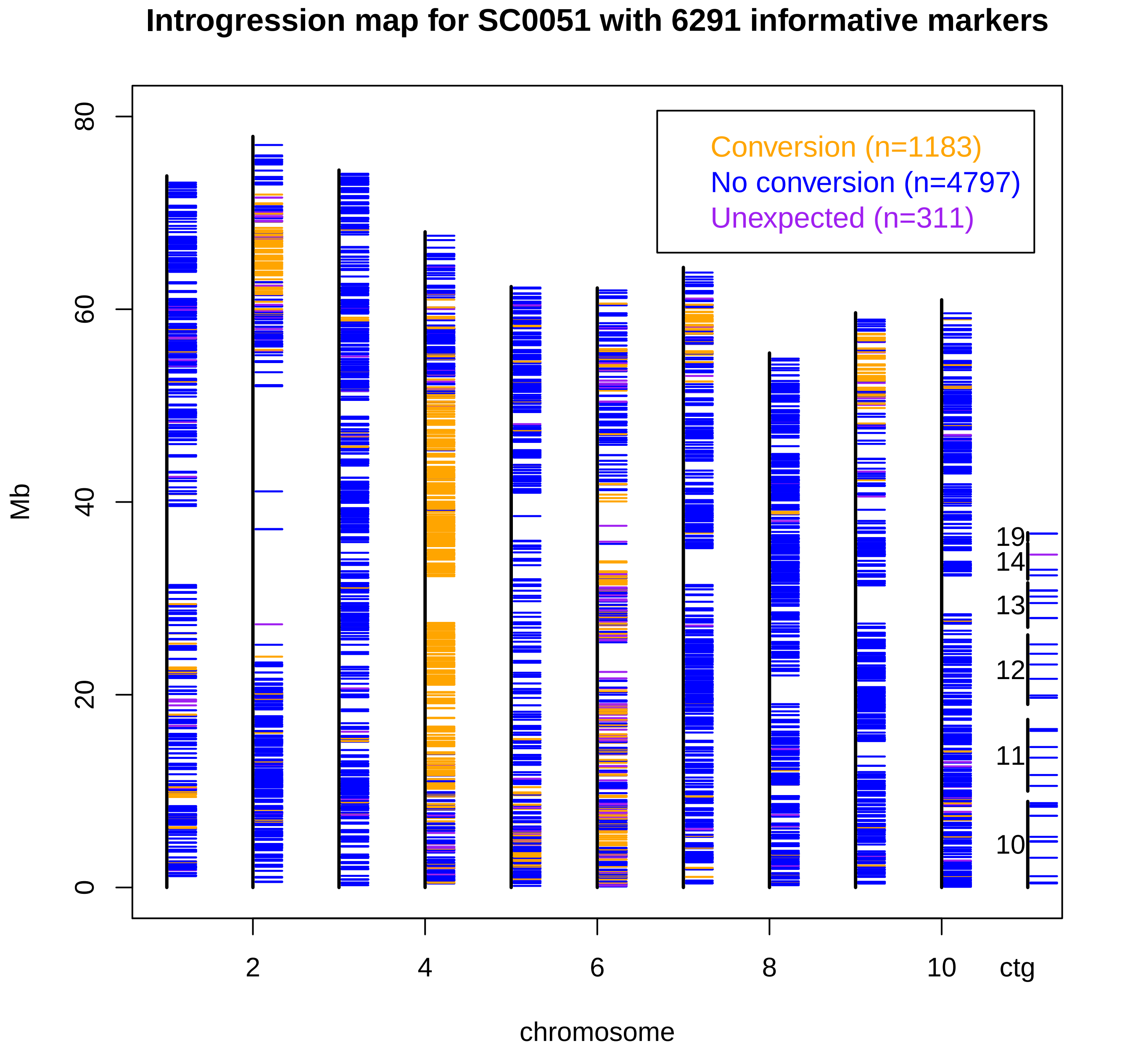
Introgression map for SC0051 with 6291 informative markers
Conversion (n=1183)
No conversion (n=4797)
Unexpected (n=311)
Mb
0
20
40
60
80
2
4
6
8
10
ctg
19
14
13
12
11
10
chromosome

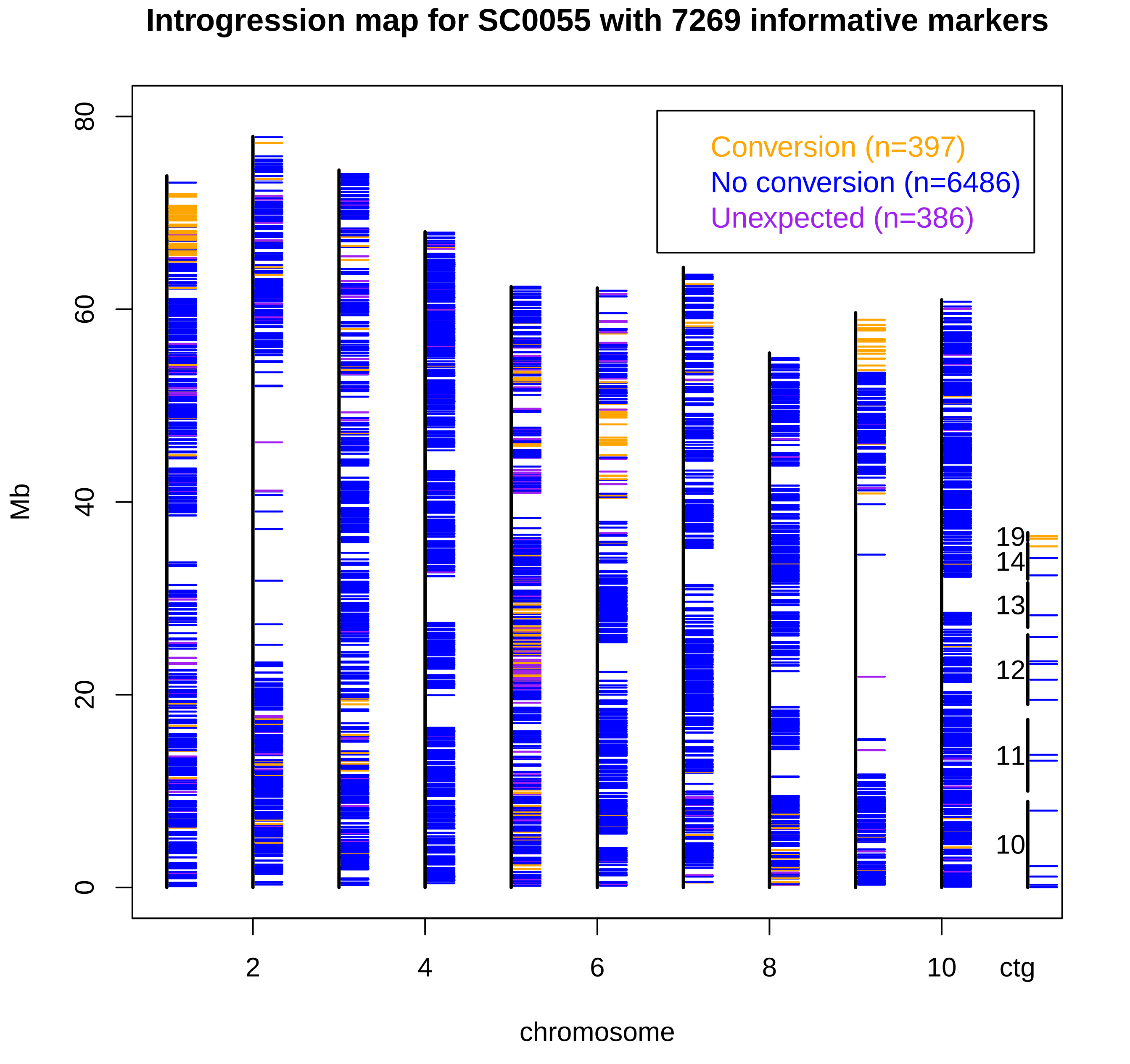
Introgression map for SC0055 with 7269 informative markers
Conversion (n=397)
No conversion (n=6486)
Unexpected (n=386)
Mb
0
20
40
60
80
chromosome
2
4
6
8
10
ctg
19
14
13
12
11
10

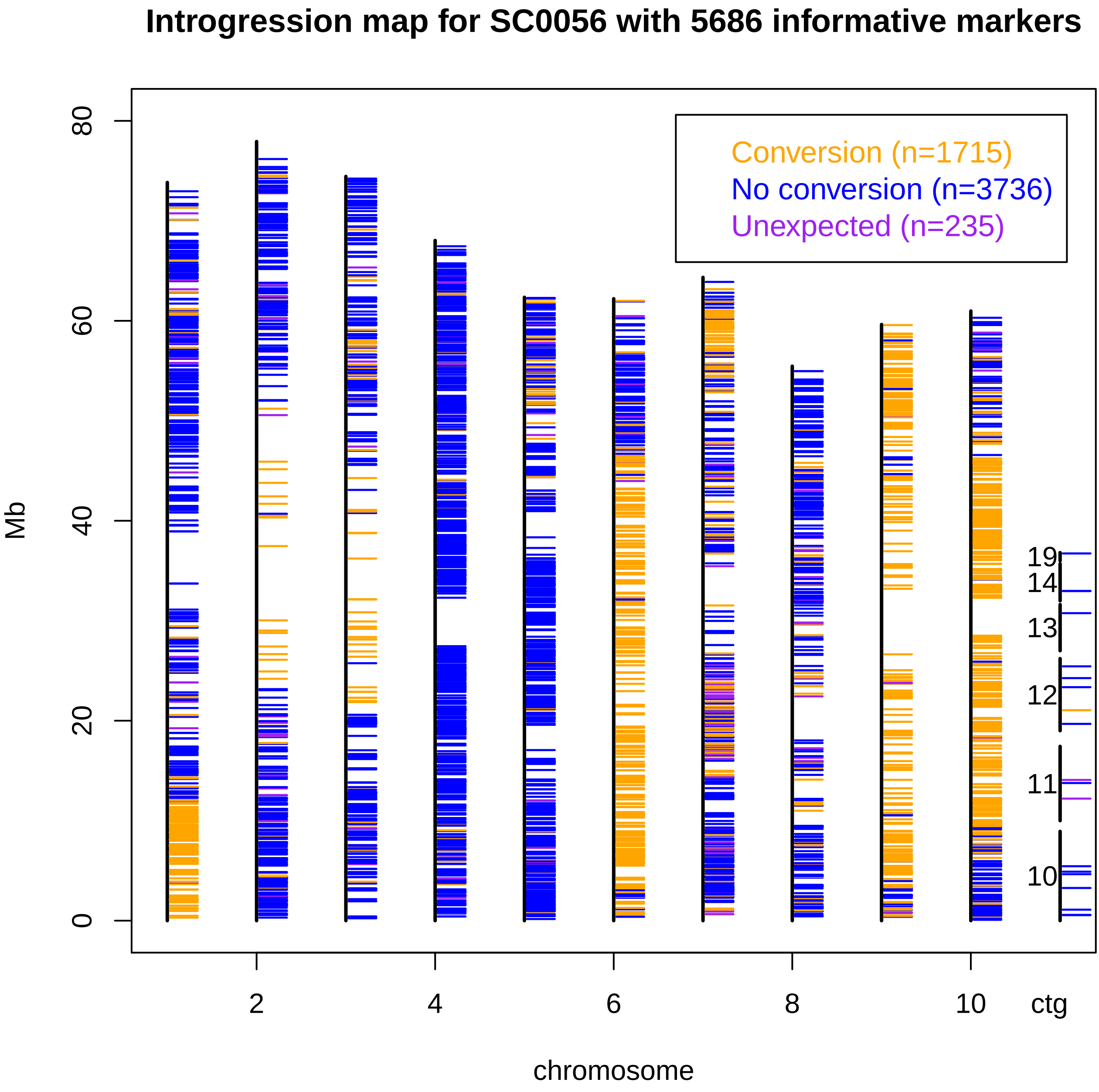
Introgression map for SC0056 with 5686 informative markers
Conversion (n=1715)
No conversion (n=3736)
Unexpected (n=235)
Mb
0
20
40
60
80
chromosome
2
4
6
8
10
ctg
19
14
13
12
11
10

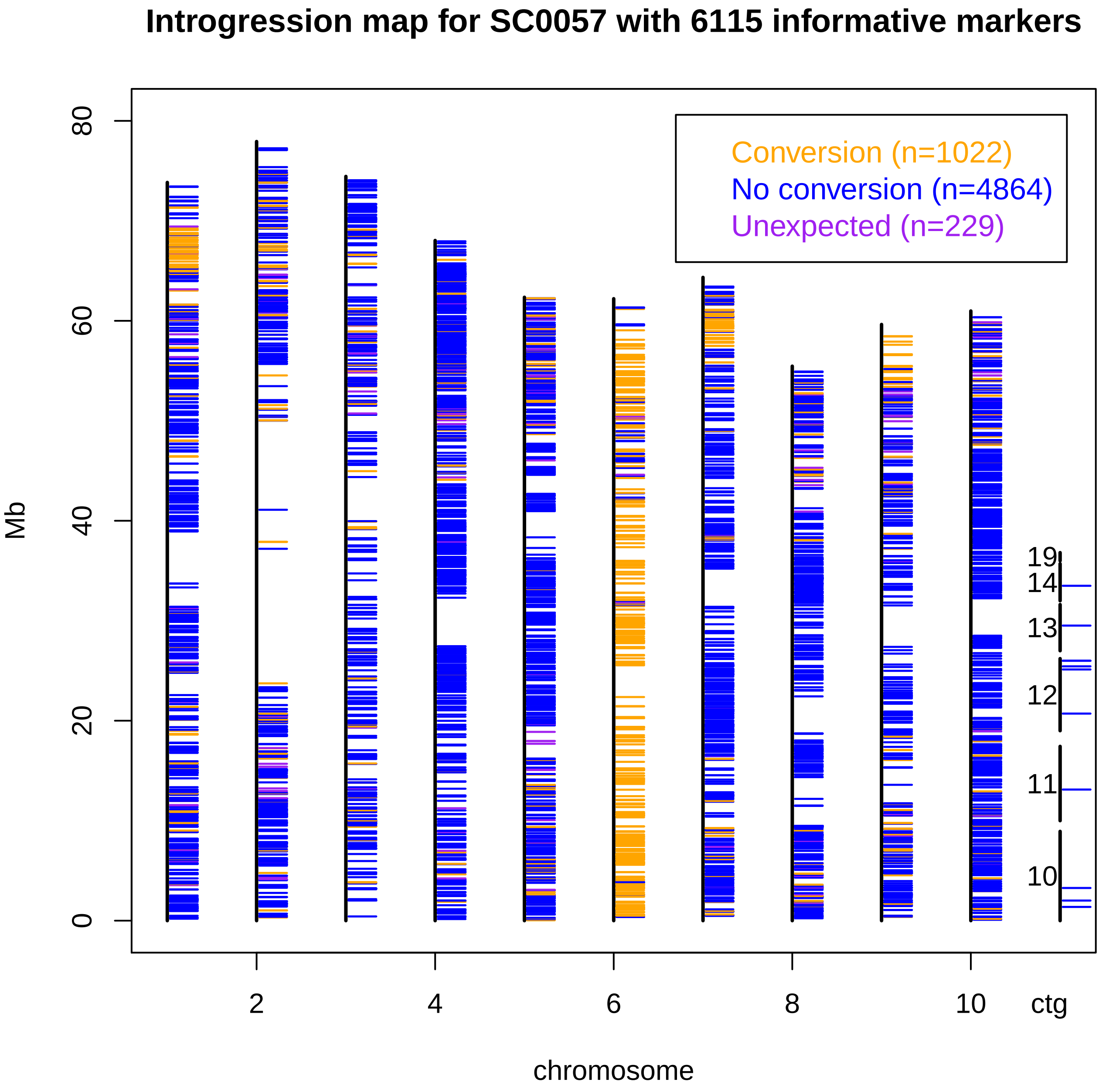

Introgression map for SC0057 with 6115 informative markers
Conversion (n=1022)
No conversion (n=4864)
Unexpected (n=229)
Mb
0
20
40
60
80
chromosome
2
4
6
8
10
ctg
19
14
13
12
11
10

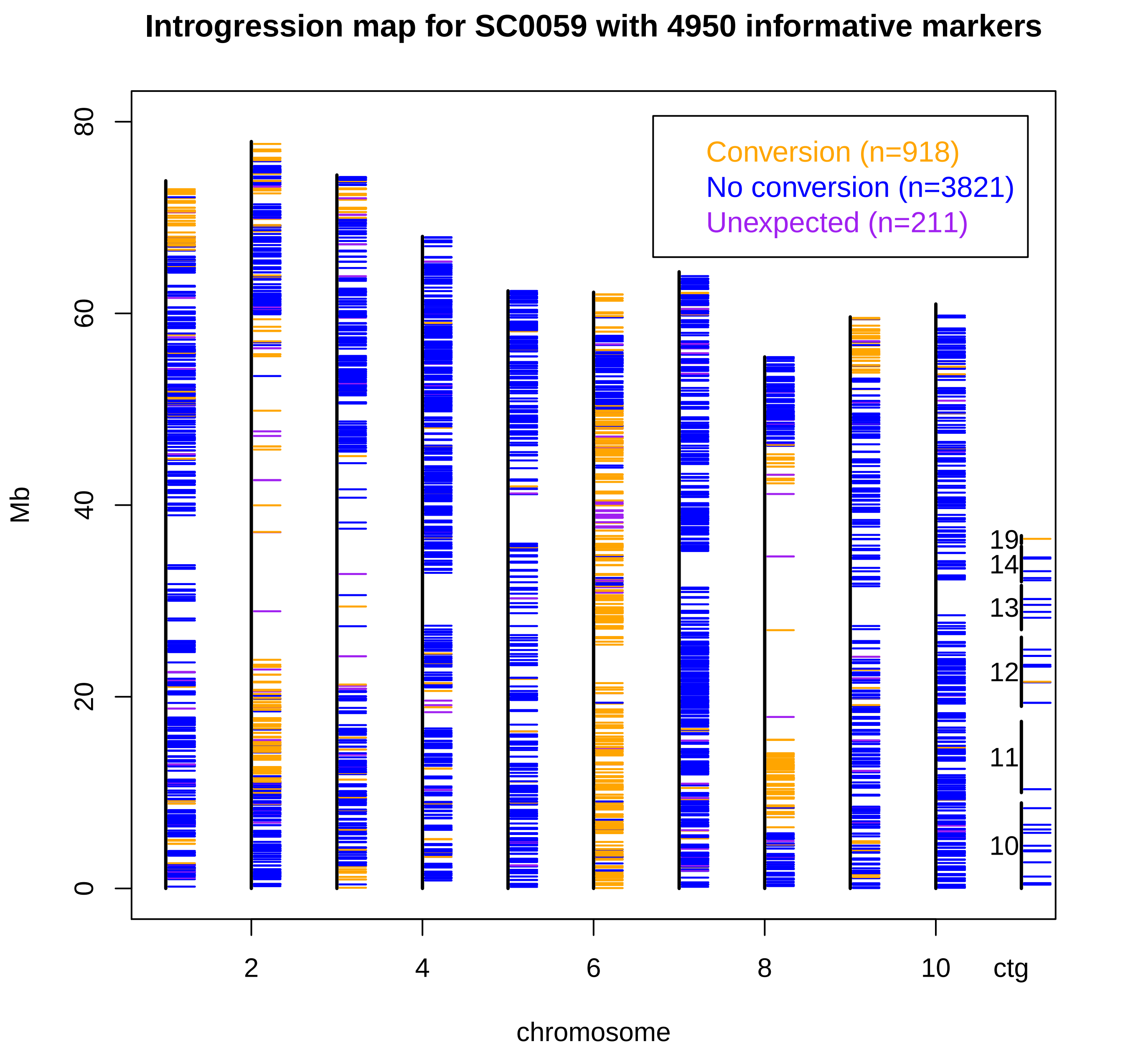
Introgression map for SC0059 with 4950 informative markers
Conversion (n=918)
No conversion (n=3821)
Unexpected (n=211)
Mb
0
20
40
60
80
chromosome
2
4
6
8
10
ctg
19
14
13
12
11
10

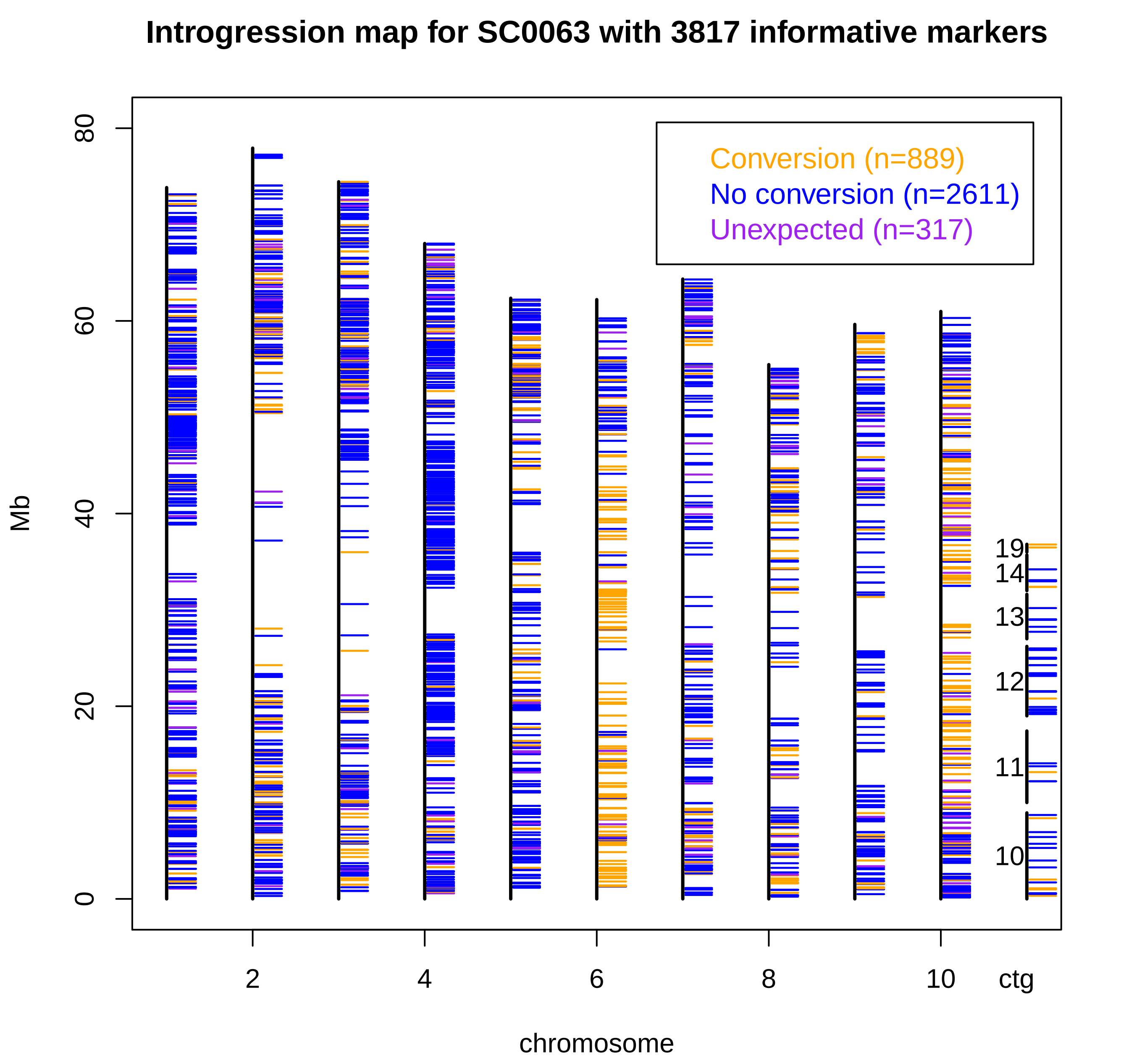

Introgression map for SC0063 with 3817 informative markers
Conversion (n=889)
No conversion (n=2611)
Unexpected (n=317)
Mb
0
20
40
60
80
2
4
6
8
10
ctg
19
14
13
12
11
10
chromosome

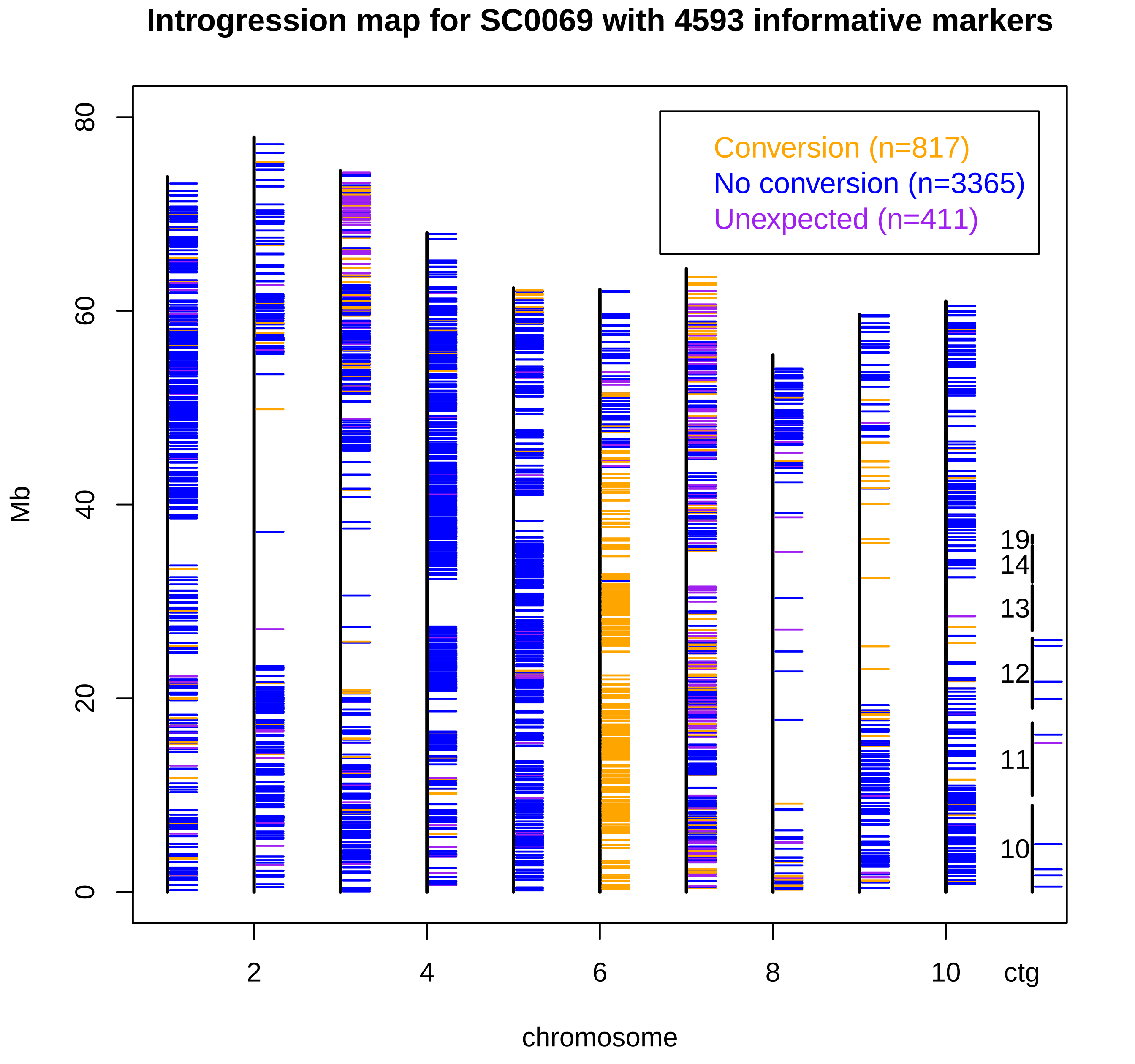

Introgression map for SC0069 with 4593 informative markers
Conversion (n=817)
No conversion (n=3365)
Unexpected (n=411)
Mb
0
20
40
60
80
chromosome
2
4
6
8
10
ctg
19
14
13
12
11
10

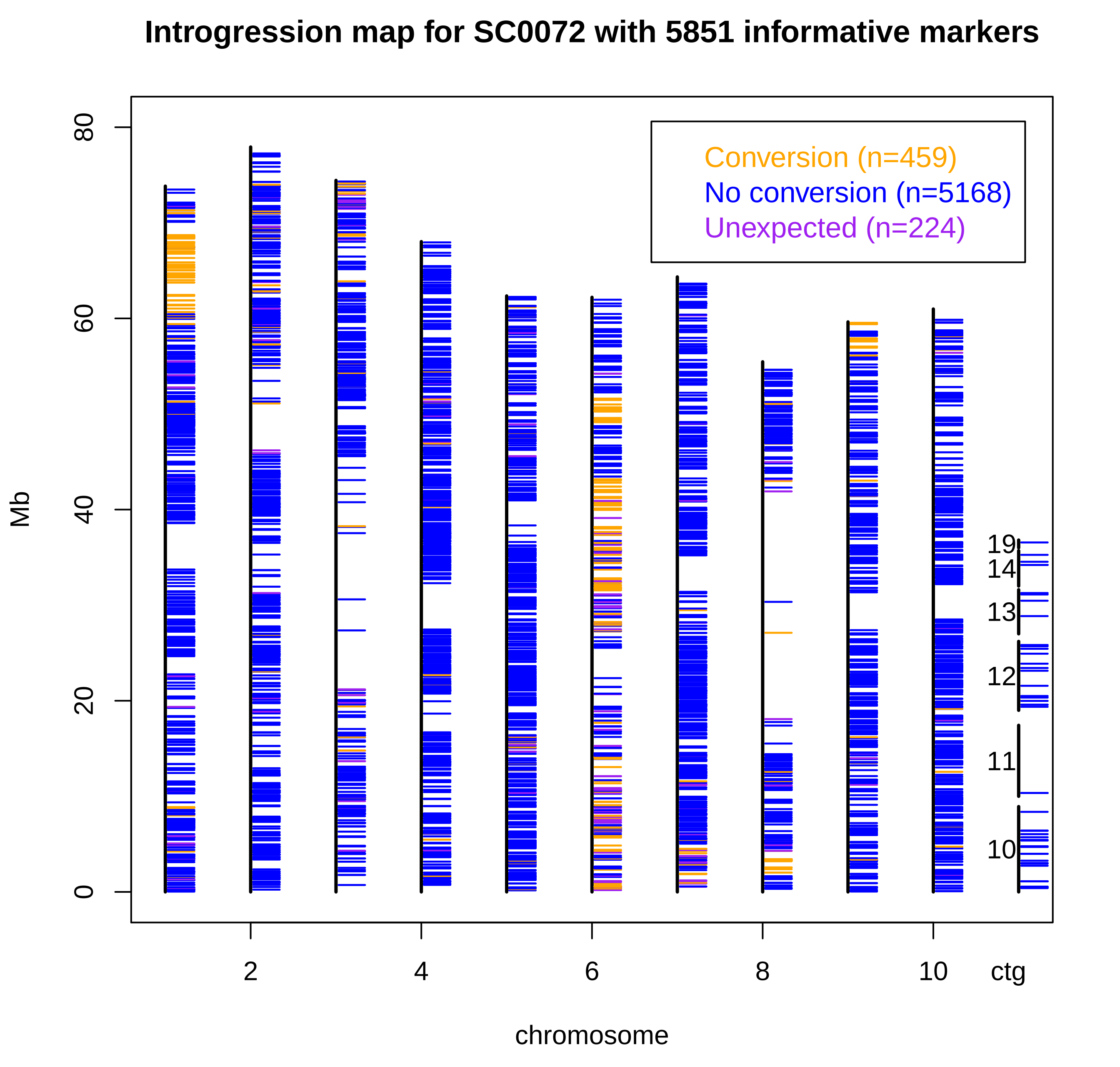
Introgression map for SC0072 with 5851 informative markers
Conversion (n=459)
No conversion (n=5168)
Unexpected (n=224)
Mb
0
20
40
60
80
chromosome
2
4
6
8
10
ctg
19
14
13
12
11
10

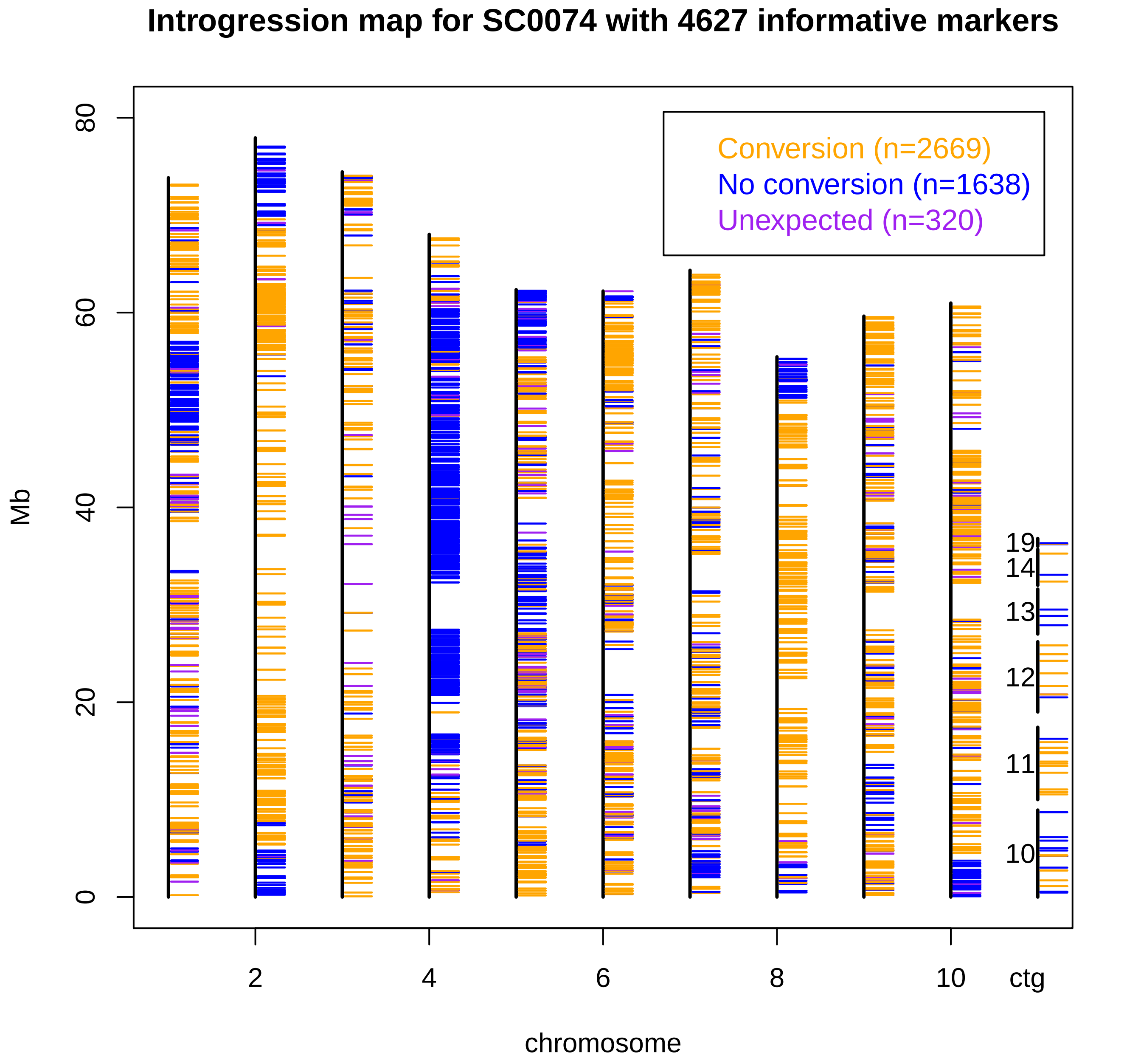

Introgression map for SC0074 with 4627 informative markers
Conversion (n=2669)
No conversion (n=1638)
Unexpected (n=320)
Mb
0
20
40
60
80
chromosome
2
4
6
8
10
ctg
19
14
13
12
11
10

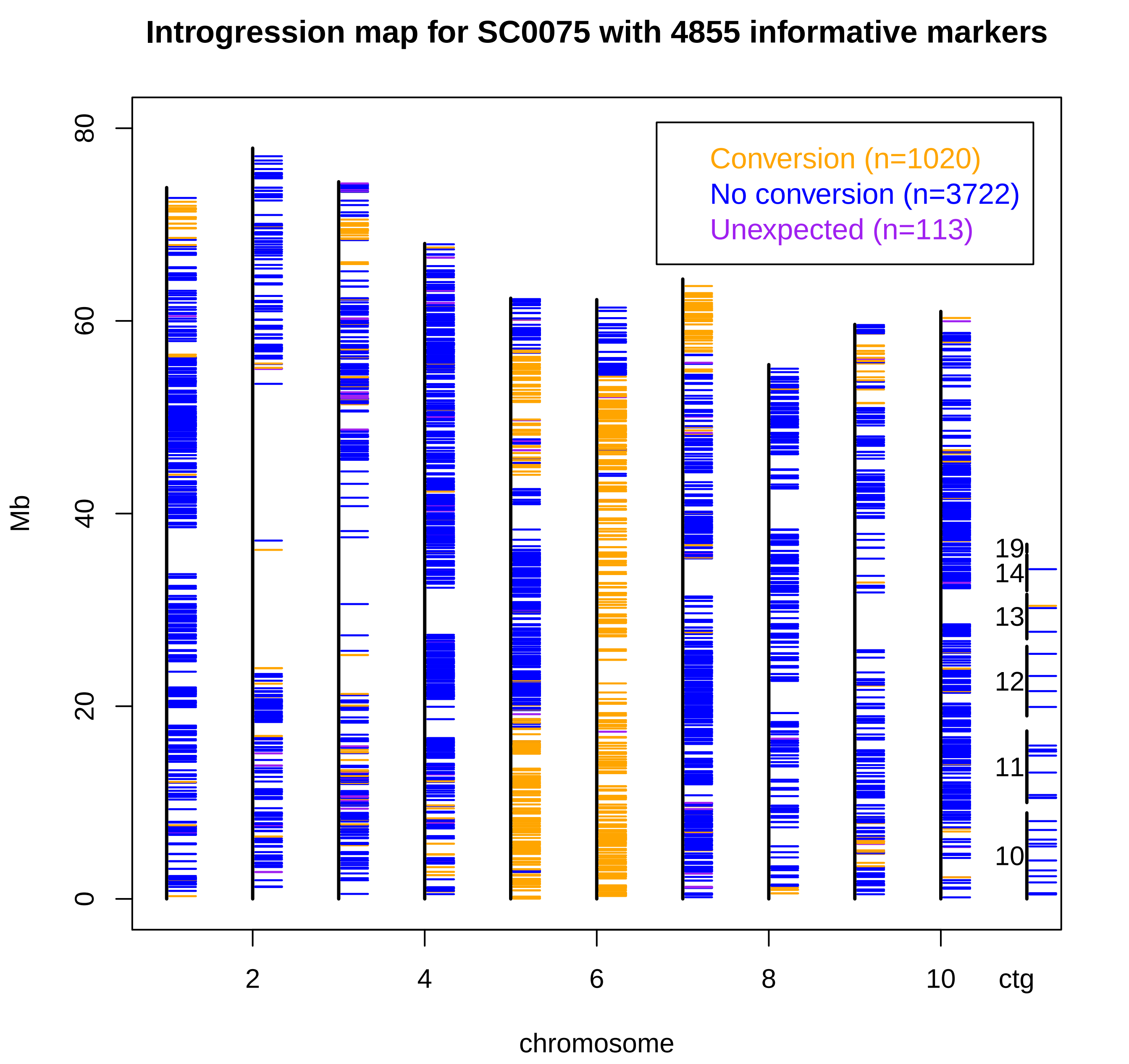

Introgression map for SC0075 with 4855 informative markers
Conversion (n=1020)
No conversion (n=3722)
Unexpected (n=113)
Mb
0
20
40
60
80
chromosome
2
4
6
8
10
ctg
19
14
13
12
11
10

Introgression map for SC0078 with 5493 informative markers

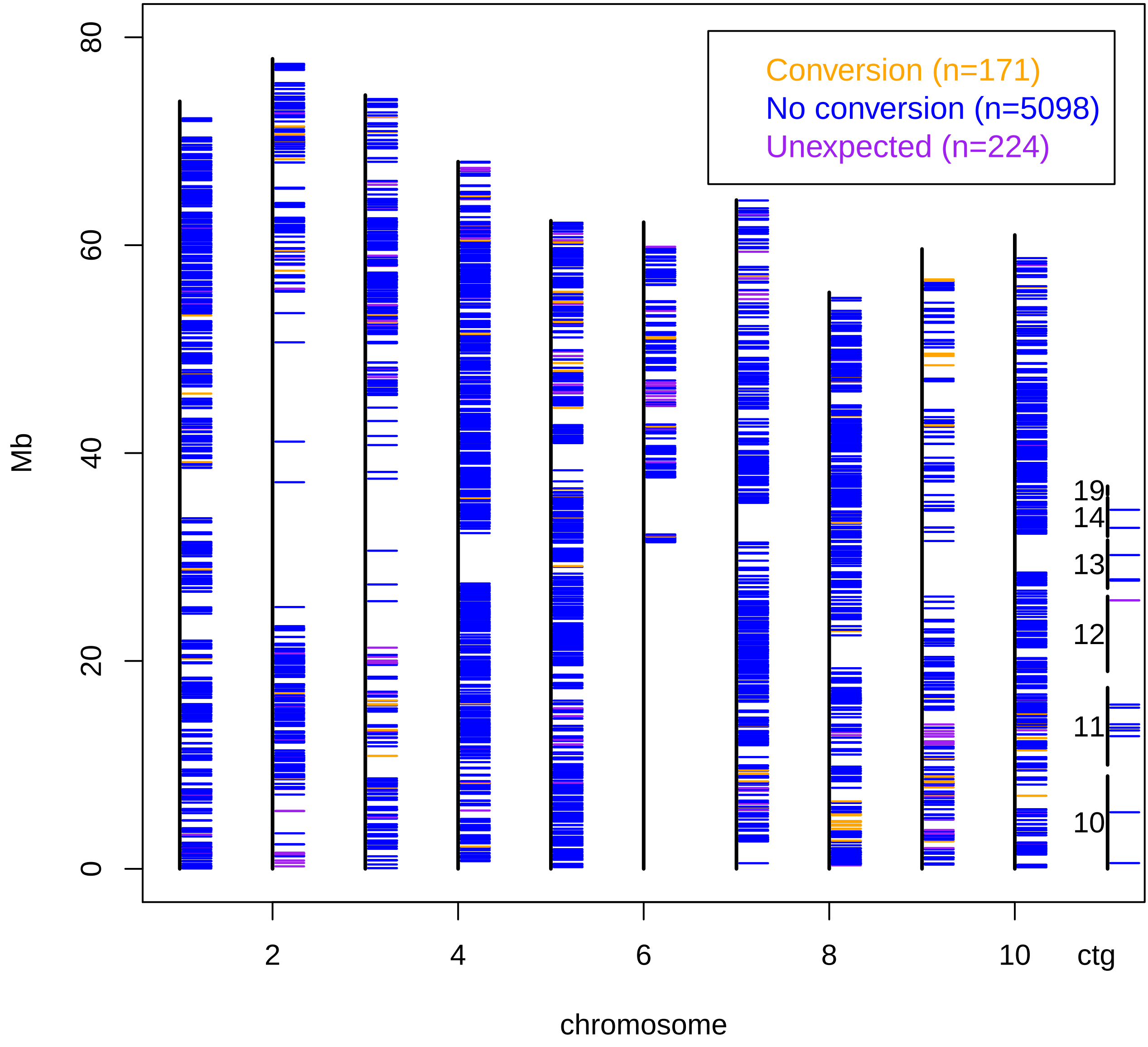

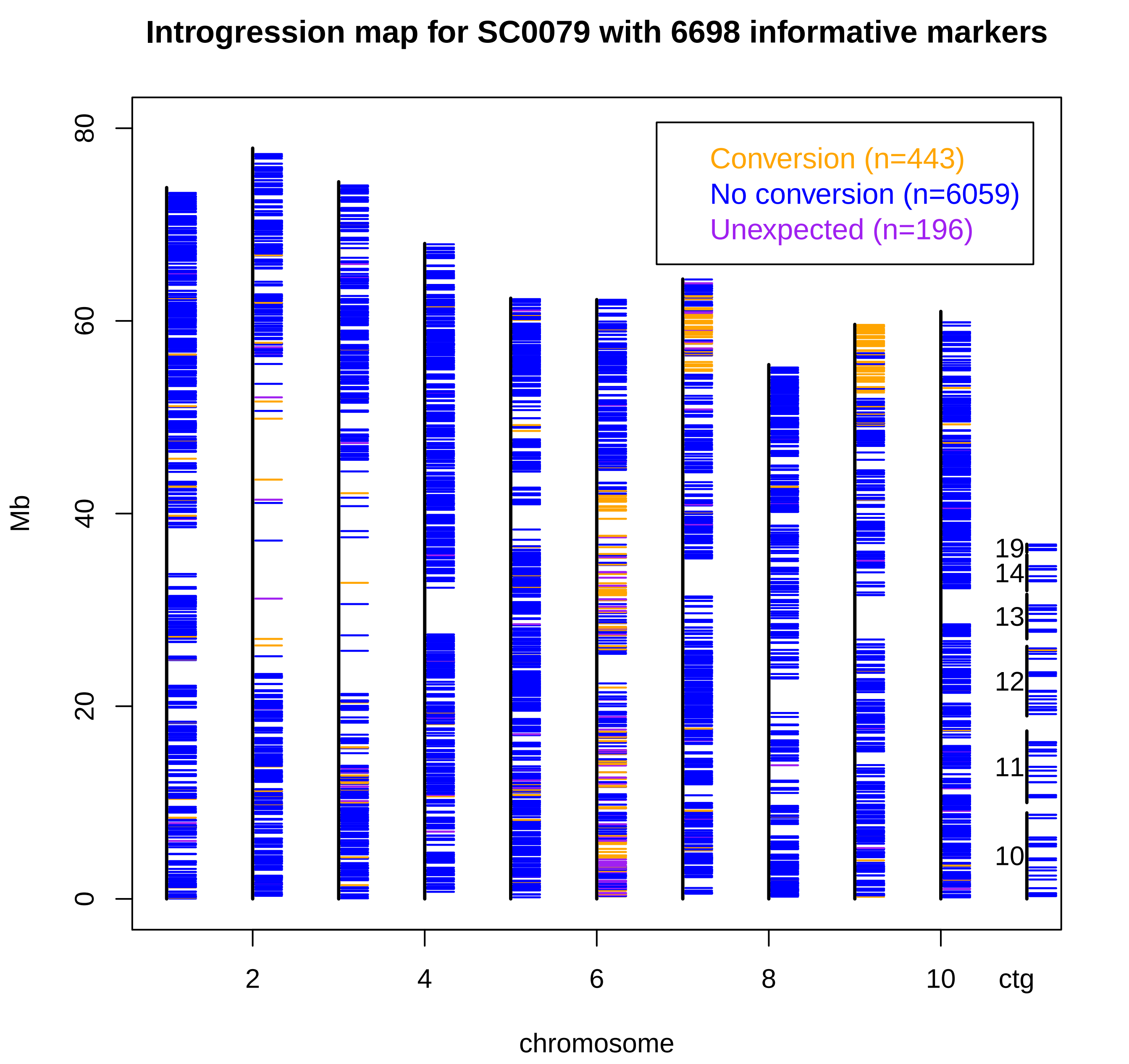
Introgression map for SC0079 with 6698 informative markers
Conversion (n=443)
No conversion (n=6059)
Unexpected (n=196)
Mb
0
20
40
60
80
2
4
6
8
10
ctg
19
14
13
12
11
10
chromosome

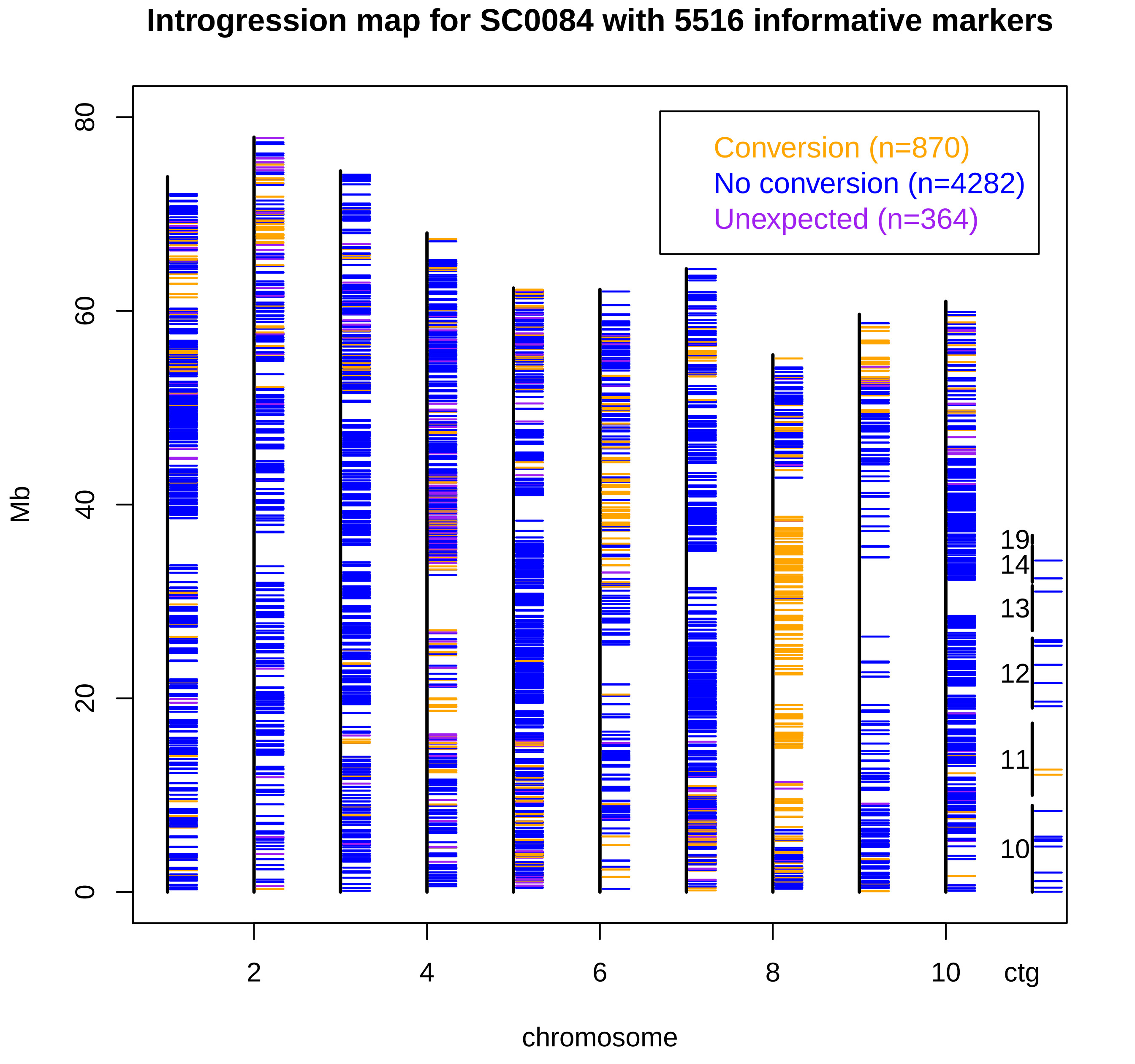
Introgression map for SC0084 with 5516 informative markers
Conversion (n=870)
No conversion (n=4282)
Unexpected (n=364)
Mb
0
20
40
60
80
chromosome
2
4
6
8
10
ctg
19
14
13
12
11
10

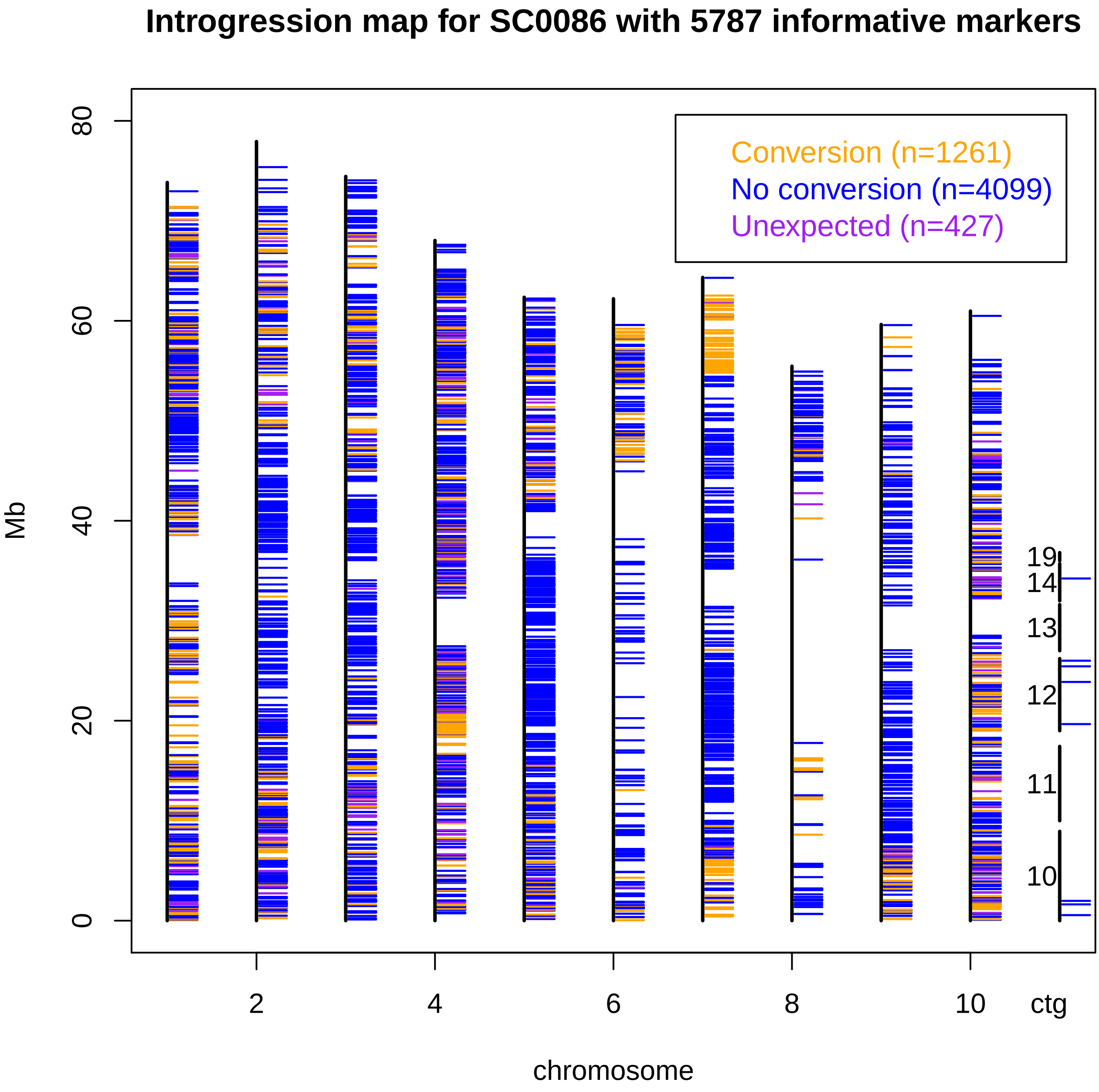

Introgression map for SC0086 with 5787 informative markers
Conversion (n=1261)
No conversion (n=4099)
Unexpected (n=427)
Mb
0
20
40
60
80
chromosome
2
4
6
8
10
ctg
19
14
13
12
11
10

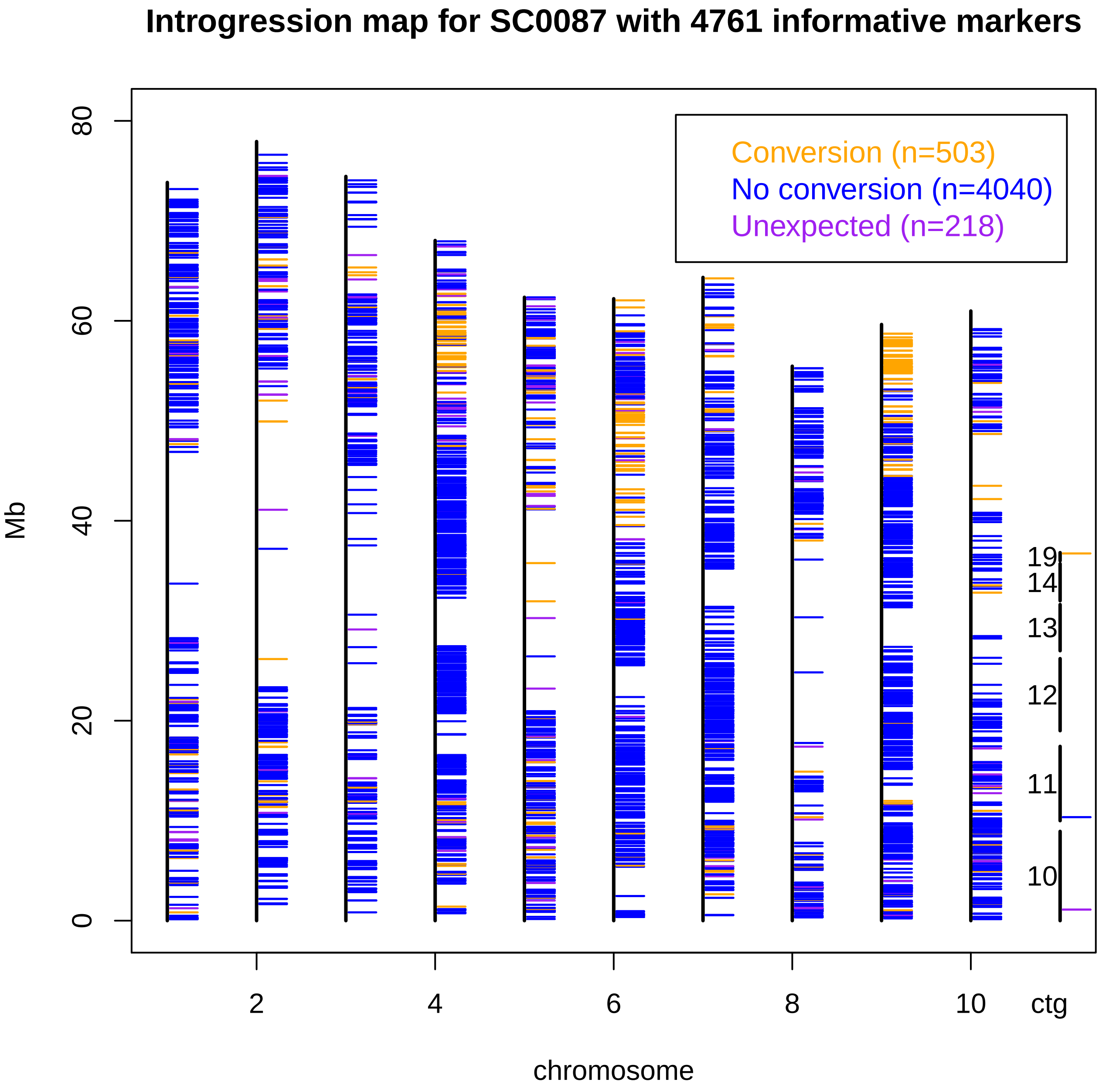

Introgression map for SC0087 with 4761 informative markers
Conversion (n=503)
No conversion (n=4040)
Unexpected (n=218)
Mb
0
20
40
60
80
chromosome
2
4
6
8
10
ctg
19
14
13
12
11
10

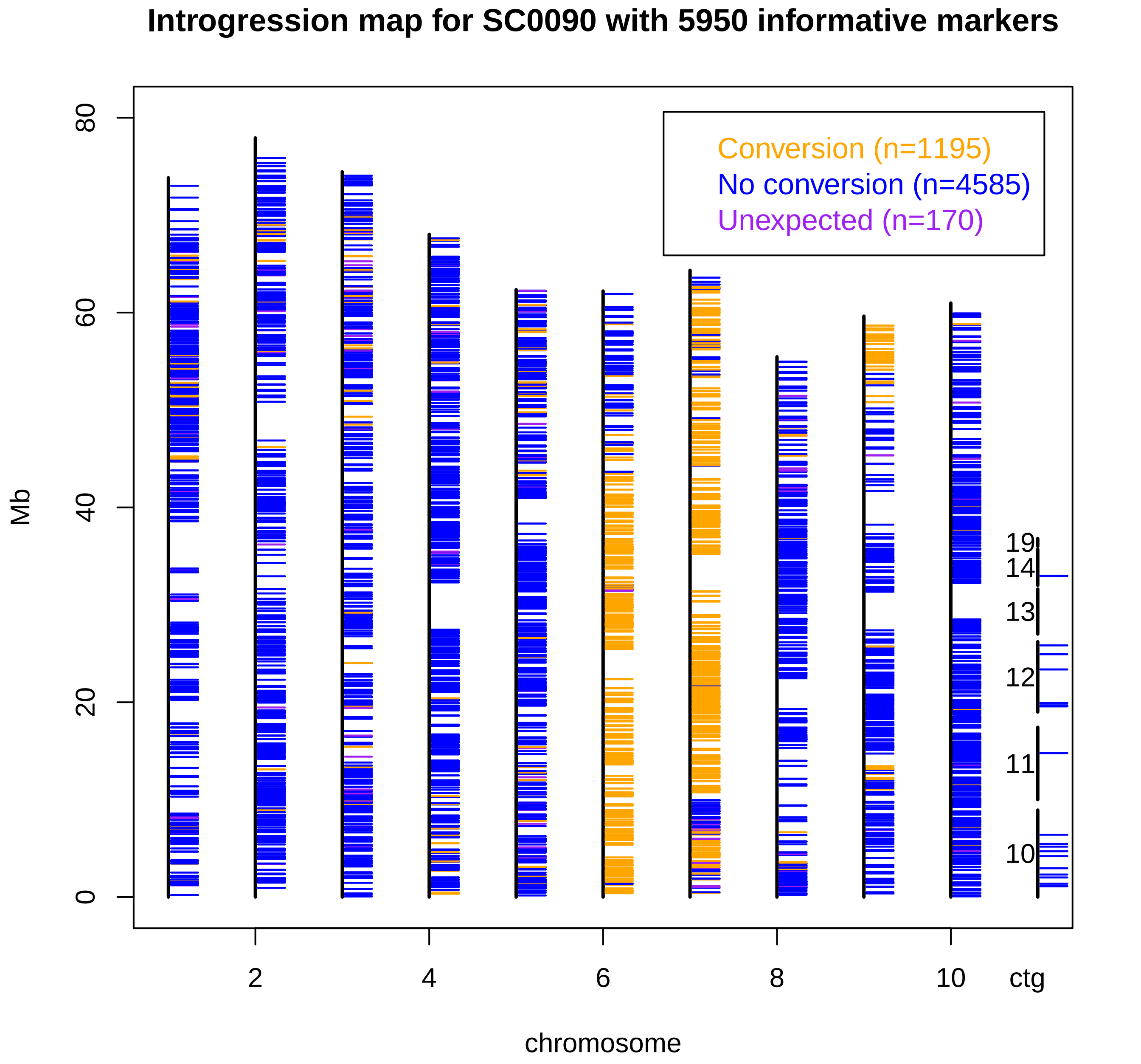

Introgression map for SC0090 with 5950 informative markers
Conversion (n=1195)
No conversion (n=4585)
Unexpected (n=170)
Mb
0
20
40
60
80
2
4
6
8
10
ctg
19
14
13
12
11
10
chromosome

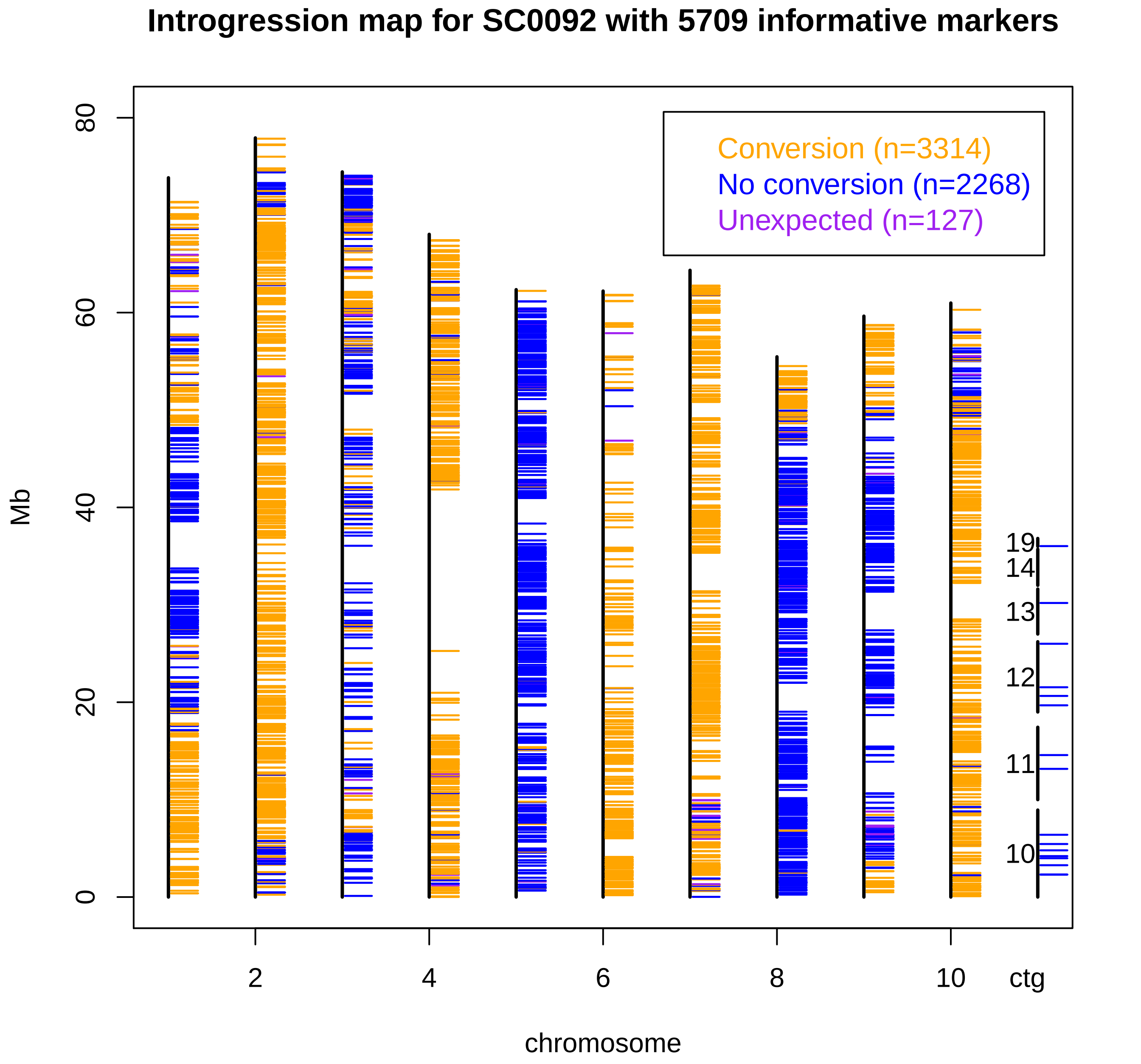
Introgression map for SC0092 with 5709 informative markers
Conversion (n=3314)
No conversion (n=2268)
Unexpected (n=127)
Mb
0
20
40
60
80
chromosome
2
4
6
8
10
ctg
19
14
13
12
11
10

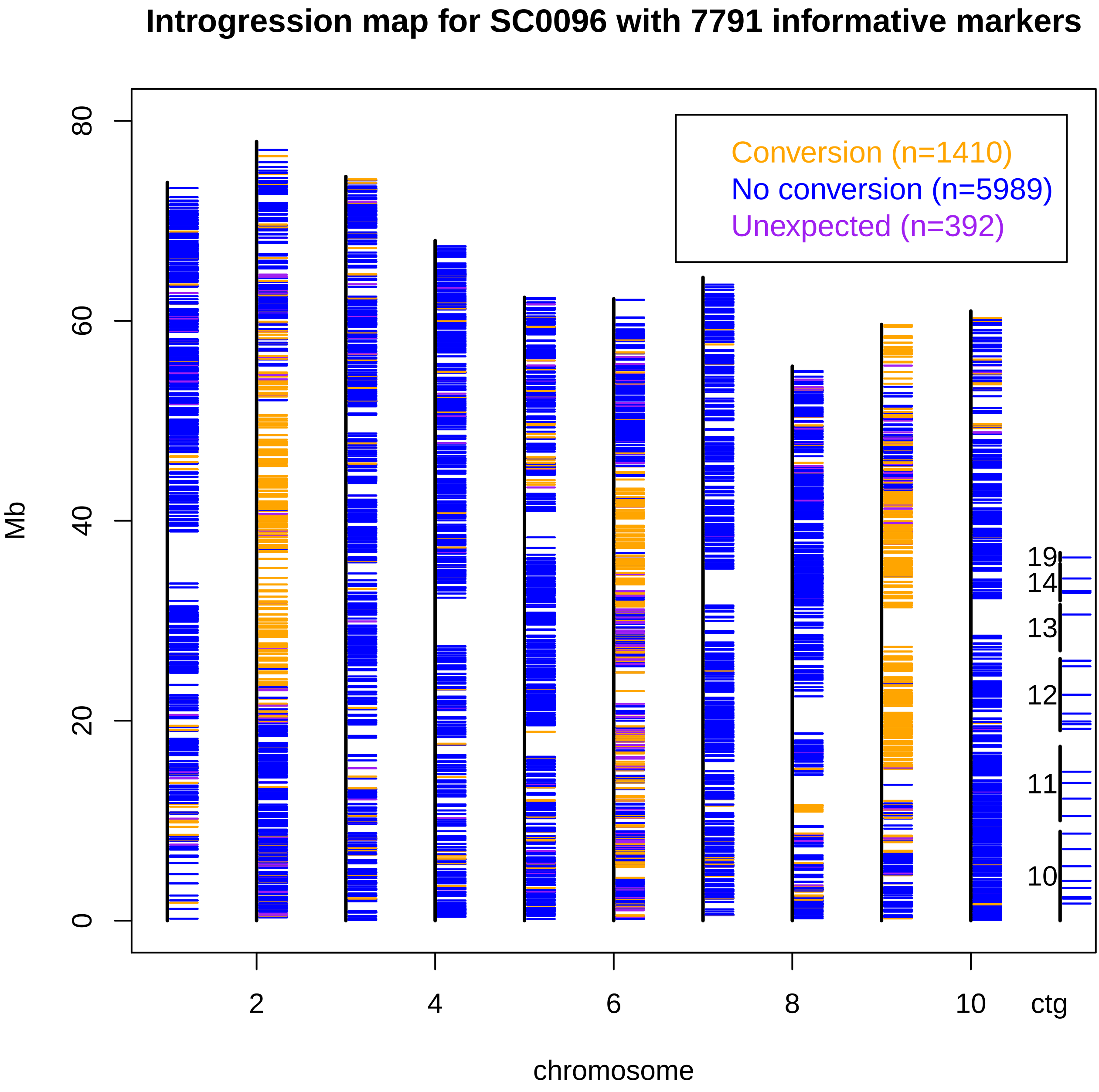
Introgression map for SC0096 with 7791 informative markers
Conversion (n=1410)
No conversion (n=5989)
Unexpected (n=392)
Mb
0
20
40
60
80
2
4
6
8
10
ctg
19
14
13
12
11
10
chromosome

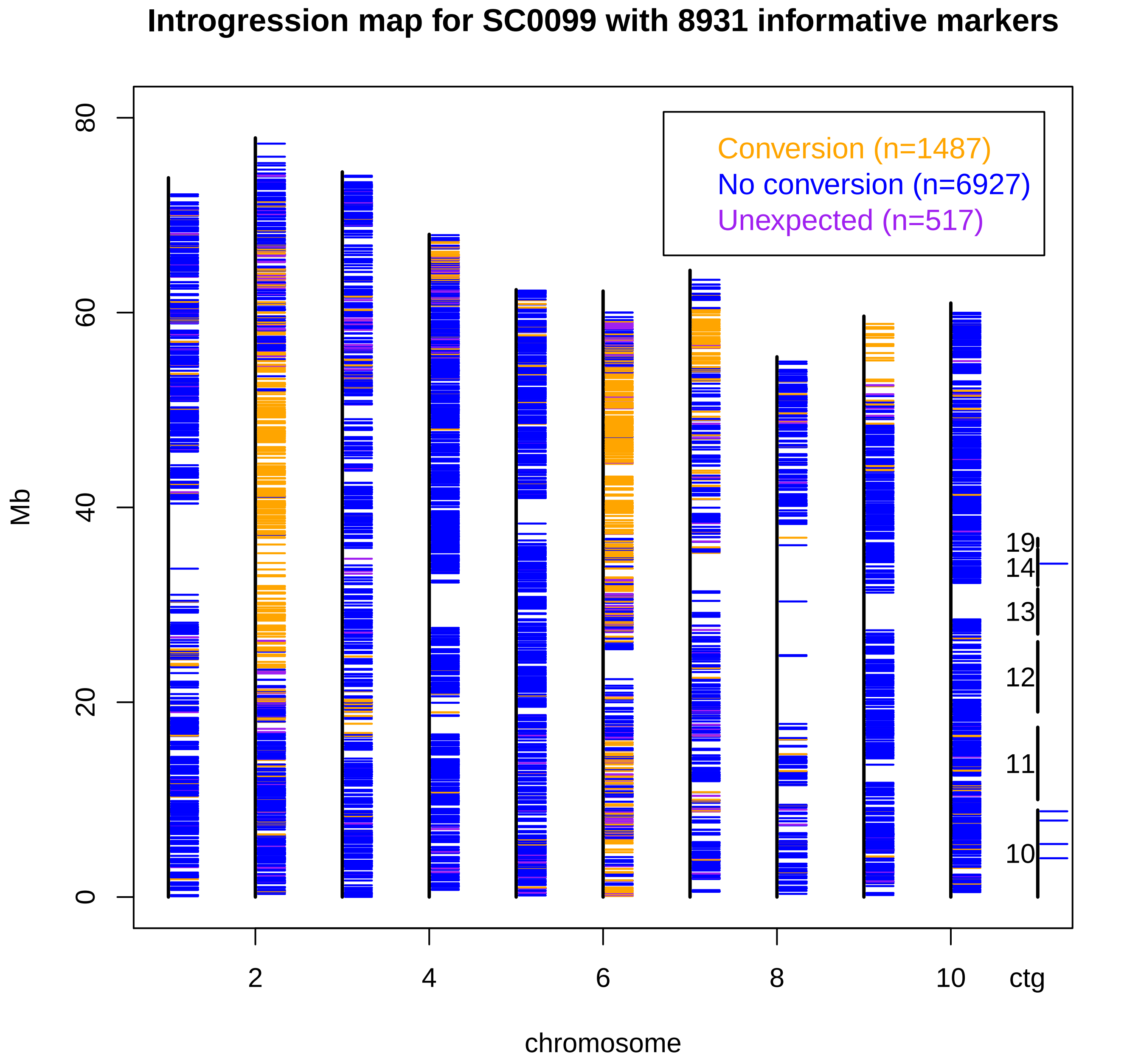
Introgression map for SC0099 with 8931 informative markers
Conversion (n=1487)
No conversion (n=6927)
Unexpected (n=517)
Mb
0
20
40
60
80
chromosome
2
4
6
8
10
ctg
19
14
13
12
11
10

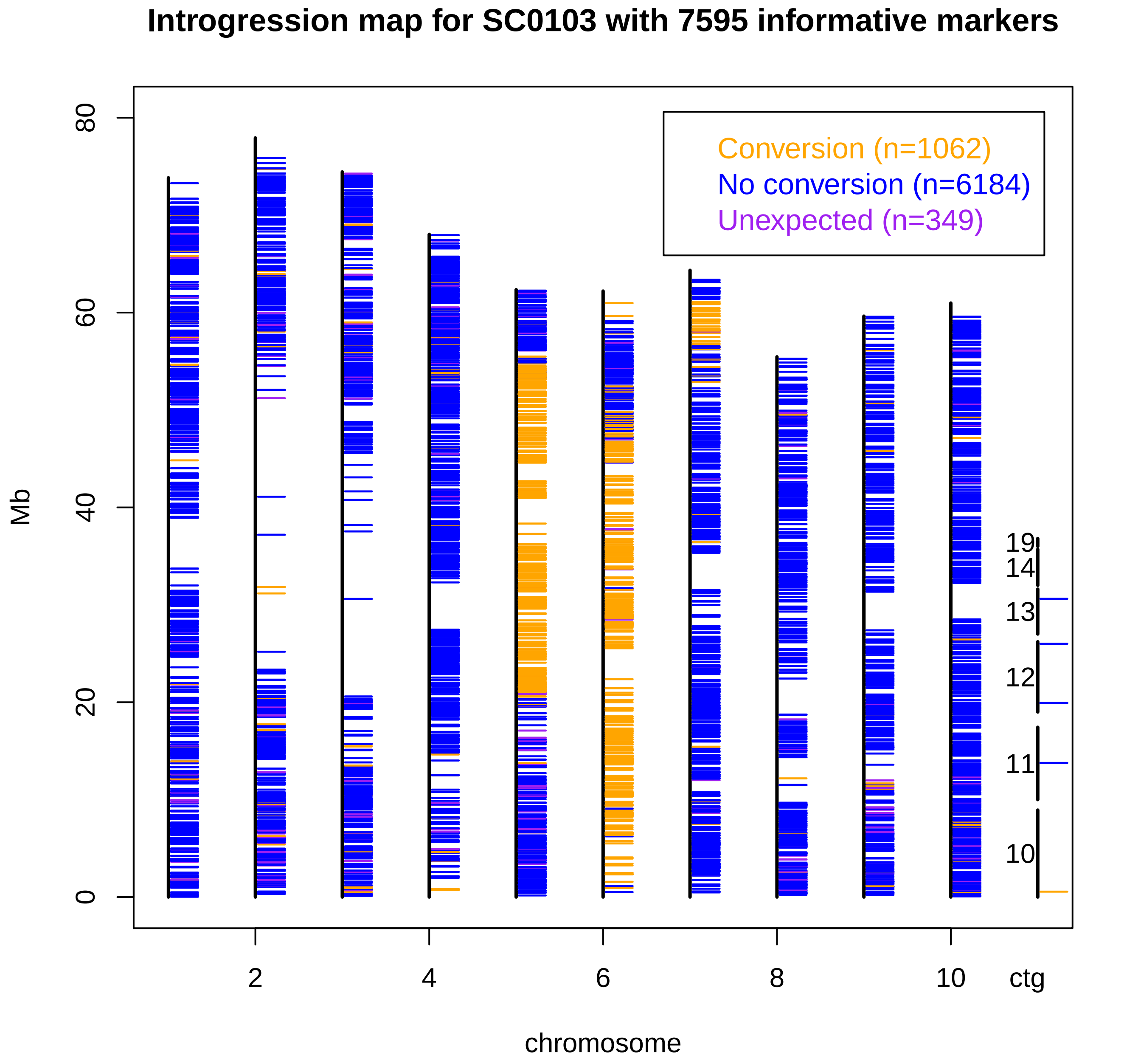

Introgression map for SC0103 with 7595 informative markers
Conversion (n=1062)
No conversion (n=6184)
Unexpected (n=349)
Mb
0
20
40
60
80
2
4
6
8
10
ctg
19
14
13
12
11
10
chromosome

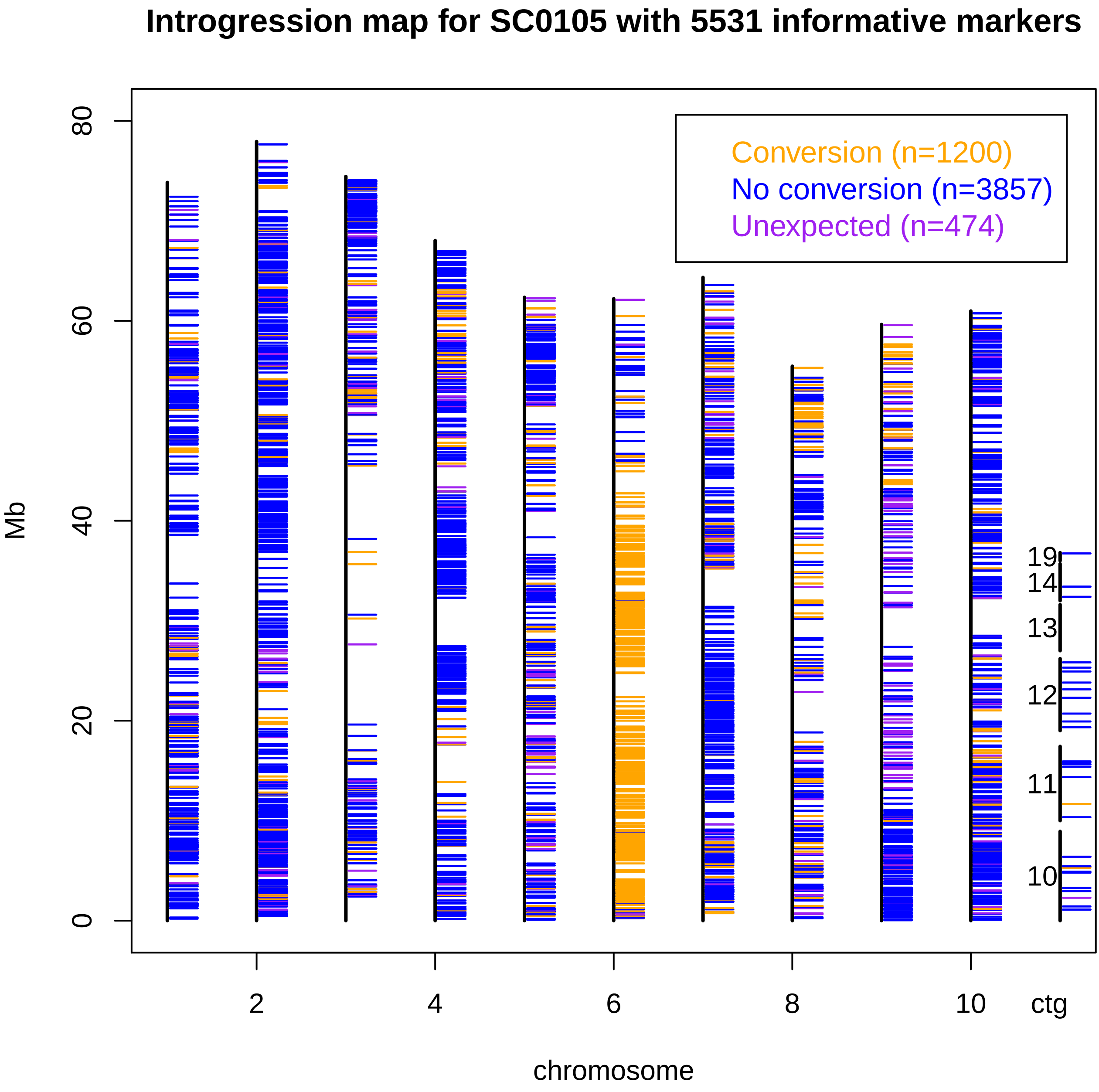
Introgression map for SC0105 with 5531 informative markers
Conversion (n=1200)
No conversion (n=3857)
Unexpected (n=474)
Mb
0
20
40
60
80
chromosome
2
4
6
8
10
ctg
19
14
13
12
11
10

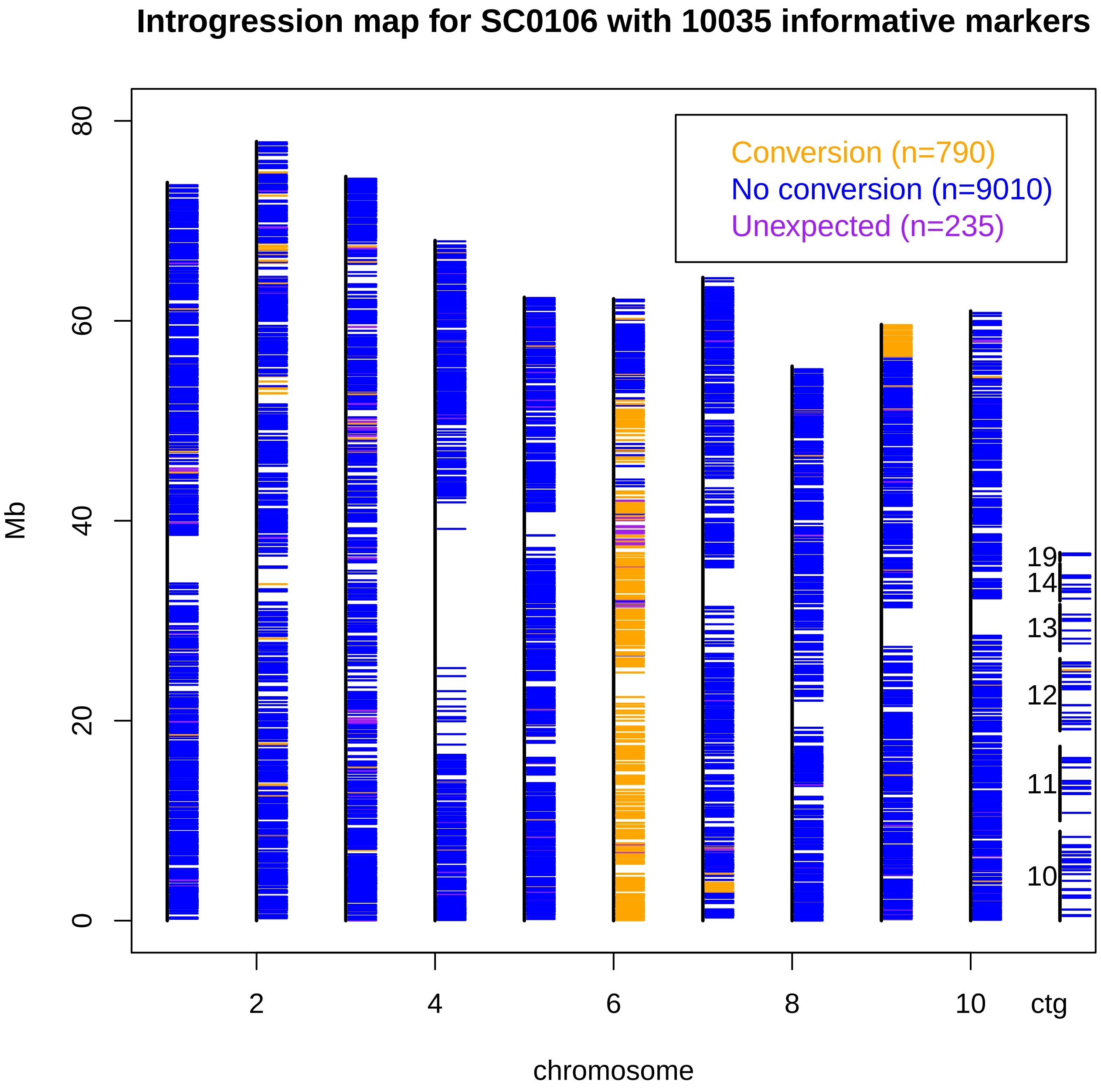
Introgression map for SC0106 with 10035 informative markers
Conversion (n=790)
No conversion (n=9010)
Unexpected (n=235)
Mb
0
20
40
60
80
2
4
6
8
10
ctg
19
14
13
12
11
10
chromosome

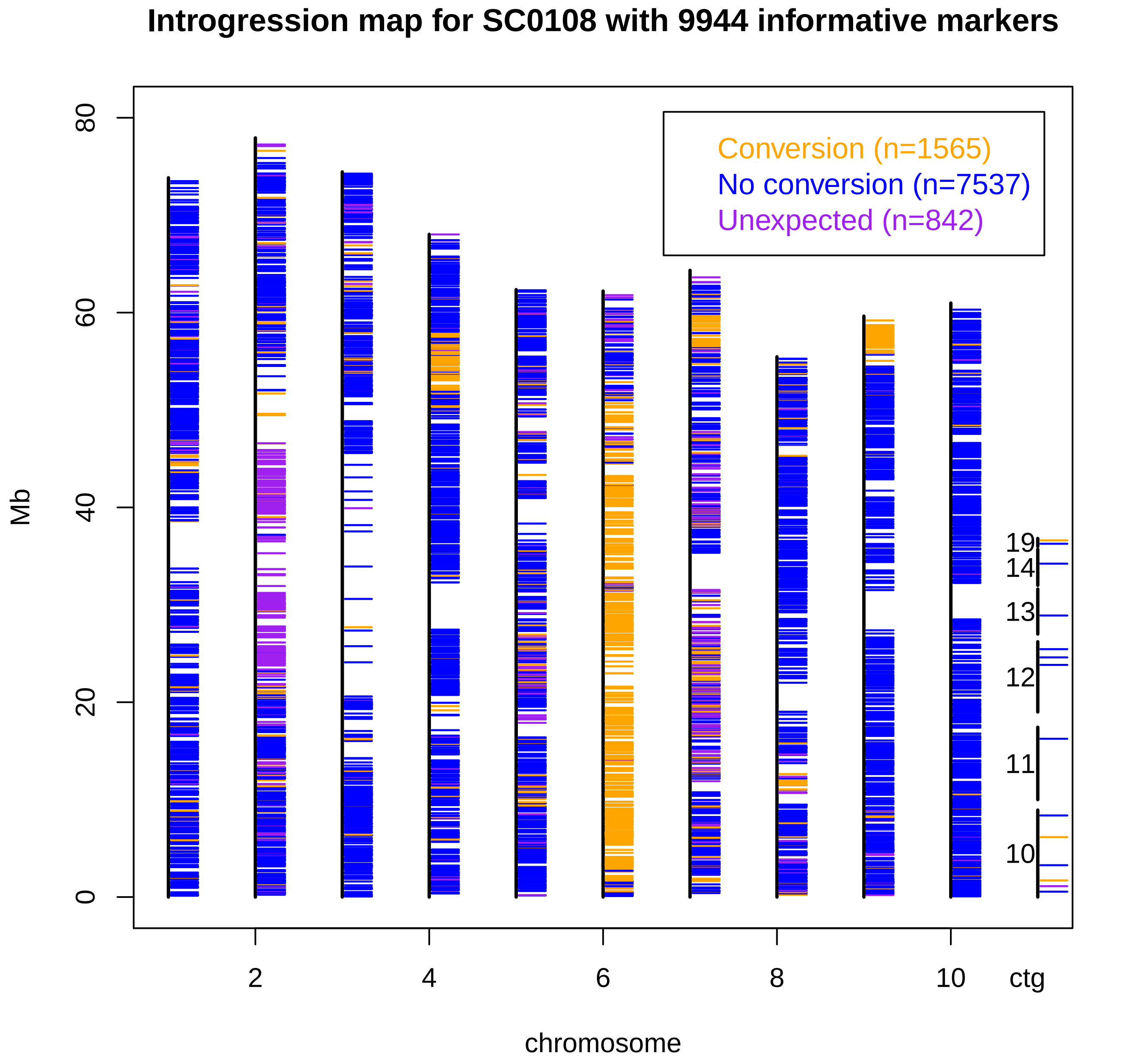

Introgression map for SC0108 with 9944 informative markers
Conversion (n=1565)
No conversion (n=7537)
Unexpected (n=842)
Mb
0
20
40
60
80
chromosome
2
4
6
8
10
ctg
19
14
13
12
11
10

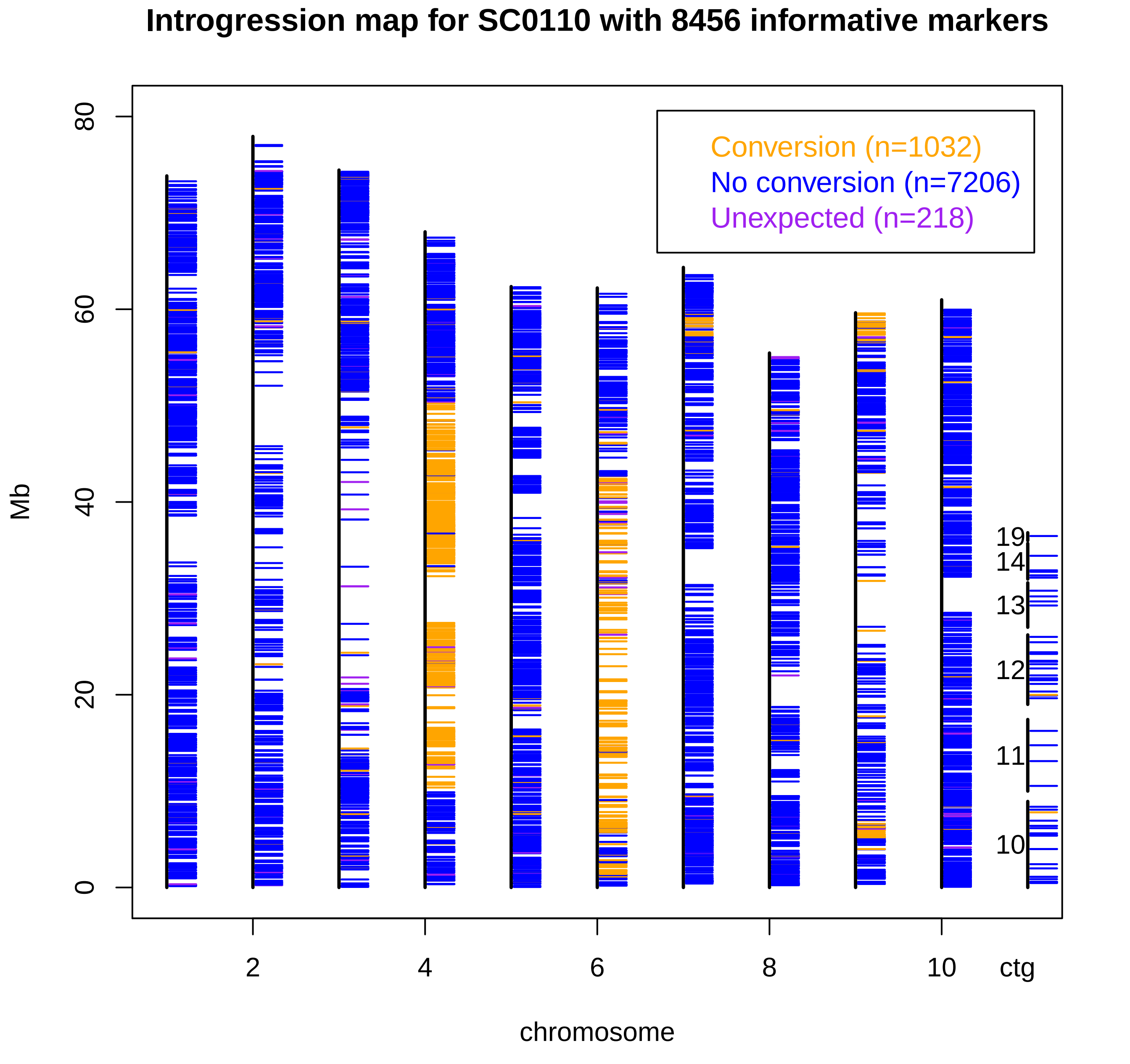
Introgression map for SC0110 with 8456 informative markers
Conversion (n=1032)
No conversion (n=7206)
Unexpected (n=218)
Mb
0
20
40
60
80
2
4
6
8
10
ctg
19
14
13
12
11
10
chromosome

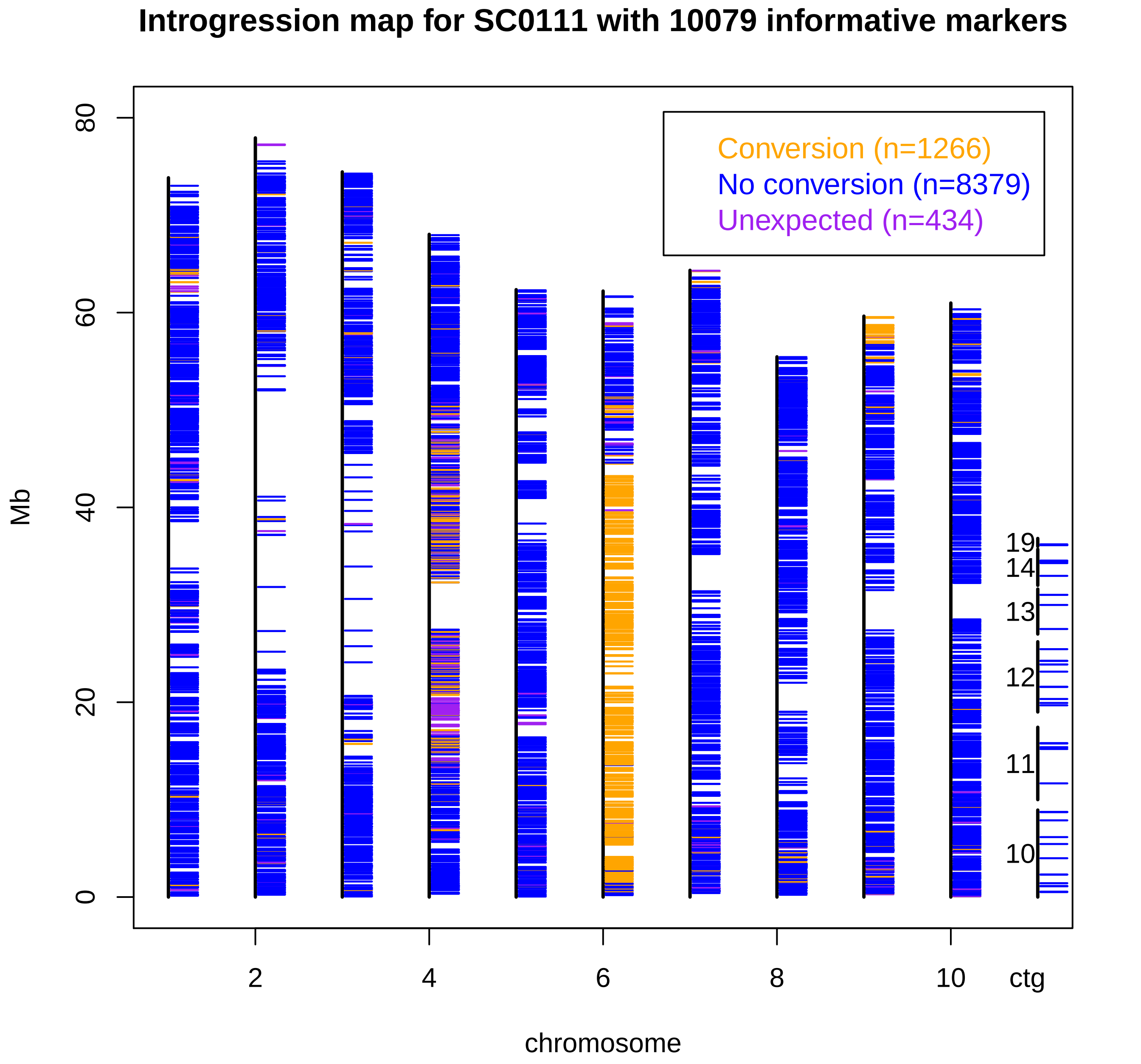

Introgression map for SC0111 with 10079 informative markers
Conversion (n=1266)
No conversion (n=8379)
Unexpected (n=434)
Mb
0
20
40
60
80
chromosome
2
4
6
8
10
ctg
19
14
13
12
11
10

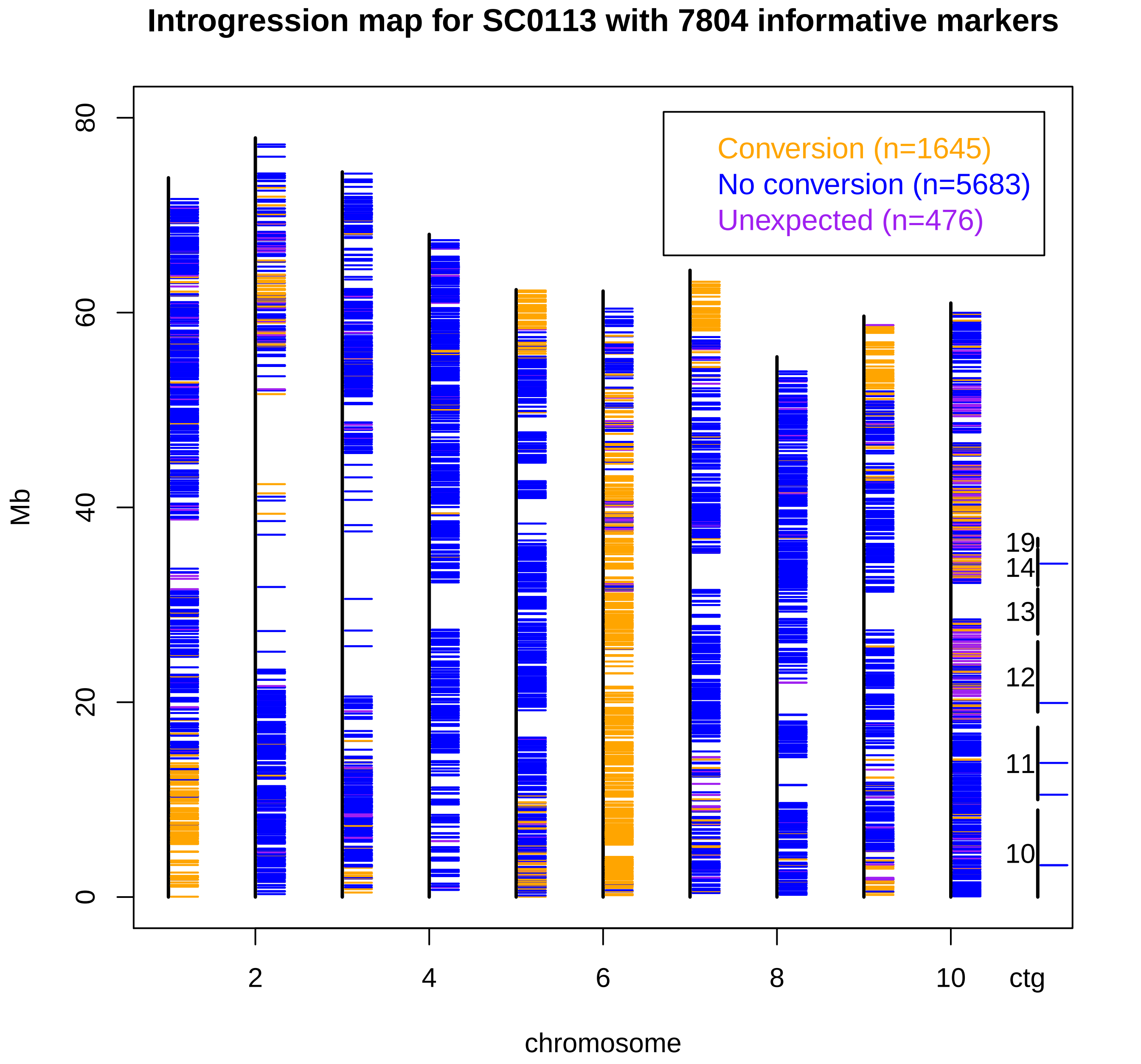

Introgression map for SC0113 with 7804 informative markers
Conversion (n=1645)
No conversion (n=5683)
Unexpected (n=476)
Mb
0
20
40
60
80
2
4
6
8
10
ctg
19
14
13
12
11
10
chromosome

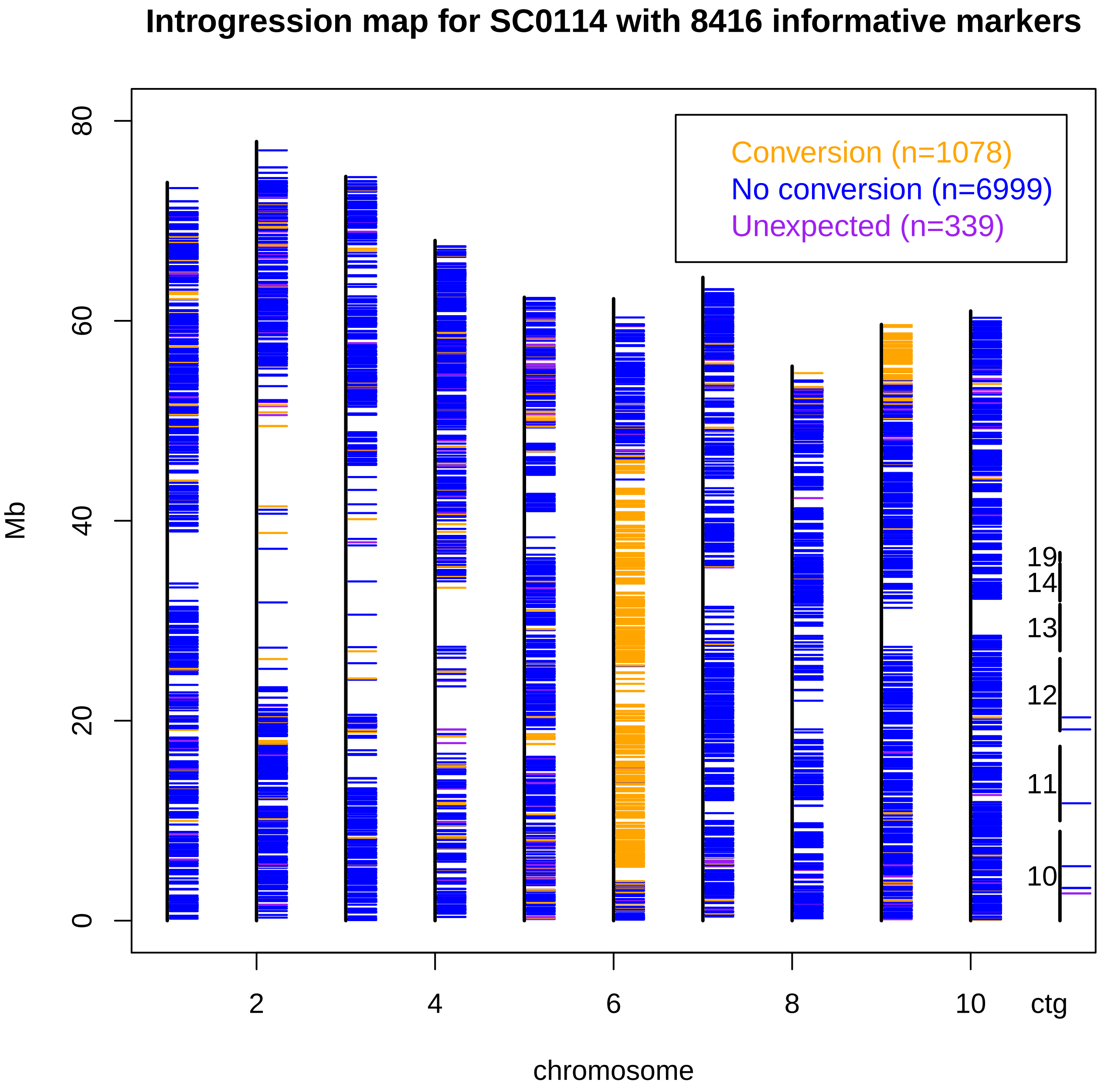

Introgression map for SC0114 with 8416 informative markers
Conversion (n=1078)
No conversion (n=6999)
Unexpected (n=339)
Mb
0
20
40
60
80
chromosome
2
4
6
8
10
ctg
19
14
13
12
11
10

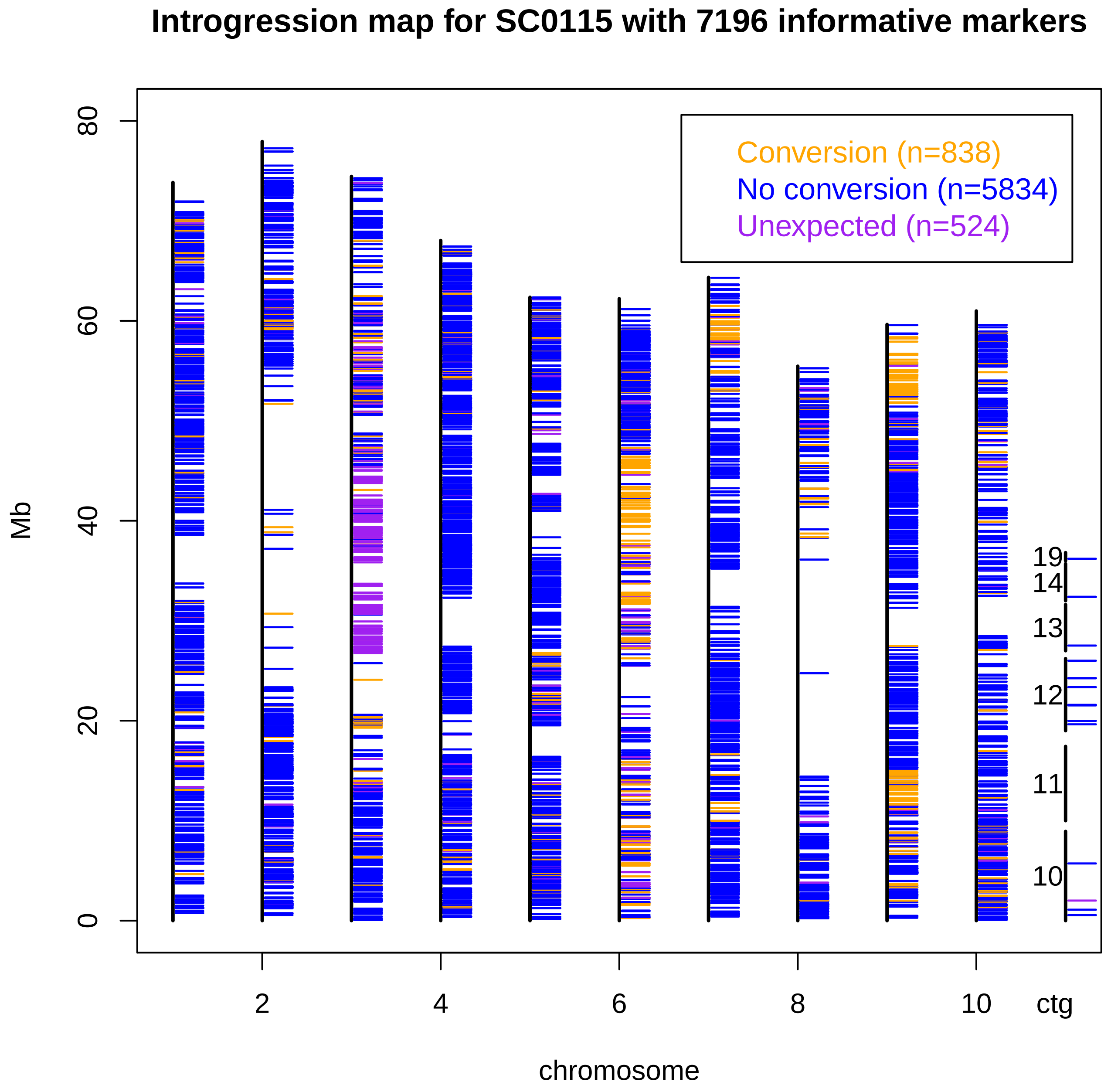
Introgression map for SC0115 with 7196 informative markers
Conversion (n=838)
No conversion (n=5834)
Unexpected (n=524)
Mb
0
20
40
60
80
chromosome
2
4
6
8
10
ctg
19
14
13
12
11
10

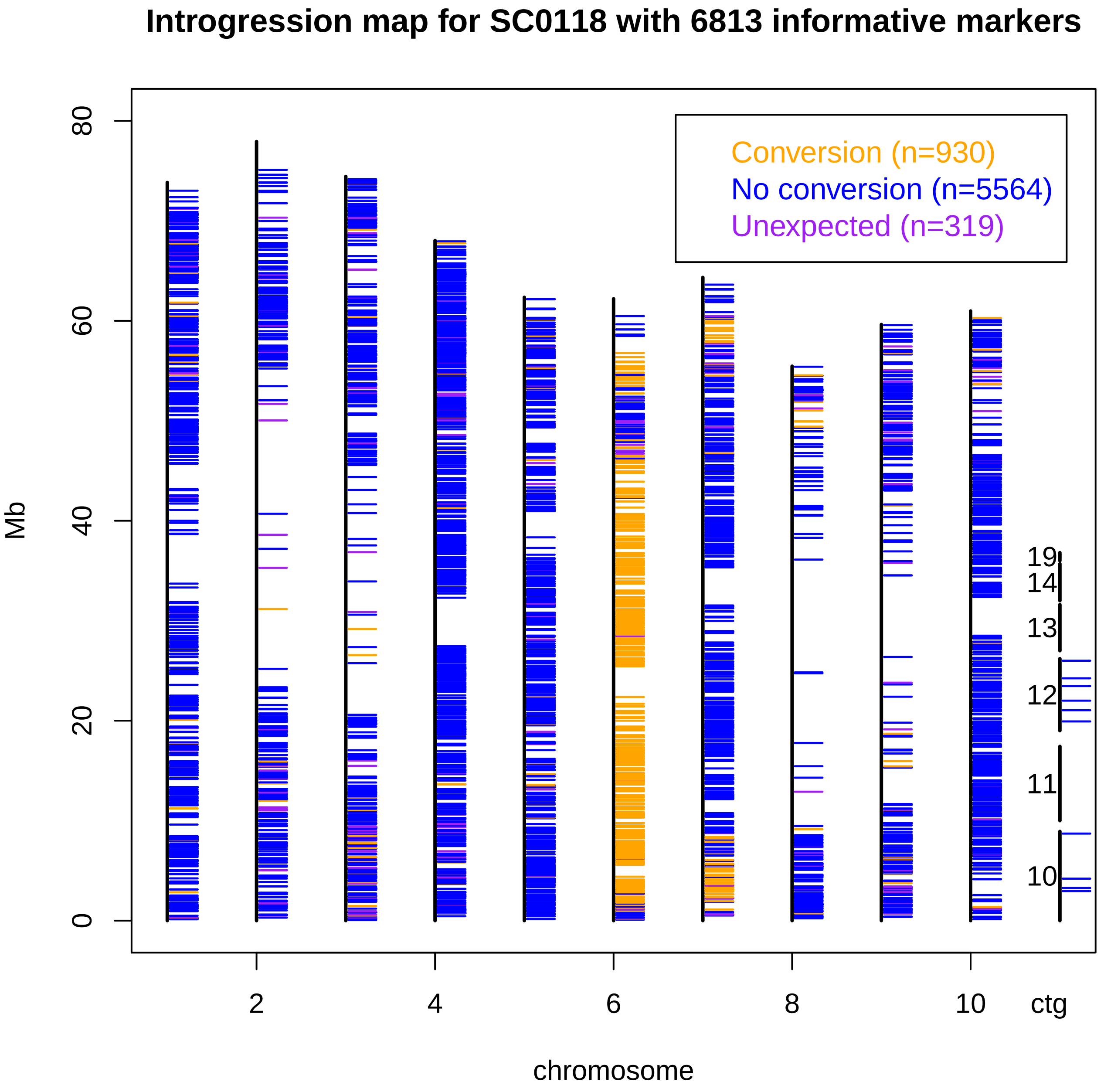

Introgression map for SC0118 with 6813 informative markers
Conversion (n=930)
No conversion (n=5564)
Unexpected (n=319)
Mb
0
20
40
60
80
chromosome
2
4
6
8
10
ctg
19
14
13
12
11
10

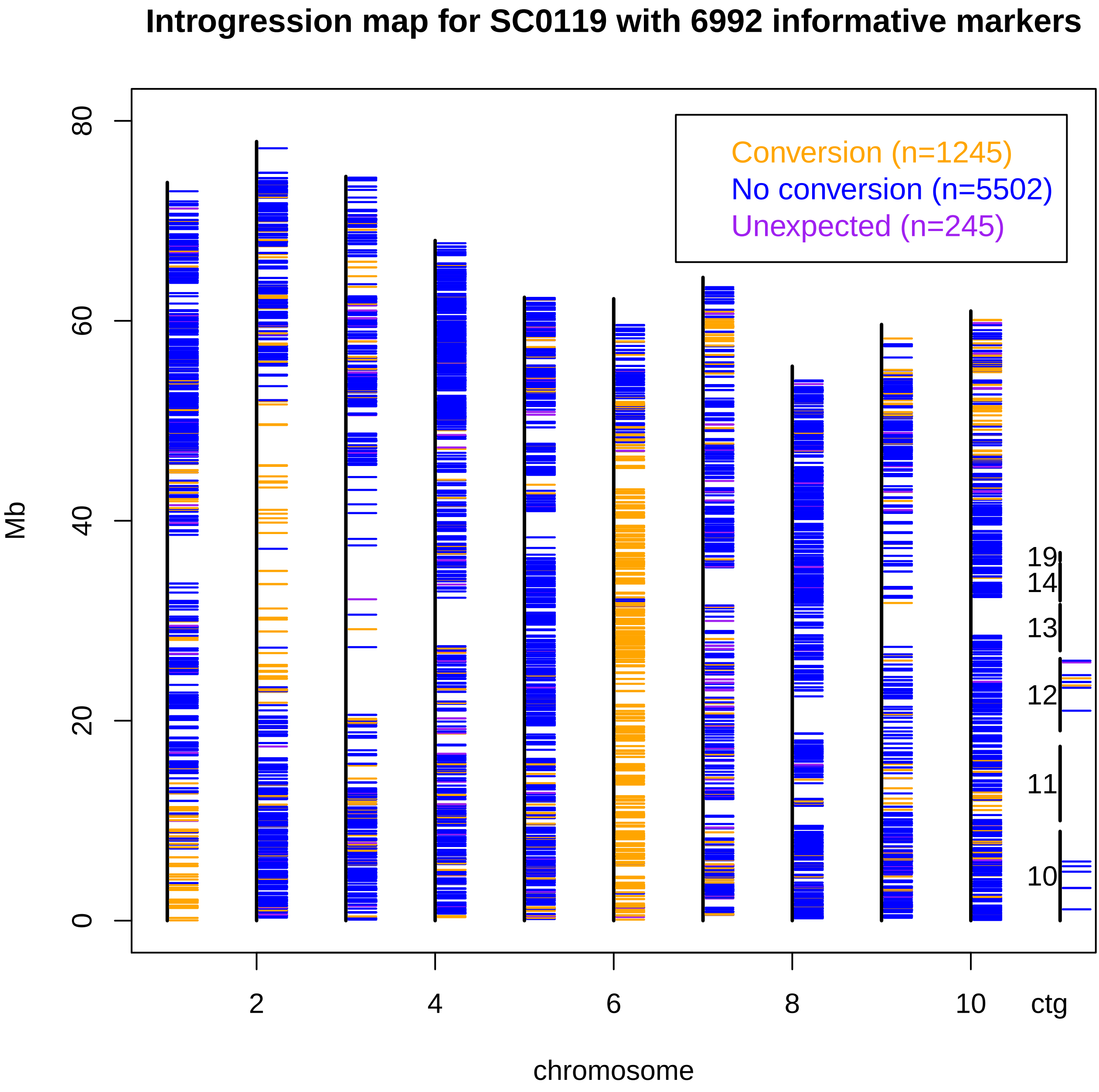

Introgression map for SC0119 with 6992 informative markers
Conversion (n=1245)
No conversion (n=5502)
Unexpected (n=245)
Mb
0
20
40
60
80
2
4
6
8
10
ctg
10
11
12
13
14
19
chromosome

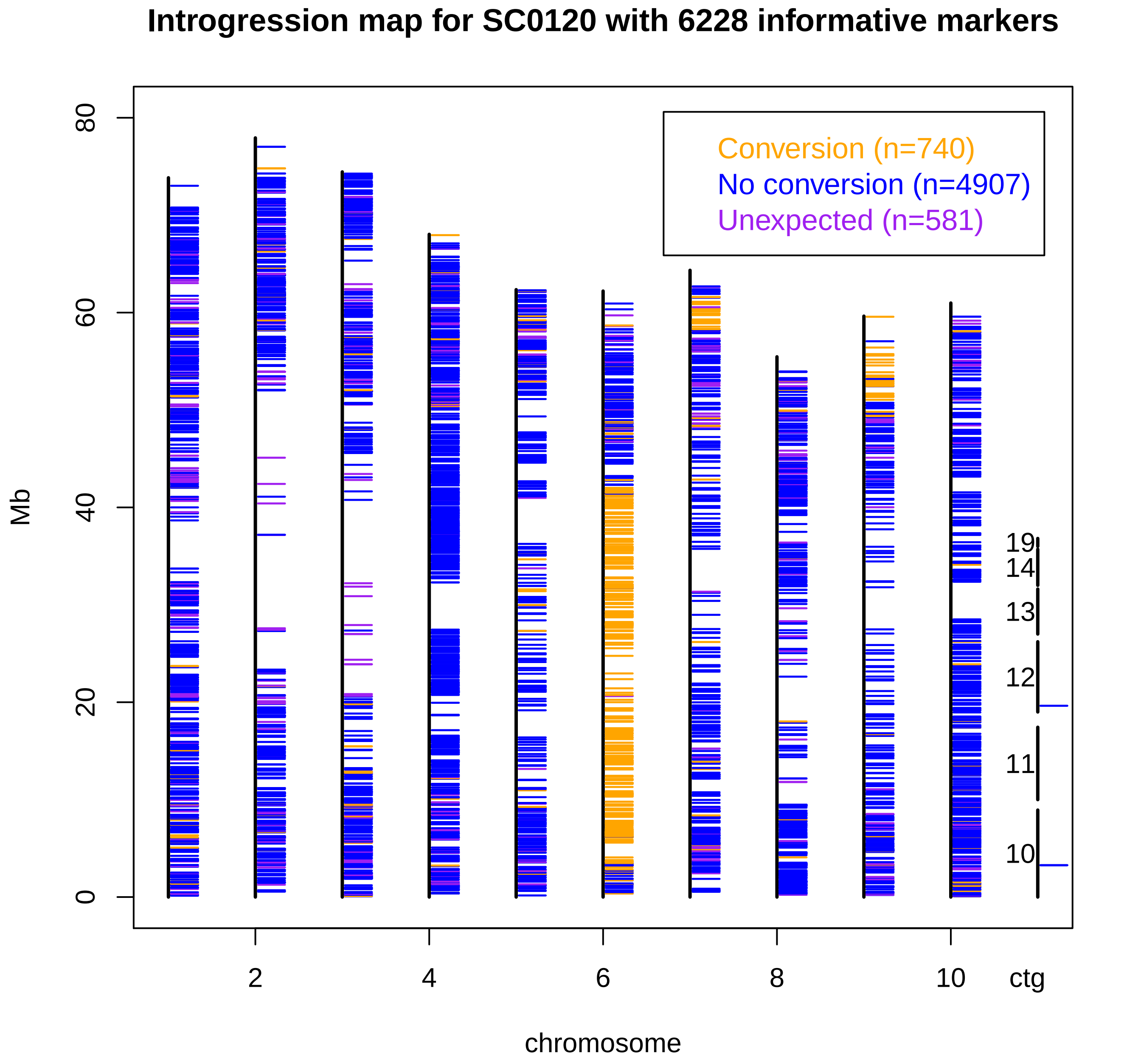
Introgression map for SC0120 with 6228 informative markers
Conversion (n=740)
No conversion (n=4907)
Unexpected (n=581)
Mb
0
20
40
60
80
chromosome
2
4
6
8
10
ctg
19
14
13
12
11
10

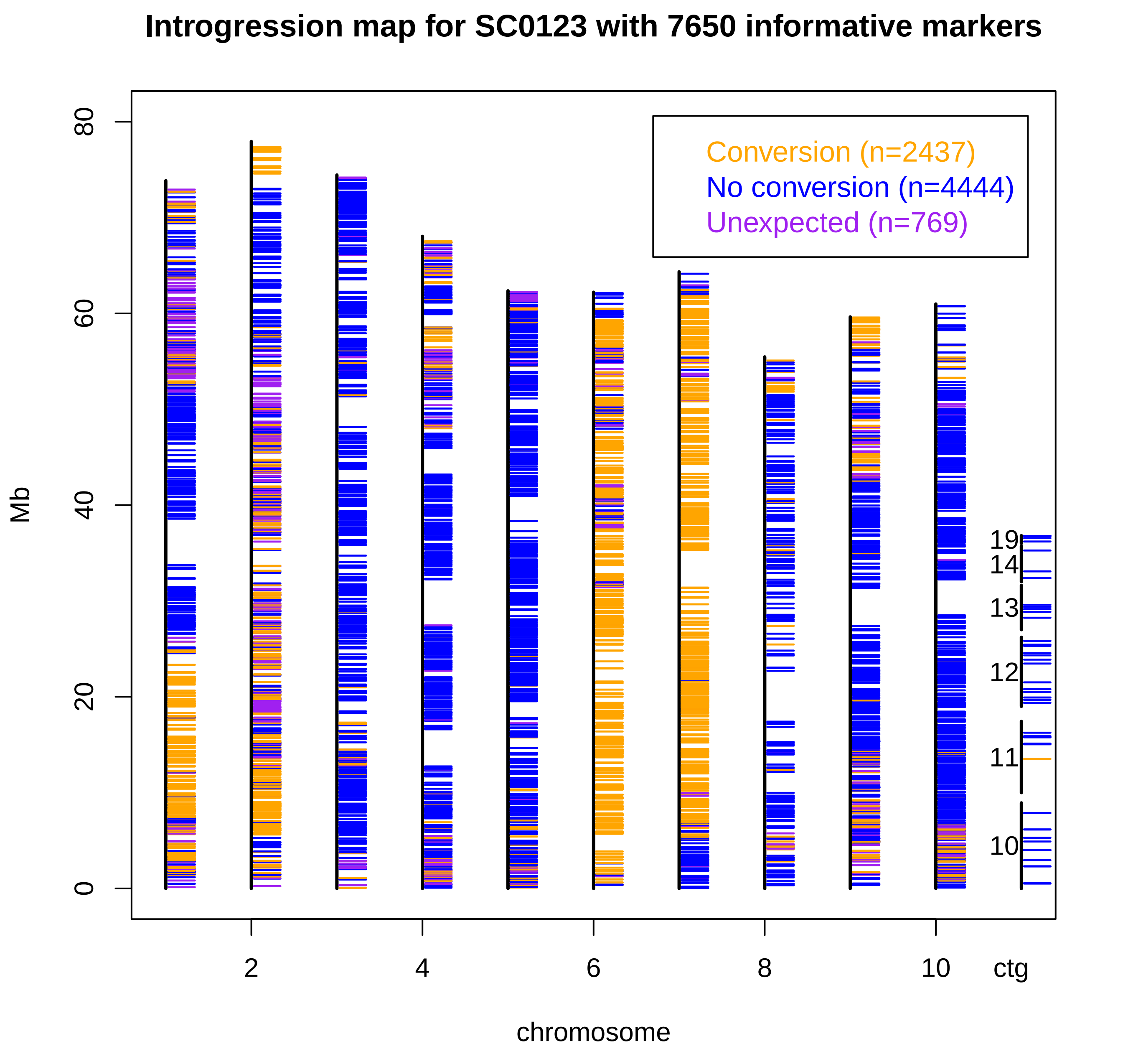

Introgression map for SC0123 with 7650 informative markers
Conversion (n=2437)
No conversion (n=4444)
Unexpected (n=769)
Mb
0
20
40
60
80
2
4
6
8
10
ctg
19
14
13
12
11
10
chromosome

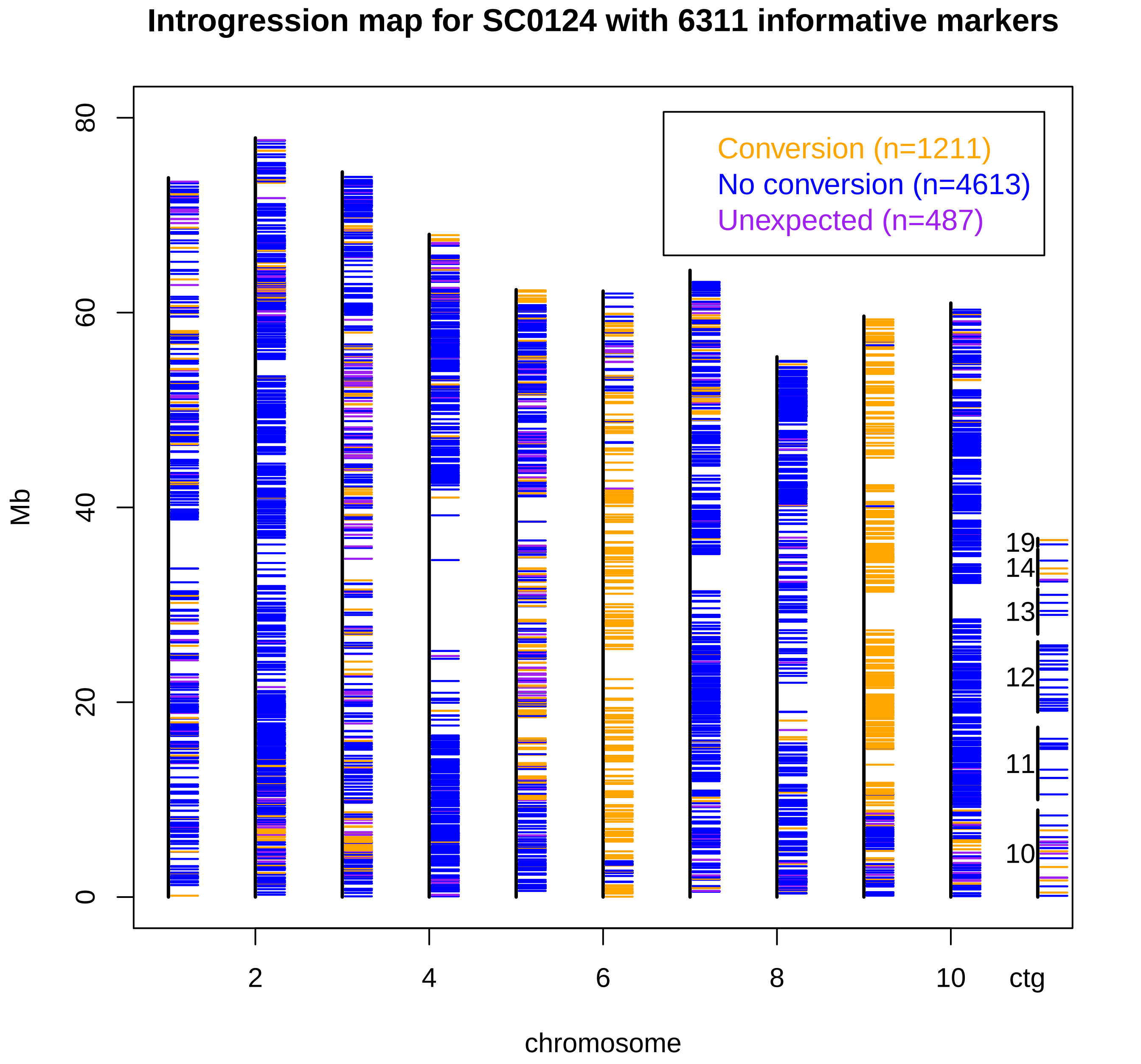

Introgression map for SC0124 with 6311 informative markers
Conversion (n=1211)
No conversion (n=4613)
Unexpected (n=487)
Mb
0
20
40
60
80
2
4
6
8
10
ctg
19
14
13
12
11
10
chromosome

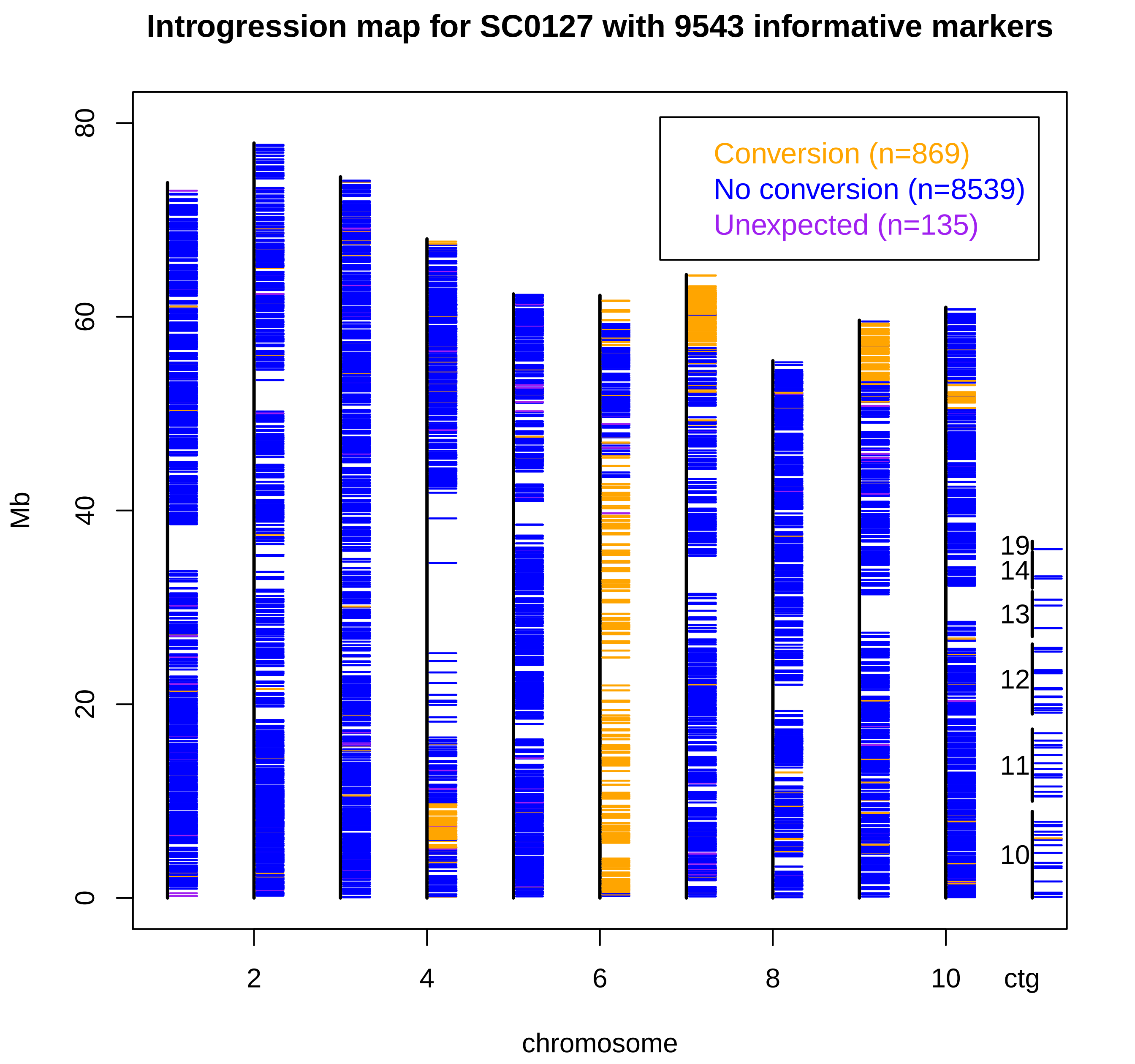

Introgression map for SC0127 with 9543 informative markers
Conversion (n=869)
No conversion (n=8539)
Unexpected (n=135)
Mb
0
20
40
60
80
chromosome
2
4
6
8
10
ctg
19
14
13
12
11
10

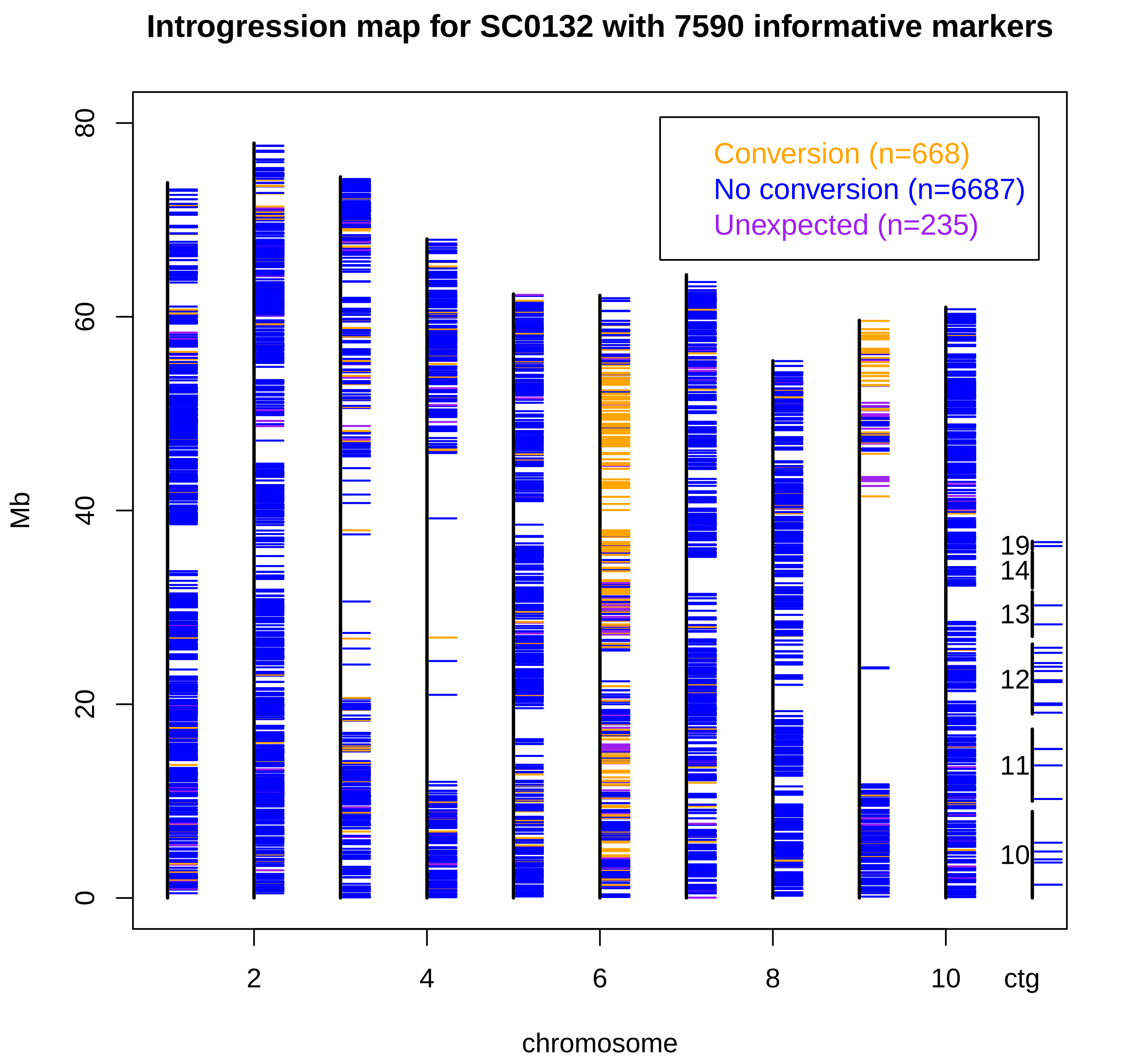

Introgression map for SC0132 with 7590 informative markers
Conversion (n=668)
No conversion (n=6687)
Unexpected (n=235)
Mb
0
20
40
60
80
2
4
6
8
10
ctg
19
14
13
12
11
10
chromosome

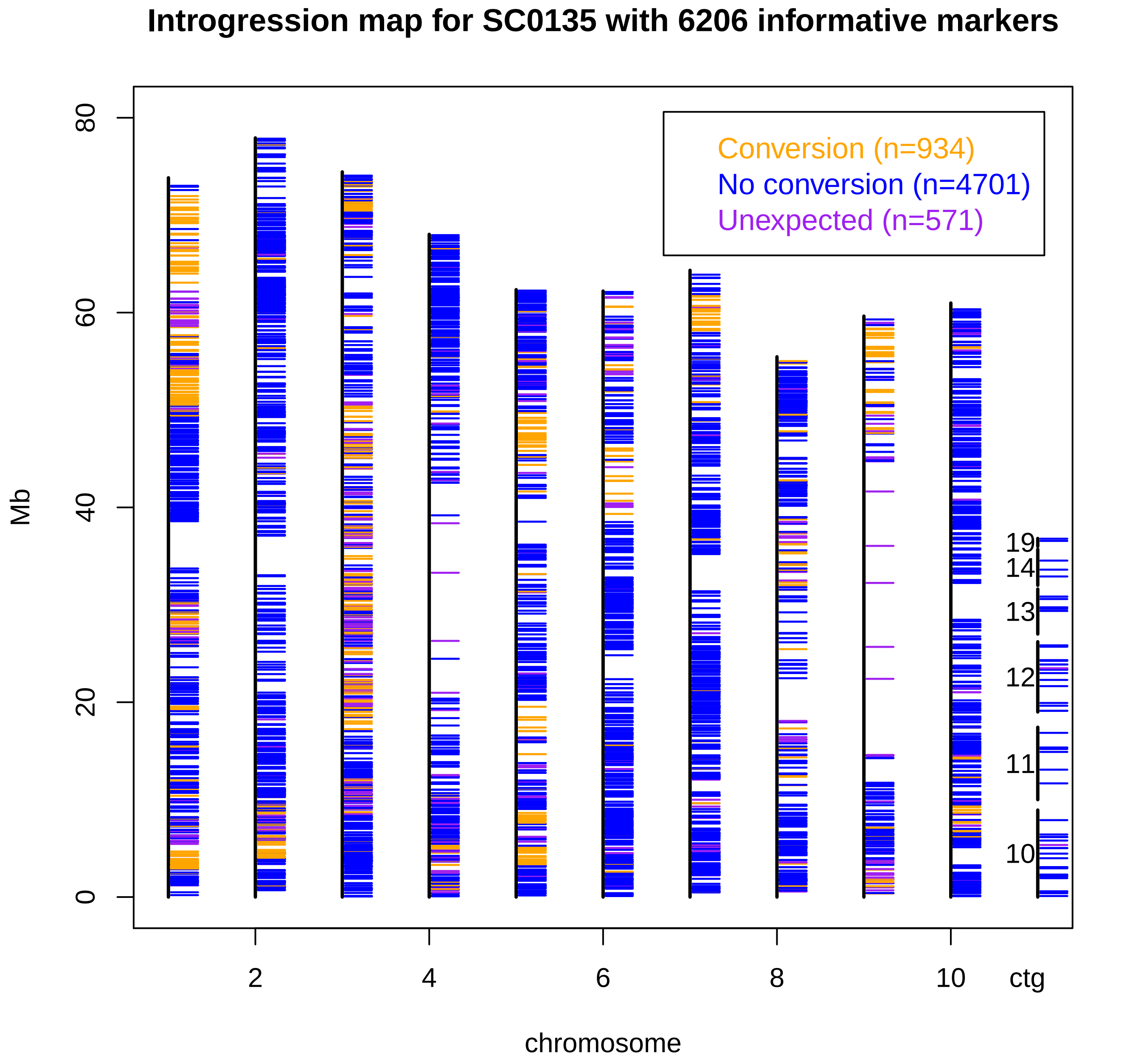

Introgression map for SC0135 with 6206 informative markers
Conversion (n=934)
No conversion (n=4701)
Unexpected (n=571)
Mb
0
20
40
60
80
chromosome
2
4
6
8
10
ctg
19
14
13
12
11
10

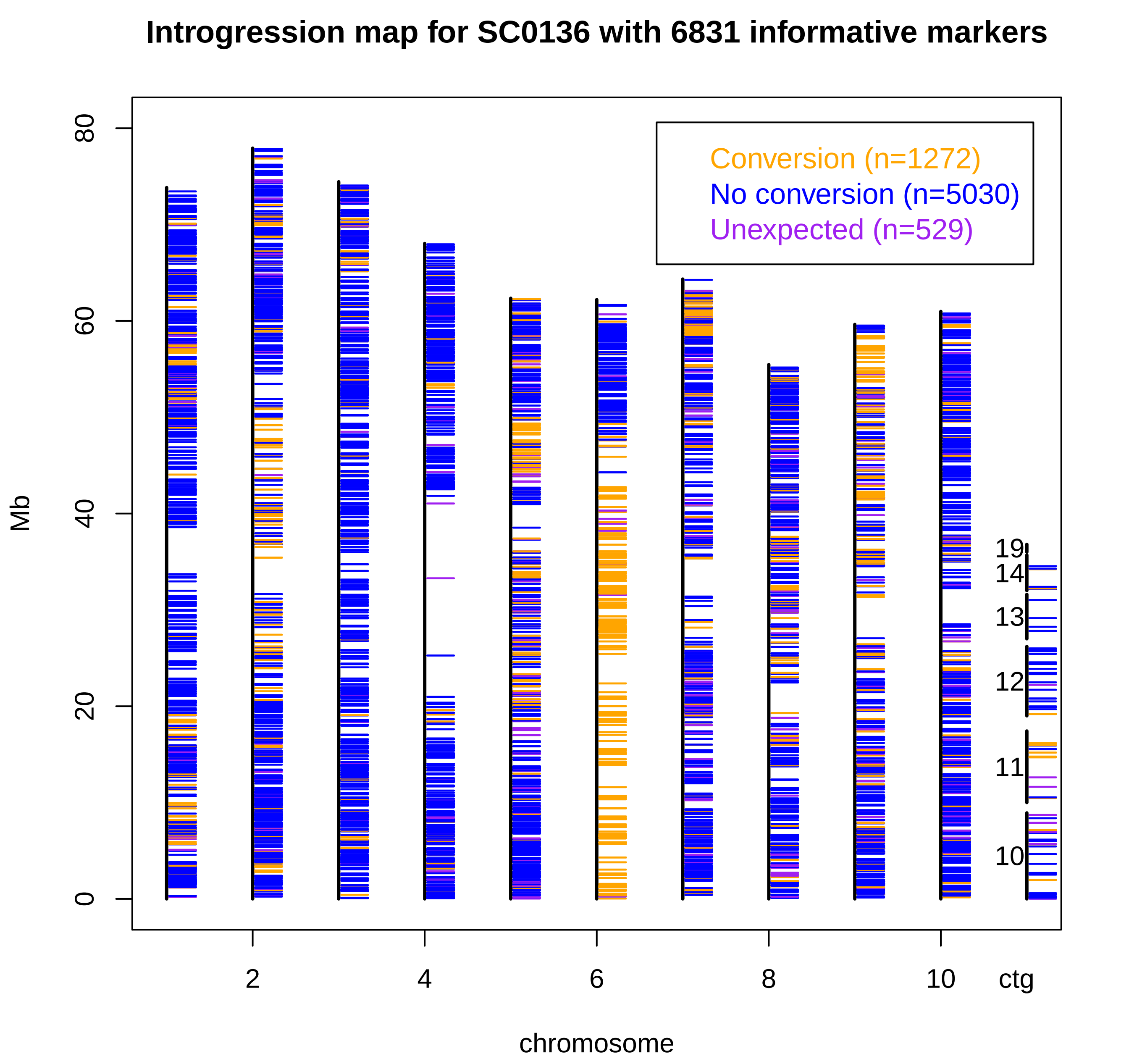

Introgression map for SC0136 with 6831 informative markers
Conversion (n=1272)
No conversion (n=5030)
Unexpected (n=529)
Mb
0
20
40
60
80
2
4
6
8
10
ctg
19
14
13
12
11
10
chromosome

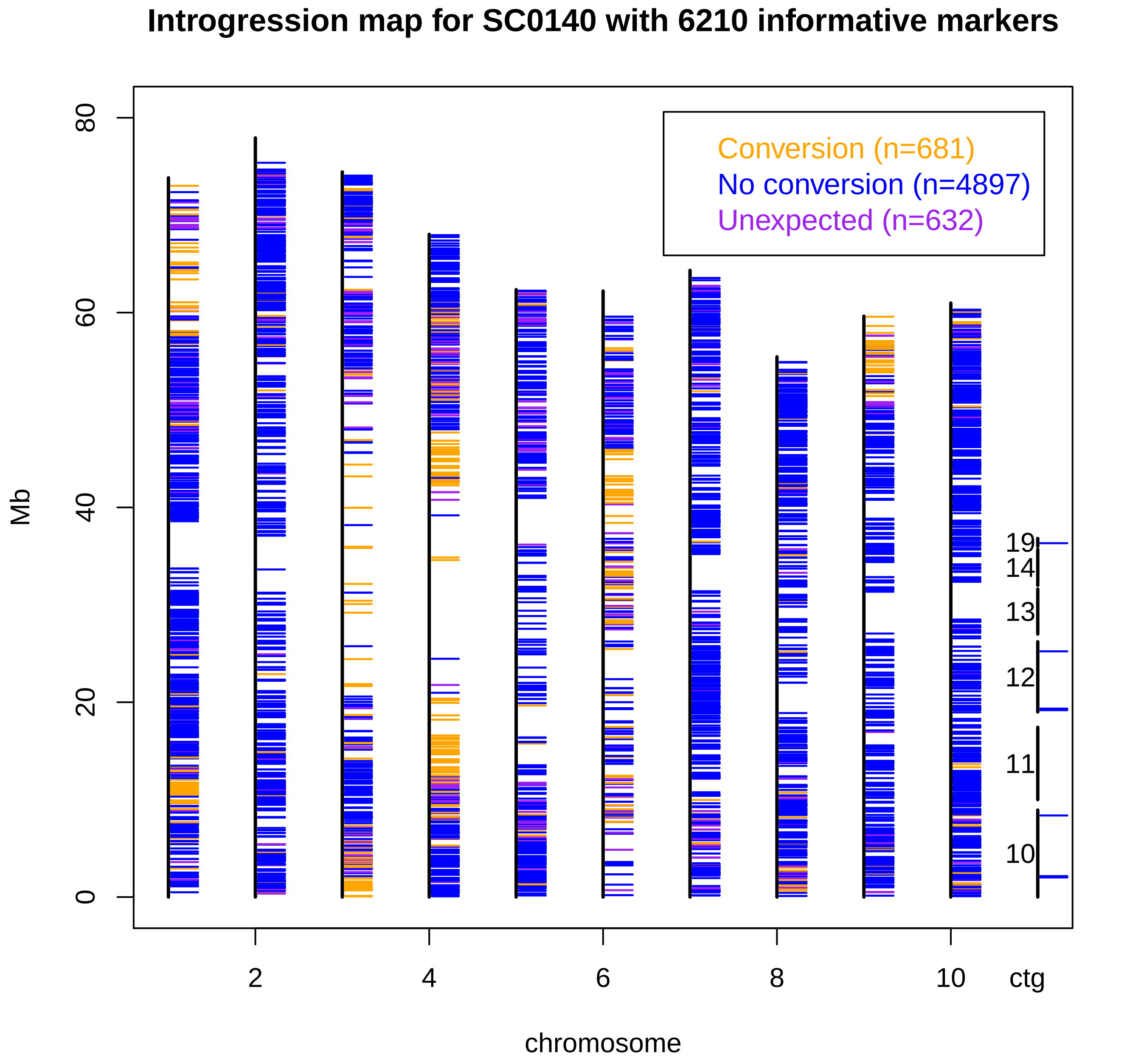
Introgression map for SC0140 with 6210 informative markers
Conversion (n=681)
No conversion (n=4897)
Unexpected (n=632)
Mb
0
20
40
60
80
chromosome
2
4
6
8
10
ctg
19
14
13
12
11
10

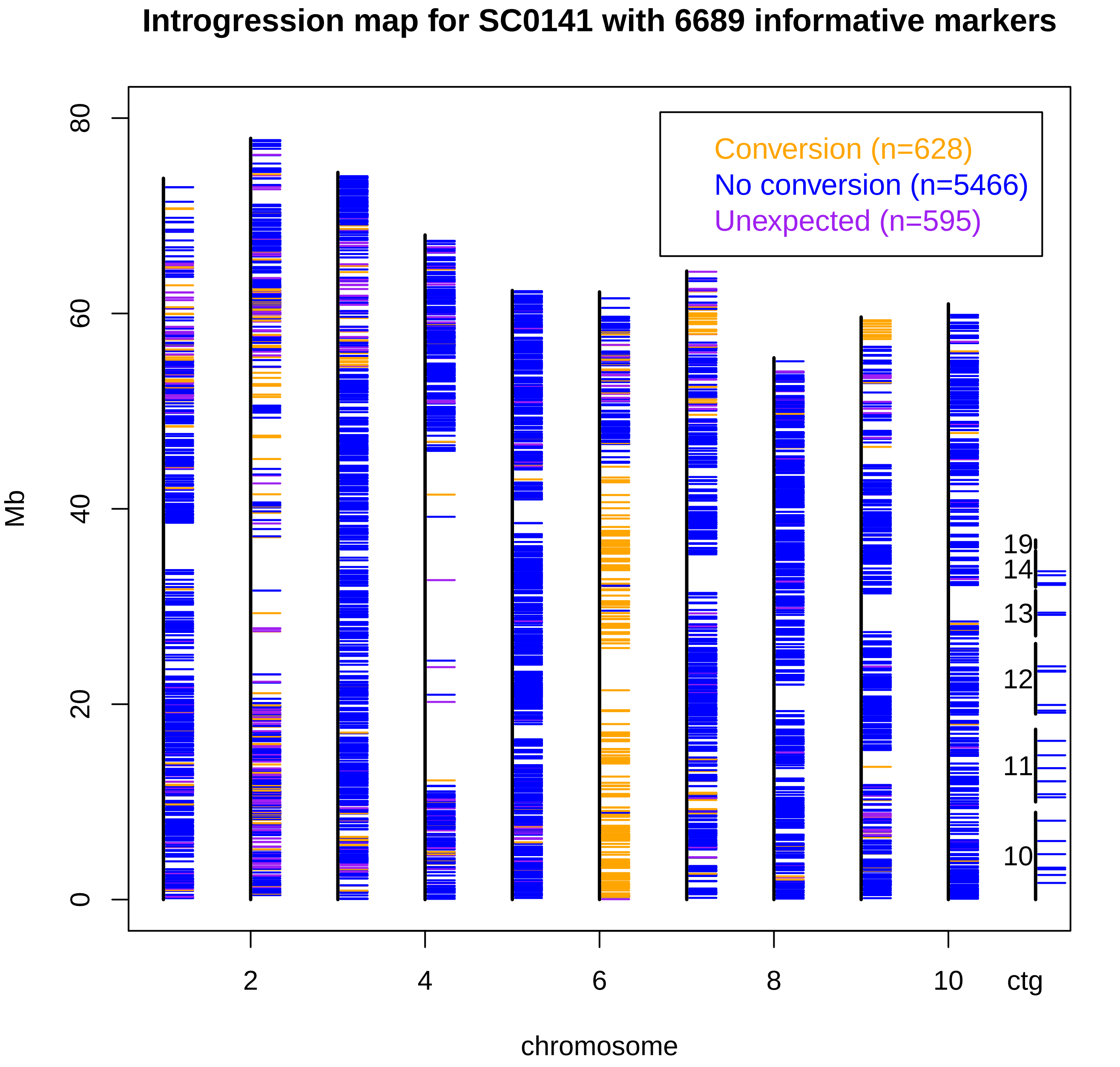
Introgression map for SC0141 with 6689 informative markers
Conversion (n=628)
No conversion (n=5466)
Unexpected (n=595)
Mb
0
20
40
60
80
2
4
6
8
10
ctg
19
14
13
12
11
10
chromosome

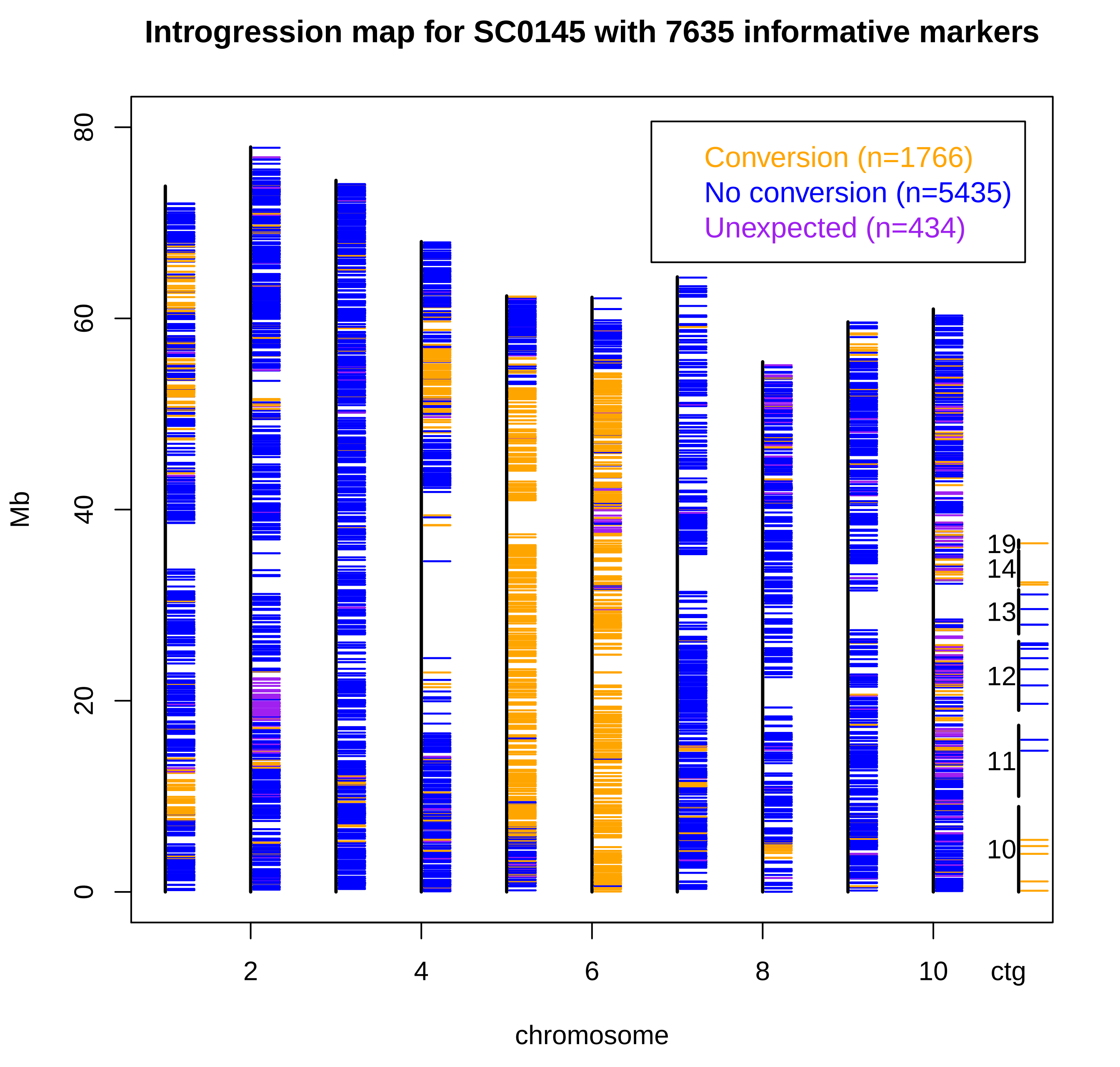
Introgression map for SC0145 with 7635 informative markers
Conversion (n=1766)
No conversion (n=5435)
Unexpected (n=434)
Mb
0
20
40
60
80
chromosome
2
4
6
8
10
ctg
19
14
13
12
11
10

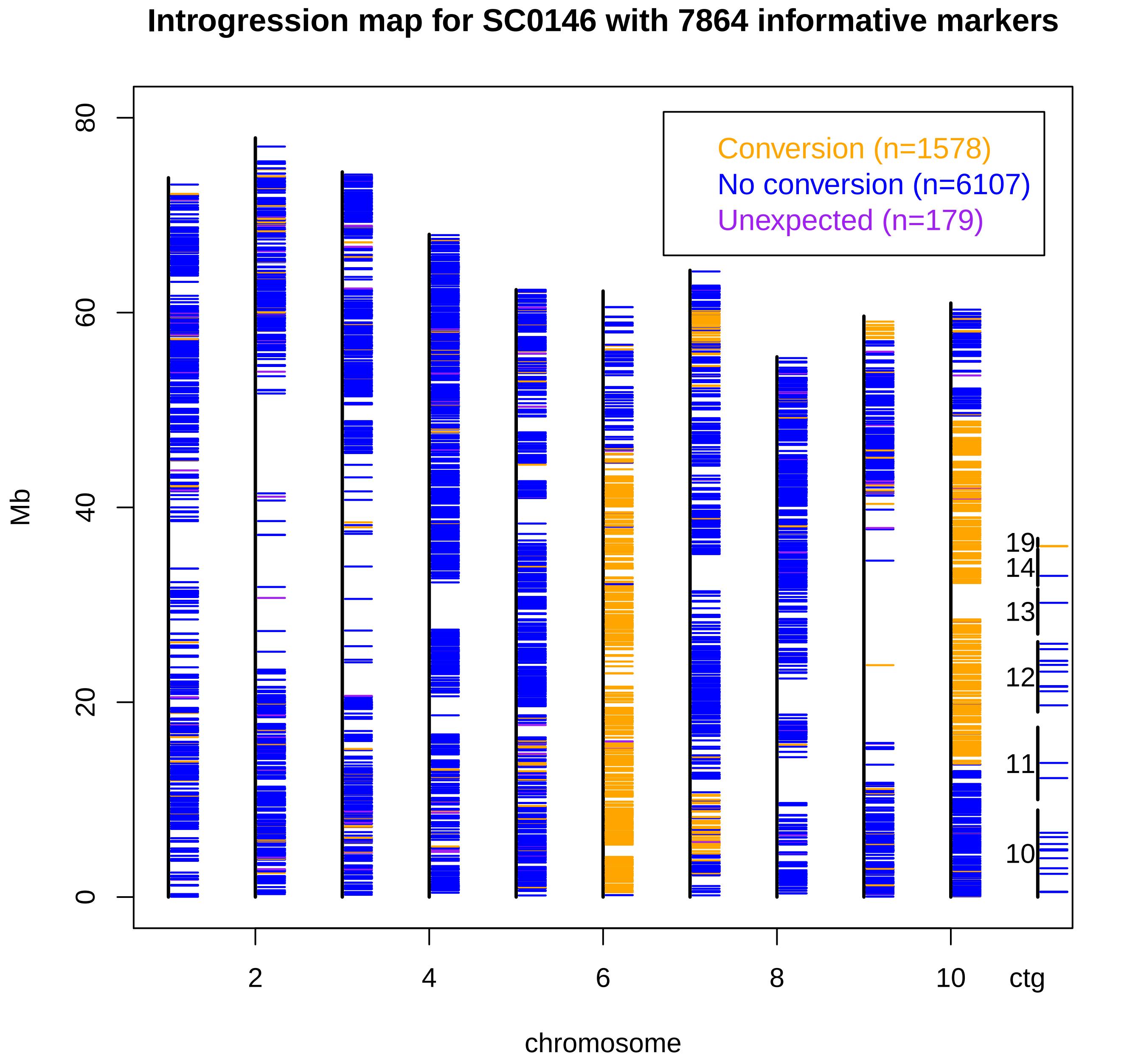

Introgression map for SC0146 with 7864 informative markers
Conversion (n=1578)
No conversion (n=6107)
Unexpected (n=179)
Mb
0
20
40
60
80
chromosome
2
4
6
8
10
ctg
19
14
13
12
11
10

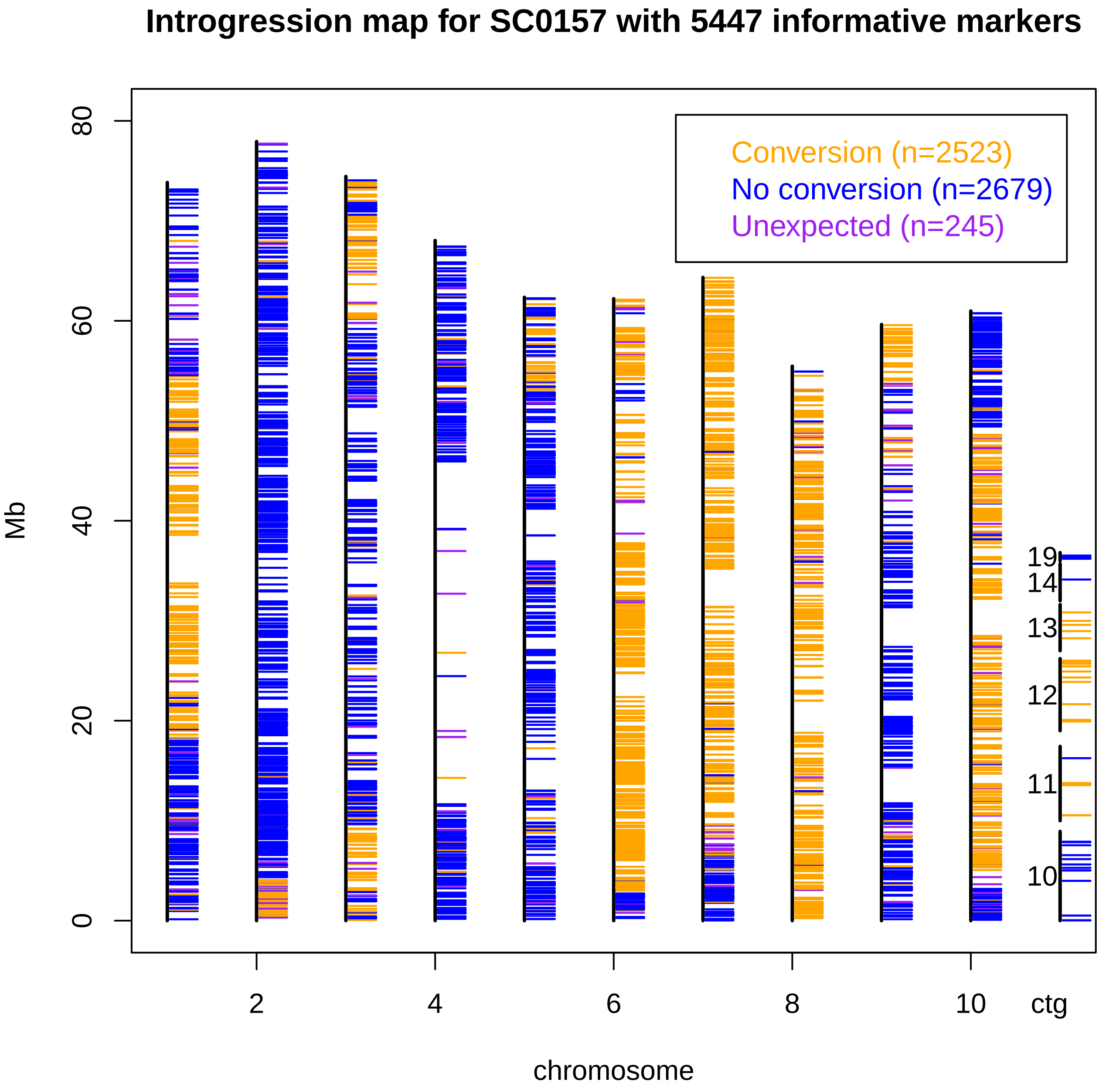

Introgression map for SC0157 with 5447 informative markers
Conversion (n=2523)
No conversion (n=2679)
Unexpected (n=245)
Mb
0
20
40
60
80
2
4
6
8
10
ctg
19
14
13
12
11
10
chromosome

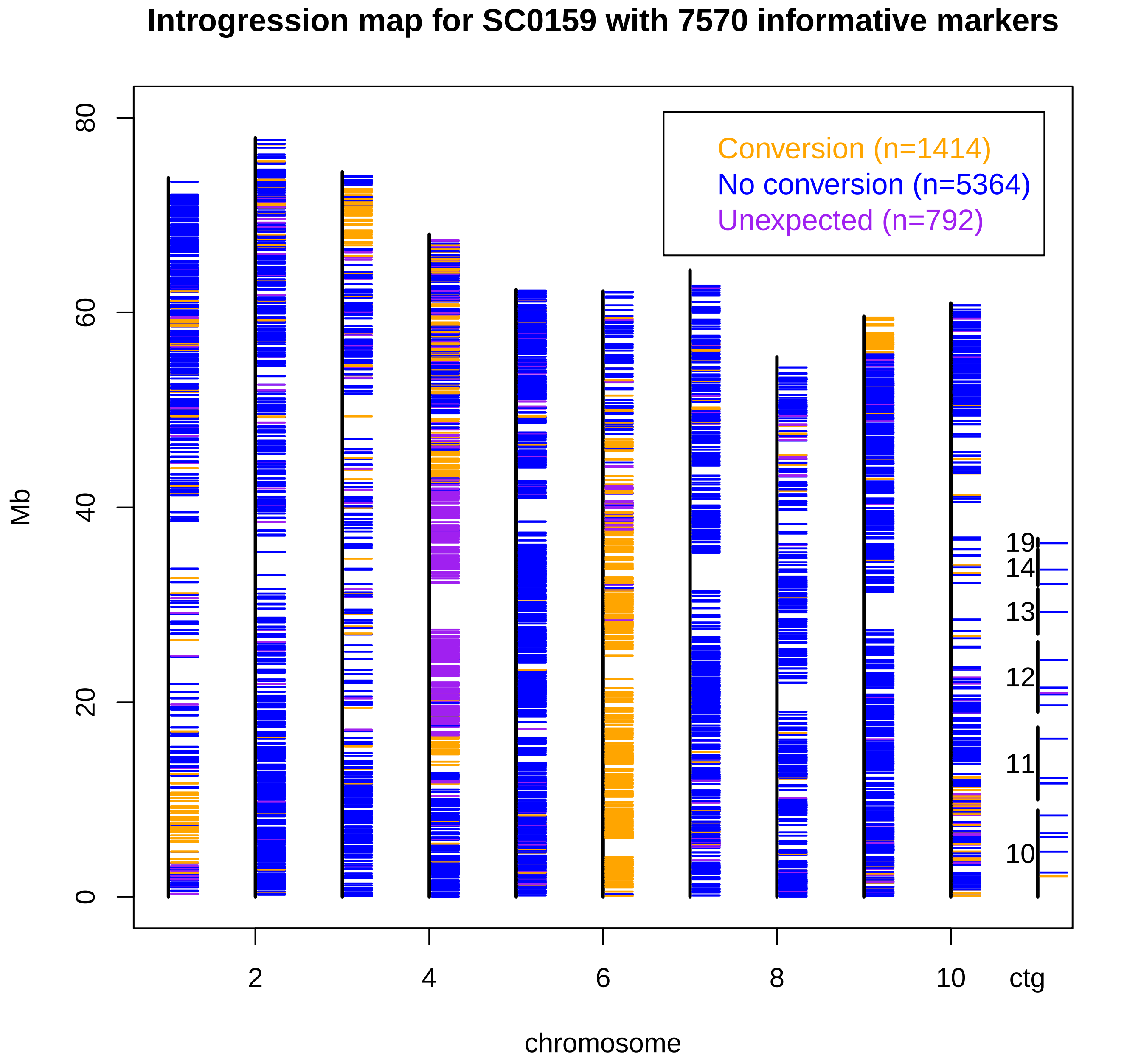

Introgression map for SC0159 with 7570 informative markers
Conversion (n=1414)
No conversion (n=5364)
Unexpected (n=792)
Mb
0
20
40
60
80
chromosome
2
4
6
8
10
ctg
19
14
13
12
11
10

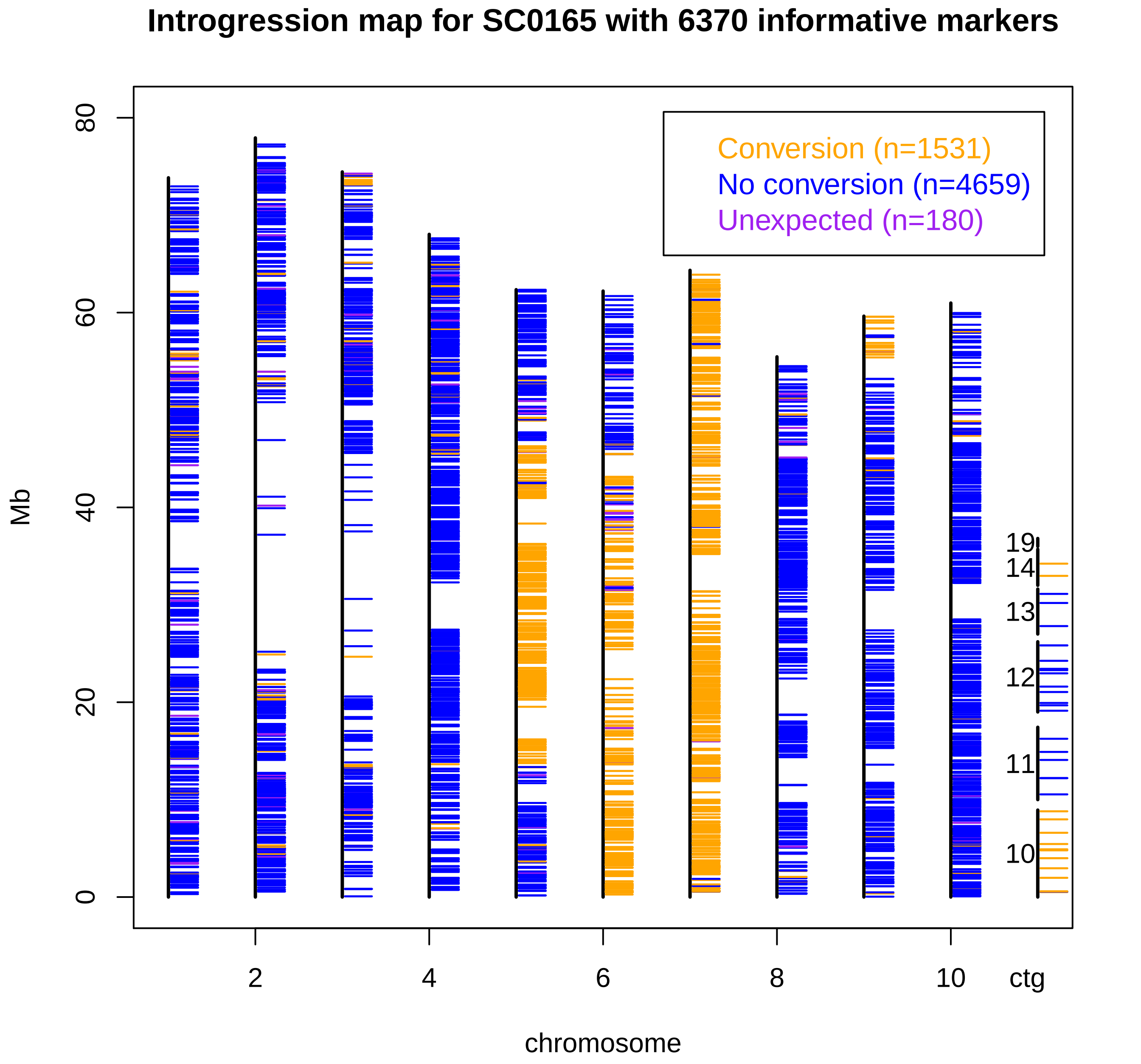
Introgression map for SC0165 with 6370 informative markers
Conversion (n=1531)
No conversion (n=4659)
Unexpected (n=180)
Mb
0
20
40
60
80
2
4
6
8
10
ctg
19
14
13
12
11
10
chromosome

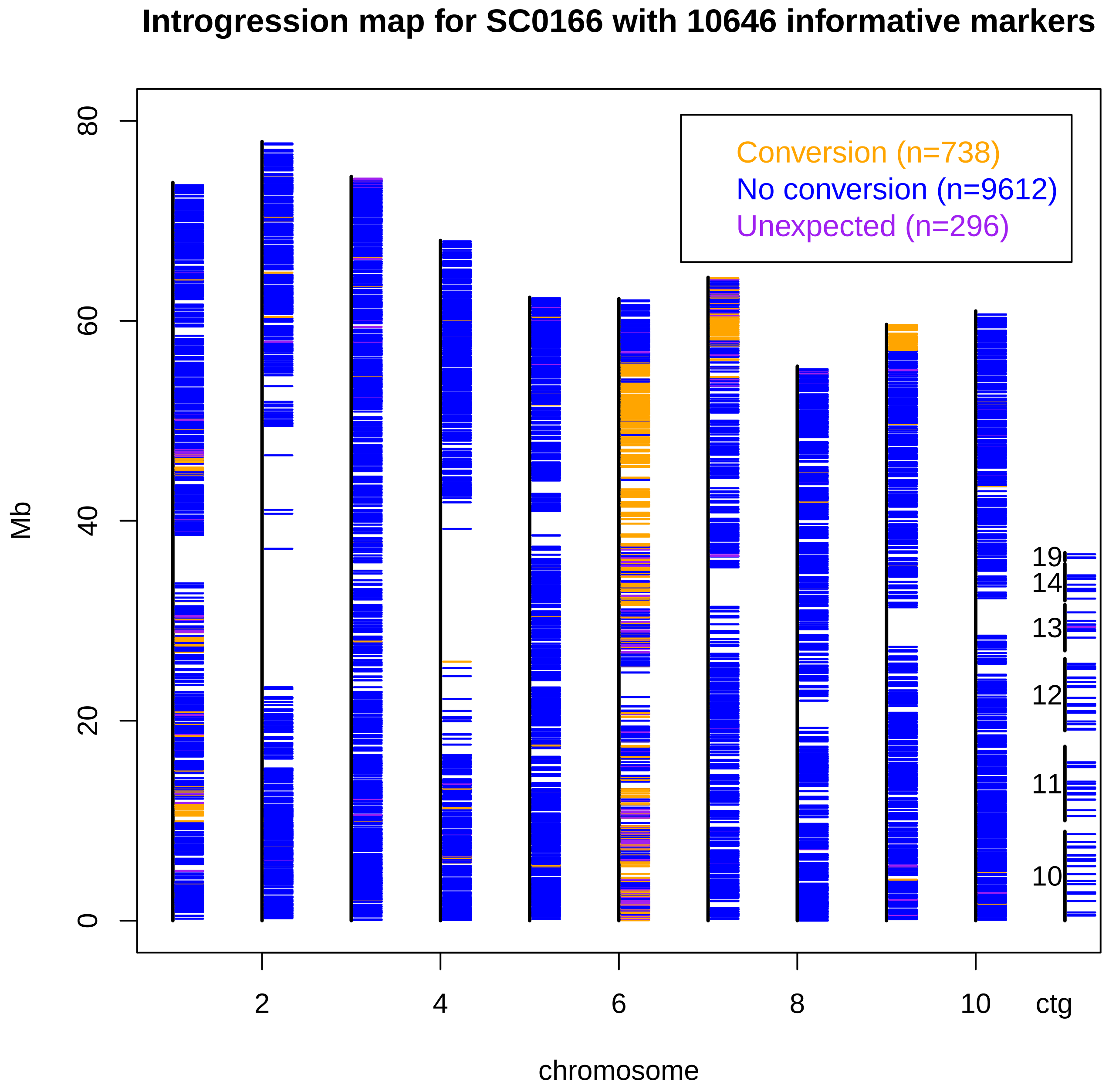
Introgression map for SC0166 with 10646 informative markers
Conversion (n=738)
No conversion (n=9612)
Unexpected (n=296)
Mb
0
20
40
60
80
chromosome
2
4
6
8
10
ctg
19
14
13
12
11
10

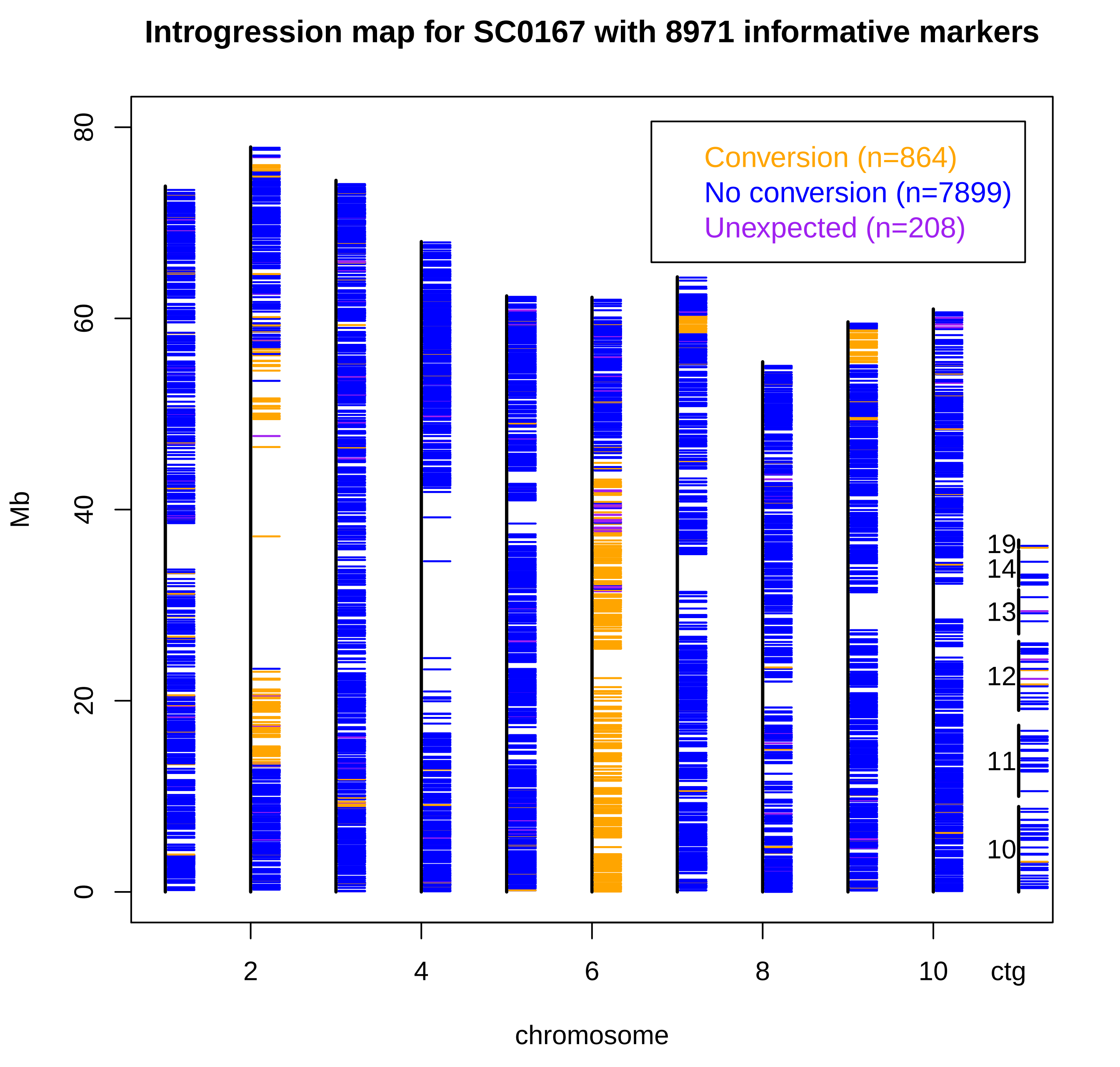
Introgression map for SC0167 with 8971 informative markers
Conversion (n=864)
No conversion (n=7899)
Unexpected (n=208)
Mb
0
20
40
60
80
chromosome
2
4
6
8
10
ctg
19
14
13
12
11
10

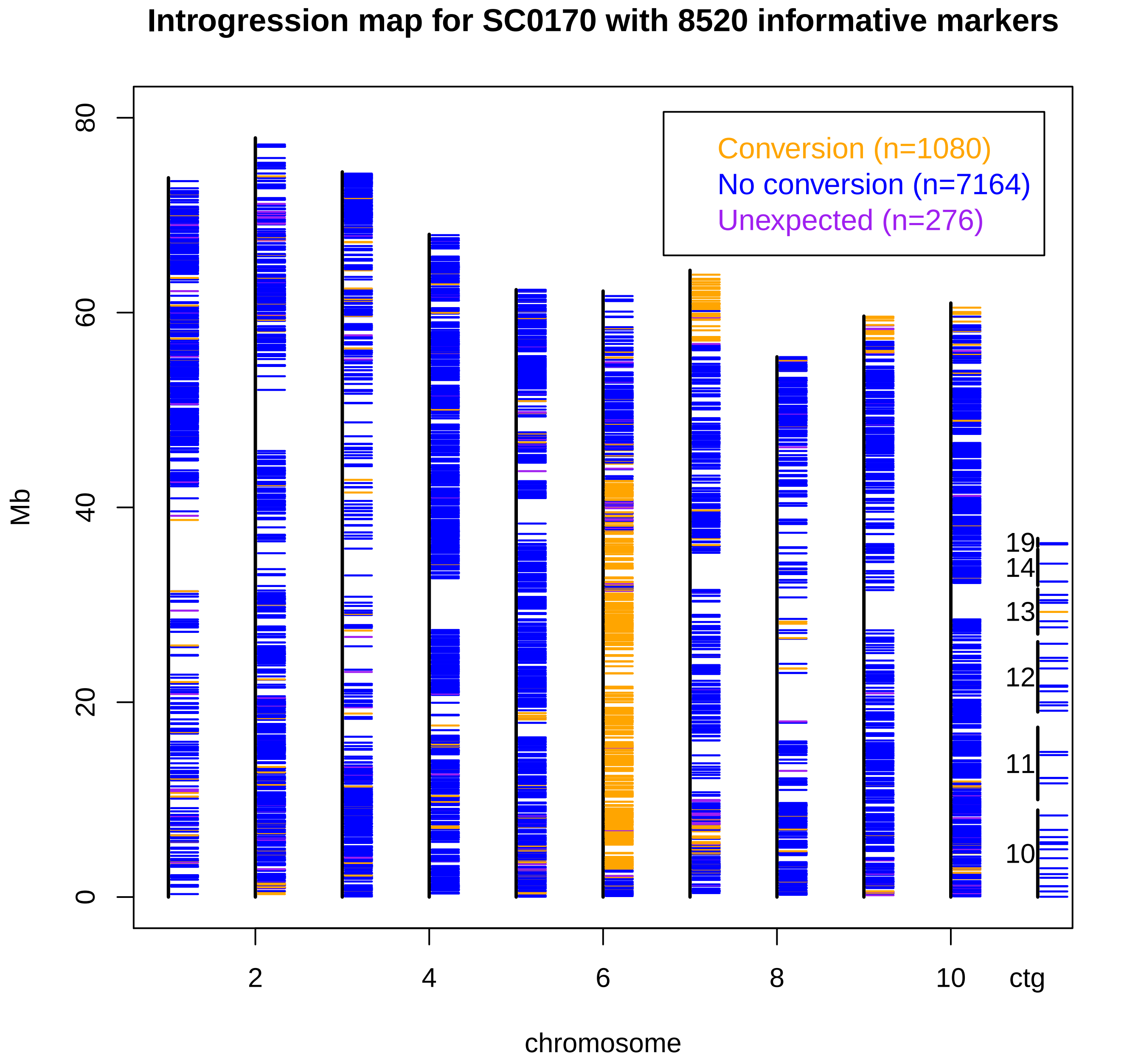

Introgression map for SC0170 with 8520 informative markers
Conversion (n=1080)
No conversion (n=7164)
Unexpected (n=276)
Mb
0
20
40
60
80
2
4
6
8
10
ctg
19
14
13
12
11
10
chromosome

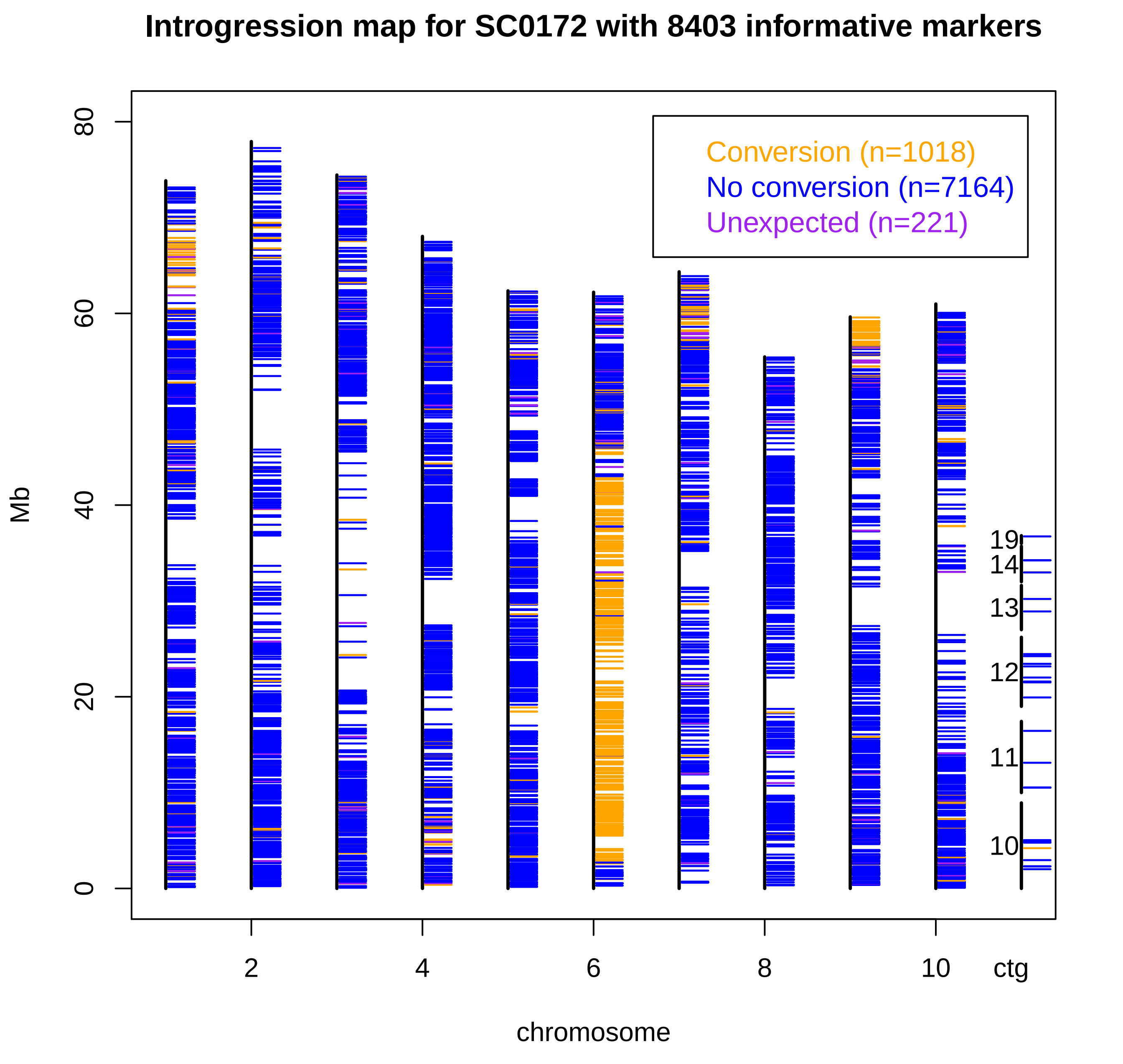
Introgression map for SC0172 with 8403 informative markers
Conversion (n=1018)
No conversion (n=7164)
Unexpected (n=221)
Mb
0
20
40
60
80
2
4
6
8
10
ctg
19
14
13
12
11
10
chromosome

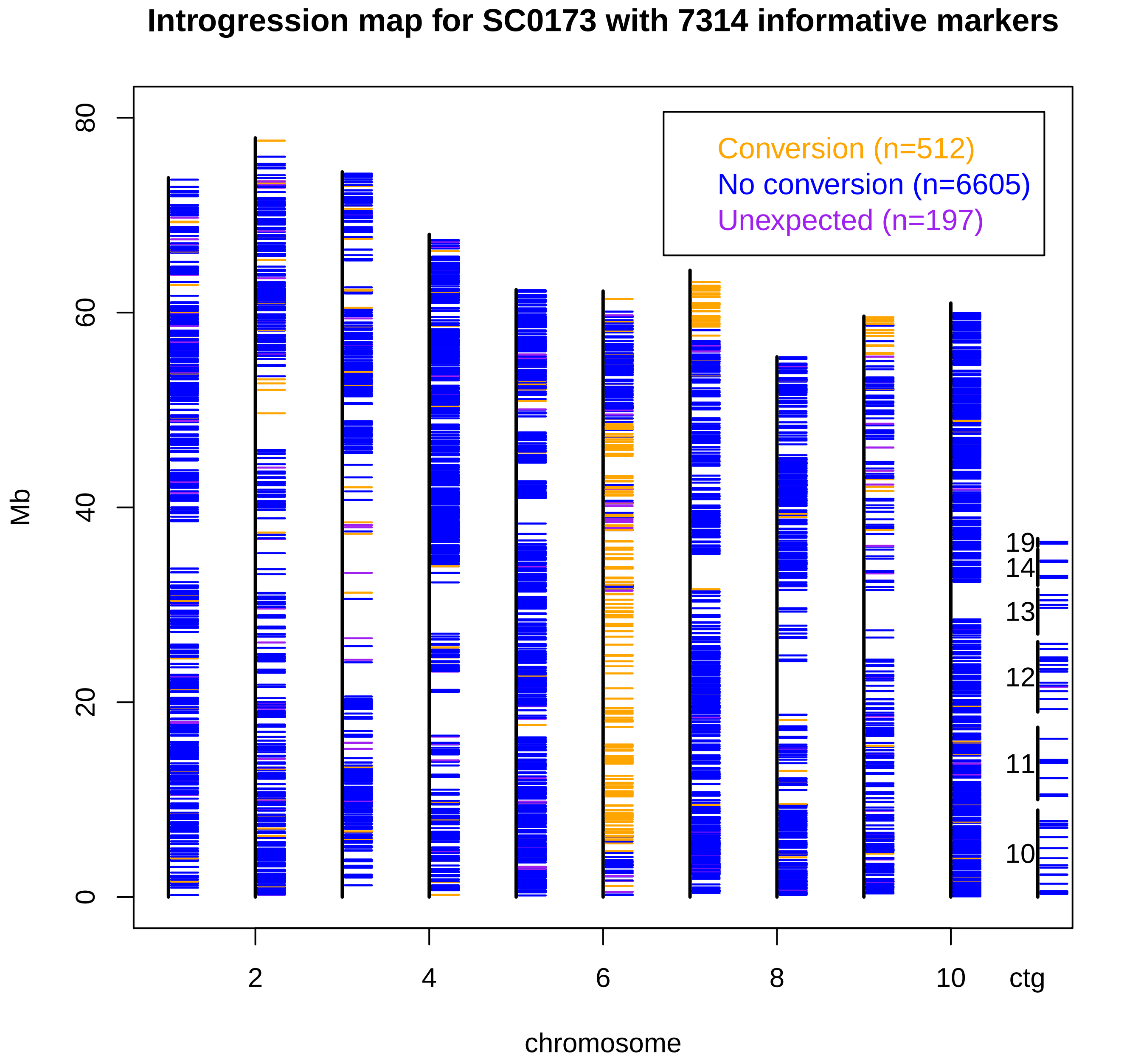

Introgression map for SC0173 with 7314 informative markers
Conversion (n=512)
No conversion (n=6605)
Unexpected (n=197)
Mb
0
20
40
60
80
19
14
13
12
11
10
2
4
6
8
10
ctg
chromosome

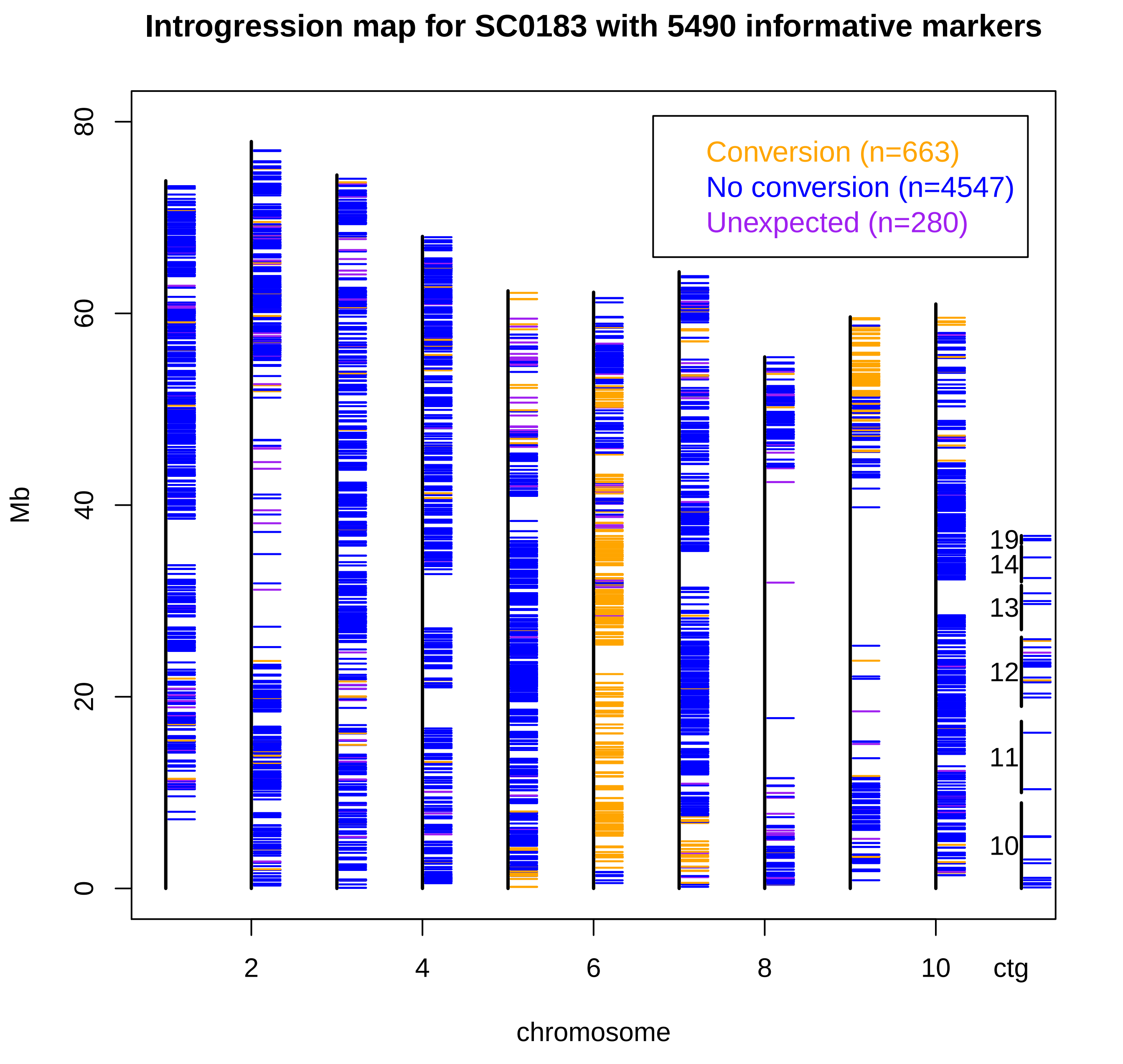
Introgression map for SC0183 with 5490 informative markers
Conversion (n=663)
No conversion (n=4547)
Unexpected (n=280)
Mb
0
20
40
60
80
chromosome
2
4
6
8
10
ctg
19
14
13
12
11
10

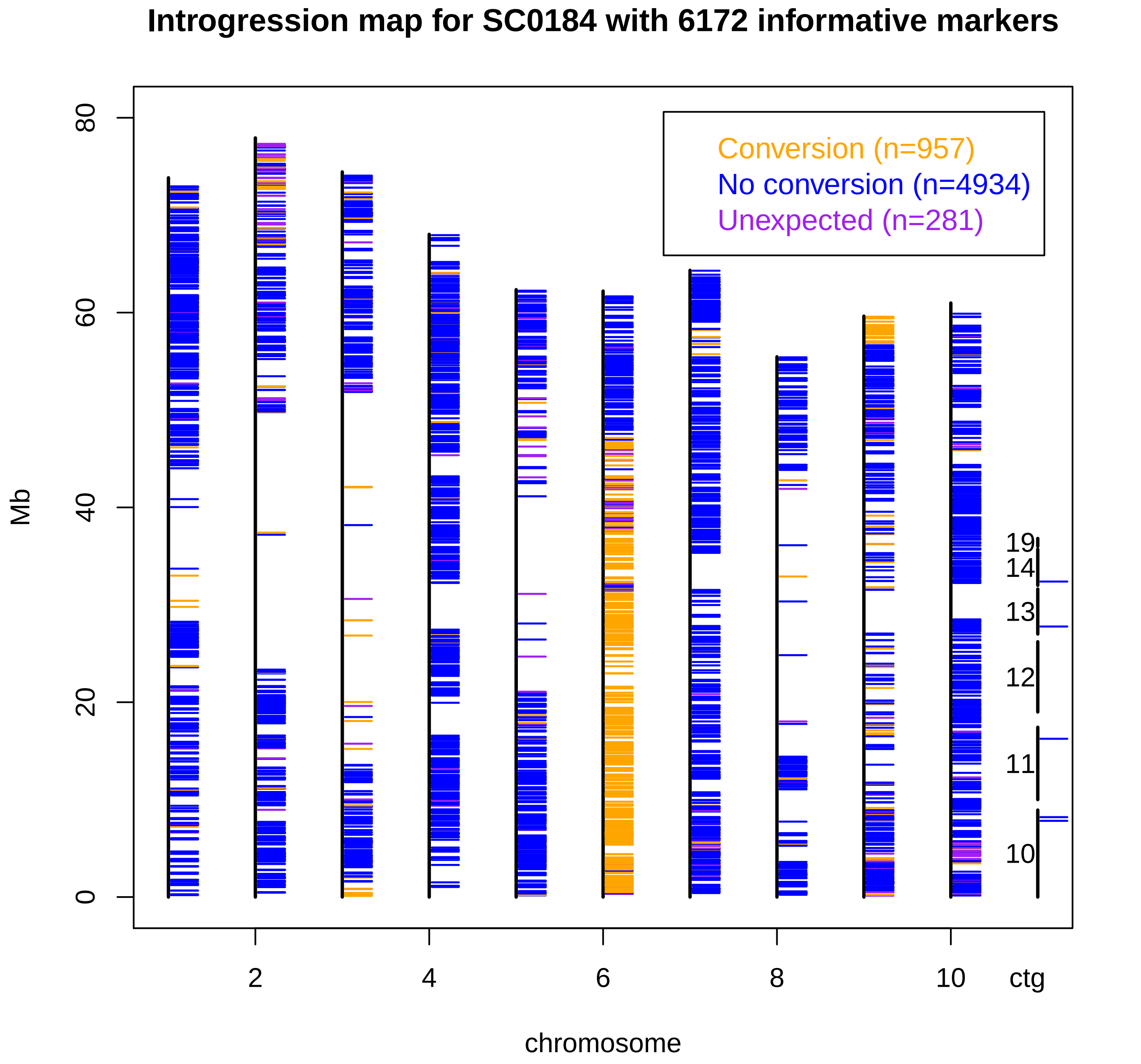

Introgression map for SC0184 with 6172 informative markers
Conversion (n=957)
No conversion (n=4934)
Unexpected (n=281)
Mb
0
20
40
60
80
chromosome
2
4
6
8
10
ctg
19
14
13
12
11
10

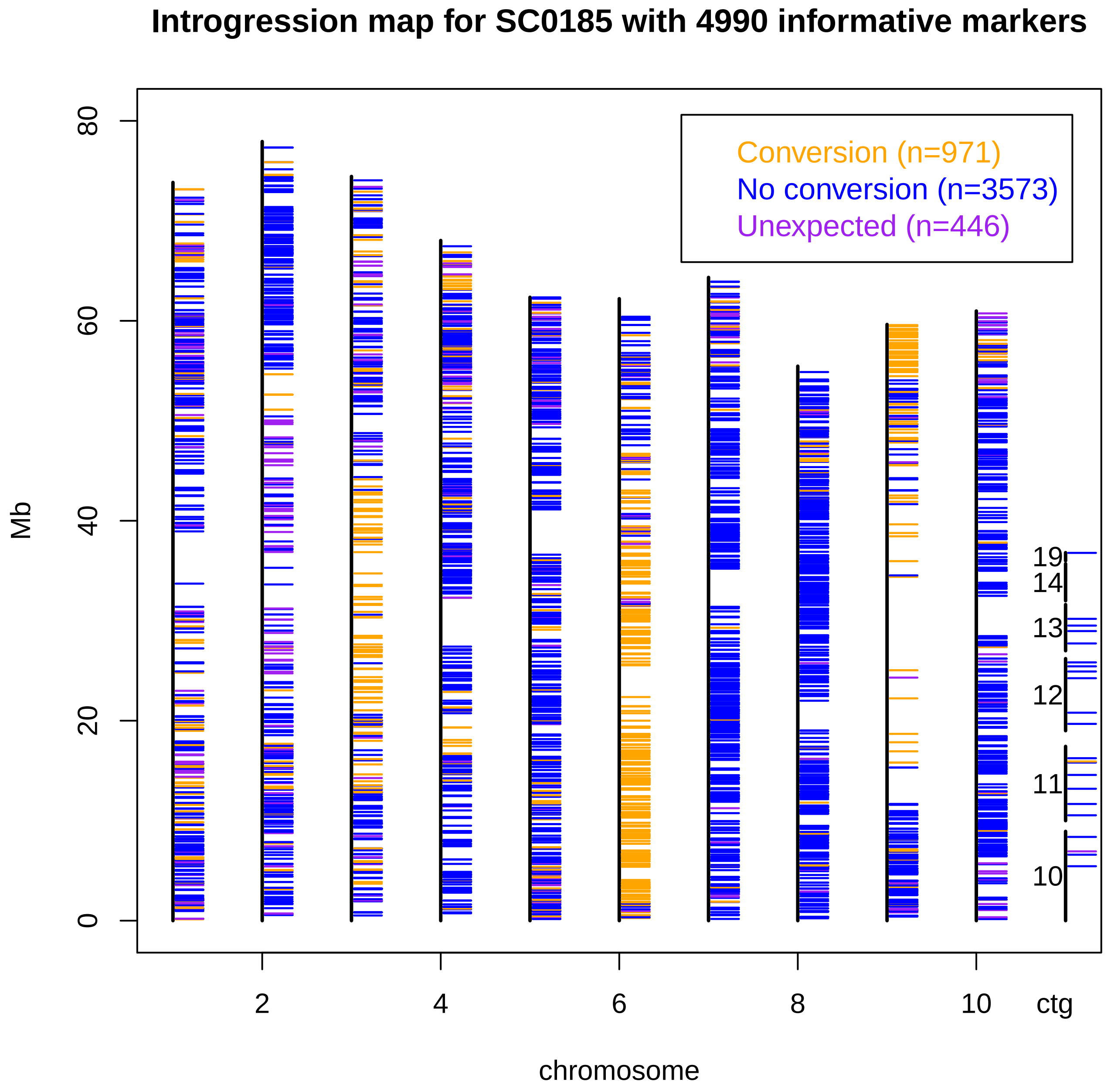

Introgression map for SC0185 with 4990 informative markers
Conversion (n=971)
No conversion (n=3573)
Unexpected (n=446)
Mb
0
20
40
60
80
2
4
6
8
10
ctg
19
14
13
12
11
10
chromosome

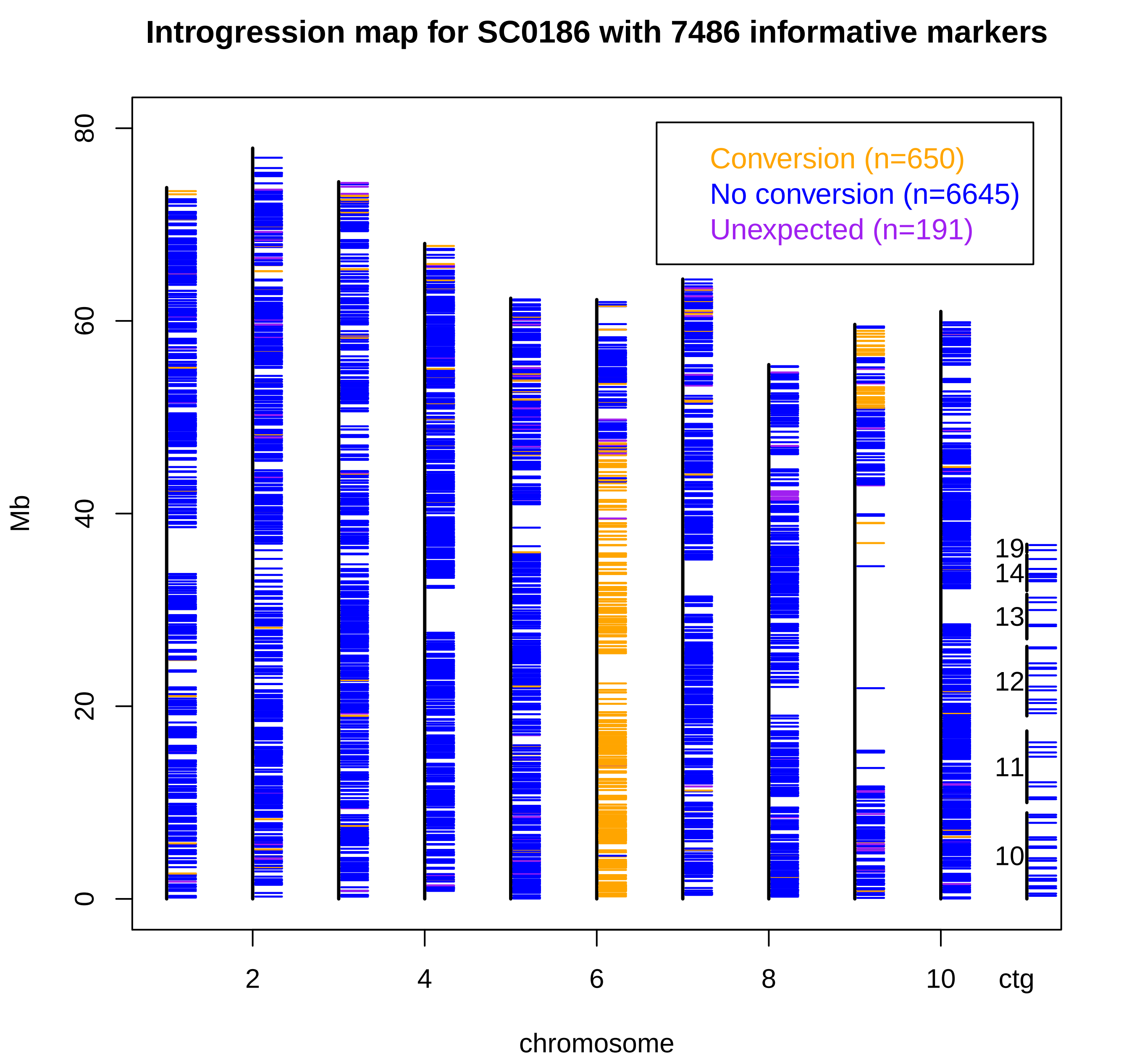
Introgression map for SC0186 with 7486 informative markers
Conversion (n=650)
No conversion (n=6645)
Unexpected (n=191)
Mb
0
20
40
60
80
chromosome
2
4
6
8
10
ctg
19
14
13
12
11
10

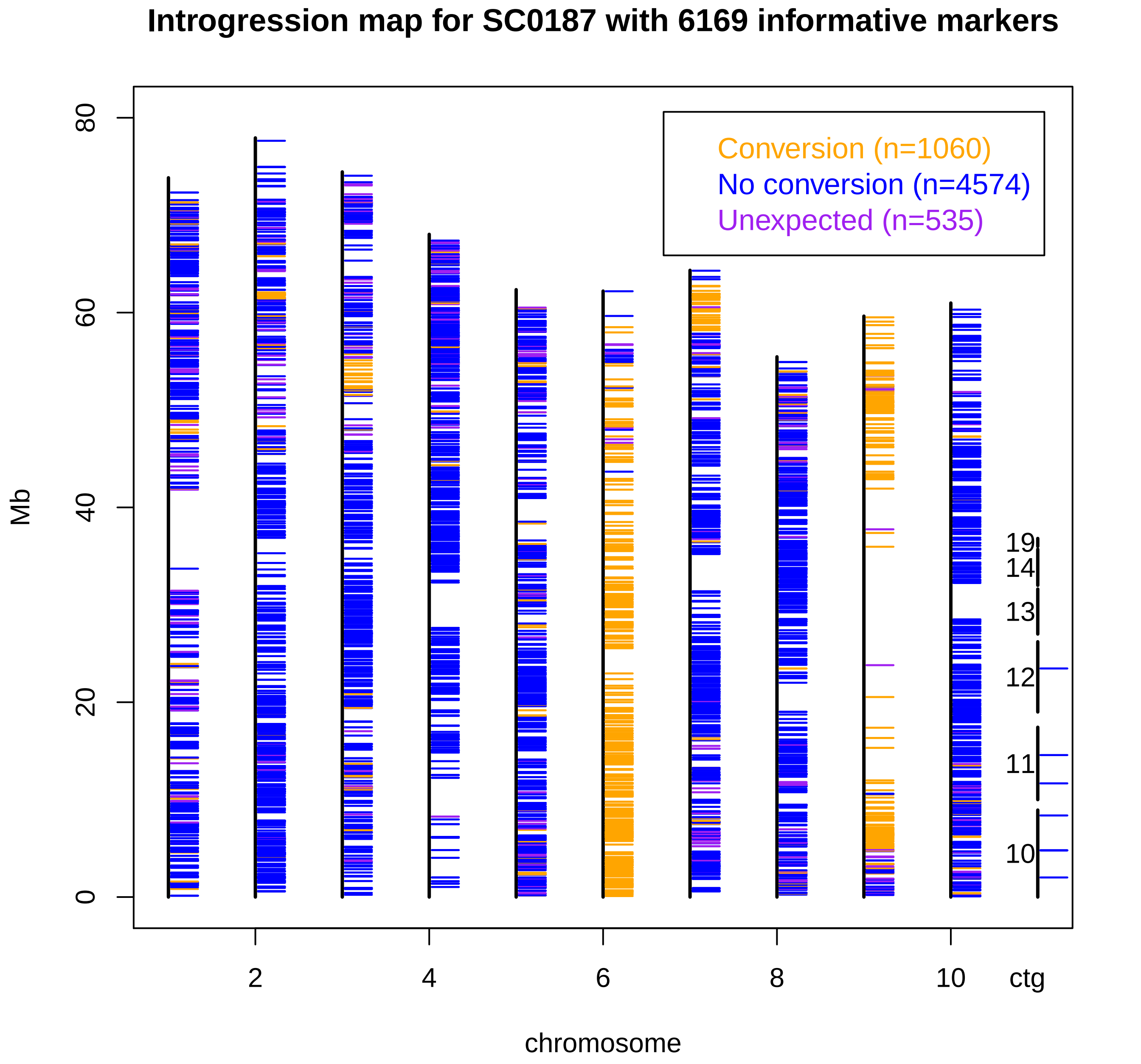

Introgression map for SC0187 with 6169 informative markers
Conversion (n=1060)
No conversion (n=4574)
Unexpected (n=535)
Mb
0
20
40
60
80
chromosome
2
4
6
8
10
ctg
19
14
13
12
11
10

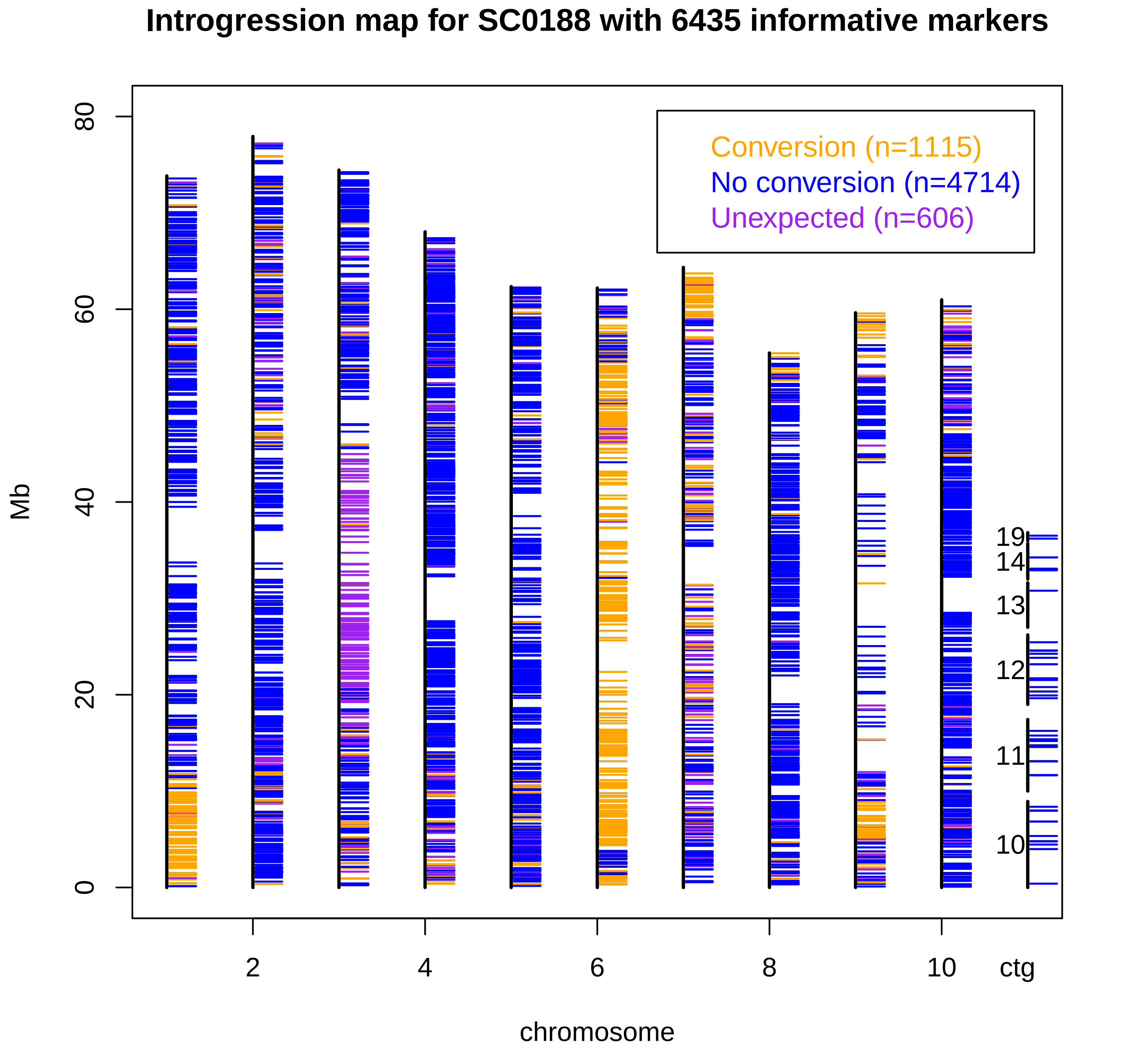
Introgression map for SC0188 with 6435 informative markers
Conversion (n=1115)
No conversion (n=4714)
Unexpected (n=606)
Mb
0
20
40
60
80
chromosome
2
4
6
8
10
ctg
19
14
13
12
11
10

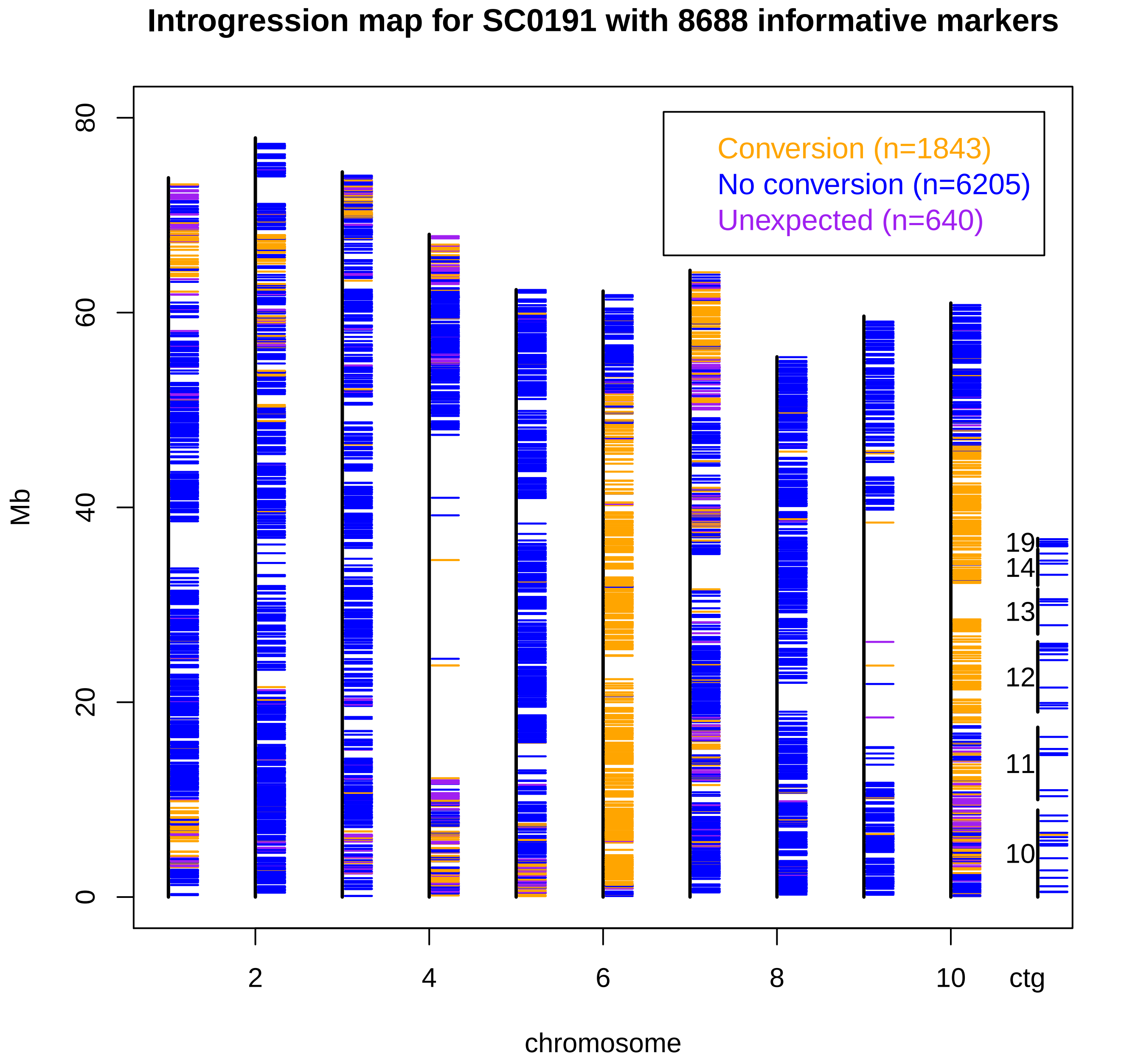

Introgression map for SC0191 with 8688 informative markers
Conversion (n=1843)
No conversion (n=6205)
Unexpected (n=640)
Mb
0
20
40
60
80
chromosome
2
4
6
8
10
ctg
19
14
13
12
11
10

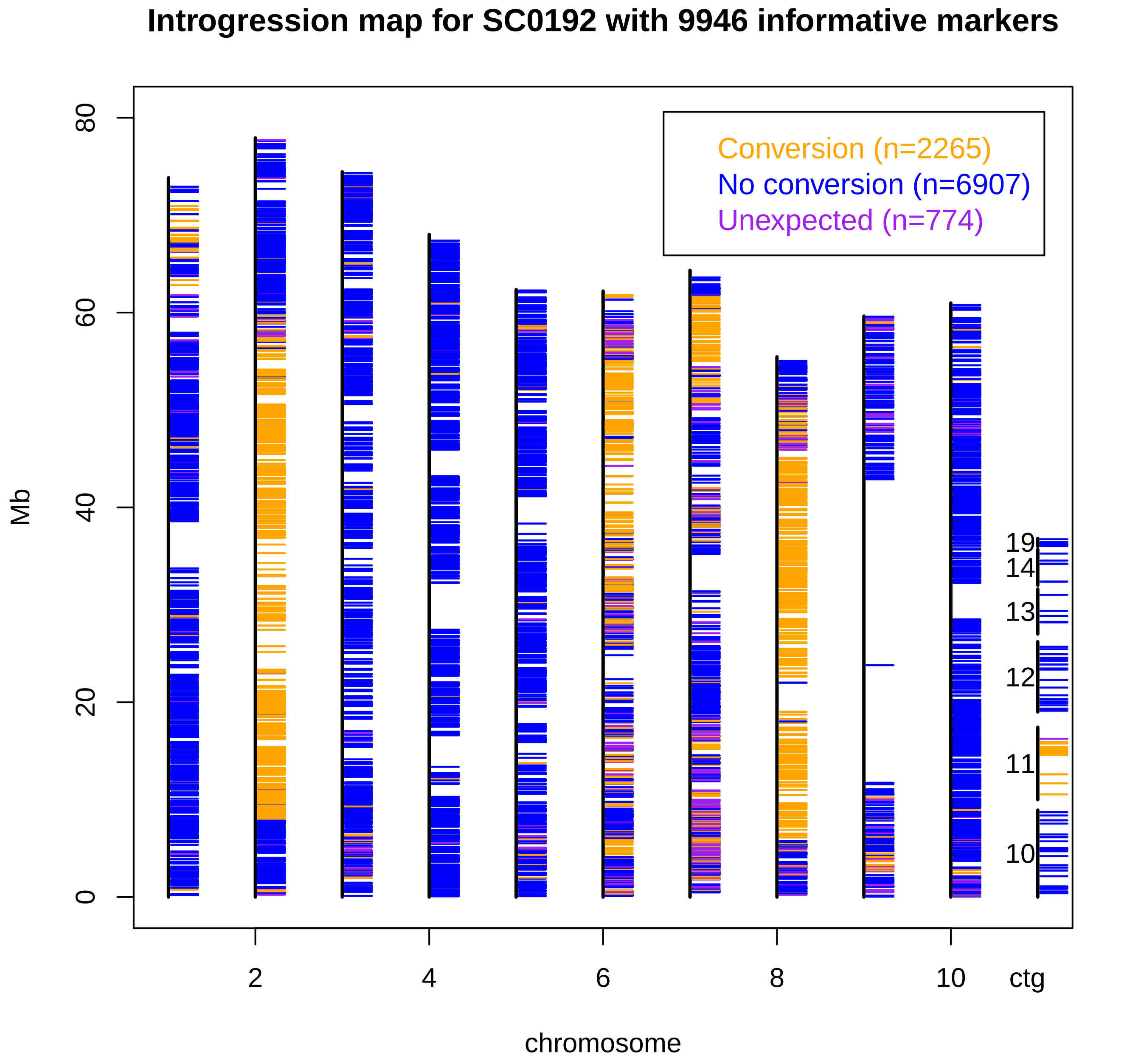

Introgression map for SC0192 with 9946 informative markers
Conversion (n=2265)
No conversion (n=6907)
Unexpected (n=774)
Mb
0
20
40
60
80
2
4
6
8
10
ctg
chromosome
19
14
13
12
11
10

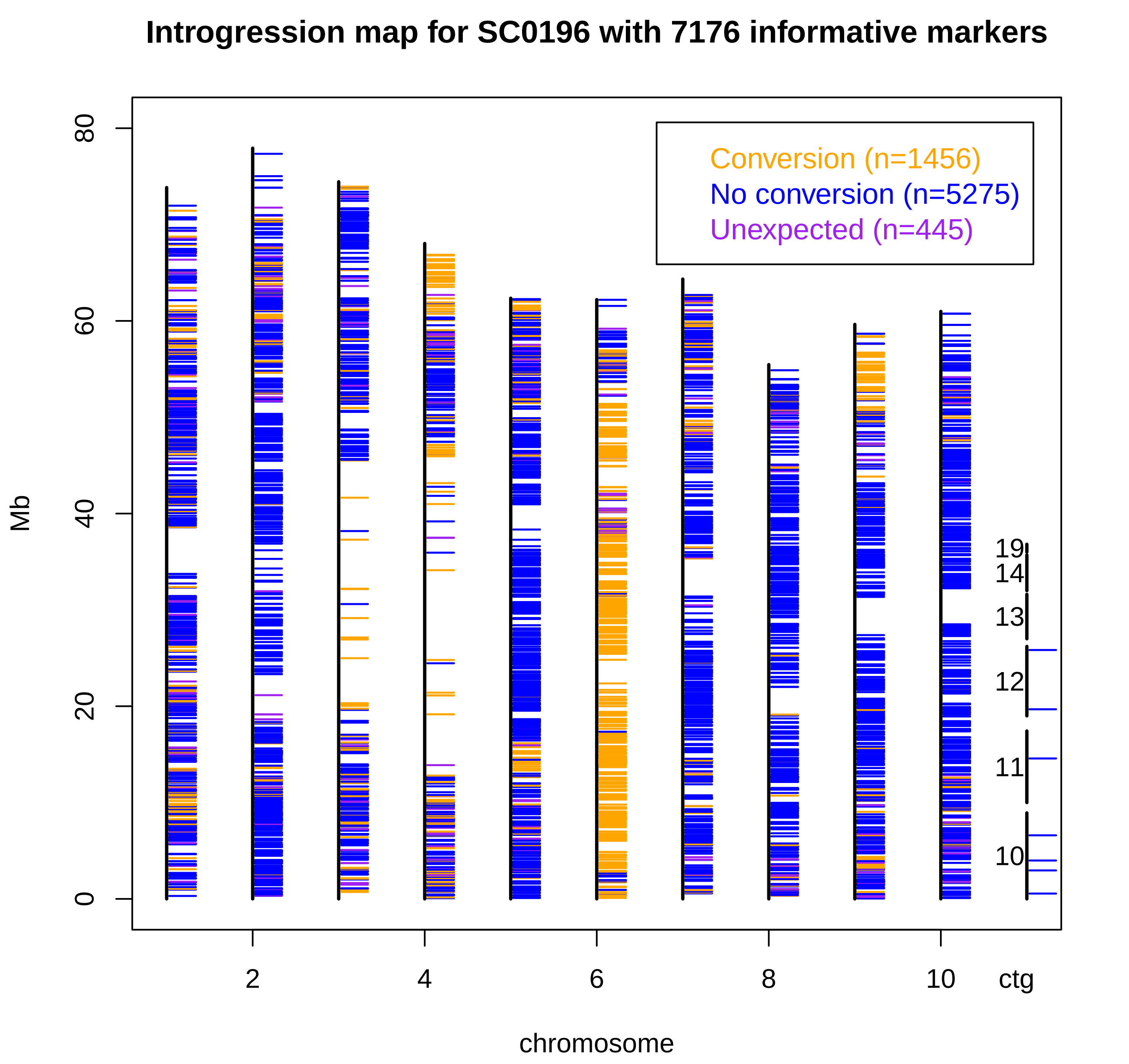
Introgression map for SC0196 with 7176 informative markers
Conversion (n=1456)
No conversion (n=5275)
Unexpected (n=445)
Mb
0
20
40
60
80
chromosome
2
4
6
8
10
ctg
19
14
13
12
11
10

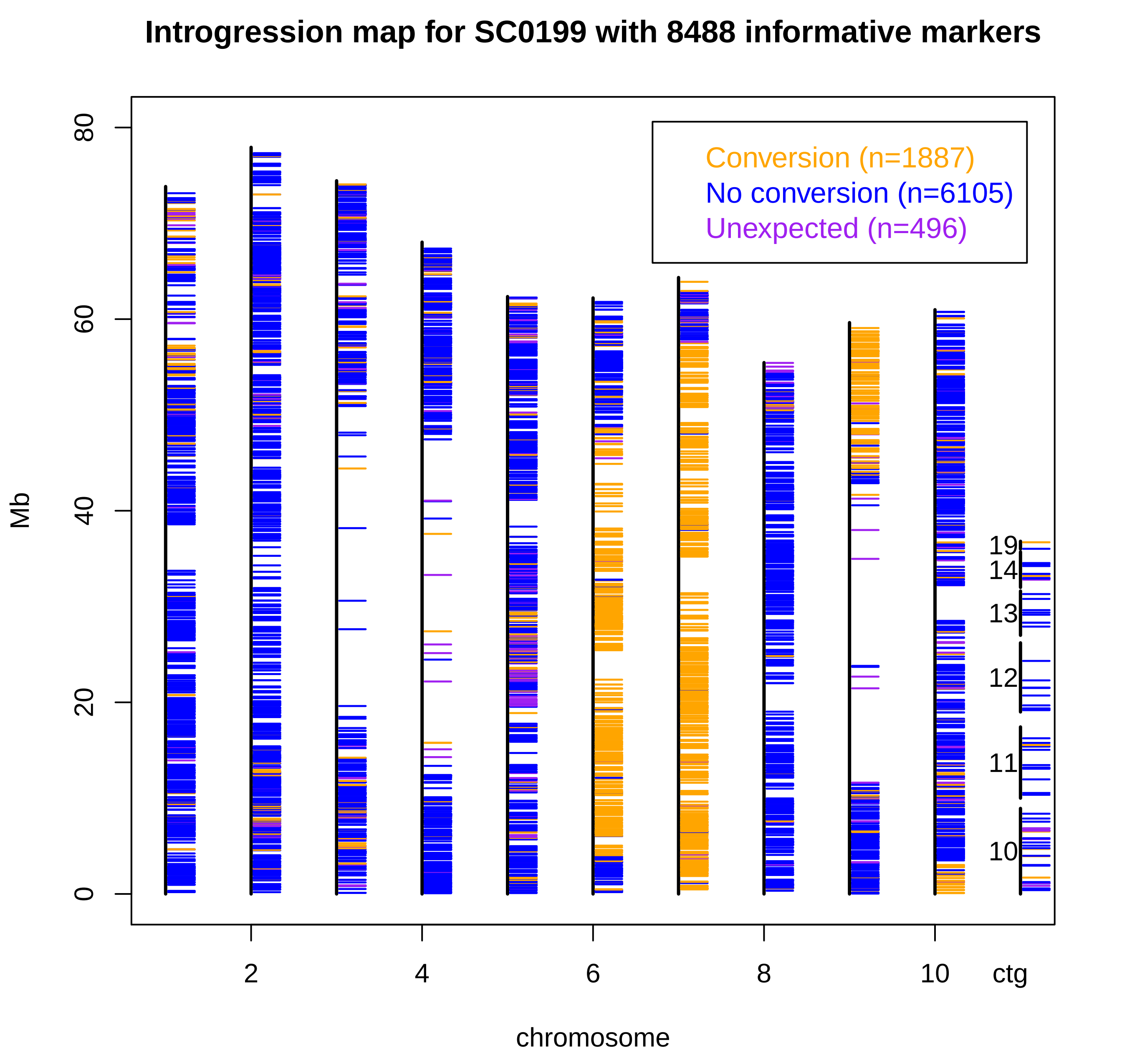

Introgression map for SC0199 with 8488 informative markers
Conversion (n=1887)
No conversion (n=6105)
Unexpected (n=496)
Mb
0
20
40
60
80
chromosome
2
4
6
8
10
ctg
19
14
13
12
11
10

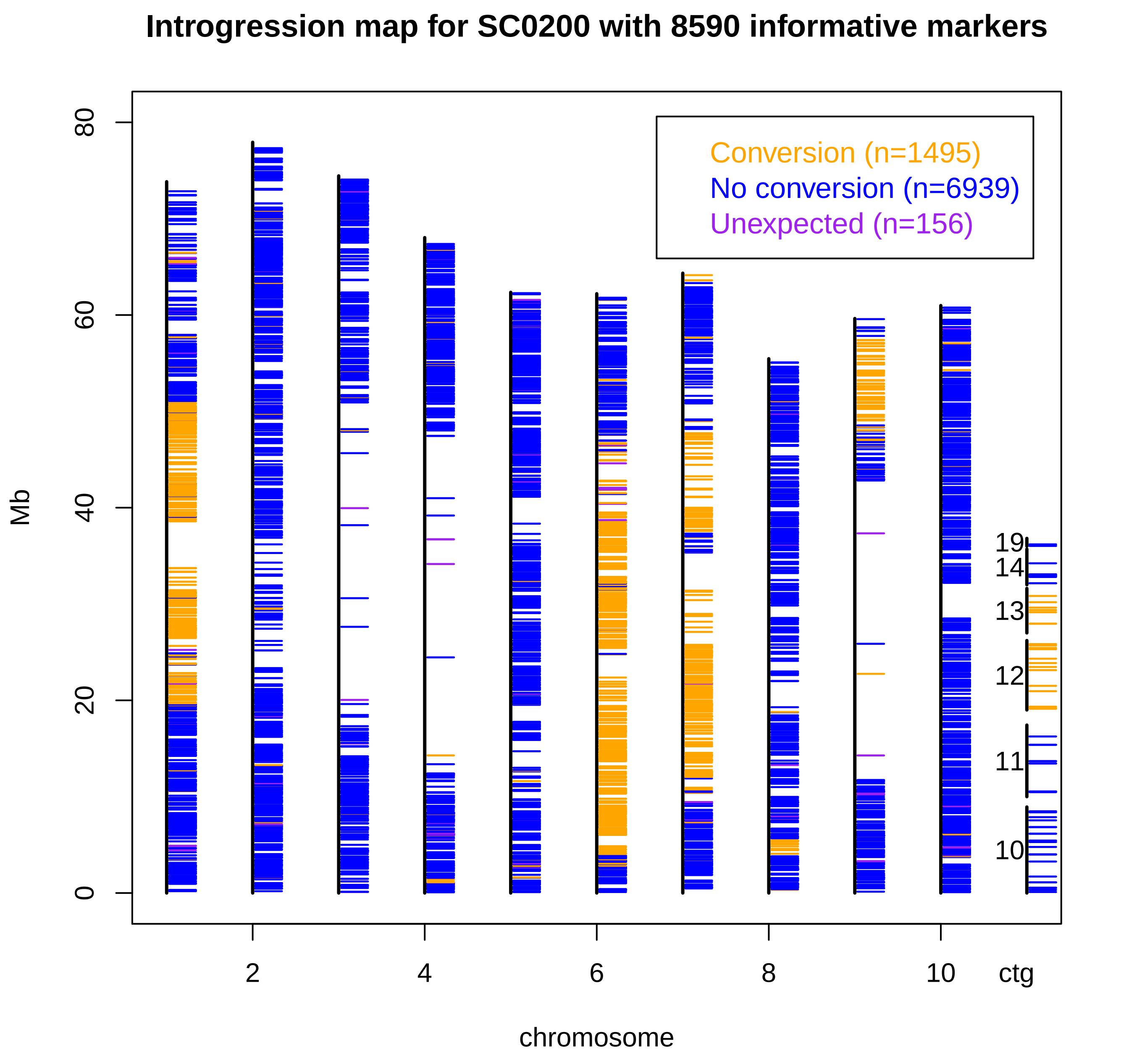

Introgression map for SC0200 with 8590 informative markers
Conversion (n=1495)
No conversion (n=6939)
Unexpected (n=156)
Mb
0
20
40
60
80
chromosome
2
4
6
8
10
ctg
19
14
13
12
11
10

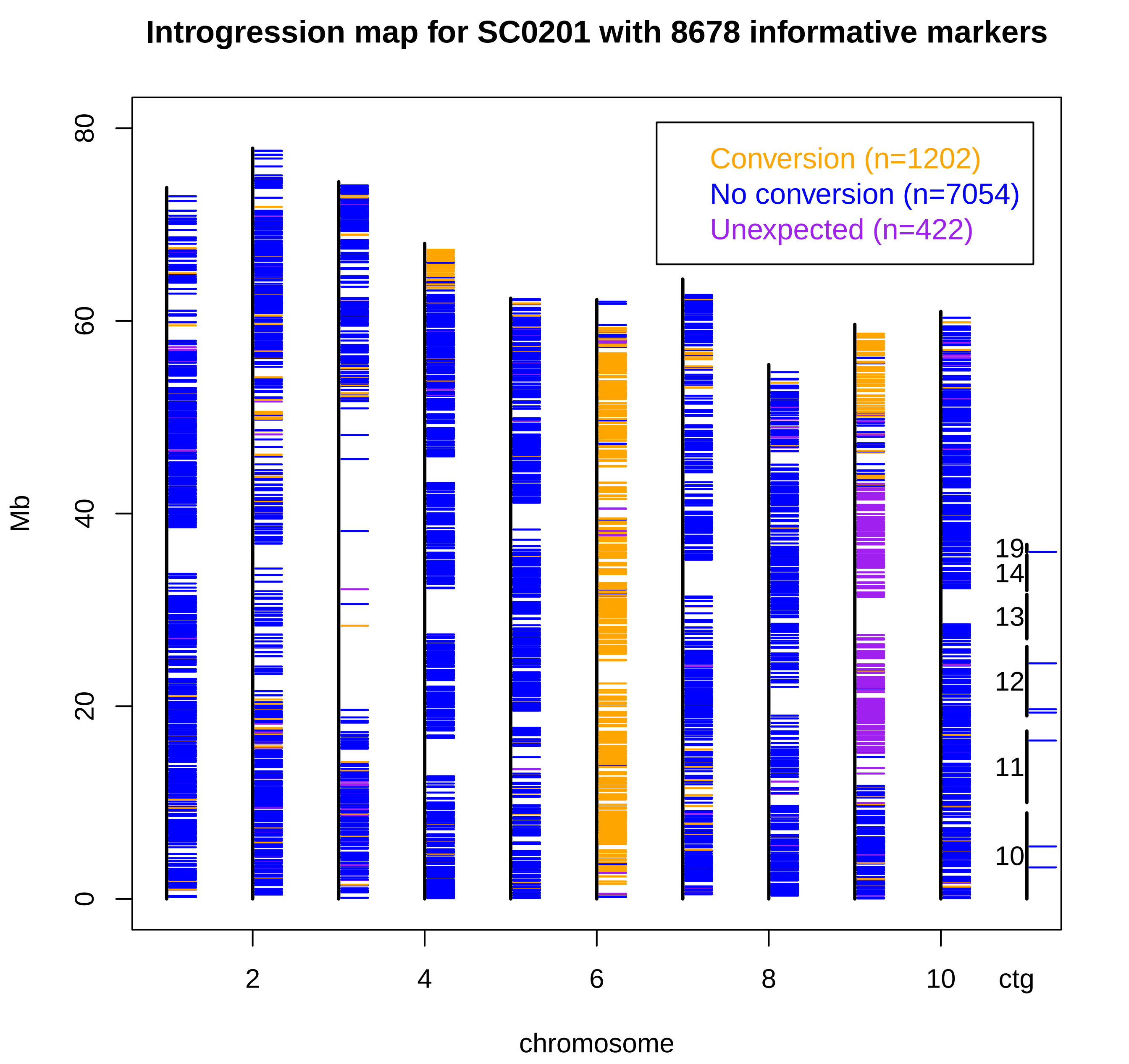
Introgression map for SC0201 with 8678 informative markers
Conversion (n=1202)
No conversion (n=7054)
Unexpected (n=422)
Mb
0
20
40
60
80
chromosome
2
4
6
8
10
ctg
19
14
13
12
11
10

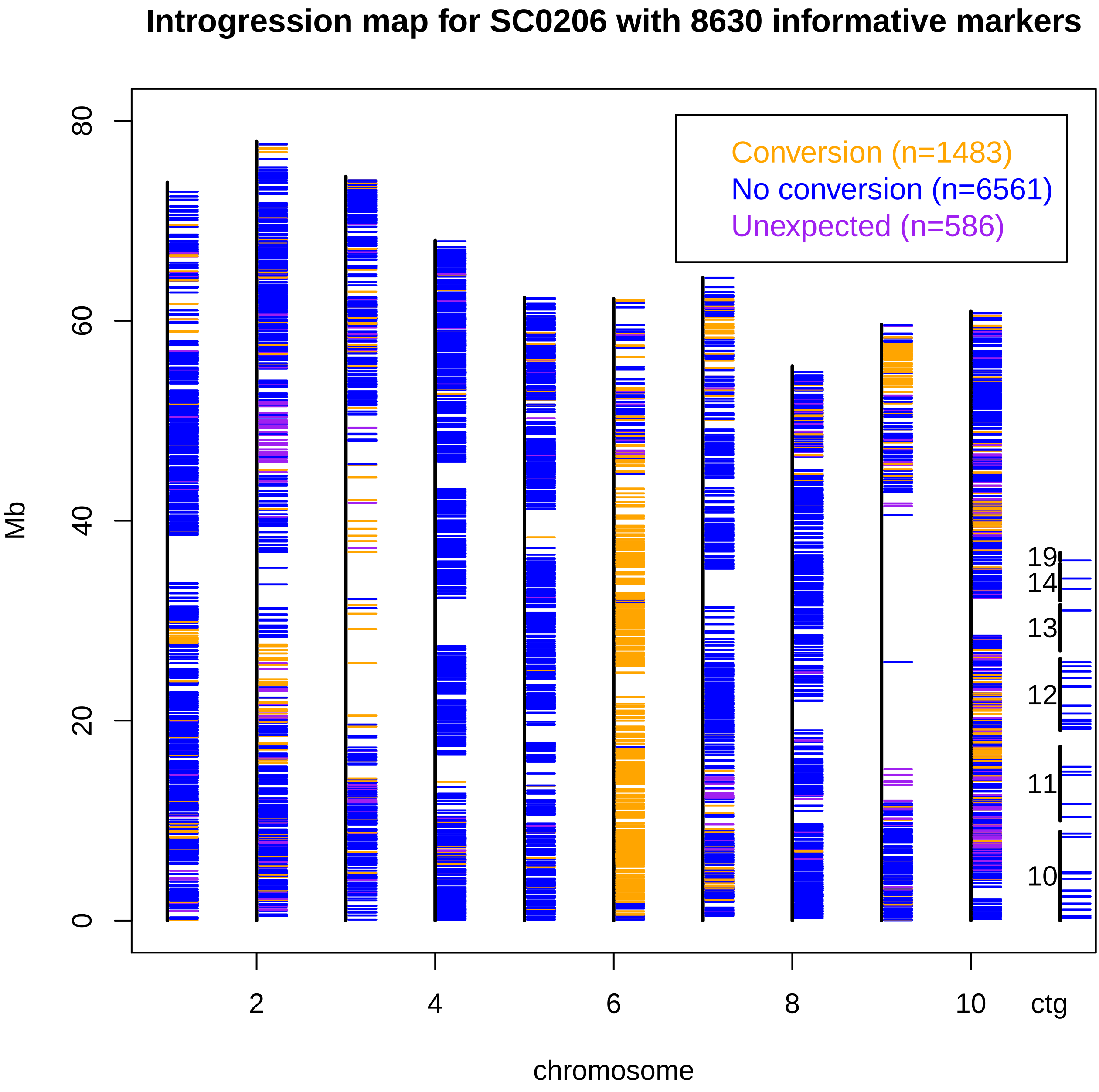

Introgression map for SC0206 with 8630 informative markers
Conversion (n=1483)
No conversion (n=6561)
Unexpected (n=586)
Mb
0
20
40
60
80
chromosome
2
4
6
8
10
ctg
19
14
13
12
11
10

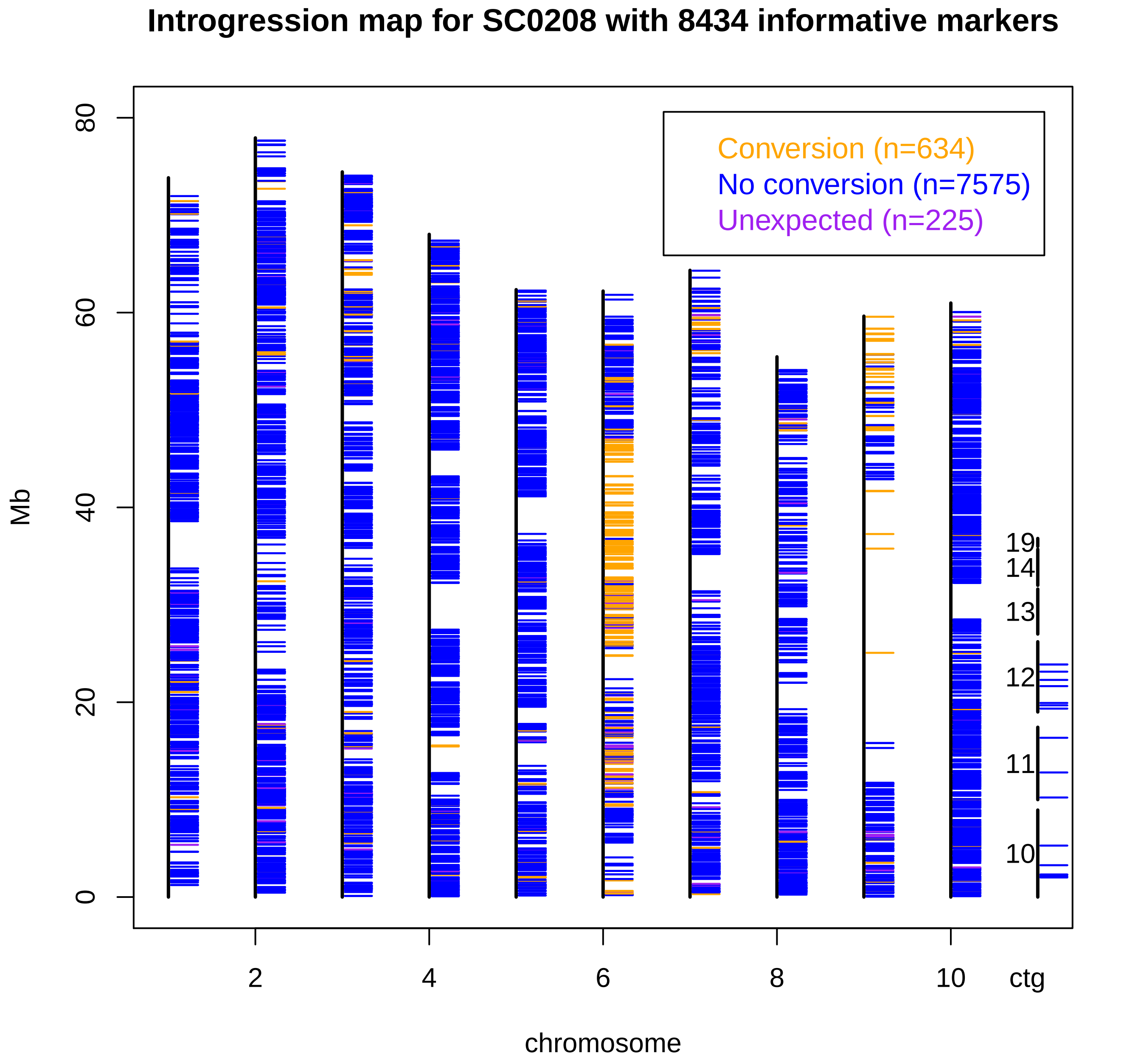
Introgression map for SC0208 with 8434 informative markers
Conversion (n=634)
No conversion (n=7575)
Unexpected (n=225)
Mb
0
20
40
60
80
chromosome
2
4
6
8
10
ctg
19
14
13
12
11
10

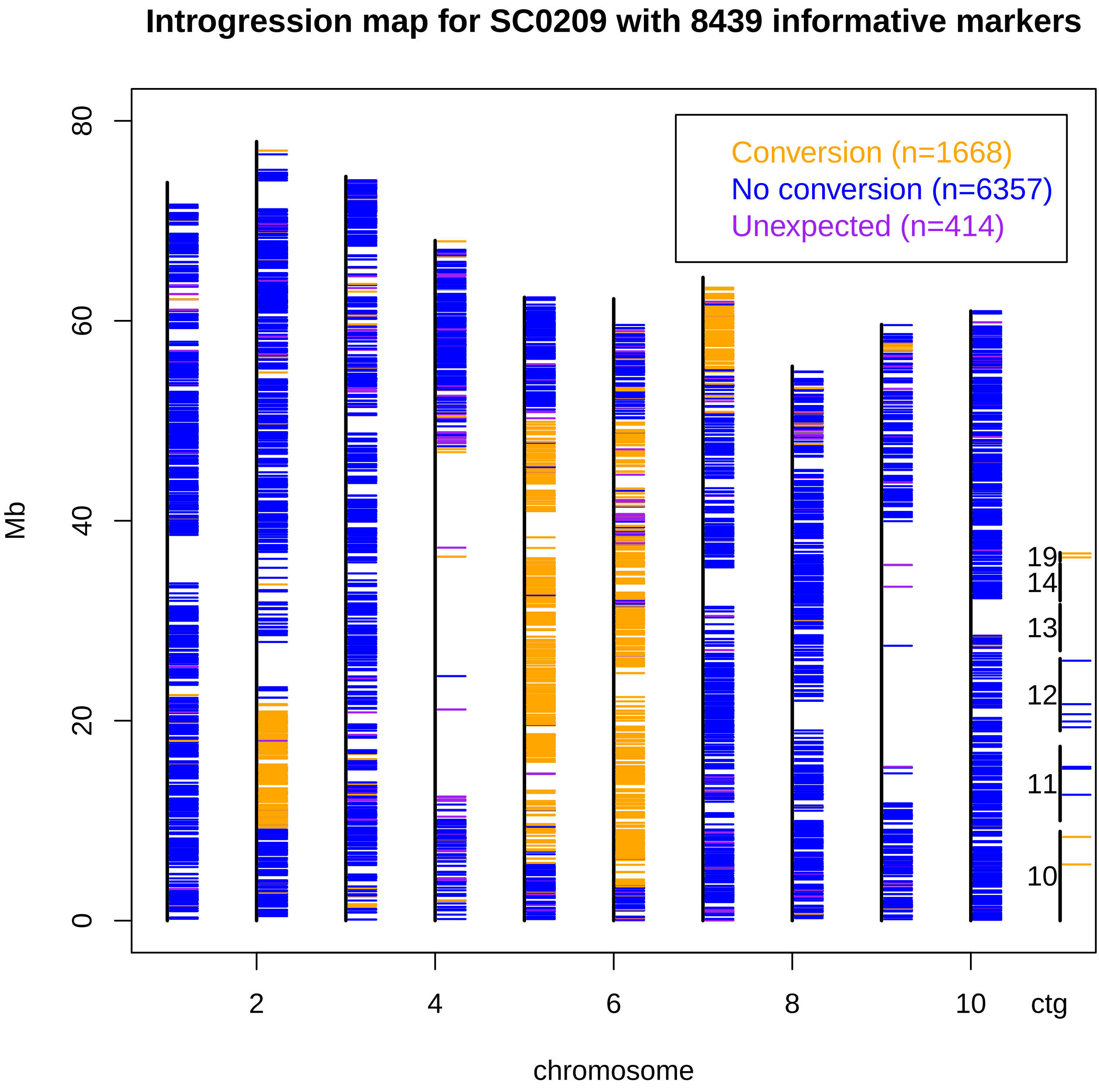

Introgression map for SC0209 with 8439 informative markers
Conversion (n=1668)
No conversion (n=6357)
Unexpected (n=414)
Mb
0
20
40
60
80
chromosome
2
4
6
8
10
ctg
19
14
13
12
11
10

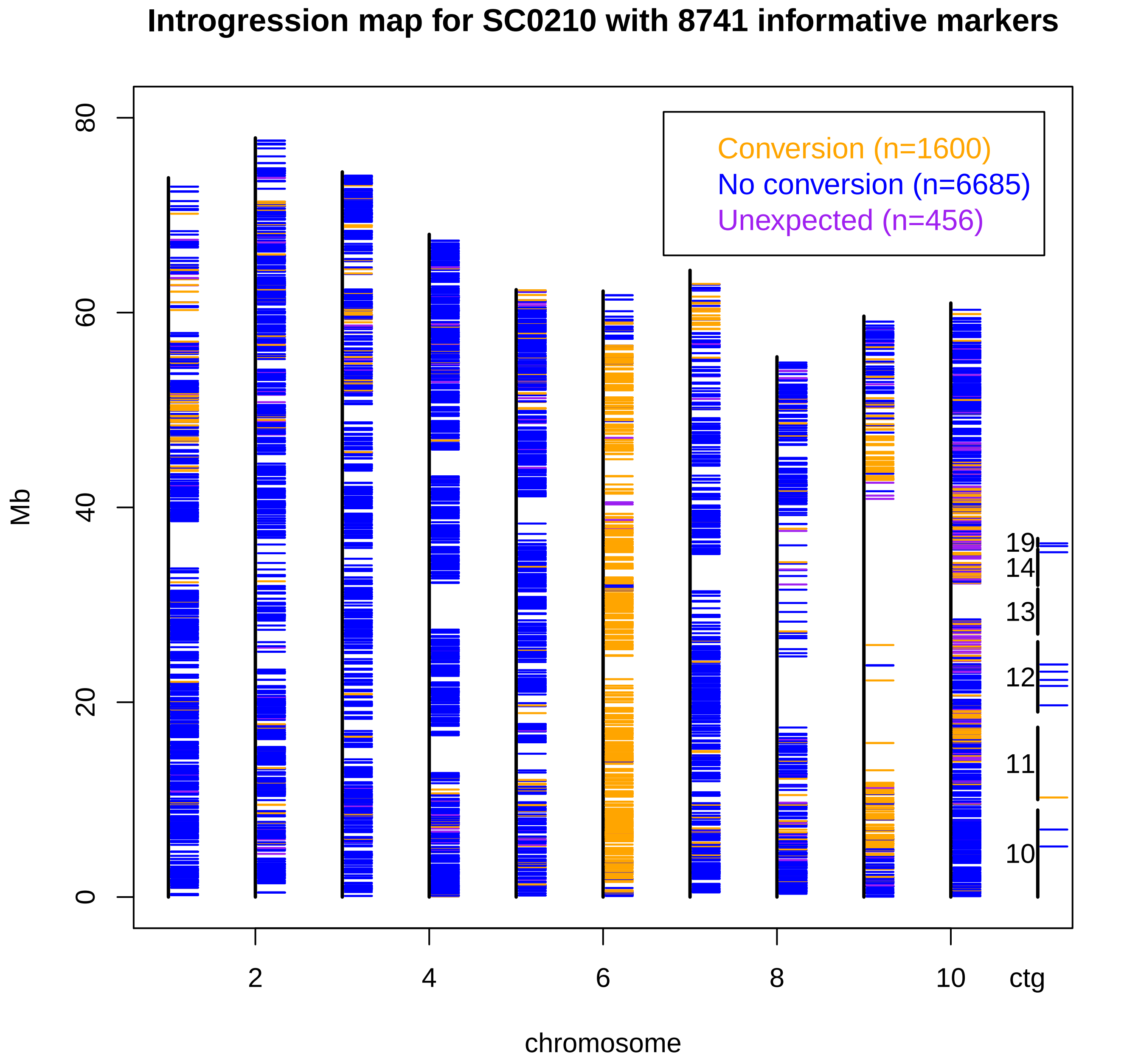

Introgression map for SC0210 with 8741 informative markers
Conversion (n=1600)
No conversion (n=6685)
Unexpected (n=456)
Mb
0
20
40
60
80
chromosome
2
4
6
8
10
ctg
19
14
13
12
11
10

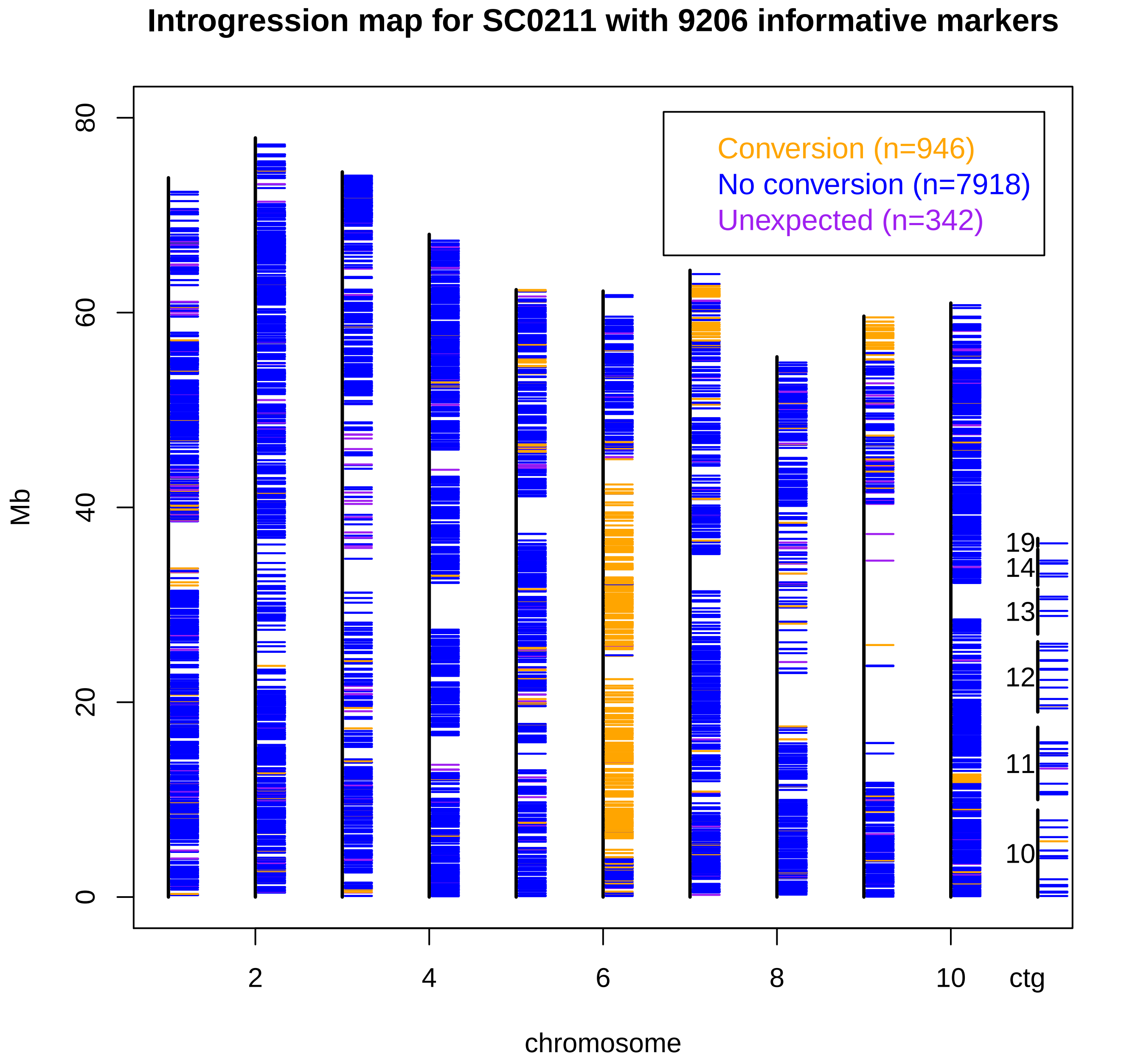
Introgression map for SC0211 with 9206 informative markers
Conversion (n=946)
No conversion (n=7918)
Unexpected (n=342)
Mb
0
20
40
60
80
2
4
6
8
10
ctg
19
14
13
12
11
10
chromosome

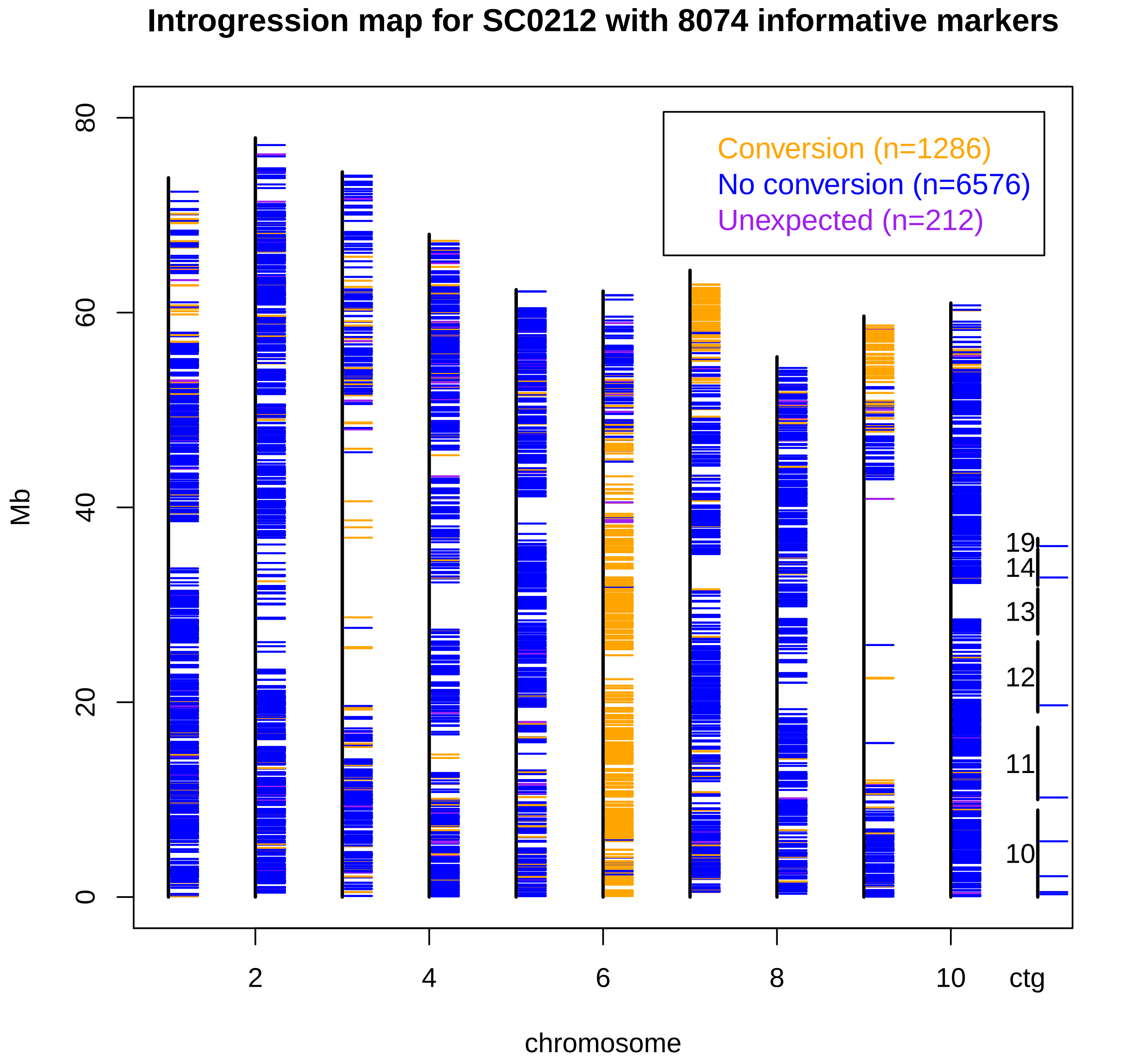

Introgression map for SC0212 with 8074 informative markers
Conversion (n=1286)
No conversion (n=6576)
Unexpected (n=212)
Mb
0
20
40
60
80
chromosome
2
4
6
8
10
ctg
19
14
13
12
11
10

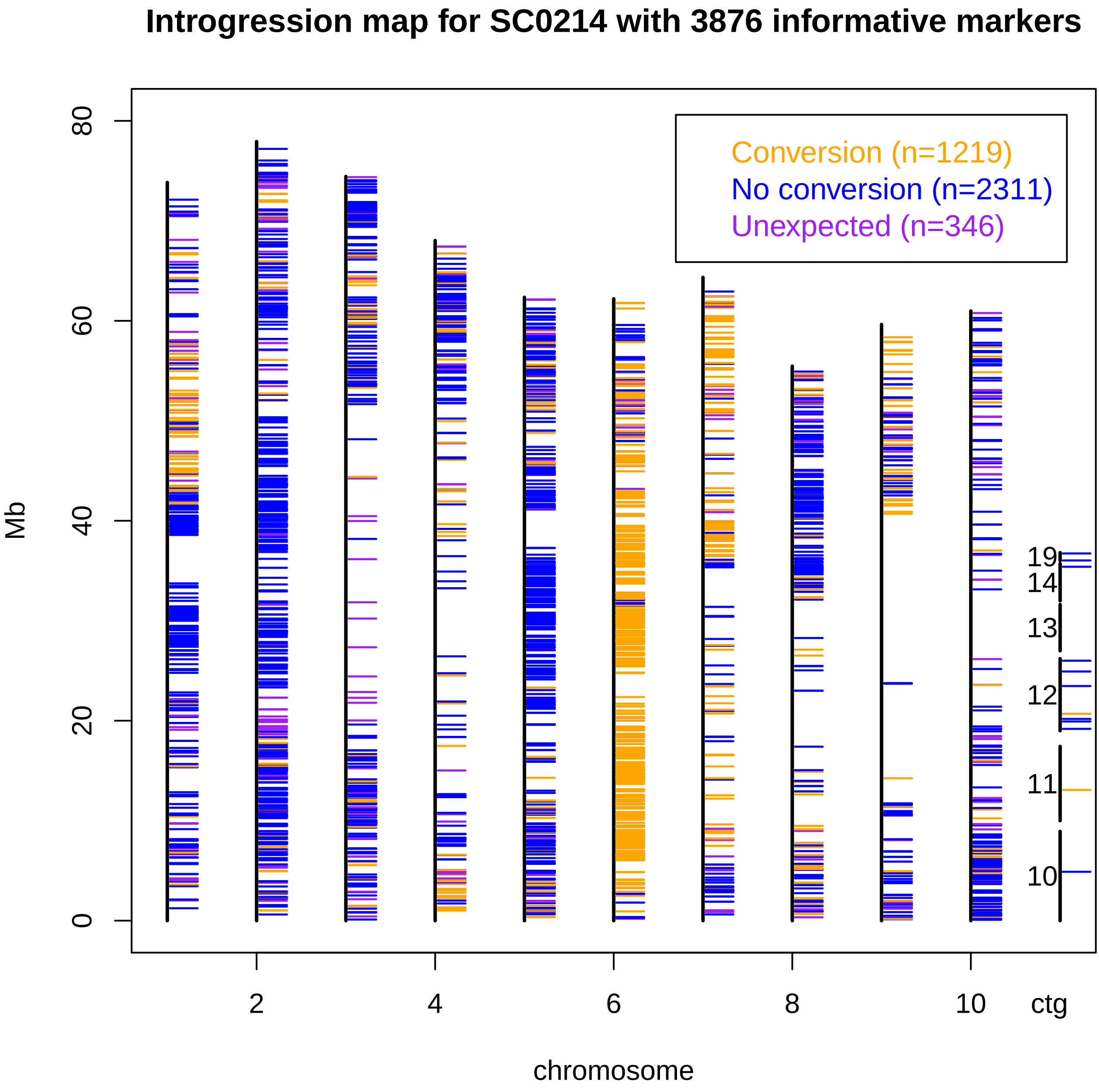

Introgression map for SC0214 with 3876 informative markers
Conversion (n=1219)
No conversion (n=2311)
Unexpected (n=346)
Mb
0
20
40
60
80
chromosome
2
4
6
8
10
ctg
19
14
13
12
11
10

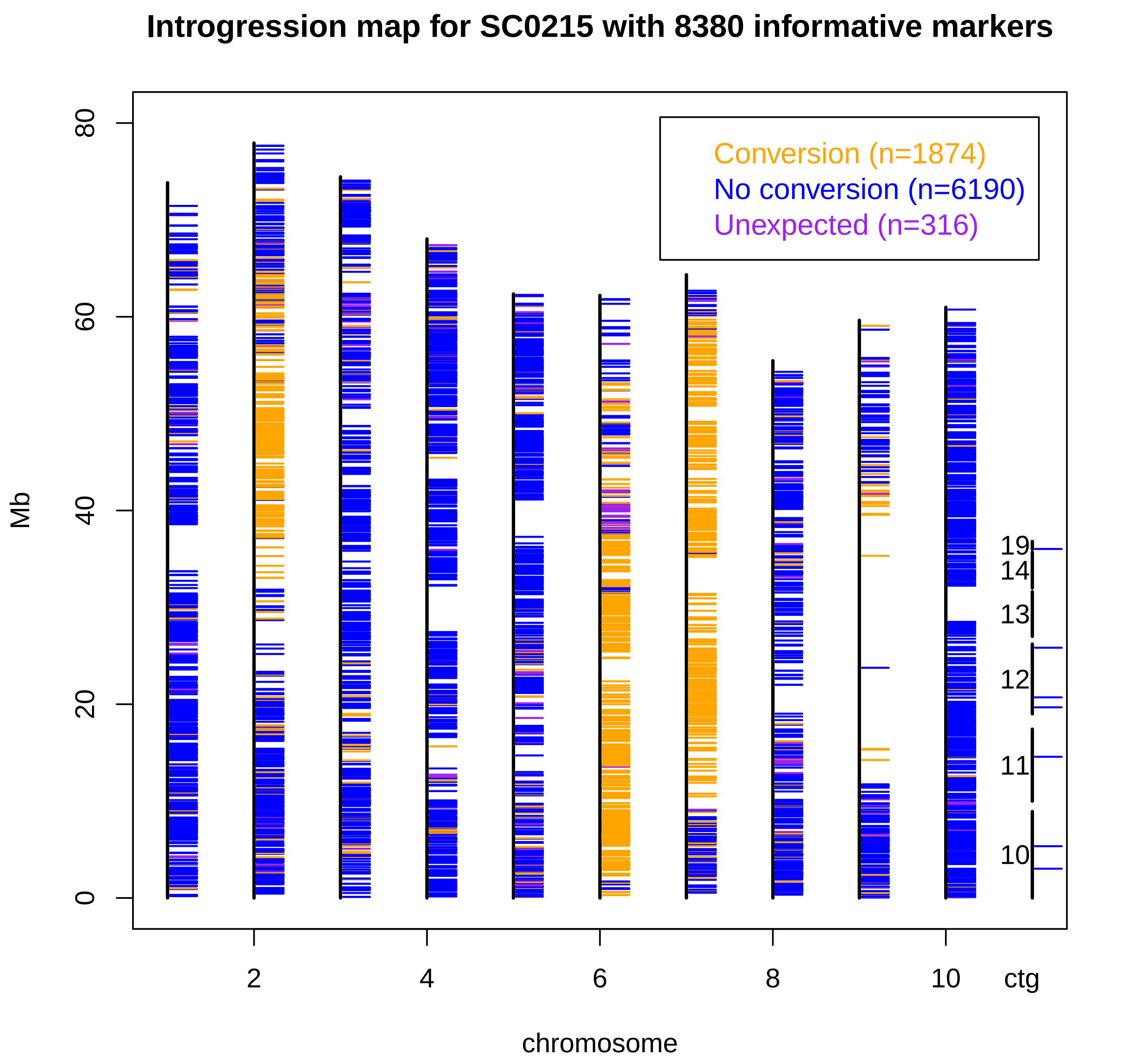

Introgression map for SC0215 with 8380 informative markers
Conversion (n=1874)
No conversion (n=6190)
Unexpected (n=316)
Mb
0
20
40
60
80
chromosome
2
4
6
8
10
ctg
19
14
13
12
11
10

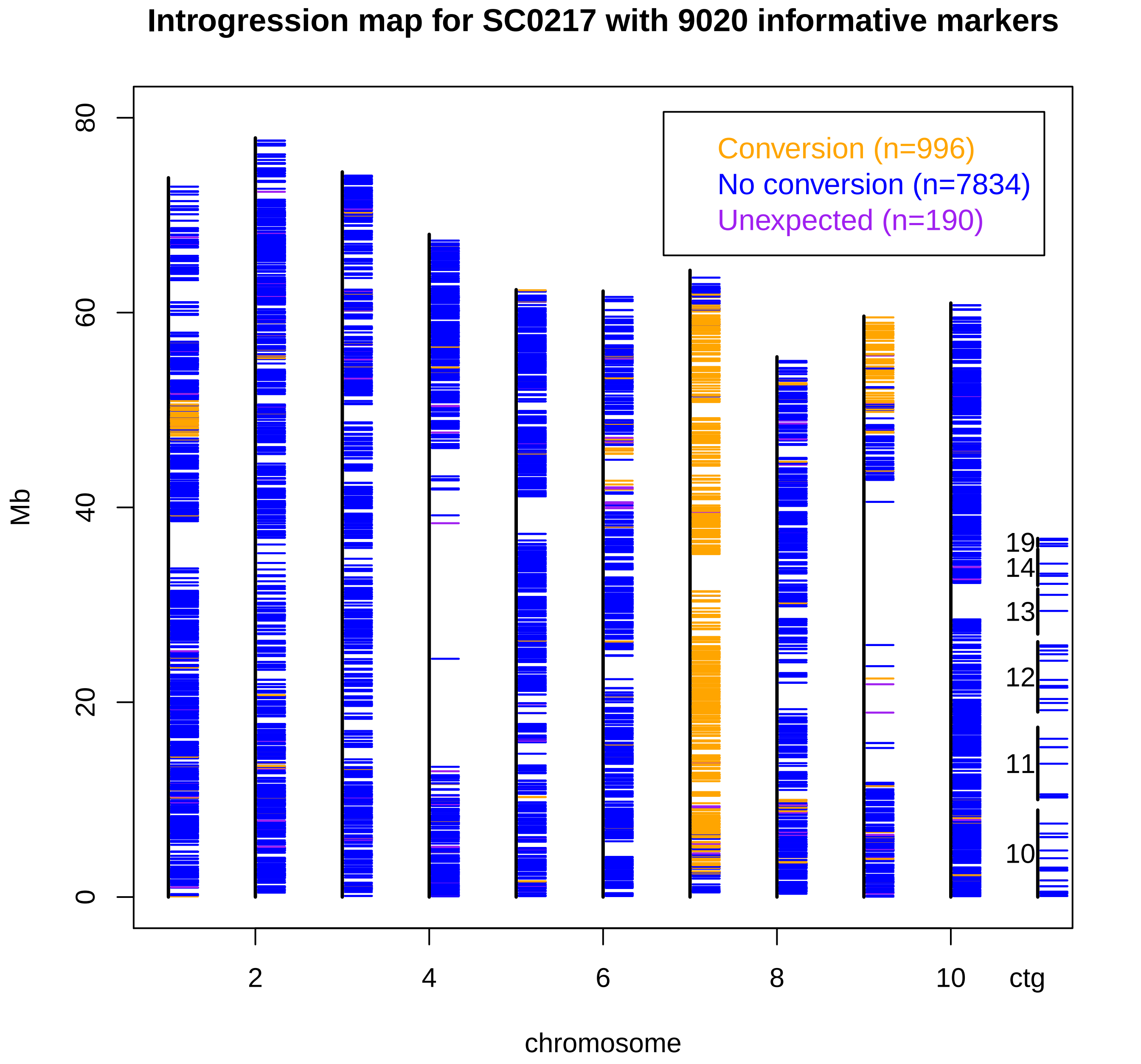
Introgression map for SC0217 with 9020 informative markers
Conversion (n=996)
No conversion (n=7834)
Unexpected (n=190)
Mb
0
20
40
60
80
chromosome
2
4
6
8
10
ctg
19
14
13
12
11
10

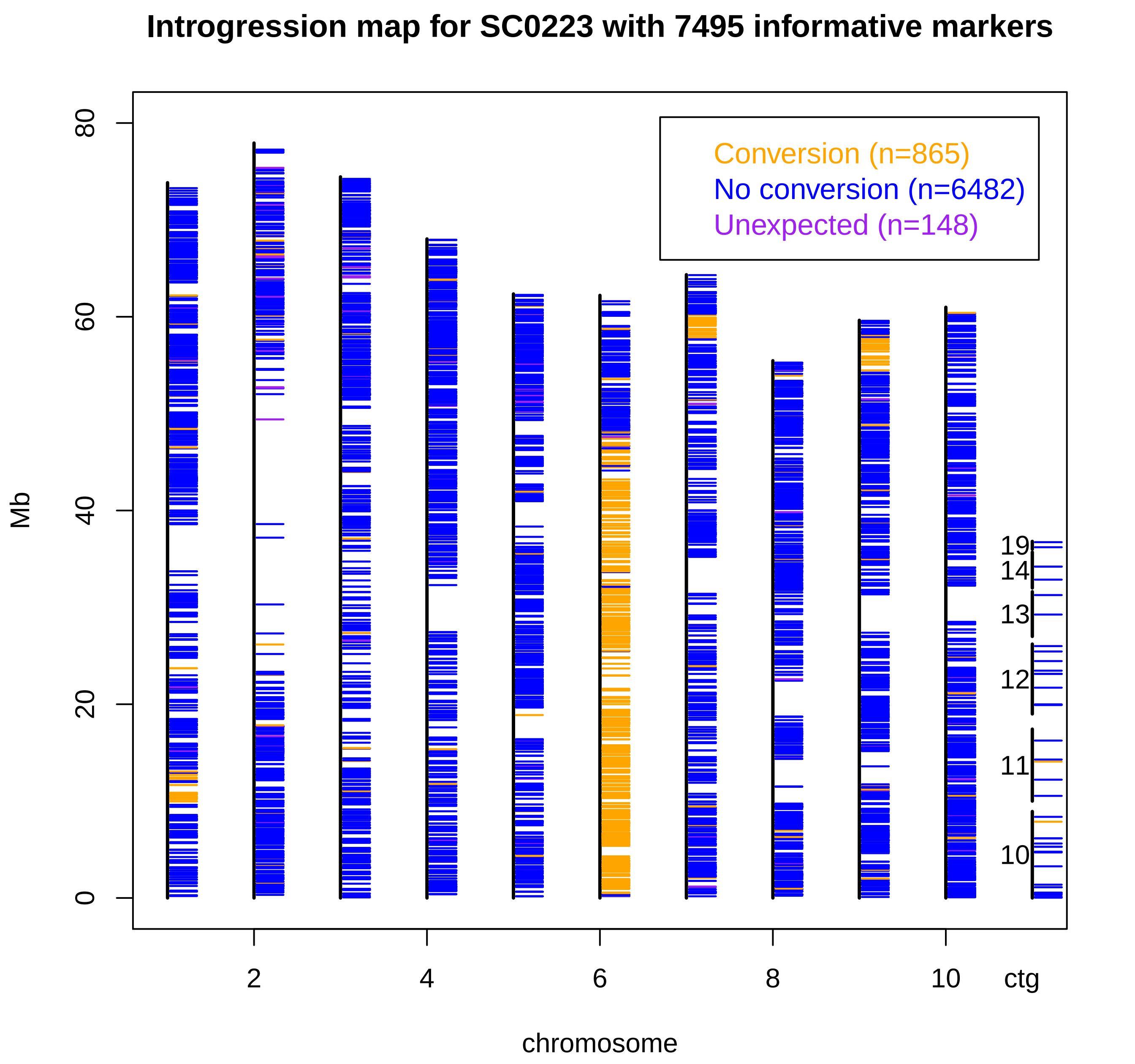

Introgression map for SC0223 with 7495 informative markers
Conversion (n=865)
No conversion (n=6482)
Unexpected (n=148)
Mb
0
20
40
60
80
chromosome
2
4
6
8
10
ctg
19
14
13
12
11
10

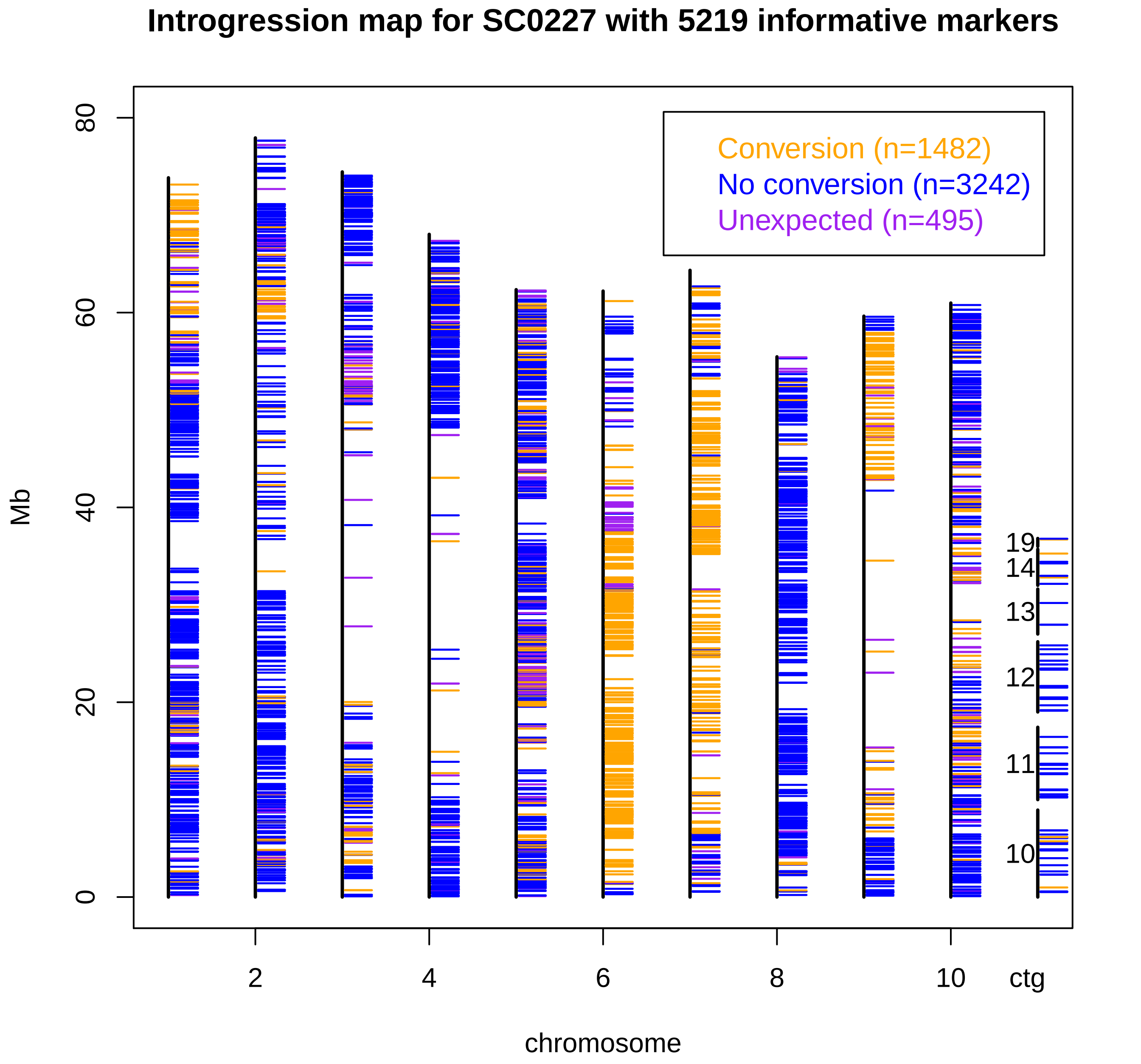
Introgression map for SC0227 with 5219 informative markers
Conversion (n=1482)
No conversion (n=3242)
Unexpected (n=495)
Mb
0
20
40
60
80
chromosome
2
4
6
8
10
ctg
19
14
13
12
11
10

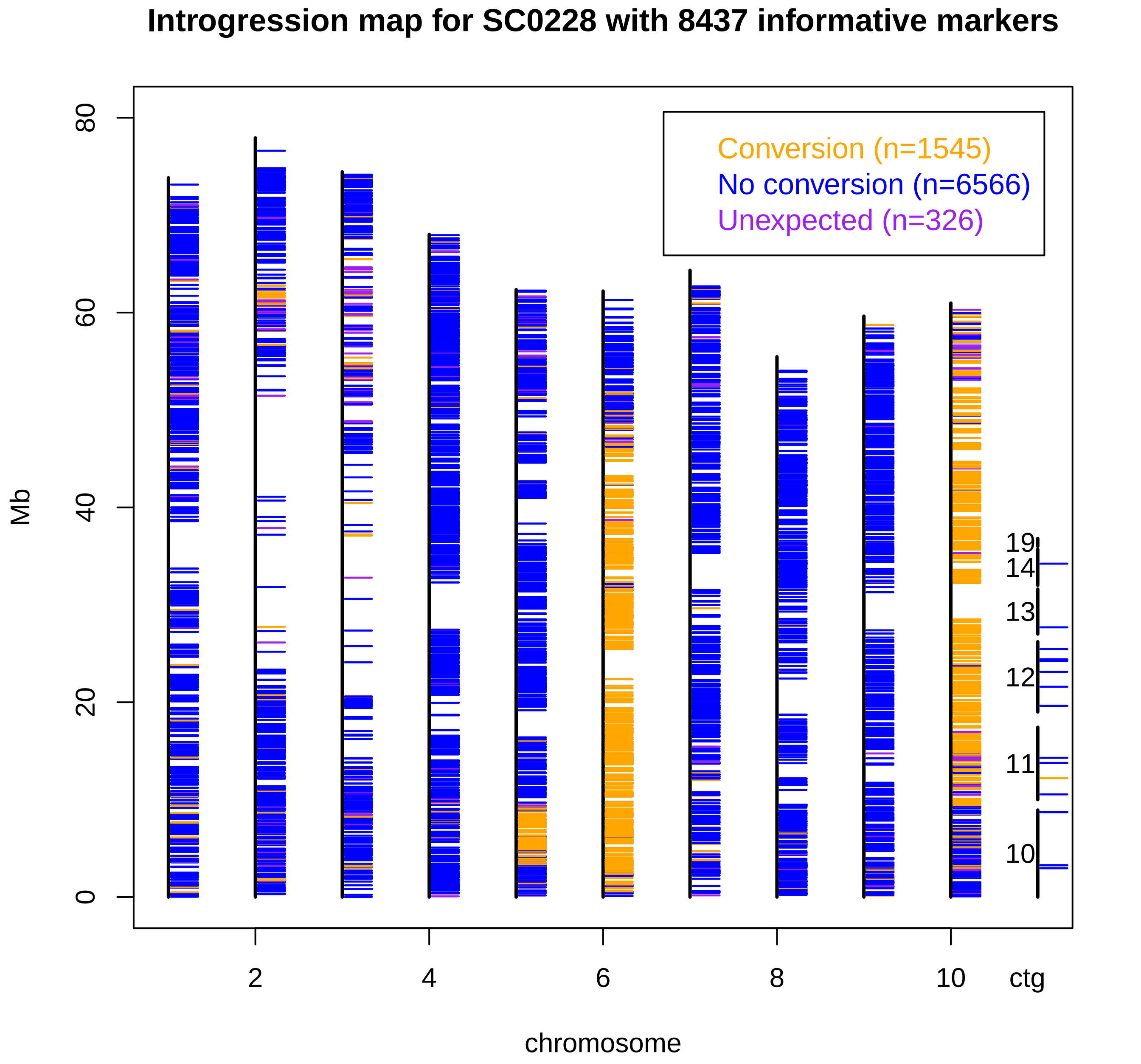

Introgression map for SC0228 with 8437 informative markers
Conversion (n=1545)
No conversion (n=6566)
Unexpected (n=326)
Mb
0
20
40
60
80
chromosome
2
4
6
8
10
ctg
19
14
13
12
11
10

Introgression map for SC0230 with 8549 informative markers

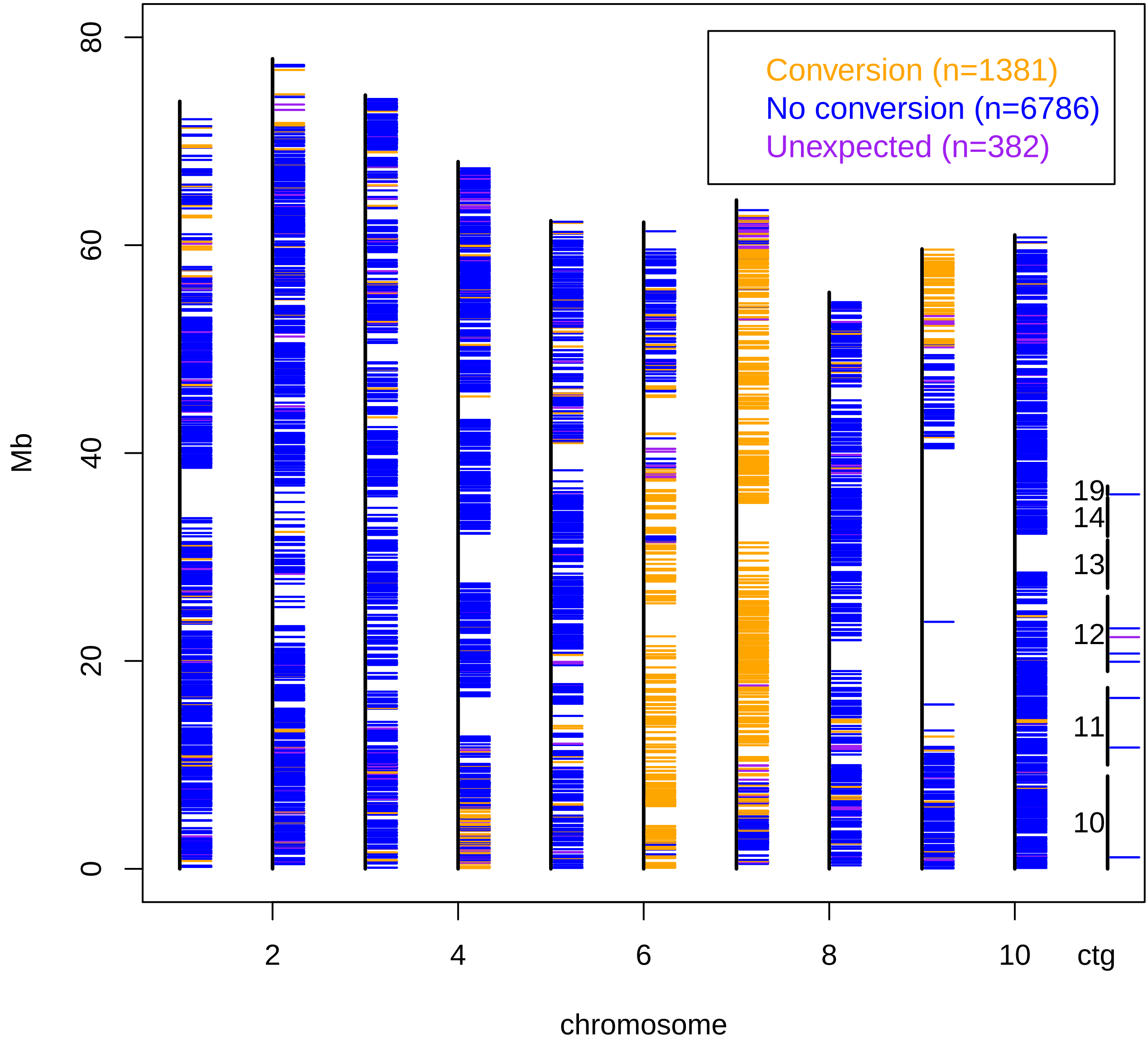

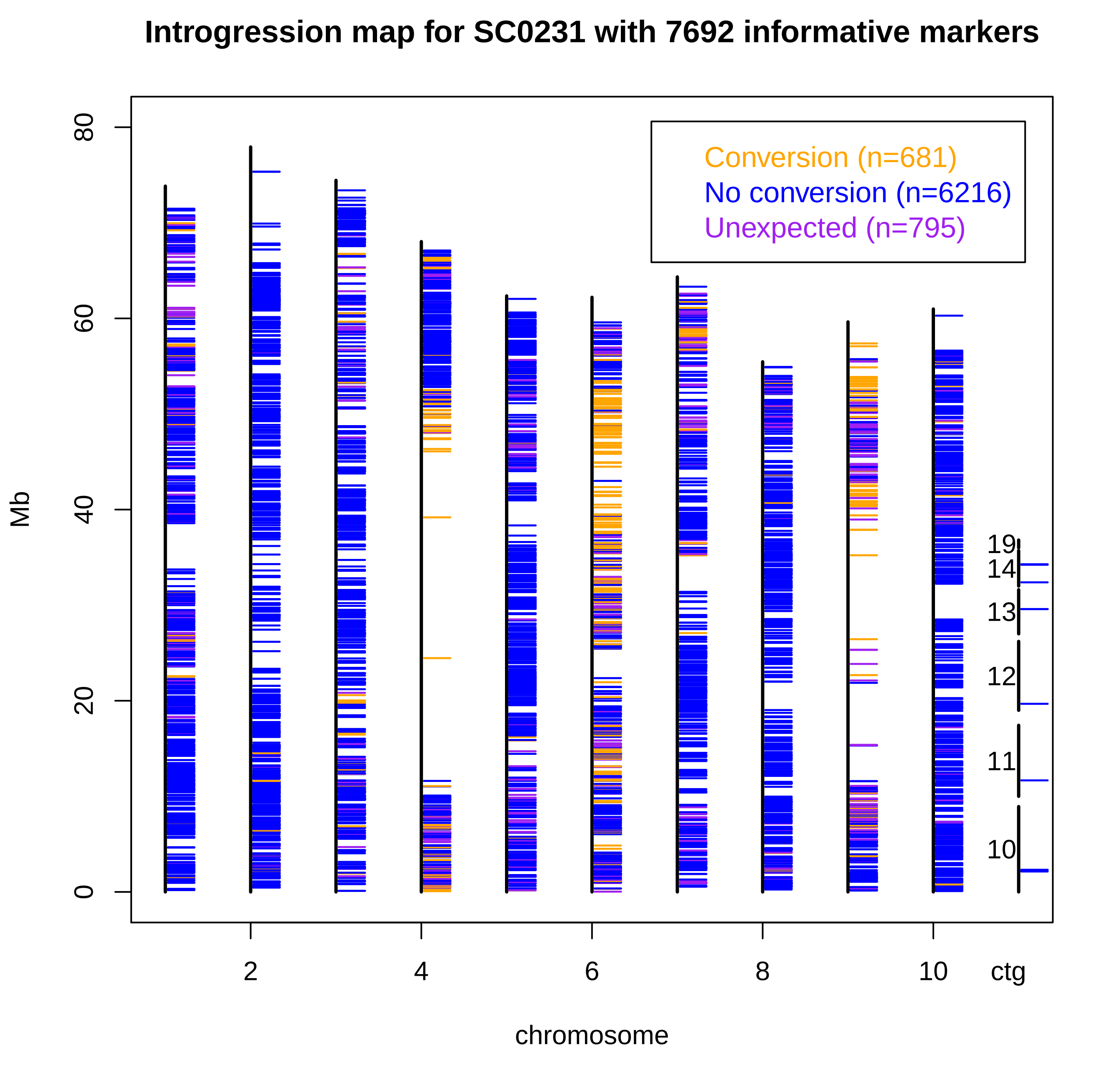

Introgression map for SC0231 with 7692 informative markers
Conversion (n=681)
No conversion (n=6216)
Unexpected (n=795)
Mb
0
20
40
60
80
2
4
6
8
10
ctg
chromosome
19
14
13
12
11
10

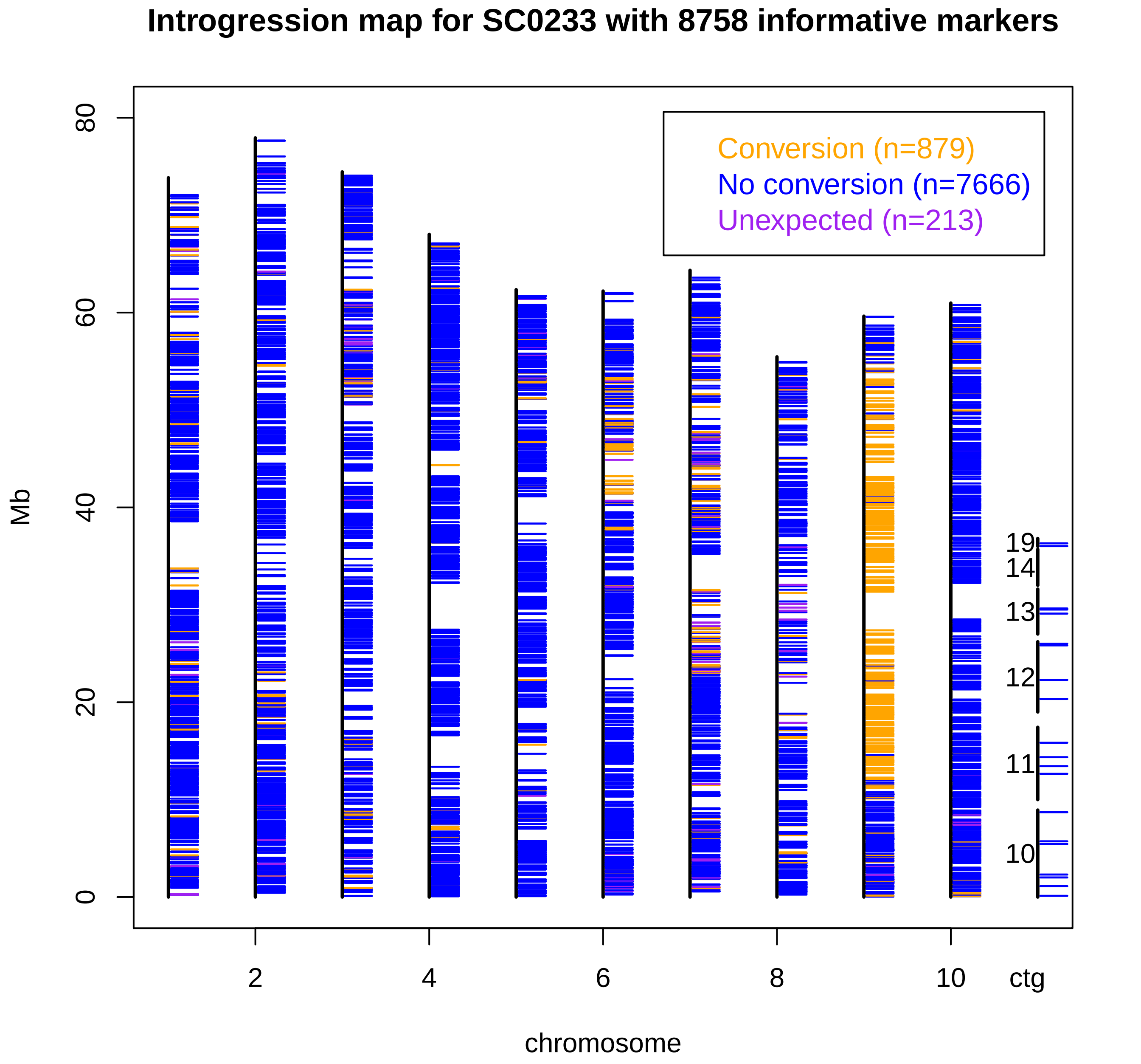

Introgression map for SC0233 with 8758 informative markers
Conversion (n=879)
No conversion (n=7666)
Unexpected (n=213)
Mb
0
20
40
60
80
2
4
6
8
10
ctg
chromosome
19
14
13
12
11
10

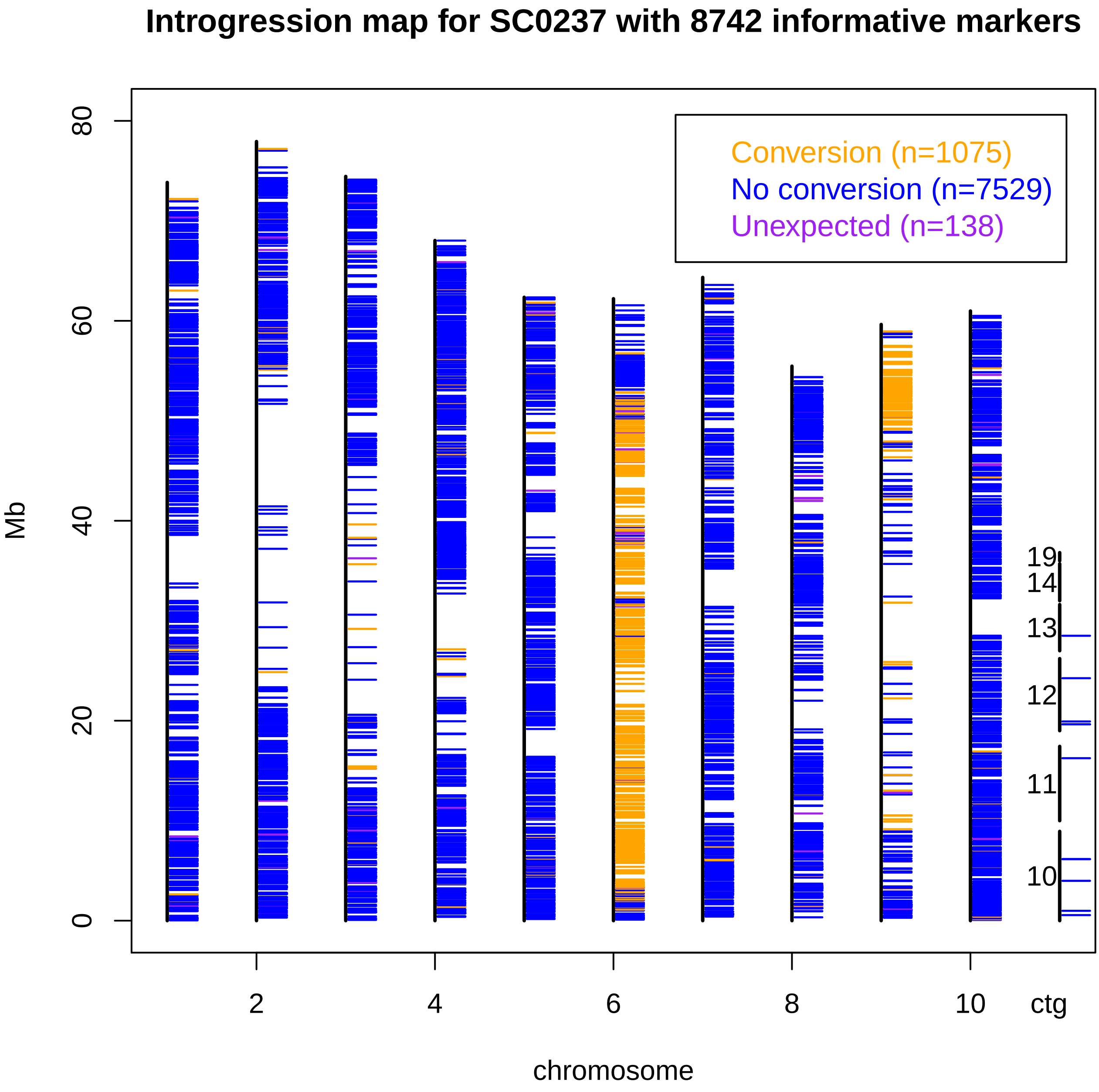

Introgression map for SC0237 with 8742 informative markers
Conversion (n=1075)
No conversion (n=7529)
Unexpected (n=138)
Mb
0
20
40
60
80
chromosome
2
4
6
8
10
ctg
19
14
13
12
11
10

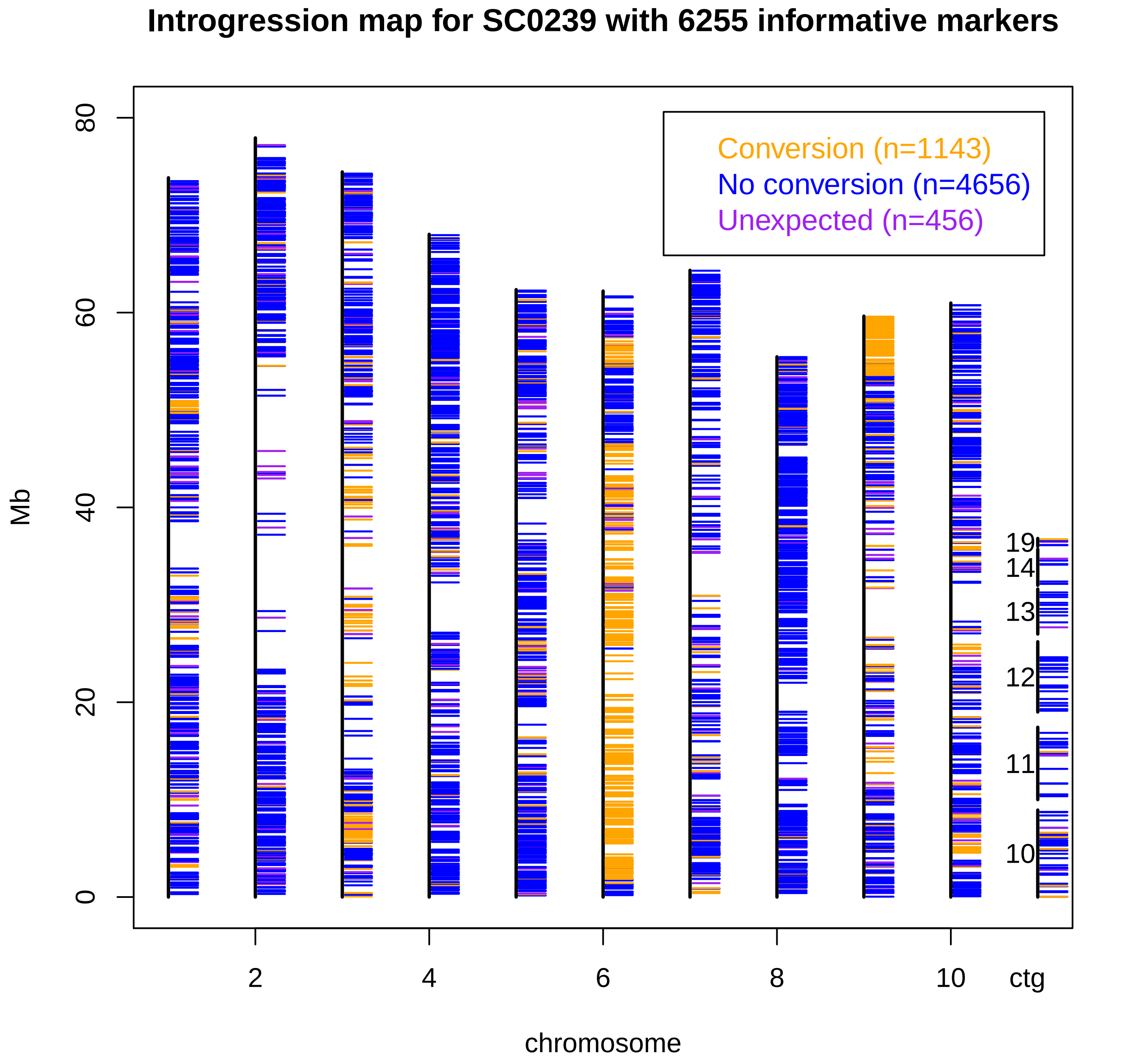

Introgression map for SC0239 with 6255 informative markers
Conversion (n=1143)
No conversion (n=4656)
Unexpected (n=456)
Mb
0
20
40
60
80
2
4
6
8
10
ctg
19
14
13
12
11
10
chromosome

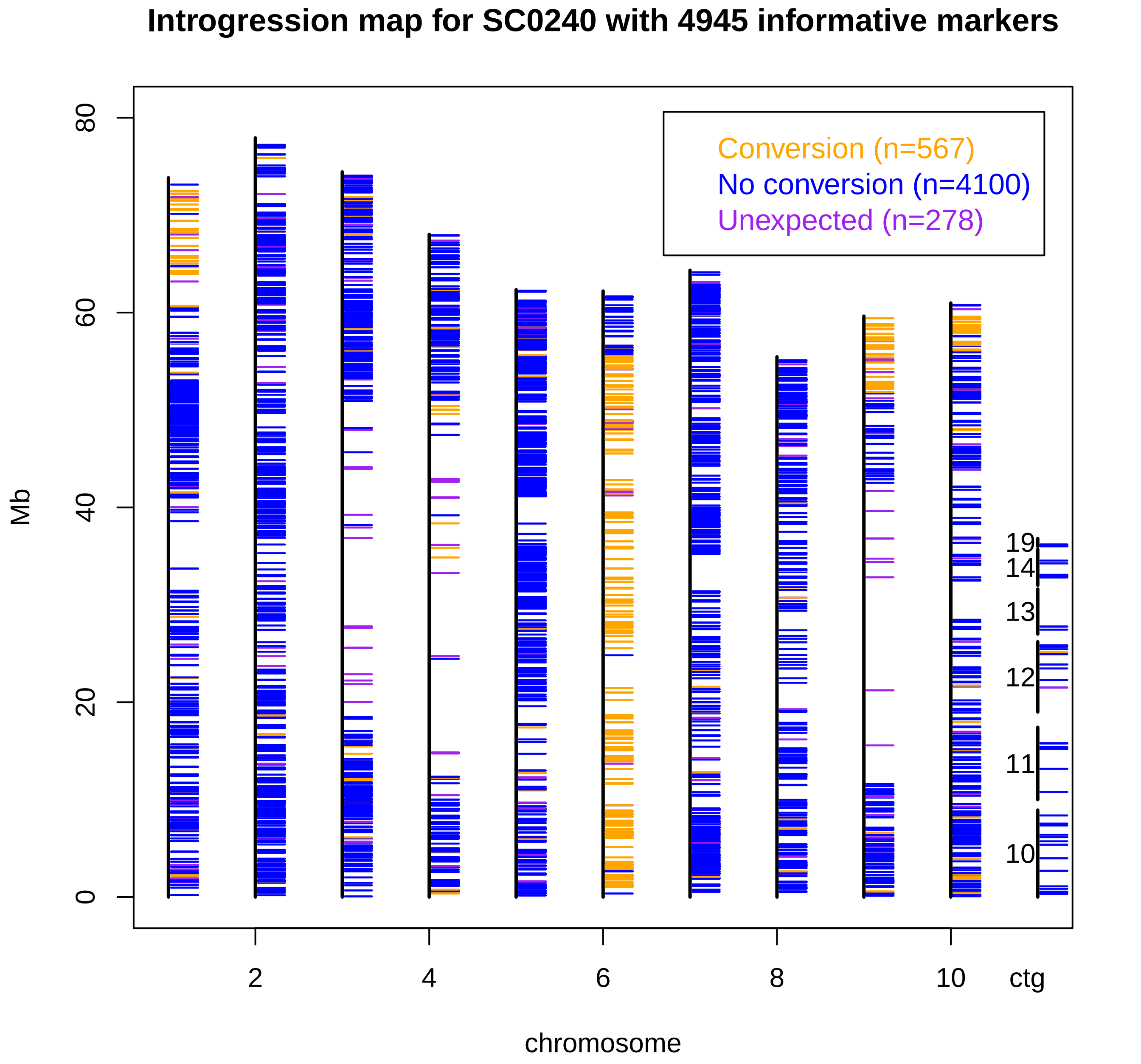
Introgression map for SC0240 with 4945 informative markers
Conversion (n=567)
No conversion (n=4100)
Unexpected (n=278)
Mb
0
20
40
60
80
2
4
6
8
10
ctg
19
14
13
12
11
10
chromosome

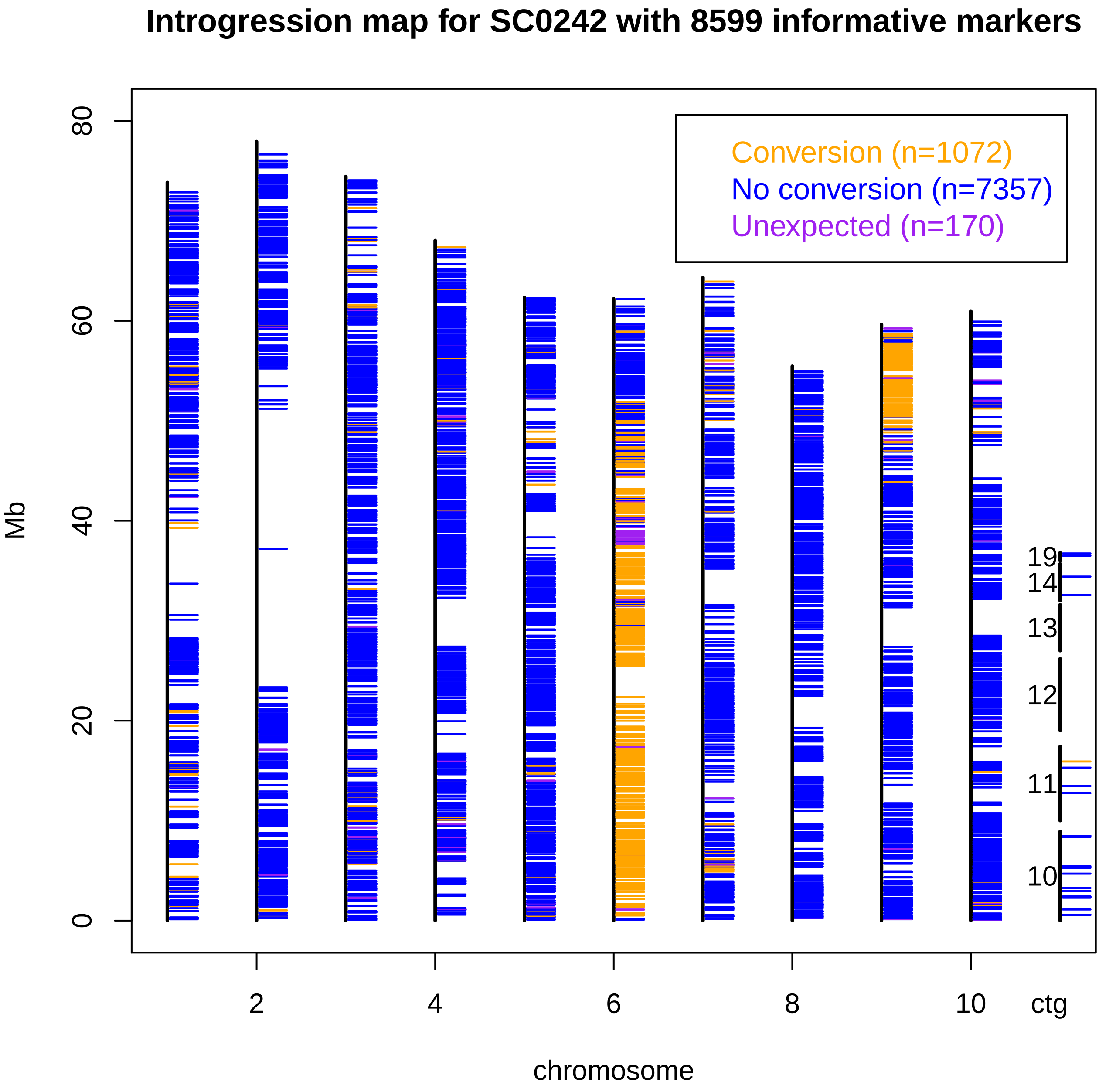
Introgression map for SC0242 with 8599 informative markers
Conversion (n=1072)
No conversion (n=7357)
Unexpected (n=170)
Mb
0
20
40
60
80
chromosome
2
4
6
8
10
ctg
19
14
13
12
11
10

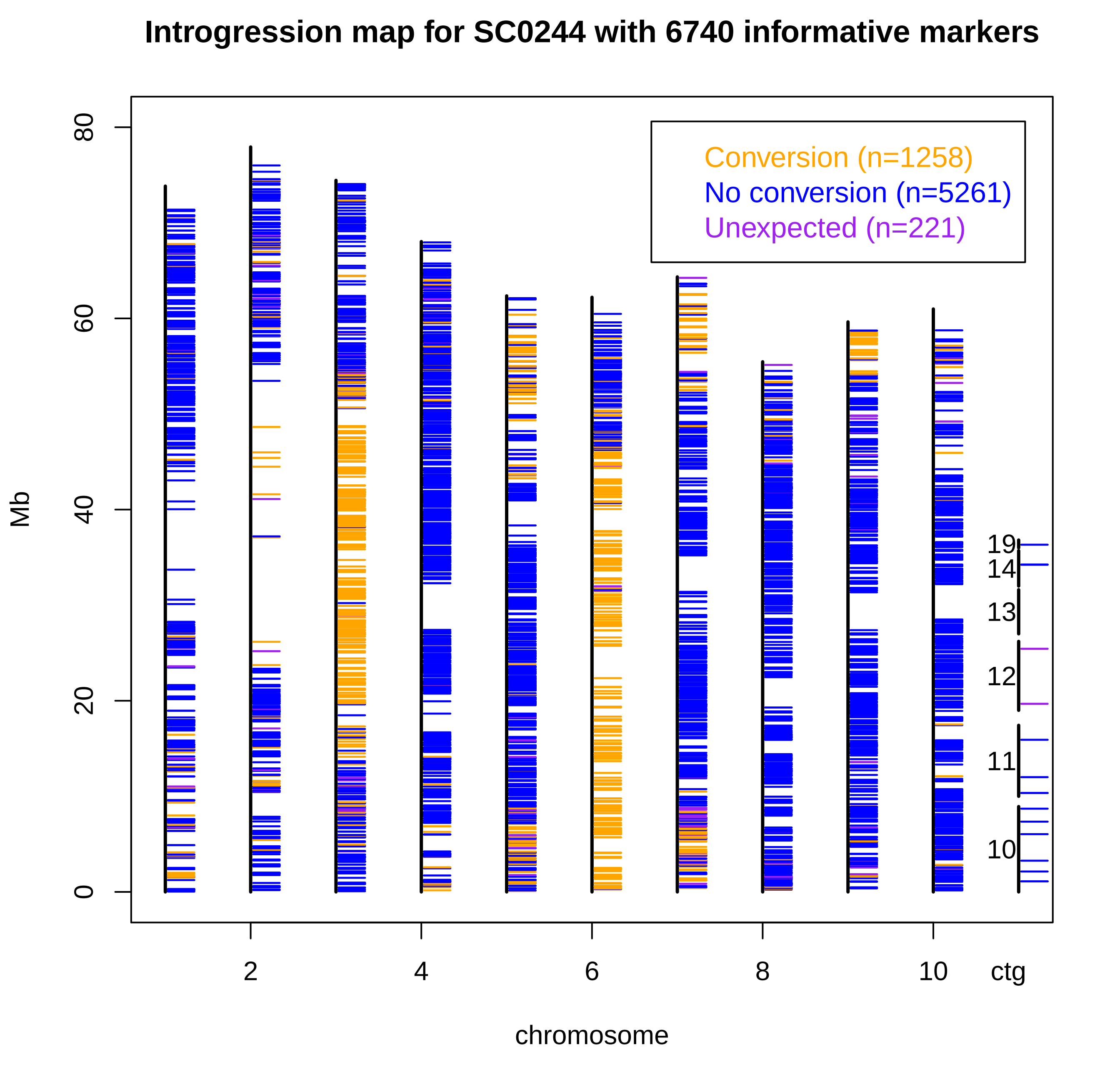
Introgression map for SC0244 with 6740 informative markers
Conversion (n=1258)
No conversion (n=5261)
Unexpected (n=221)
Mb
0
20
40
60
80
2
4
6
8
10
ctg
19
14
13
12
11
10
chromosome

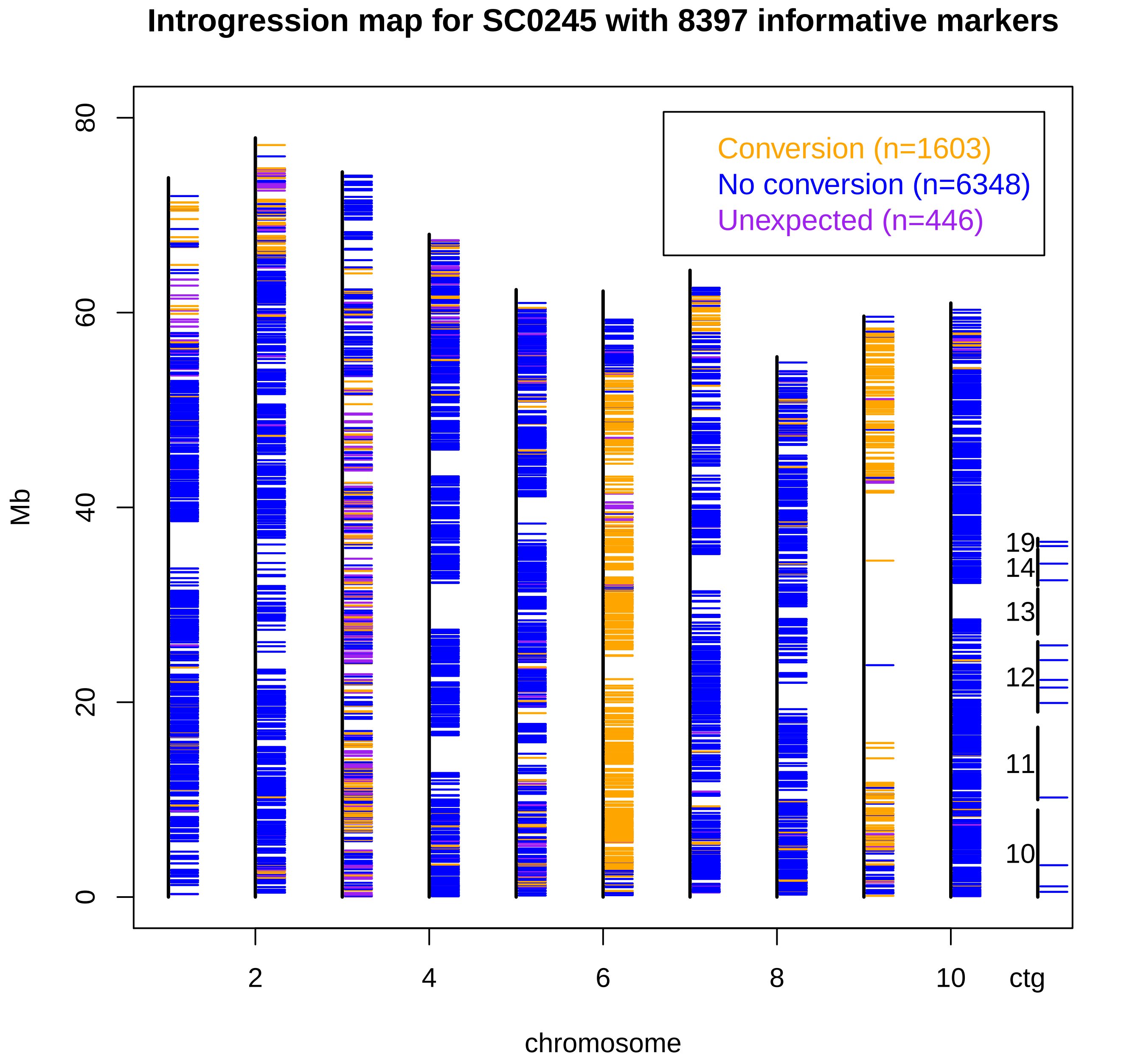

Introgression map for SC0245 with 8397 informative markers
Conversion (n=1603)
No conversion (n=6348)
Unexpected (n=446)
Mb
0
20
40
60
80
chromosome
2
4
6
8
10
ctg
19
14
13
12
11
10

Introgression map for SC0248 with 6851 informative markers

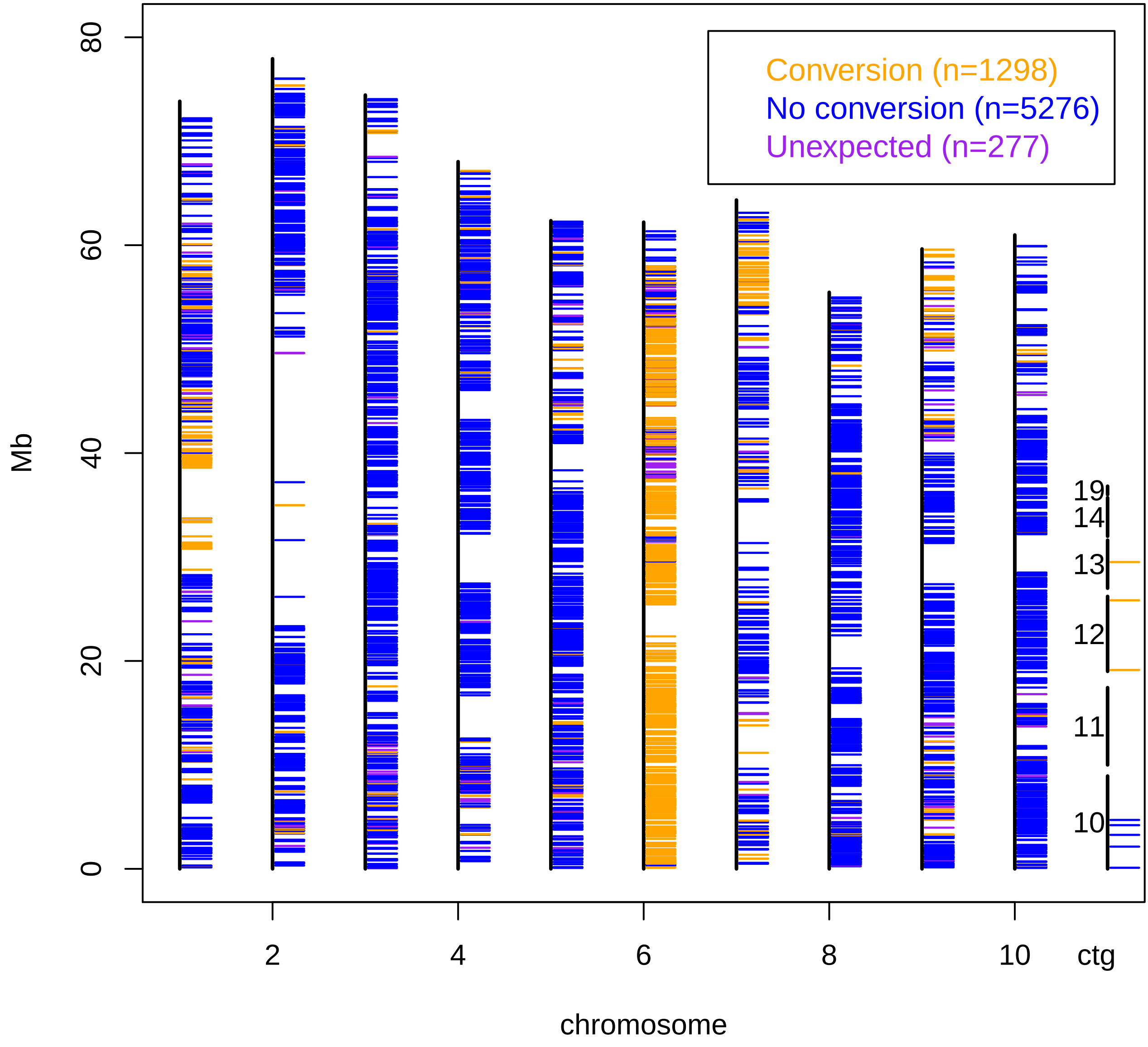

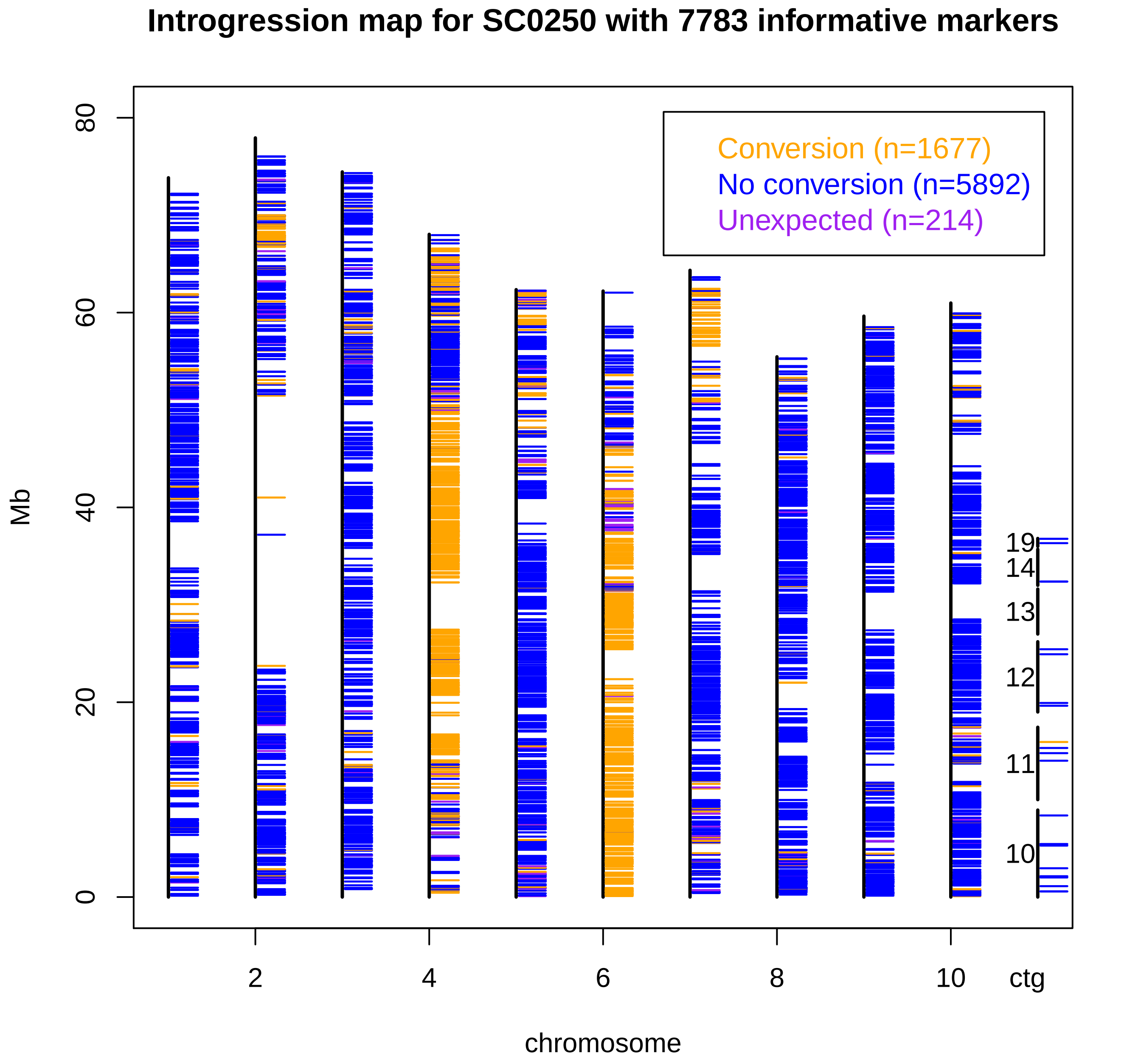

Introgression map for SC0250 with 7783 informative markers
Conversion (n=1677)
No conversion (n=5892)
Unexpected (n=214)
Mb
0
20
40
60
80
2
4
6
8
10
ctg
19
14
13
12
11
10
chromosome

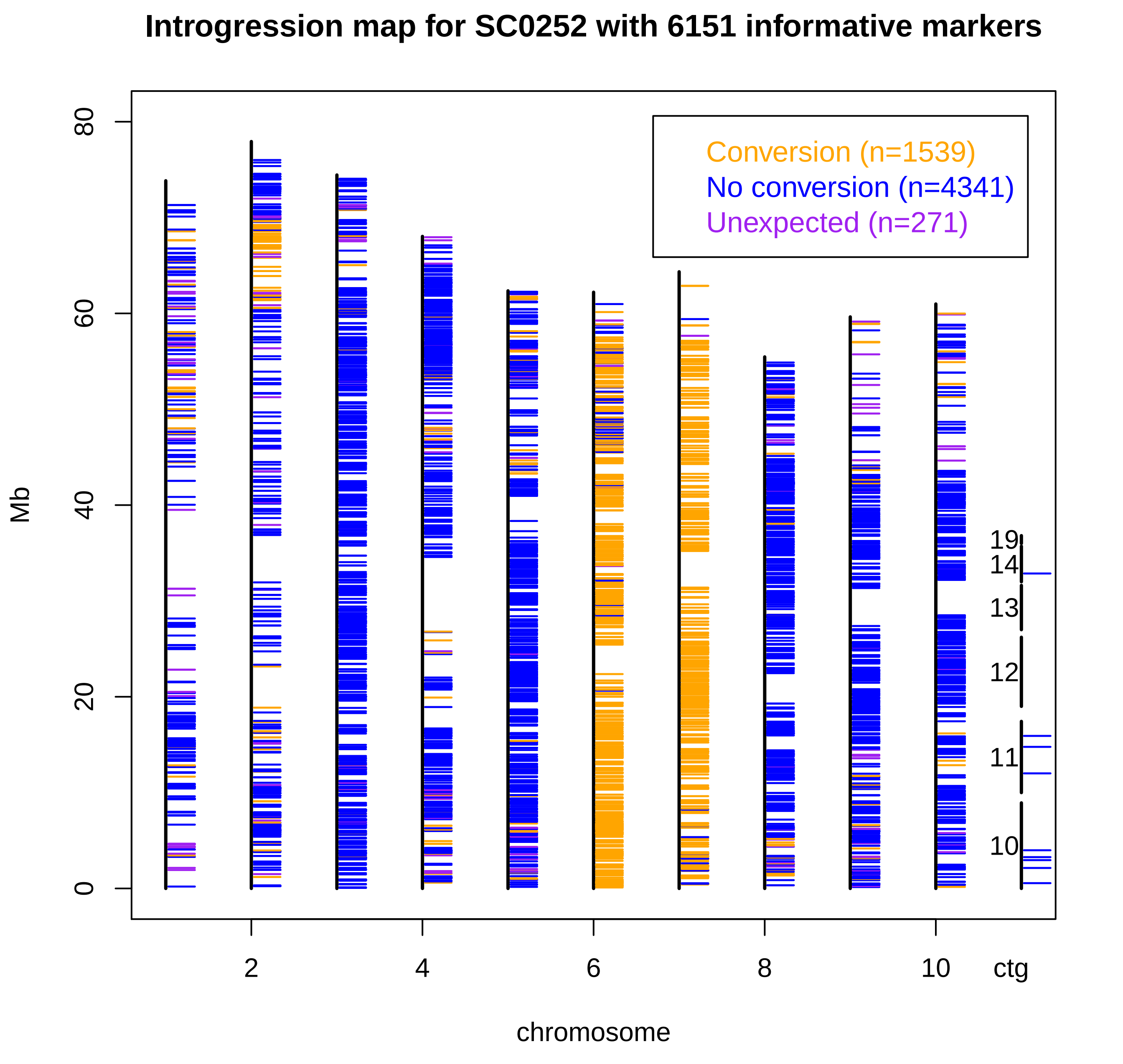
Introgression map for SC0252 with 6151 informative markers
Conversion (n=1539)
No conversion (n=4341)
Unexpected (n=271)
Mb
0
20
40
60
80
chromosome
2
4
6
8
10
ctg
19
14
13
12
11
10

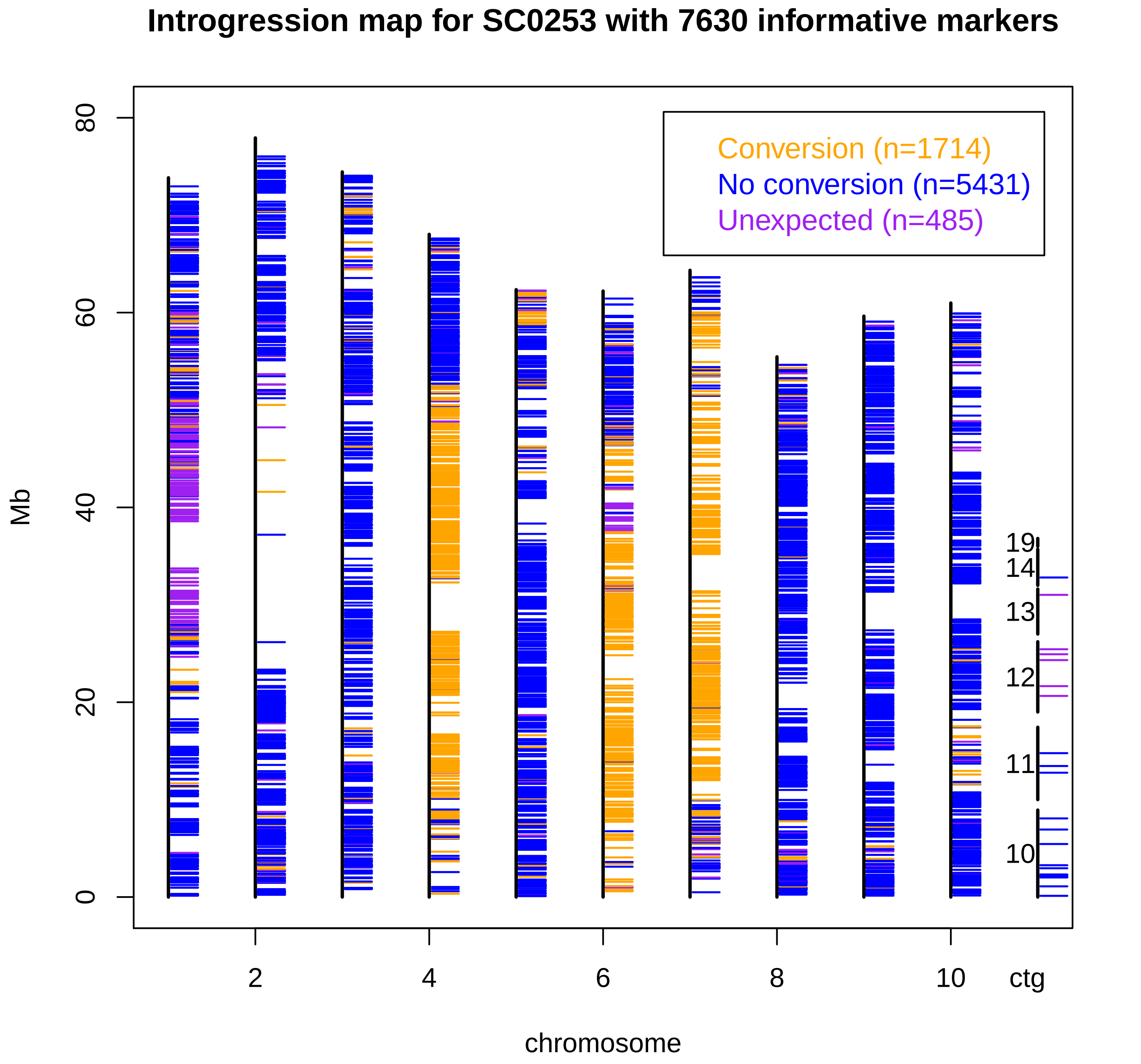

Introgression map for SC0253 with 7630 informative markers
Conversion (n=1714)
No conversion (n=5431)
Unexpected (n=485)
Mb
0
20
40
60
80
chromosome
2
4
6
8
10
ctg
19
14
13
12
11
10

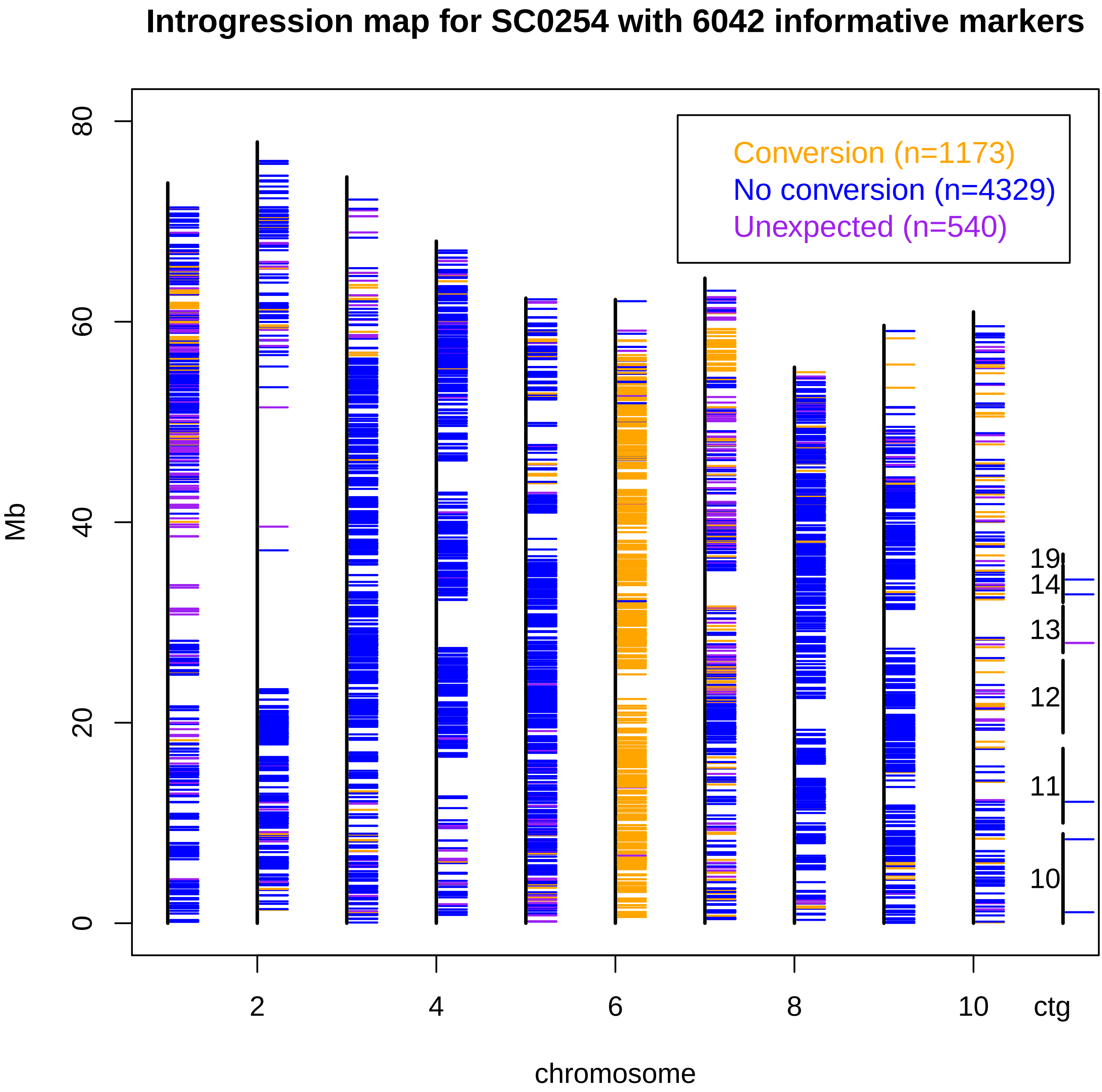

Introgression map for SC0254 with 6042 informative markers
Conversion (n=1173)
No conversion (n=4329)
Unexpected (n=540)
Mb
0
20
40
60
80
chromosome
2
4
6
8
10
ctg
19
14
13
12
11
10

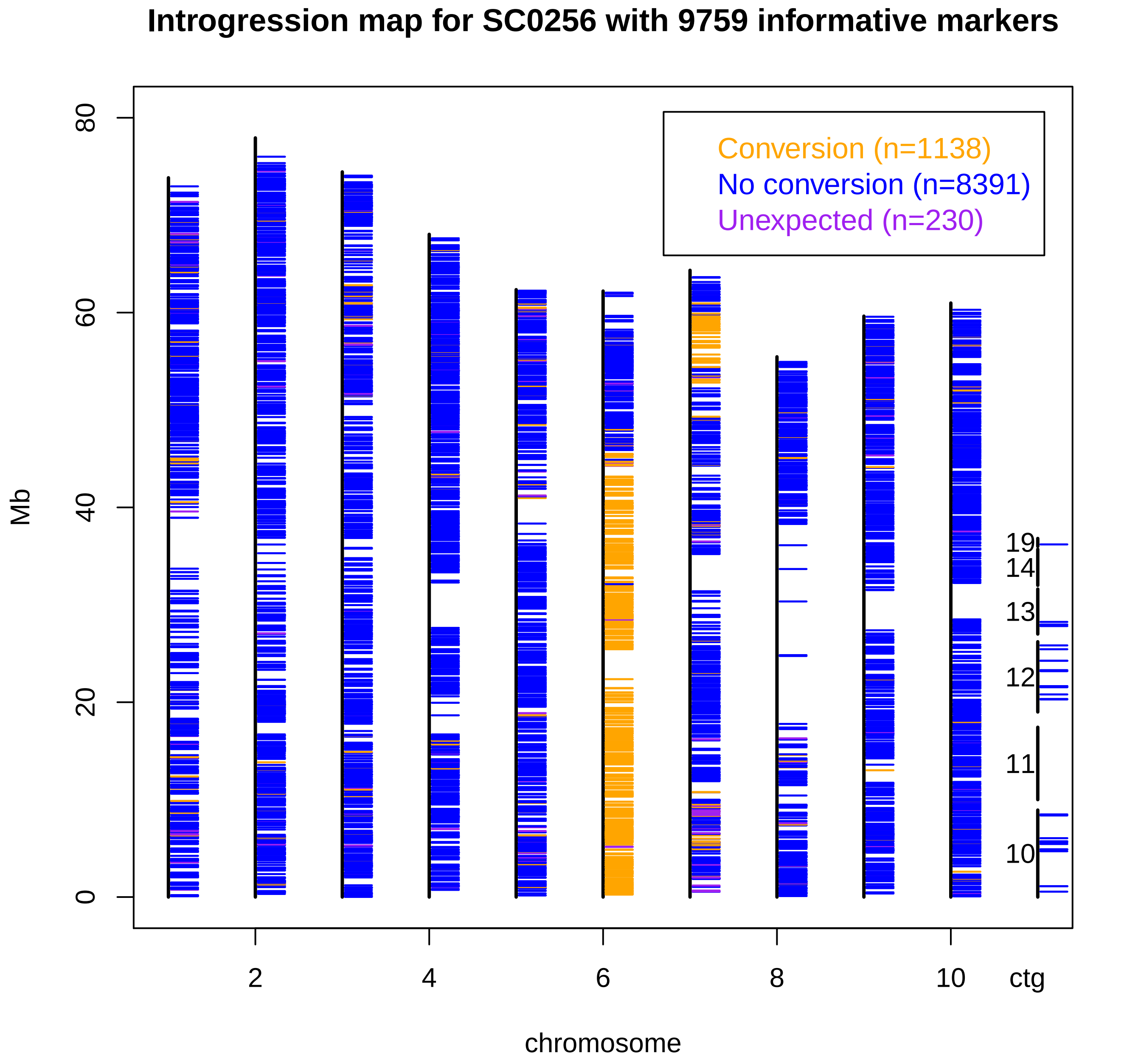

Introgression map for SC0256 with 9759 informative markers
Conversion (n=1138)
No conversion (n=8391)
Unexpected (n=230)
Mb
0
20
40
60
80
chromosome
2
4
6
8
10
ctg
19
14
13
12
11
10

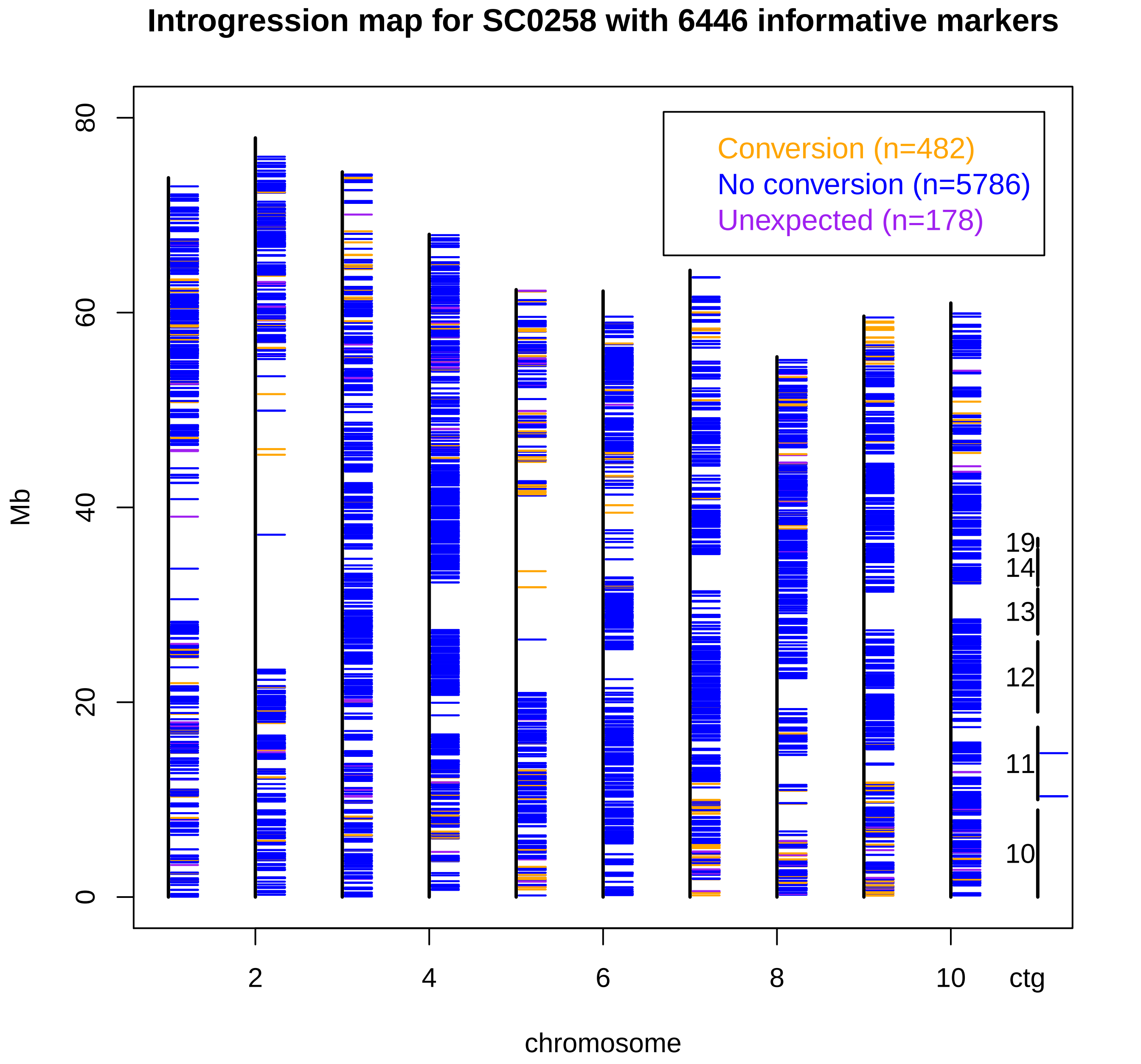

Introgression map for SC0258 with 6446 informative markers
Conversion (n=482)
No conversion (n=5786)
Unexpected (n=178)
Mb
0
20
40
60
80
2
4
6
8
10
ctg
chromosome
19
14
13
12
11
10

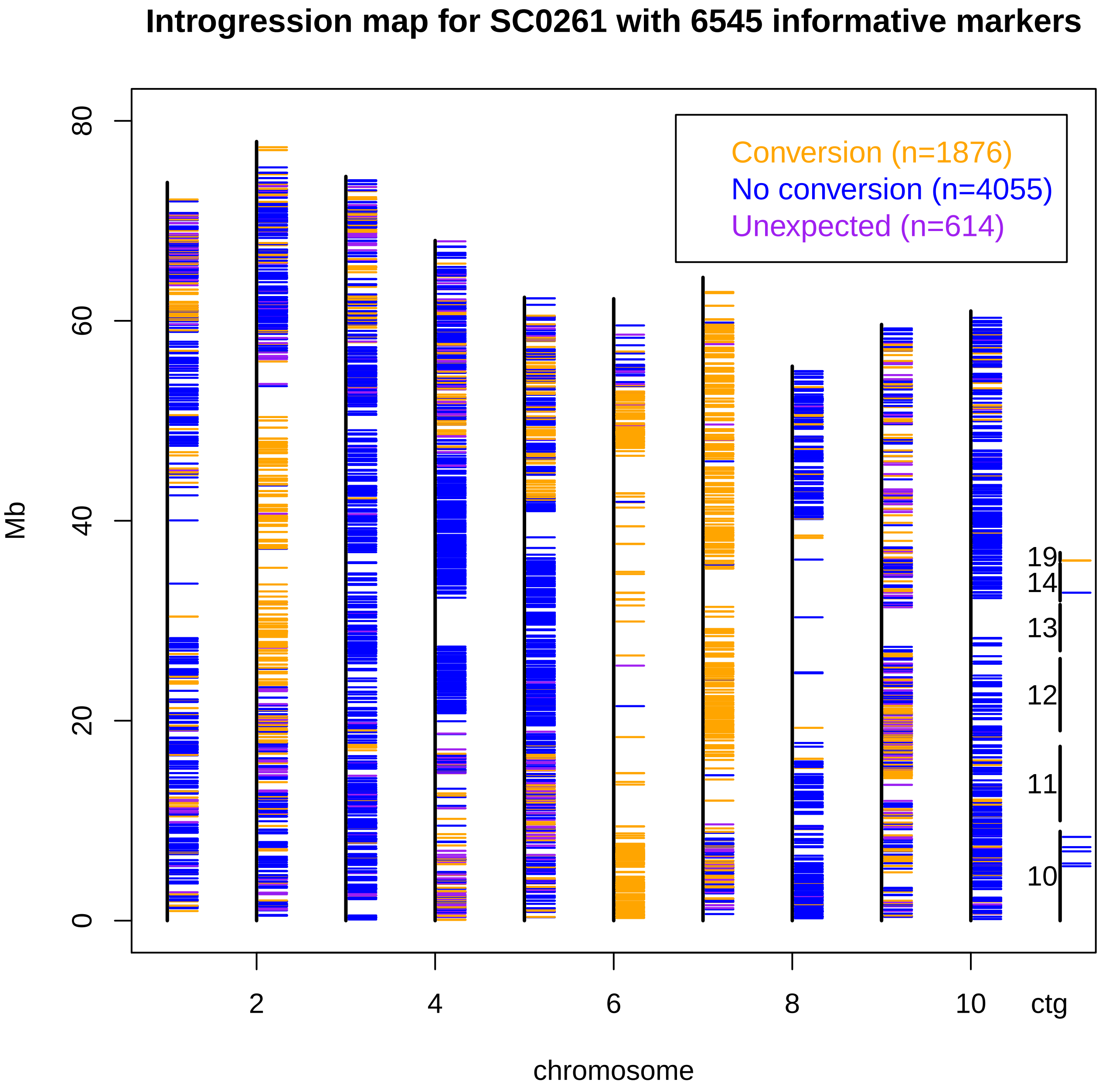
Introgression map for SC0261 with 6545 informative markers
Conversion (n=1876)
No conversion (n=4055)
Unexpected (n=614)
Mb
0
20
40
60
80
2
4
6
8
10
ctg
19
14
13
12
11
10
chromosome

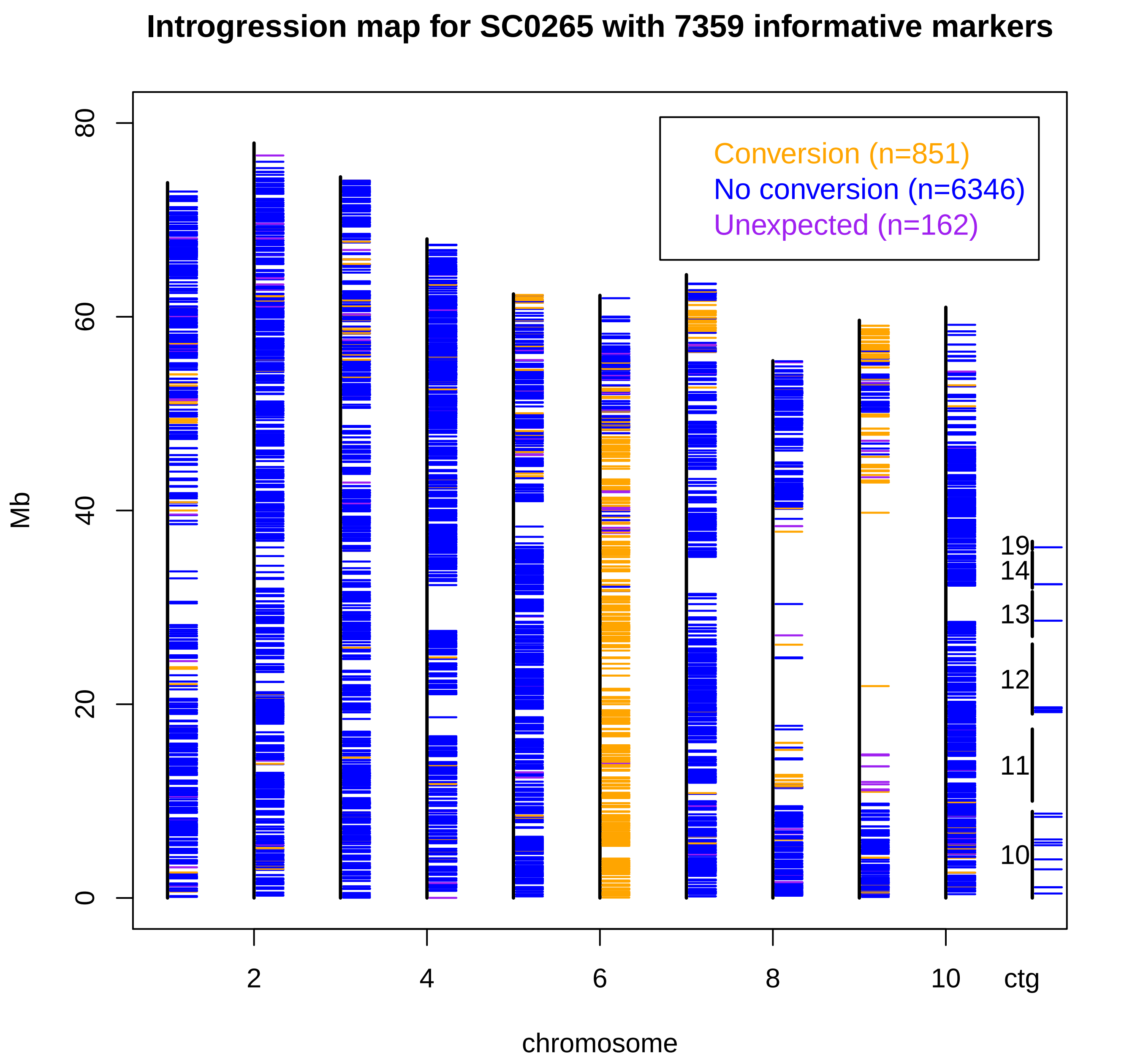

Introgression map for SC0265 with 7359 informative markers
Conversion (n=851)
No conversion (n=6346)
Unexpected (n=162)
Mb
0
20
40
60
80
chromosome
2
4
6
8
10
ctg
19
14
13
12
11
10

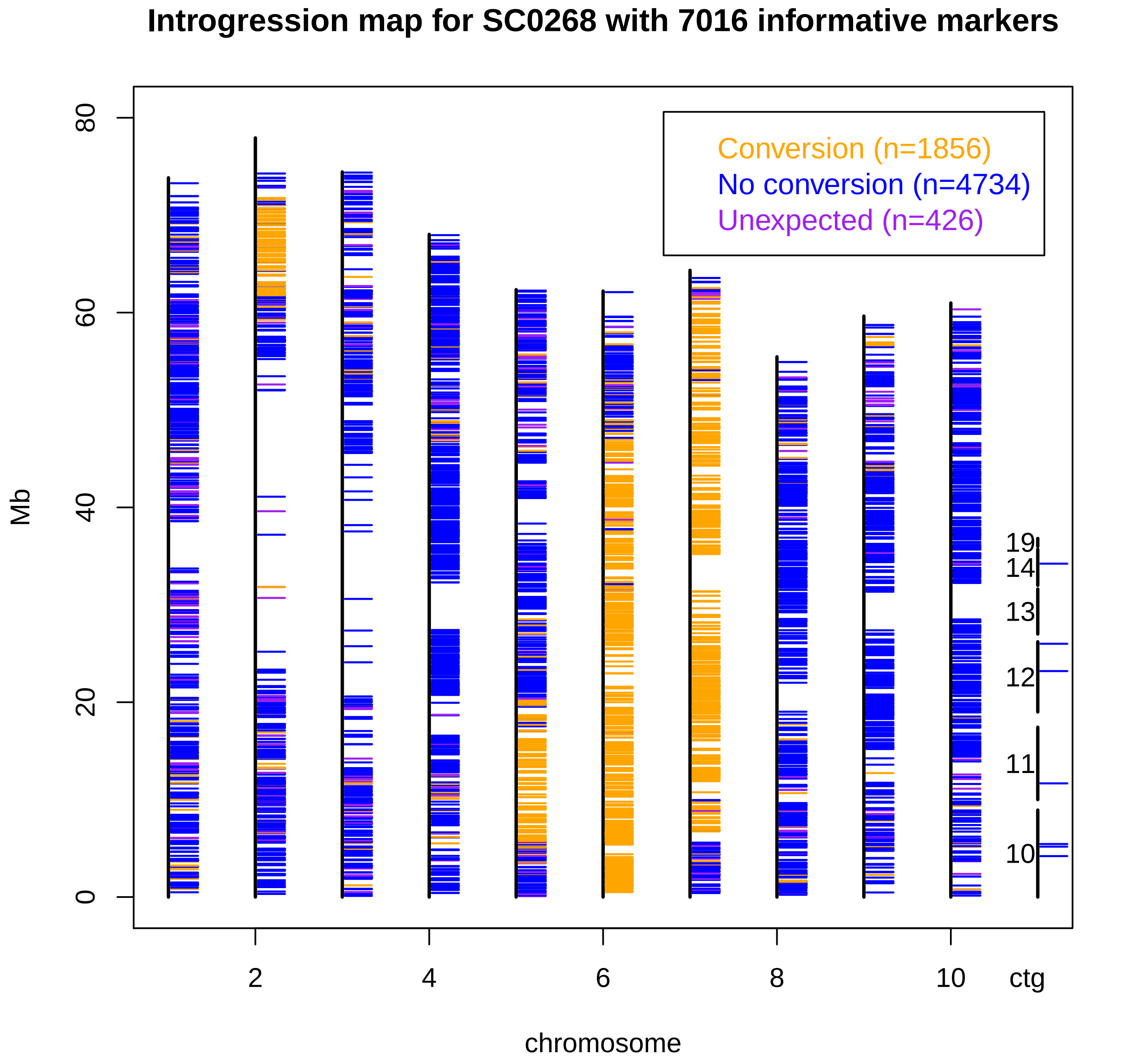

Introgression map for SC0268 with 7016 informative markers
Conversion (n=1856)
No conversion (n=4734)
Unexpected (n=426)
Mb
0
20
40
60
80
2
4
6
8
10
ctg
19
14
13
12
11
10
chromosome

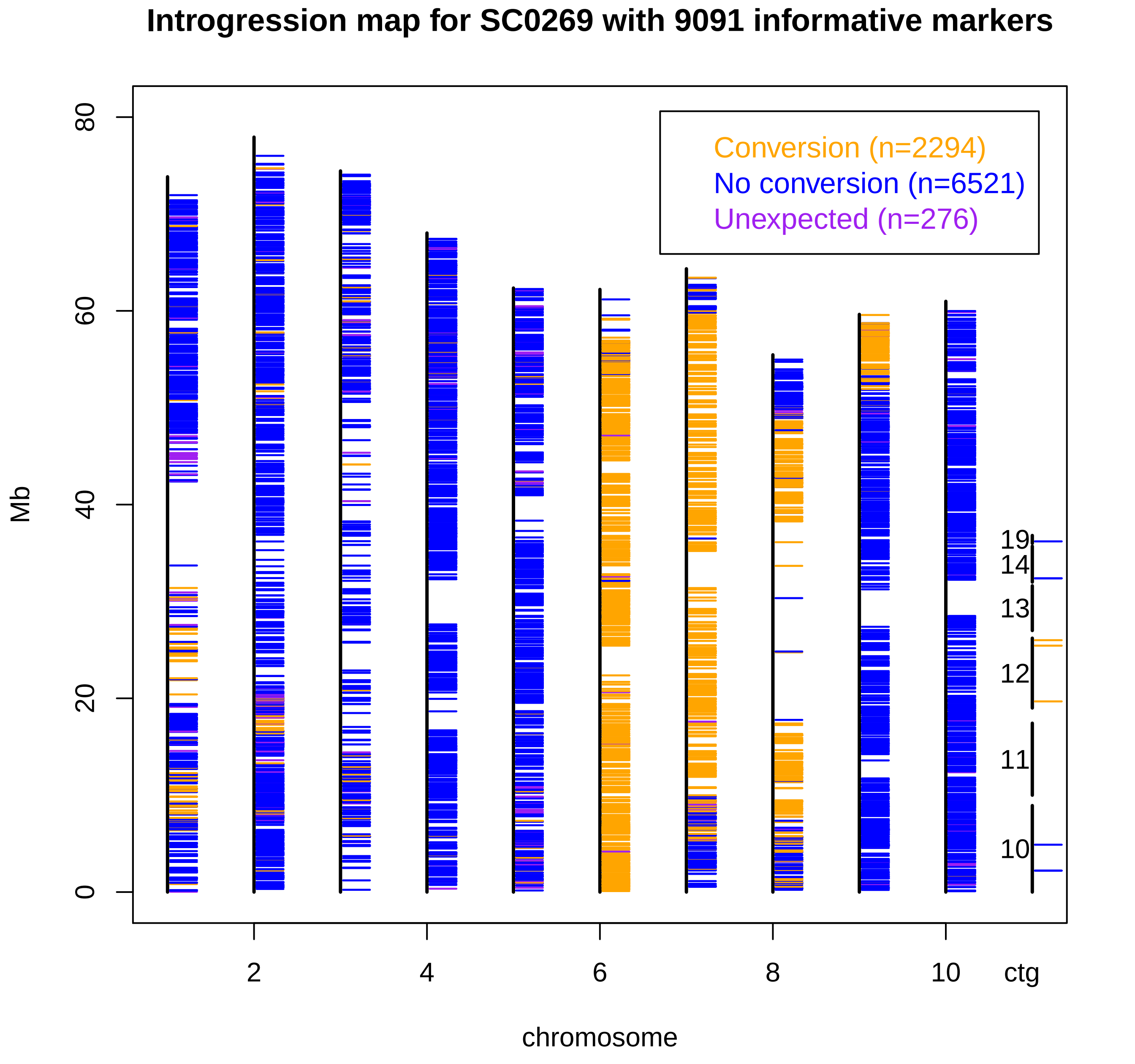

Introgression map for SC0269 with 9091 informative markers
Conversion (n=2294)
No conversion (n=6521)
Unexpected (n=276)
Mb
0
20
40
60
80
2
4
6
8
10
ctg
19
14
13
12
11
10
chromosome

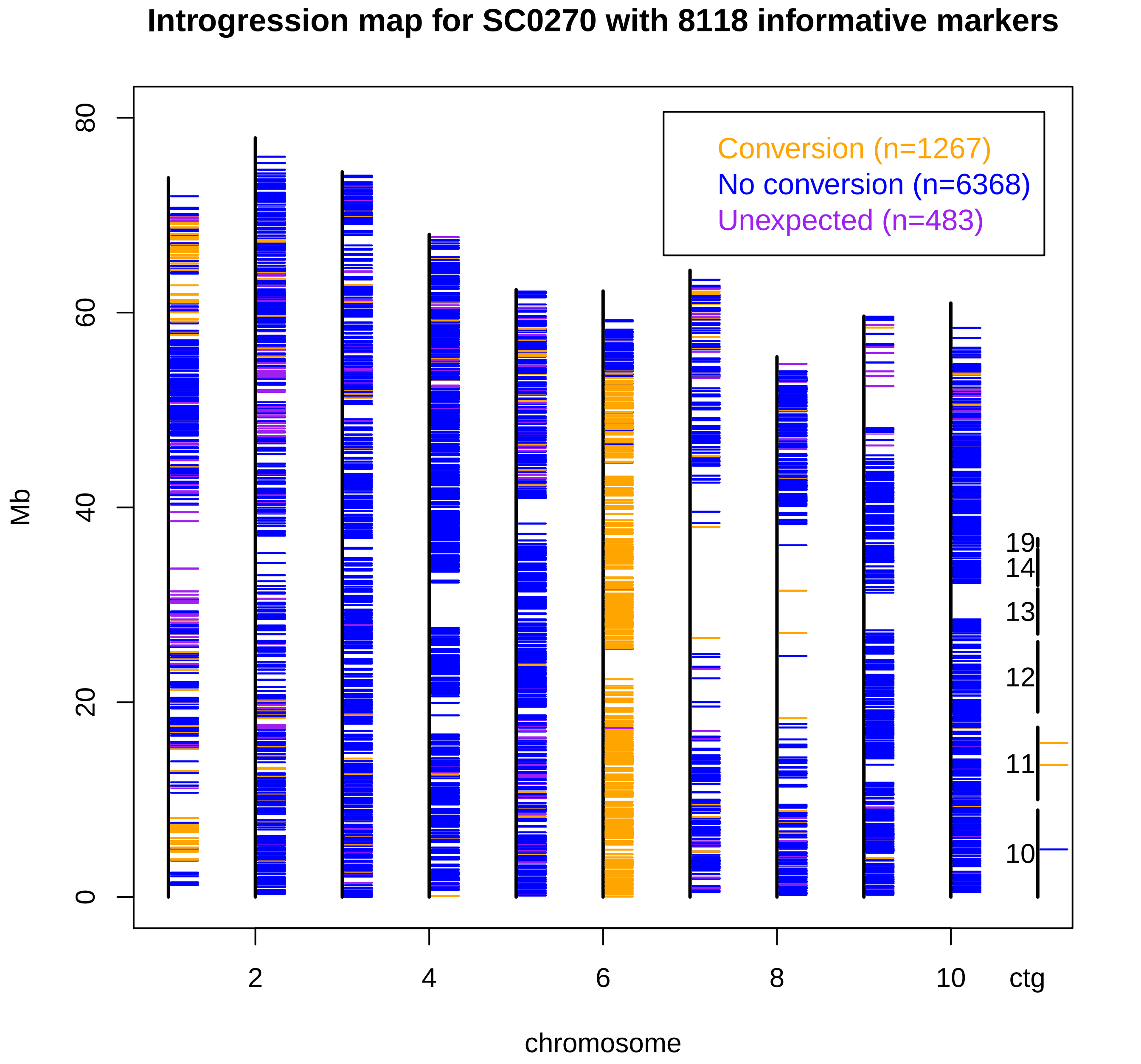

Introgression map for SC0270 with 8118 informative markers
Conversion (n=1267)
No conversion (n=6368)
Unexpected (n=483)
Mb
0
20
40
60
80
chromosome
2
4
6
8
10
ctg
19
14
13
12
11
10

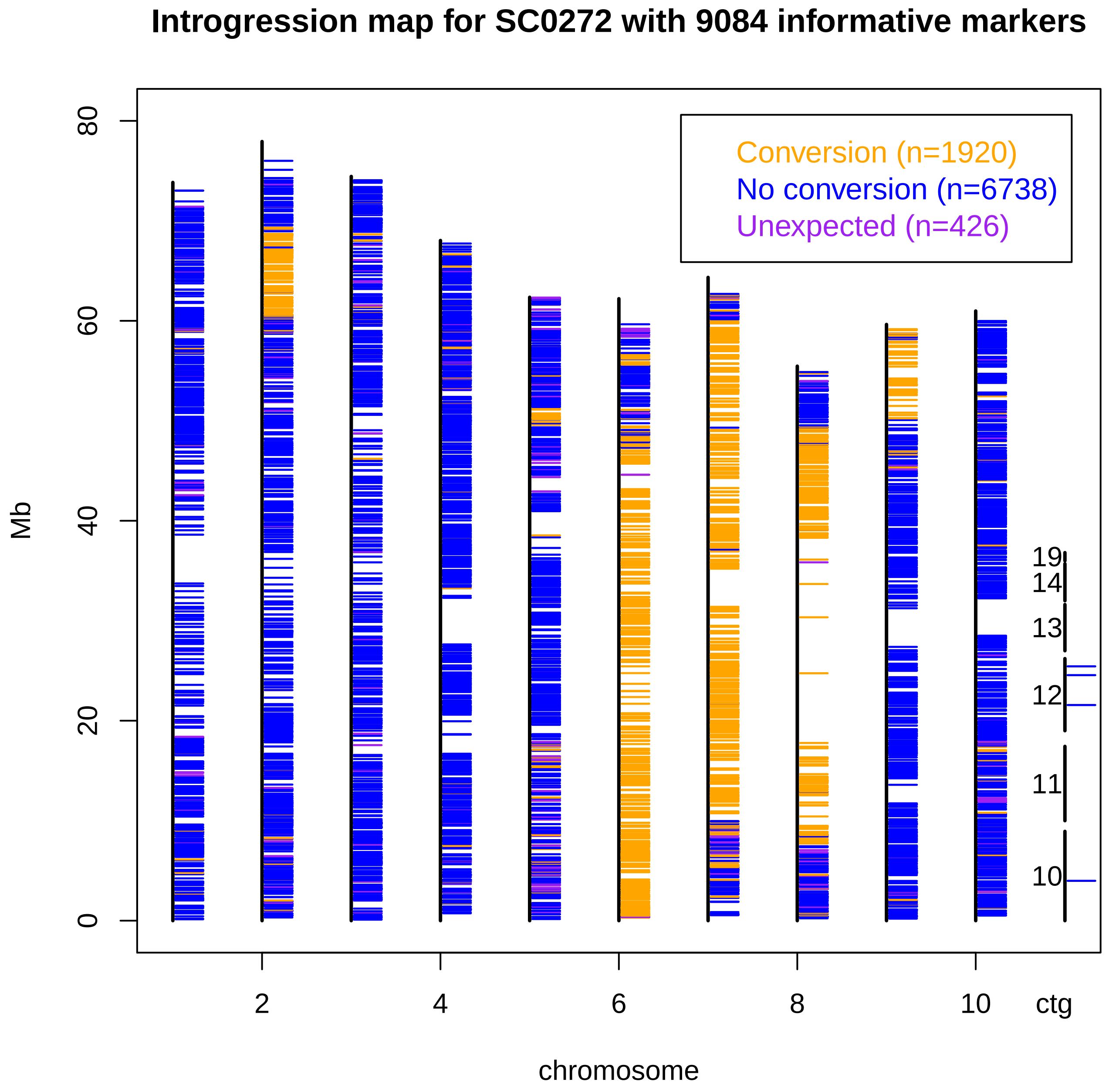
Introgression map for SC0272 with 9084 informative markers
Conversion (n=1920)
No conversion (n=6738)
Unexpected (n=426)
Mb
0
20
40
60
80
chromosome
2
4
6
8
10
ctg
19
14
13
12
11
10

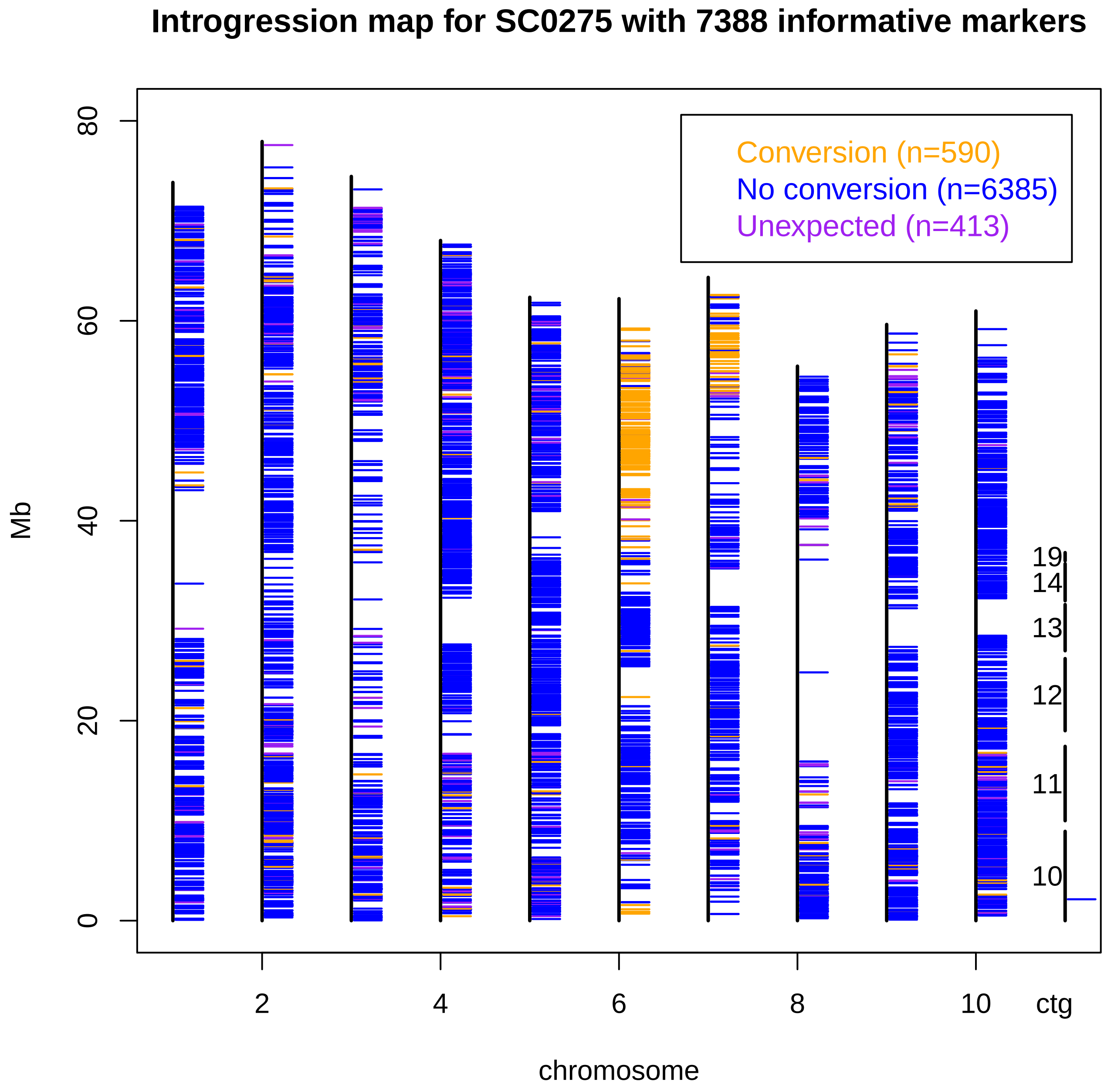

Introgression map for SC0275 with 7388 informative markers
Conversion (n=590)
No conversion (n=6385)
Unexpected (n=413)
Mb
0
20
40
60
80
2
4
6
8
10
ctg
chromosome
19
14
13
12
11
10

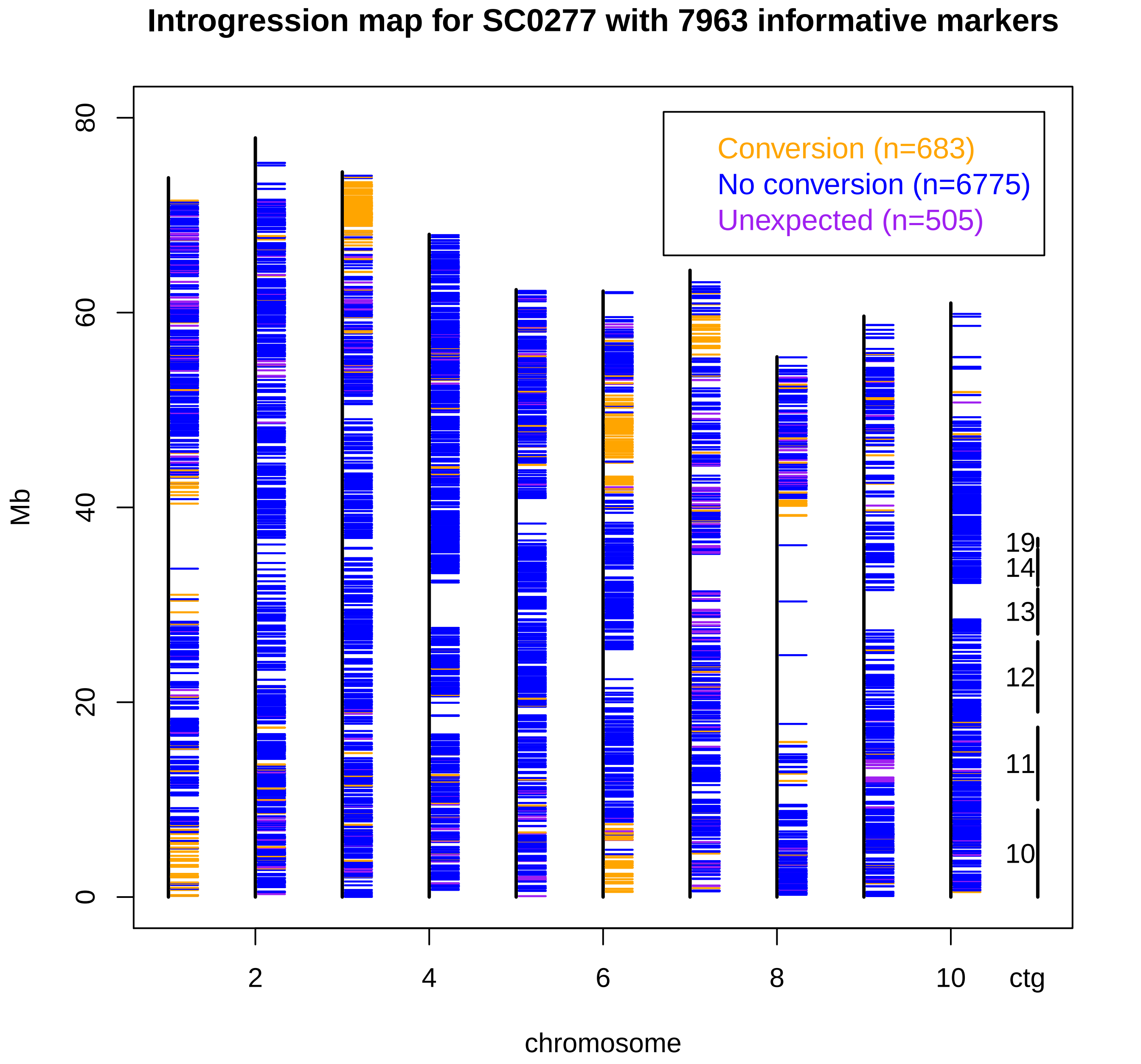

Introgression map for SC0277 with 7963 informative markers
Conversion (n=683)
No conversion (n=6775)
Unexpected (n=505)
Mb
0
20
40
60
80
chromosome
2
4
6
8
10
ctg
19
14
13
12
11
10

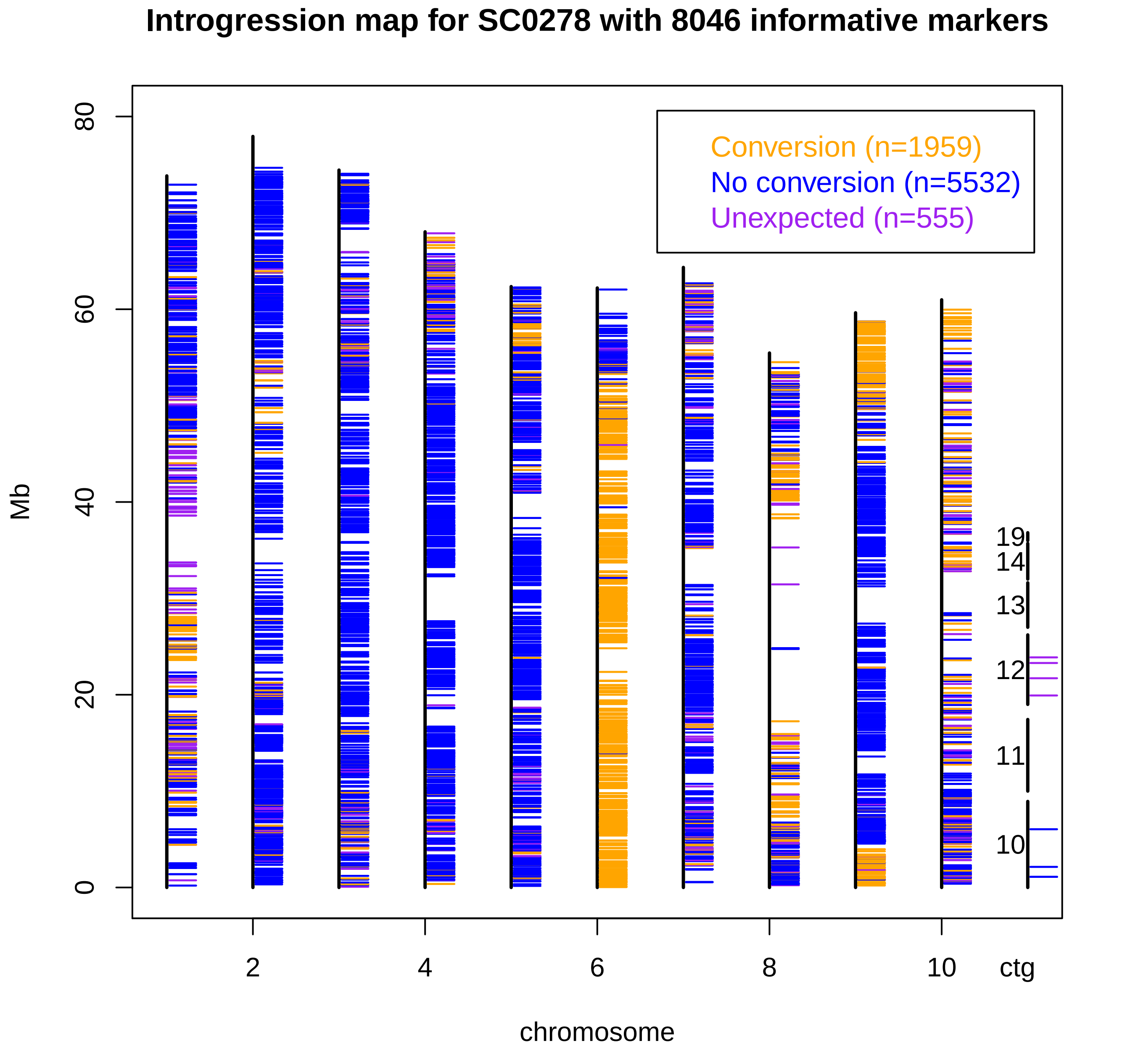
Introgression map for SC0278 with 8046 informative markers
Conversion (n=1959)
No conversion (n=5532)
Unexpected (n=555)
Mb
0
20
40
60
80
2
4
6
8
10
ctg
19
14
13
12
11
10
chromosome

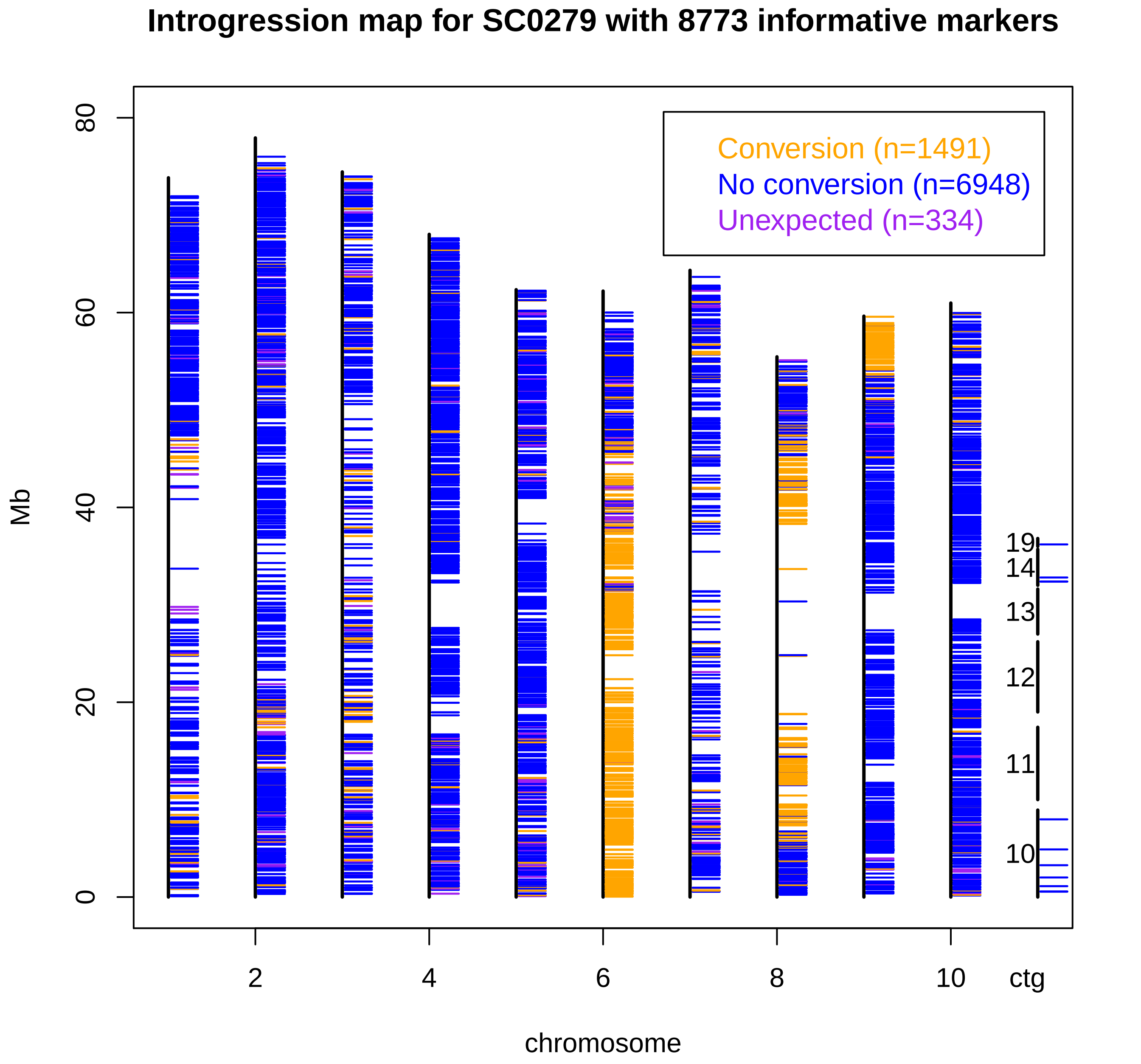
Introgression map for SC0279 with 8773 informative markers
Conversion (n=1491)
No conversion (n=6948)
Unexpected (n=334)
Mb
0
20
40
60
80
2
4
6
8
10
ctg
19
14
13
12
11
10
chromosome

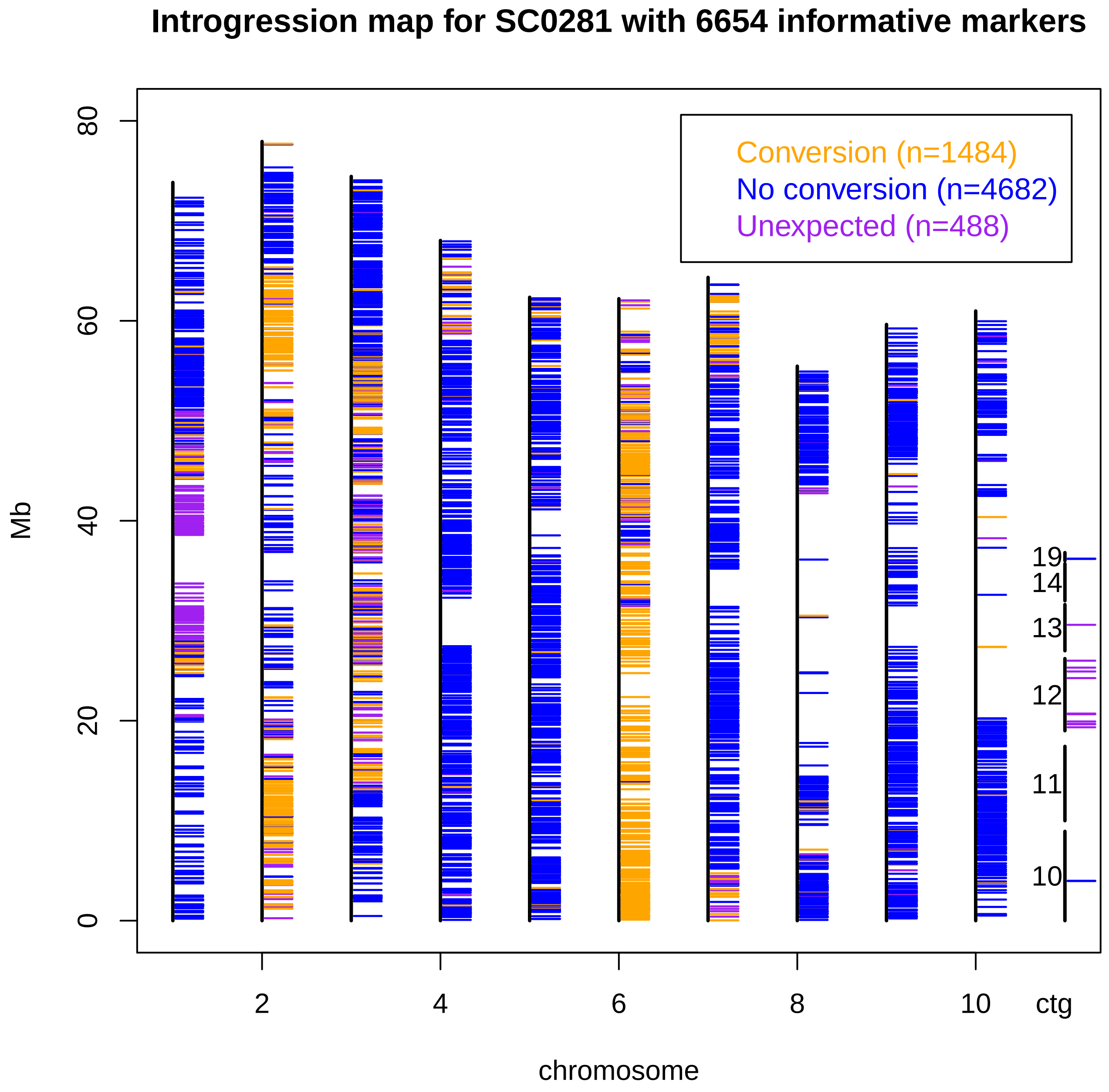

Introgression map for SC0281 with 6654 informative markers
Conversion (n=1484)
No conversion (n=4682)
Unexpected (n=488)
Mb
0
20
40
60
80
2
4
6
8
10
ctg
19
14
13
12
11
10
chromosome

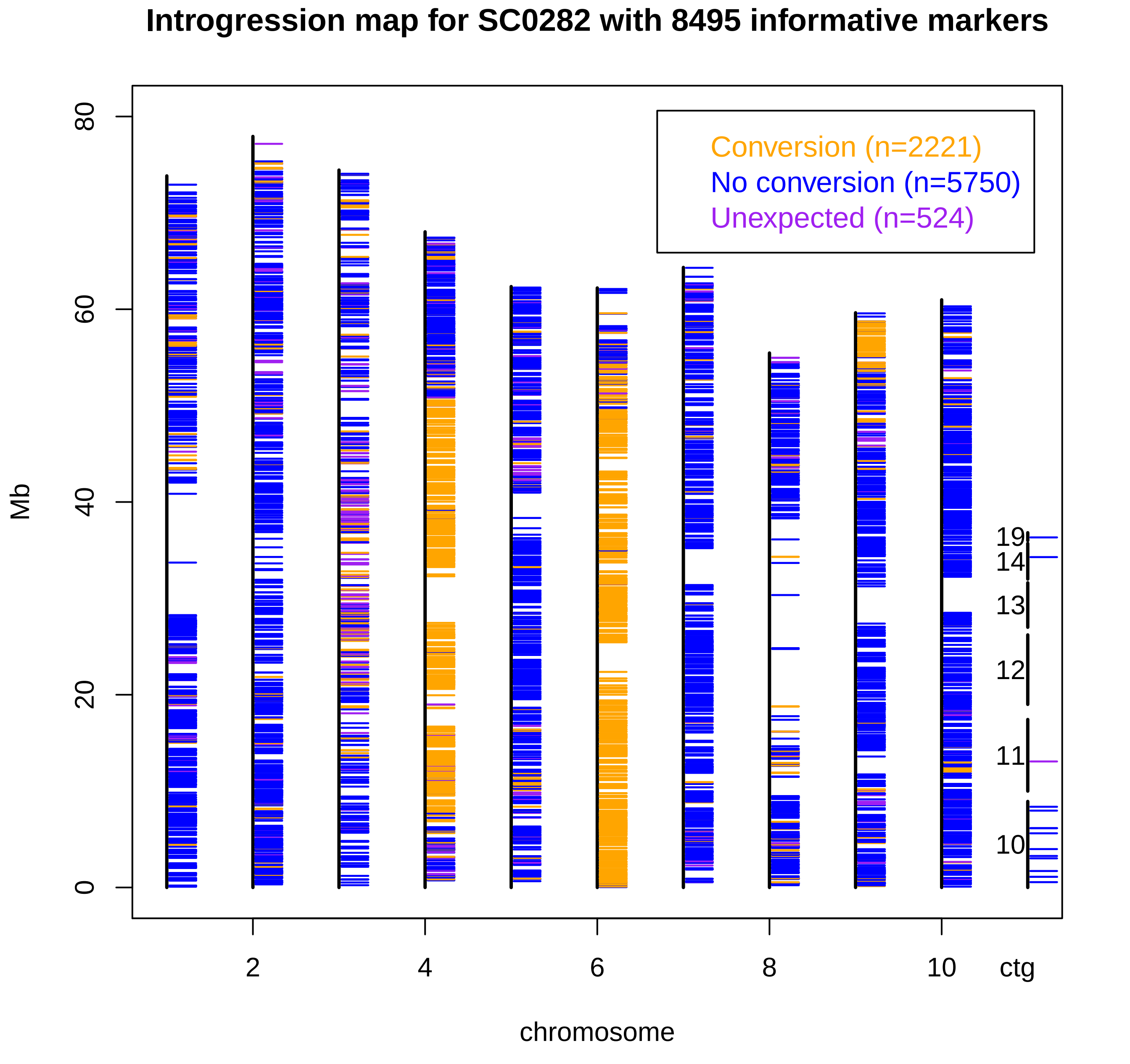

Introgression map for SC0282 with 8495 informative markers
Conversion (n=2221)
No conversion (n=5750)
Unexpected (n=524)
Mb
0
20
40
60
80
2
4
6
8
10
ctg
19
14
13
12
11
10
chromosome

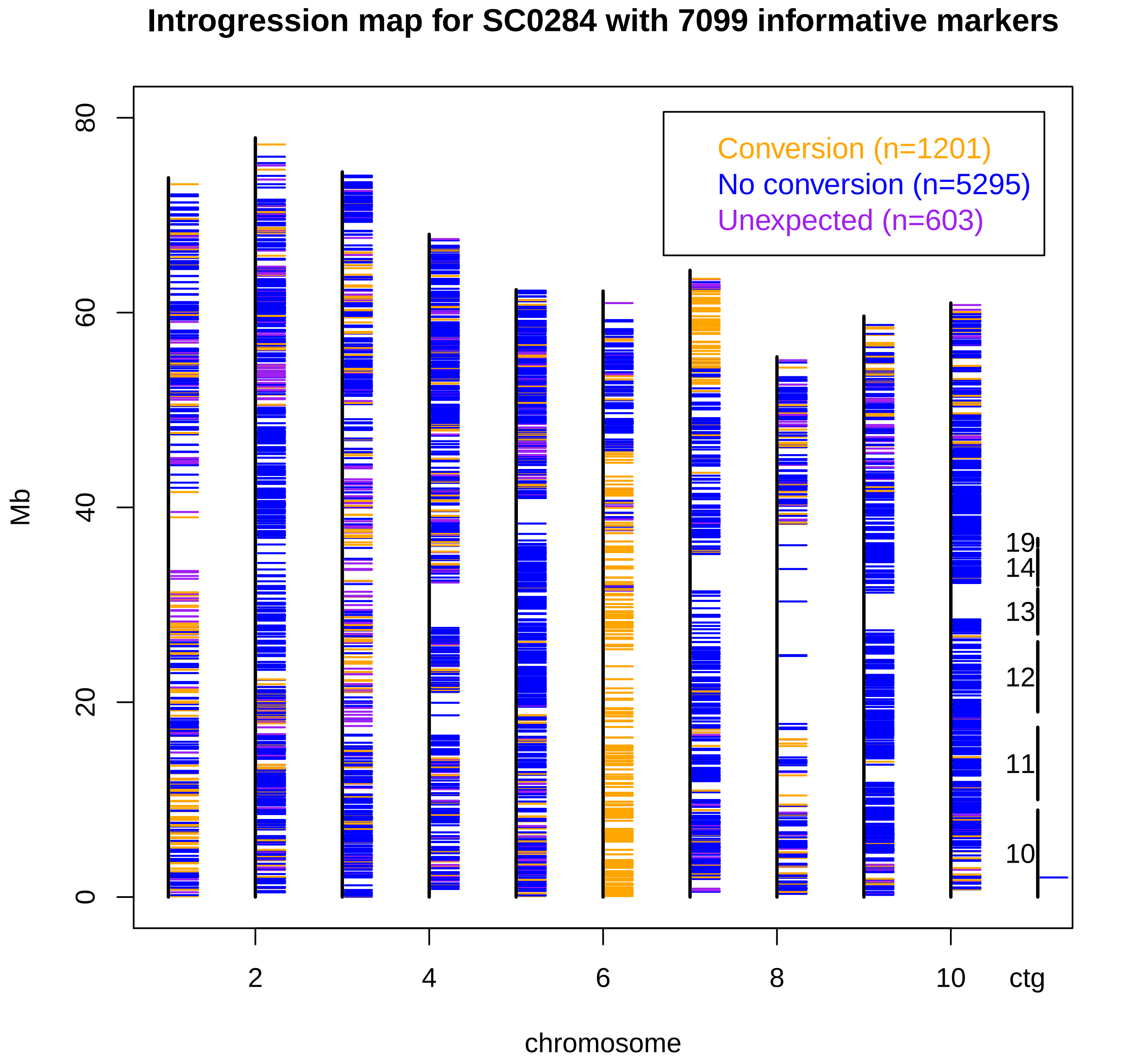
Introgression map for SC0284 with 7099 informative markers
Conversion (n=1201)
No conversion (n=5295)
Unexpected (n=603)
Mb
0
20
40
60
80
chromosome
2
4
6
8
10
ctg
19
14
13
12
11
10

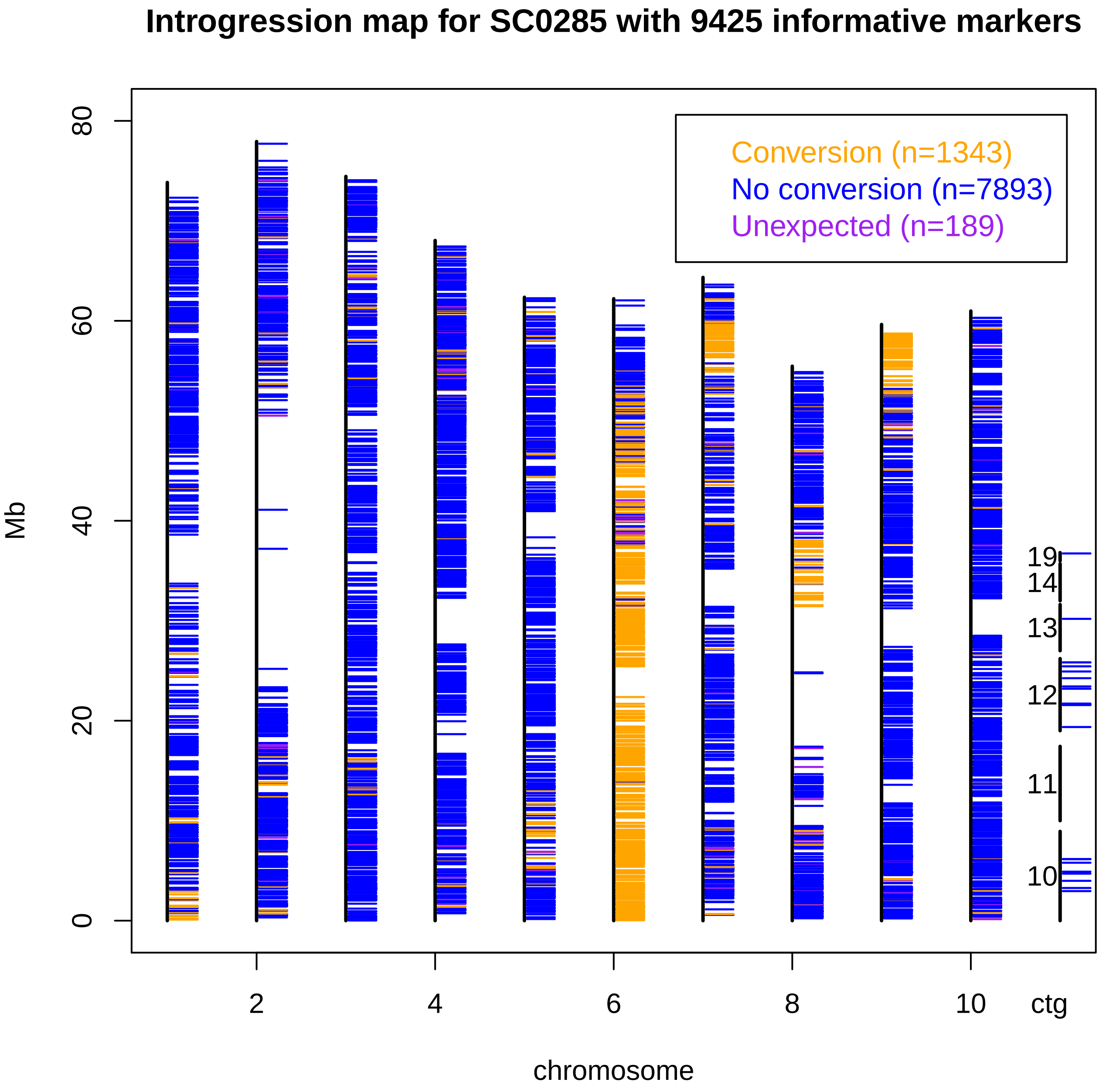

Introgression map for SC0285 with 9425 informative markers
Conversion (n=1343)
No conversion (n=7893)
Unexpected (n=189)
Mb
0
20
40
60
80
19
14
13
12
11
10
2
4
6
8
10
ctg
chromosome

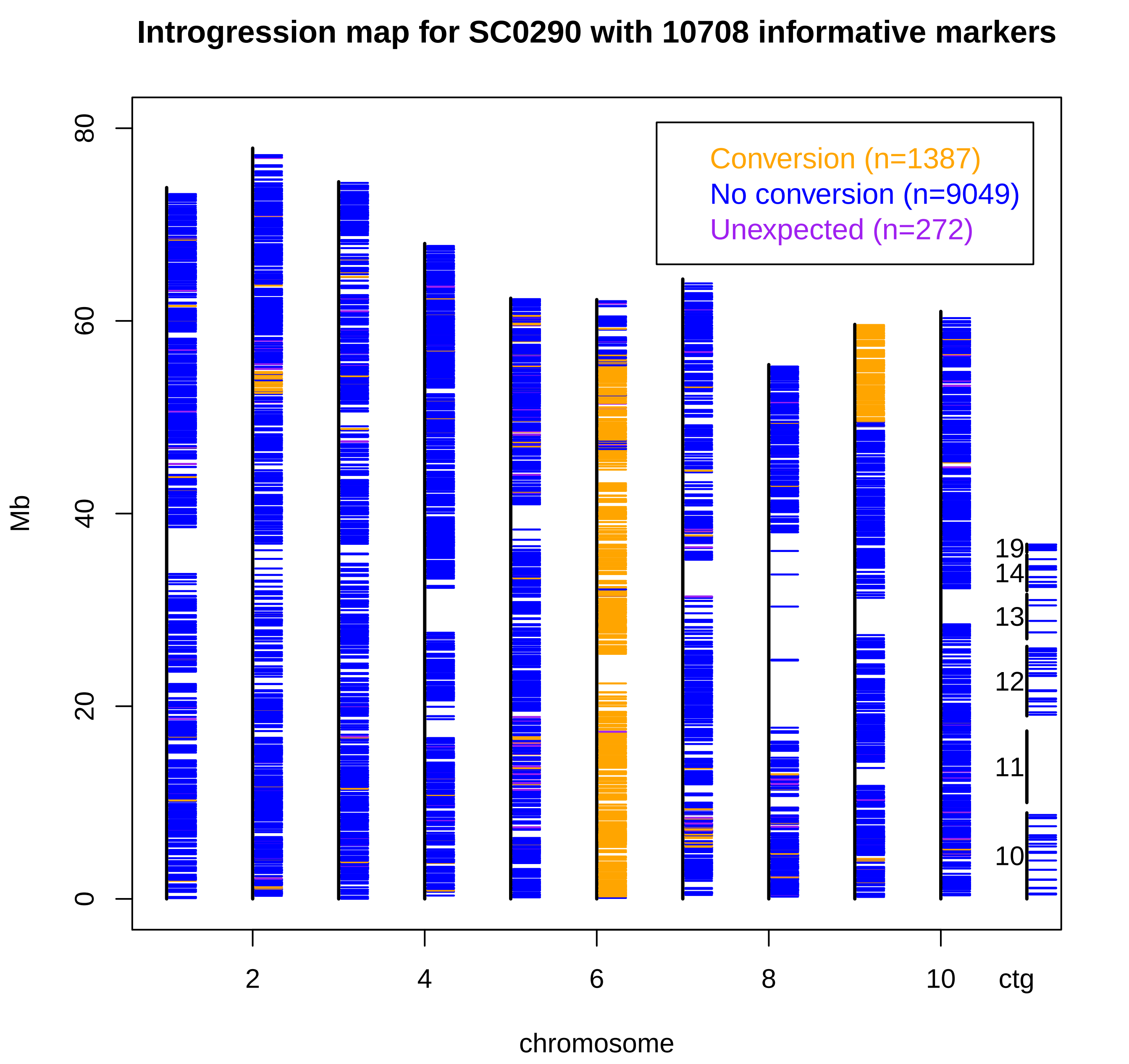

Introgression map for SC0290 with 10708 informative markers
Conversion (n=1387)
No conversion (n=9049)
Unexpected (n=272)
Mb
0
20
40
60
80
2
4
6
8
10
ctg
19
14
13
12
11
10
chromosome

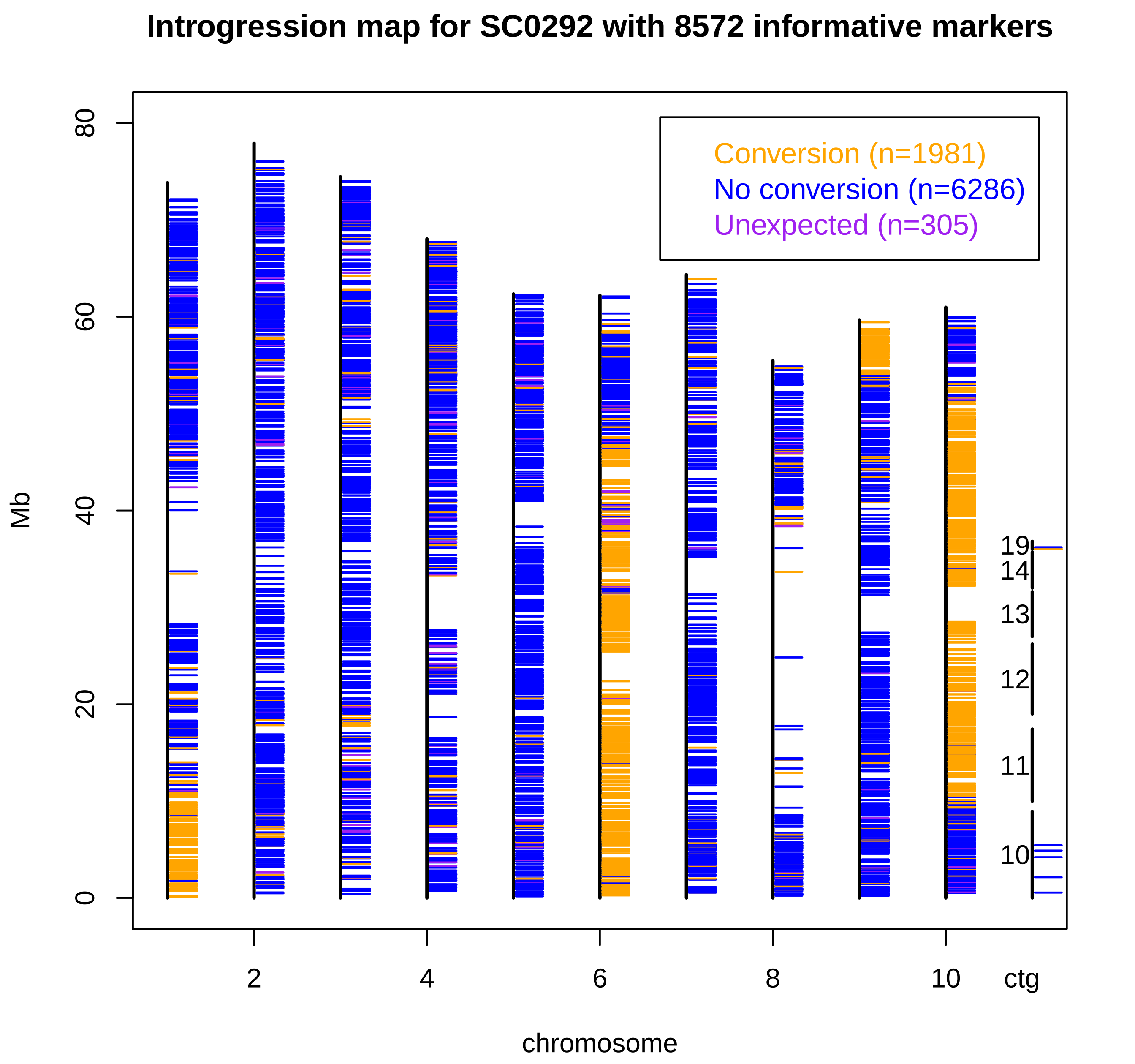
Introgression map for SC0292 with 8572 informative markers
Conversion (n=1981)
No conversion (n=6286)
Unexpected (n=305)
Mb
0
20
40
60
80
chromosome
2
4
6
8
10
ctg
19
14
13
12
11
10

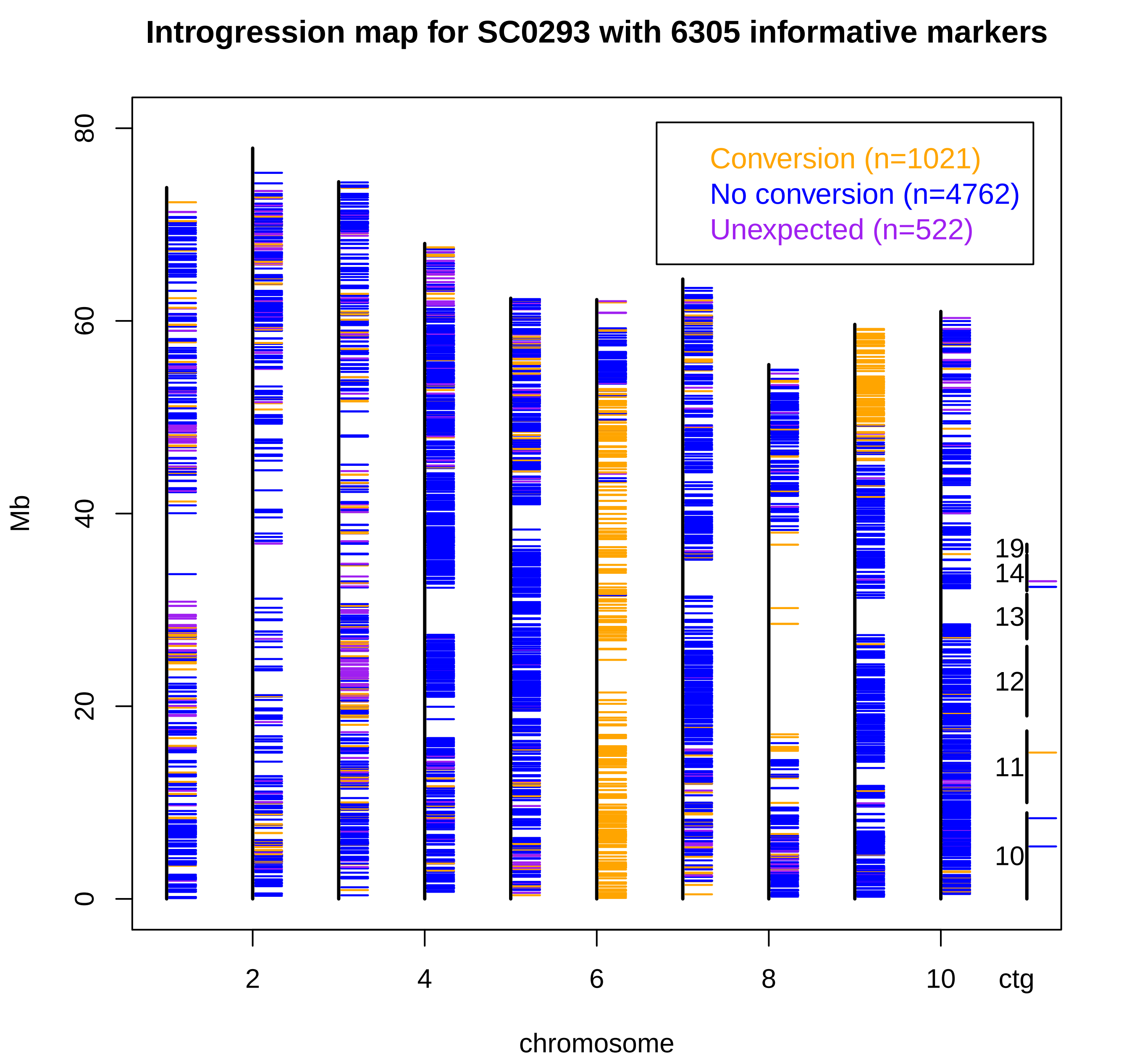
Introgression map for SC0293 with 6305 informative markers
Conversion (n=1021)
No conversion (n=4762)
Unexpected (n=522)
Mb
0
20
40
60
80
2
4
6
8
10
ctg
19
14
13
12
11
10
chromosome

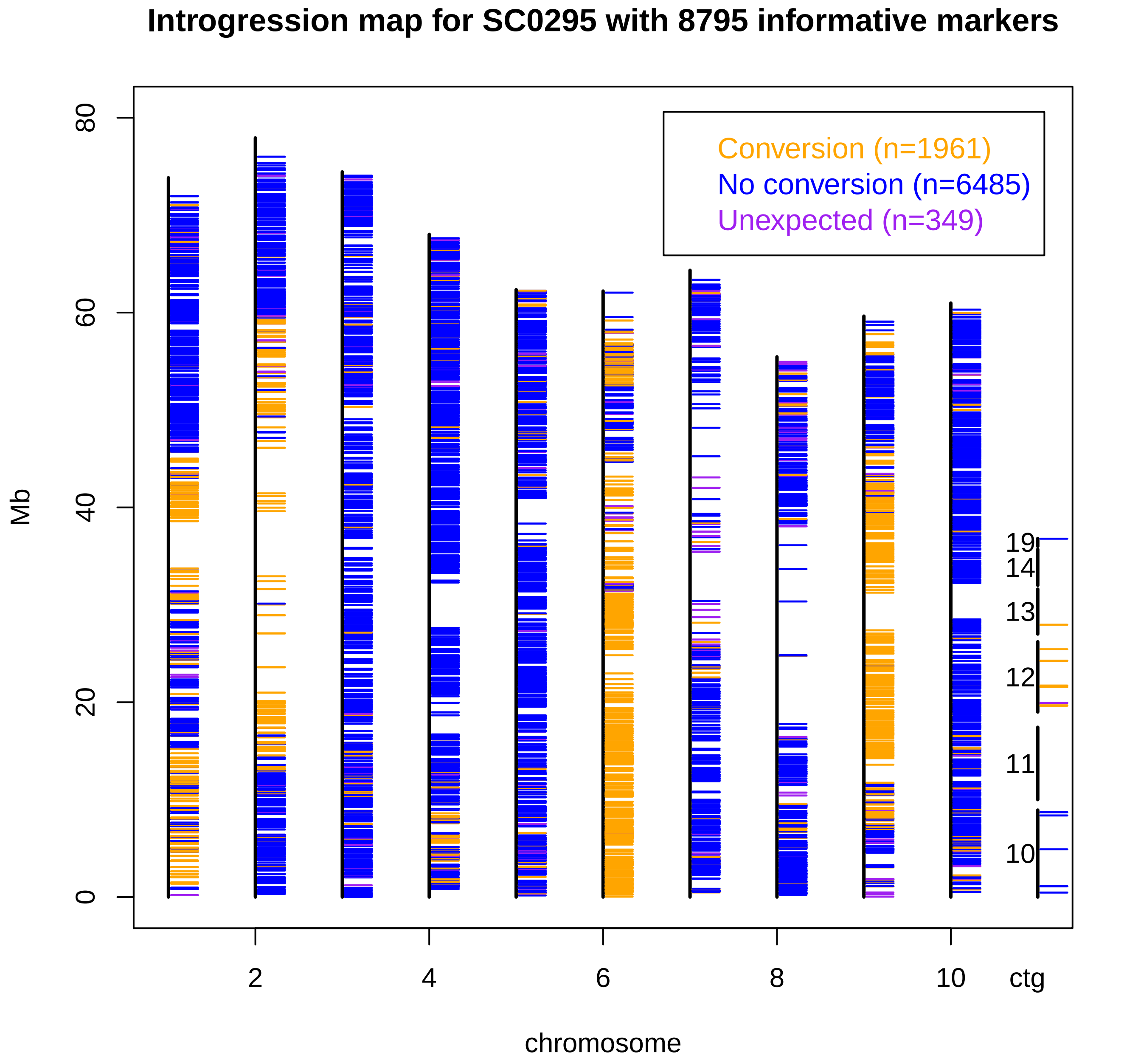
Introgression map for SC0295 with 8795 informative markers
Conversion (n=1961)
No conversion (n=6485)
Unexpected (n=349)
Mb
0
20
40
60
80
chromosome
2
4
6
8
10
ctg
19
14
13
12
11
10

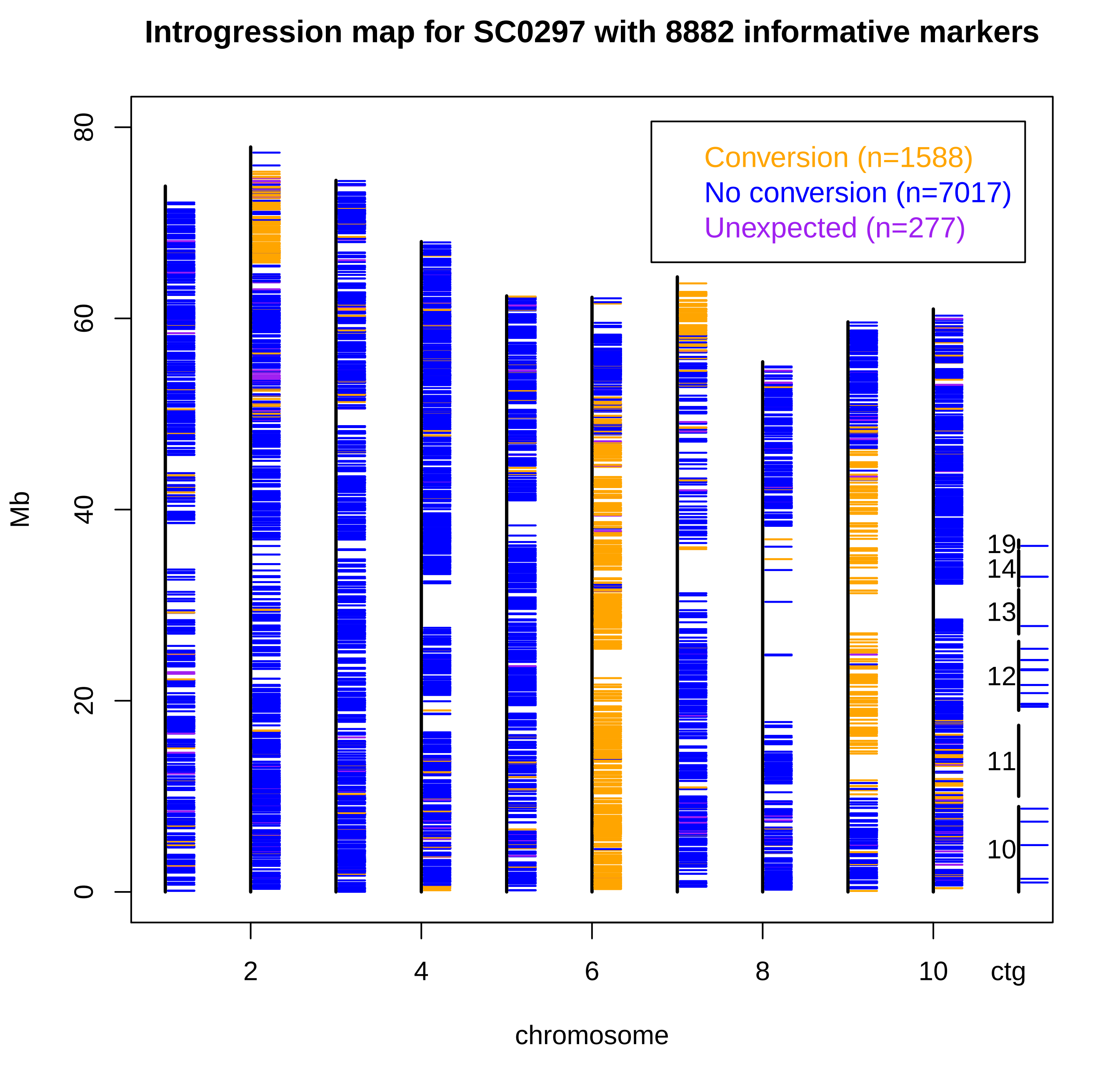
Introgression map for SC0297 with 8882 informative markers
Conversion (n=1588)
No conversion (n=7017)
Unexpected (n=277)
Mb
0
20
40
60
80
chromosome
2
4
6
8
10
ctg
19
14
13
12
11
10

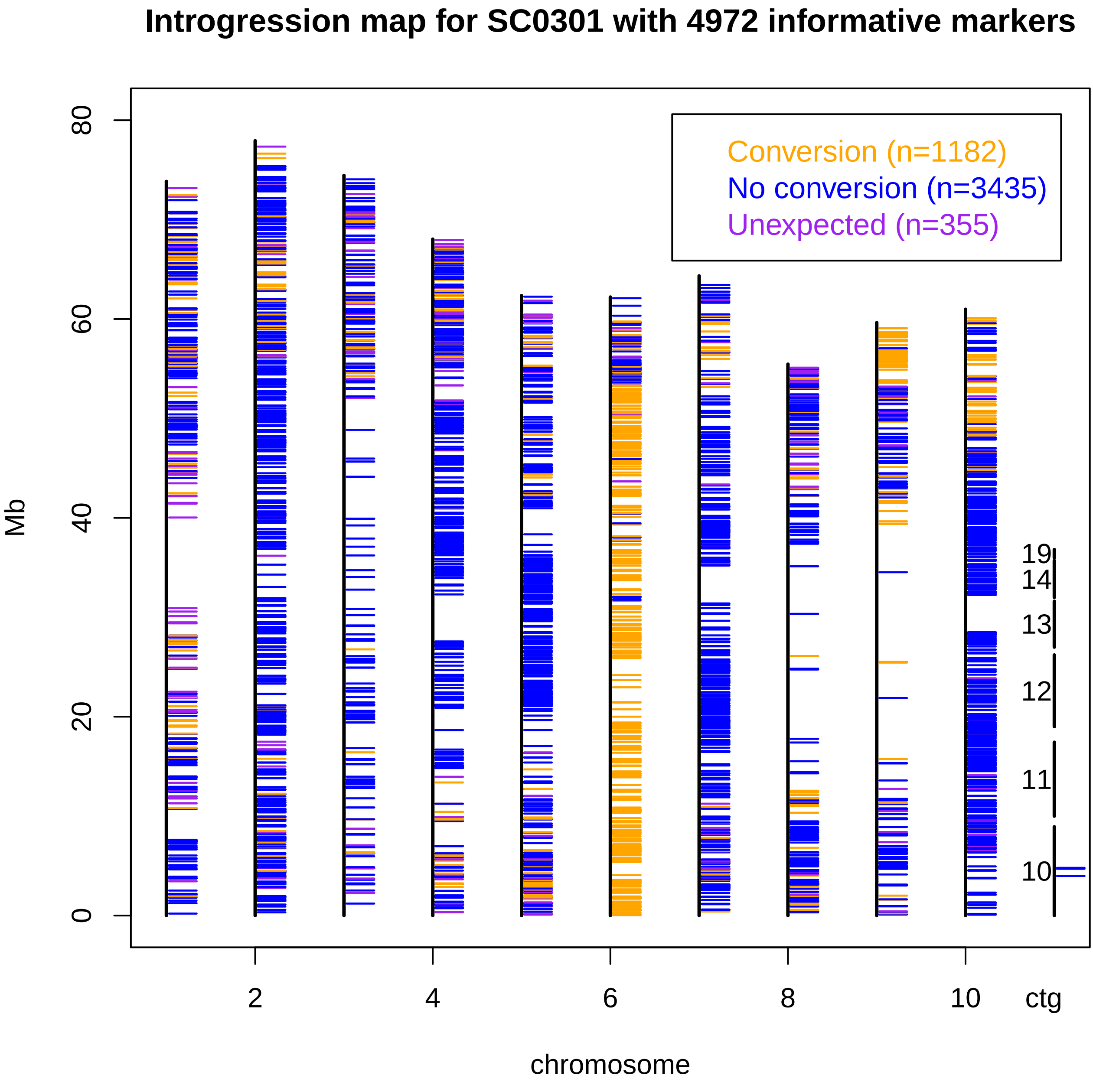
Introgression map for SC0301 with 4972 informative markers
Conversion (n=1182)
No conversion (n=3435)
Unexpected (n=355)
Mb
0
20
40
60
80
2
4
6
8
10
ctg
chromosome
19
14
13
12
11
10

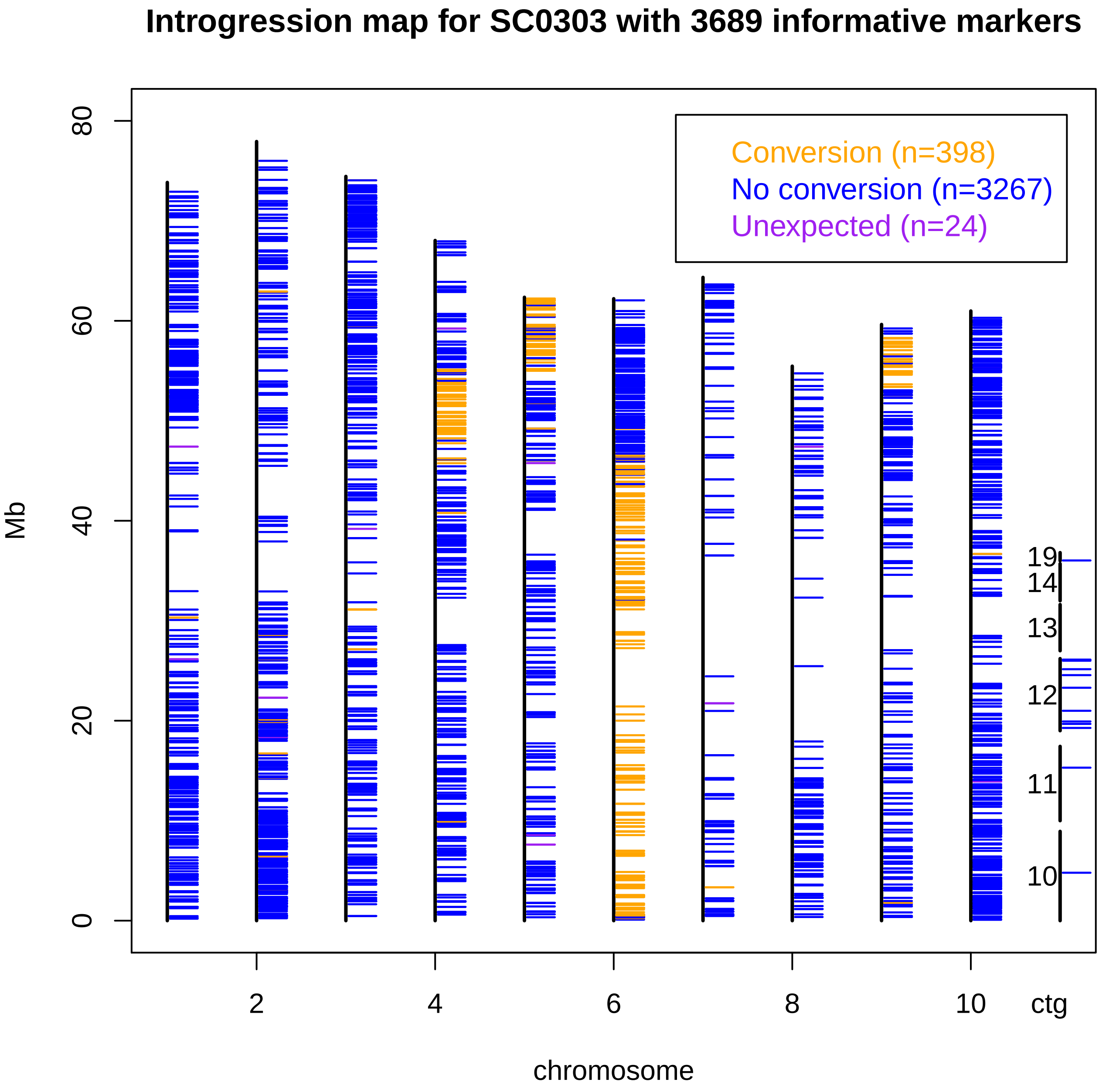
Introgression map for SC0303 with 3689 informative markers
Conversion (n=398)
No conversion (n=3267)
Unexpected (n=24)
Mb
0
20
40
60
80
chromosome
2
4
6
8
10
ctg
19
14
13
12
11
10

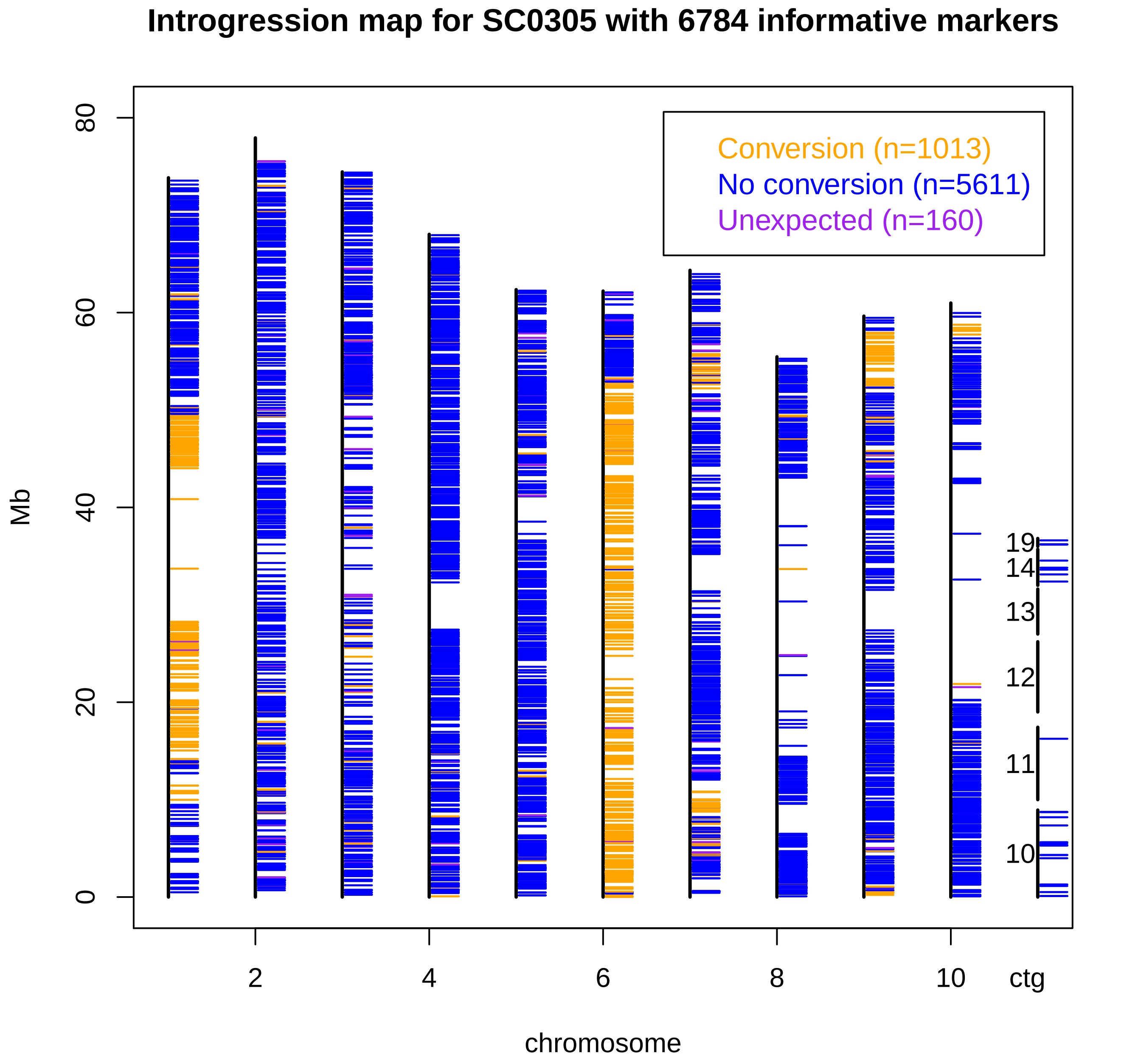

Introgression map for SC0305 with 6784 informative markers
Conversion (n=1013)
No conversion (n=5611)
Unexpected (n=160)
Mb
0
20
40
60
80
2
4
6
8
10
ctg
19
14
13
12
11
10
chromosome

Introgression map for SC0306 with 9821 informative markers

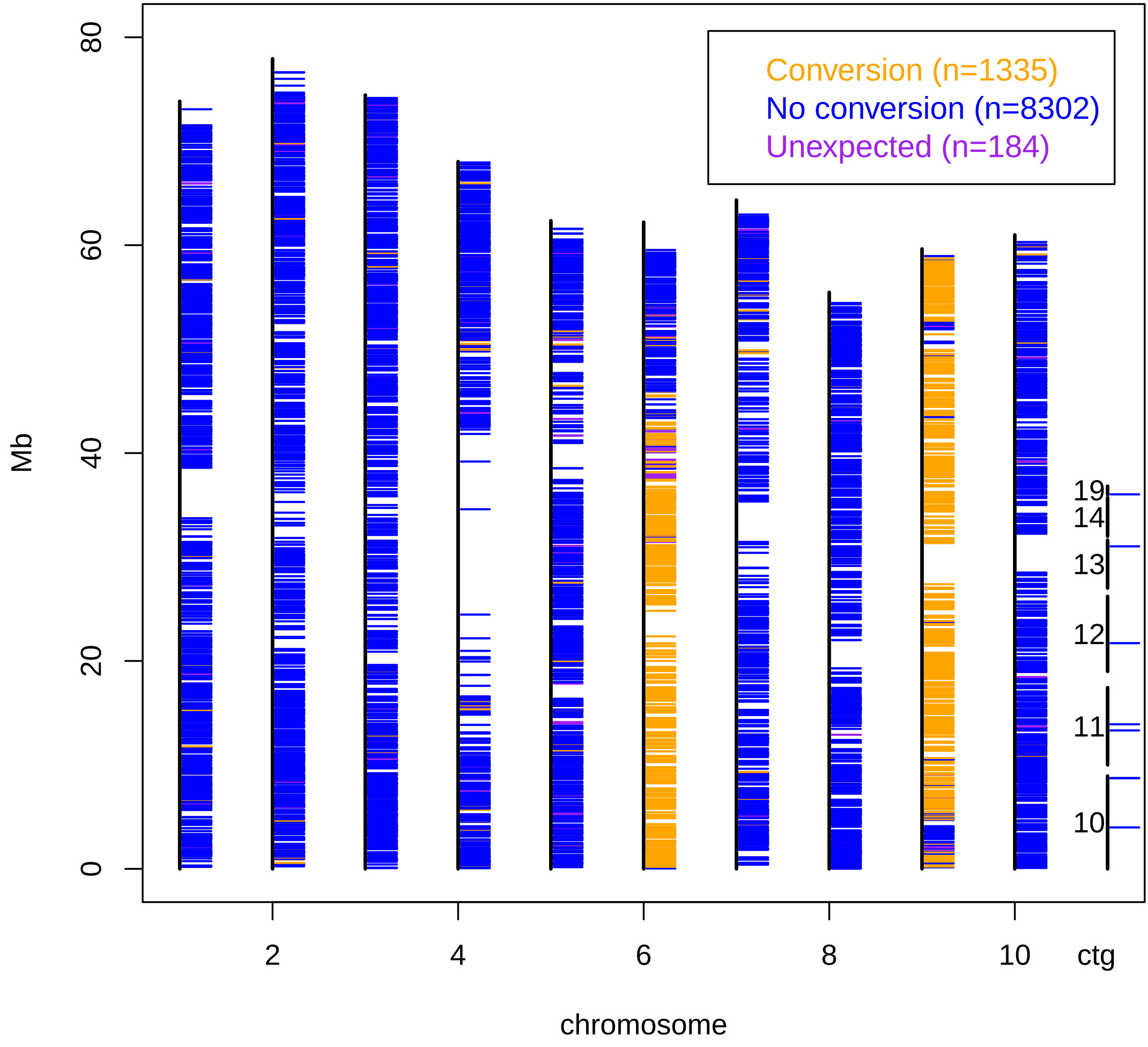

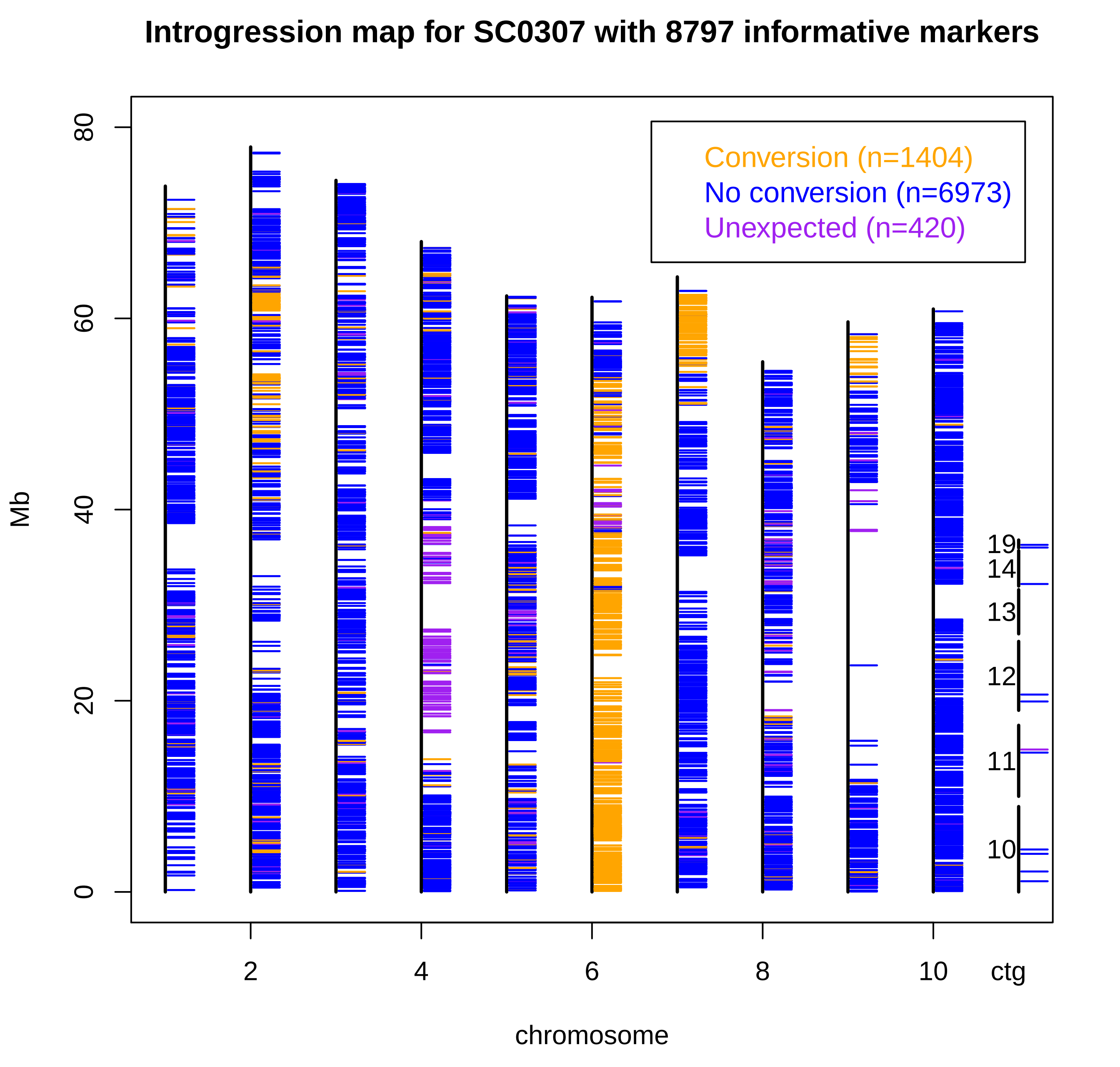

Introgression map for SC0307 with 8797 informative markers
Conversion (n=1404)
No conversion (n=6973)
Unexpected (n=420)
Mb
0
20
40
60
80
chromosome
2
4
6
8
10
ctg
19
14
13
12
11
10

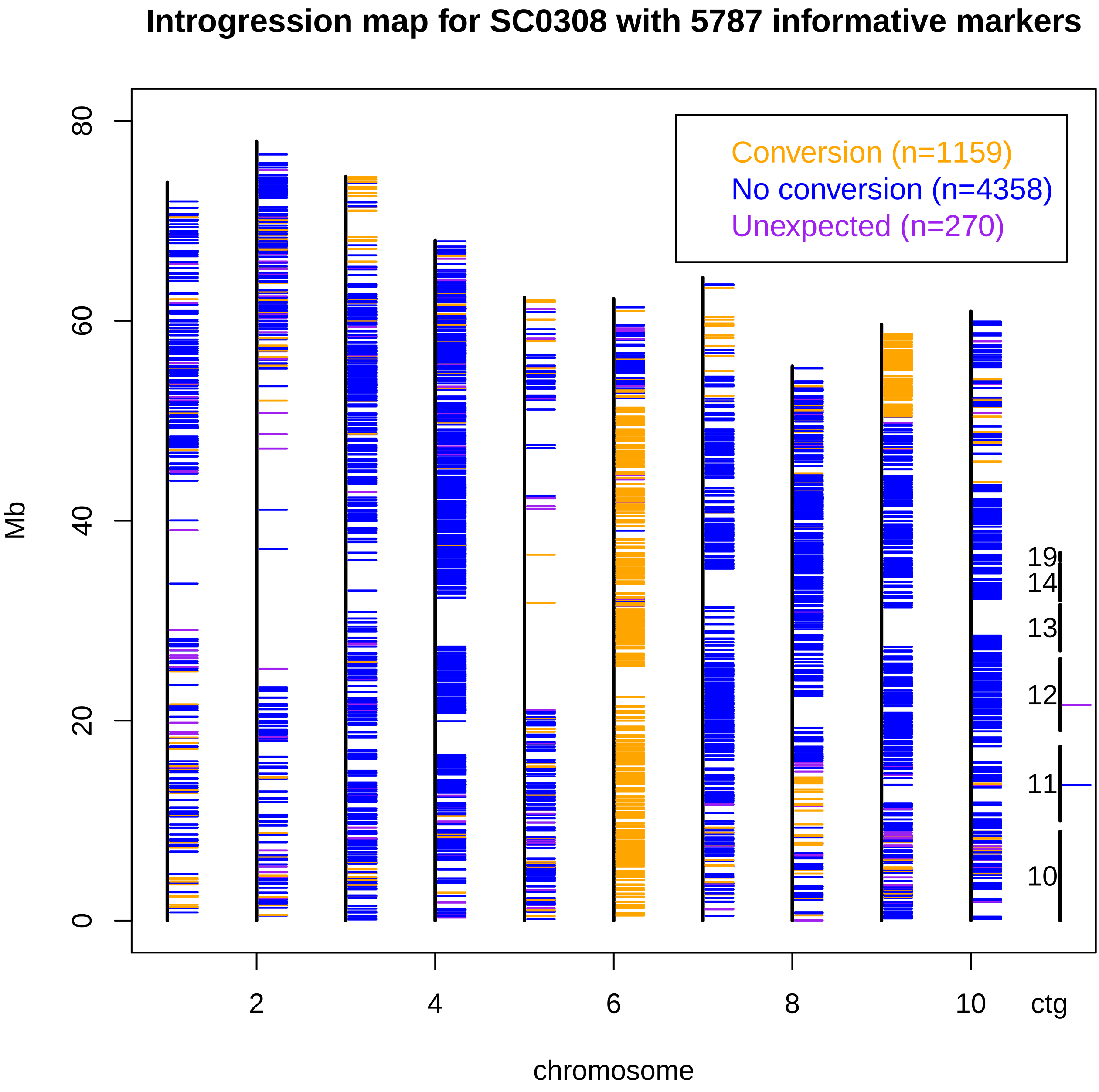

Introgression map for SC0308 with 5787 informative markers
Conversion (n=1159)
No conversion (n=4358)
Unexpected (n=270)
Mb
0
20
40
60
80
chromosome
2
4
6
8
10
ctg
19
14
13
12
11
10

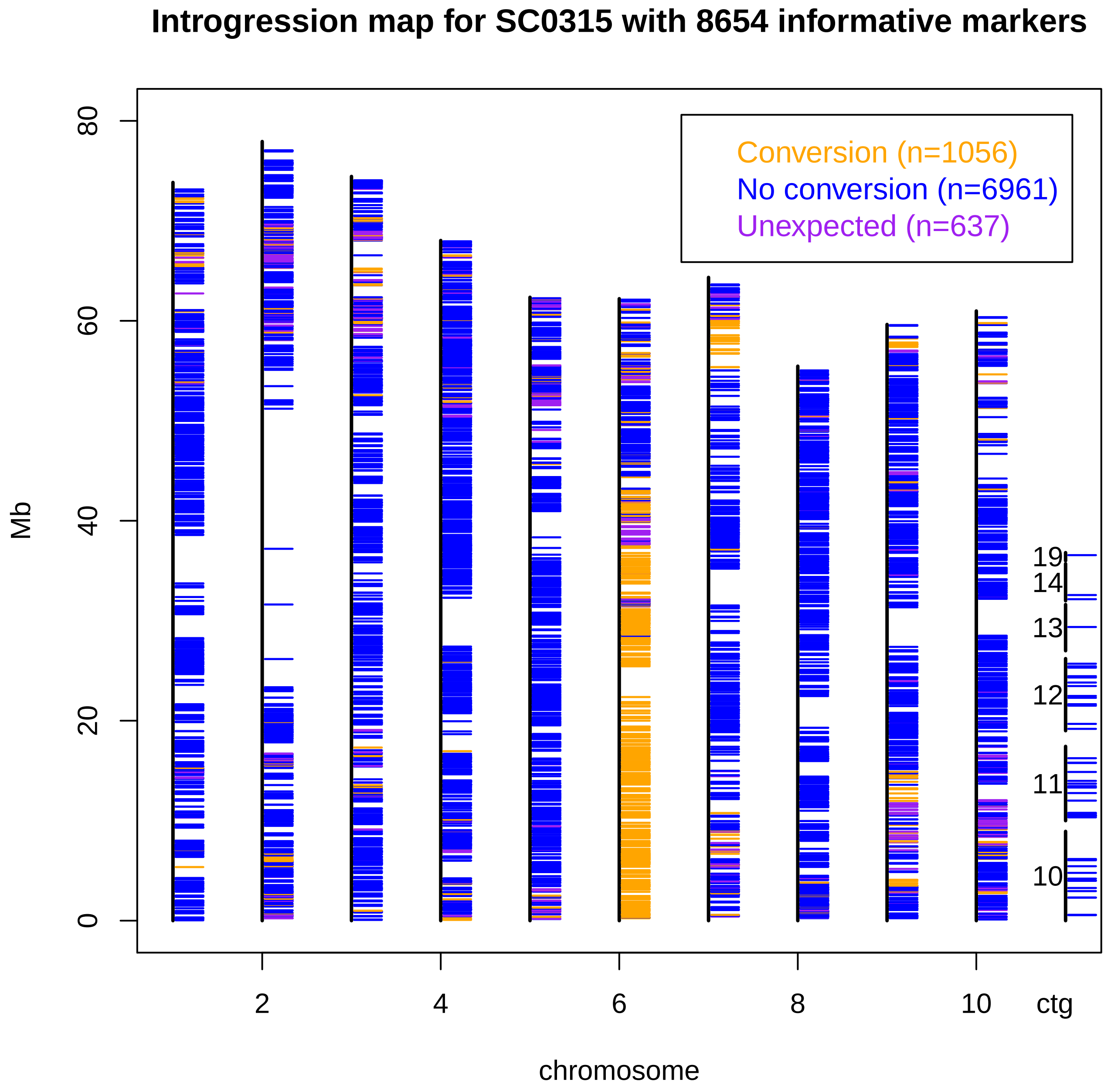

Introgression map for SC0315 with 8654 informative markers
Conversion (n=1056)
No conversion (n=6961)
Unexpected (n=637)
Mb
0
20
40
60
80
2
4
6
8
10
ctg
19
14
13
12
11
10
chromosome

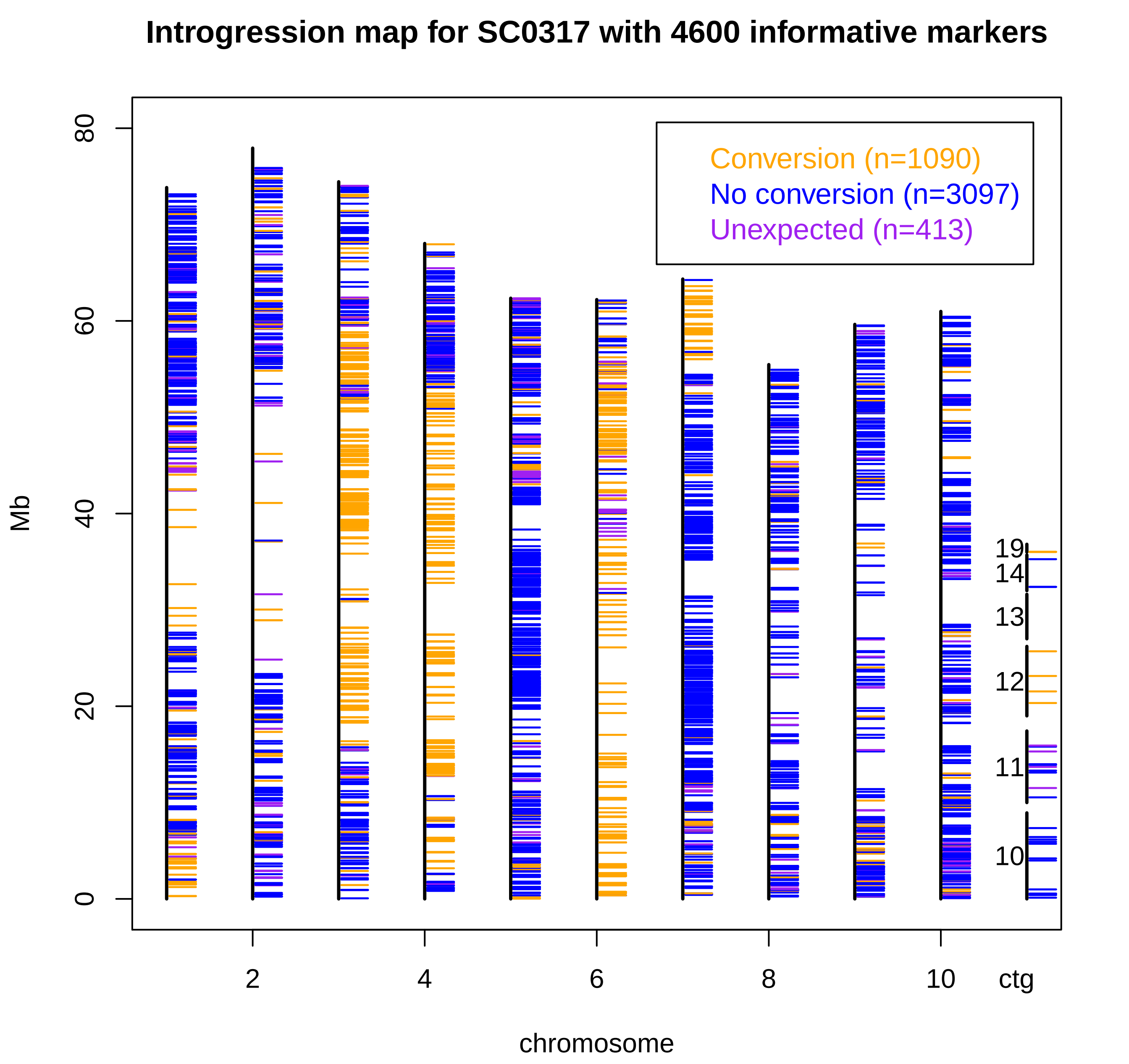

Introgression map for SC0317 with 4600 informative markers
Conversion (n=1090)
No conversion (n=3097)
Unexpected (n=413)
Mb
0
20
40
60
80
chromosome
2
4
6
8
10
ctg
19
14
13
12
11
10

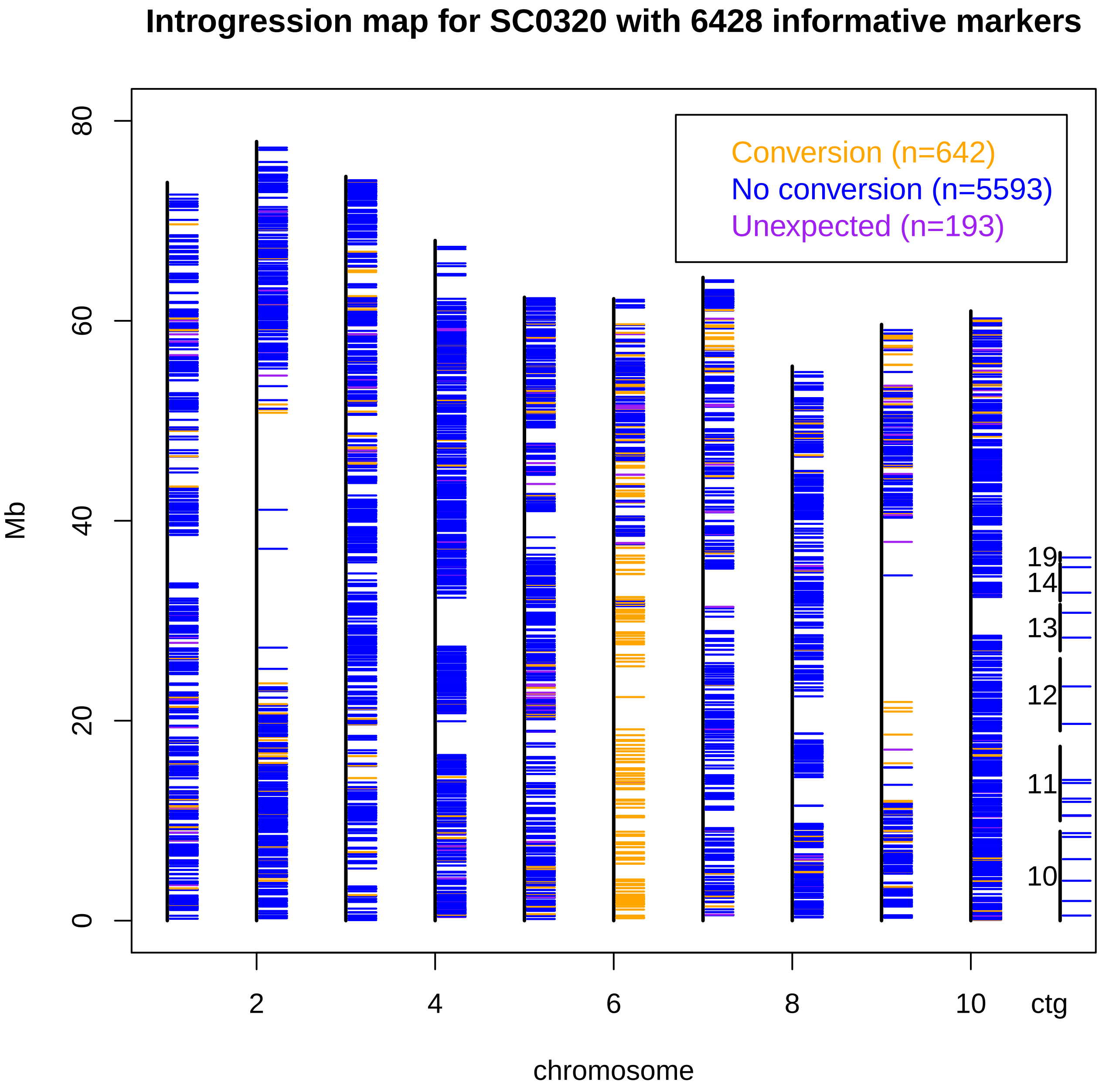

Introgression map for SC0320 with 6428 informative markers
Conversion (n=642)
No conversion (n=5593)
Unexpected (n=193)
Mb
0
20
40
60
80
2
4
6
8
10
ctg
19
14
13
12
11
10
chromosome

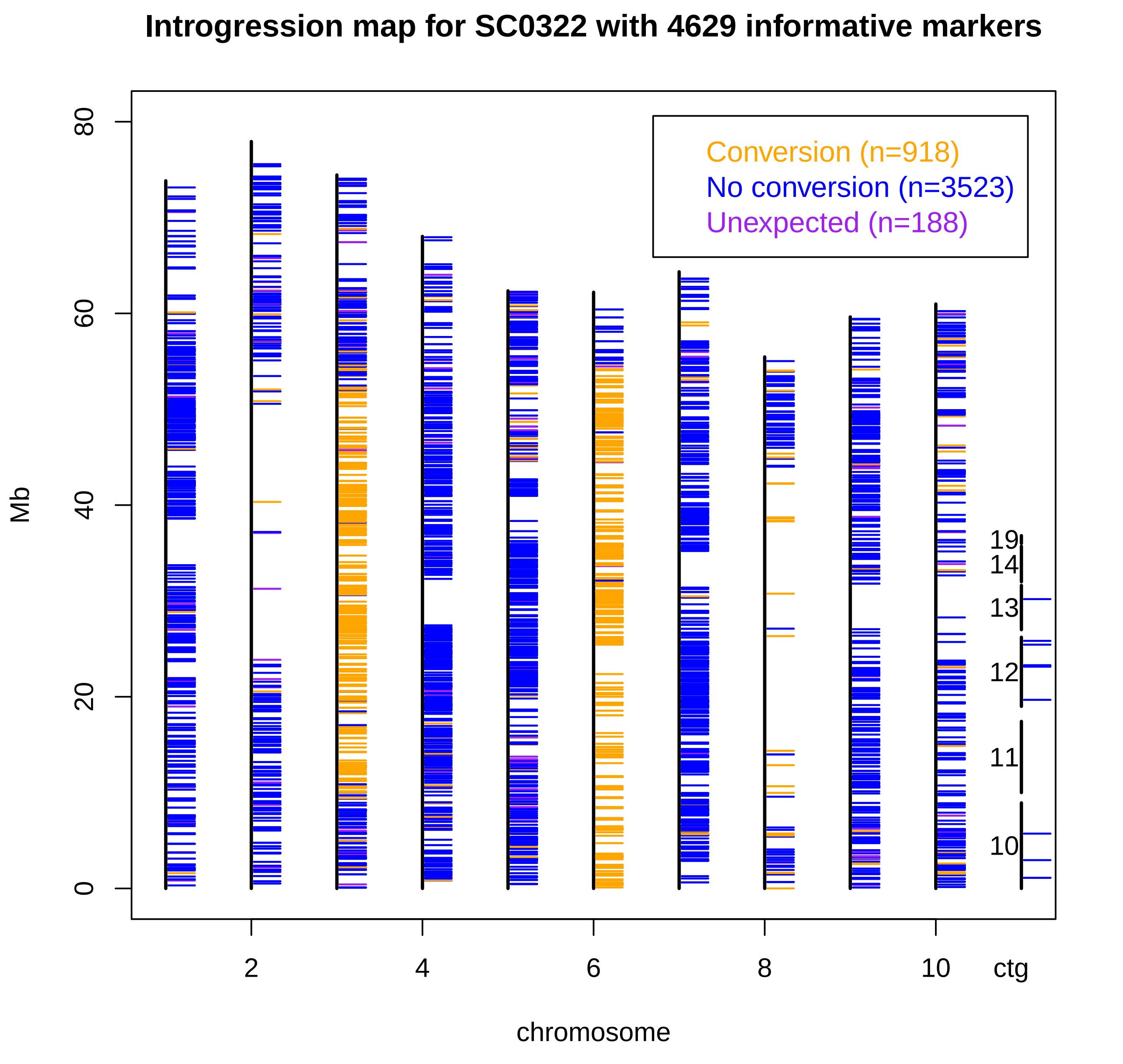

Introgression map for SC0322 with 4629 informative markers
Conversion (n=918)
No conversion (n=3523)
Unexpected (n=188)
Mb
0
20
40
60
80
2
4
6
8
10
ctg
19
14
13
12
11
10
chromosome

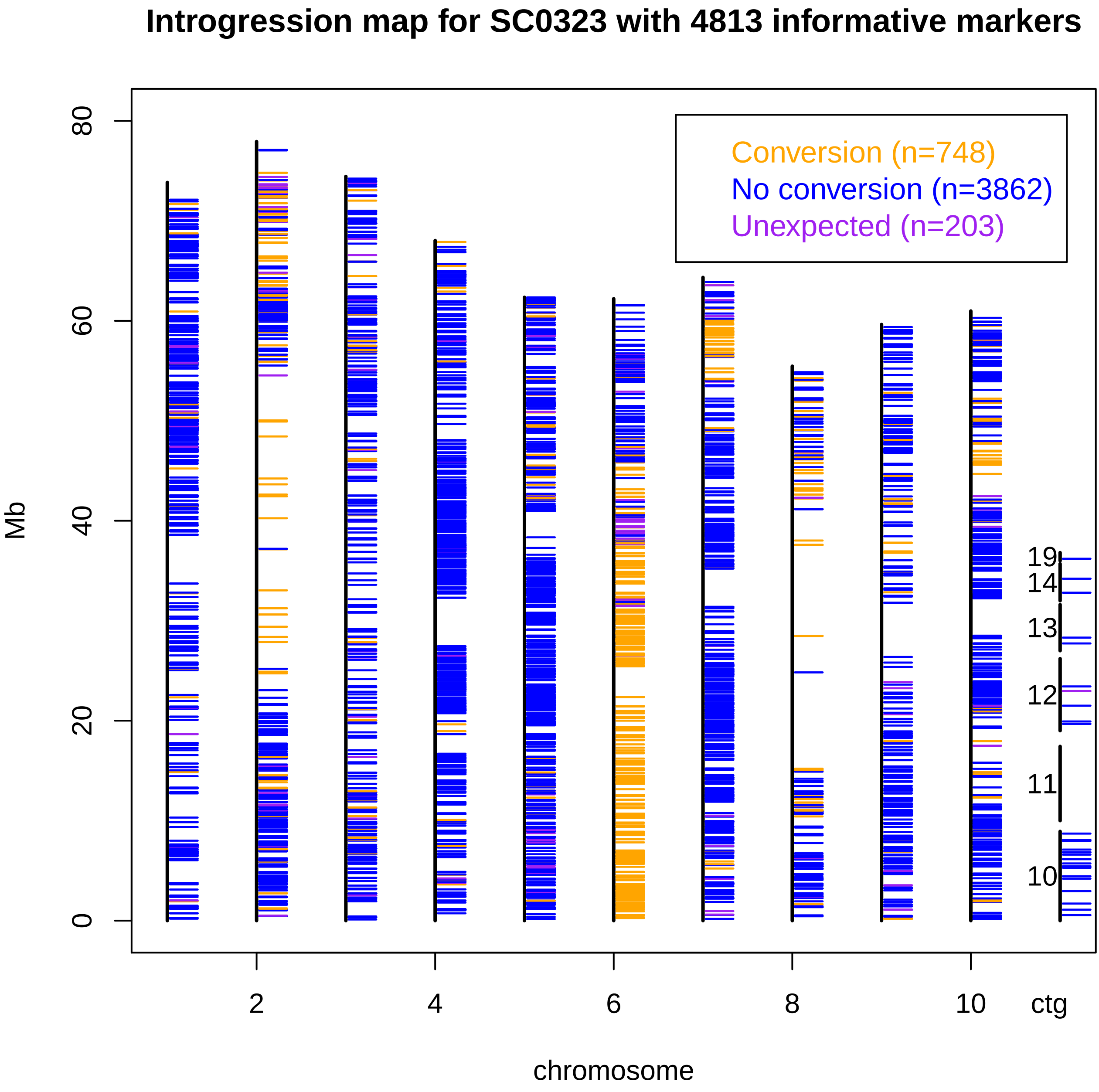

Introgression map for SC0323 with 4813 informative markers
Conversion (n=748)
No conversion (n=3862)
Unexpected (n=203)
Mb
0
20
40
60
80
chromosome
2
4
6
8
10
ctg
19
14
13
12
11
10

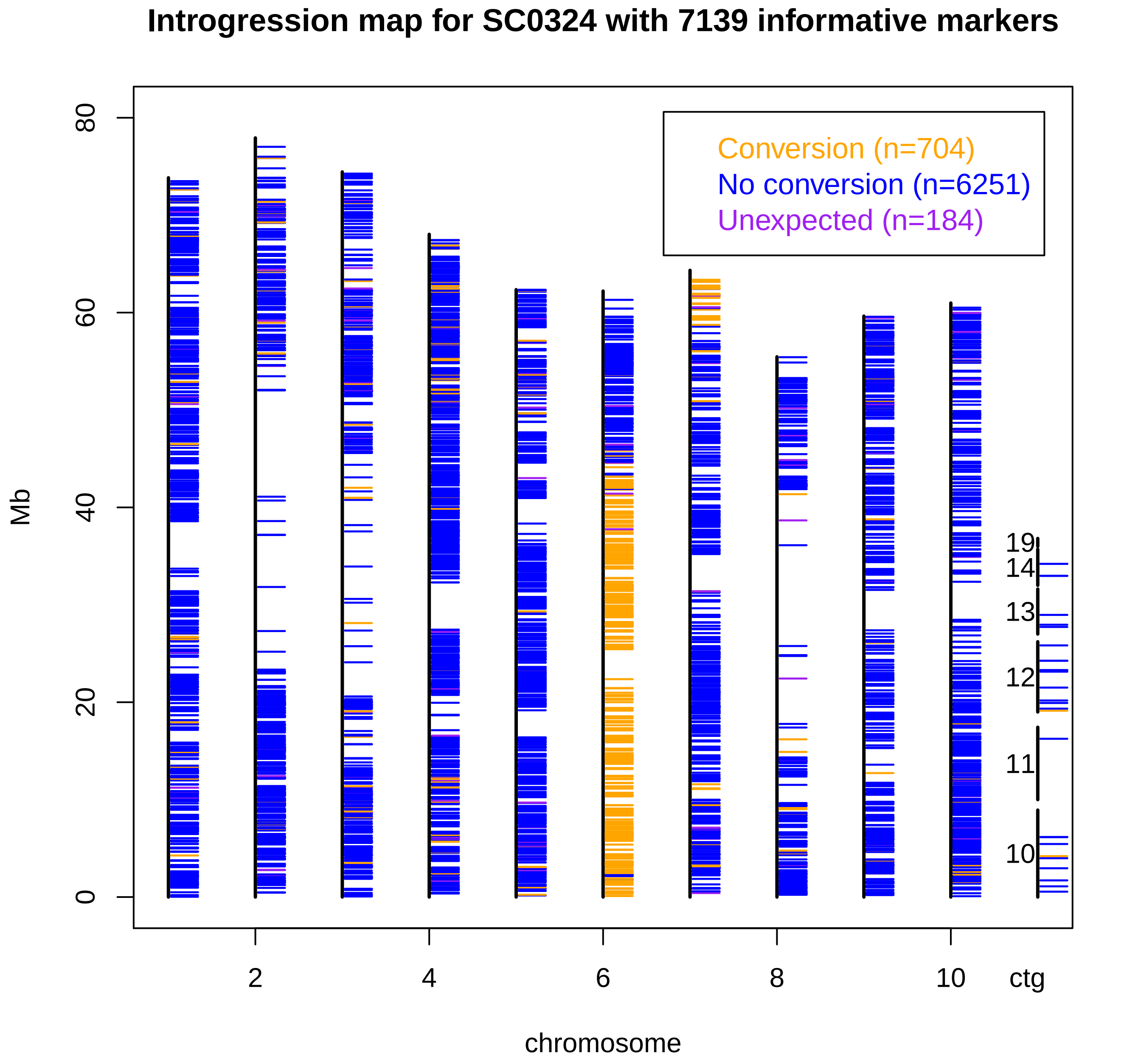

Introgression map for SC0324 with 7139 informative markers
Conversion (n=704)
No conversion (n=6251)
Unexpected (n=184)
Mb
0
20
40
60
80
2
4
6
8
10
ctg
chromosome
19
14
13
12
11
10

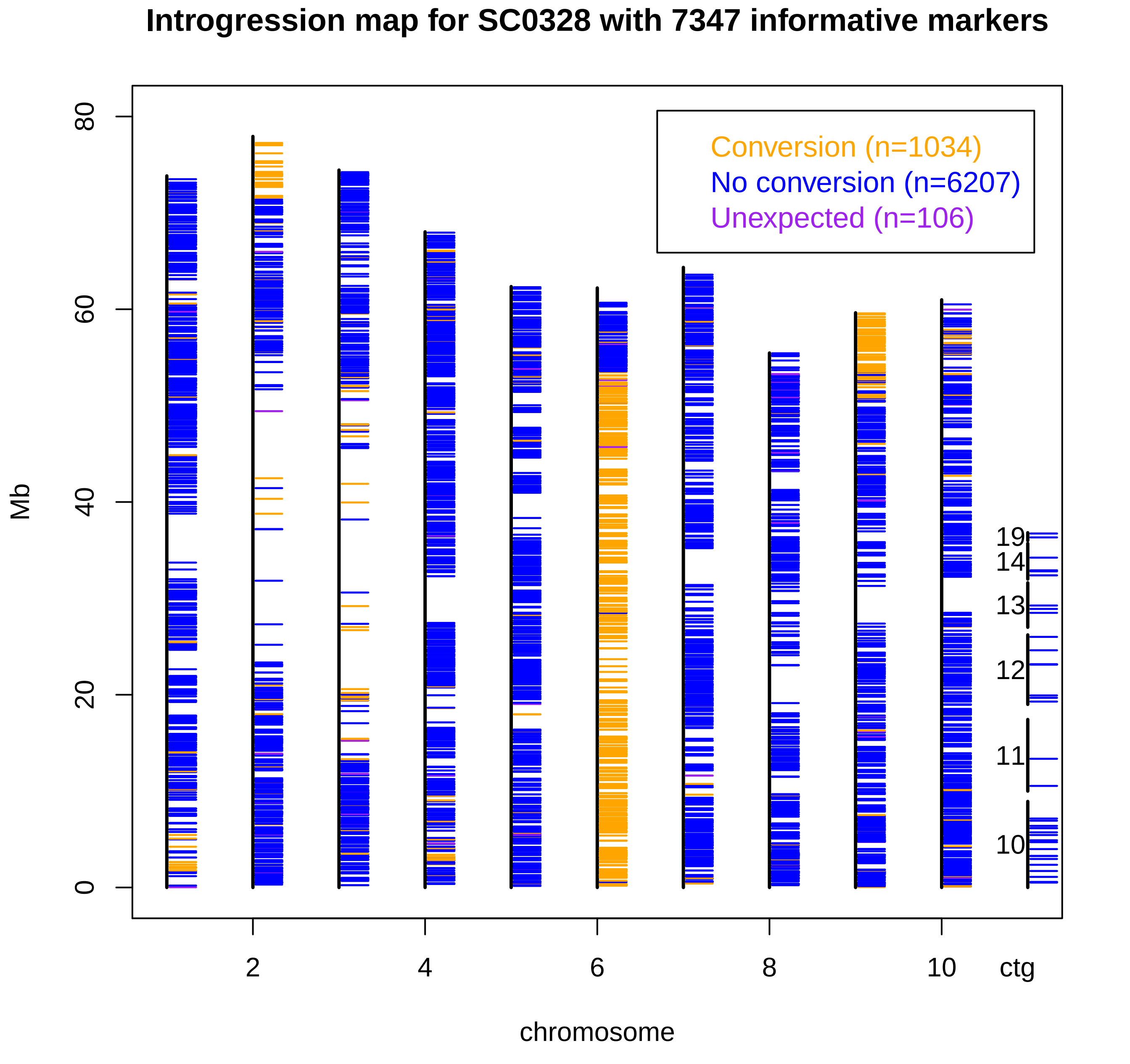

Introgression map for SC0328 with 7347 informative markers
Conversion (n=1034)
No conversion (n=6207)
Unexpected (n=106)
Mb
0
20
40
60
80
chromosome
2
4
6
8
10
ctg
19
14
13
12
11
10

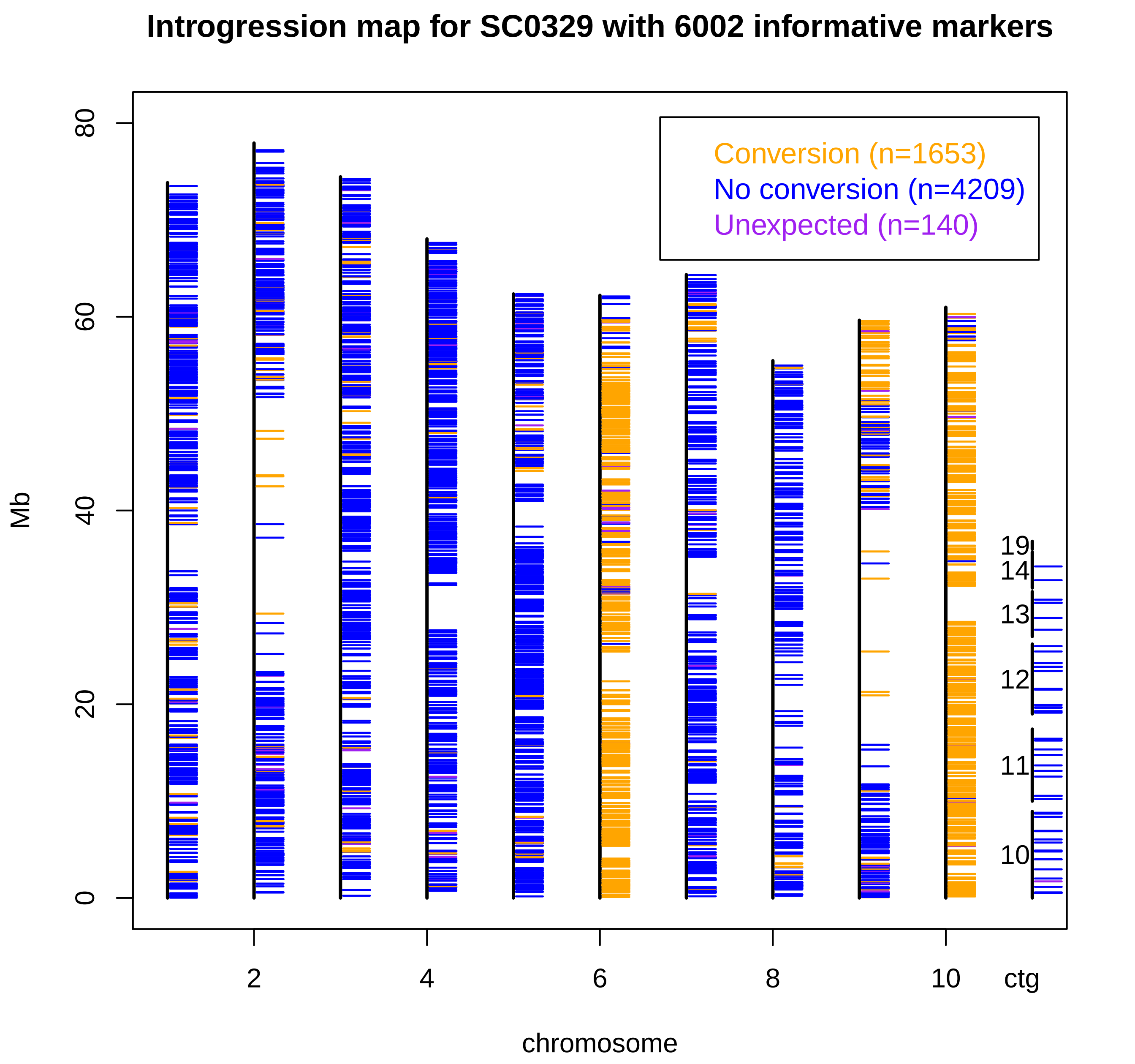

Introgression map for SC0329 with 6002 informative markers
Conversion (n=1653)
No conversion (n=4209)
Unexpected (n=140)
Mb
0
20
40
60
80
2
4
6
8
10
ctg
19
14
13
12
11
10
chromosome

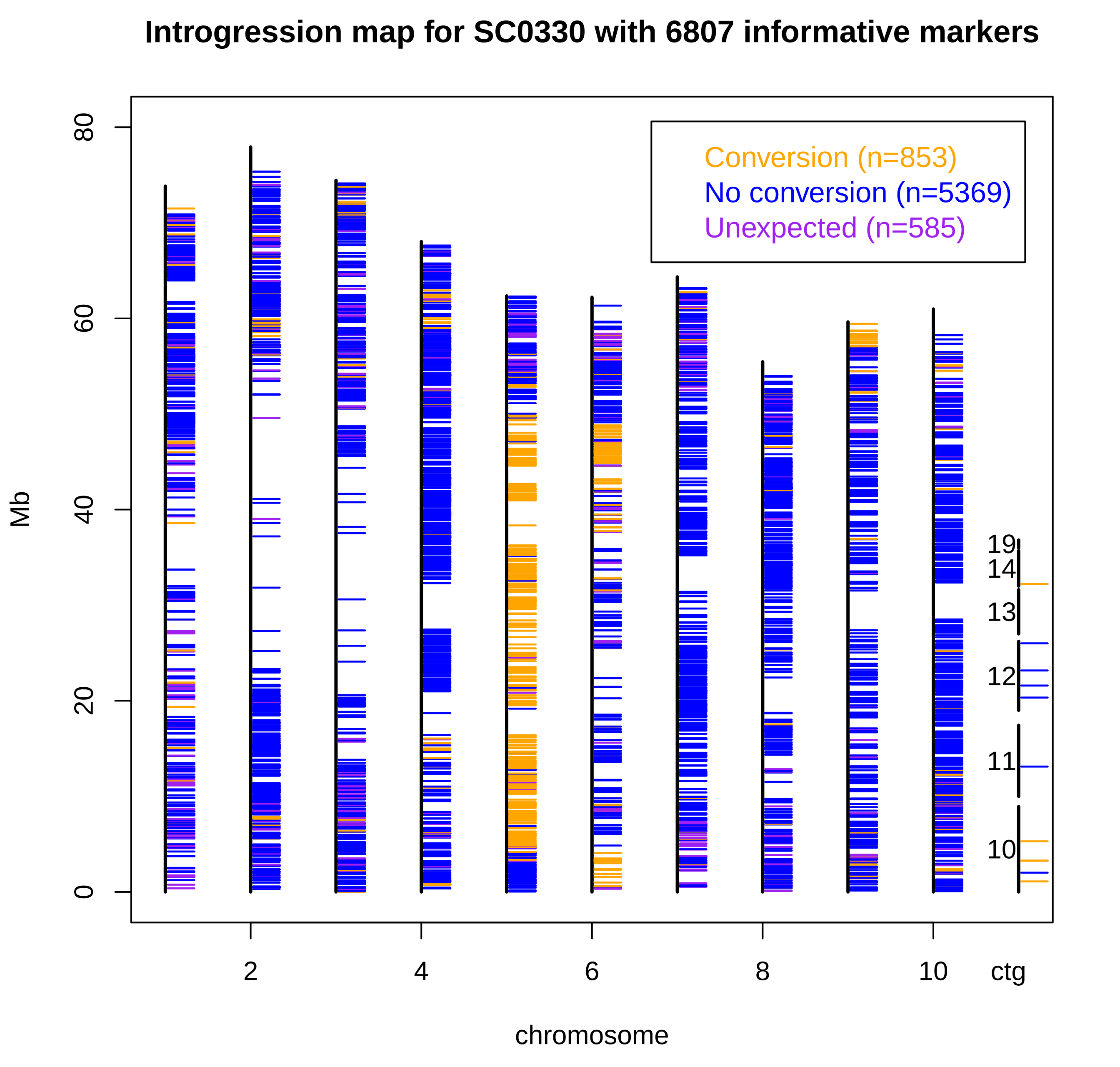
Introgression map for SC0330 with 6807 informative markers
Conversion (n=853)
No conversion (n=5369)
Unexpected (n=585)
Mb
0
20
40
60
80
chromosome
2
4
6
8
10
ctg
19
14
13
12
11
10

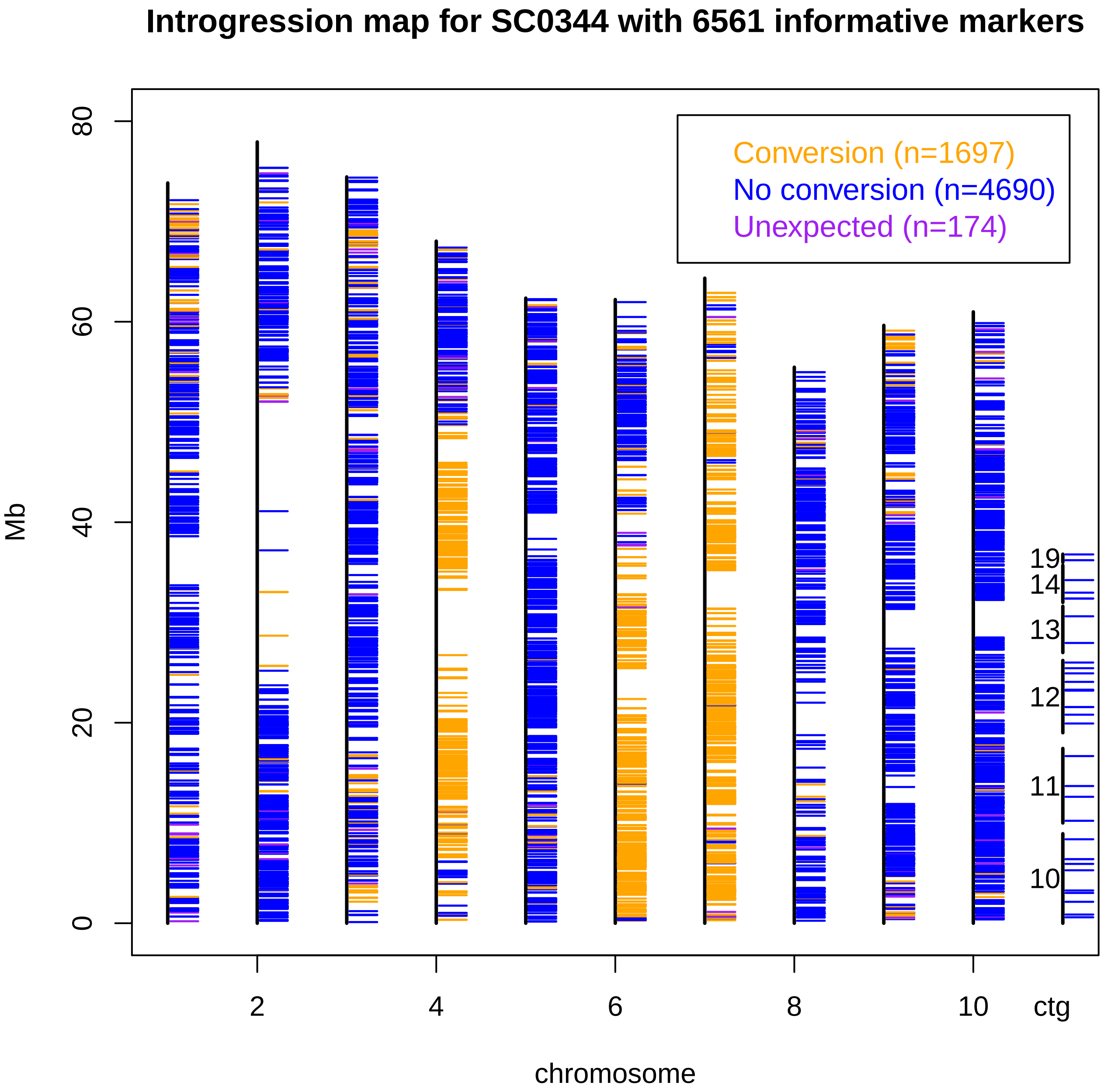

Introgression map for SC0344 with 6561 informative markers
Conversion (n=1697)
No conversion (n=4690)
Unexpected (n=174)
Mb
0
20
40
60
80
chromosome
2
4
6
8
10
ctg
19
14
13
12
11
10

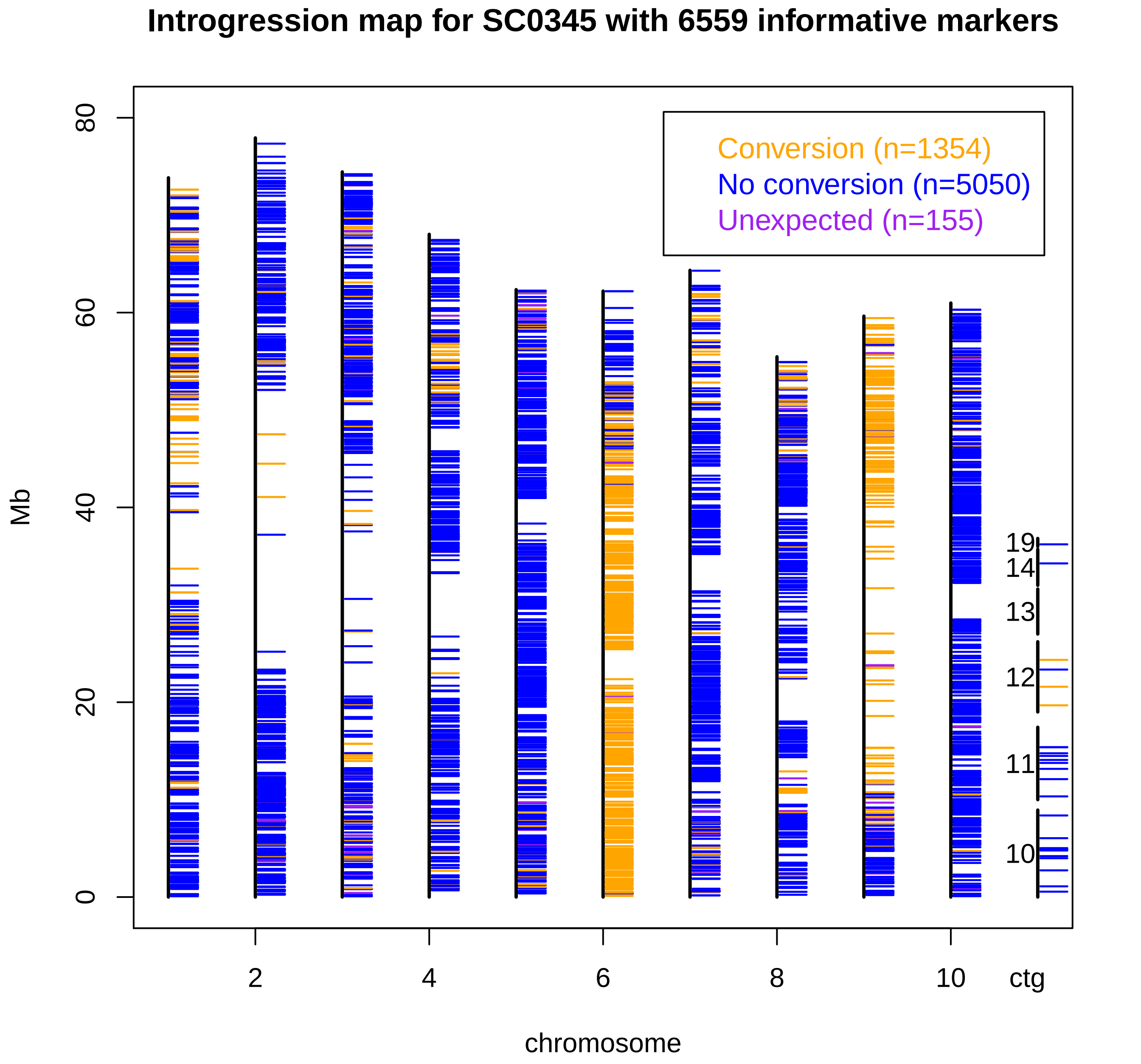

Introgression map for SC0345 with 6559 informative markers
Conversion (n=1354)
No conversion (n=5050)
Unexpected (n=155)
Mb
0
20
40
60
80
2
4
6
8
10
ctg
chromosome
19
14
13
12
11
10

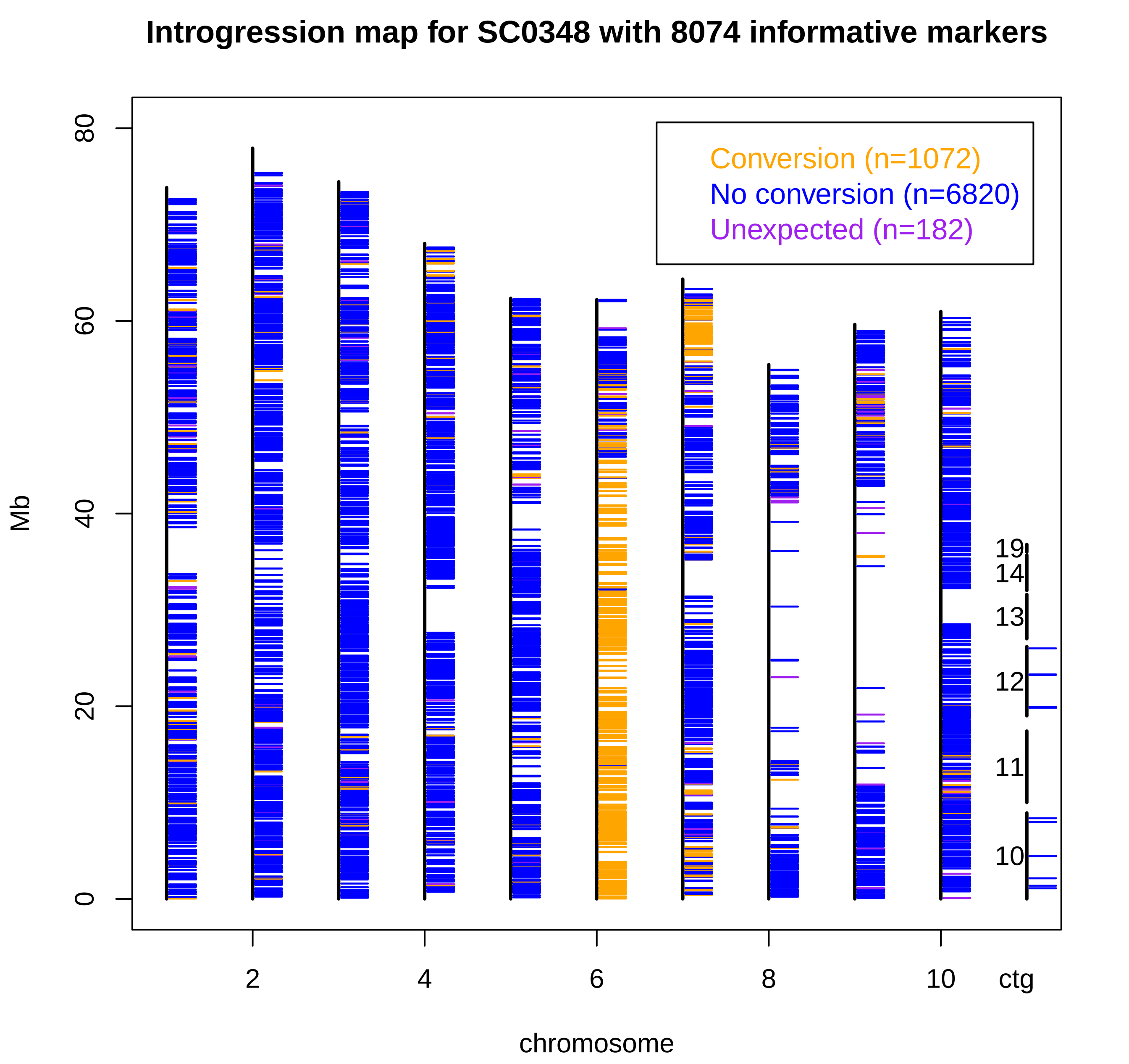

Introgression map for SC0348 with 8074 informative markers
Conversion (n=1072)
No conversion (n=6820)
Unexpected (n=182)
Mb
0
20
40
60
80
2
4
6
8
10
ctg
19
14
13
12
11
10
chromosome

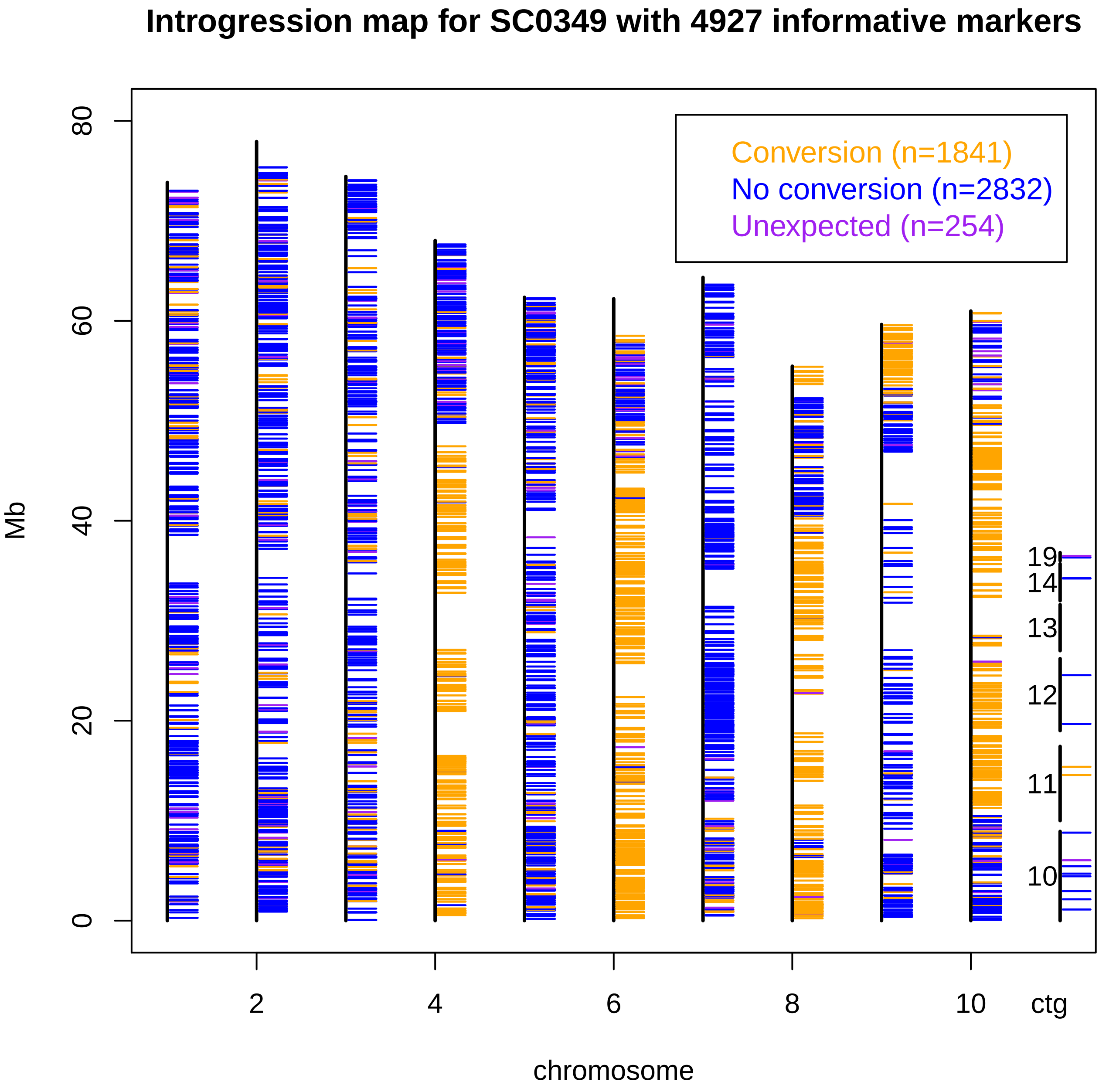

Introgression map for SC0349 with 4927 informative markers
Conversion (n=1841)
No conversion (n=2832)
Unexpected (n=254)
Mb
0
20
40
60
80
chromosome
2
4
6
8
10
ctg
19
14
13
12
11
10

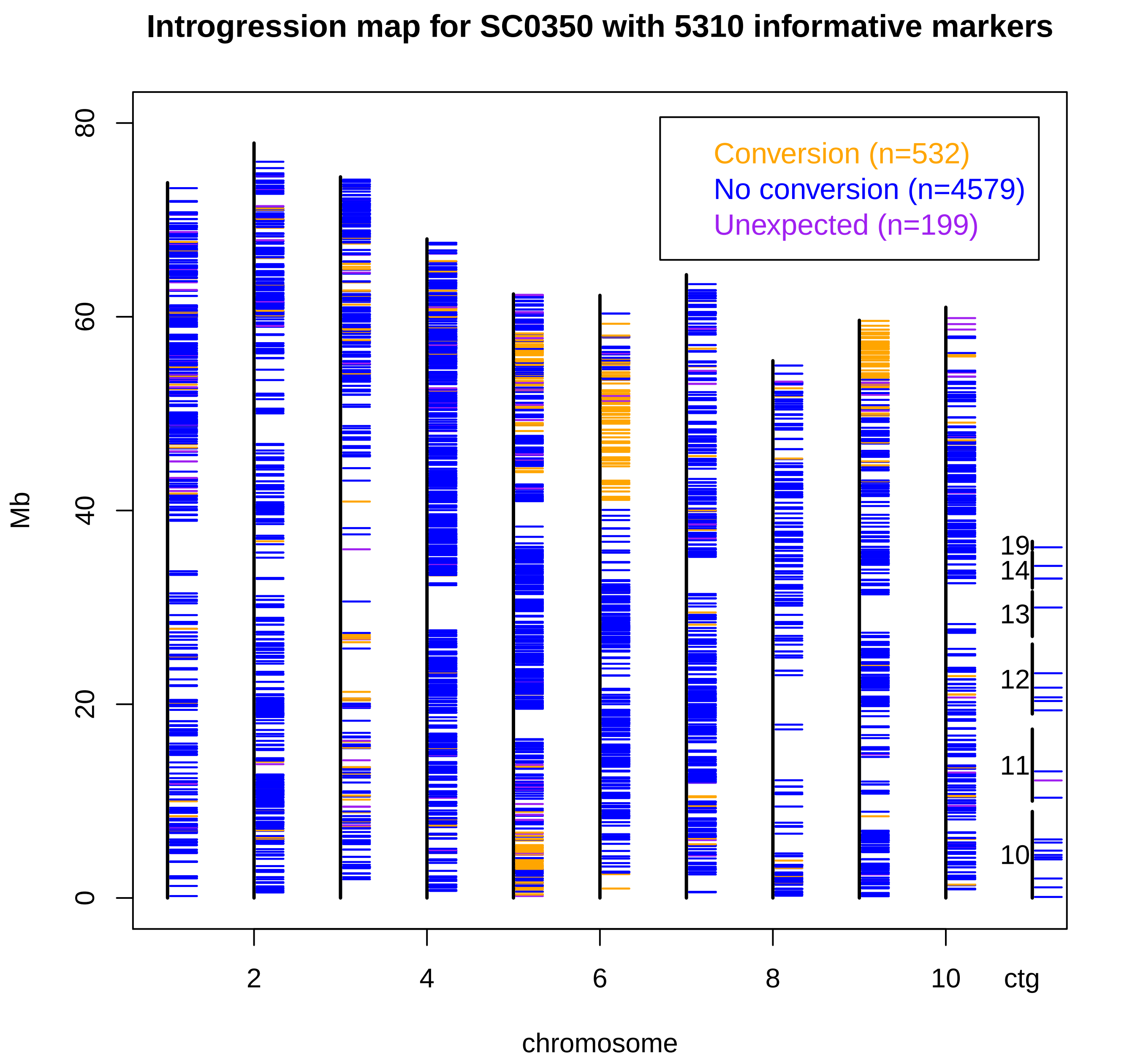

Introgression map for SC0350 with 5310 informative markers
Conversion (n=532)
No conversion (n=4579)
Unexpected (n=199)
Mb
0
20
40
60
80
chromosome
2
4
6
8
10
ctg
19
14
13
12
11
10

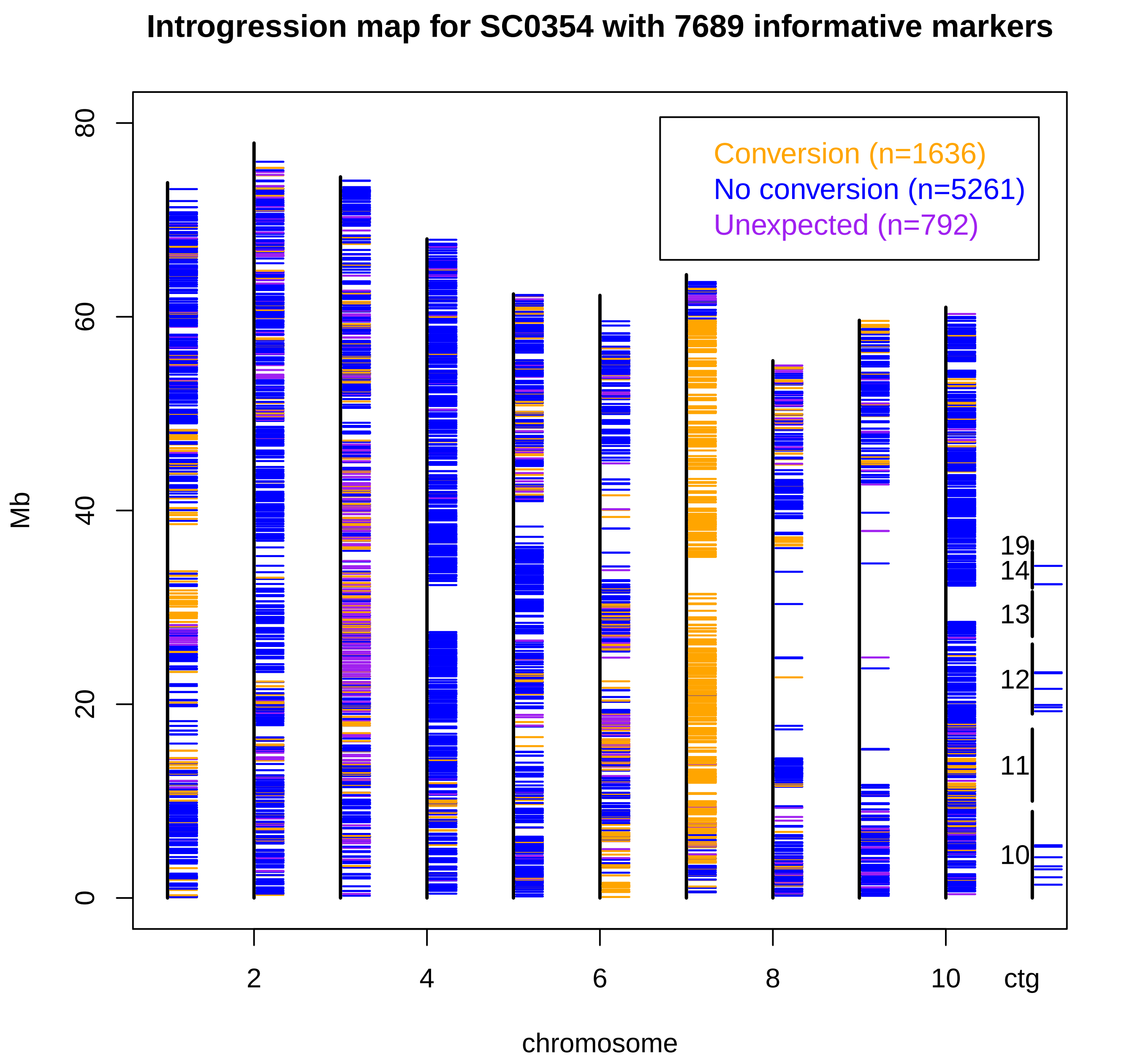
Introgression map for SC0354 with 7689 informative markers
Conversion (n=1636)
No conversion (n=5261)
Unexpected (n=792)
Mb
0
20
40
60
80
chromosome
2
4
6
8
10
ctg
19
14
13
12
11
10

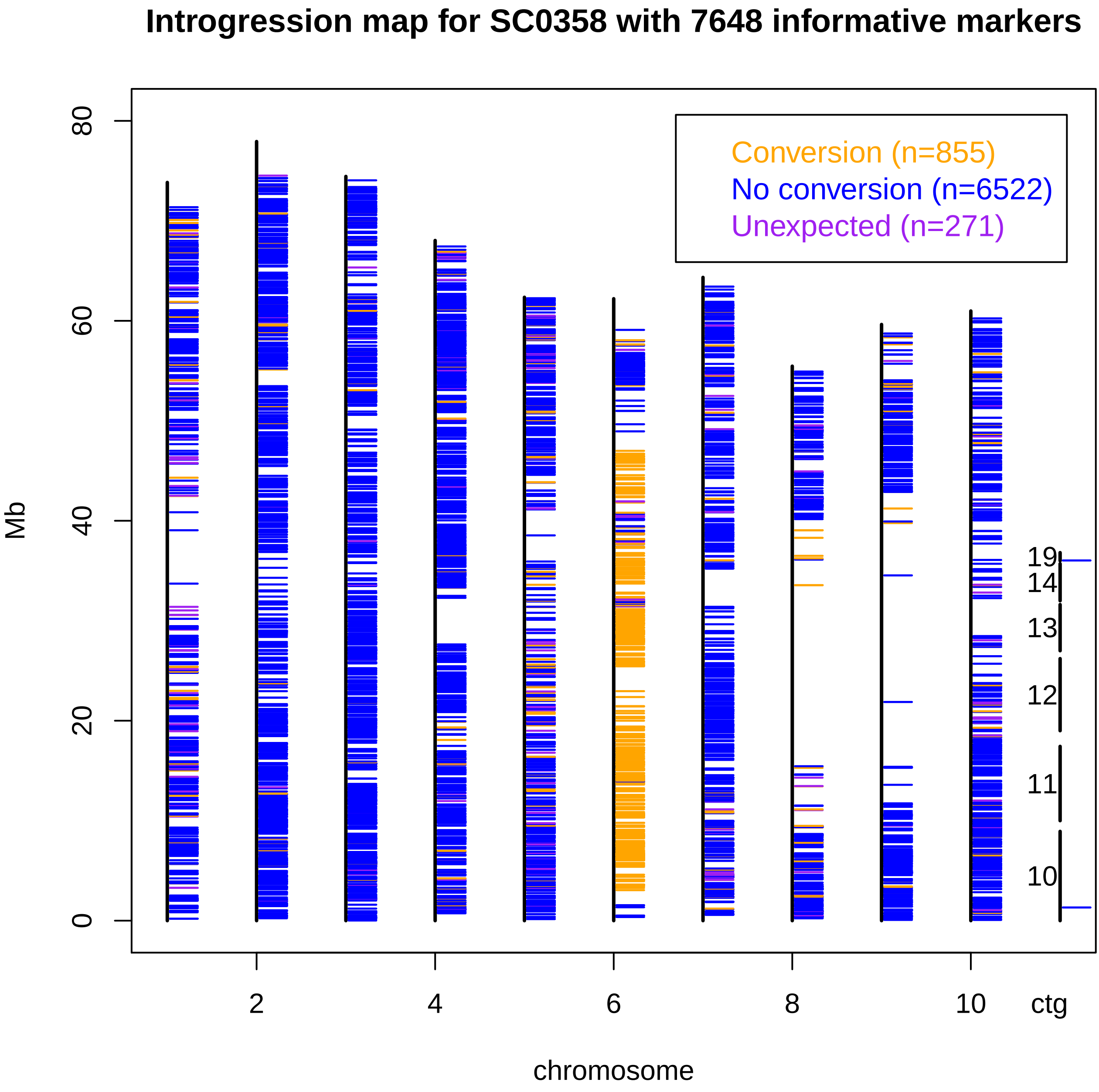

Introgression map for SC0358 with 7648 informative markers
Conversion (n=855)
No conversion (n=6522)
Unexpected (n=271)
Mb
0
20
40
60
80
chromosome
2
4
6
8
10
ctg
19
14
13
12
11
10

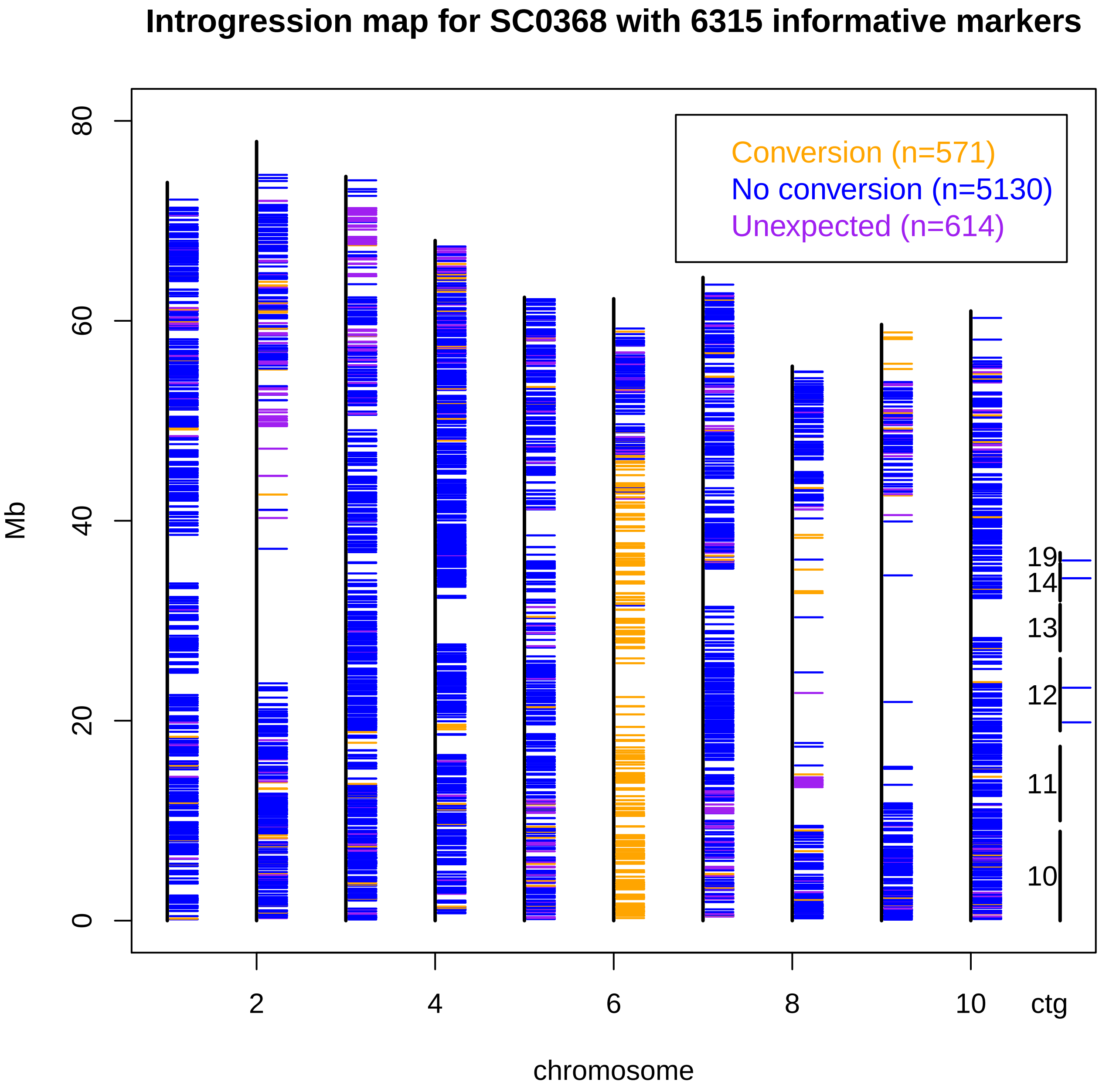
Introgression map for SC0368 with 6315 informative markers
Conversion (n=571)
No conversion (n=5130)
Unexpected (n=614)
Mb
0
20
40
60
80
2
4
6
8
10
ctg
19
14
13
12
11
10
chromosome

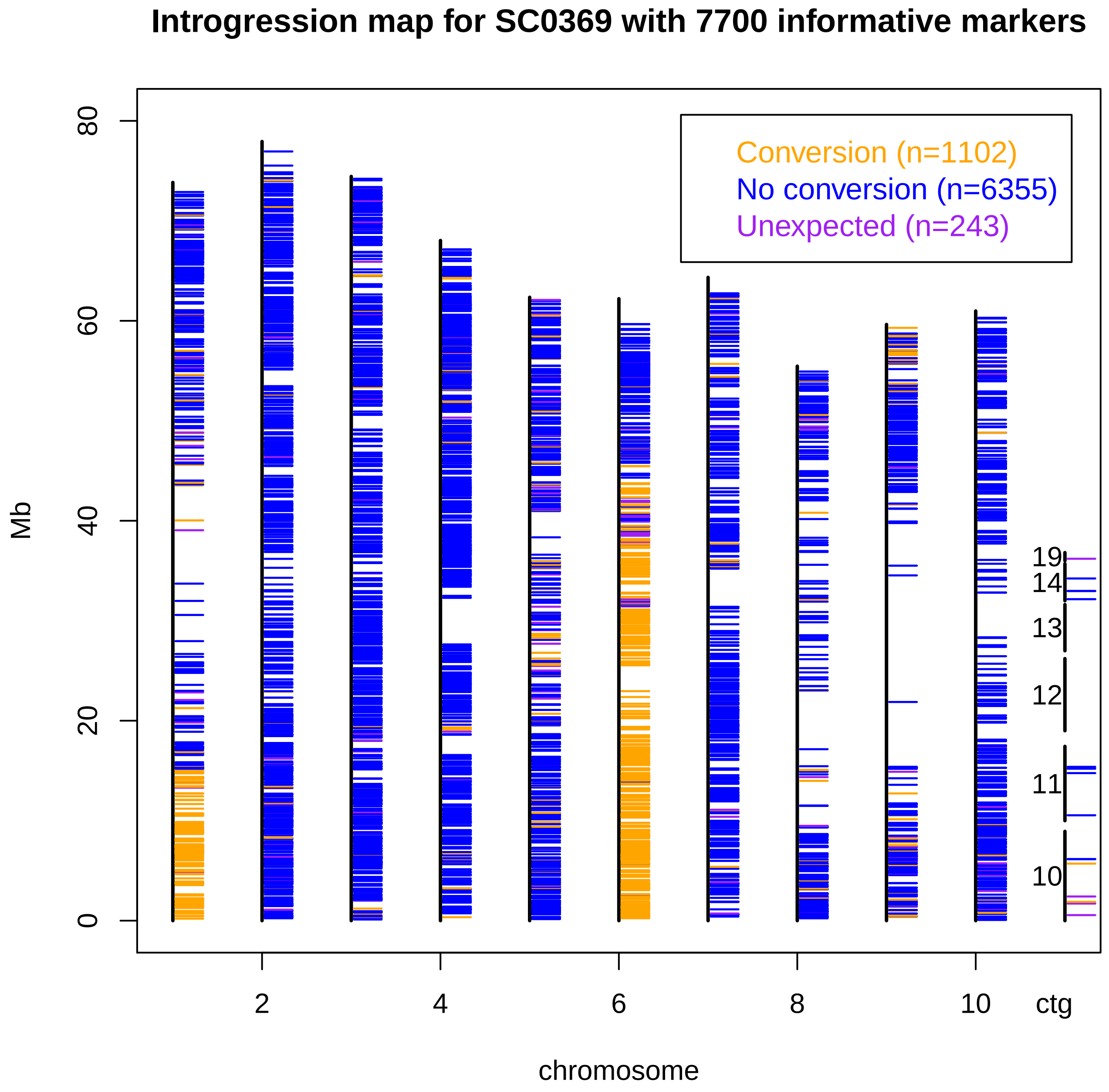

Introgression map for SC0369 with 7700 informative markers
Conversion (n=1102)
No conversion (n=6355)
Unexpected (n=243)
Mb
0
20
40
60
80
2
4
6
8
10
ctg
19
14
13
12
11
10
chromosome

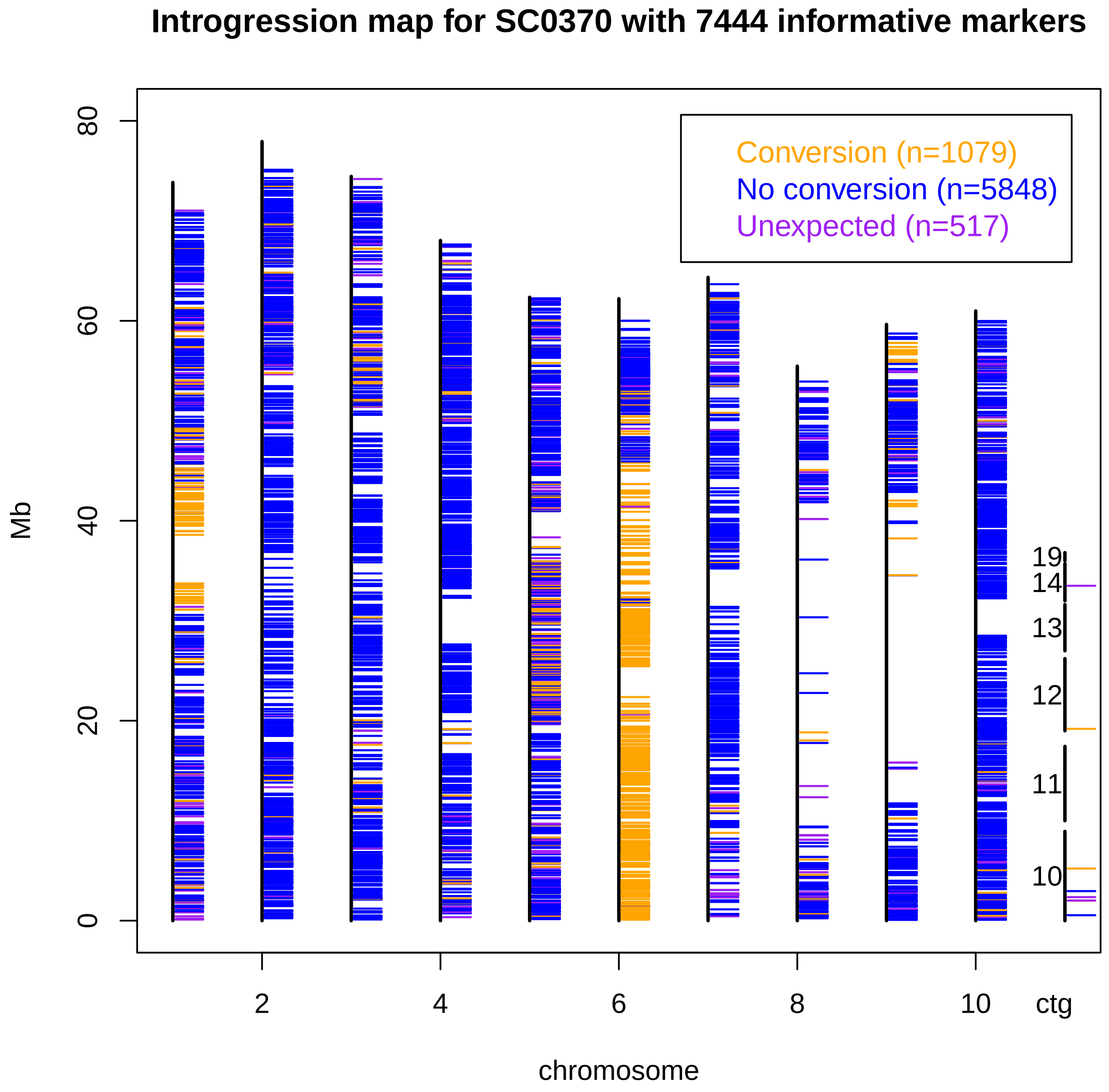

Introgression map for SC0370 with 7444 informative markers
Conversion (n=1079)
No conversion (n=5848)
Unexpected (n=517)
Mb
0
20
40
60
80
2
4
6
8
10
ctg
chromosome
19
14
13
12
11
10

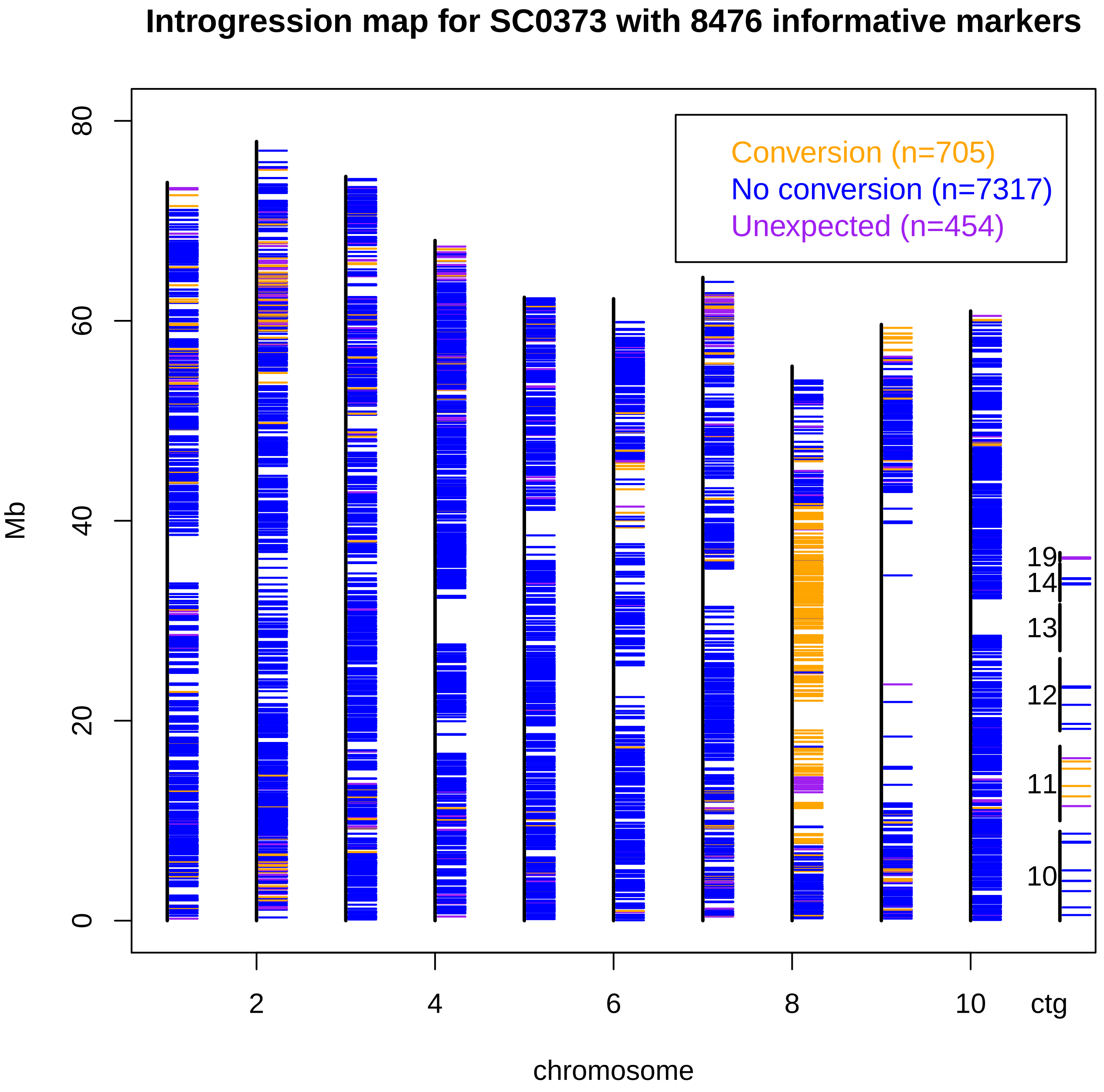

Introgression map for SC0373 with 8476 informative markers
Conversion (n=705)
No conversion (n=7317)
Unexpected (n=454)
Mb
0
20
40
60
80
chromosome
2
4
6
8
10
ctg
19
14
13
12
11
10

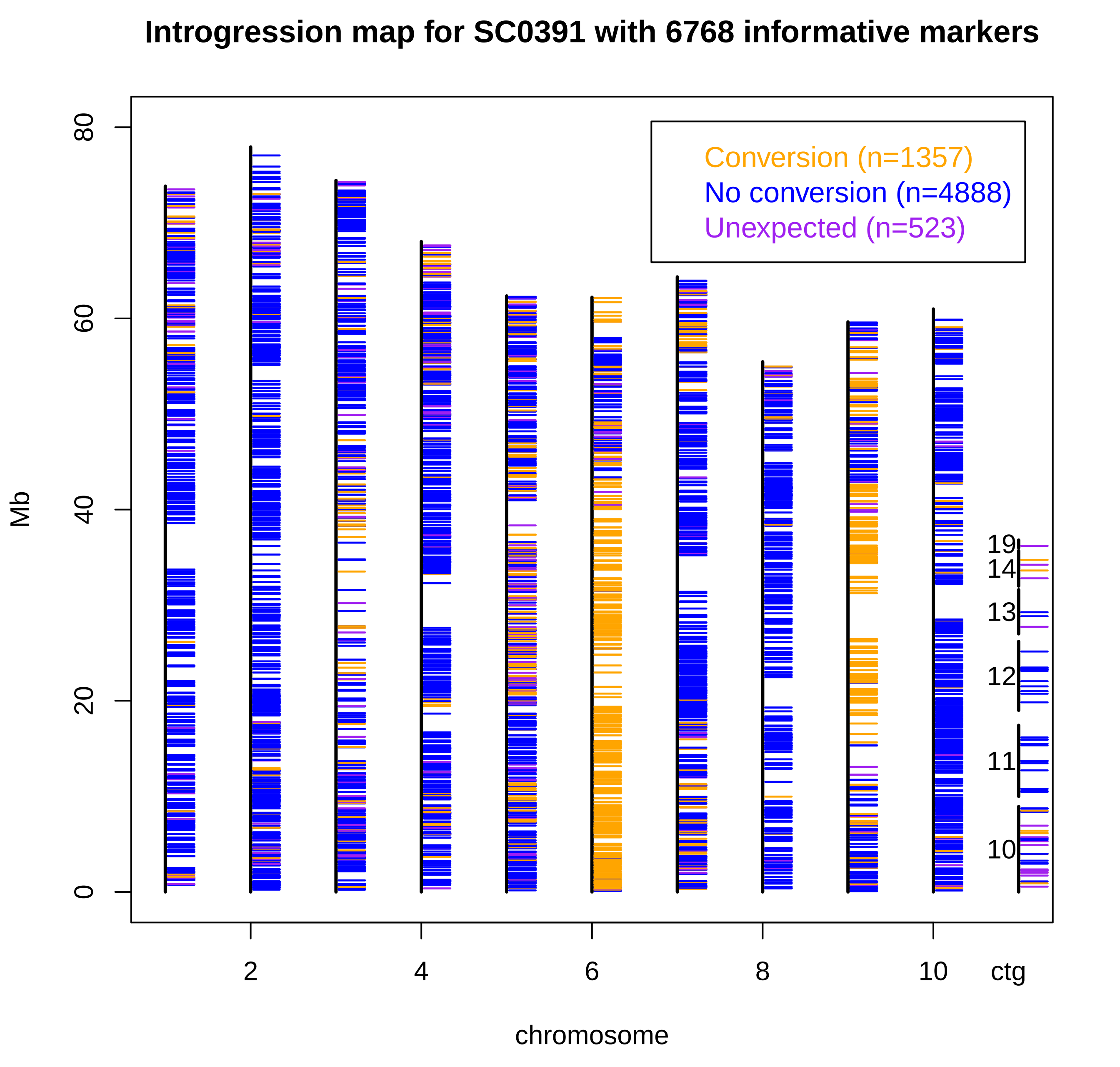

Introgression map for SC0391 with 6768 informative markers
Conversion (n=1357)
No conversion (n=4888)
Unexpected (n=523)
Mb
0
20
40
60
80
chromosome
2
4
6
8
10
ctg
19
14
13
12
11
10

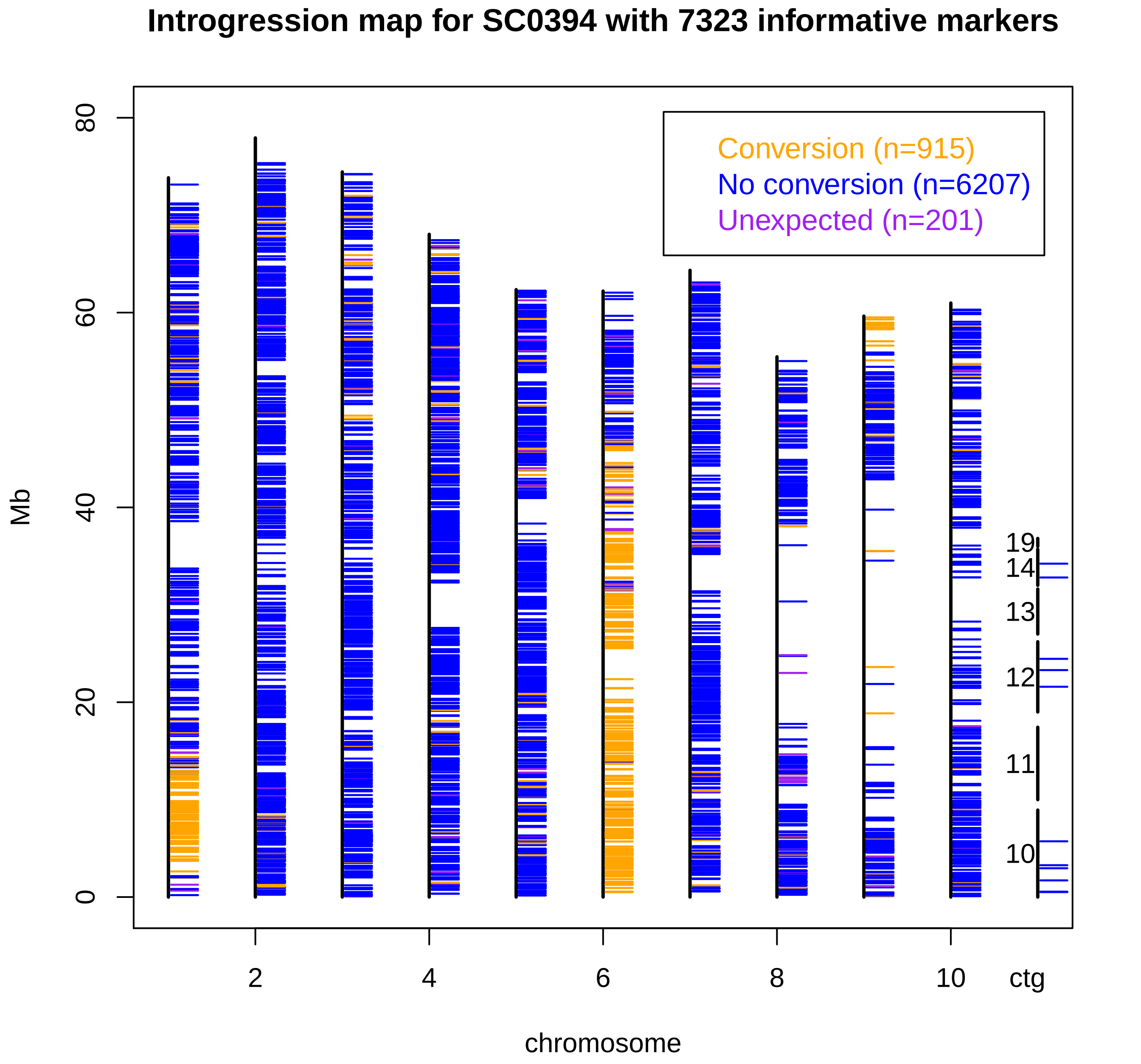
Introgression map for SC0394 with 7323 informative markers
Conversion (n=915)
No conversion (n=6207)
Unexpected (n=201)
Mb
0
20
40
60
80
chromosome
2
4
6
8
10
ctg
19
14
13
12
11
10

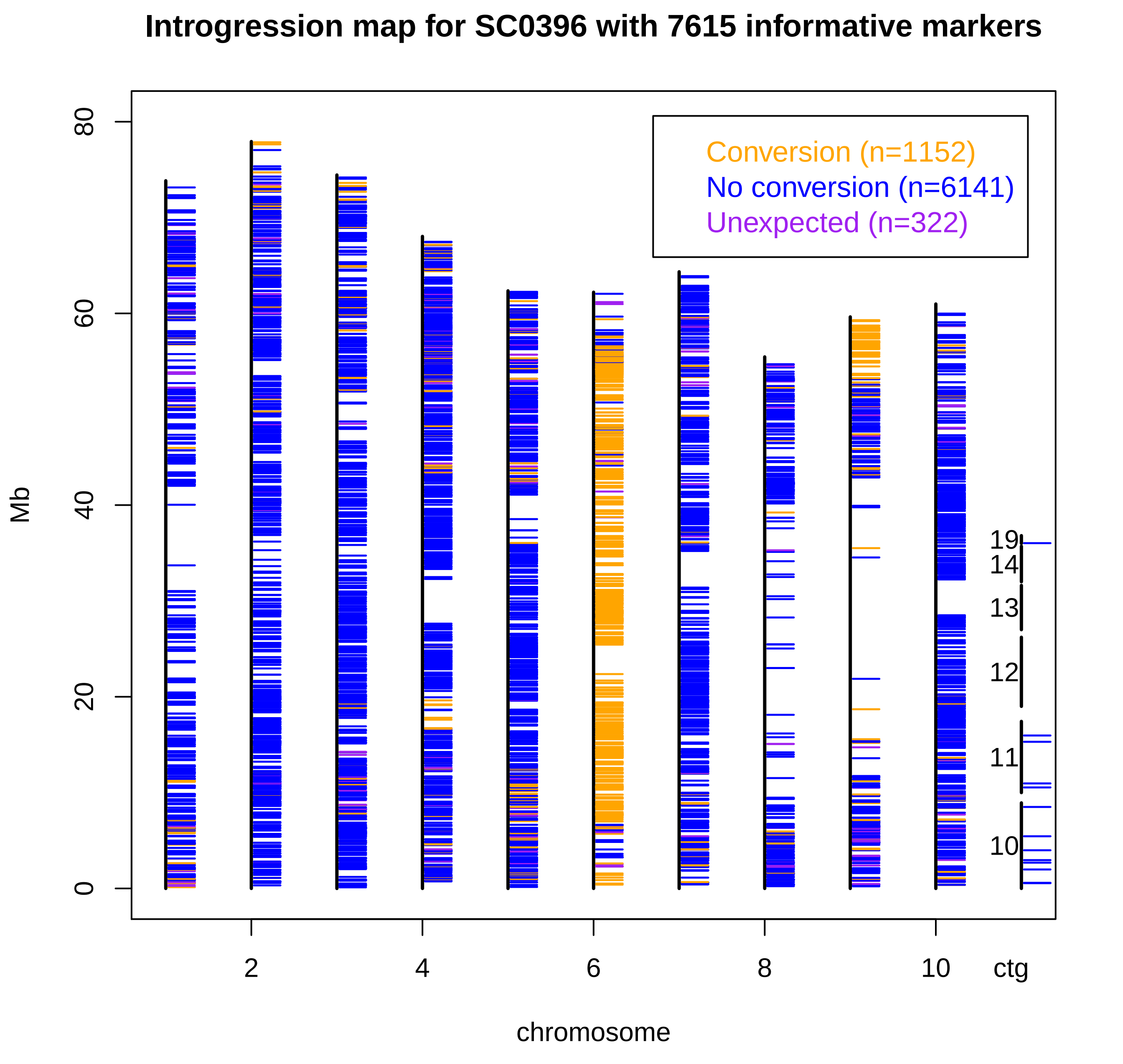

Introgression map for SC0396 with 7615 informative markers
Conversion (n=1152)
No conversion (n=6141)
Unexpected (n=322)
Mb
0
20
40
60
80
chromosome
2
4
6
8
10
ctg
19
14
13
12
11
10

Introgression map for SC0397 with 6818 informative markers

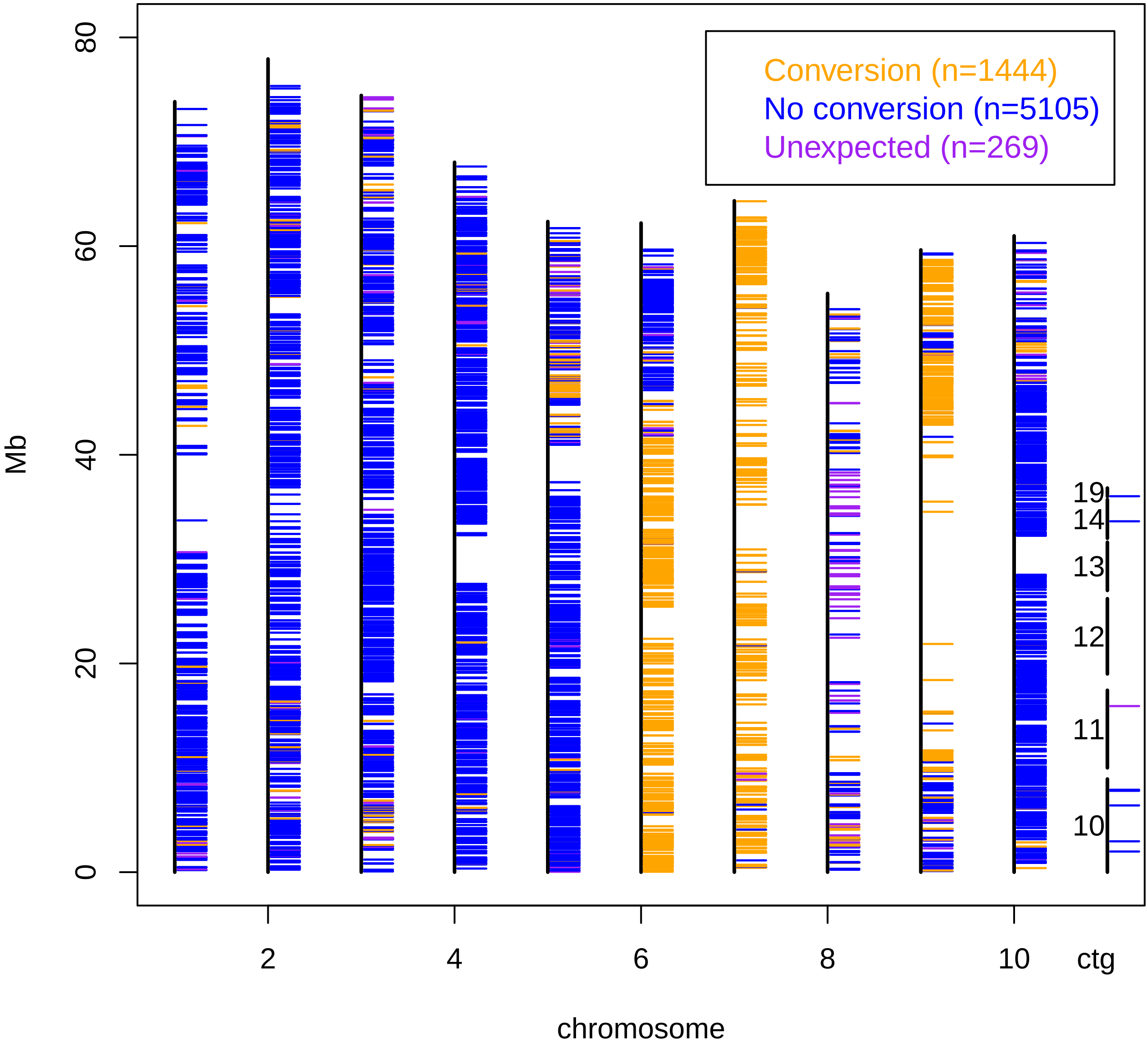

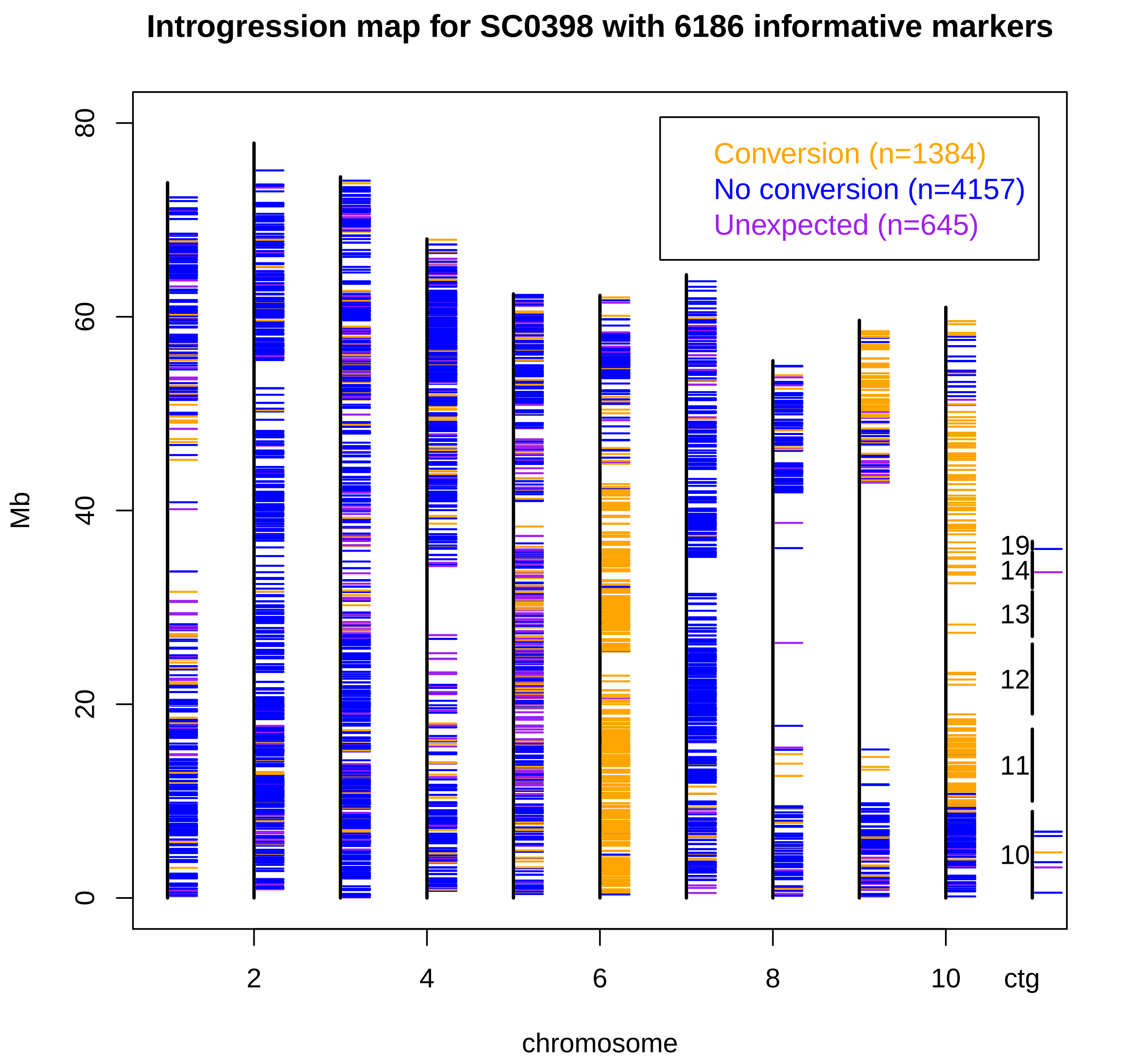
Introgression map for SC0398 with 6186 informative markers
Conversion (n=1384)
No conversion (n=4157)
Unexpected (n=645)
Mb
0
20
40
60
80
2
4
6
8
10
ctg
19
14
13
12
11
10
chromosome

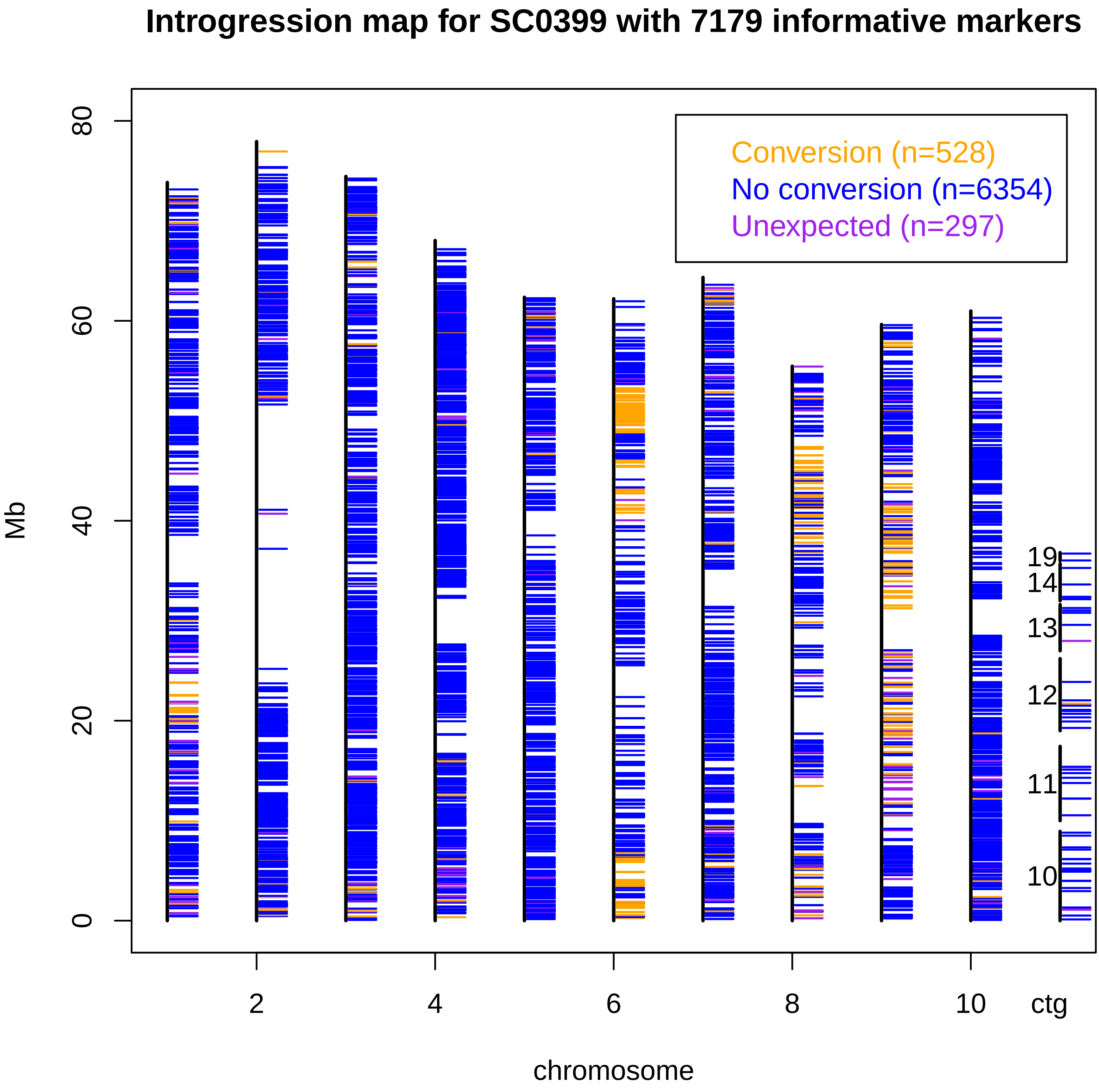

Introgression map for SC0399 with 7179 informative markers
Conversion (n=528)
No conversion (n=6354)
Unexpected (n=297)
Mb
0
20
40
60
80
2
4
6
8
10
ctg
19
14
13
12
11
10
chromosome

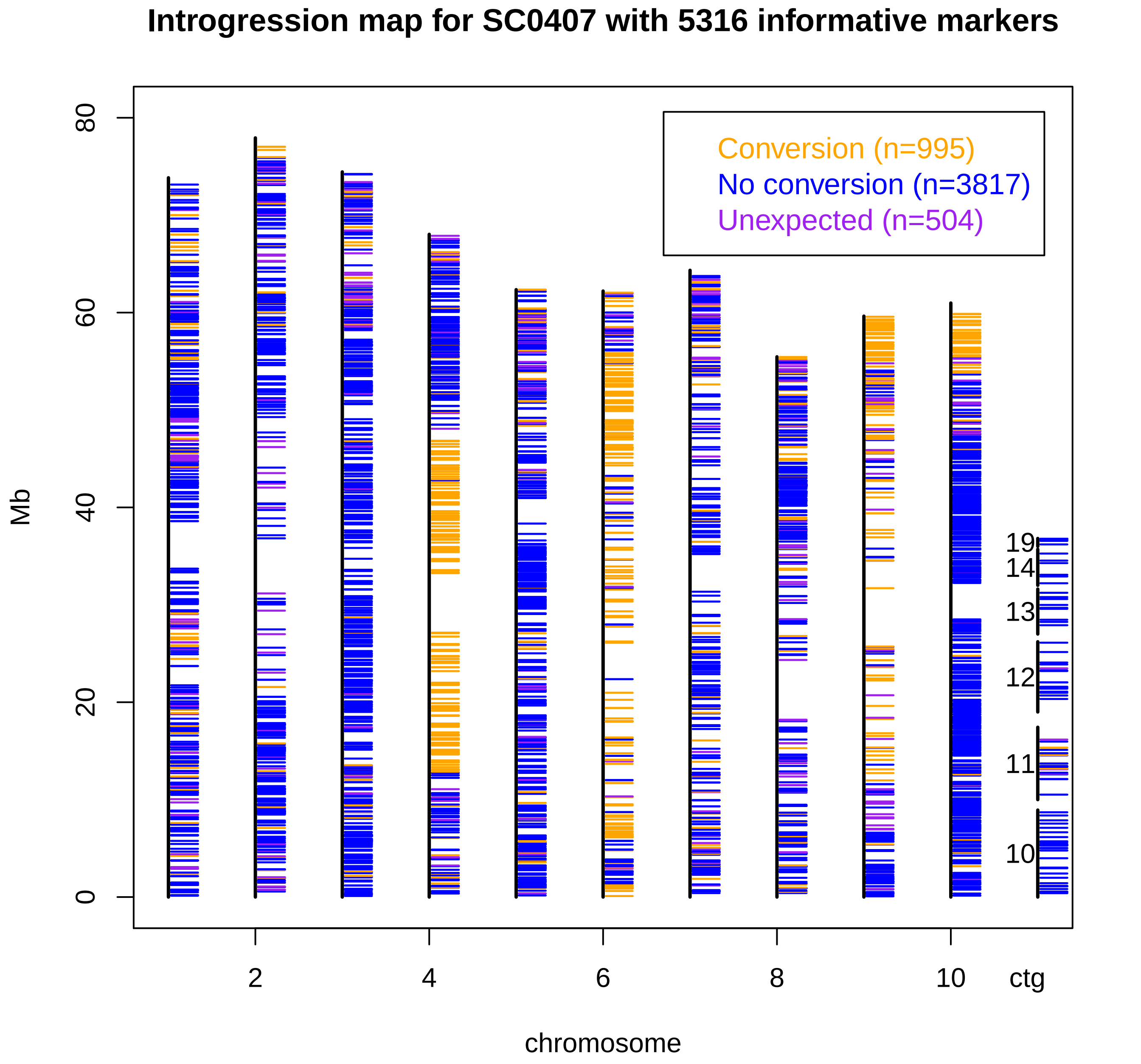
Introgression map for SC0407 with 5316 informative markers
Conversion (n=995)
No conversion (n=3817)
Unexpected (n=504)
Mb
0
20
40
60
80
chromosome
2
4
6
8
10
ctg
19
14
13
12
11
10

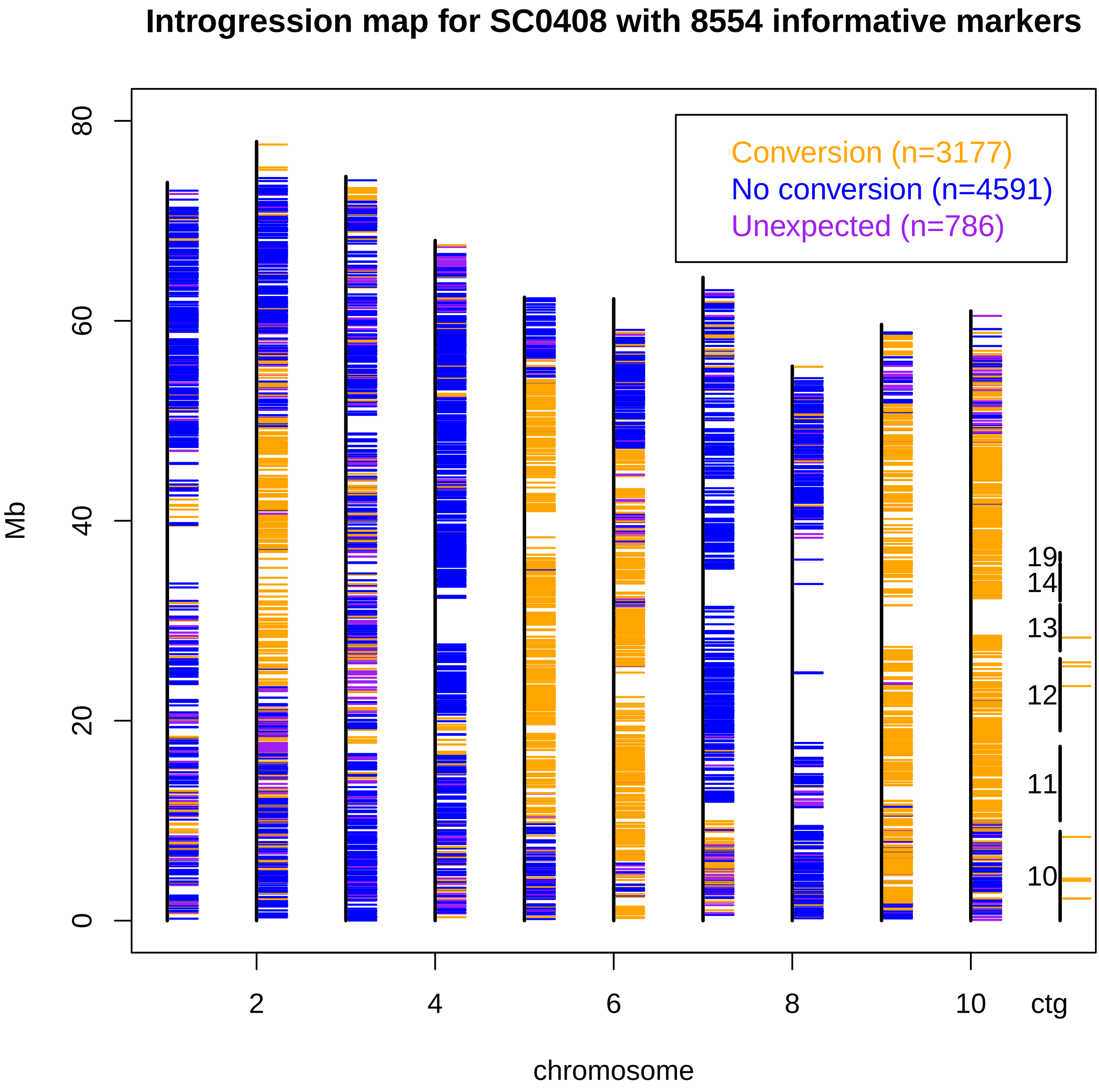
Introgression map for SC0408 with 8554 informative markers
Conversion (n=3177)
No conversion (n=4591)
Unexpected (n=786)
Mb
0
20
40
60
80
chromosome
2
4
6
8
10
ctg
19
14
13
12
11
10

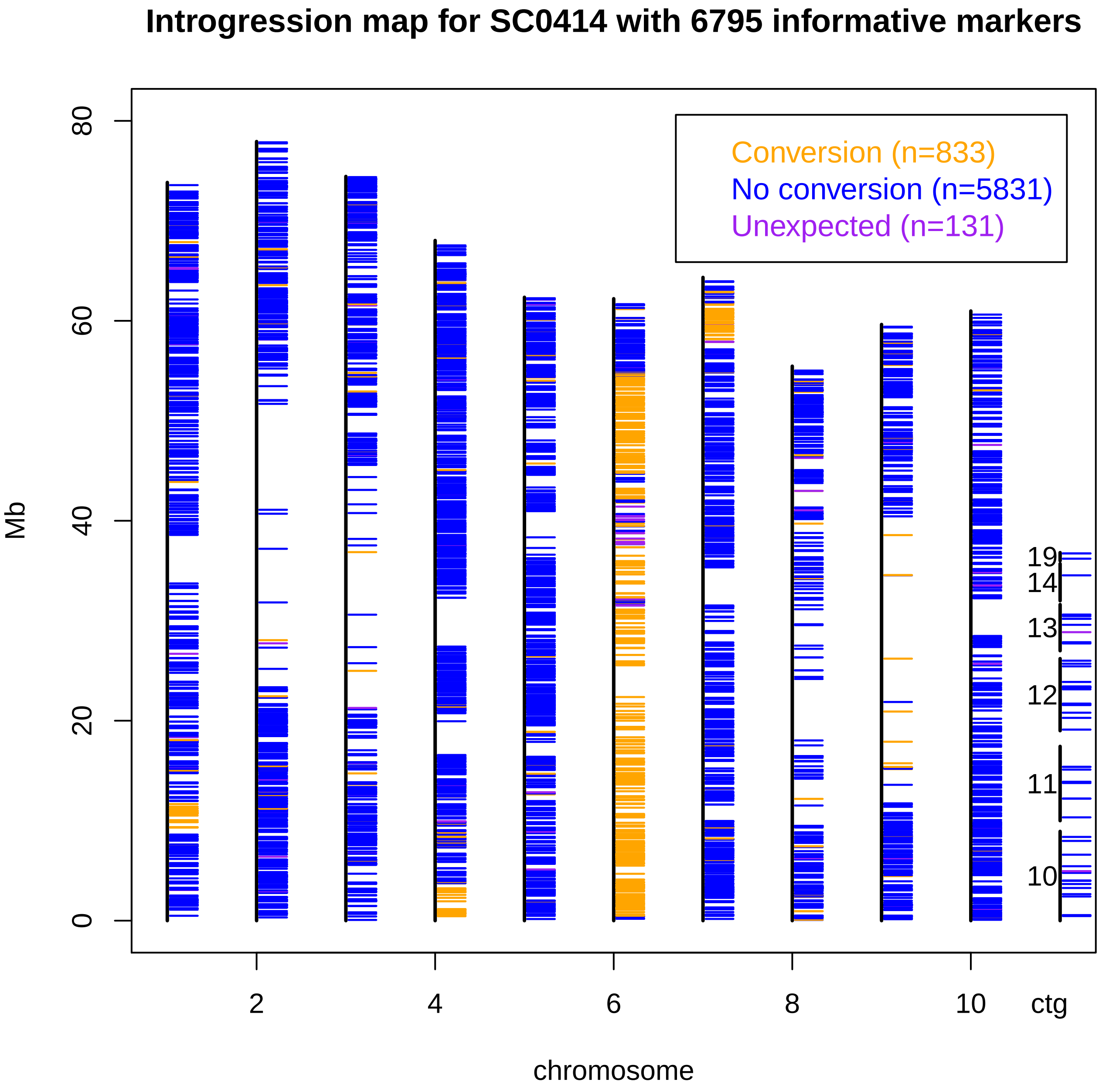

Introgression map for SC0414 with 6795 informative markers
Conversion (n=833)
No conversion (n=5831)
Unexpected (n=131)
Mb
0
20
40
60
80
chromosome
2
4
6
8
10
ctg
19
14
13
12
11
10

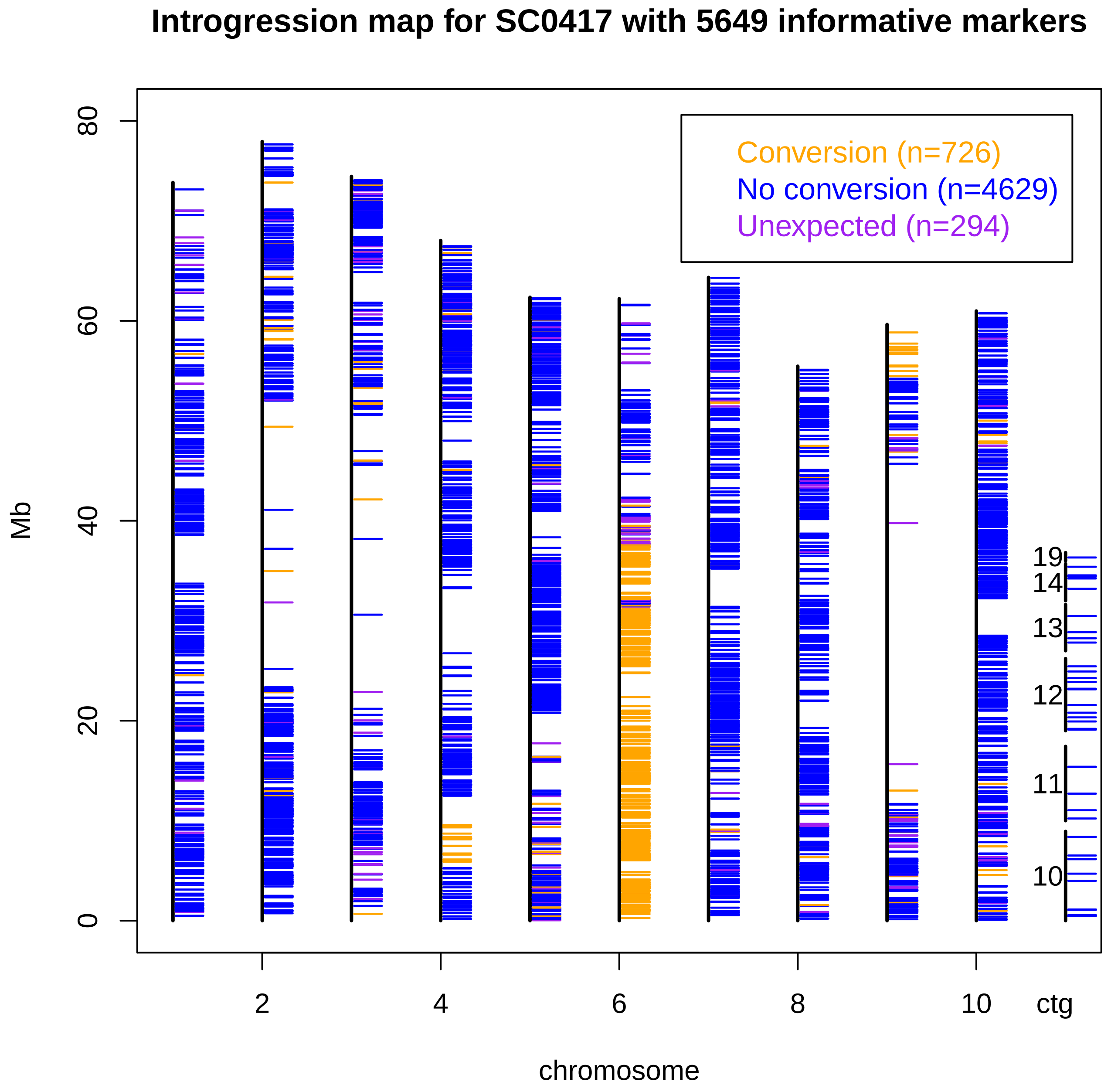

Introgression map for SC0417 with 5649 informative markers
Conversion (n=726)
No conversion (n=4629)
Unexpected (n=294)
Mb
0
20
40
60
80
chromosome
2
4
6
8
10
ctg
19
14
13
12
11
10

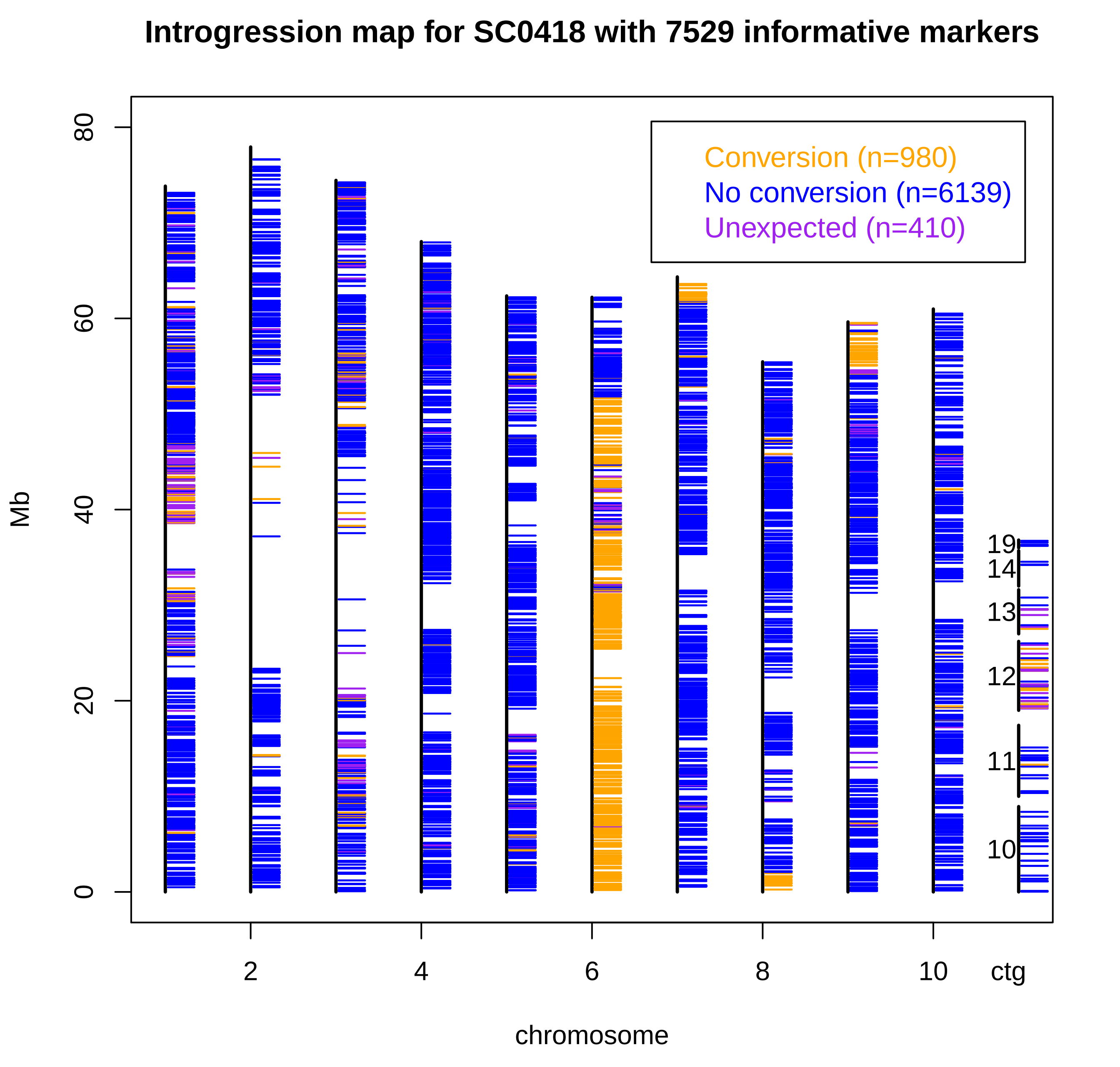
Introgression map for SC0418 with 7529 informative markers
Conversion (n=980)
No conversion (n=6139)
Unexpected (n=410)
Mb
0
20
40
60
80
chromosome
2
4
6
8
10
ctg
19
14
13
12
11
10

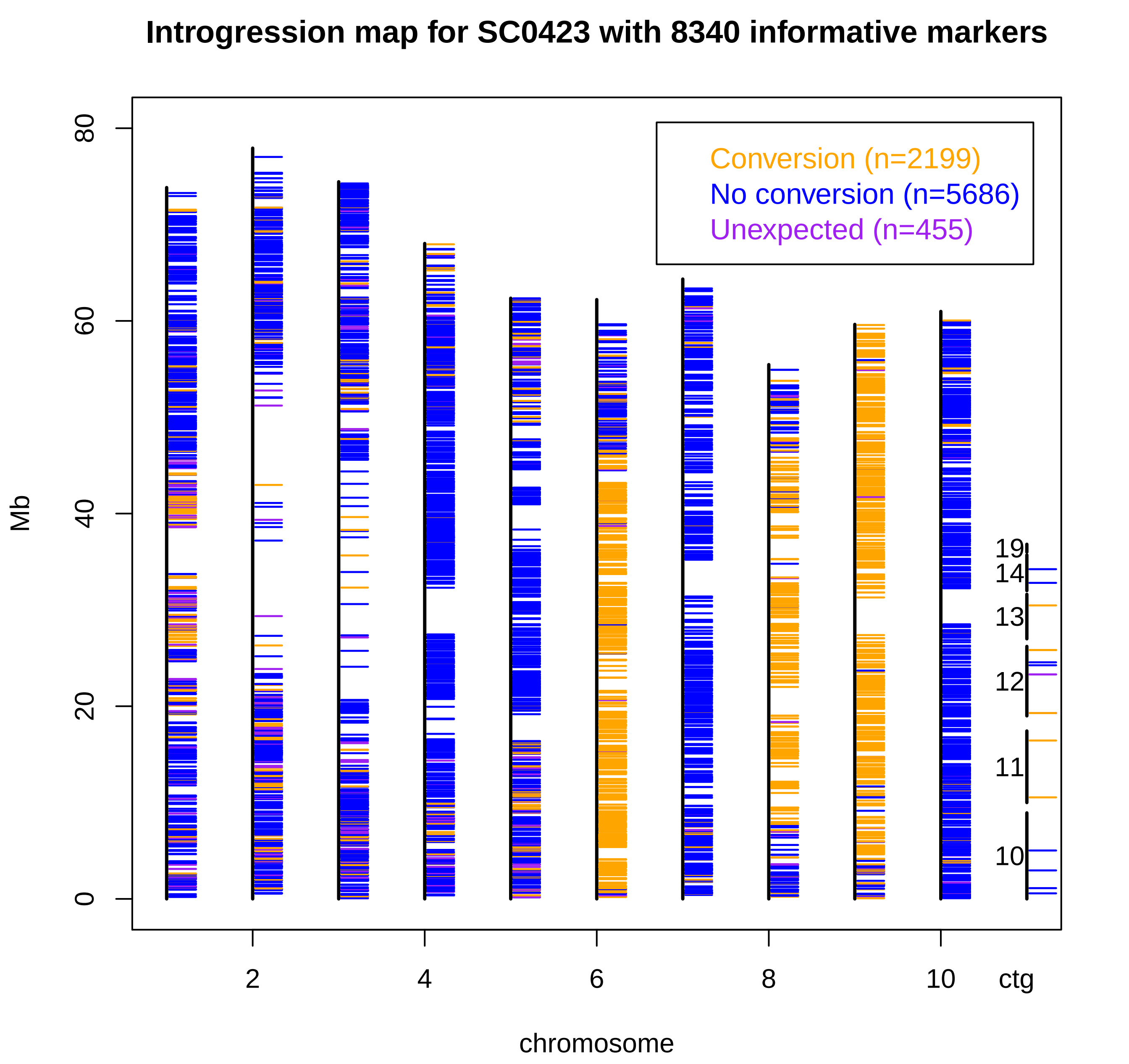
Introgression map for SC0423 with 8340 informative markers
Conversion (n=2199)
No conversion (n=5686)
Unexpected (n=455)
Mb
0
20
40
60
80
chromosome
2
4
6
8
10
ctg
19
14
13
12
11
10

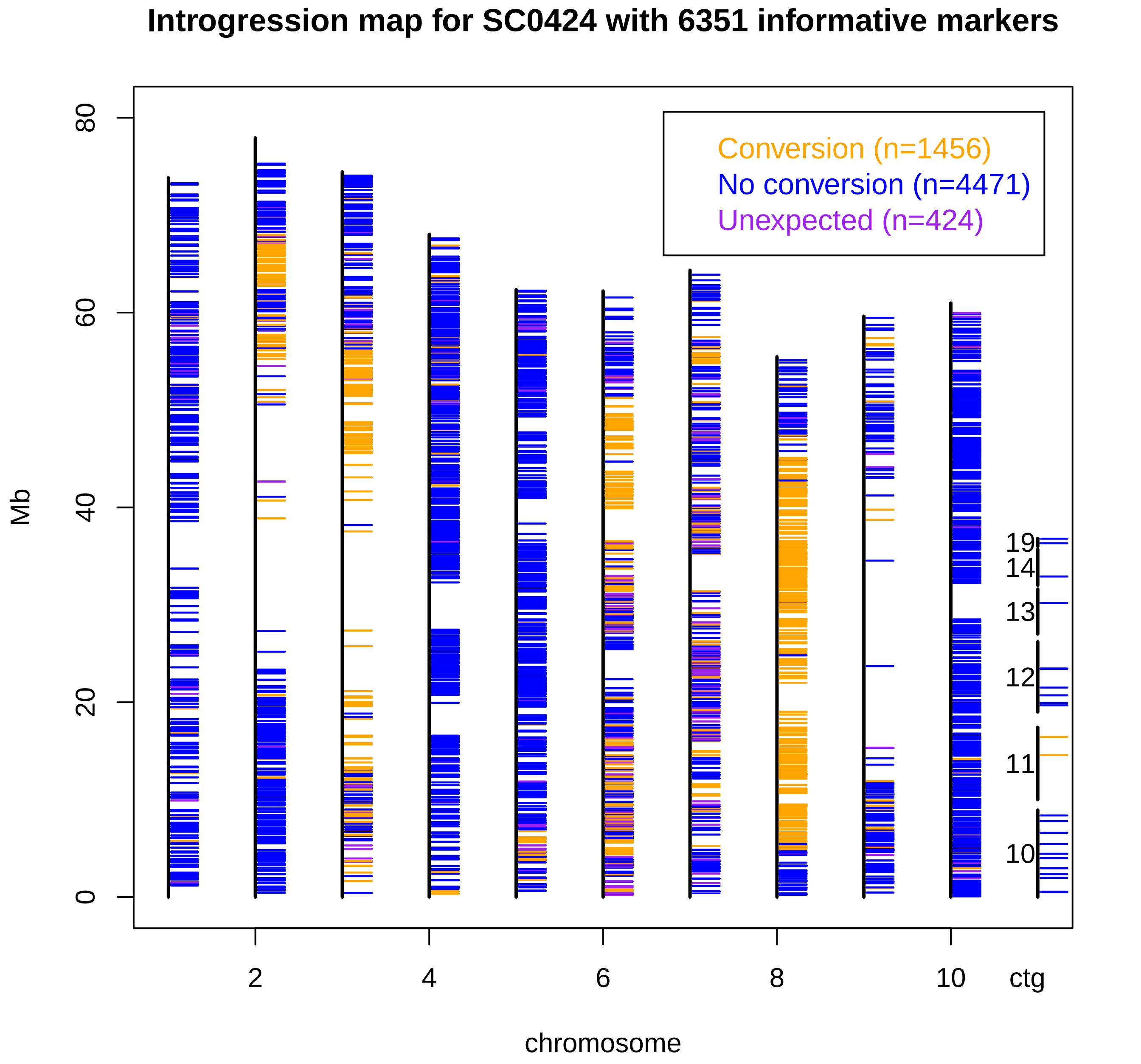

Introgression map for SC0424 with 6351 informative markers
Conversion (n=1456)
No conversion (n=4471)
Unexpected (n=424)
Mb
0
20
40
60
80
2
4
6
8
10
ctg
19
14
13
12
11
10
chromosome

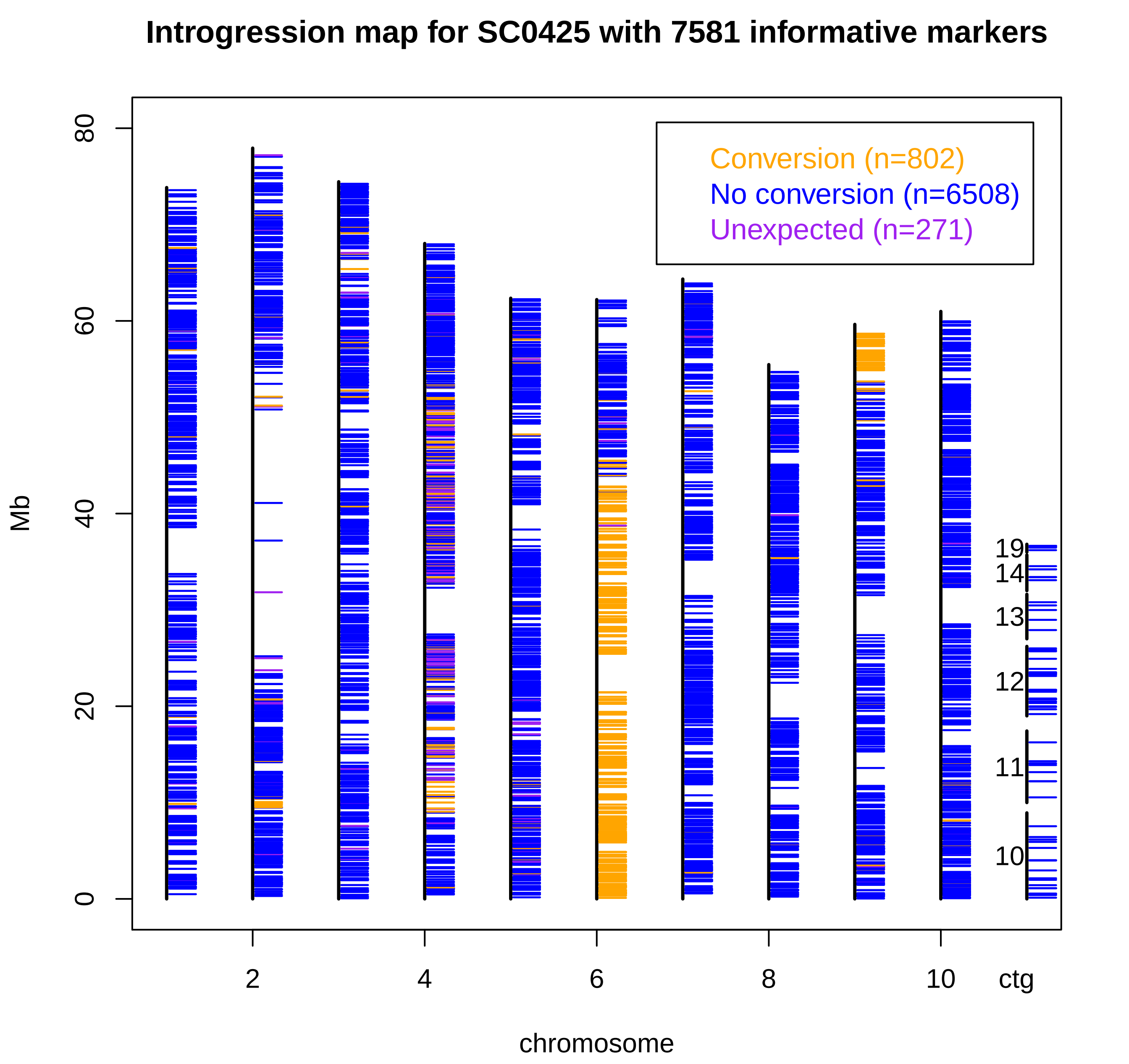

Introgression map for SC0425 with 7581 informative markers
Conversion (n=802)
No conversion (n=6508)
Unexpected (n=271)
Mb
0
20
40
60
80
2
4
6
8
10
ctg
19
14
13
12
11
10
chromosome

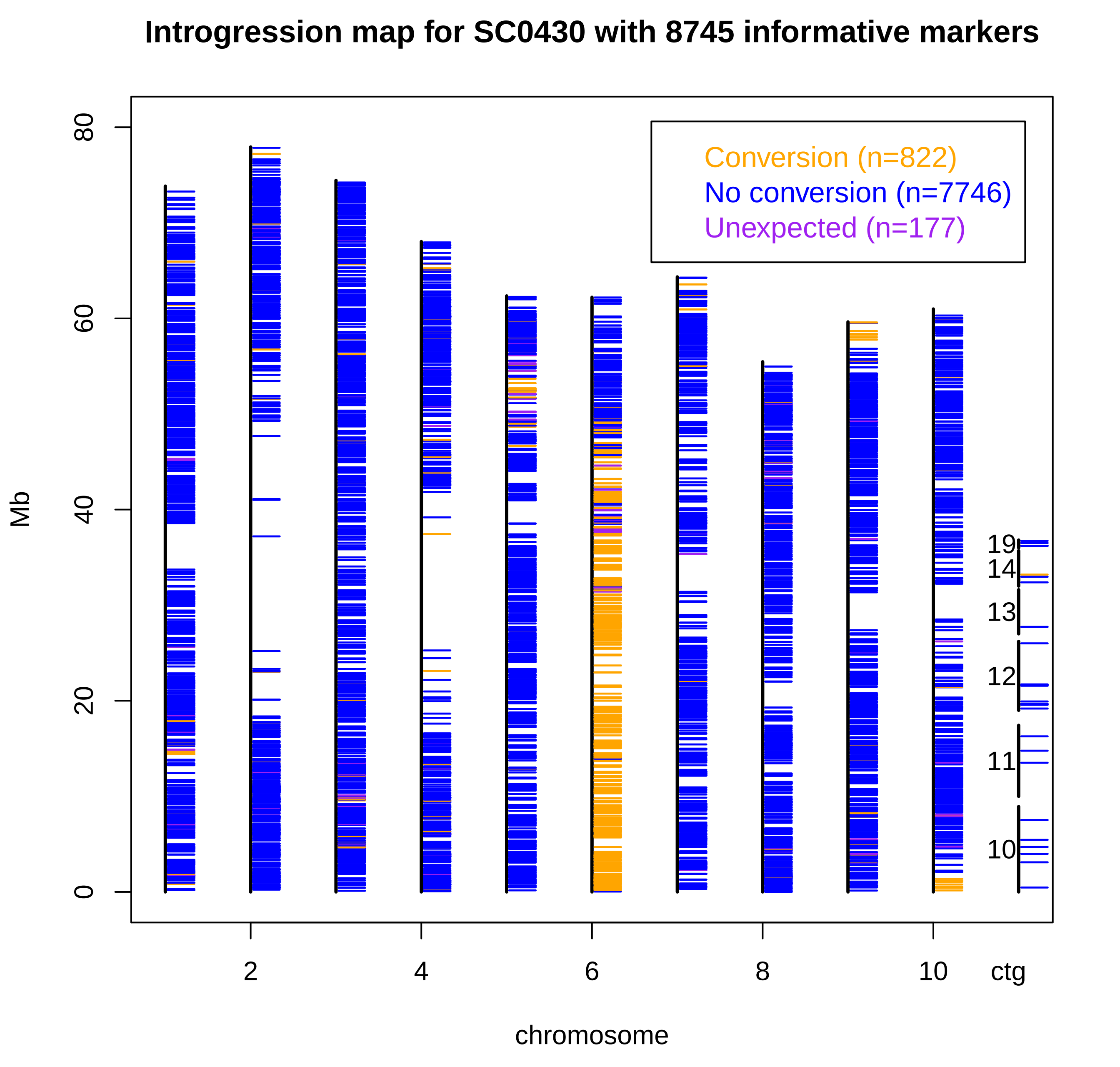
Introgression map for SC0430 with 8745 informative markers
Conversion (n=822)
No conversion (n=7746)
Unexpected (n=177)
Mb
0
20
40
60
80
chromosome
2
4
6
8
10
ctg
19
14
13
12
11
10

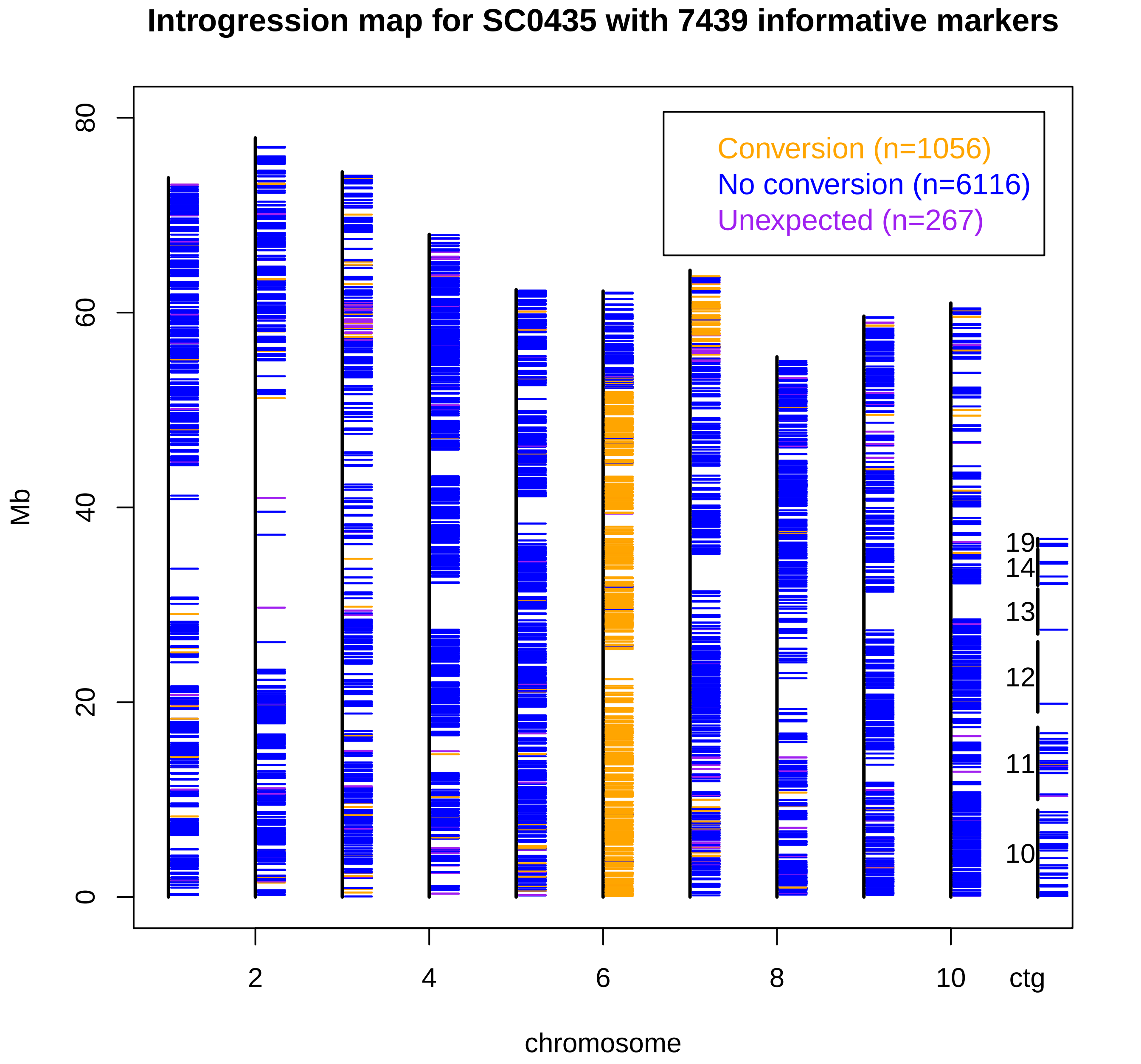

Introgression map for SC0435 with 7439 informative markers
Conversion (n=1056)
No conversion (n=6116)
Unexpected (n=267)
Mb
0
20
40
60
80
chromosome
2
4
6
8
10
ctg
19
14
13
12
11
10

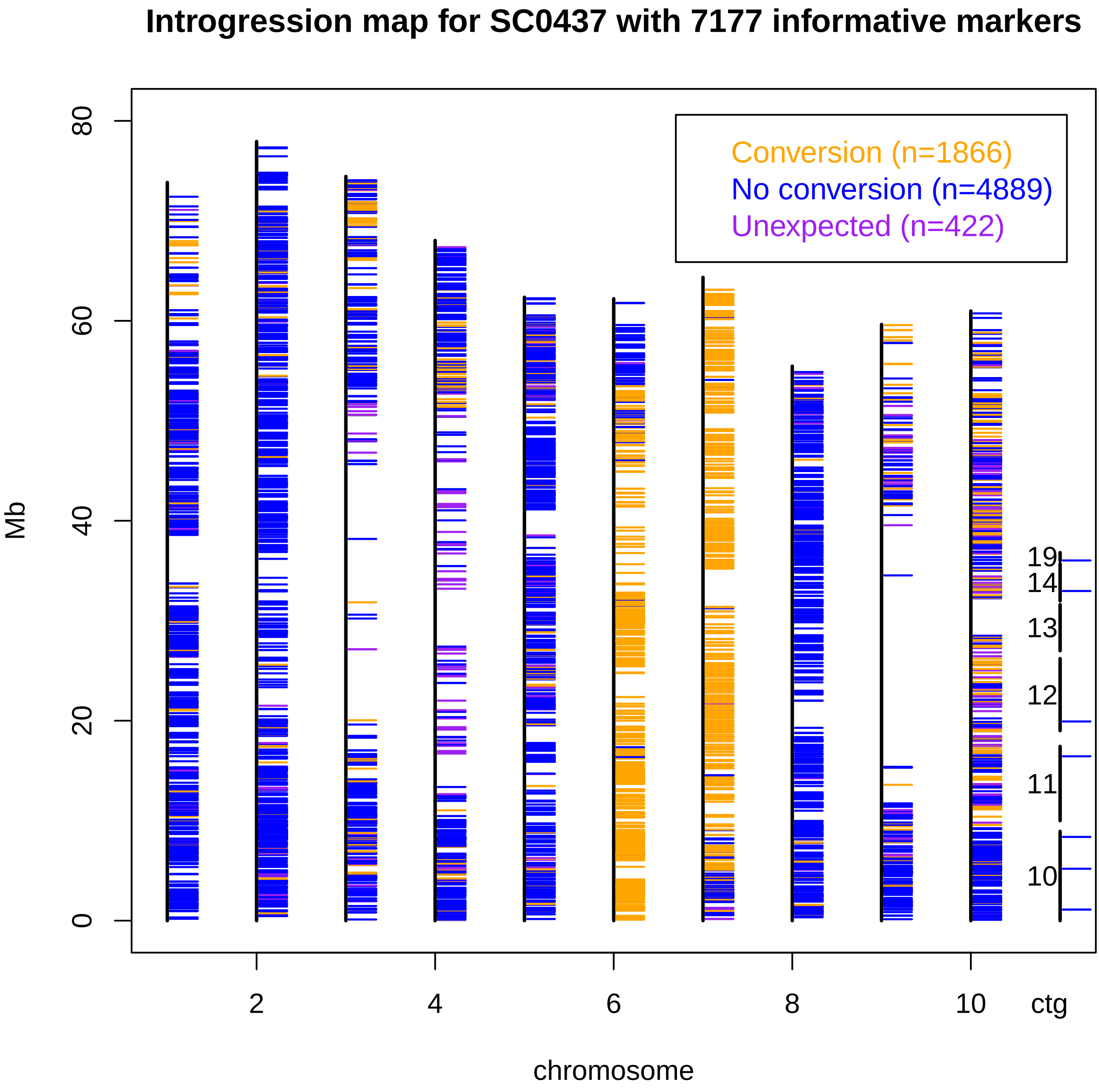
Introgression map for SC0437 with 7177 informative markers
Conversion (n=1866)
No conversion (n=4889)
Unexpected (n=422)
Mb
0
20
40
60
80
chromosome
2
4
6
8
10
ctg
19
14
13
12
11
10

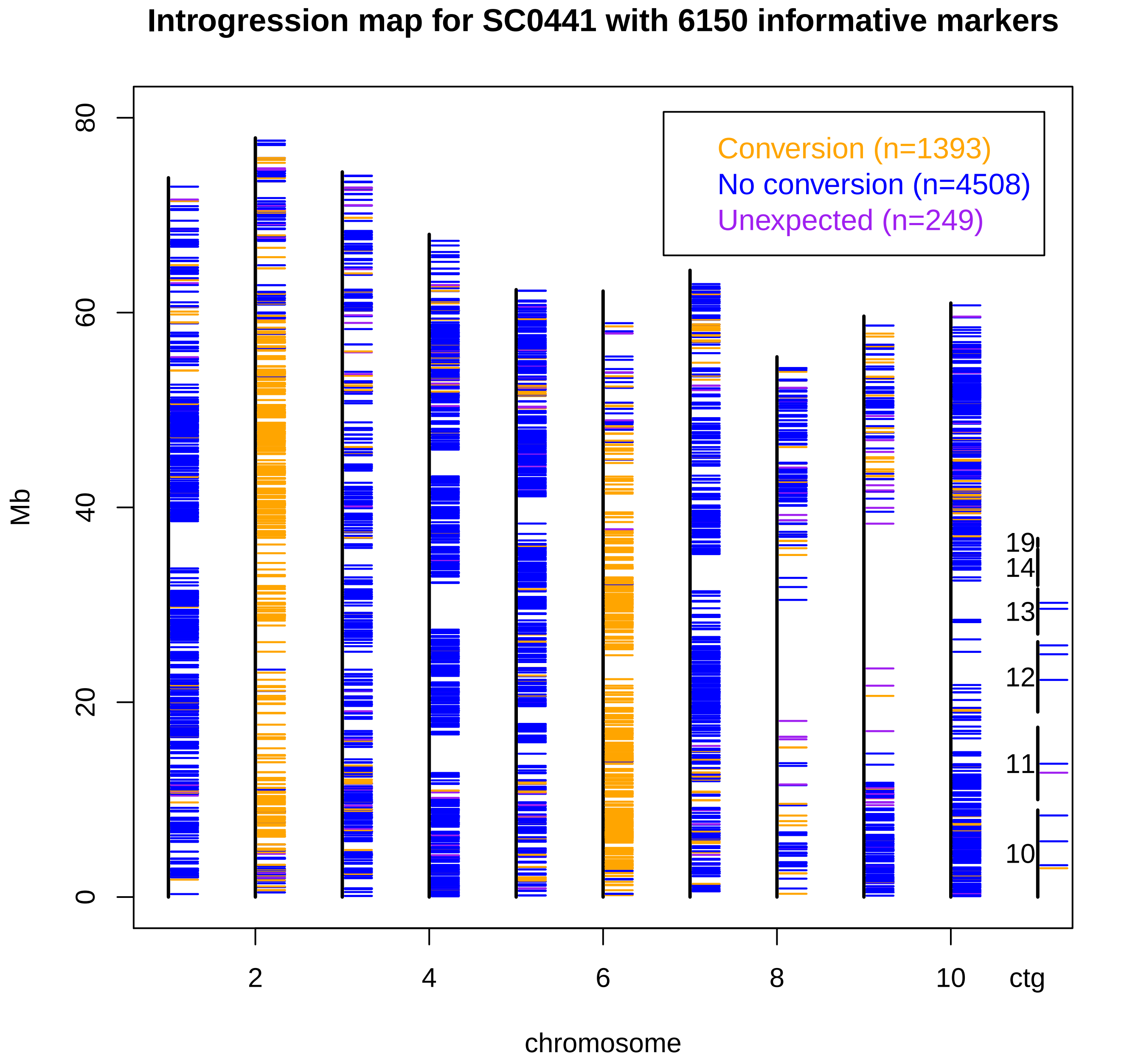

Introgression map for SC0441 with 6150 informative markers
Conversion (n=1393)
No conversion (n=4508)
Unexpected (n=249)
Mb
0
20
40
60
80
chromosome
2
4
6
8
10
ctg
19
14
13
12
11
10

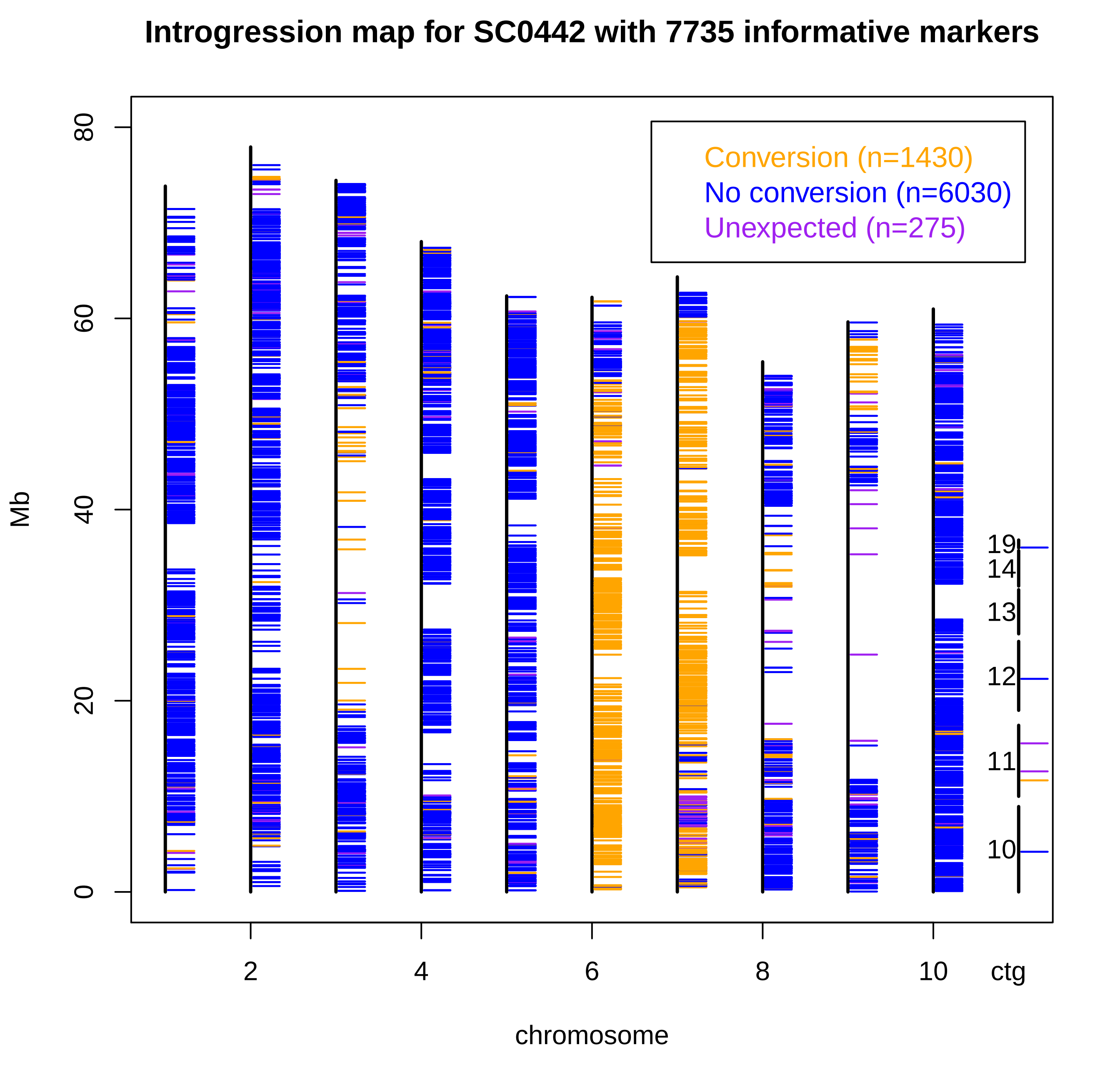

Introgression map for SC0442 with 7735 informative markers
Conversion (n=1430)
No conversion (n=6030)
Unexpected (n=275)
Mb
0
20
40
60
80
2
4
6
8
10
ctg
chromosome
19
14
13
12
11
10

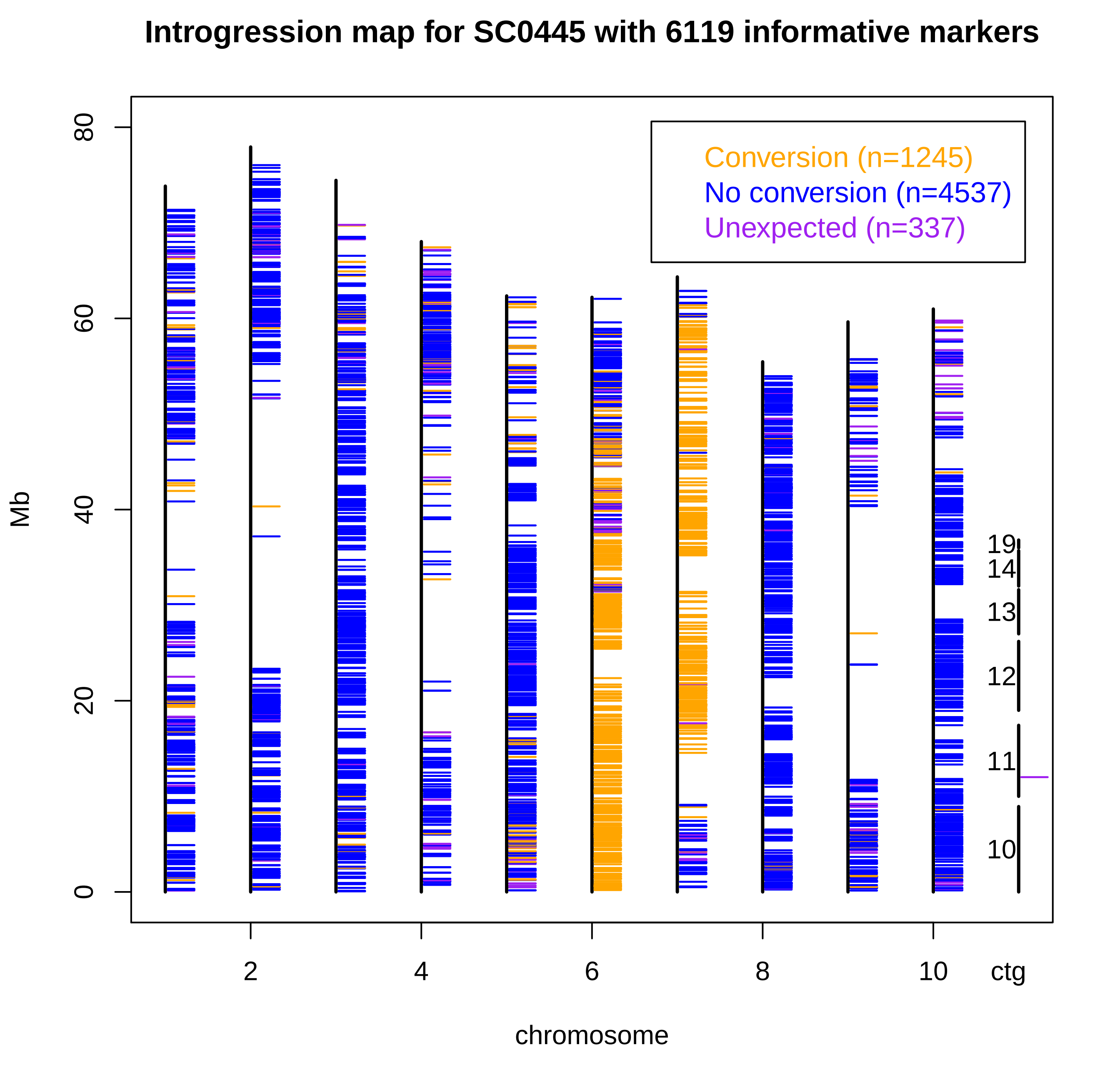

Introgression map for SC0445 with 6119 informative markers
Conversion (n=1245)
No conversion (n=4537)
Unexpected (n=337)
Mb
0
20
40
60
80
chromosome
2
4
6
8
10
ctg
19
14
13
12
11
10

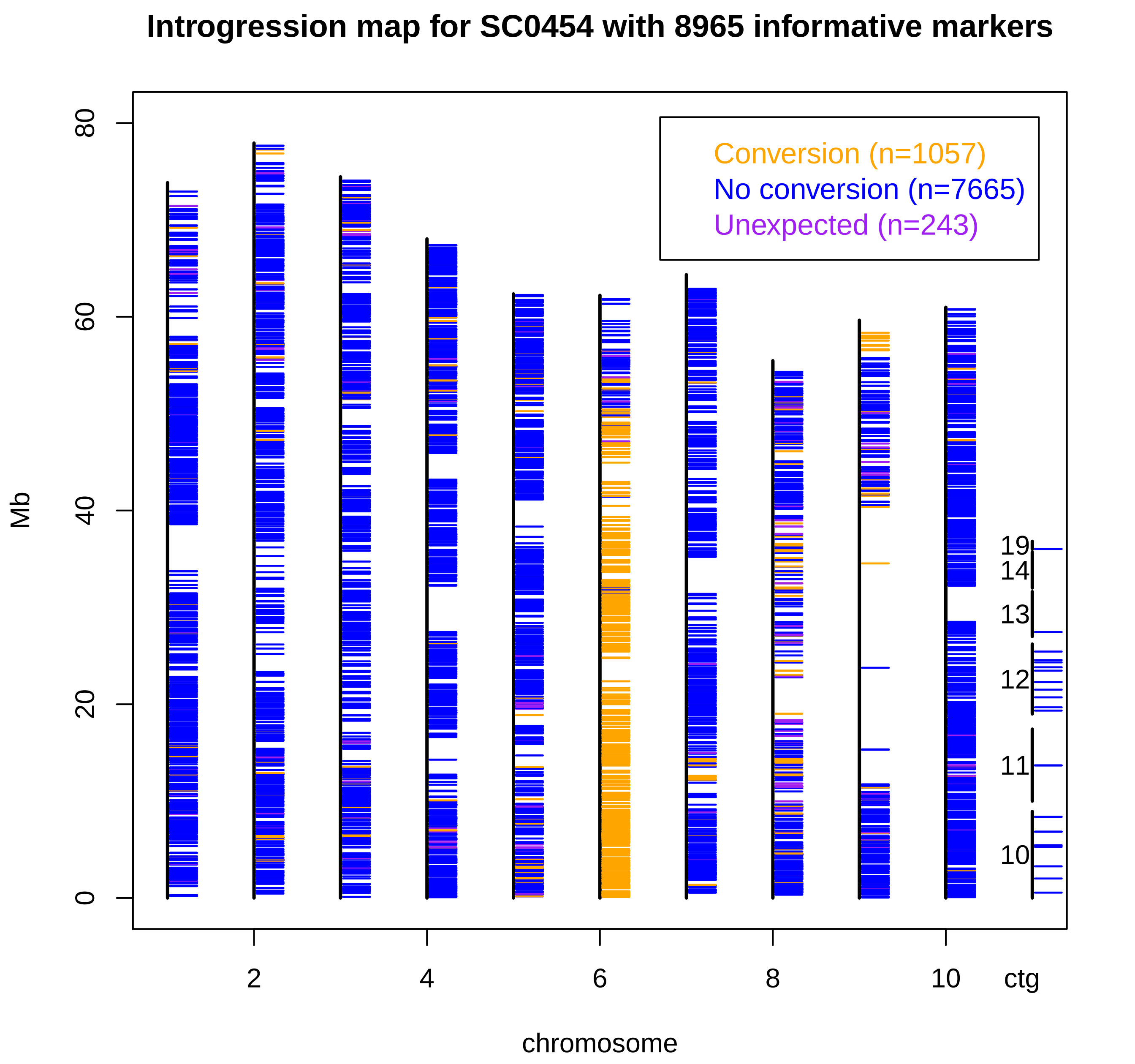

Introgression map for SC0454 with 8965 informative markers
Conversion (n=1057)
No conversion (n=7665)
Unexpected (n=243)
Mb
0
20
40
60
80
chromosome
2
4
6
8
10
ctg
19
14
13
12
11
10

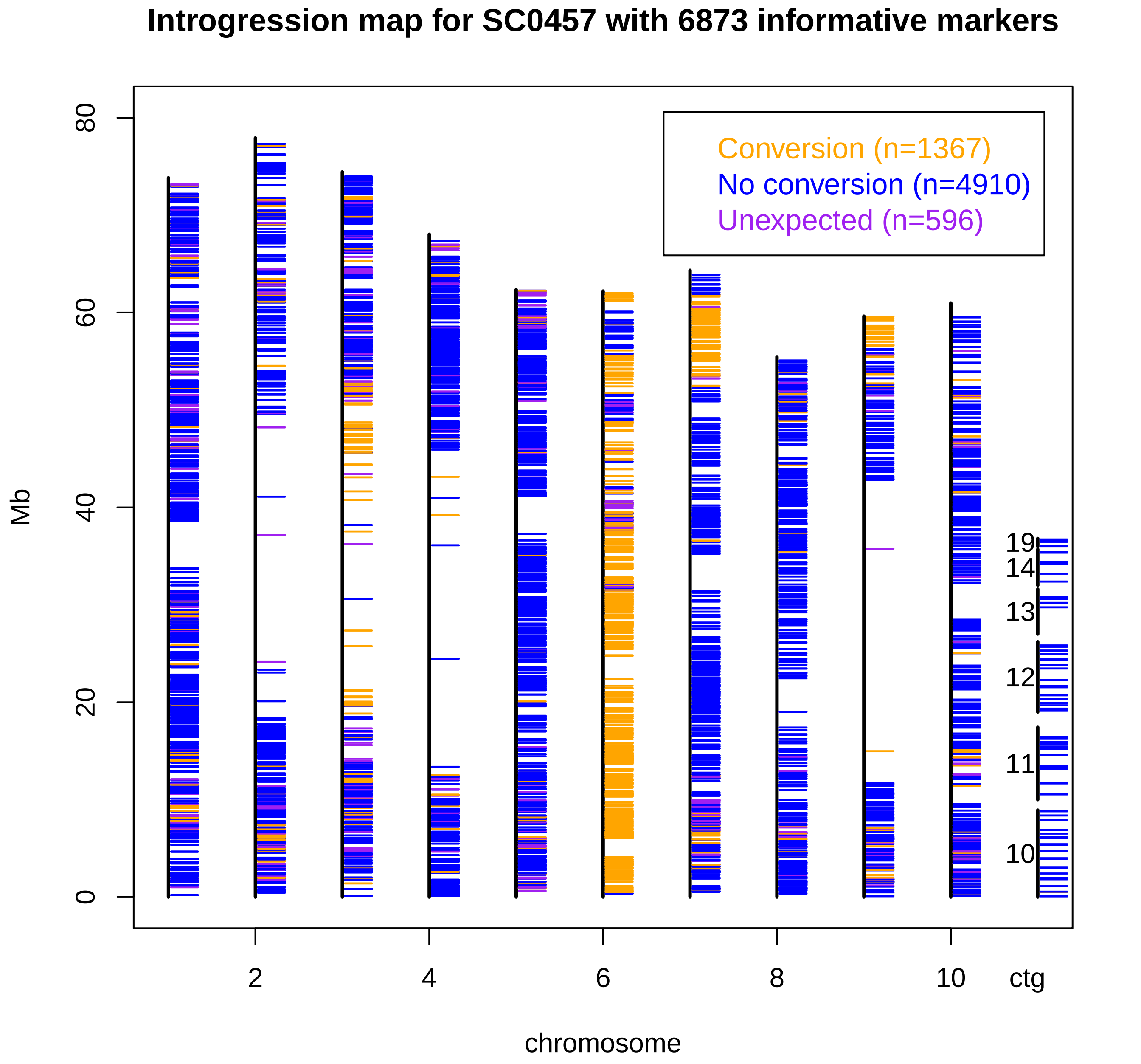
Introgression map for SC0457 with 6873 informative markers
Conversion (n=1367)
No conversion (n=4910)
Unexpected (n=596)
Mb
0
20
40
60
80
chromosome
2
4
6
8
10
ctg
19
14
13
12
11
10

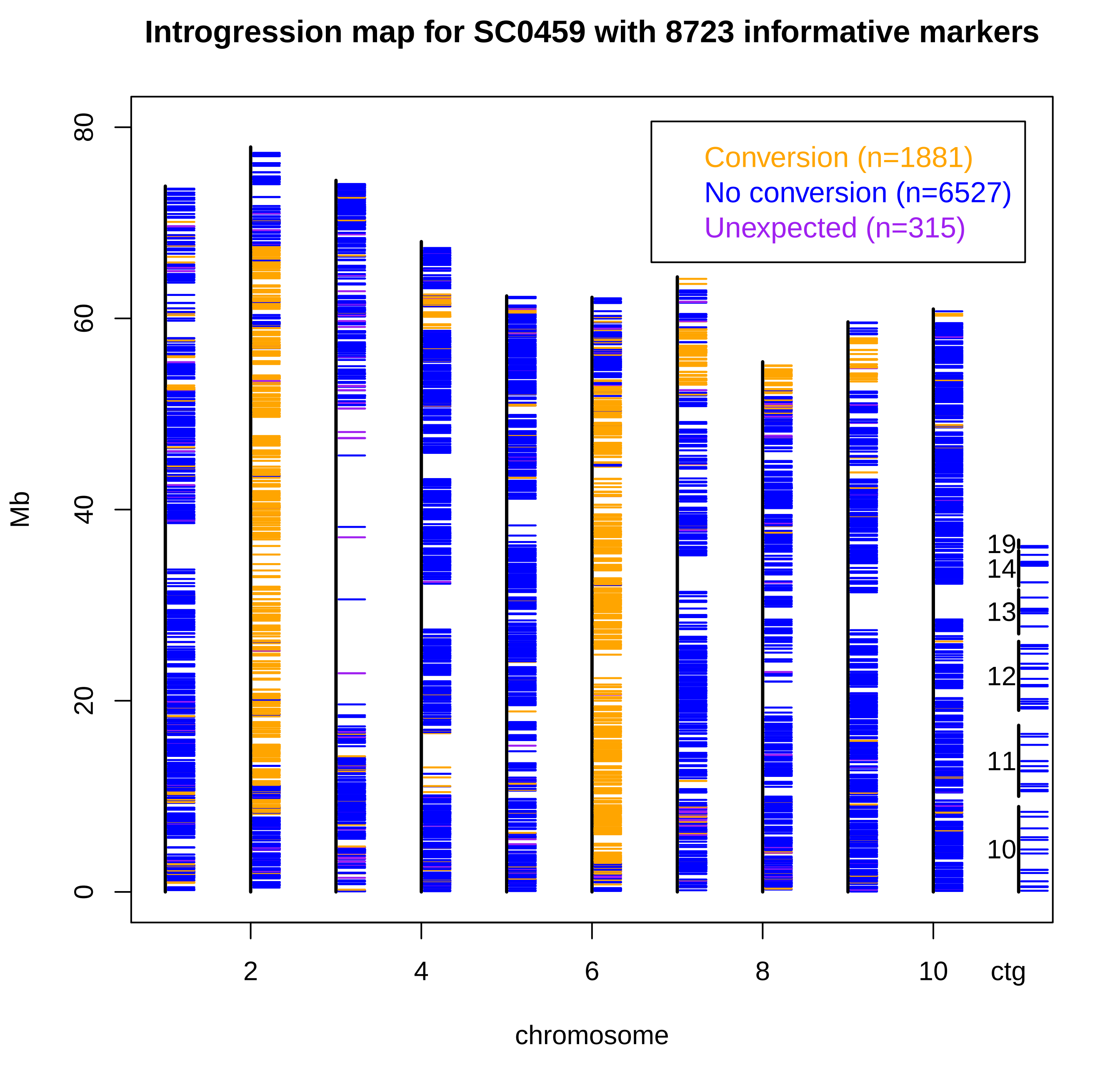

Introgression map for SC0459 with 8723 informative markers
Conversion (n=1881)
No conversion (n=6527)
Unexpected (n=315)
Mb
0
20
40
60
80
chromosome
2
4
6
8
10
ctg
19
14
13
12
11
10

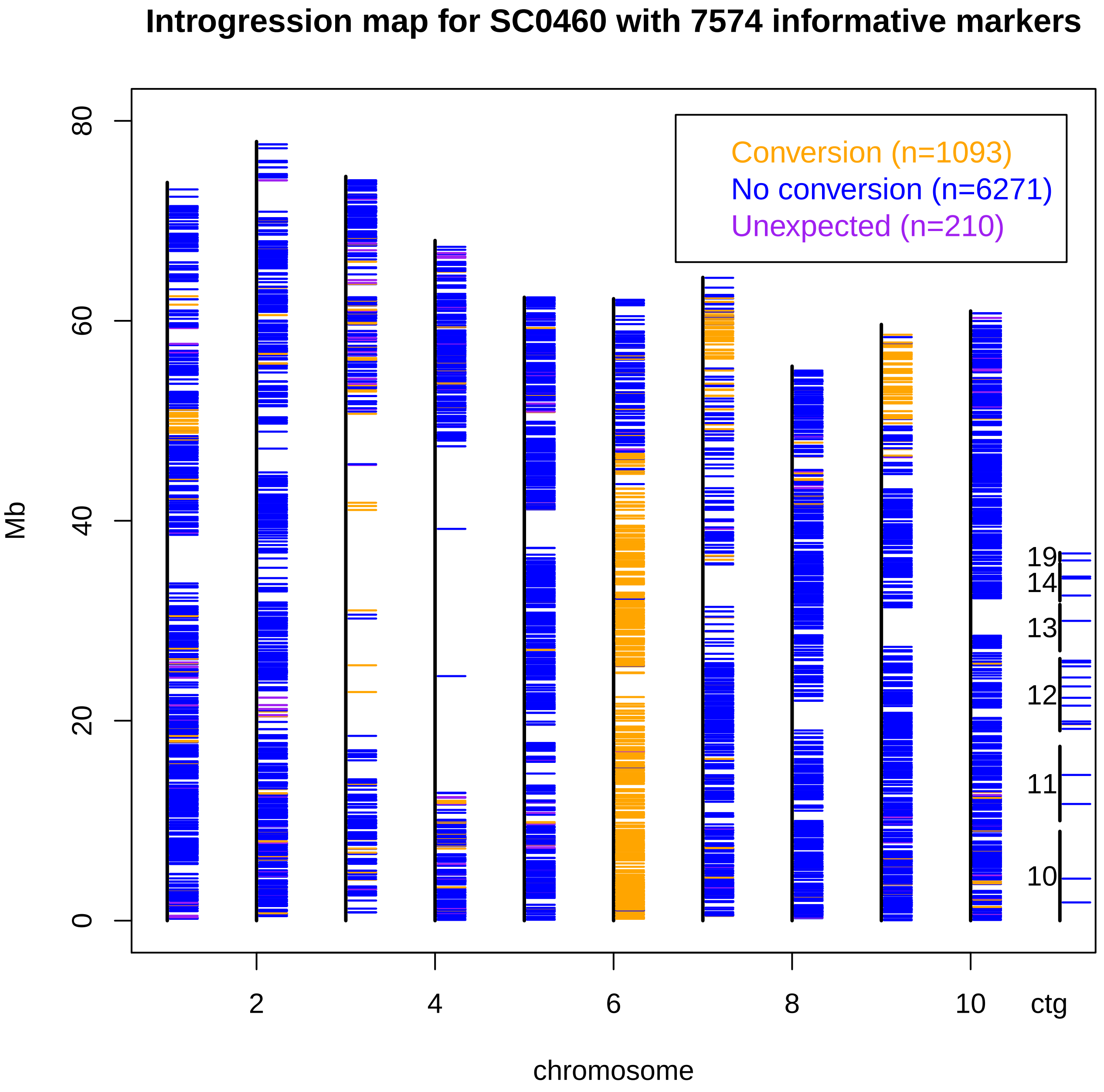
Introgression map for SC0460 with 7574 informative markers
Conversion (n=1093)
No conversion (n=6271)
Unexpected (n=210)
Mb
0
20
40
60
80
2
4
6
8
10
ctg
19
14
13
12
11
10
chromosome

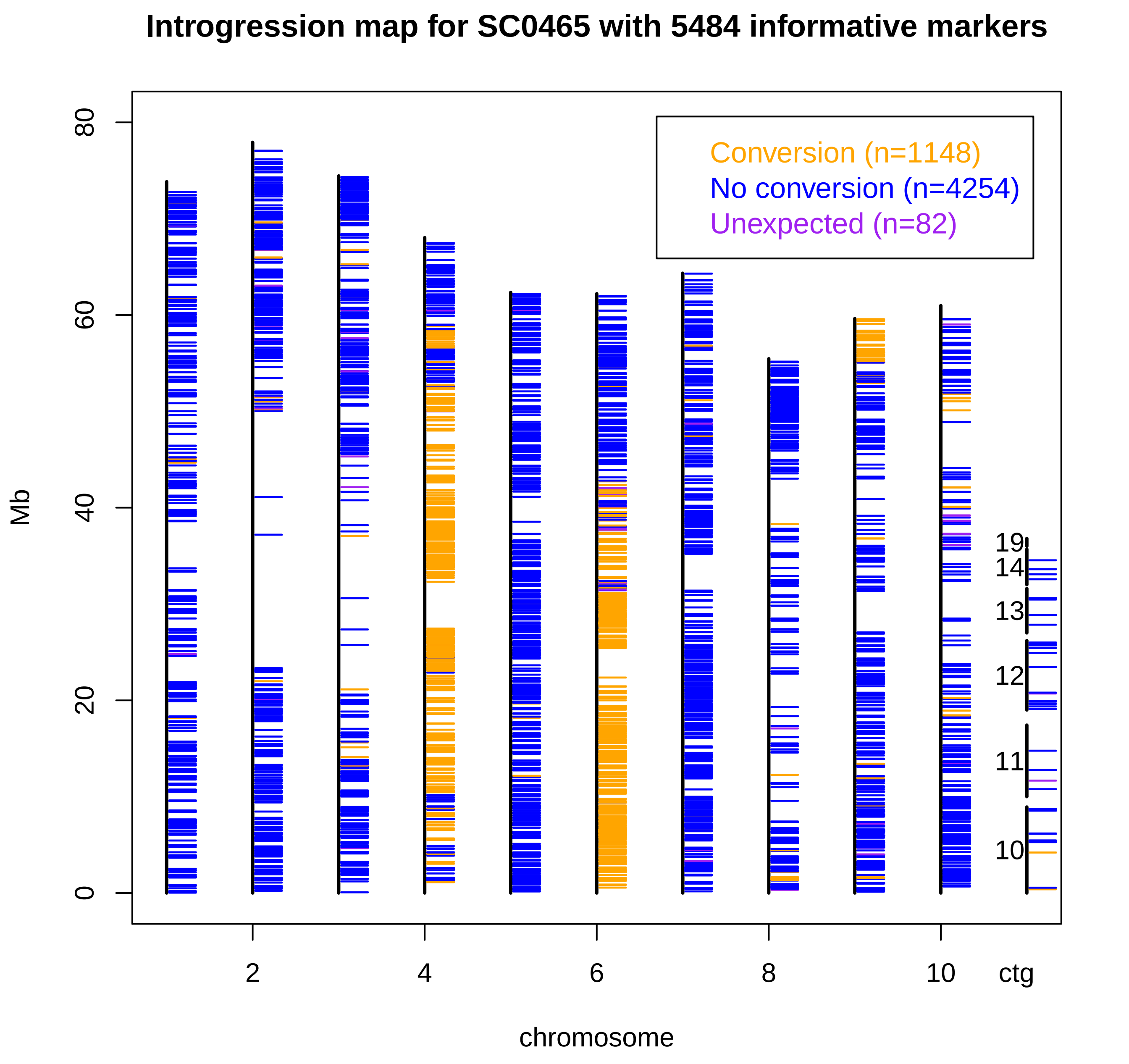

Introgression map for SC0465 with 5484 informative markers
Conversion (n=1148)
No conversion (n=4254)
Unexpected (n=82)
Mb
0
20
40
60
80
chromosome
2
4
6
8
10
ctg
19
14
13
12
11
10

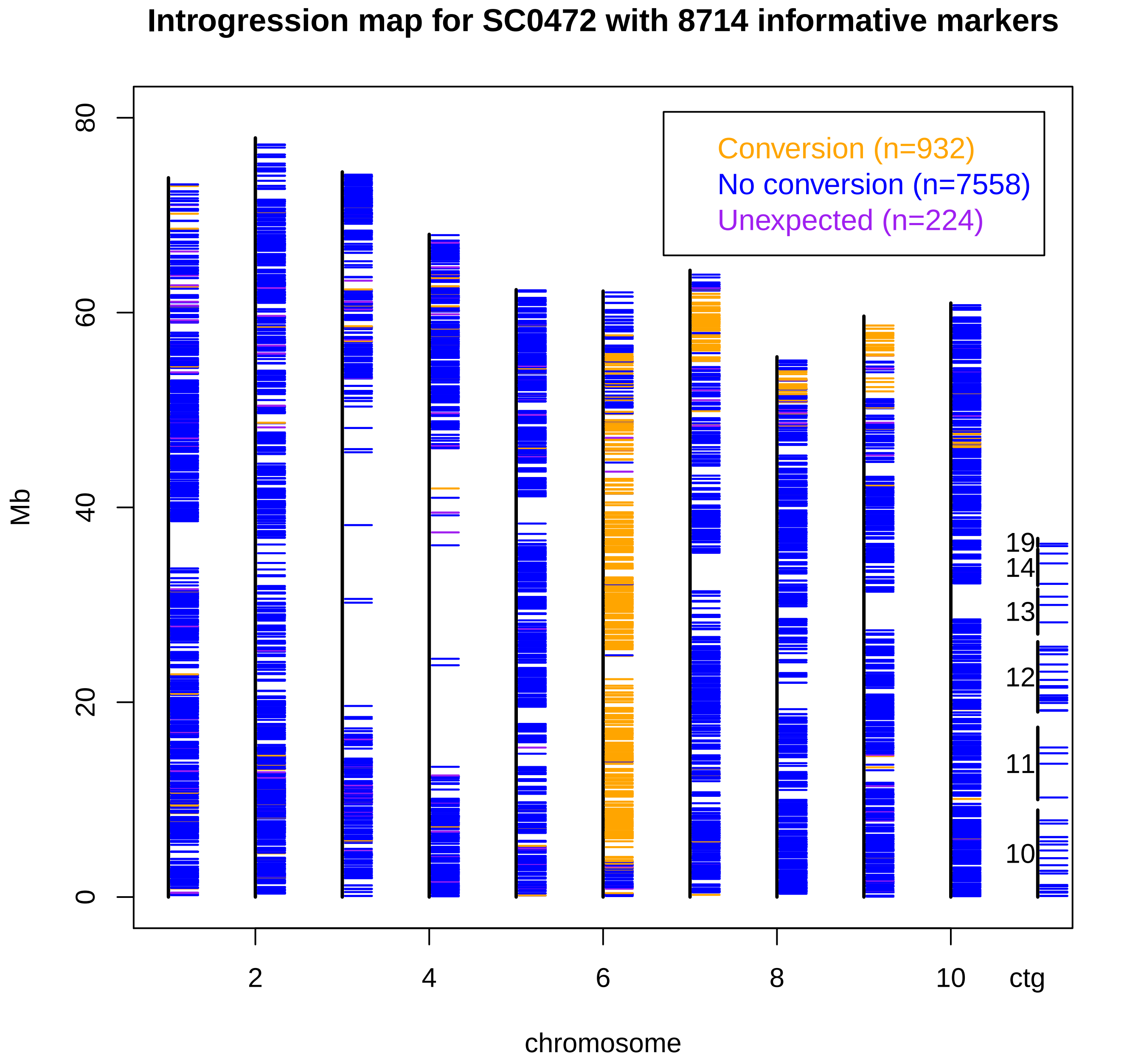

Introgression map for SC0472 with 8714 informative markers
Conversion (n=932)
No conversion (n=7558)
Unexpected (n=224)
Mb
0
20
40
60
80
chromosome
2
4
6
8
10
ctg
19
14
13
12
11
10

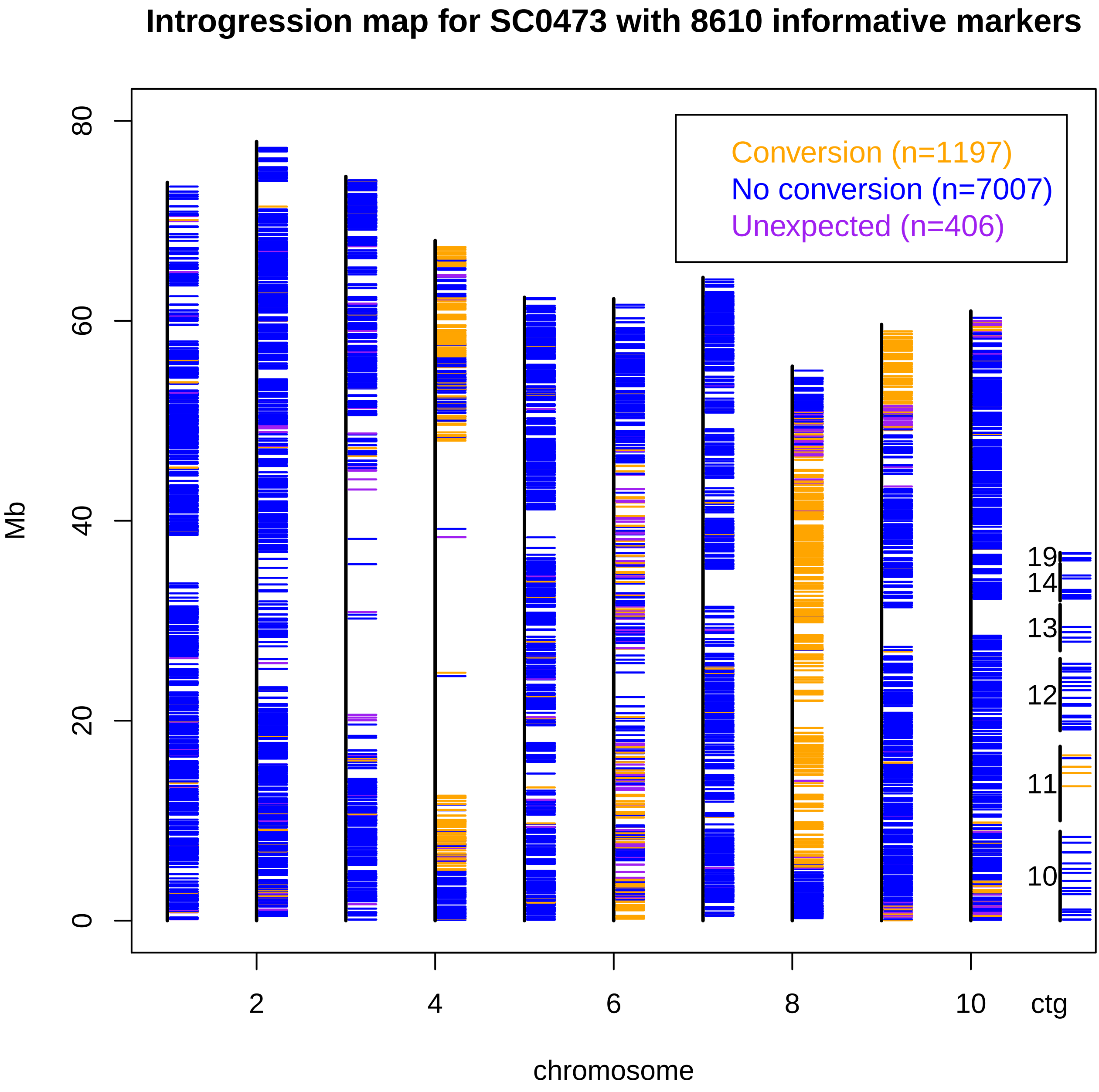
Introgression map for SC0473 with 8610 informative markers
Conversion (n=1197)
No conversion (n=7007)
Unexpected (n=406)
Mb
0
20
40
60
80
chromosome
2
4
6
8
10
ctg
19
14
13
12
11
10

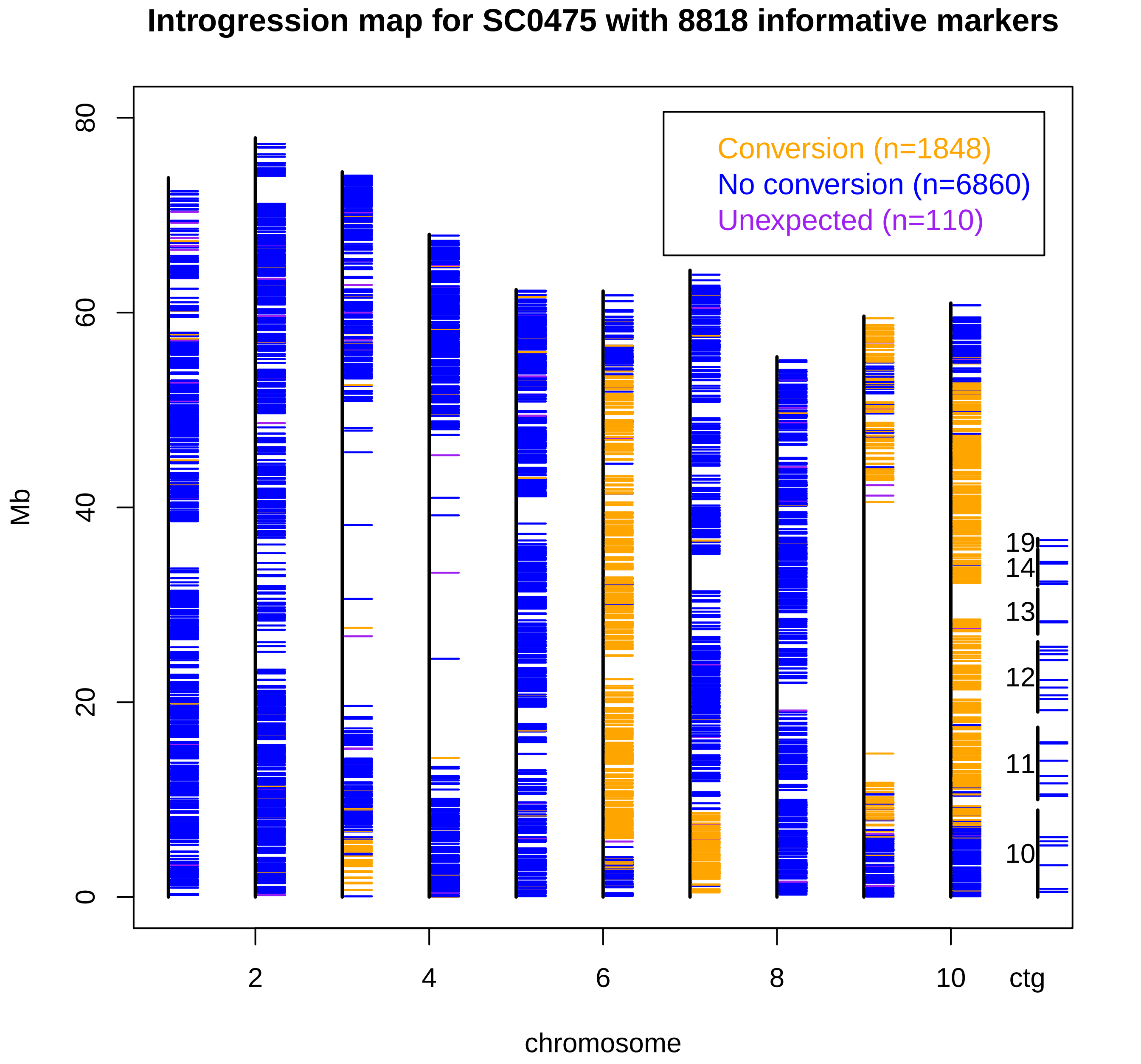

Introgression map for SC0475 with 8818 informative markers
Conversion (n=1848)
No conversion (n=6860)
Unexpected (n=110)
Mb
0
20
40
60
80
2
4
6
8
10
ctg
19
14
13
12
11
10
chromosome

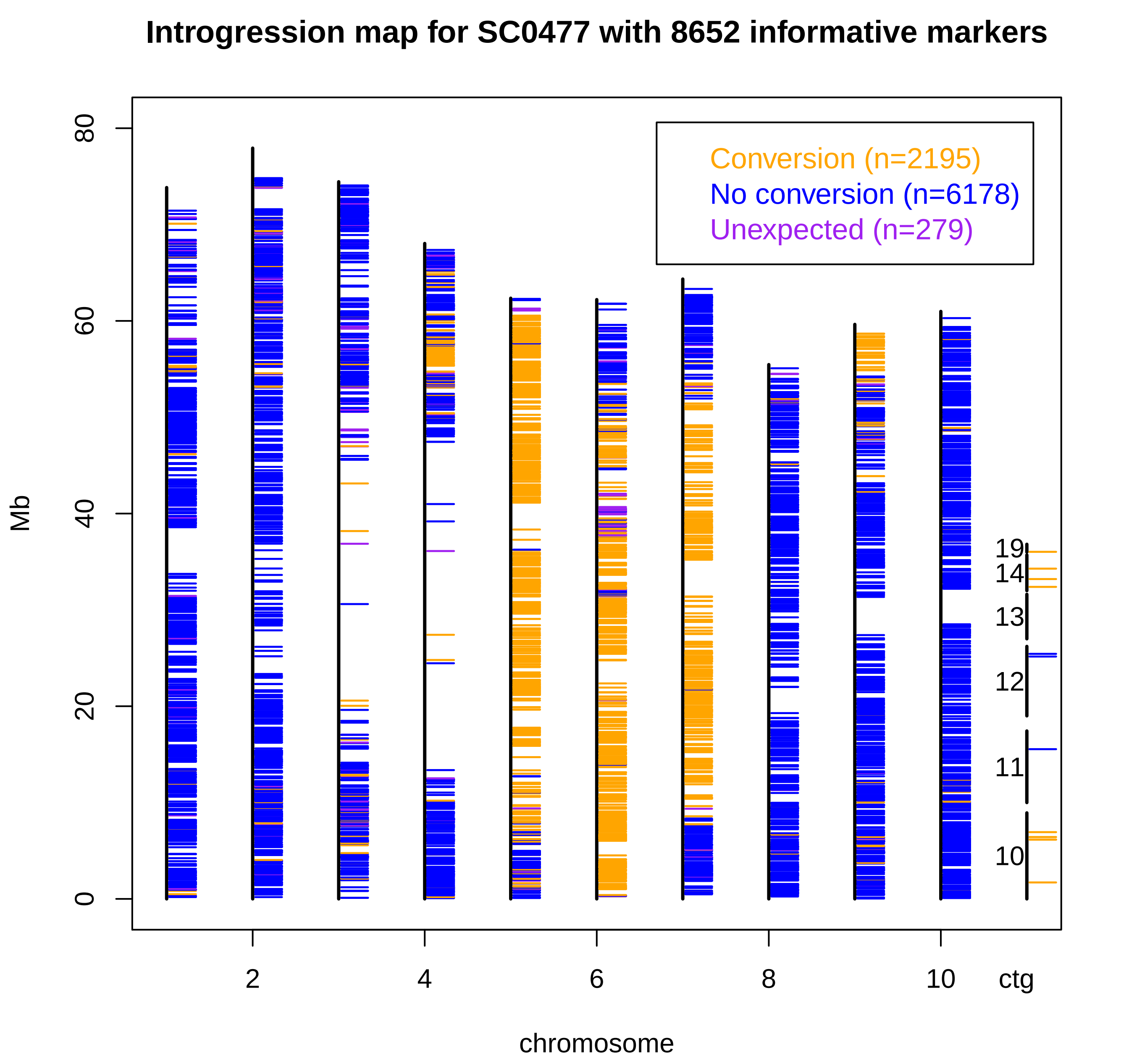
Introgression map for SC0477 with 8652 informative markers
Conversion (n=2195)
No conversion (n=6178)
Unexpected (n=279)
Mb
0
20
40
60
80
chromosome
2
4
6
8
10
ctg
19
14
13
12
11
10

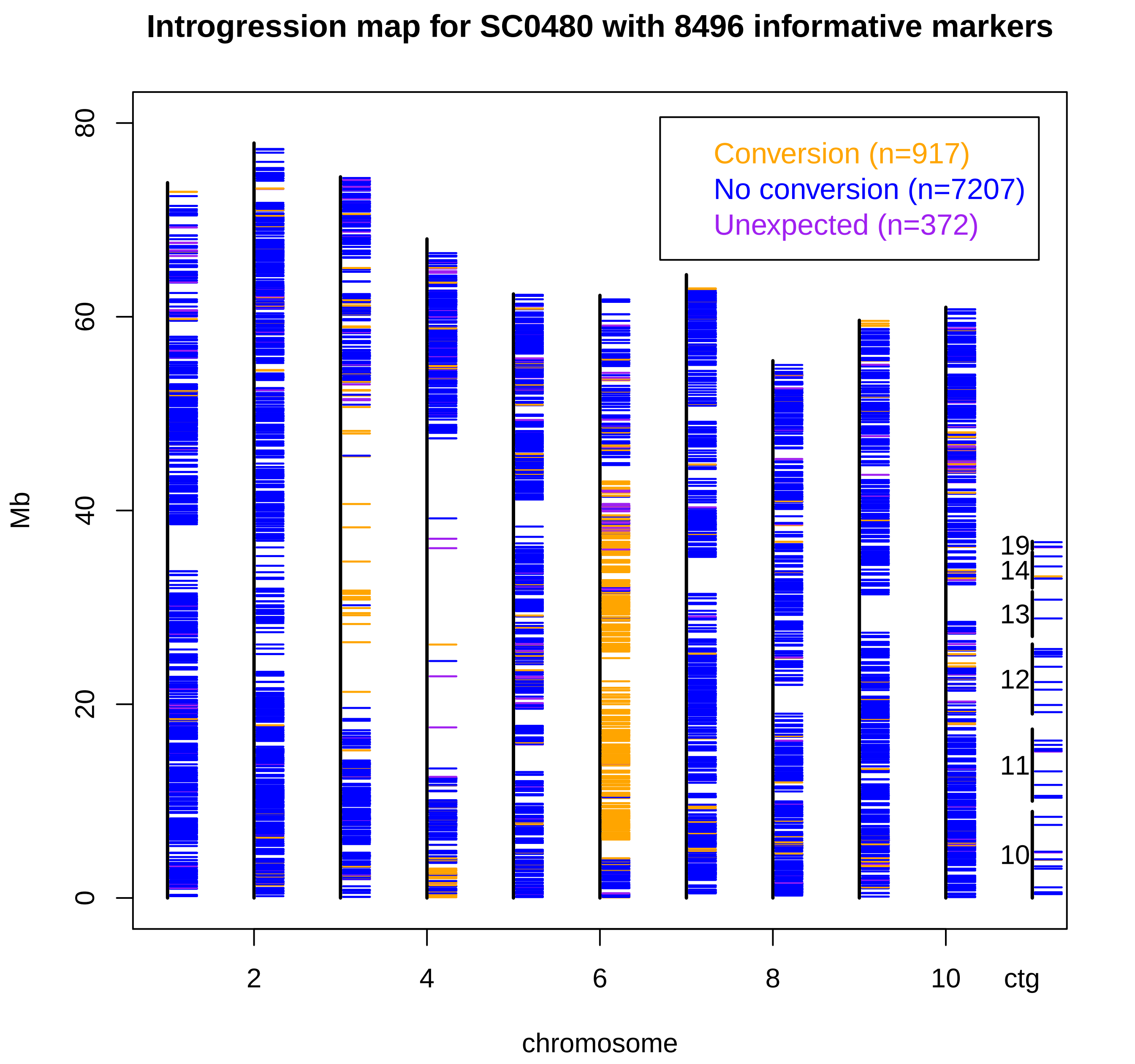

Introgression map for SC0480 with 8496 informative markers
Conversion (n=917)
No conversion (n=7207)
Unexpected (n=372)
Mb
0
20
40
60
80
chromosome
2
4
6
8
10
ctg
19
14
13
12
11
10

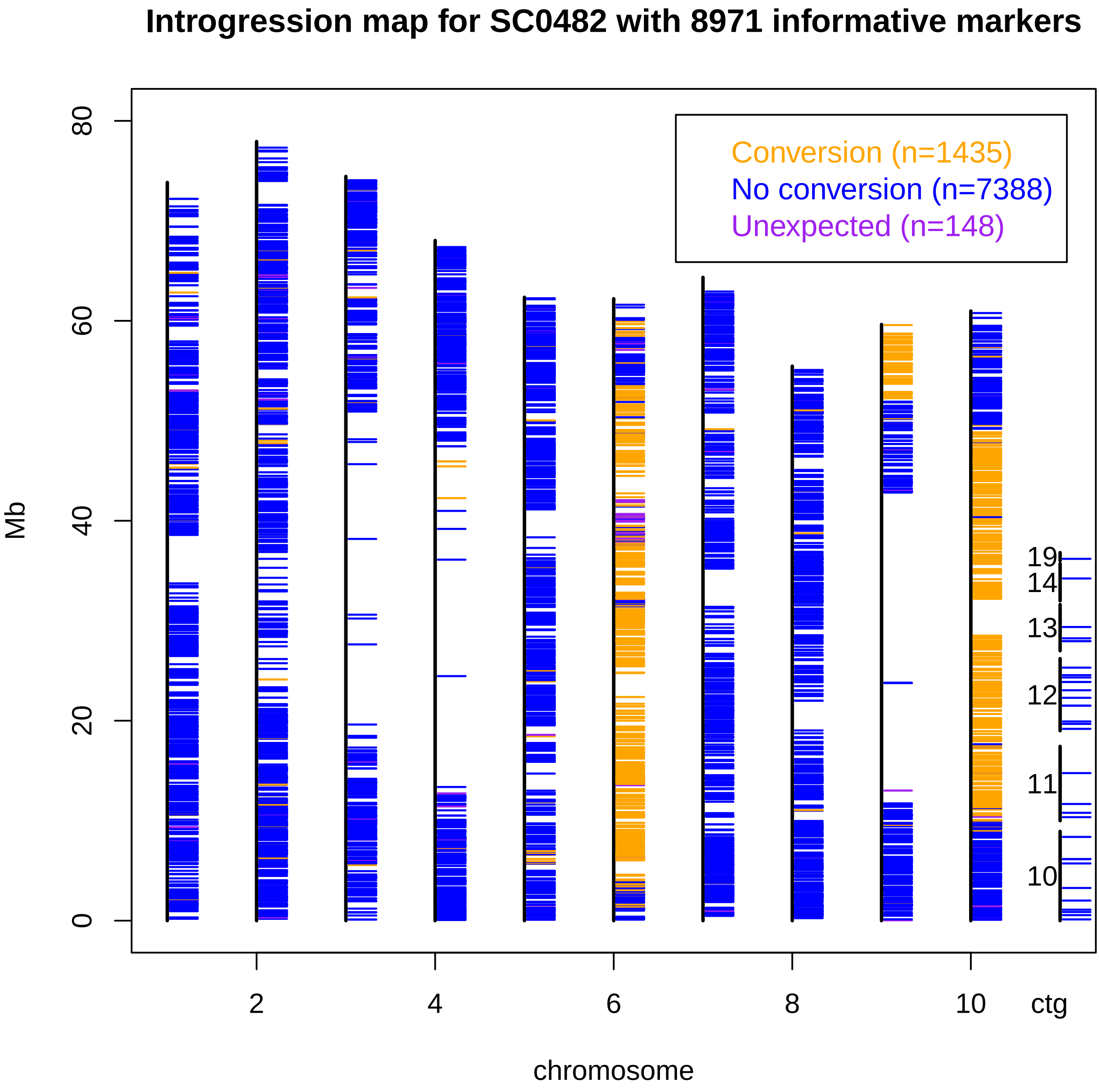
Introgression map for SC0482 with 8971 informative markers
Conversion (n=1435)
No conversion (n=7388)
Unexpected (n=148)
Mb
0
20
40
60
80
chromosome
2
4
6
8
10
ctg
19
14
13
12
11
10

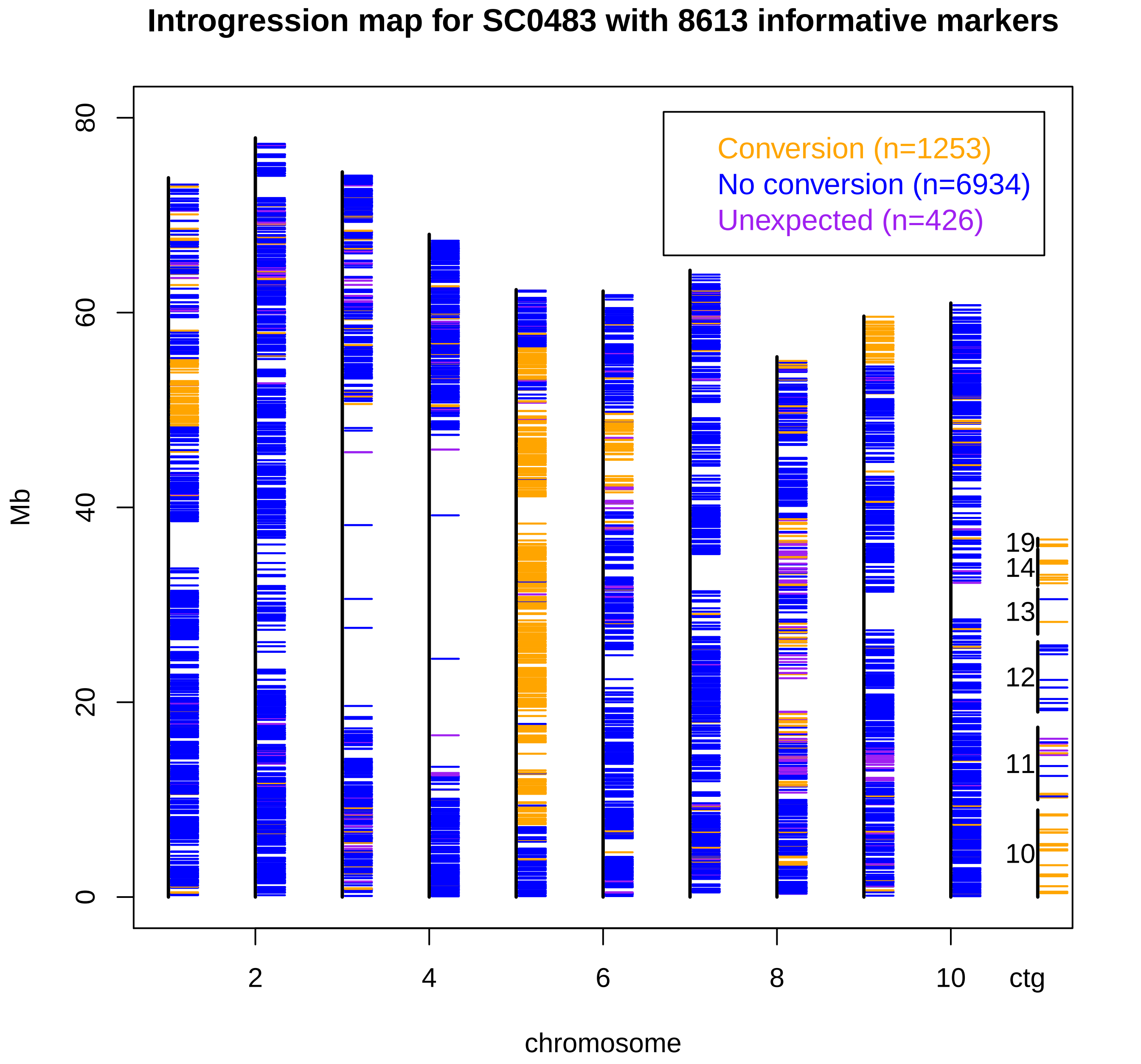

Introgression map for SC0483 with 8613 informative markers
Conversion (n=1253)
No conversion (n=6934)
Unexpected (n=426)
Mb
0
20
40
60
80
chromosome
2
4
6
8
10
ctg
19
14
13
12
11
10

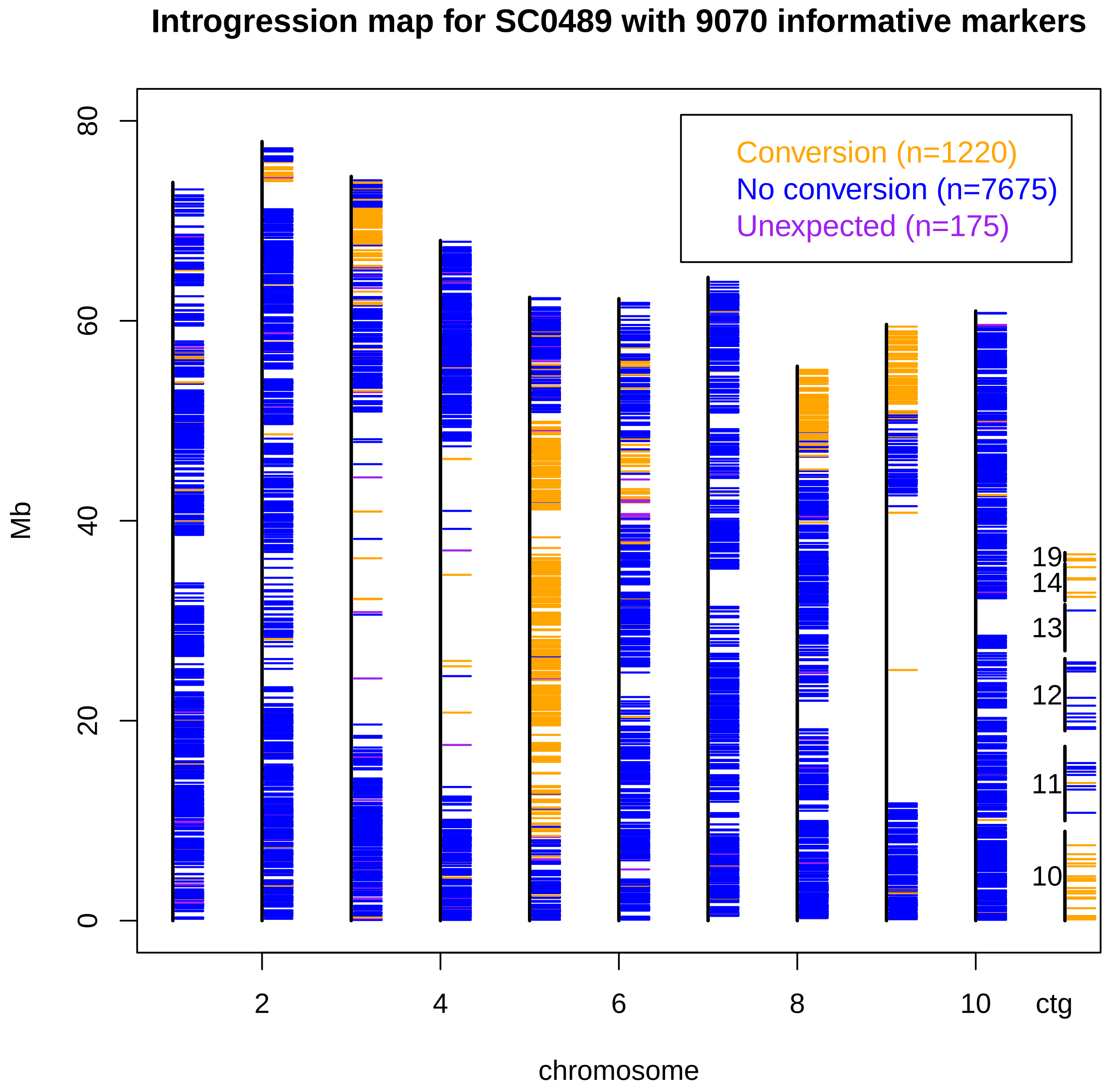

Introgression map for SC0489 with 9070 informative markers
Conversion (n=1220)
No conversion (n=7675)
Unexpected (n=175)
Mb
0
20
40
60
80
2
4
6
8
10
ctg
chromosome
19
14
13
12
11
10

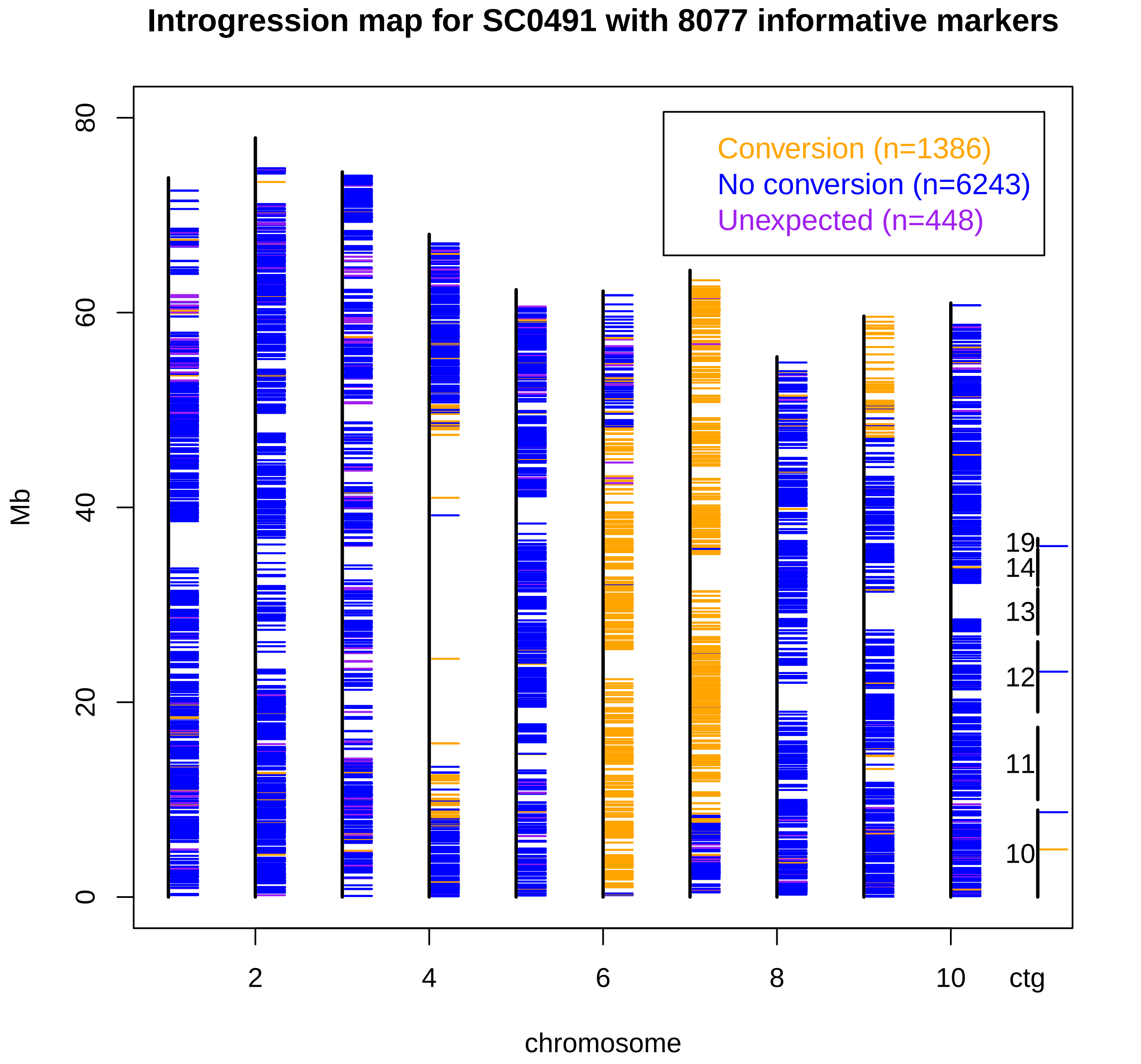

Introgression map for SC0491 with 8077 informative markers
Conversion (n=1386)
No conversion (n=6243)
Unexpected (n=448)
Mb
0
20
40
60
80
2
4
6
8
10
ctg
19
14
13
12
11
10
chromosome

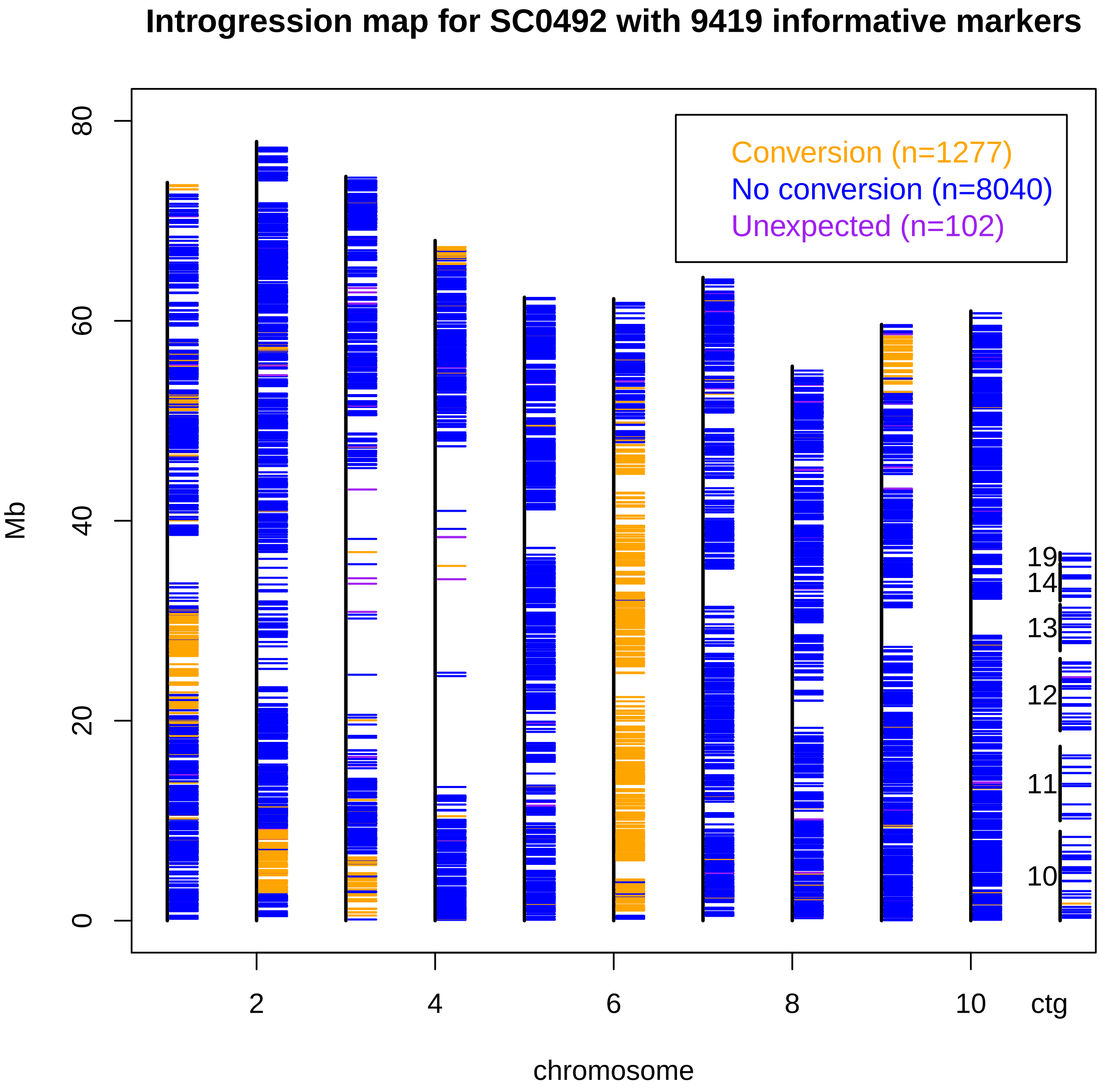

Introgression map for SC0492 with 9419 informative markers
Conversion (n=1277)
No conversion (n=8040)
Unexpected (n=102)
Mb
0
20
40
60
80
chromosome
2
4
6
8
10
ctg
19
14
13
12
11
10

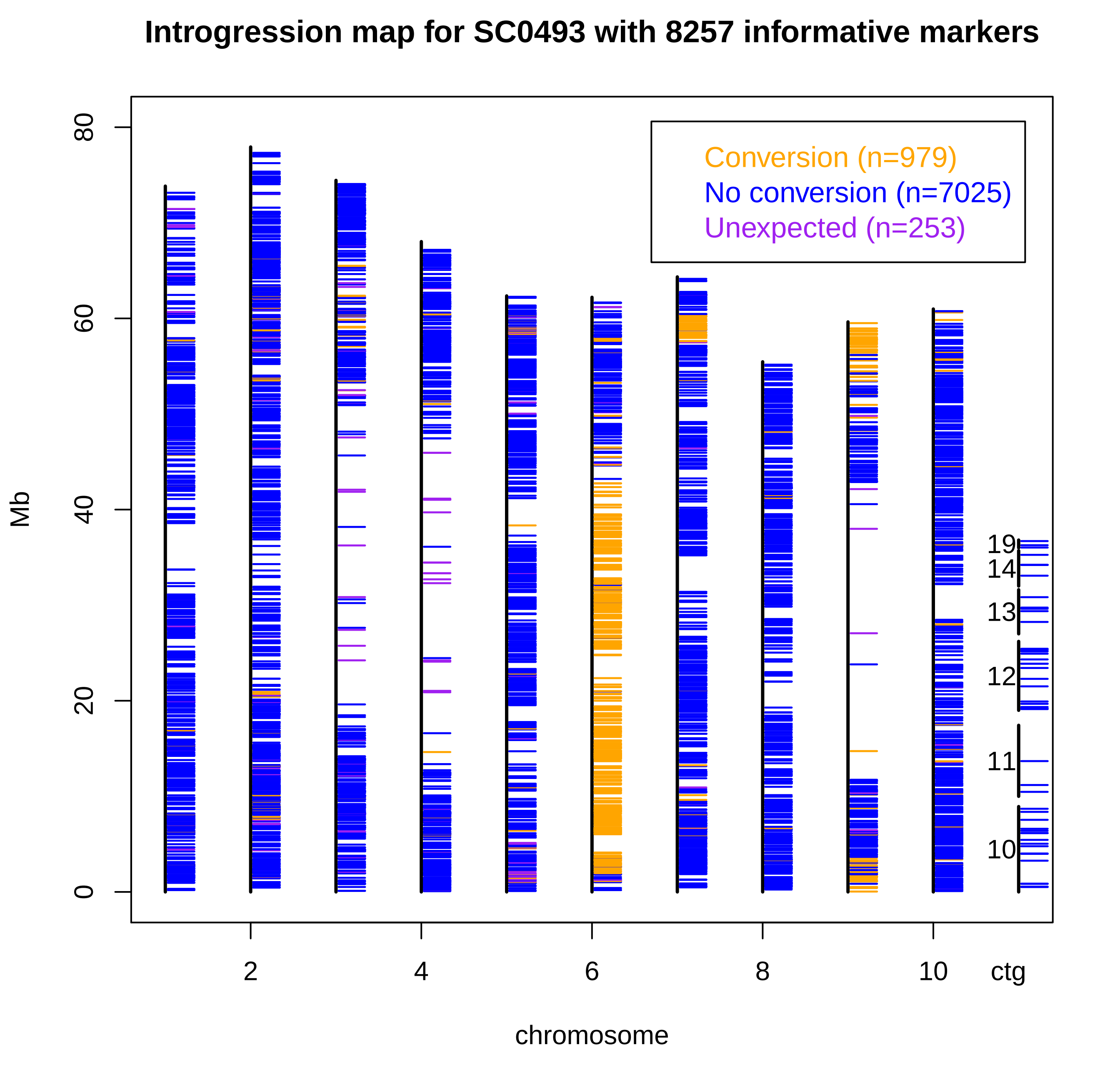

Introgression map for SC0493 with 8257 informative markers
Conversion (n=979)
No conversion (n=7025)
Unexpected (n=253)
Mb
0
20
40
60
80
2
4
6
8
10
ctg
19
14
13
12
11
10
chromosome

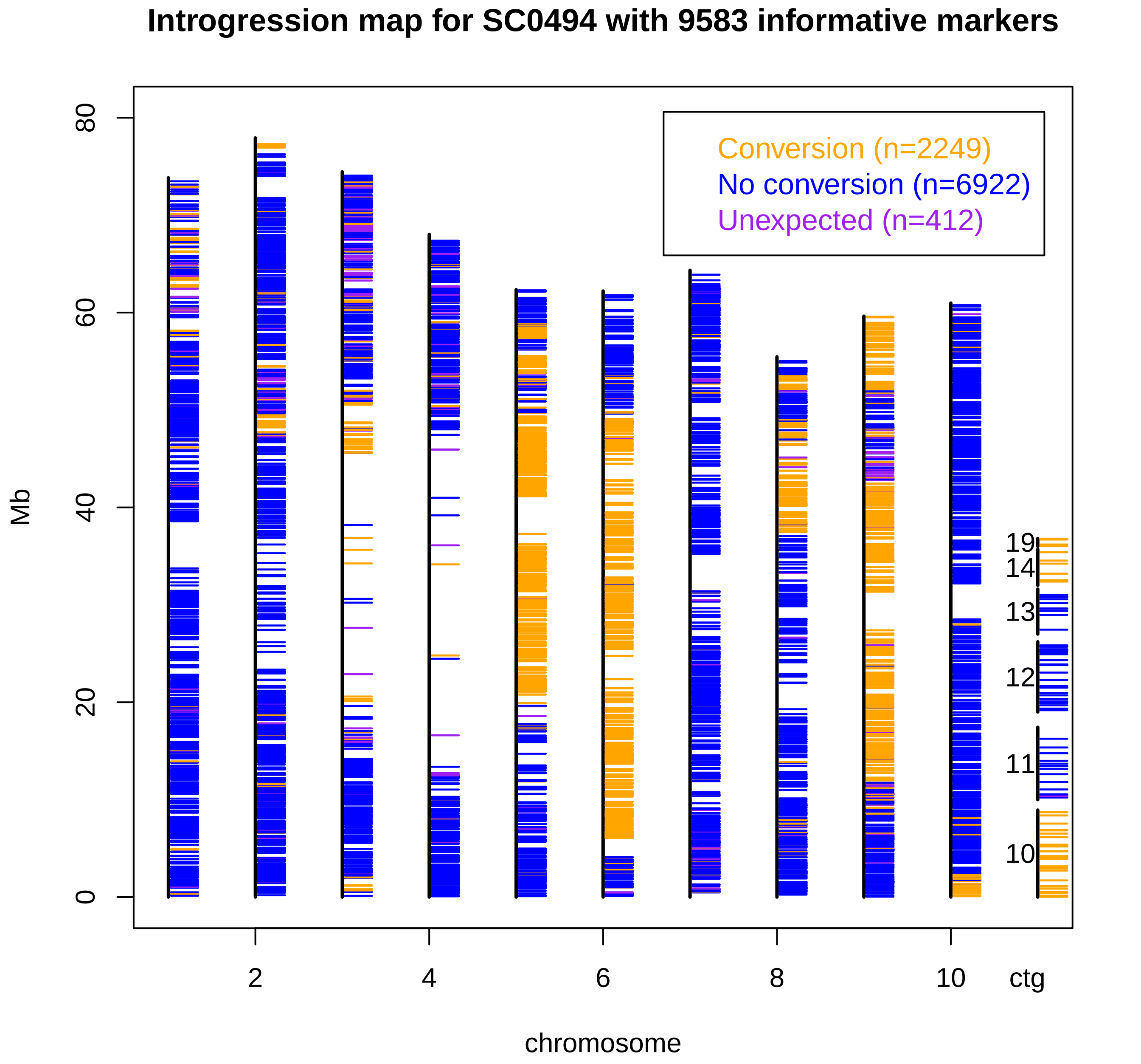

Introgression map for SC0494 with 9583 informative markers
Conversion (n=2249)
No conversion (n=6922)
Unexpected (n=412)
Mb
0
20
40
60
80
chromosome
2
4
6
8
10
ctg
19
14
13
12
11
10

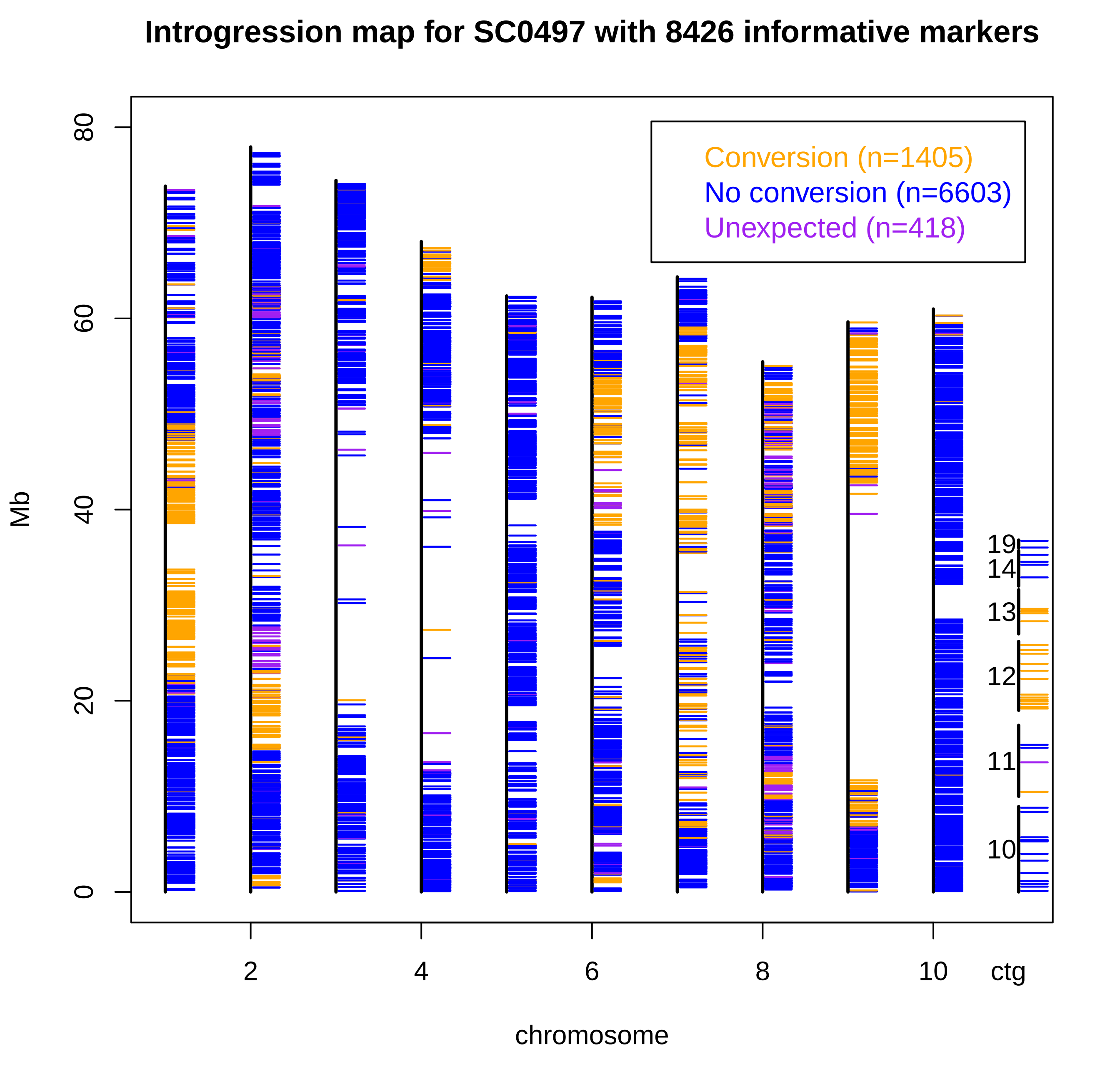

Introgression map for SC0497 with 8426 informative markers
Conversion (n=1405)
No conversion (n=6603)
Unexpected (n=418)
Mb
0
20
40
60
80
2
4
6
8
10
ctg
19
14
13
12
11
10
chromosome

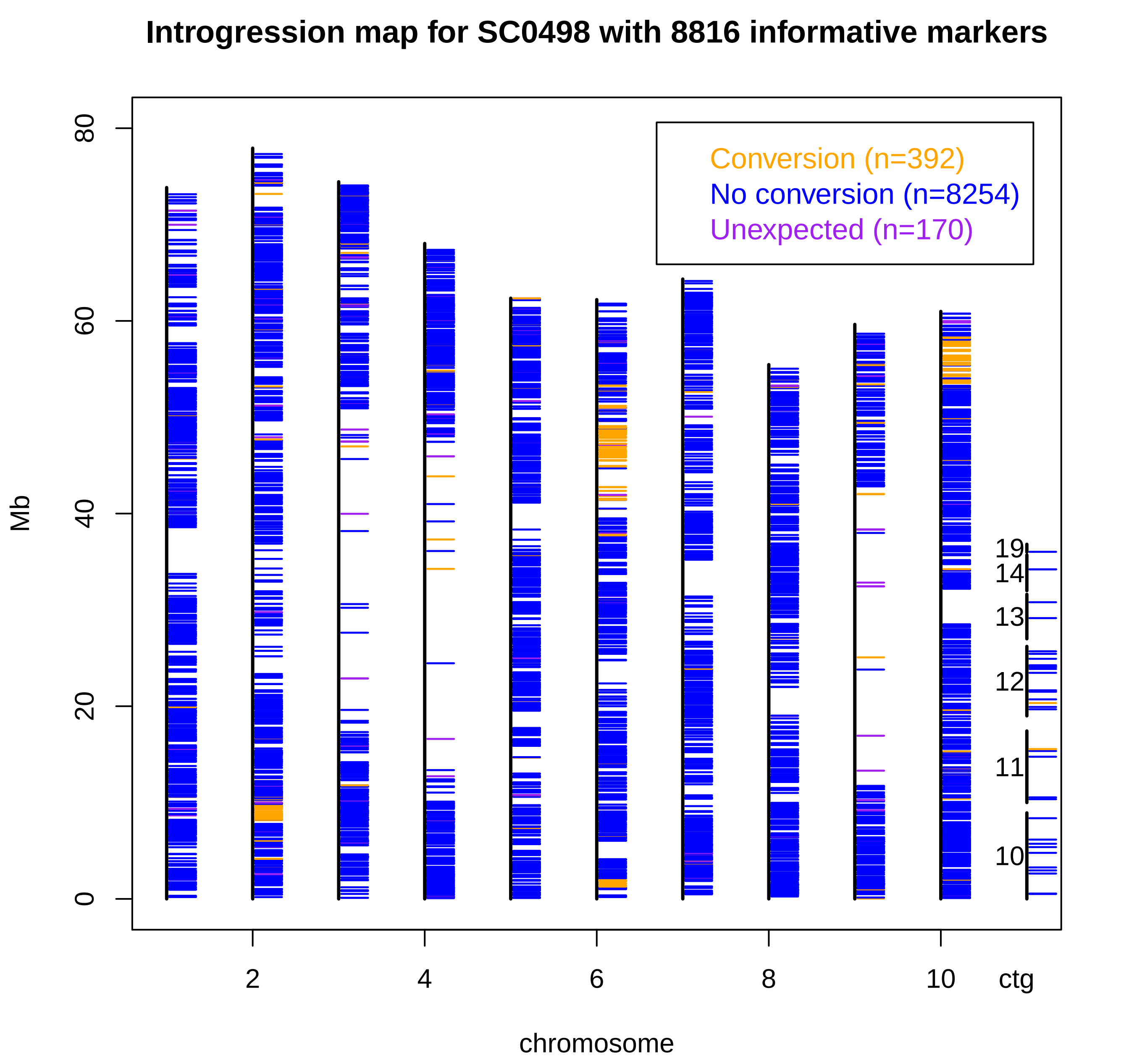

Introgression map for SC0498 with 8816 informative markers
Conversion (n=392)
No conversion (n=8254)
Unexpected (n=170)
Mb
0
20
40
60
80
chromosome
2
4
6
8
10
ctg
19
14
13
12
11
10

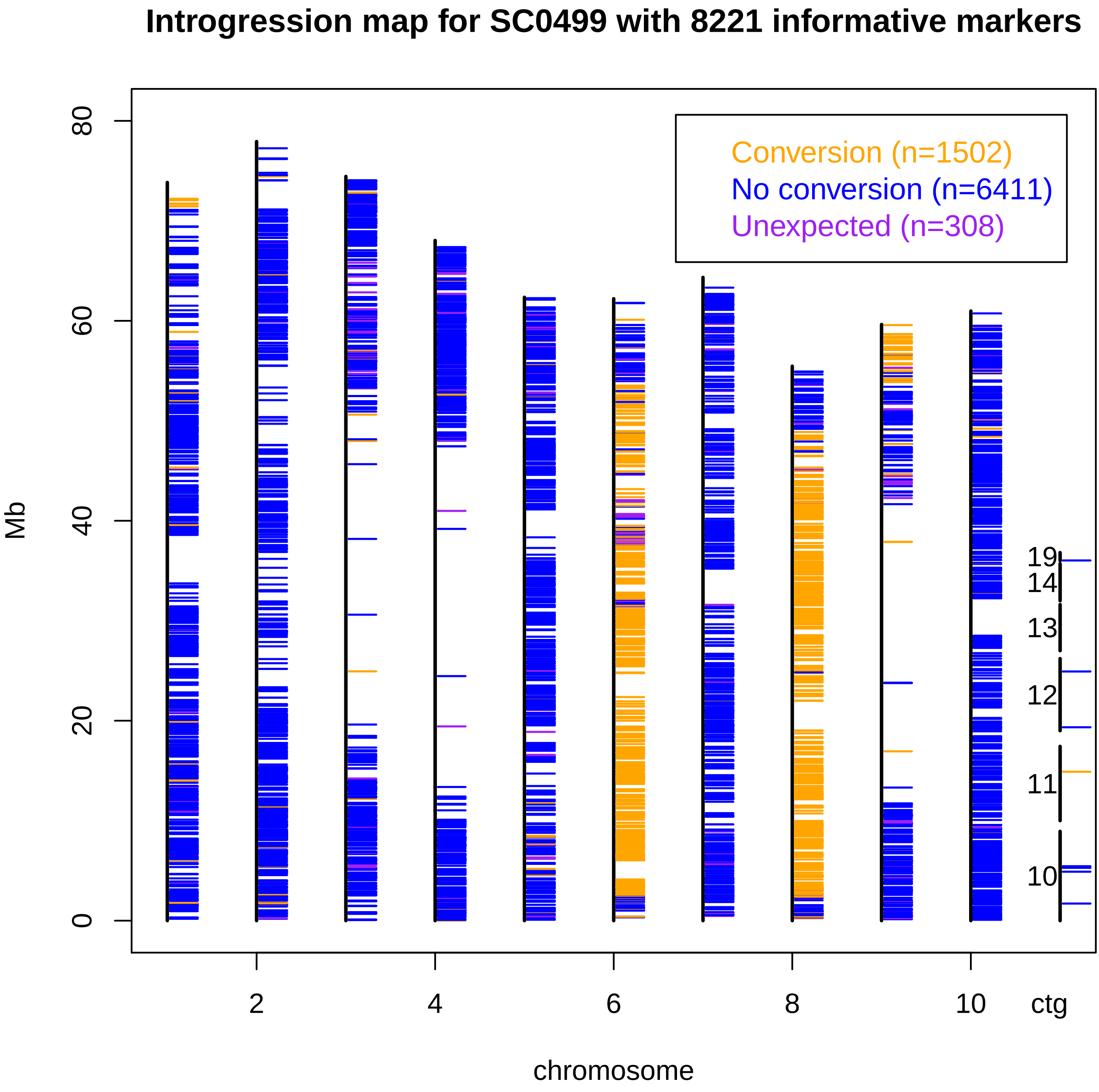

Introgression map for SC0499 with 8221 informative markers
Conversion (n=1502)
No conversion (n=6411)
Unexpected (n=308)
Mb
0
20
40
60
80
chromosome
2
4
6
8
10
ctg
19
14
13
12
11
10

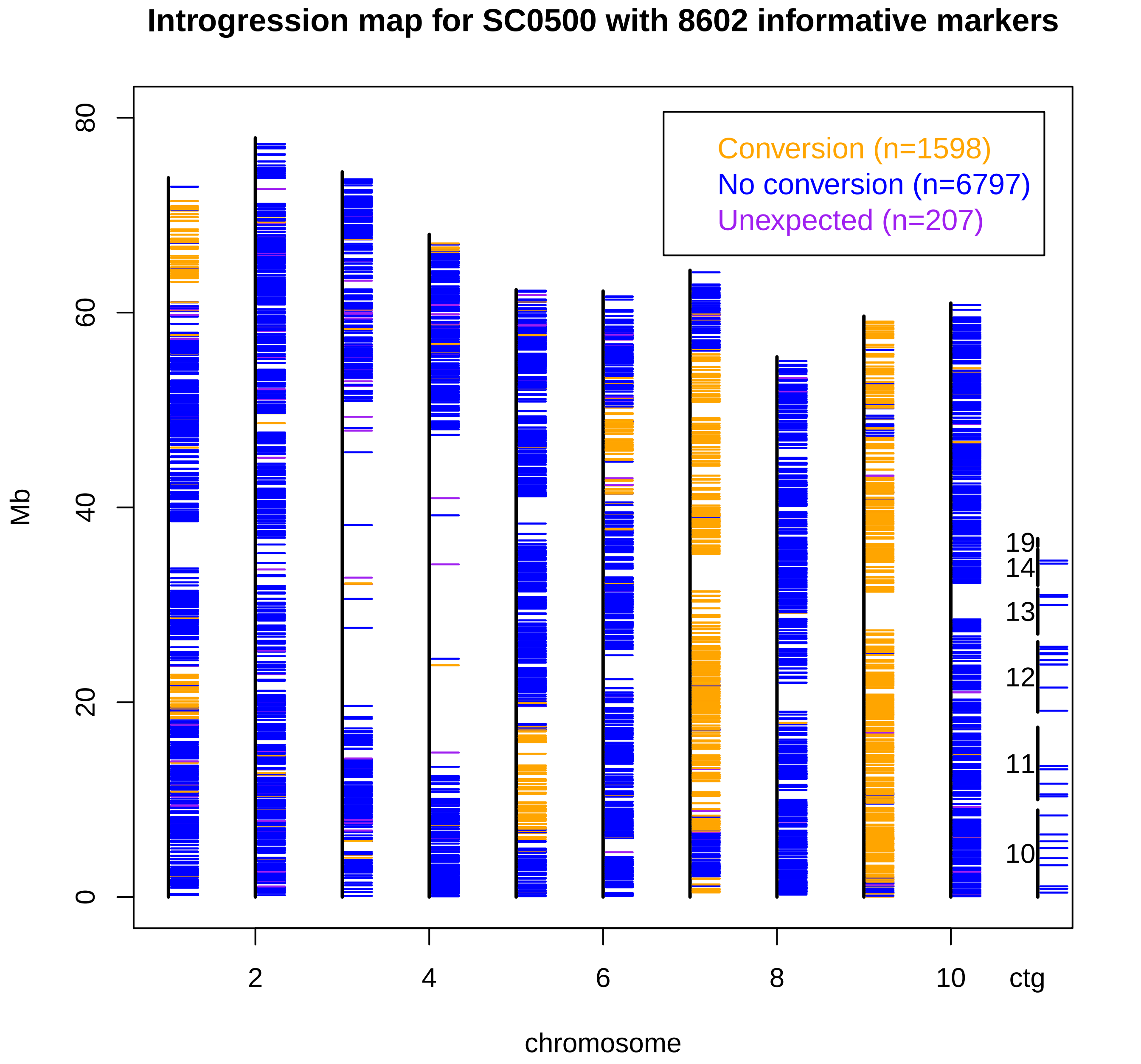

Introgression map for SC0500 with 8602 informative markers
Conversion (n=1598)
No conversion (n=6797)
Unexpected (n=207)
Mb
0
20
40
60
80
chromosome
2
4
6
8
10
ctg
19
14
13
12
11
10

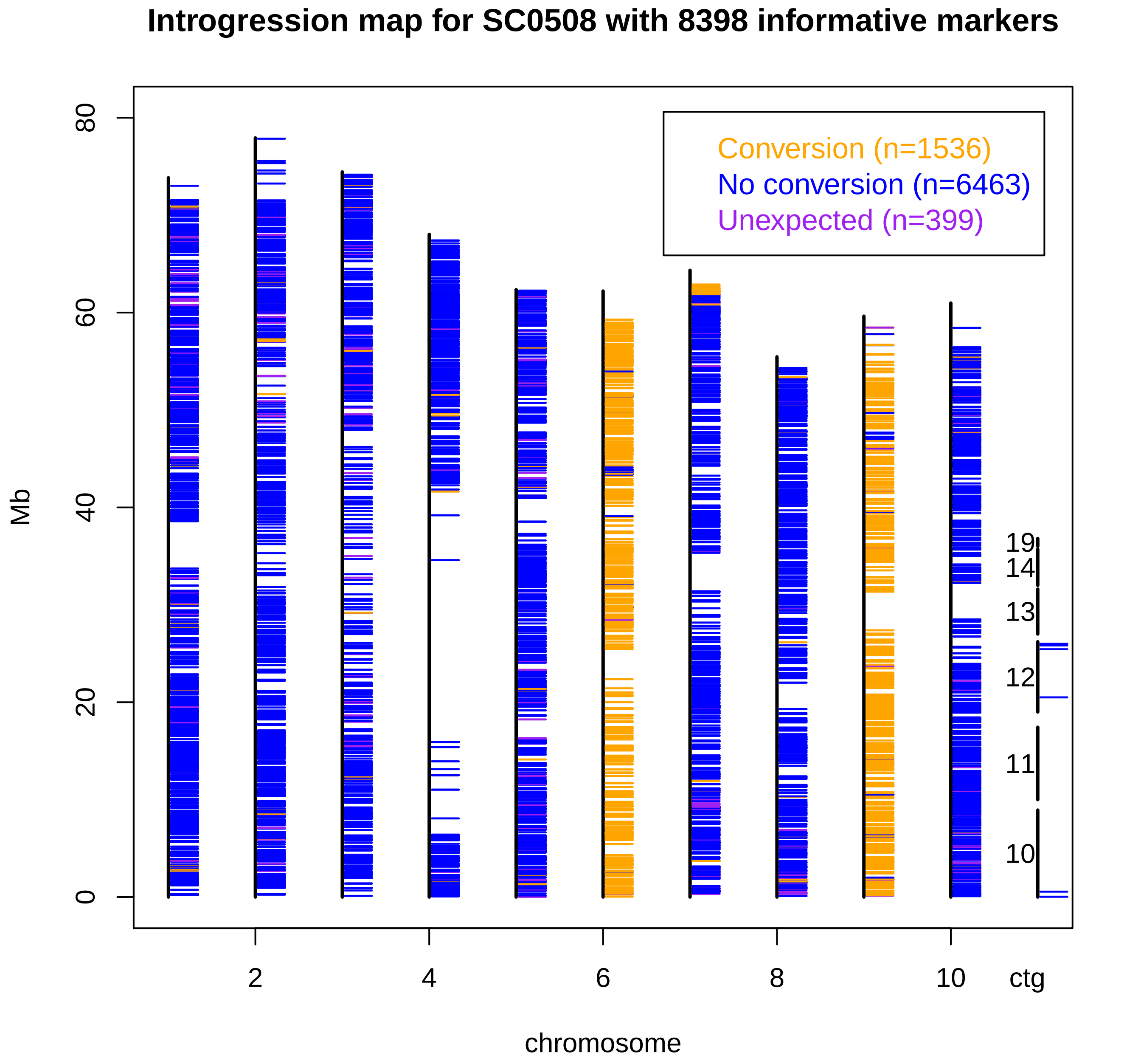

Introgression map for SC0508 with 8398 informative markers
Conversion (n=1536)
No conversion (n=6463)
Unexpected (n=399)
Mb
0
20
40
60
80
chromosome
2
4
6
8
10
ctg
19
14
13
12
11
10

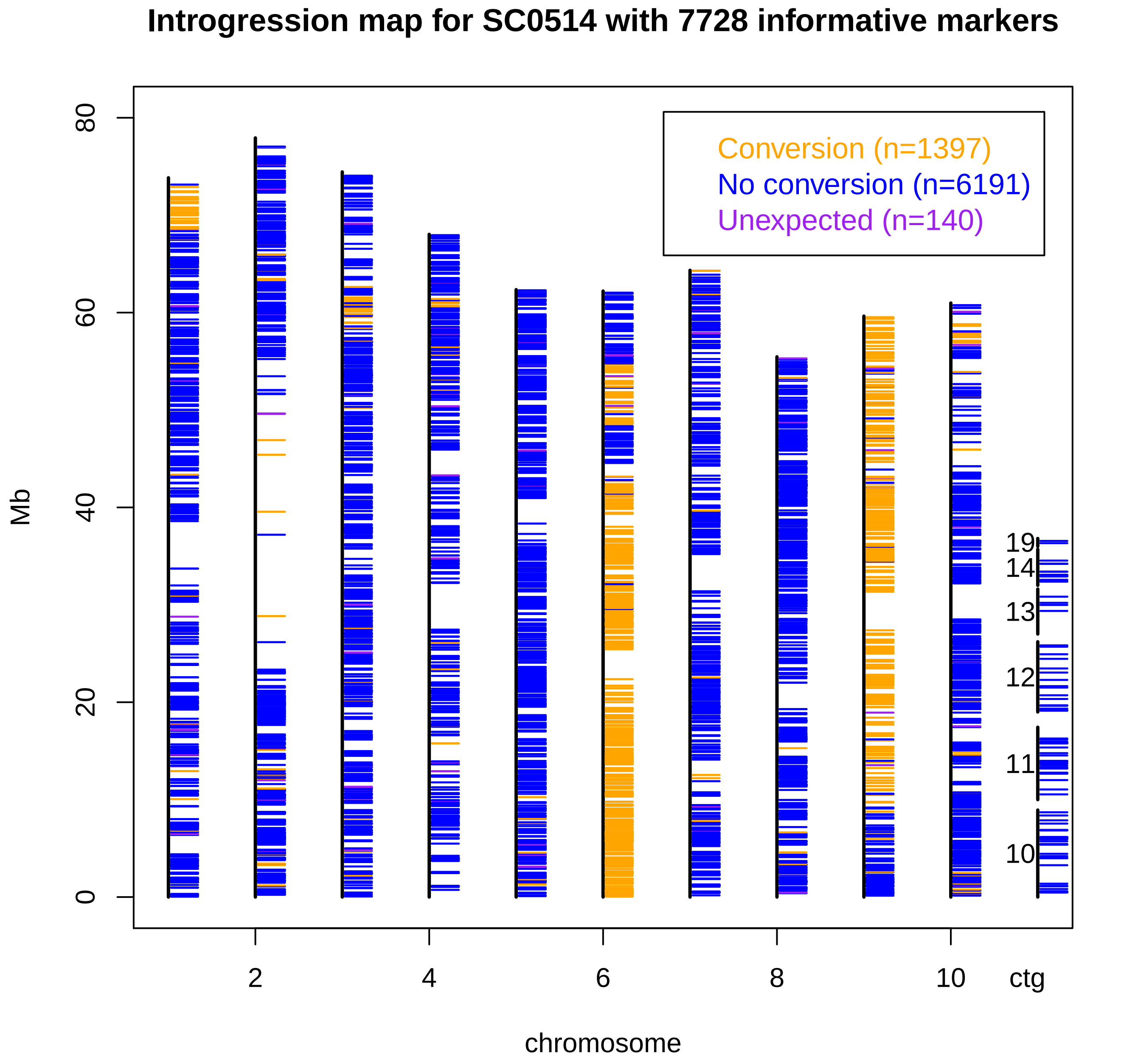

Introgression map for SC0514 with 7728 informative markers
Conversion (n=1397)
No conversion (n=6191)
Unexpected (n=140)
Mb
0
20
40
60
80
chromosome
2
4
6
8
10
ctg
19
14
13
12
11
10

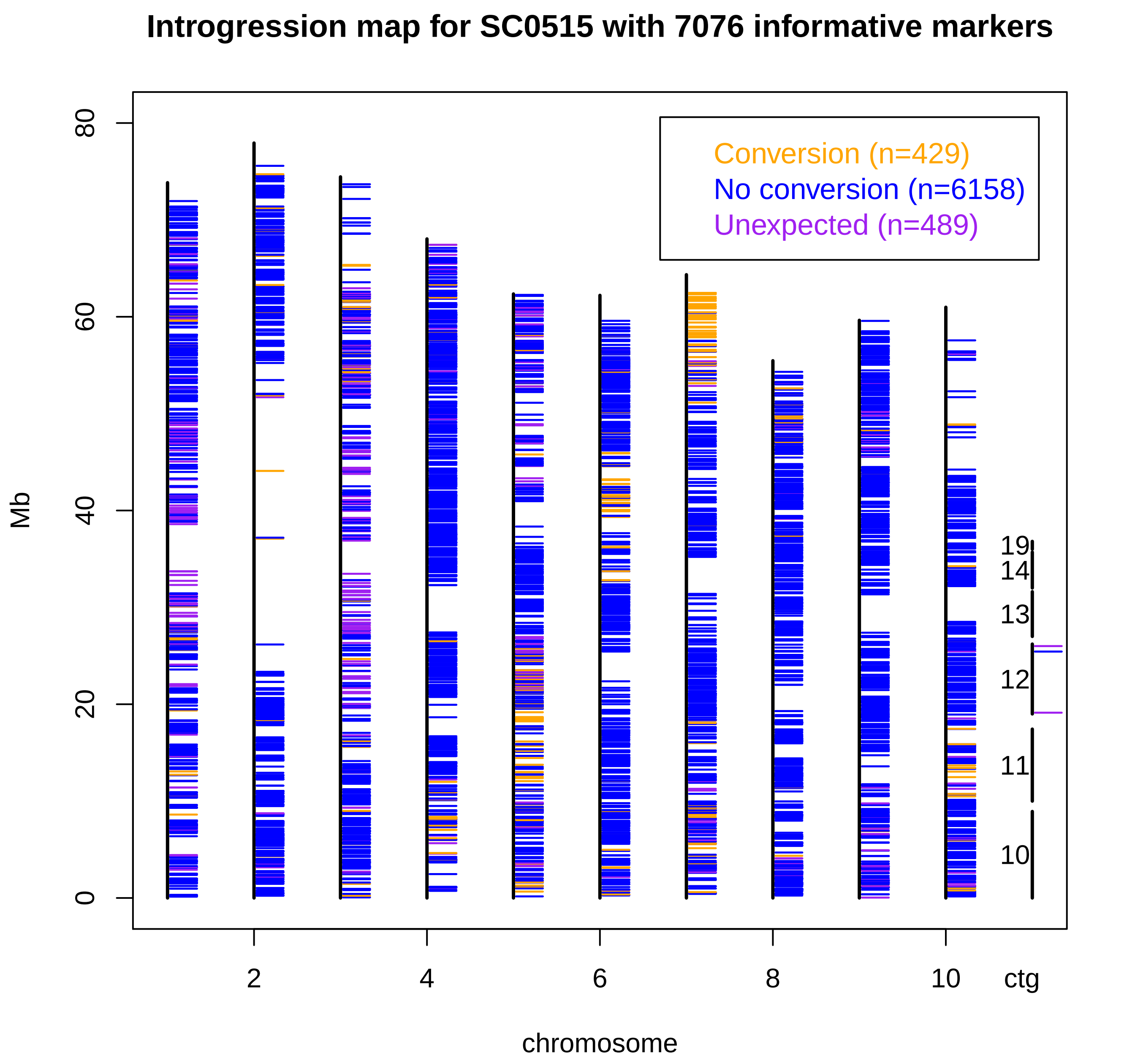

Introgression map for SC0515 with 7076 informative markers
Conversion (n=429)
No conversion (n=6158)
Unexpected (n=489)
Mb
0
20
40
60
80
2
4
6
8
10
ctg
19
14
13
12
11
10
chromosome

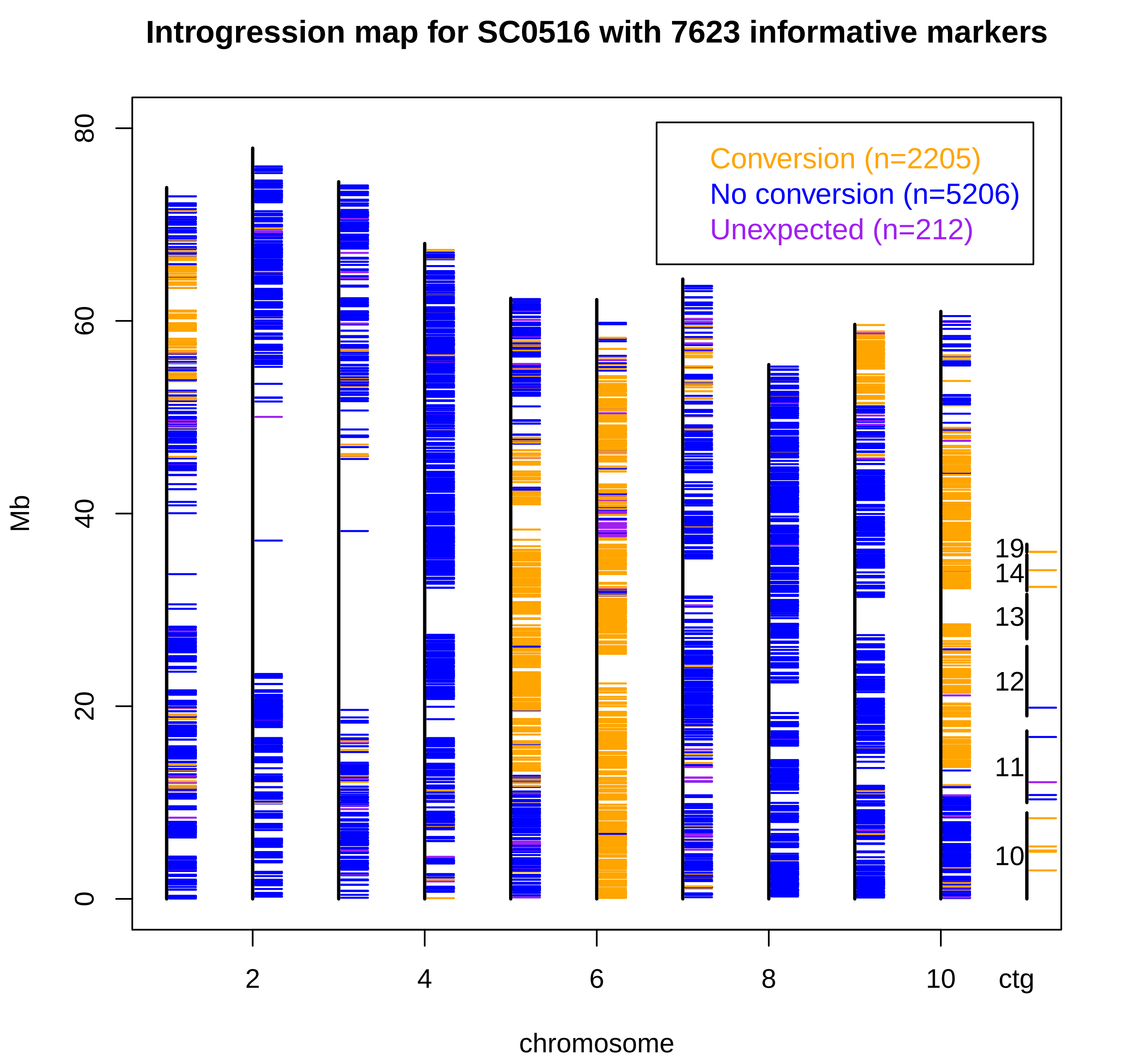

Introgression map for SC0516 with 7623 informative markers
Conversion (n=2205)
No conversion (n=5206)
Unexpected (n=212)
Mb
0
20
40
60
80
chromosome
2
4
6
8
10
ctg
19
14
13
12
11
10

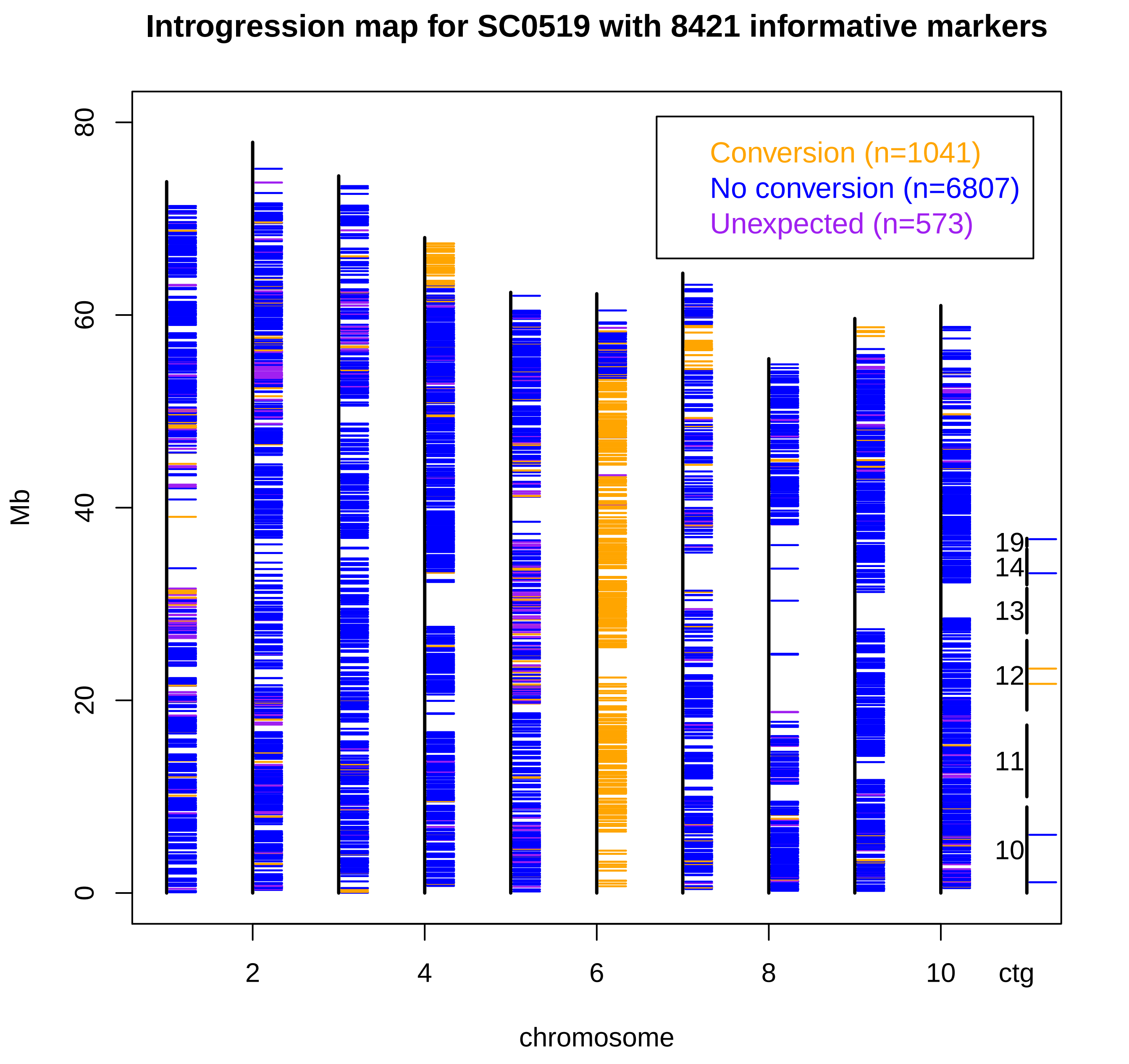
Introgression map for SC0519 with 8421 informative markers
Conversion (n=1041)
No conversion (n=6807)
Unexpected (n=573)
Mb
0
20
40
60
80
chromosome
2
4
6
8
10
ctg
19
14
13
12
11
10

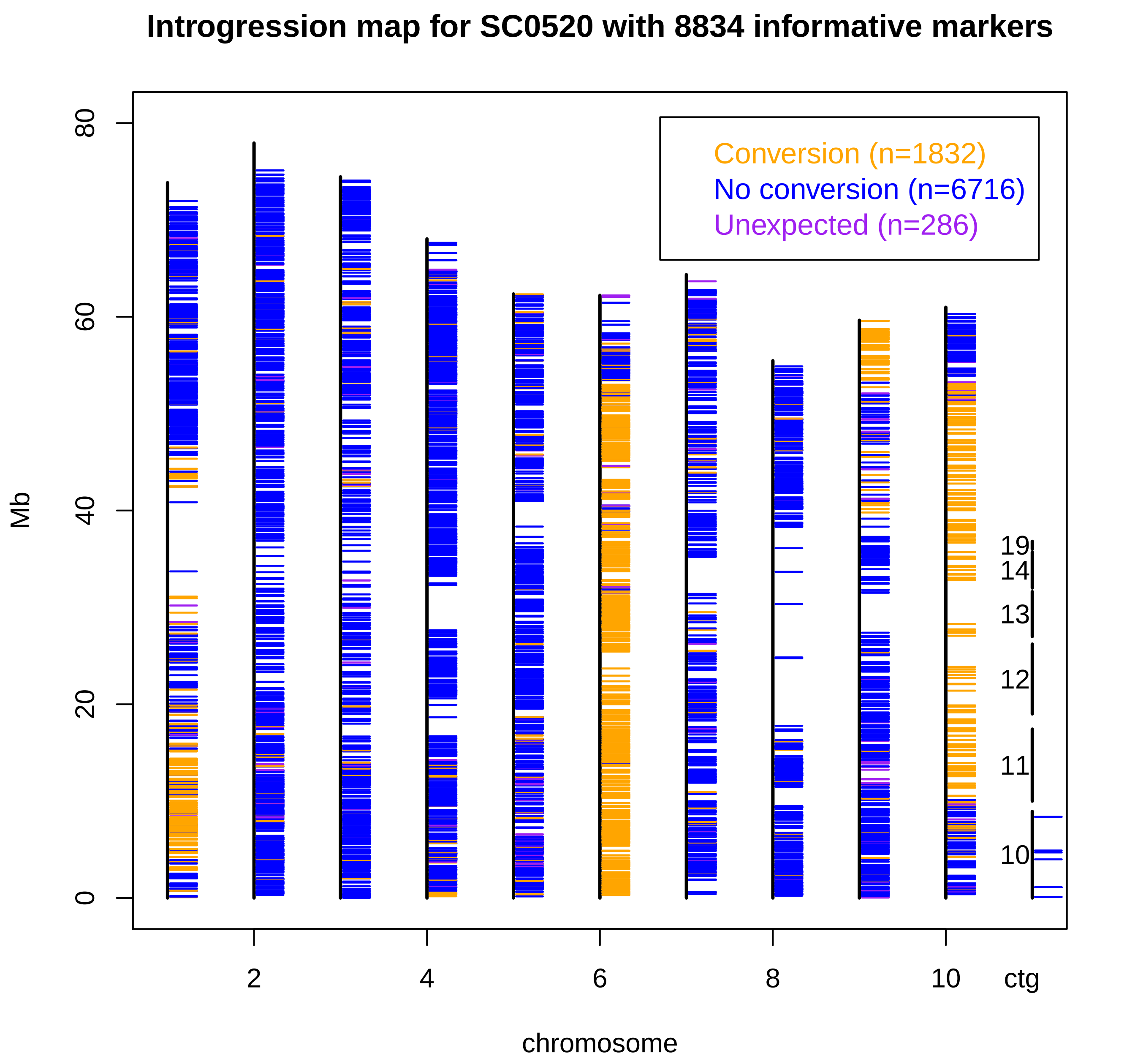
Introgression map for SC0520 with 8834 informative markers
Conversion (n=1832)
No conversion (n=6716)
Unexpected (n=286)
Mb
0
20
40
60
80
chromosome
2
4
6
8
10
ctg
19
14
13
12
11
10

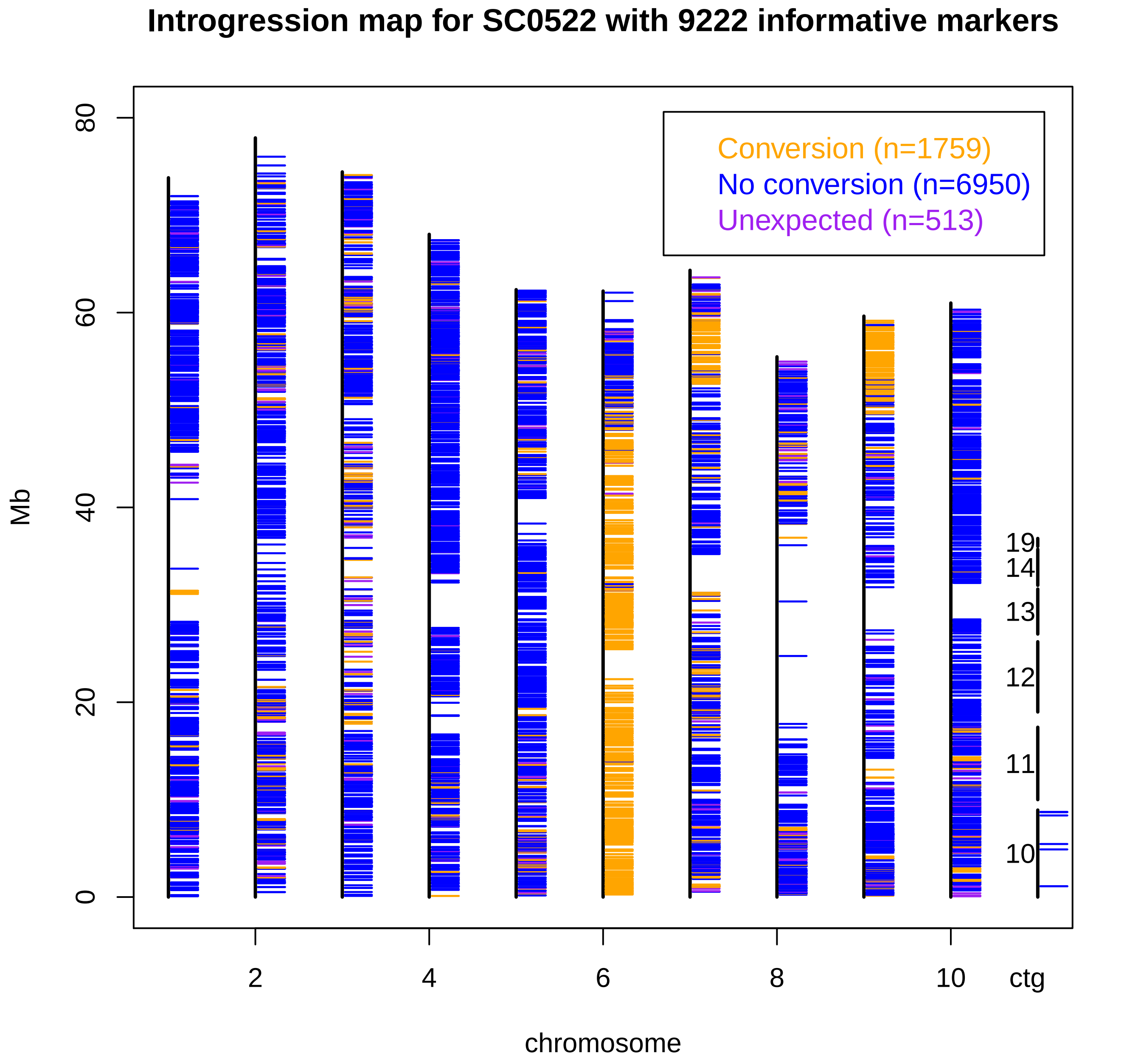

Introgression map for SC0522 with 9222 informative markers
Conversion (n=1759)
No conversion (n=6950)
Unexpected (n=513)
Mb
0
20
40
60
80
2
4
6
8
10
ctg
19
14
13
12
11
10
chromosome

Introgression map for SC0523 with 9167 informative markers

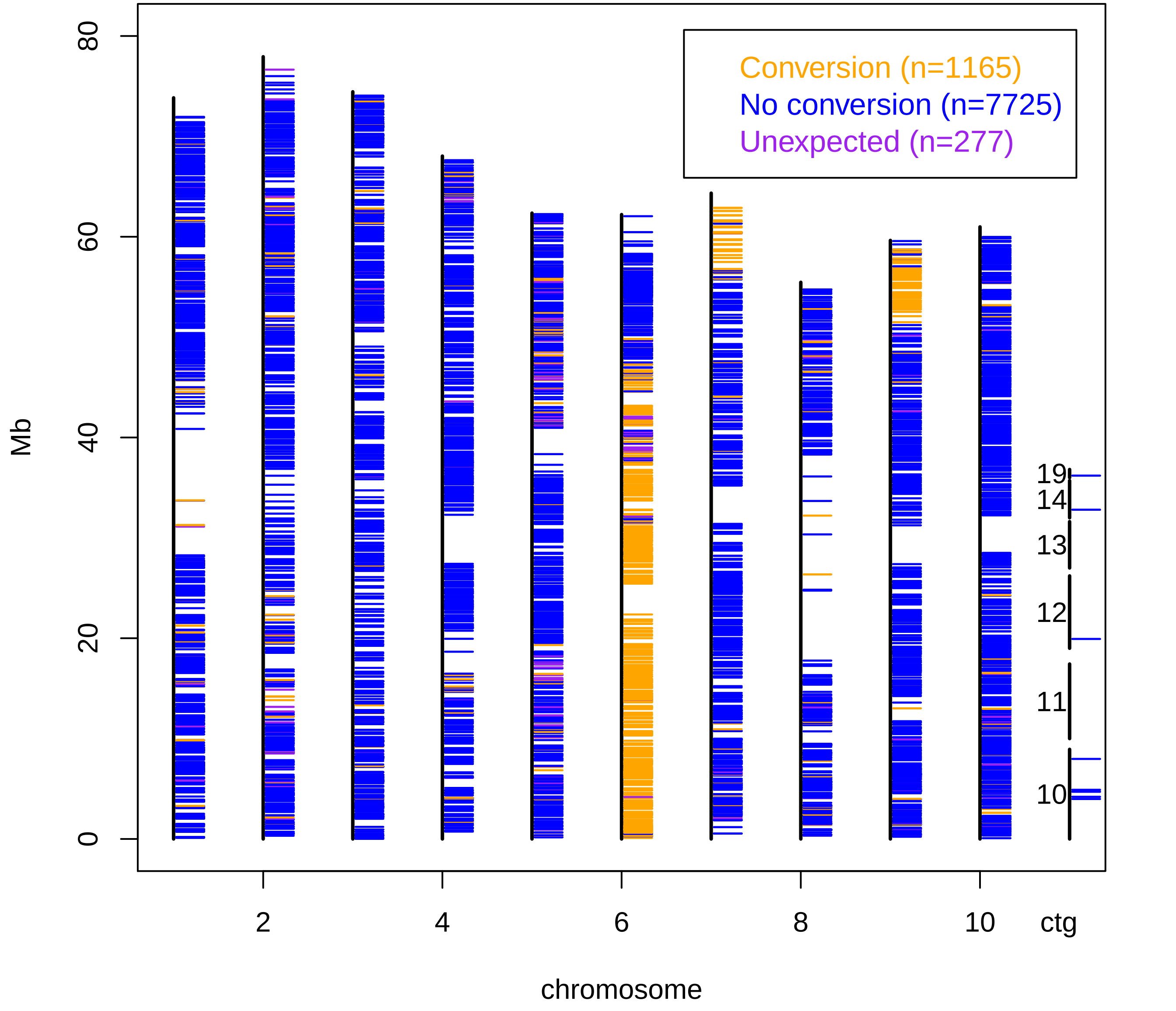

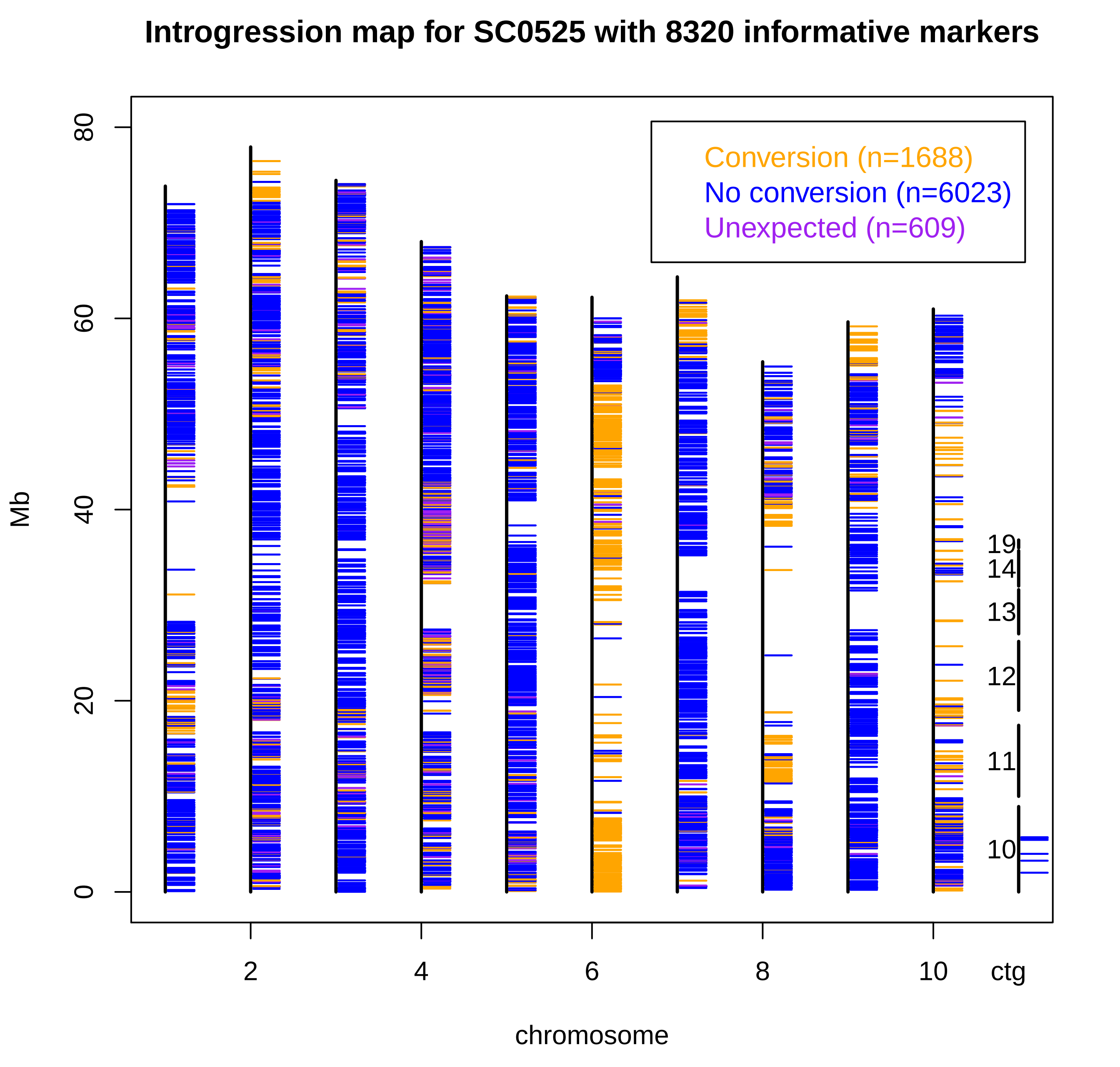

Introgression map for SC0525 with 8320 informative markers
Conversion (n=1688)
No conversion (n=6023)
Unexpected (n=609)
Mb
0
20
40
60
80
2
4
6
8
10
ctg
19
14
13
12
11
10
chromosome

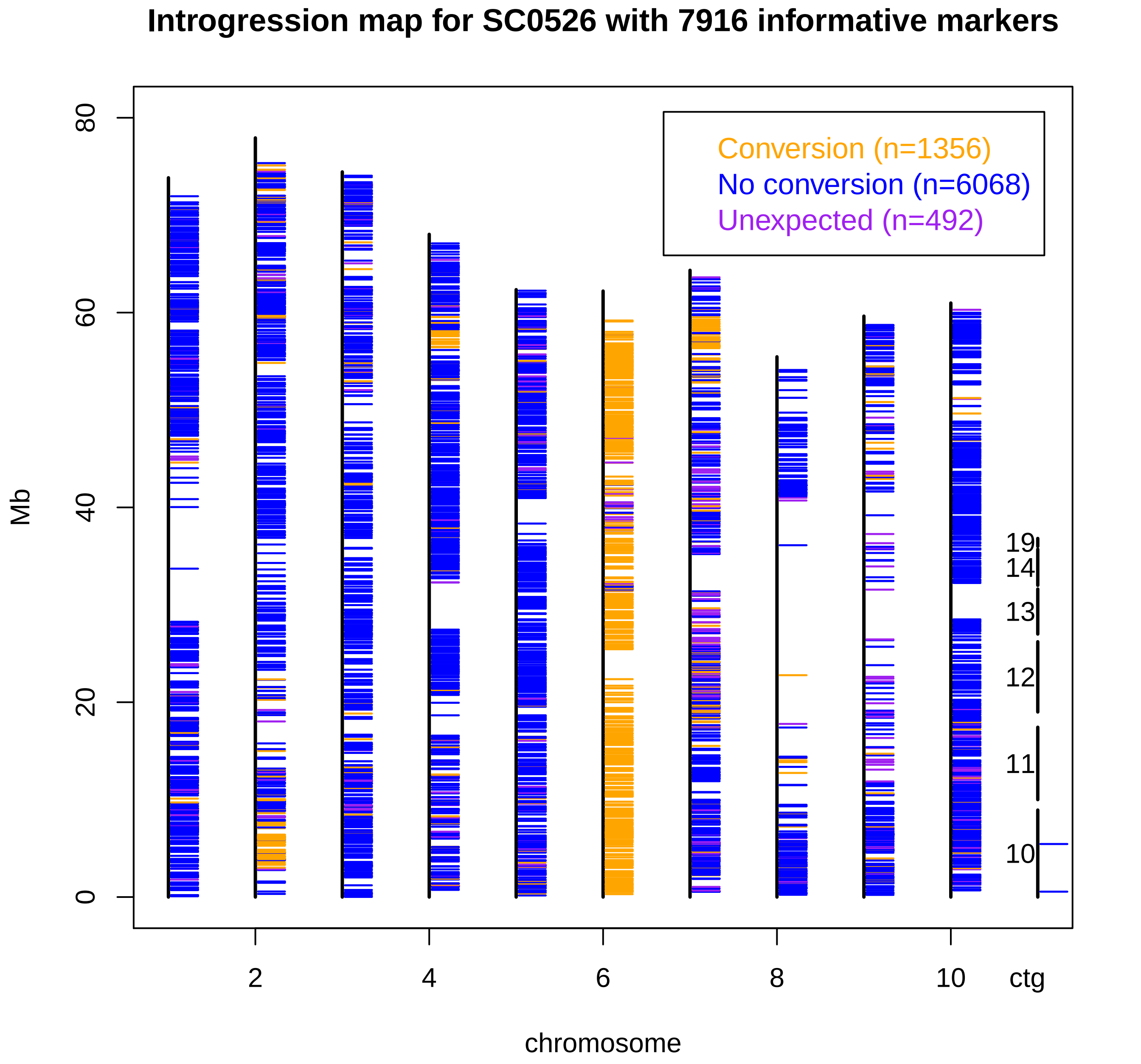
Introgression map for SC0526 with 7916 informative markers
Conversion (n=1356)
No conversion (n=6068)
Unexpected (n=492)
Mb
0
20
40
60
80
chromosome
2
4
6
8
10
ctg
19
14
13
12
11
10

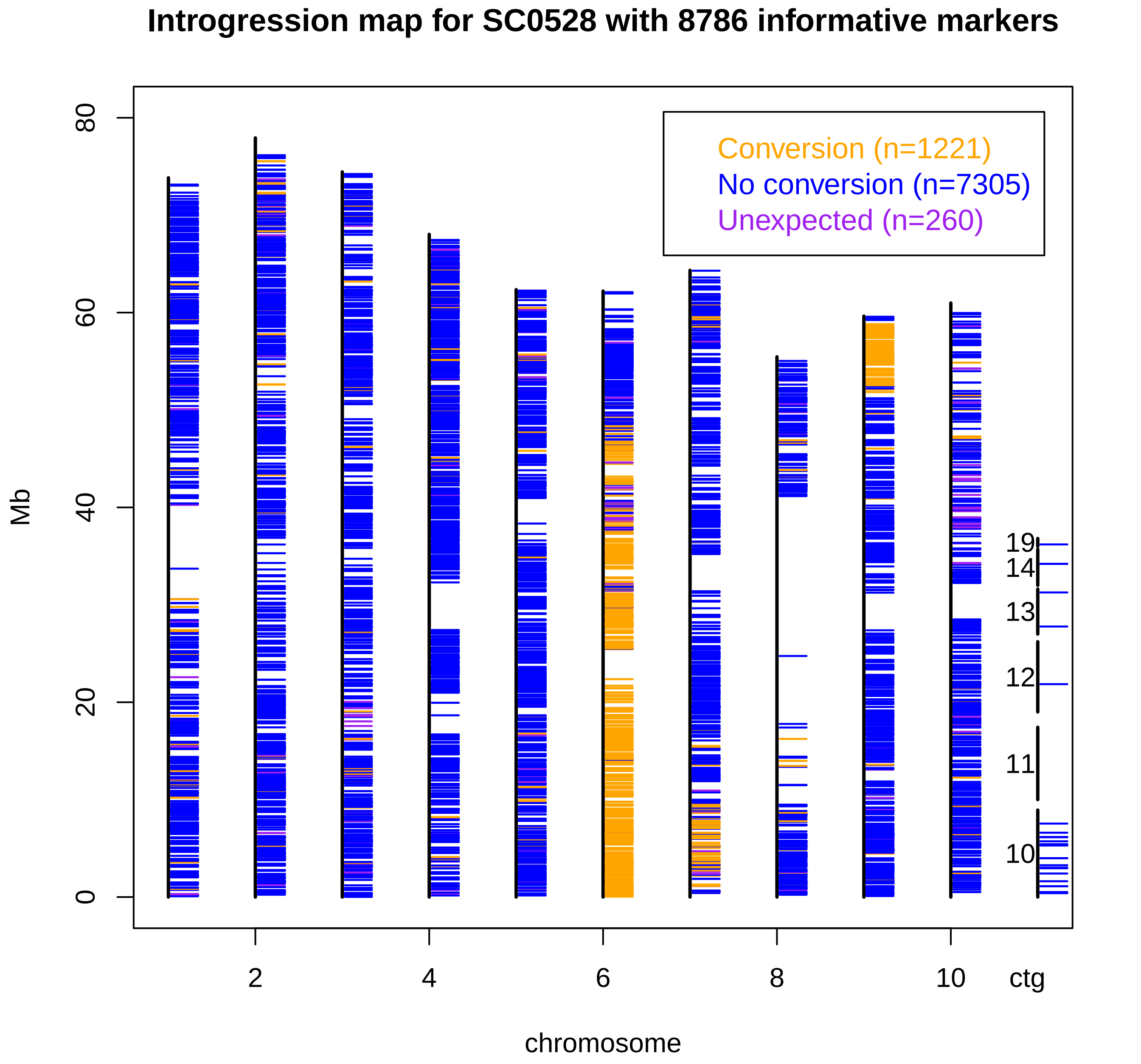

Introgression map for SC0528 with 8786 informative markers
Conversion (n=1221)
No conversion (n=7305)
Unexpected (n=260)
Mb
0
20
40
60
80
2
4
6
8
10
ctg
19
14
13
12
11
10
chromosome

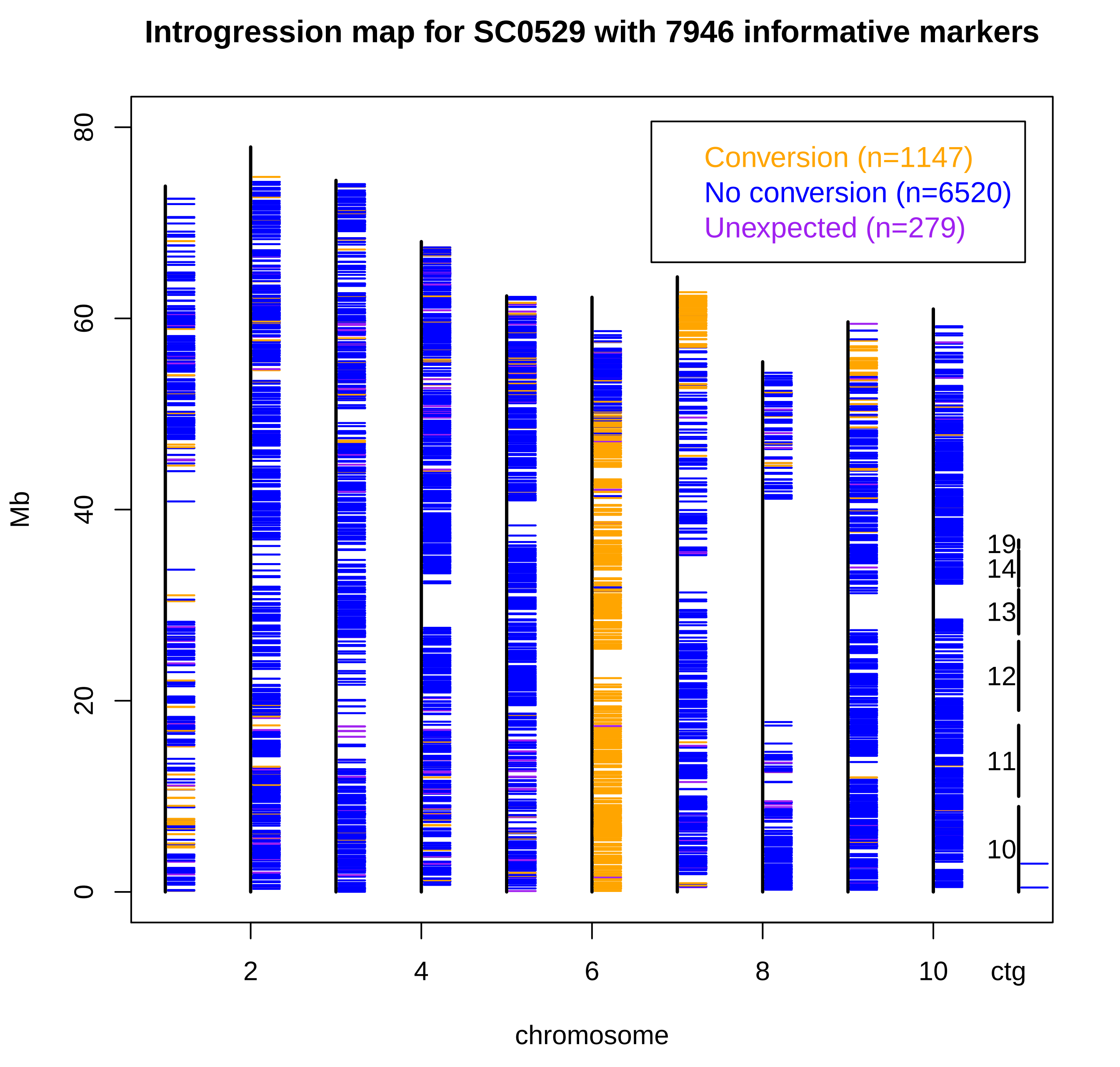

Introgression map for SC0529 with 7946 informative markers
Conversion (n=1147)
No conversion (n=6520)
Unexpected (n=279)
Mb
0
20
40
60
80
chromosome
2
4
6
8
10
ctg
19
14
13
12
11
10

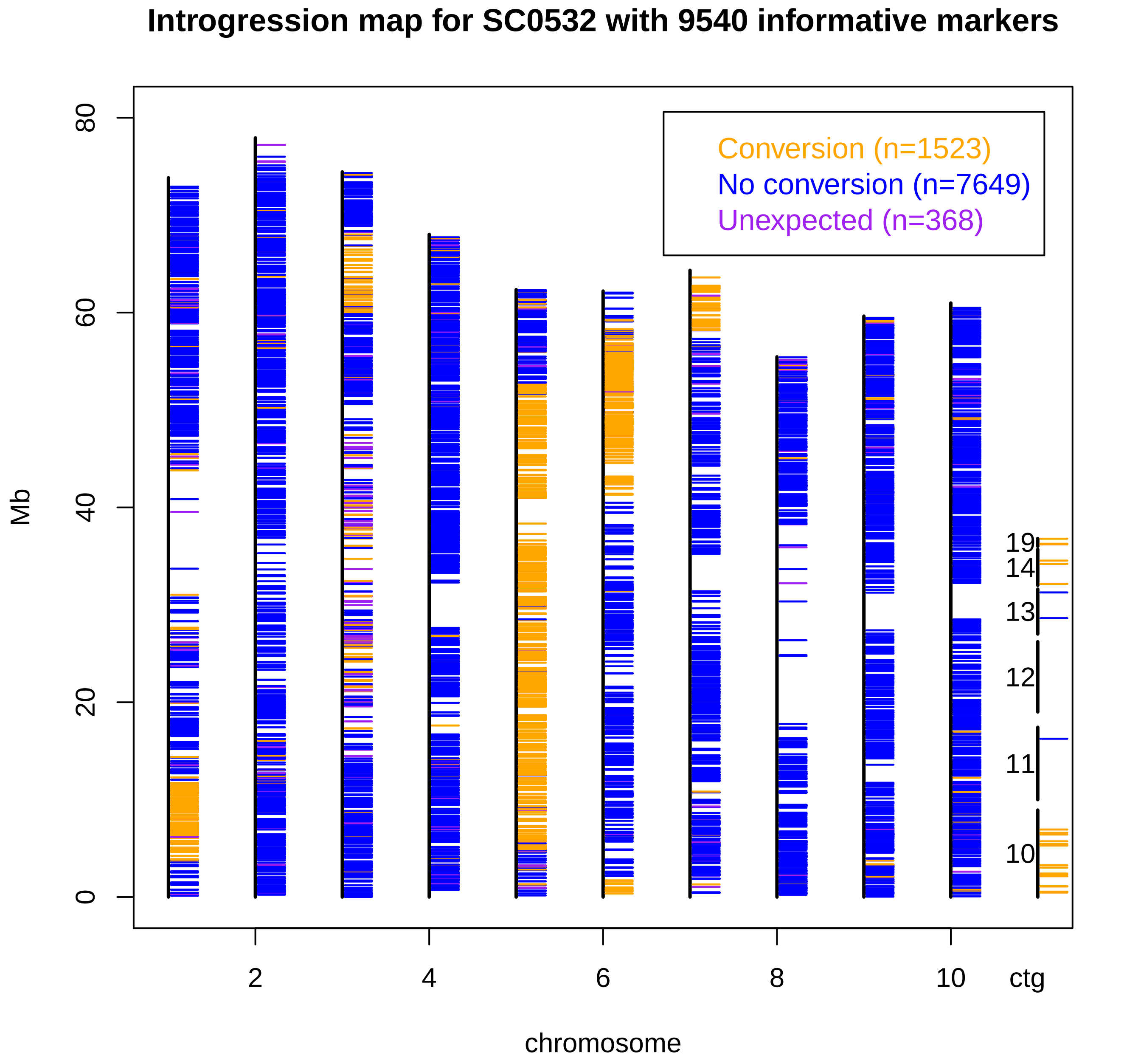
Introgression map for SC0532 with 9540 informative markers
Conversion (n=1523)
No conversion (n=7649)
Unexpected (n=368)
Mb
0
20
40
60
80
chromosome
2
4
6
8
10
ctg
19
14
13
12
11
10

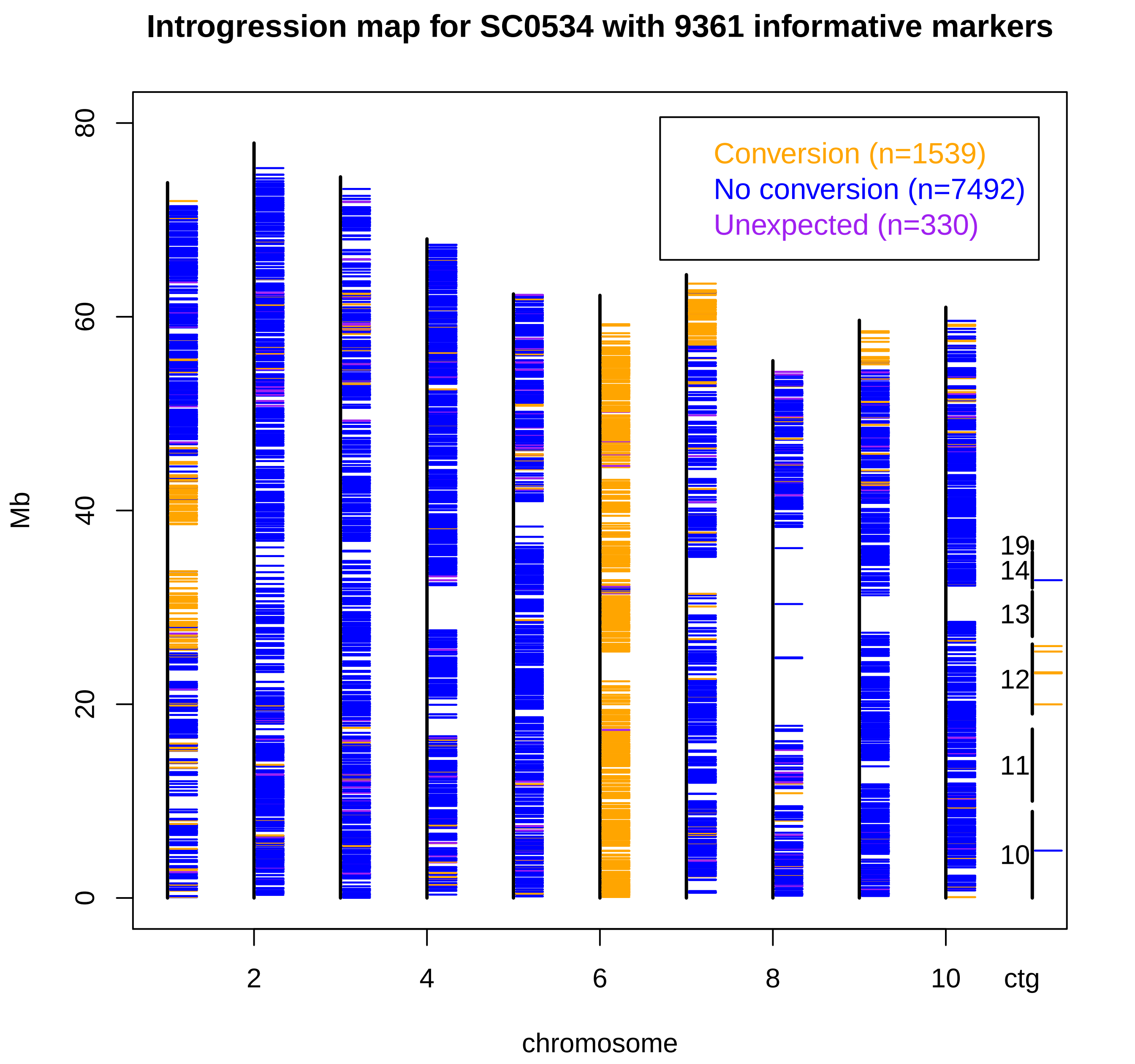

Introgression map for SC0534 with 9361 informative markers
Conversion (n=1539)
No conversion (n=7492)
Unexpected (n=330)
Mb
0
20
40
60
80
2
4
6
8
10
ctg
19
14
13
12
11
10
chromosome

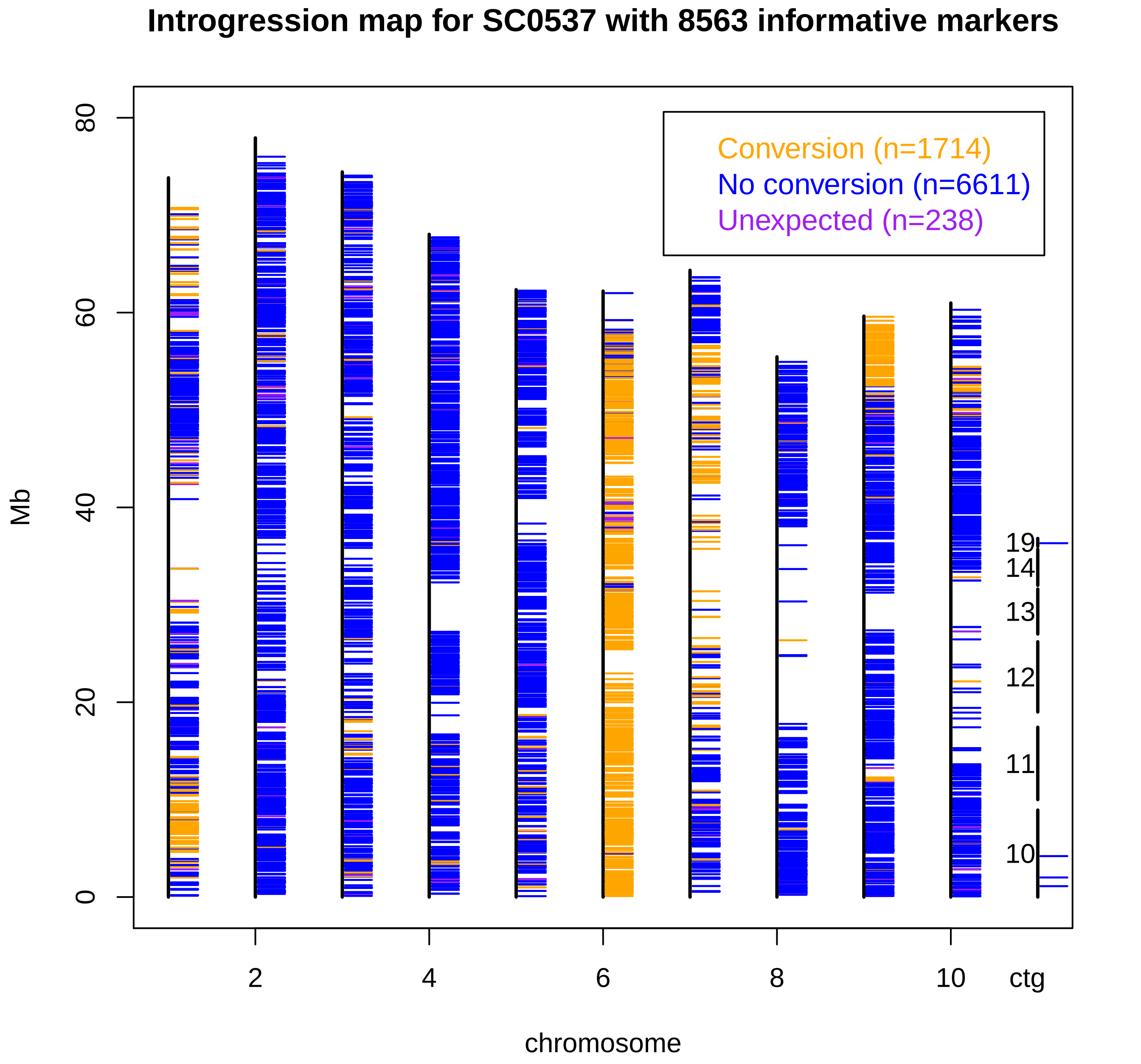
Introgression map for SC0537 with 8563 informative markers
Conversion (n=1714)
No conversion (n=6611)
Unexpected (n=238)
Mb
0
20
40
60
80
chromosome
2
4
6
8
10
ctg
19
14
13
12
11
10

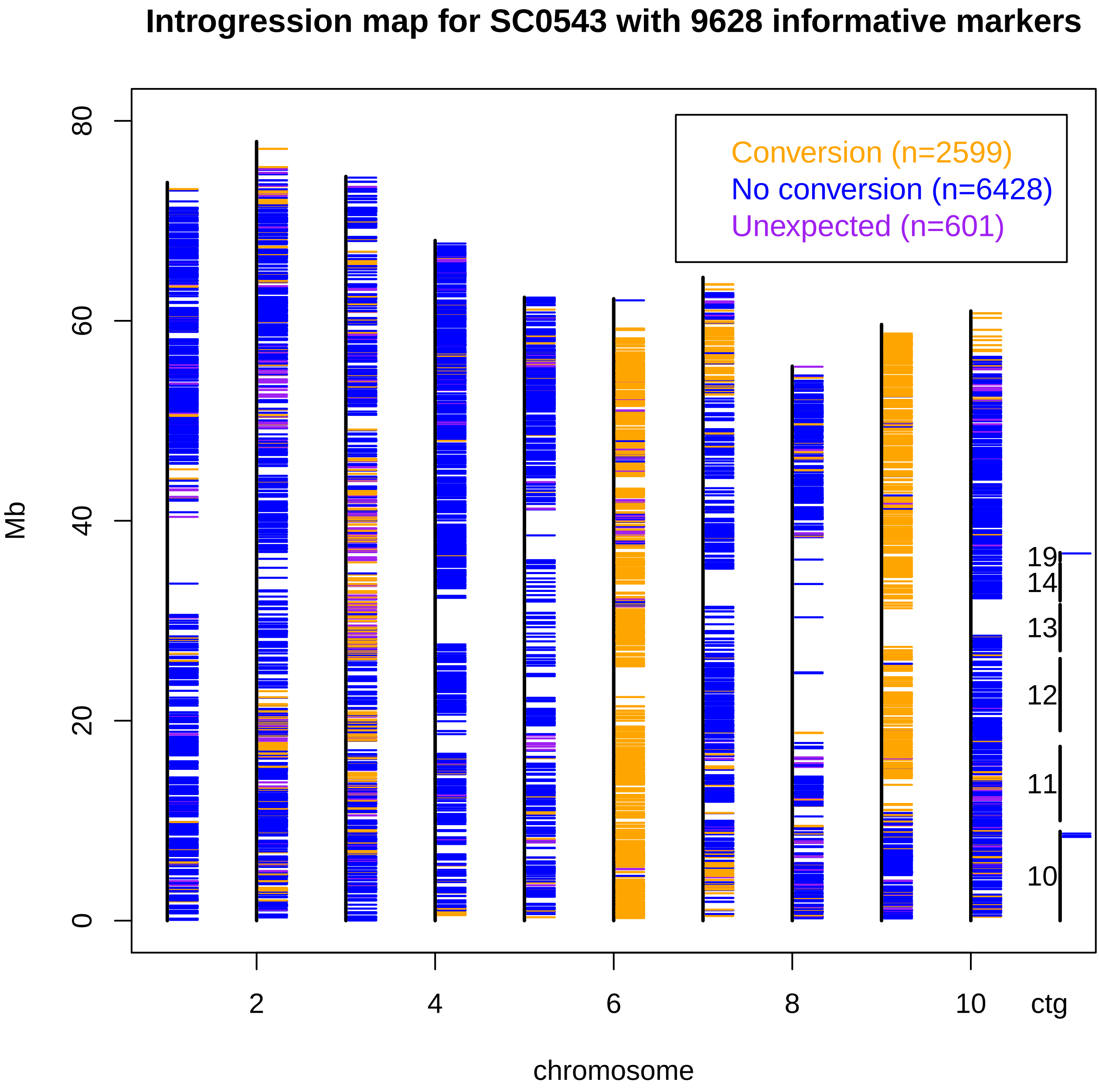

Introgression map for SC0543 with 9628 informative markers
Conversion (n=2599)
No conversion (n=6428)
Unexpected (n=601)
Mb
0
20
40
60
80
chromosome
2
4
6
8
10
ctg
19
14
13
12
11
10

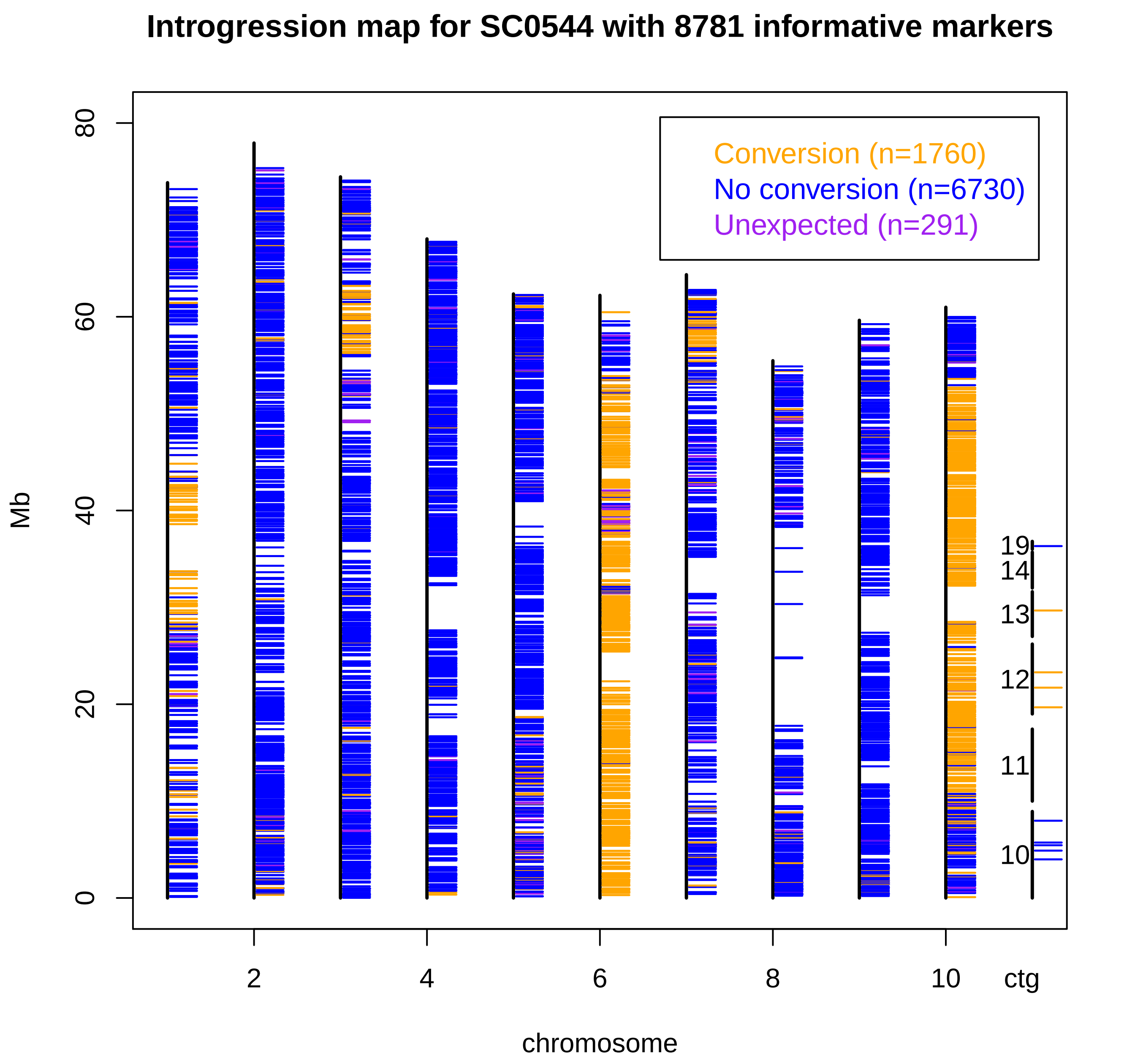

Introgression map for SC0544 with 8781 informative markers
Conversion (n=1760)
No conversion (n=6730)
Unexpected (n=291)
Mb
0
20
40
60
80
2
4
6
8
10
ctg
19
14
13
12
11
10
chromosome

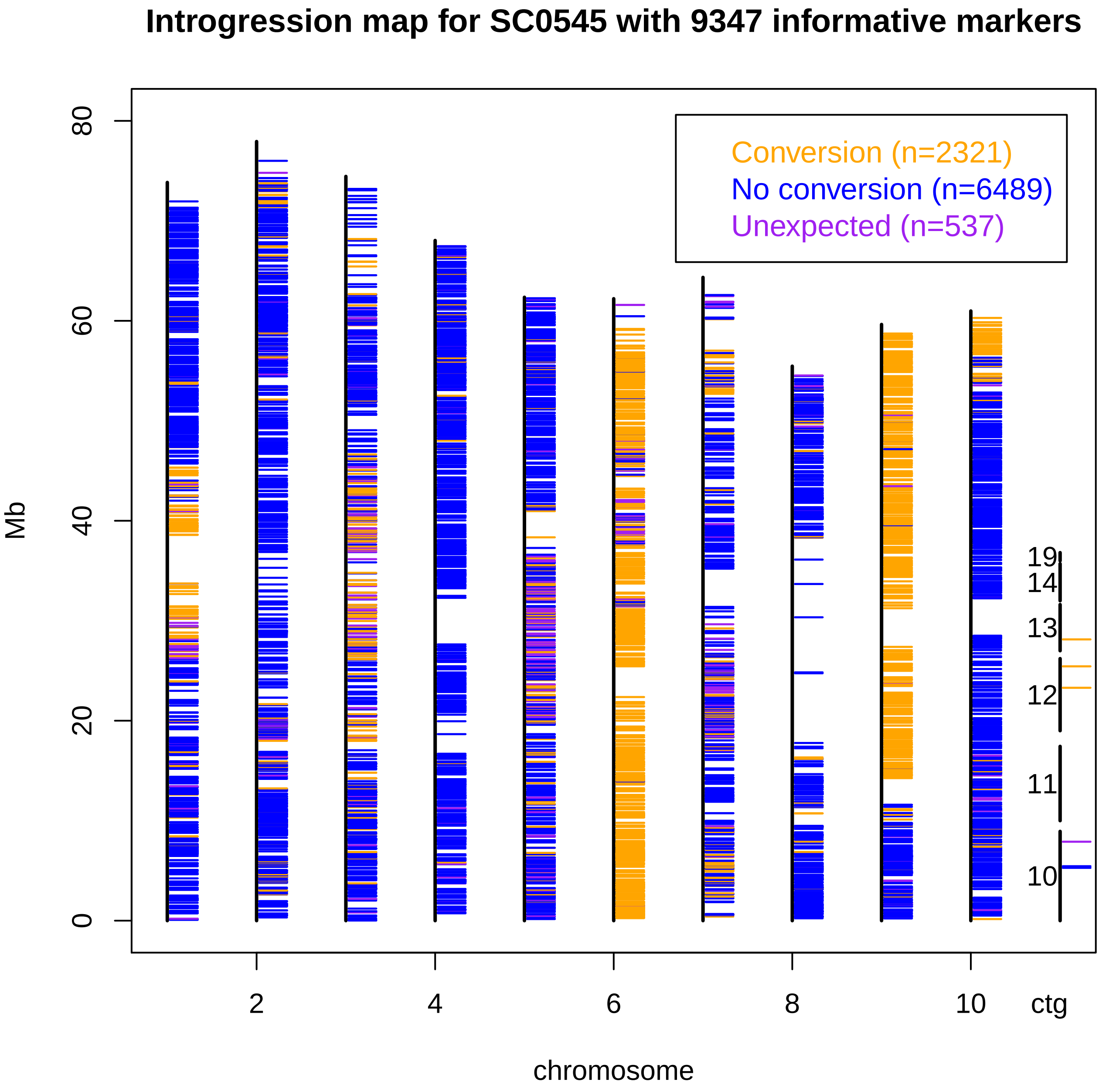

Introgression map for SC0545 with 9347 informative markers
Conversion (n=2321)
No conversion (n=6489)
Unexpected (n=537)
Mb
0
20
40
60
80
2
4
6
8
10
ctg
chromosome
19
14
13
12
11
10

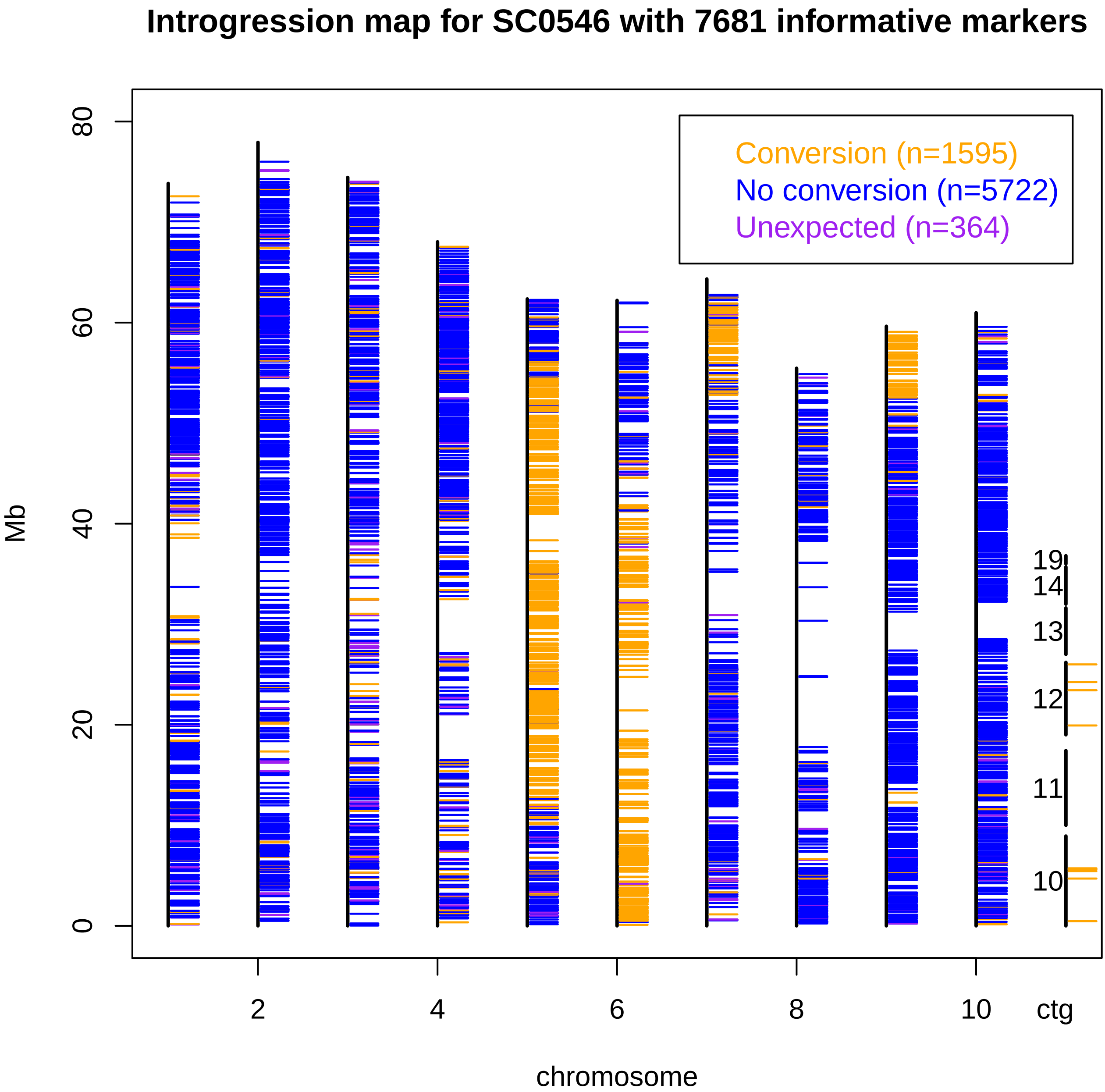
Introgression map for SC0546 with 7681 informative markers
Conversion (n=1595)
No conversion (n=5722)
Unexpected (n=364)
Mb
0
20
40
60
80
2
4
6
8
10
ctg
19
14
13
12
11
10
chromosome

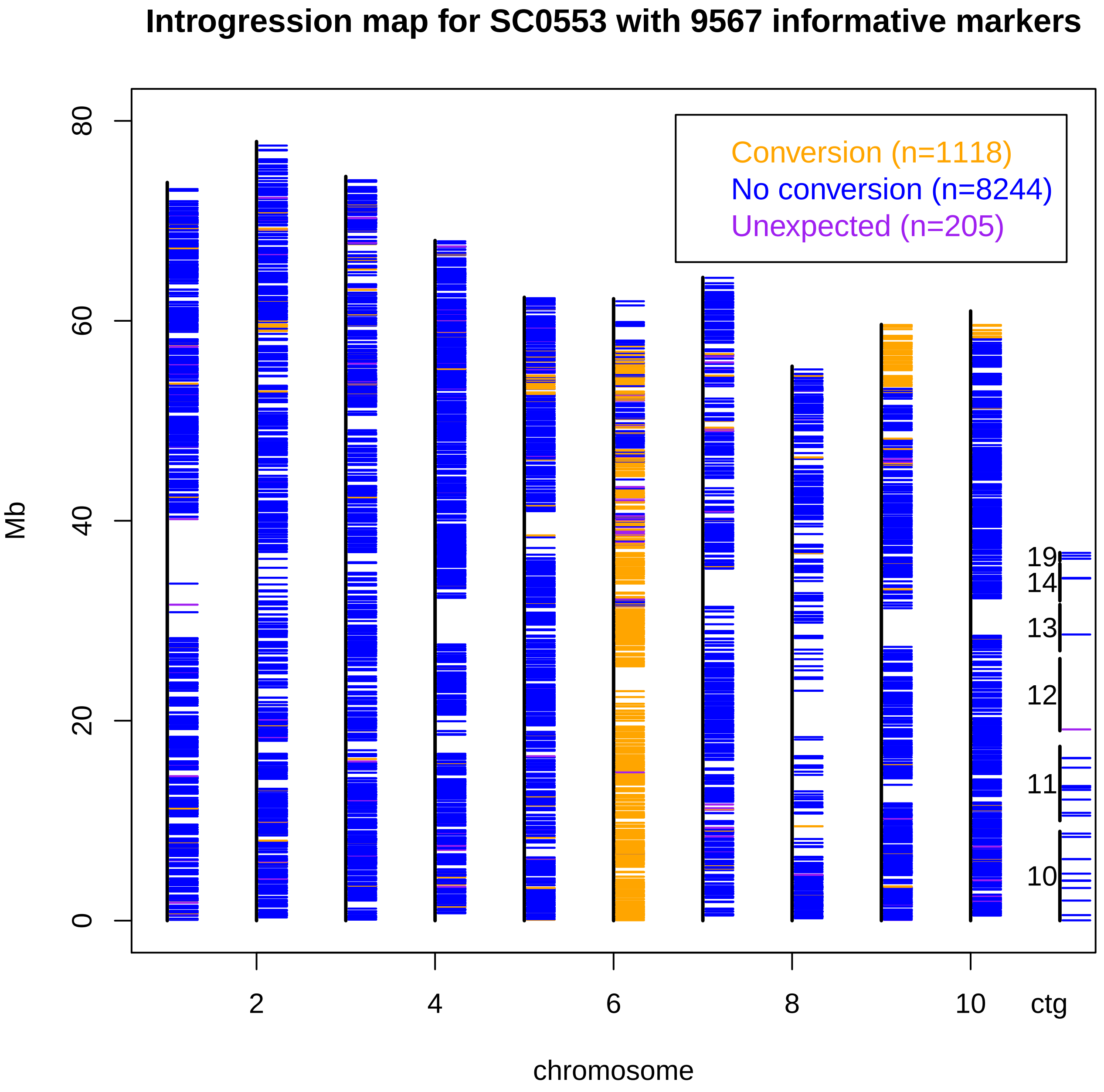
Introgression map for SC0553 with 9567 informative markers
Conversion (n=1118)
No conversion (n=8244)
Unexpected (n=205)
Mb
0
20
40
60
80
2
4
6
8
10
ctg
19
14
13
12
11
10
chromosome

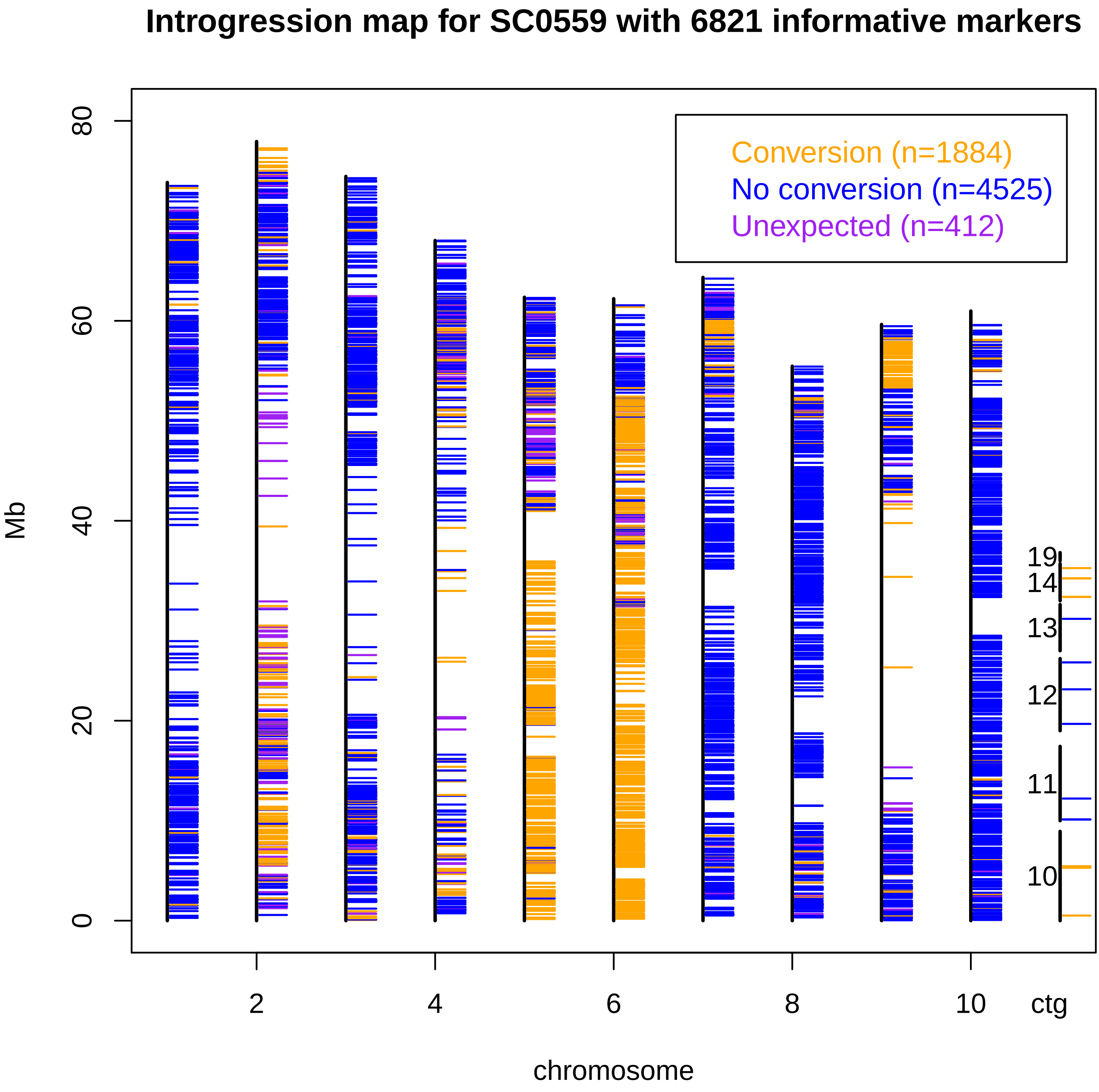
Introgression map for SC0559 with 6821 informative markers
Conversion (n=1884)
No conversion (n=4525)
Unexpected (n=412)
Mb
0
20
40
60
80
chromosome
2
4
6
8
10
ctg
19
14
13
12
11
10

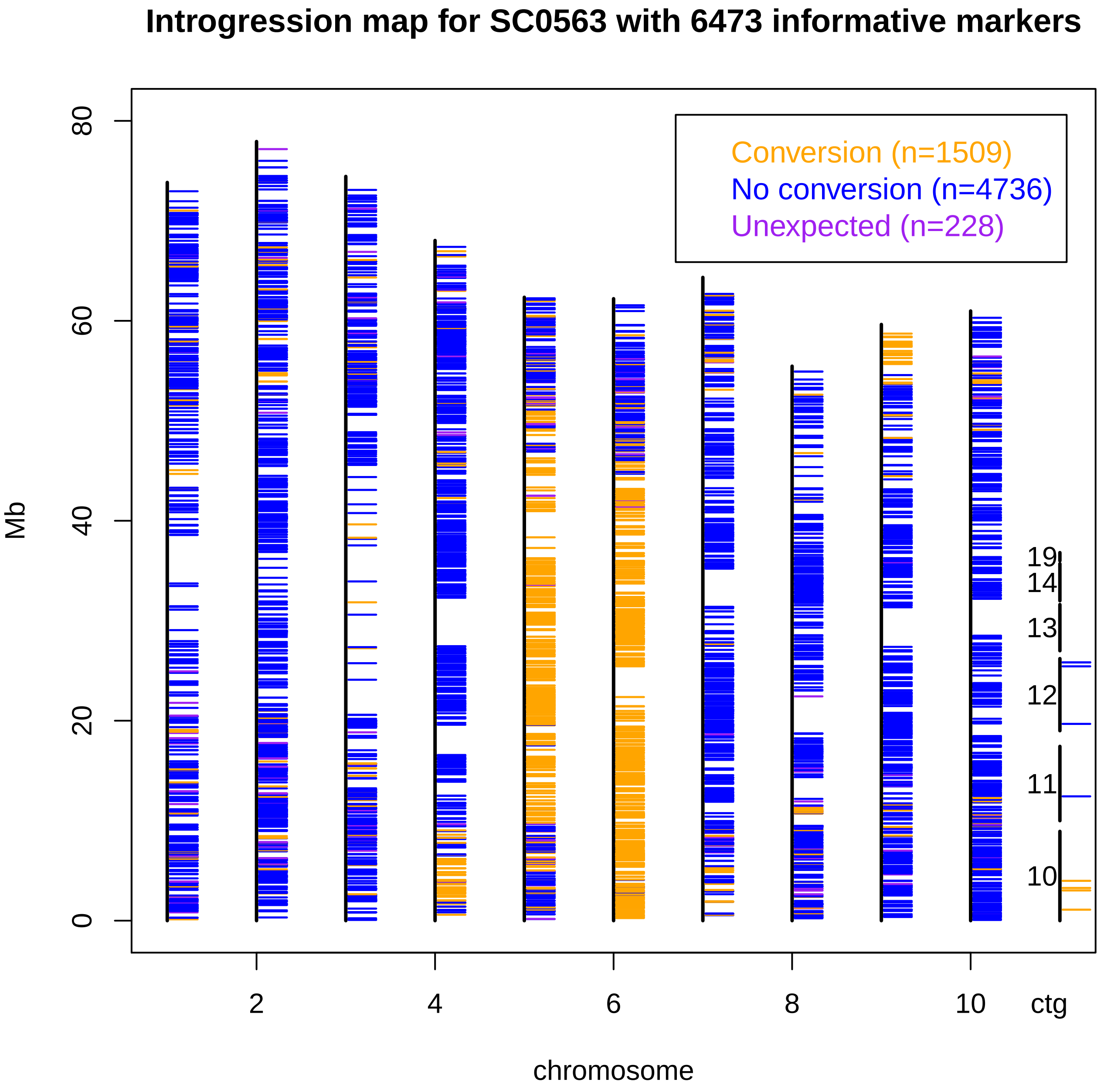
Introgression map for SC0563 with 6473 informative markers
Conversion (n=1509)
No conversion (n=4736)
Unexpected (n=228)
Mb
0
20
40
60
80
chromosome
2
4
6
8
10
ctg
19
14
13
12
11
10

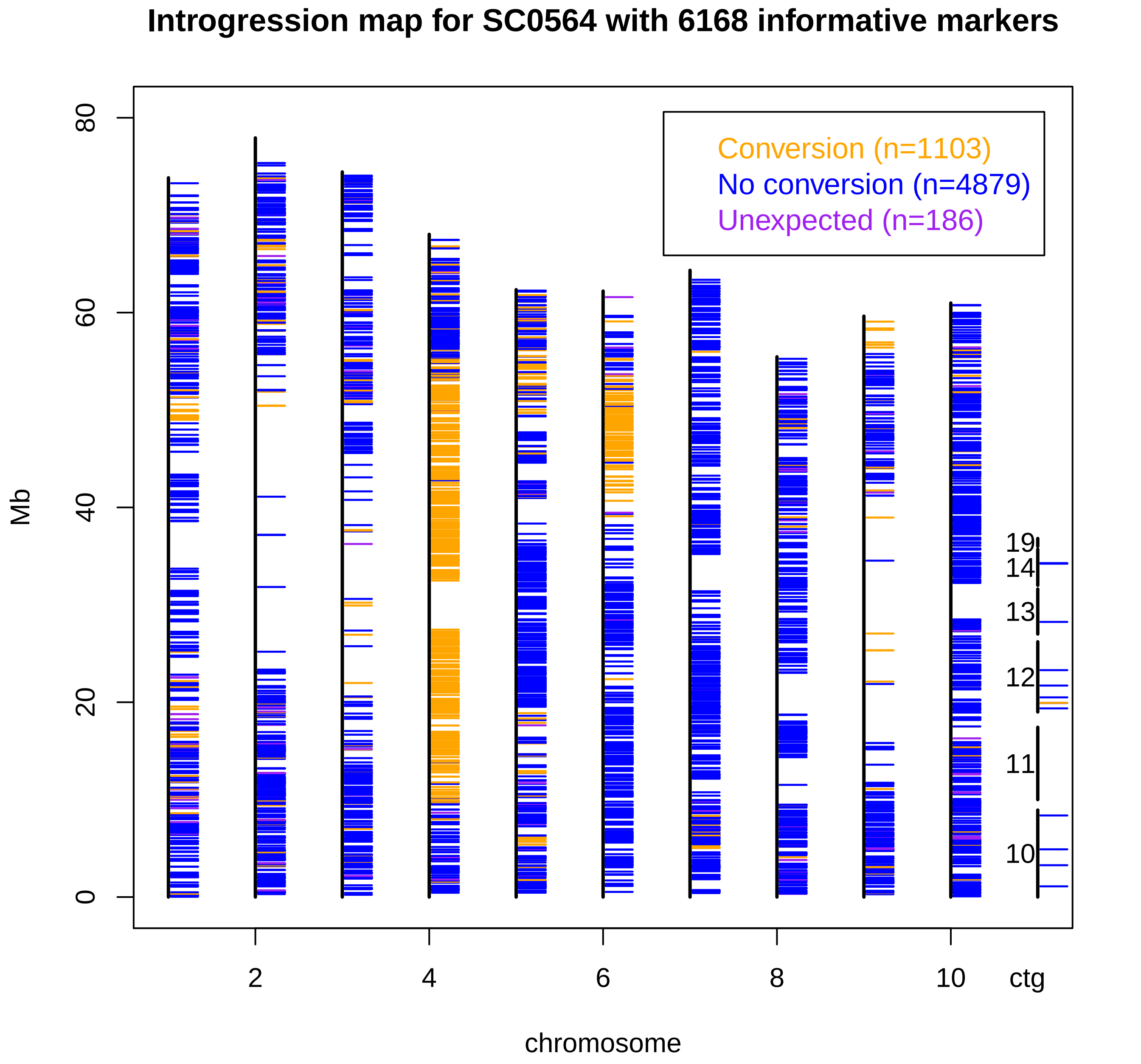

Introgression map for SC0564 with 6168 informative markers
Conversion (n=1103)
No conversion (n=4879)
Unexpected (n=186)
Mb
0
20
40
60
80
chromosome
2
4
6
8
10
ctg
19
14
13
12
11
10

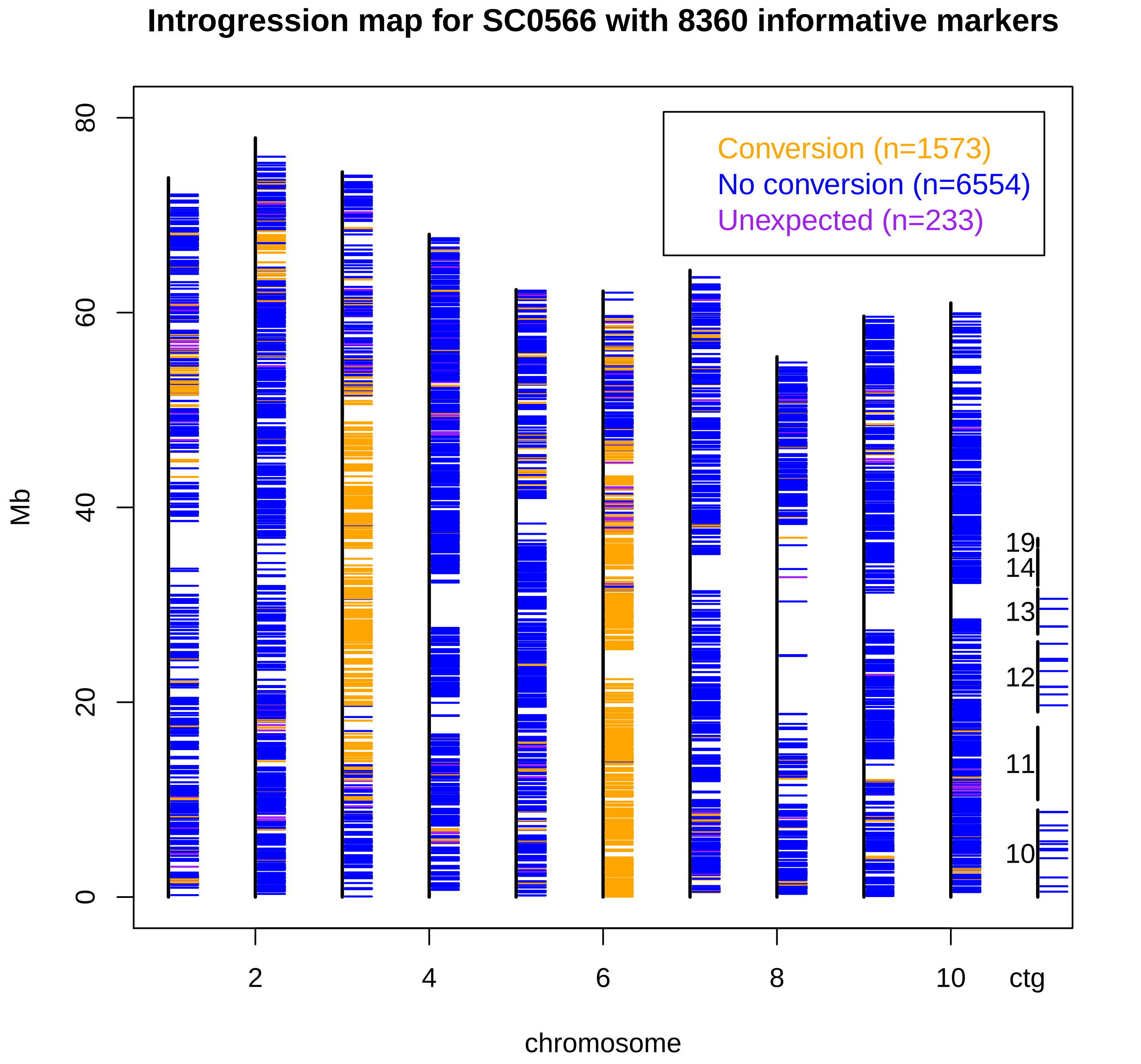

Introgression map for SC0566 with 8360 informative markers
Conversion (n=1573)
No conversion (n=6554)
Unexpected (n=233)
Mb
0
20
40
60
80
chromosome
2
4
6
8
10
ctg
19
14
13
12
11
10

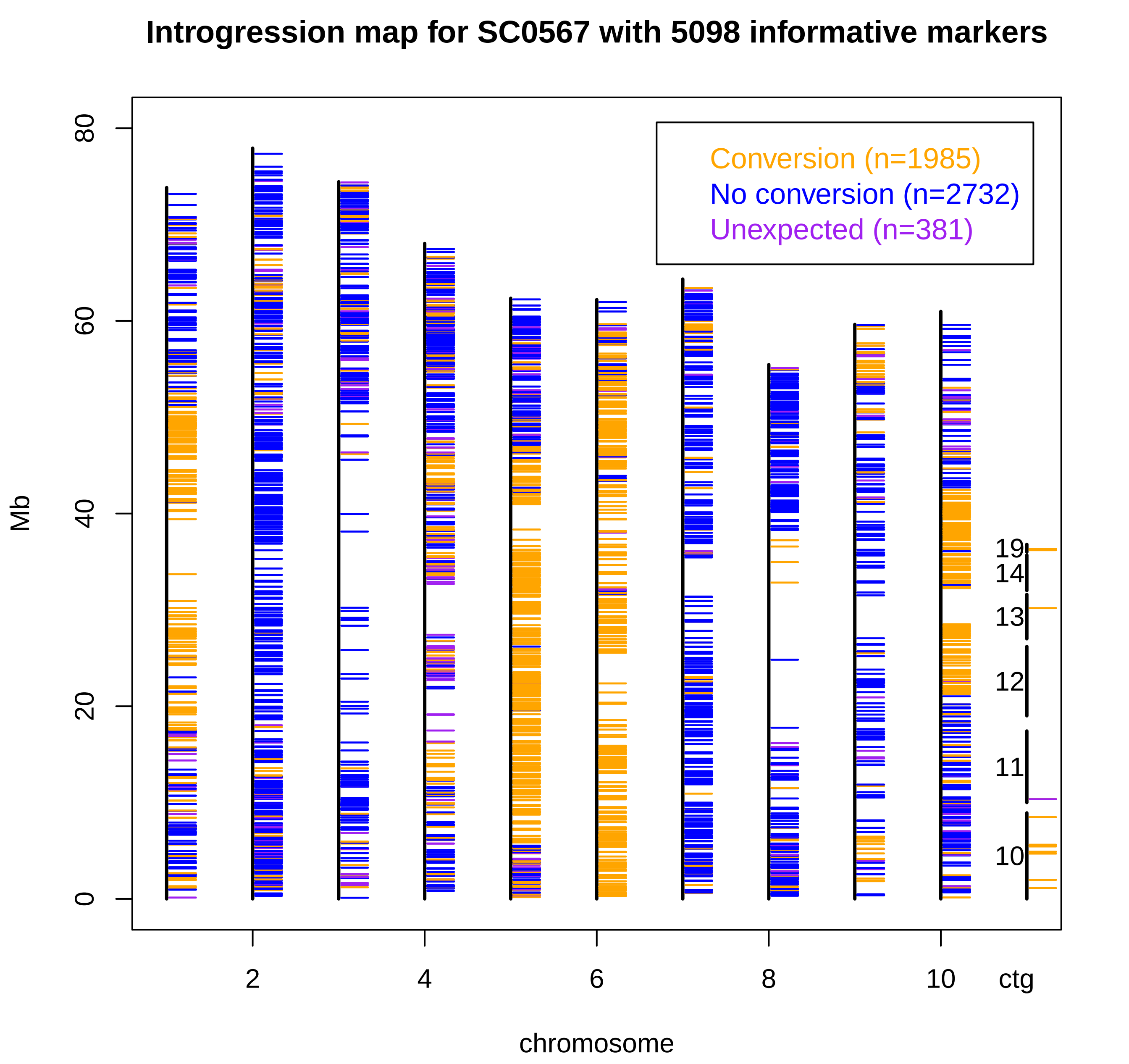

Introgression map for SC0567 with 5098 informative markers
Conversion (n=1985)
No conversion (n=2732)
Unexpected (n=381)
Mb
0
20
40
60
80
chromosome
2
4
6
8
10
ctg
19
14
13
12
11
10

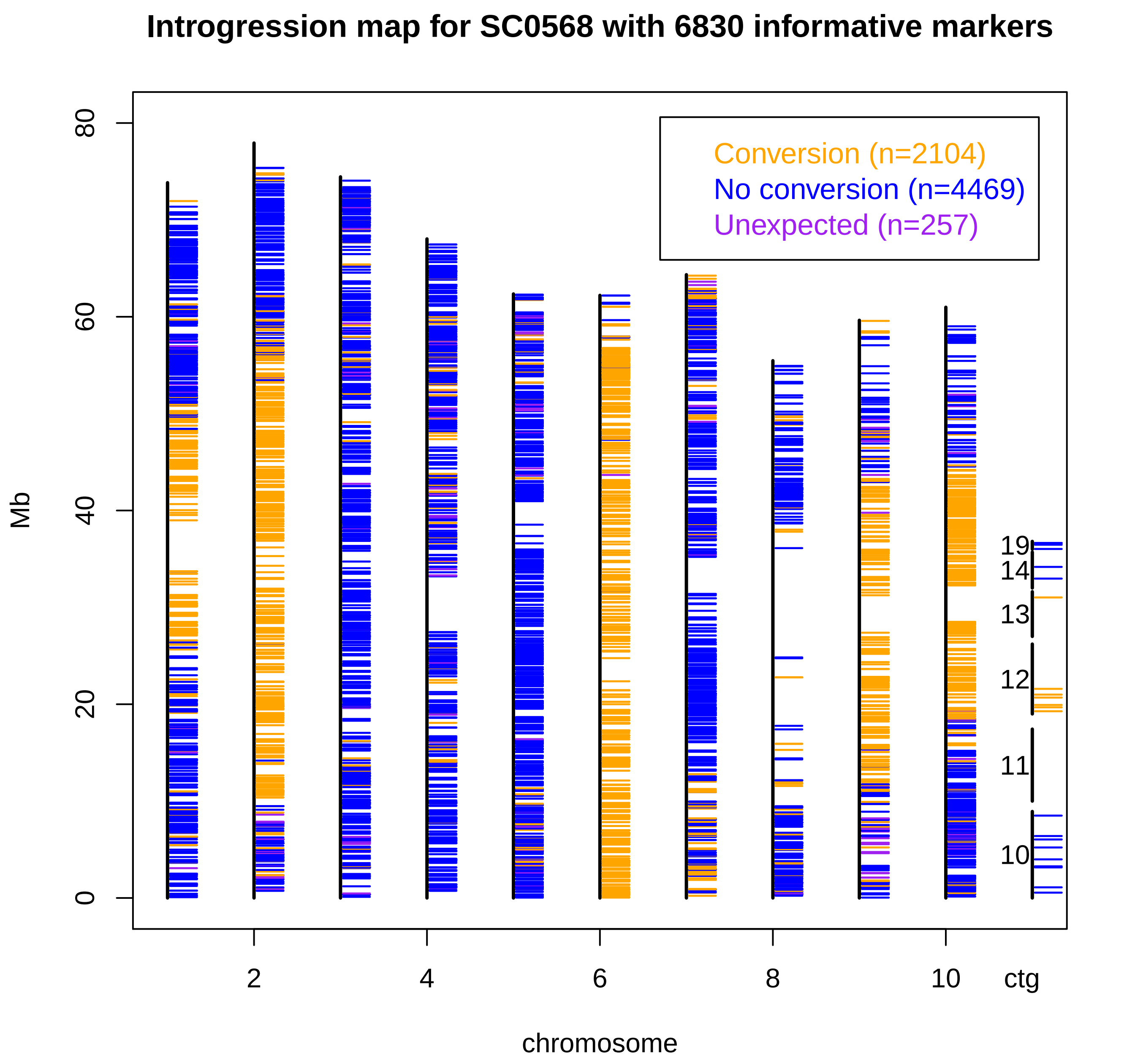

Introgression map for SC0568 with 6830 informative markers
Conversion (n=2104)
No conversion (n=4469)
Unexpected (n=257)
Mb
0
20
40
60
80
chromosome
2
4
6
8
10
ctg
19
14
13
12
11
10

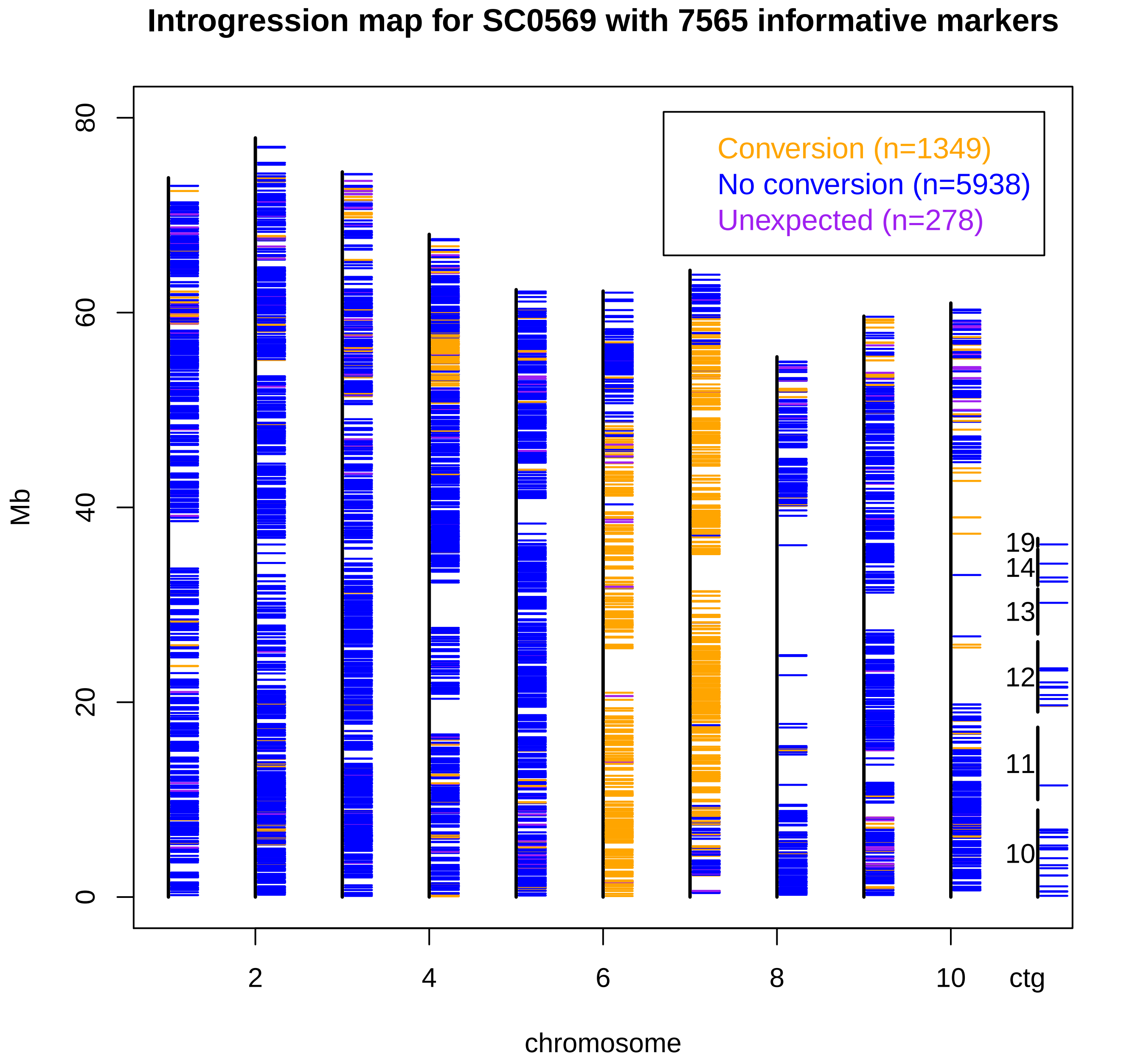
Introgression map for SC0569 with 7565 informative markers
Conversion (n=1349)
No conversion (n=5938)
Unexpected (n=278)
Mb
80
60
40
20
0
chromosome
2
4
6
8
10
ctg
19
14
13
12
11
10

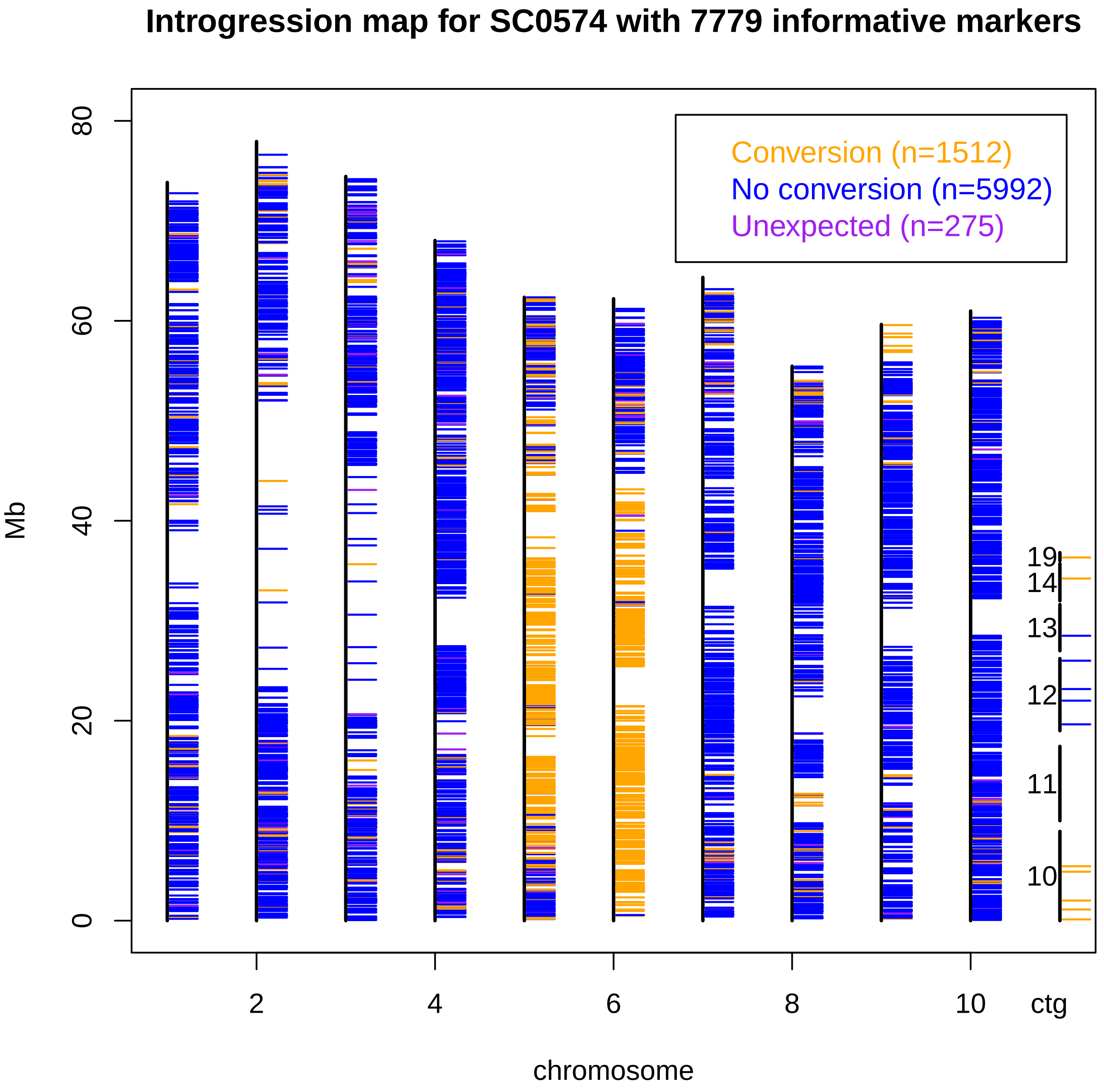
Introgression map for SC0574 with 7779 informative markers
Conversion (n=1512)
No conversion (n=5992)
Unexpected (n=275)
Mb
0
20
40
60
80
chromosome
2
4
6
8
10
ctg
19
14
13
12
11
10

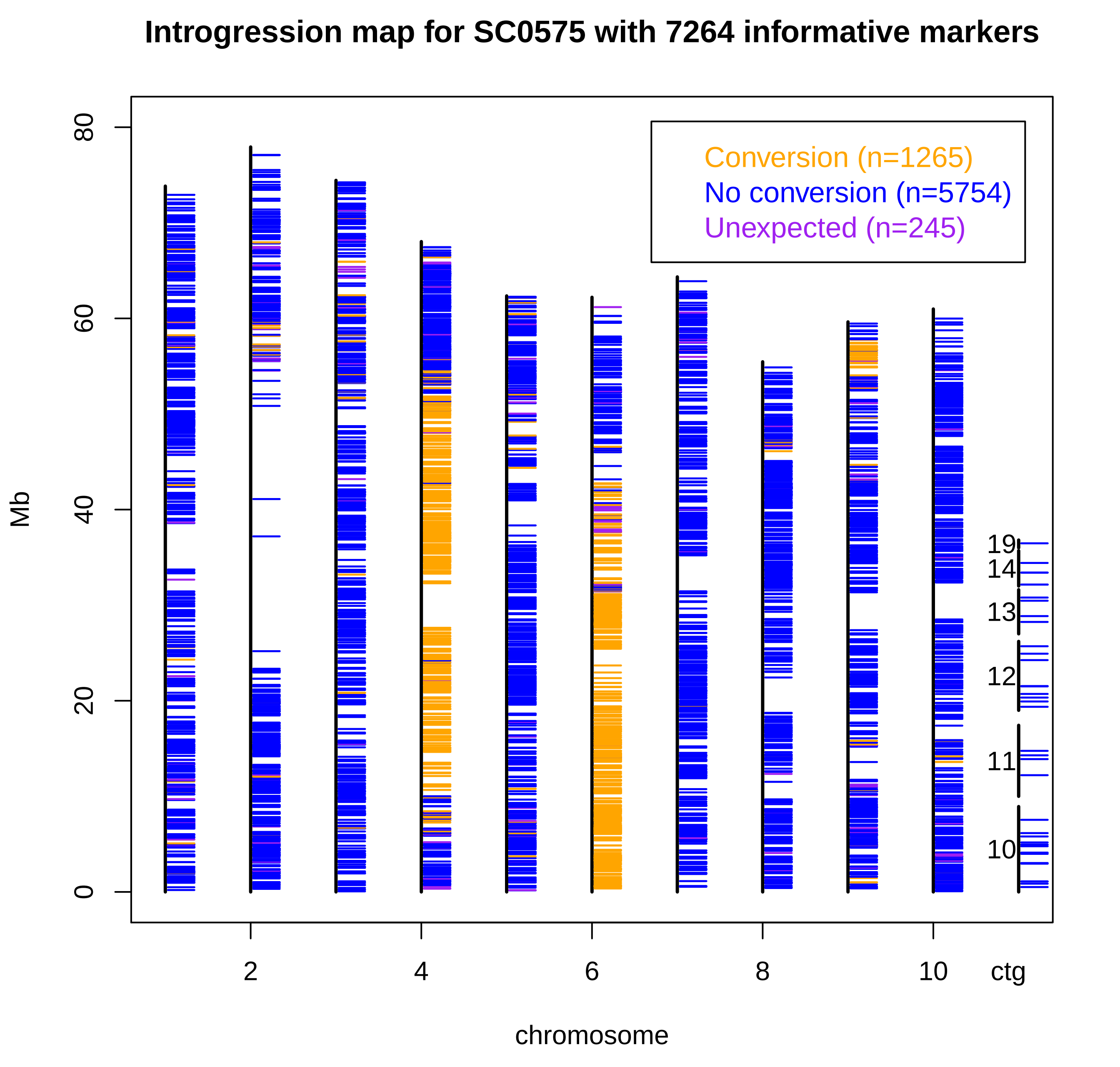
Introgression map for SC0575 with 7264 informative markers
Conversion (n=1265)
No conversion (n=5754)
Unexpected (n=245)
Mb
0
20
40
60
80
2
4
6
8
10
ctg
19
14
13
12
11
10
chromosome

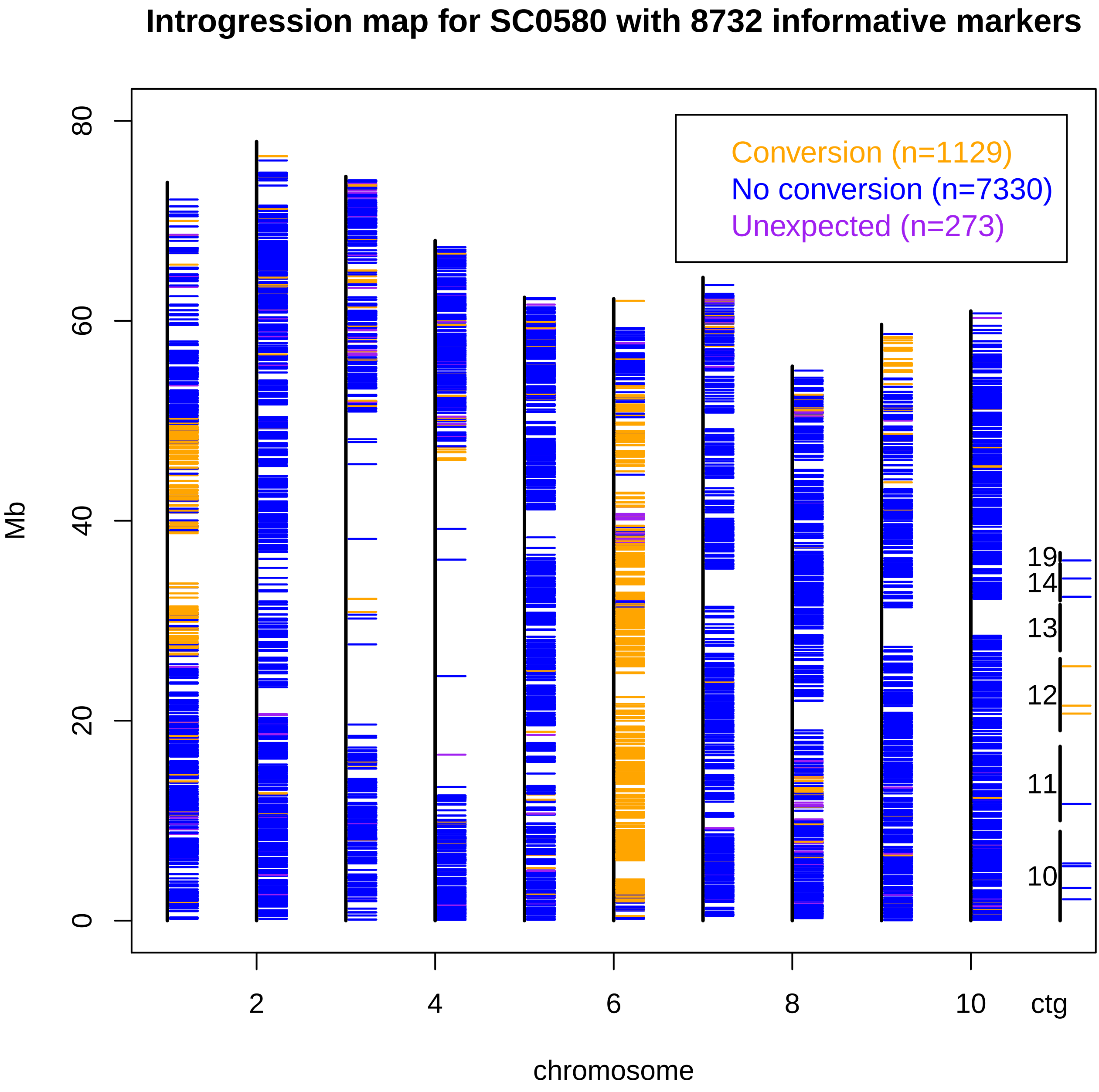

Introgression map for SC0580 with 8732 informative markers
Conversion (n=1129)
No conversion (n=7330)
Unexpected (n=273)
Mb
0
20
40
60
80
2
4
6
8
10
ctg
19
14
13
12
11
10
chromosome

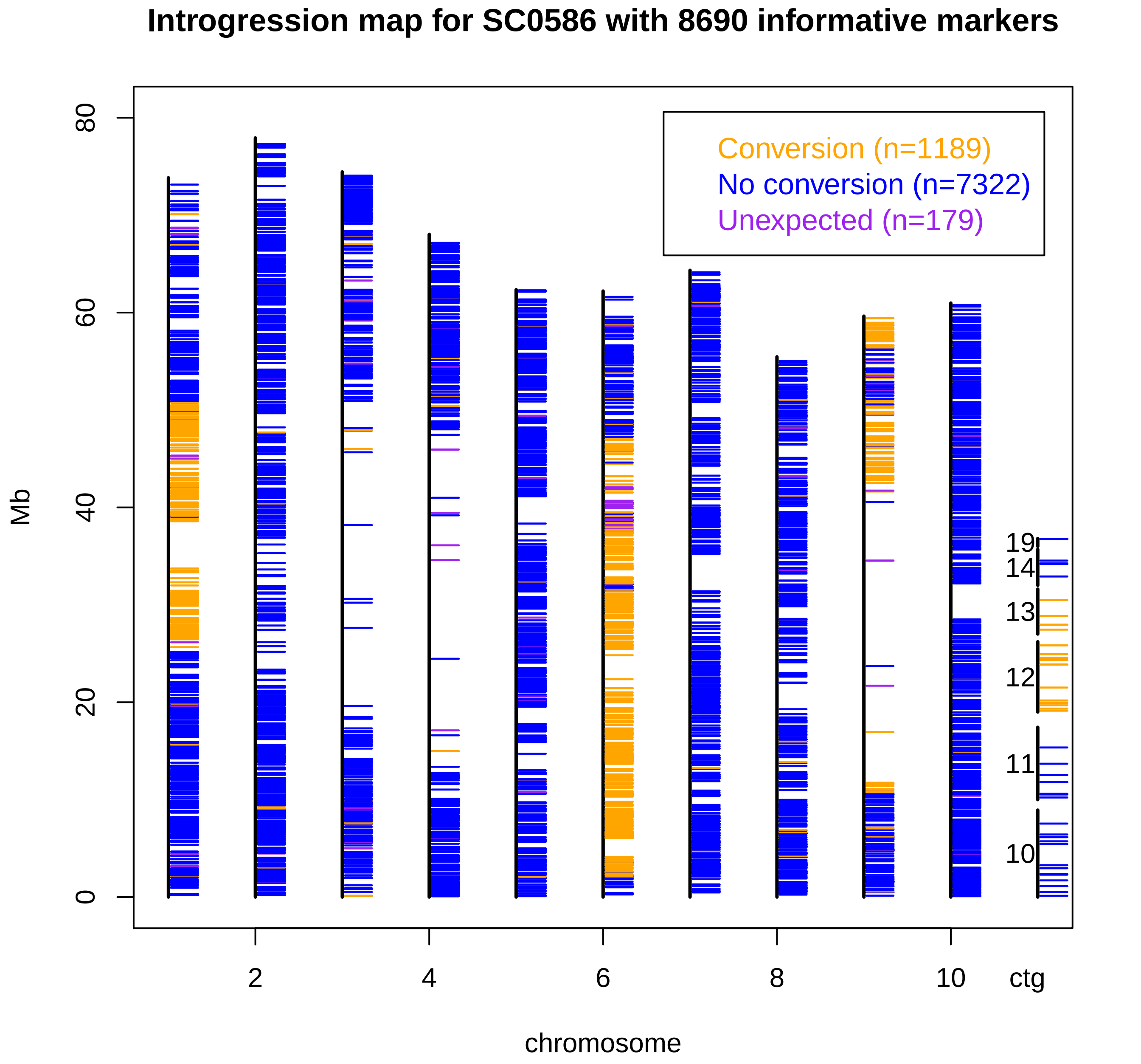
Introgression map for SC0586 with 8690 informative markers
Conversion (n=1189)
No conversion (n=7322)
Unexpected (n=179)
Mb
0
20
40
60
80
chromosome
2
4
6
8
10
ctg
19
14
13
12
11
10

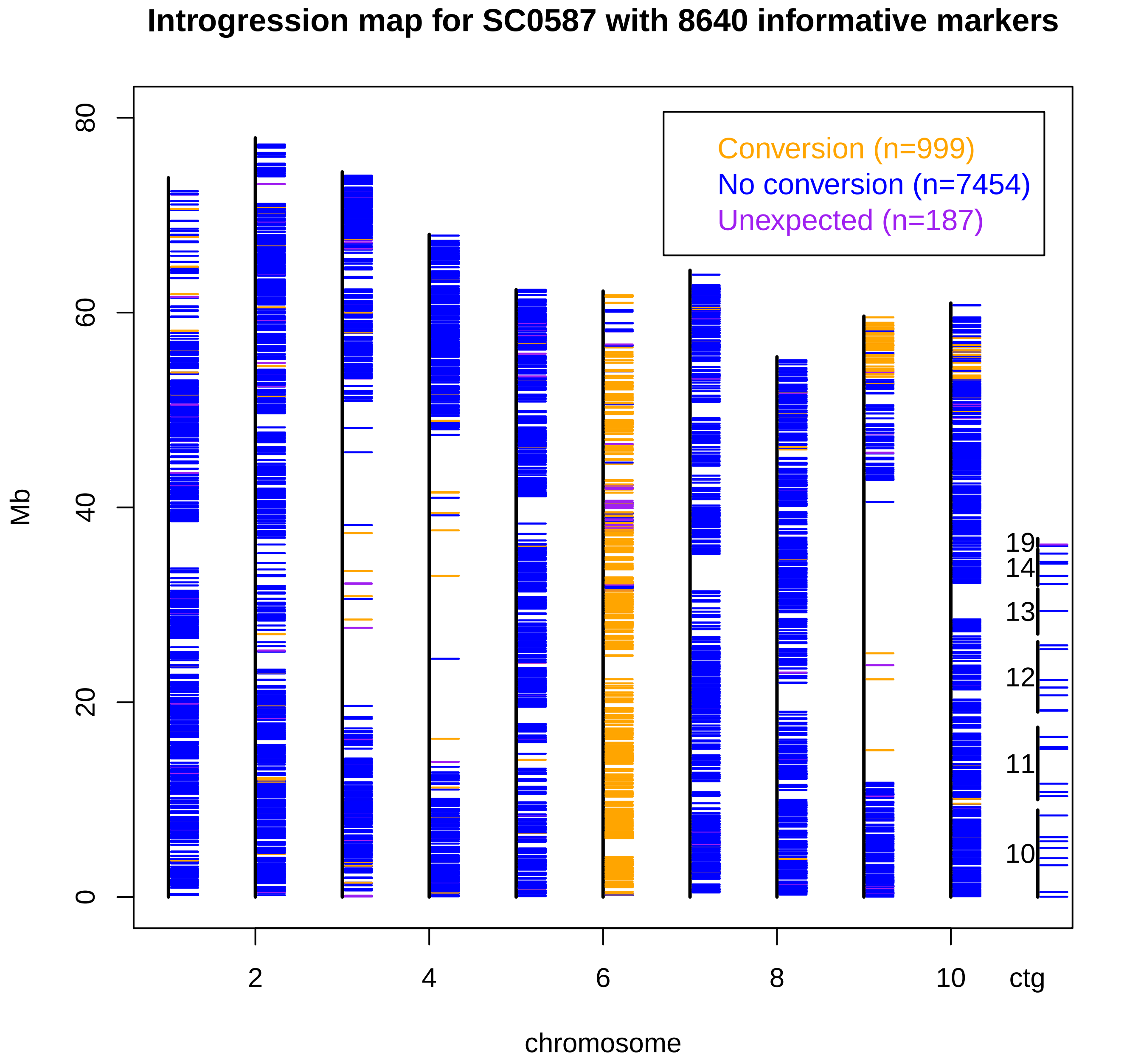

Introgression map for SC0587 with 8640 informative markers
Conversion (n=999)
No conversion (n=7454)
Unexpected (n=187)
Mb
0
20
40
60
80
chromosome
2
4
6
8
10
ctg
19
14
13
12
11
10

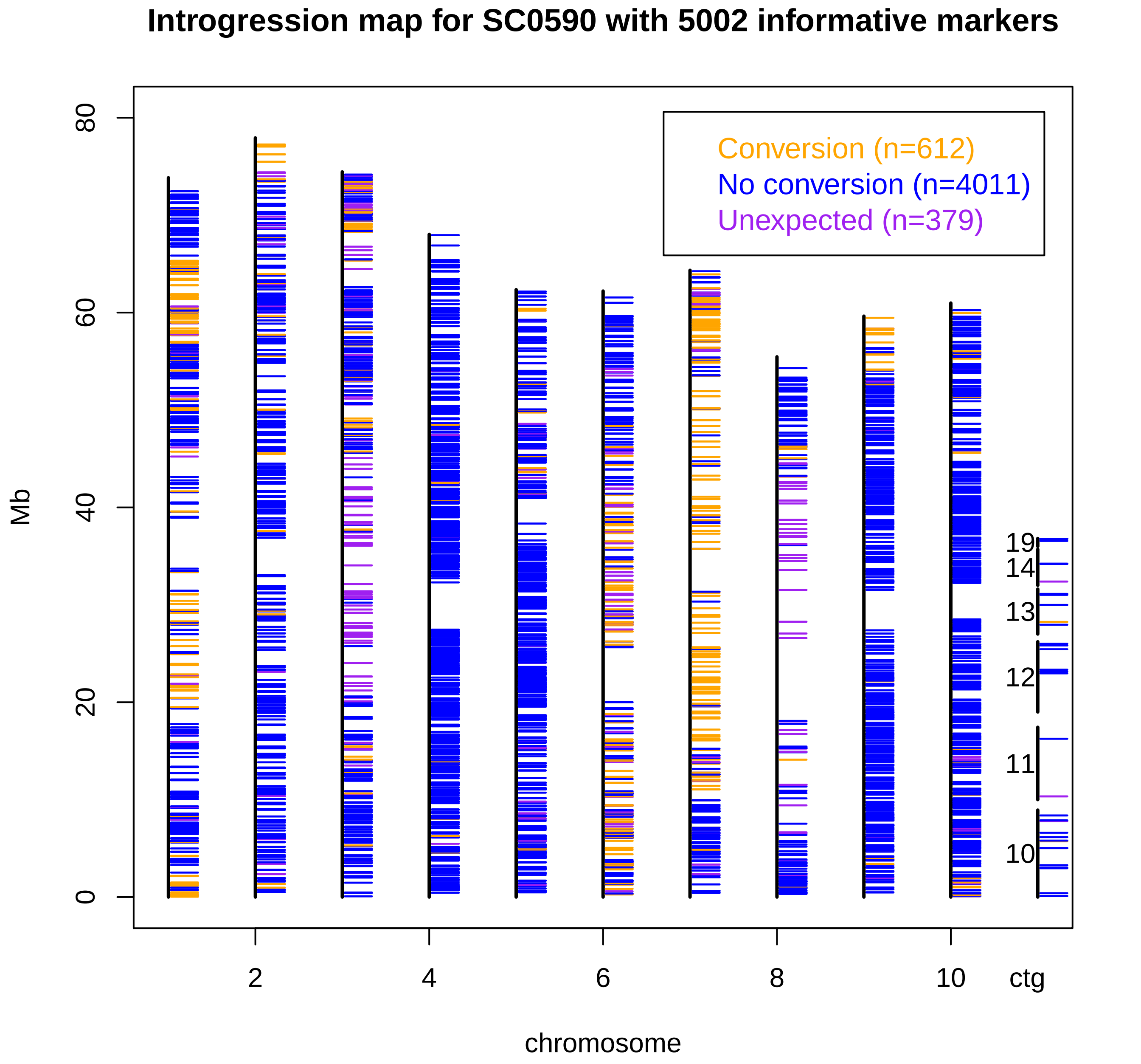

Introgression map for SC0590 with 5002 informative markers
Conversion (n=612)
No conversion (n=4011)
Unexpected (n=379)
Mb
0
20
40
60
80
chromosome
2
4
6
8
10
ctg
19
14
13
12
11
10

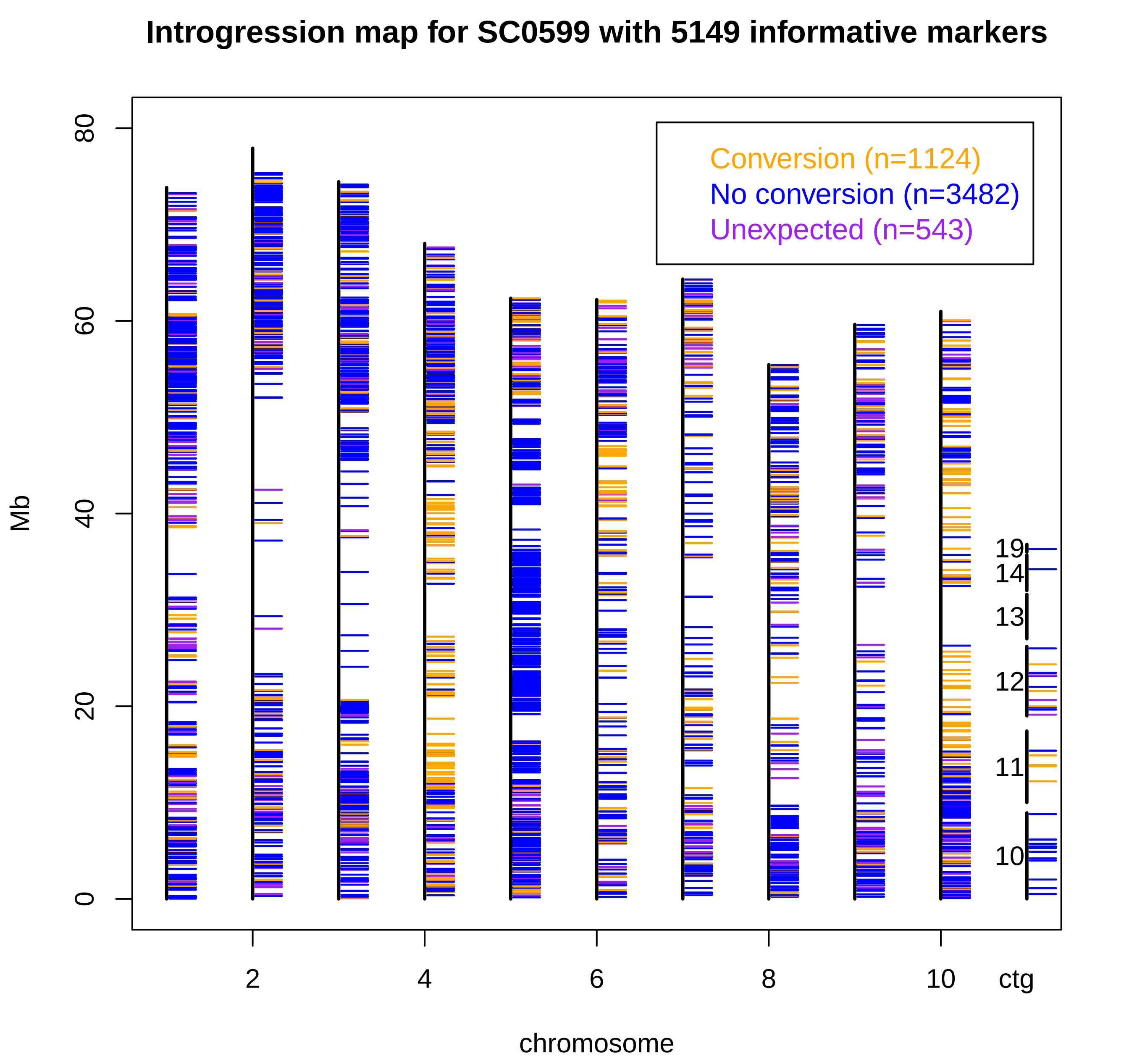

Introgression map for SC0599 with 5149 informative markers
Conversion (n=1124)
No conversion (n=3482)
Unexpected (n=543)
Mb
0
20
40
60
80
2
4
6
8
10
ctg
19
14
13
12
11
10
chromosome

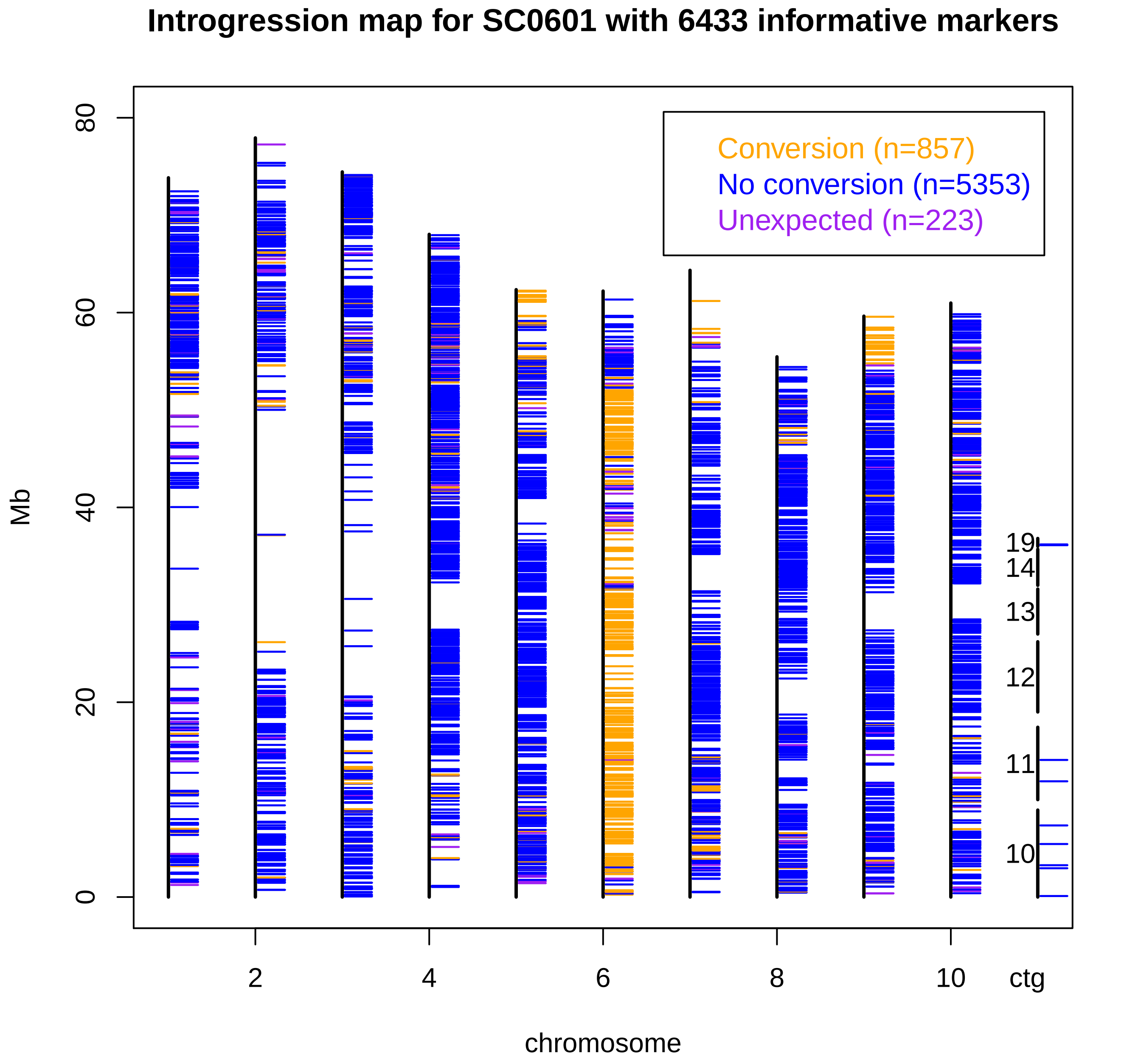

Introgression map for SC0601 with 6433 informative markers
Conversion (n=857)
No conversion (n=5353)
Unexpected (n=223)
Mb
0
20
40
60
80
chromosome
2
4
6
8
10
ctg
19
14
13
12
11
10

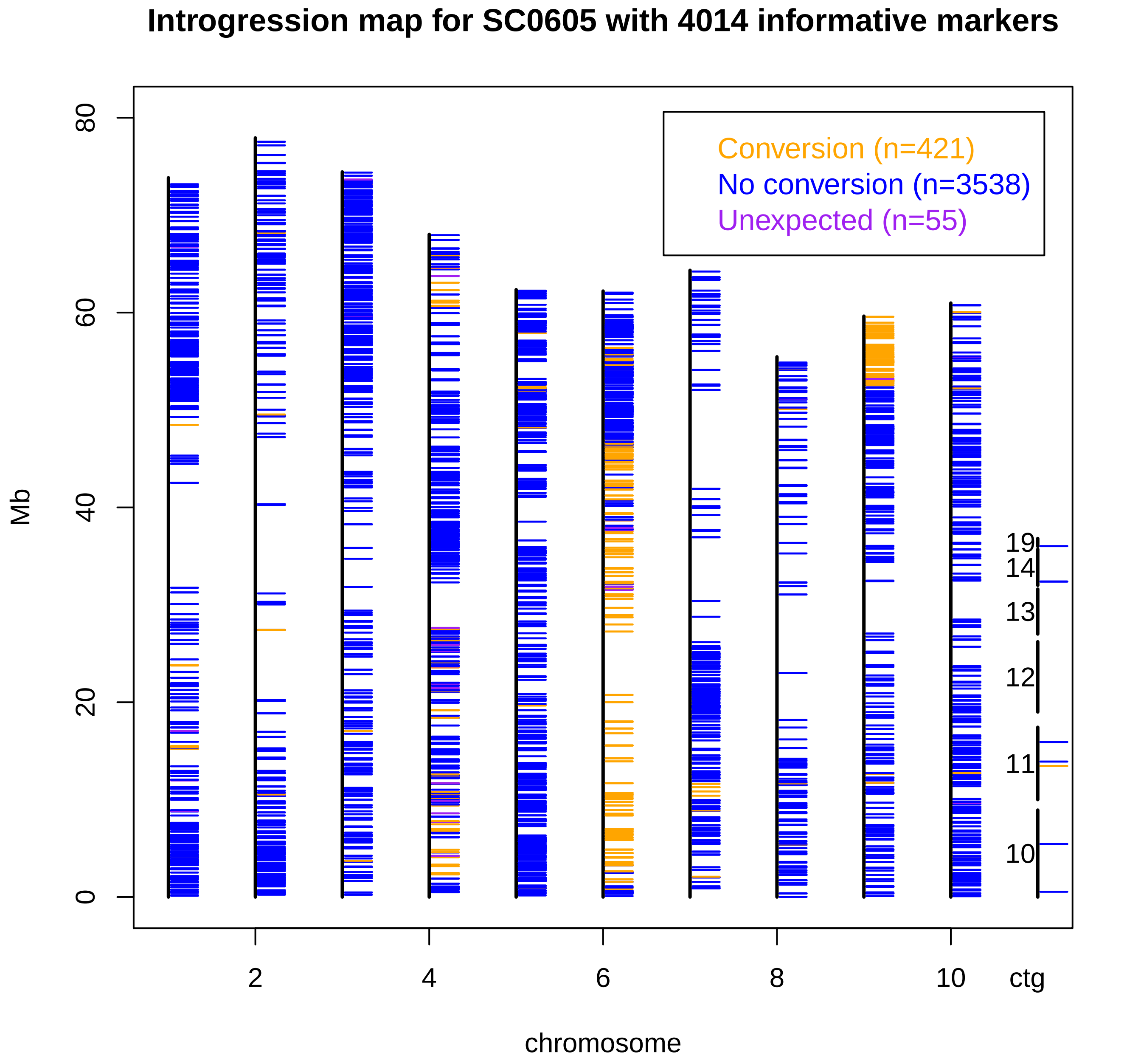

Introgression map for SC0605 with 4014 informative markers
Conversion (n=421)
No conversion (n=3538)
Unexpected (n=55)
Mb
0
20
40
60
80
chromosome
2
4
6
8
10
ctg
19
14
13
12
11
10

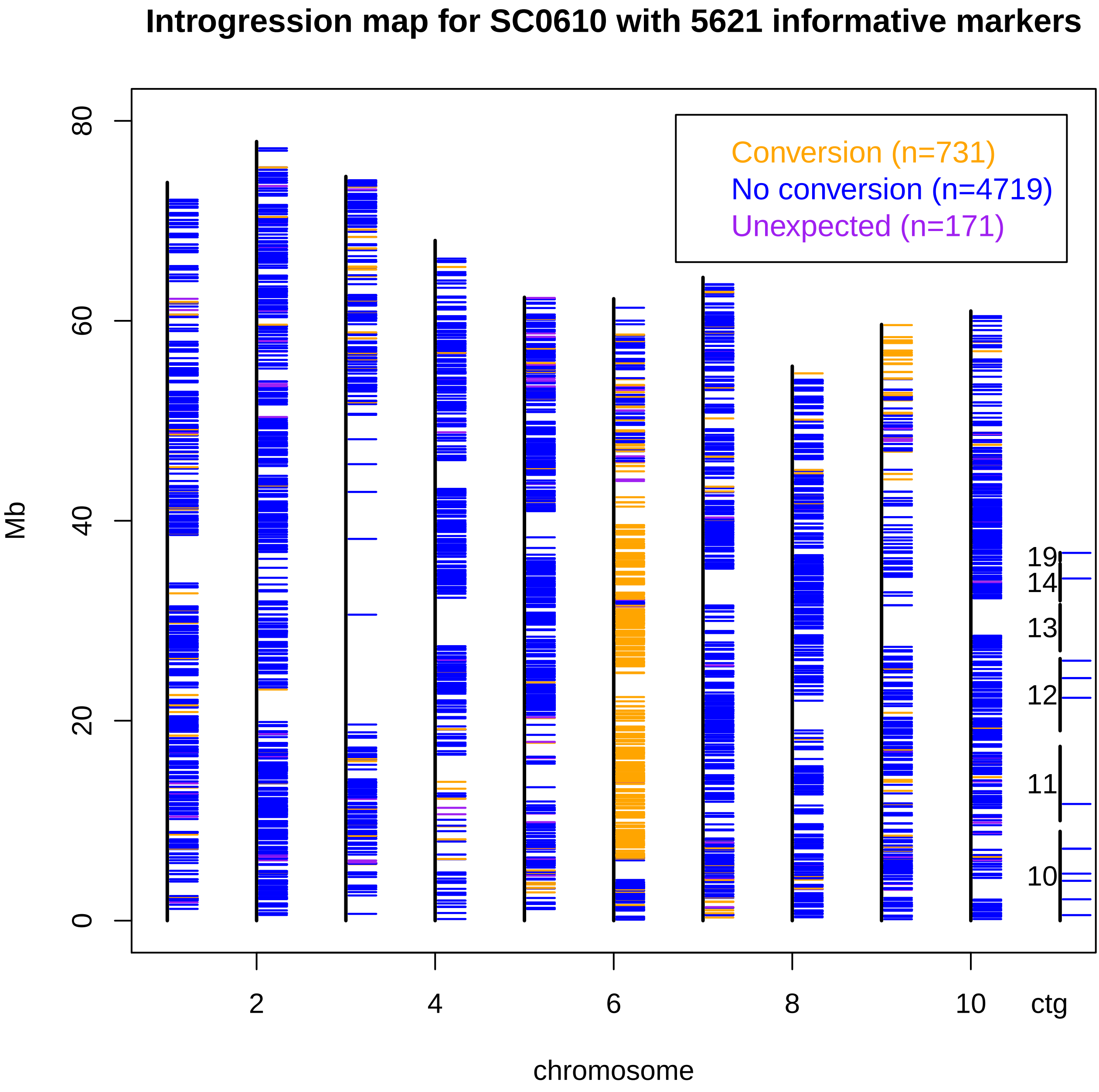

Introgression map for SC0610 with 5621 informative markers
Conversion (n=731)
No conversion (n=4719)
Unexpected (n=171)
Mb
0
20
40
60
80
chromosome
2
4
6
8
10
ctg
19
14
13
12
11
10

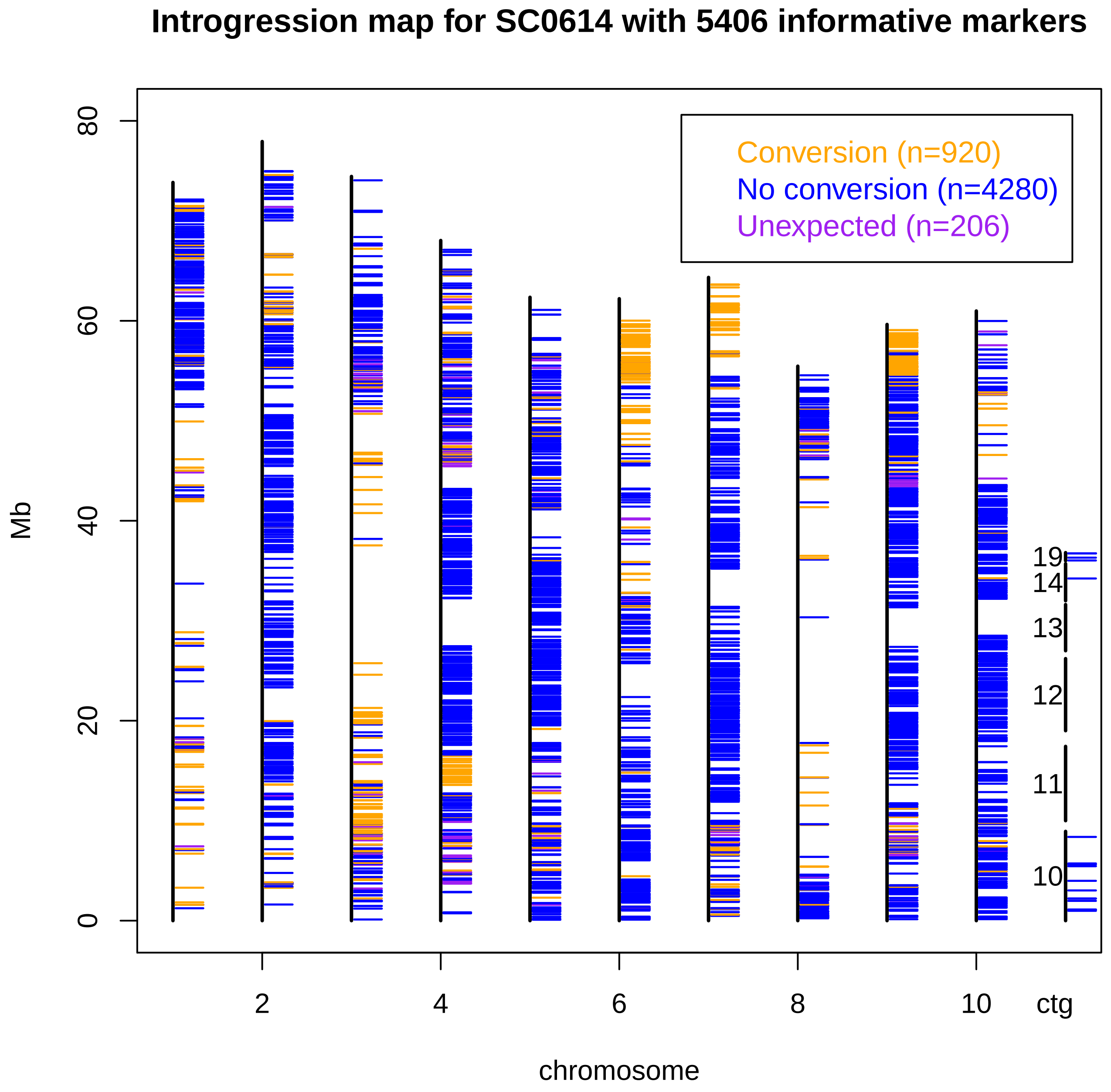

Introgression map for SC0614 with 5406 informative markers
Conversion (n=920)
No conversion (n=4280)
Unexpected (n=206)
Mb
0
20
40
60
80
2
4
6
8
10
ctg
19
14
13
12
11
10
chromosome

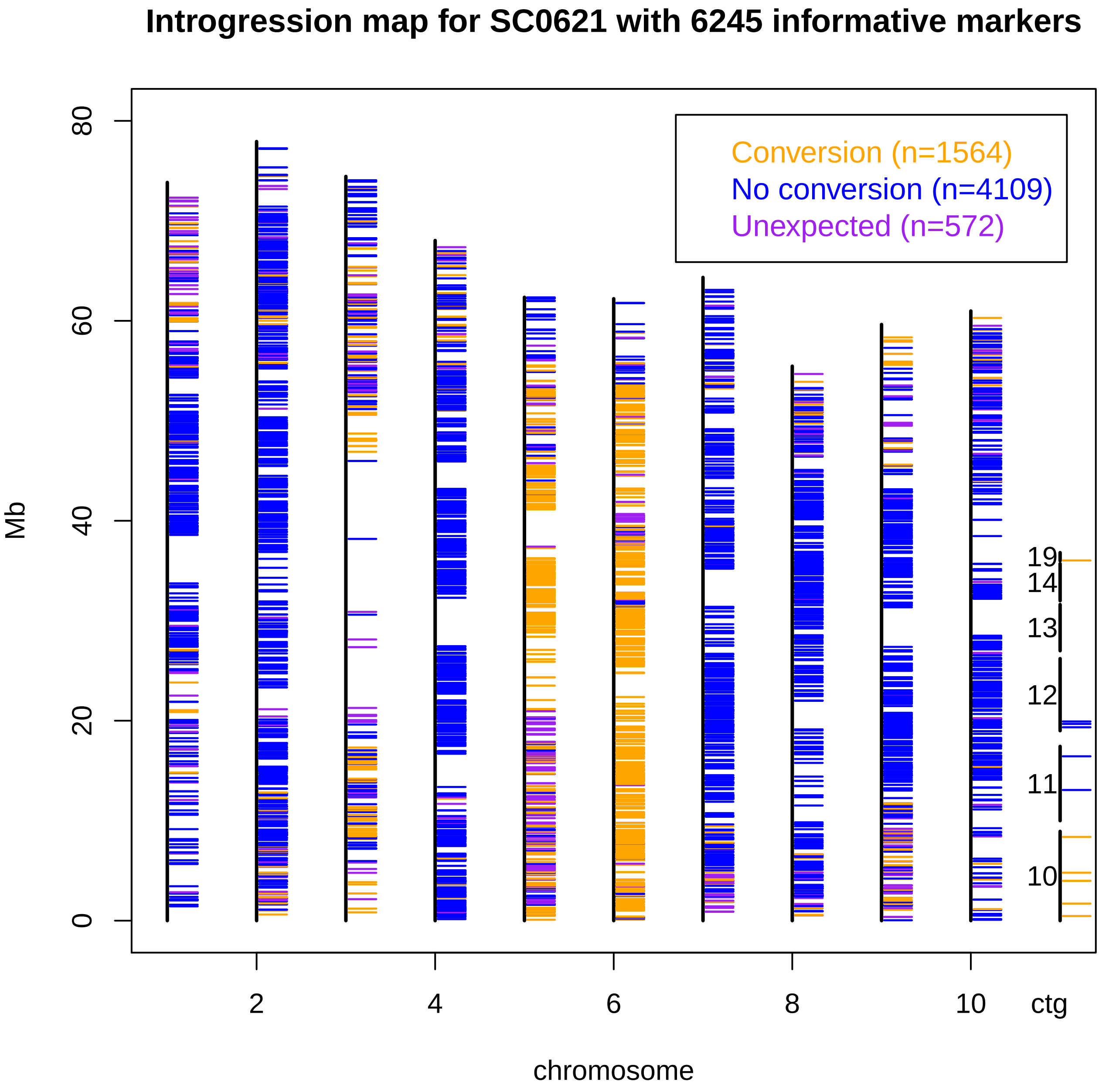
Introgression map for SC0621 with 6245 informative markers
Conversion (n=1564)
No conversion (n=4109)
Unexpected (n=572)
Mb
0
20
40
60
80
2
4
6
8
10
ctg
19
14
13
12
11
10
chromosome

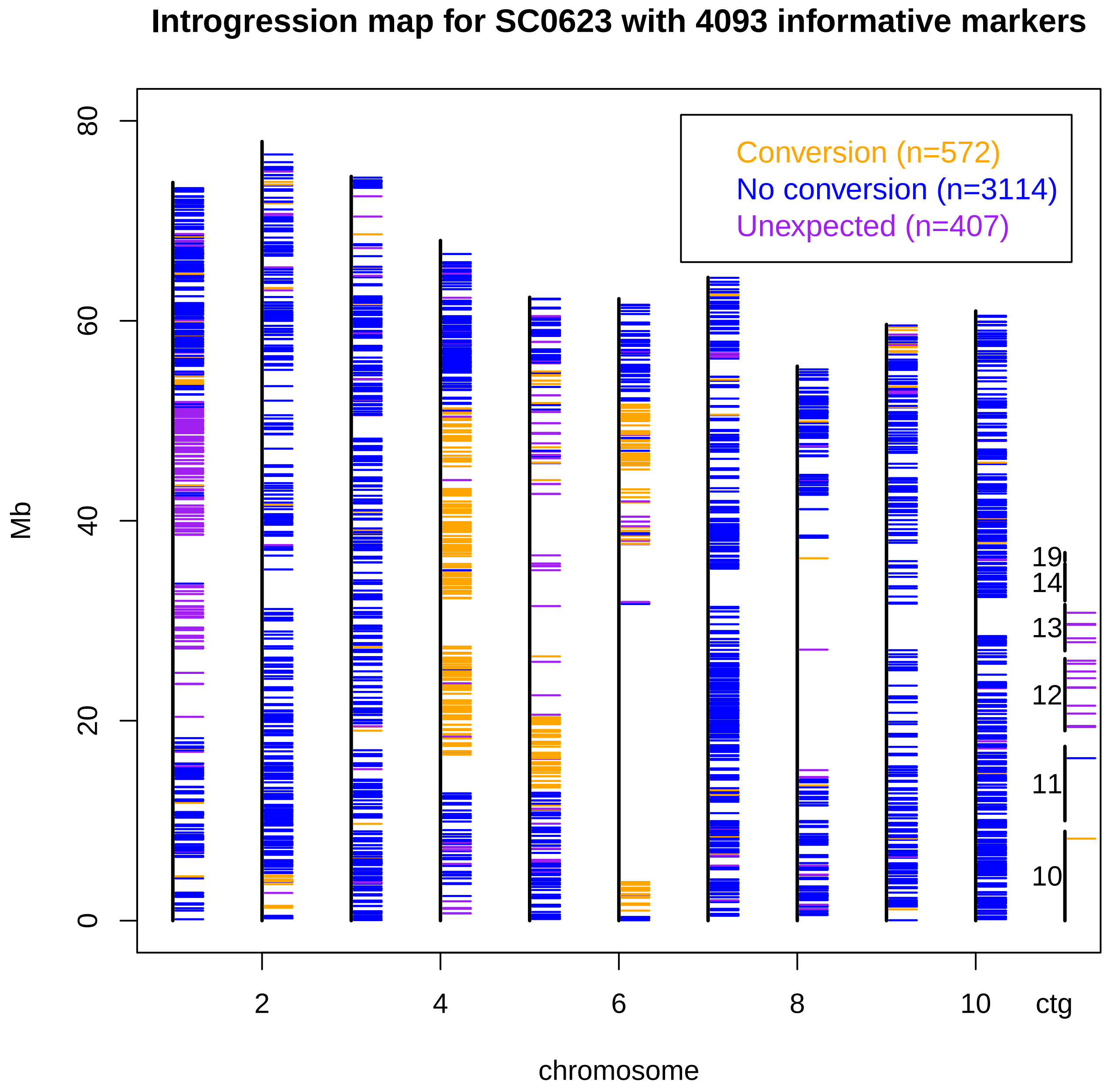

Introgression map for SC0623 with 4093 informative markers
Conversion (n=572)
No conversion (n=3114)
Unexpected (n=407)
Mb
0
20
40
60
80
2
4
6
8
10
ctg
19
14
13
12
11
10
chromosome

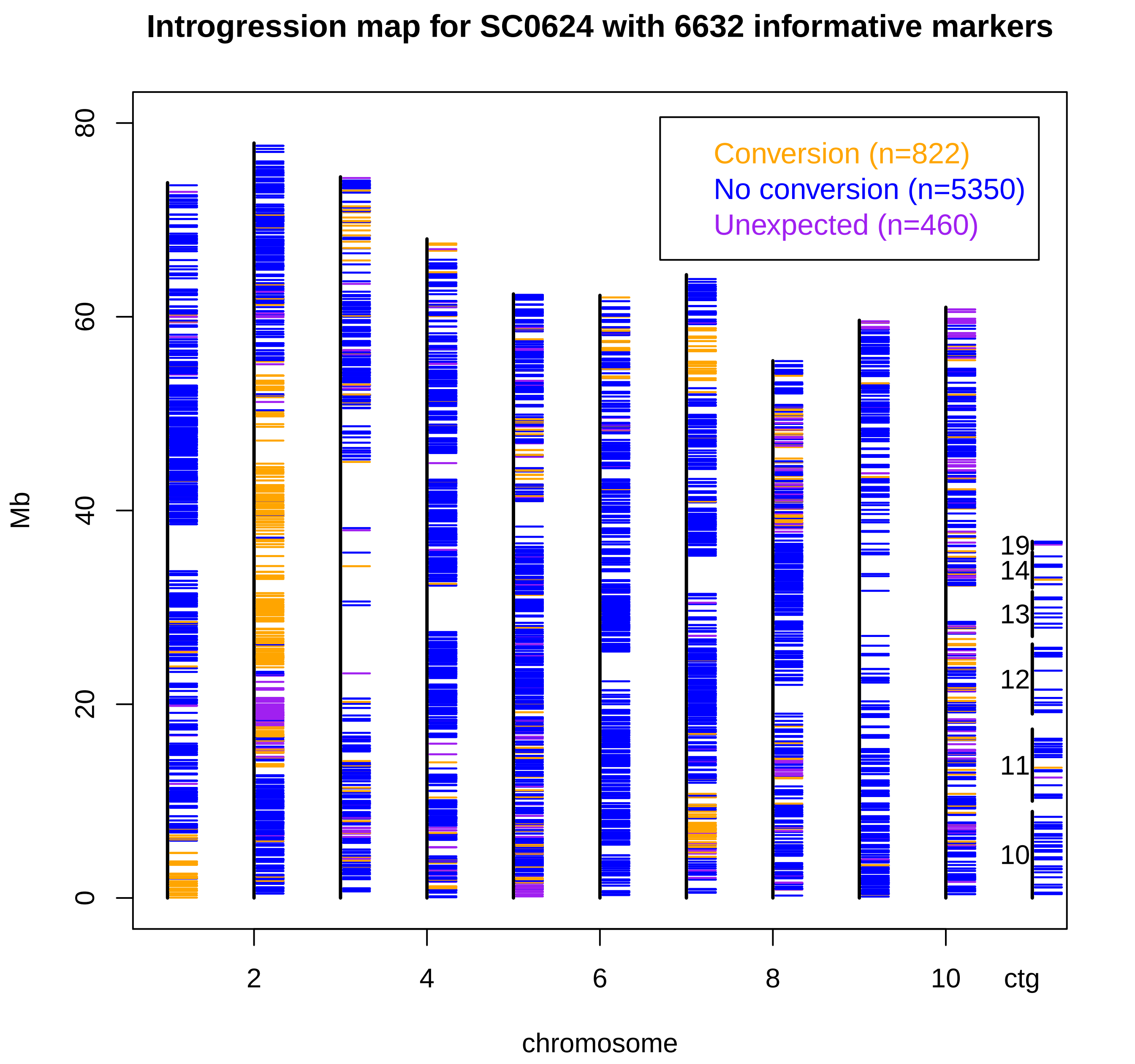
Introgression map for SC0624 with 6632 informative markers
Conversion (n=822)
No conversion (n=5350)
Unexpected (n=460)
Mb
0
20
40
60
80
chromosome
2
4
6
8
10
ctg
19
14
13
12
11
10

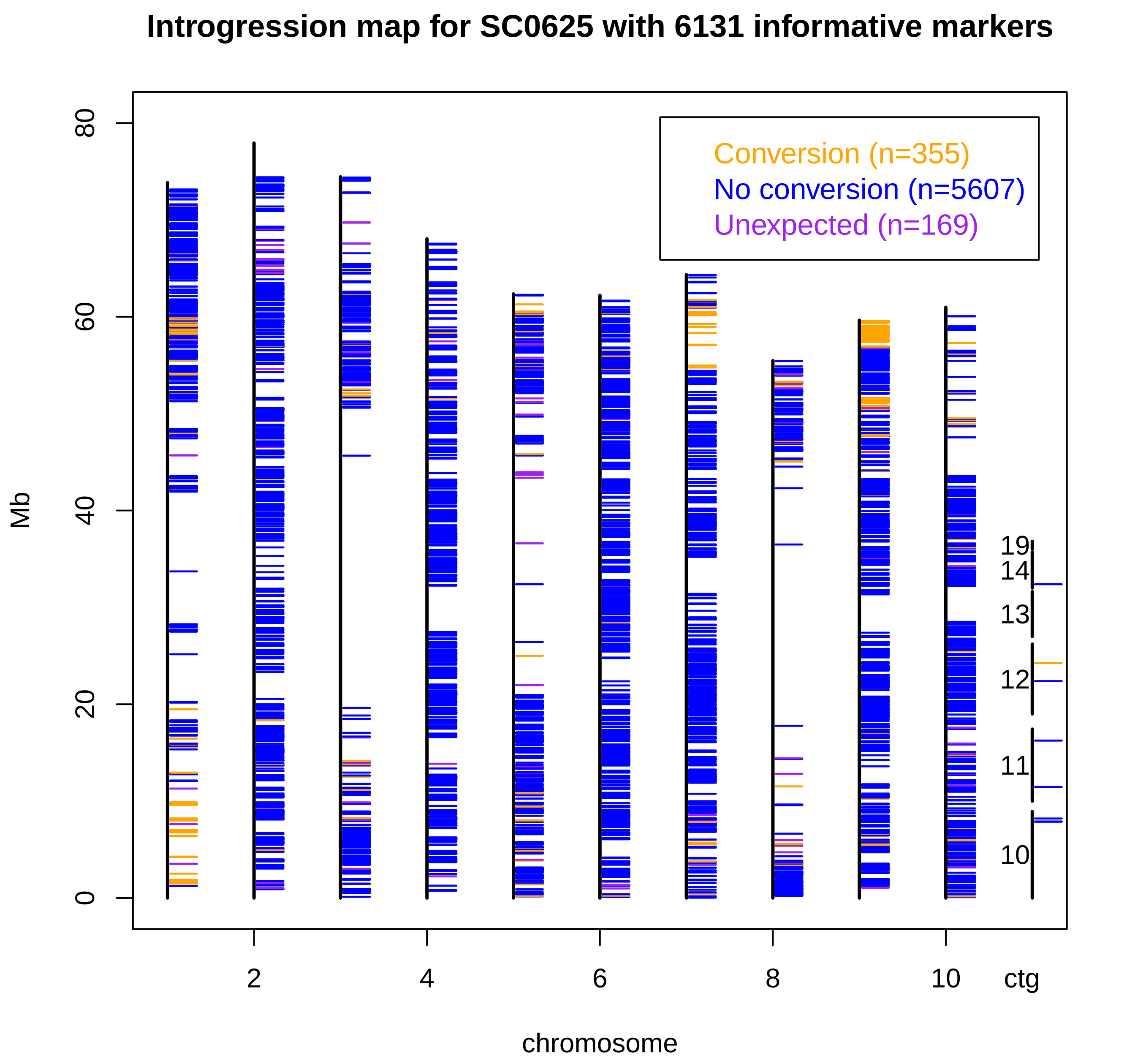

Introgression map for SC0625 with 6131 informative markers
Conversion (n=355)
No conversion (n=5607)
Unexpected (n=169)
Mb
0
20
40
60
80
chromosome
2
4
6
8
10
ctg
19
14
13
12
11
10

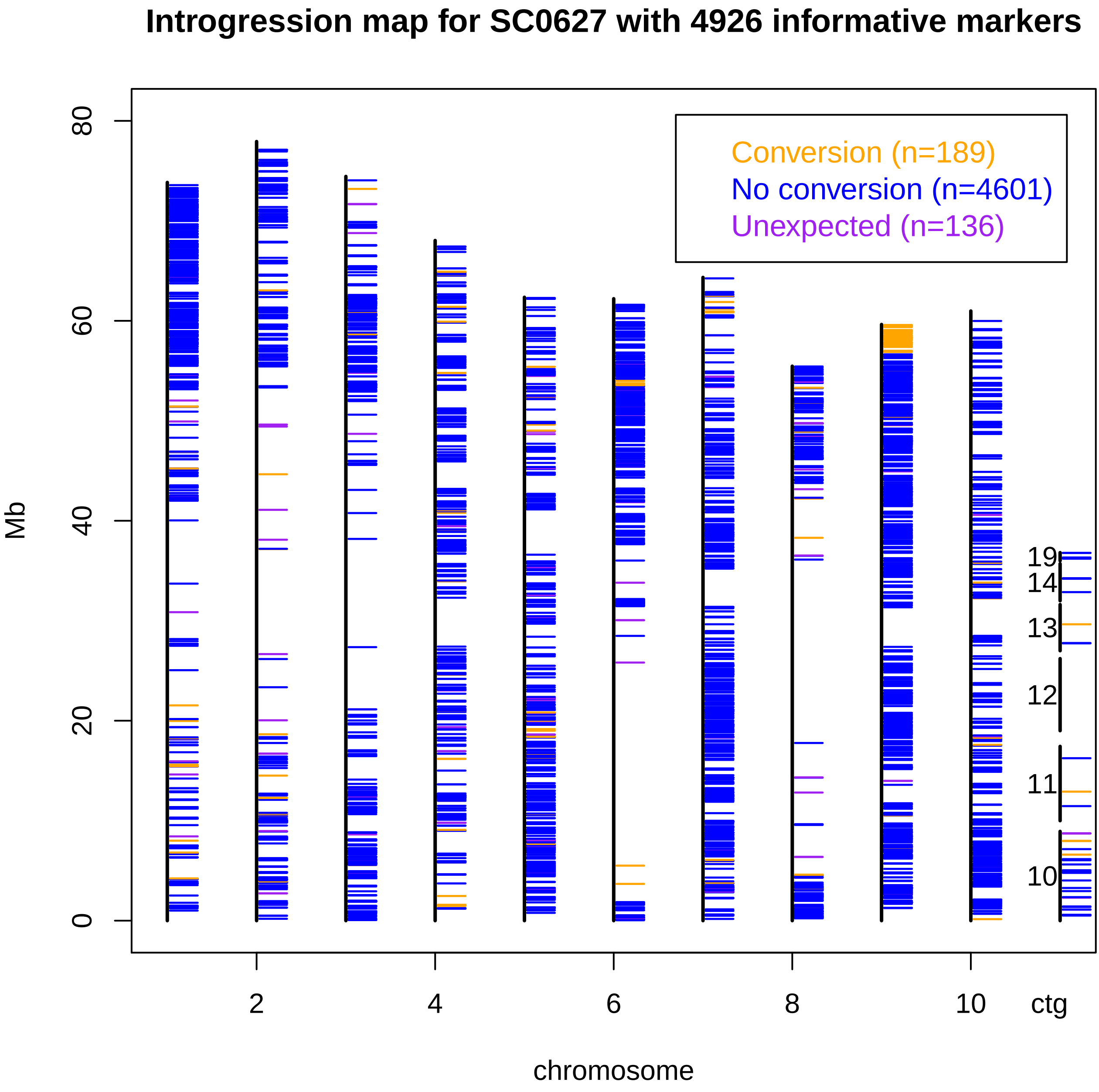

Introgression map for SC0627 with 4926 informative markers
Conversion (n=189)
No conversion (n=4601)
Unexpected (n=136)
Mb
0
20
40
60
80
chromosome
2
4
6
8
10
ctg
19
14
13
12
11
10

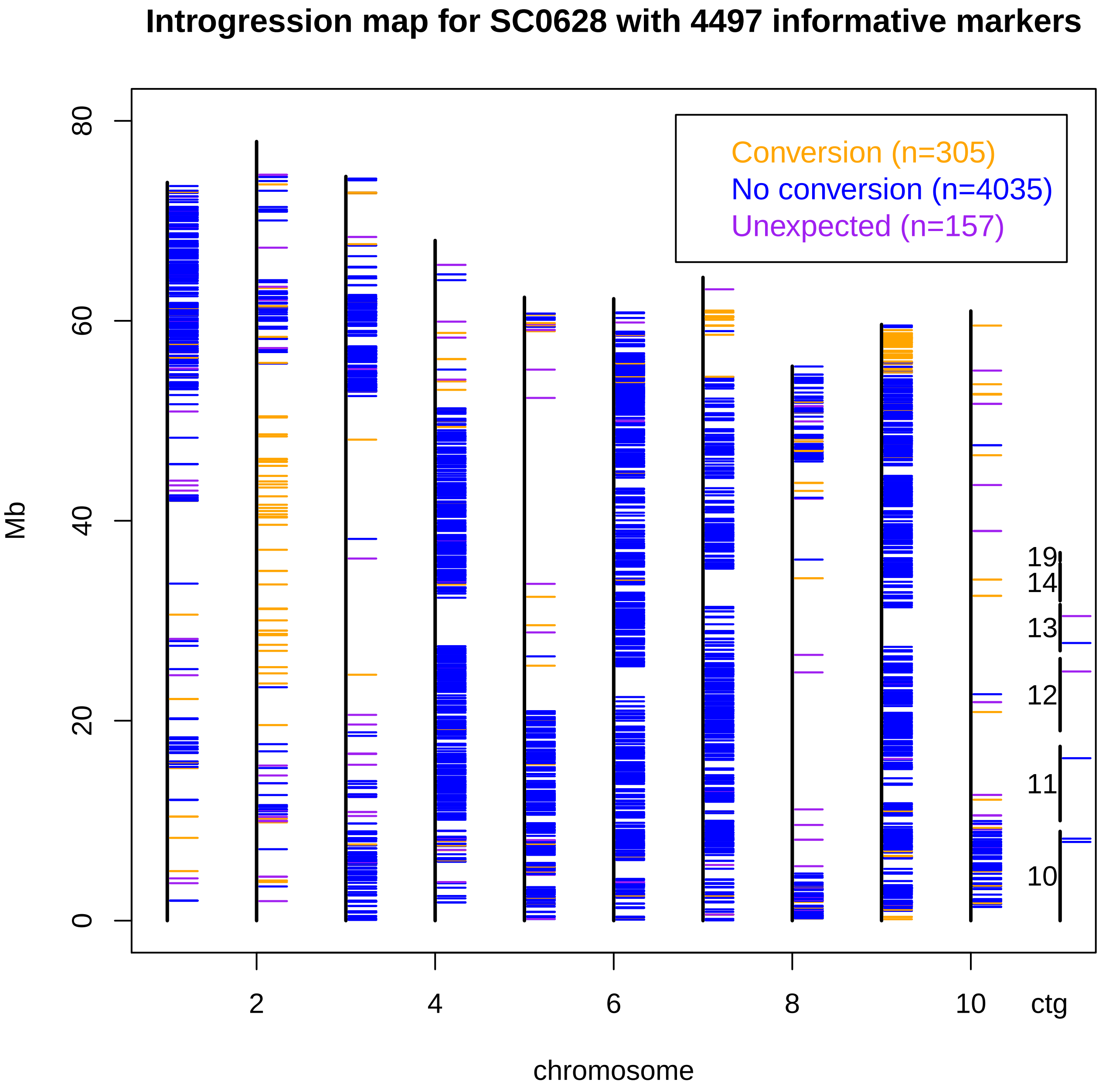
Introgression map for SC0628 with 4497 informative markers
Conversion (n=305)
No conversion (n=4035)
Unexpected (n=157)
Mb
0
20
40
60
80
chromosome
2
4
6
8
10
ctg
19
14
13
12
11
10

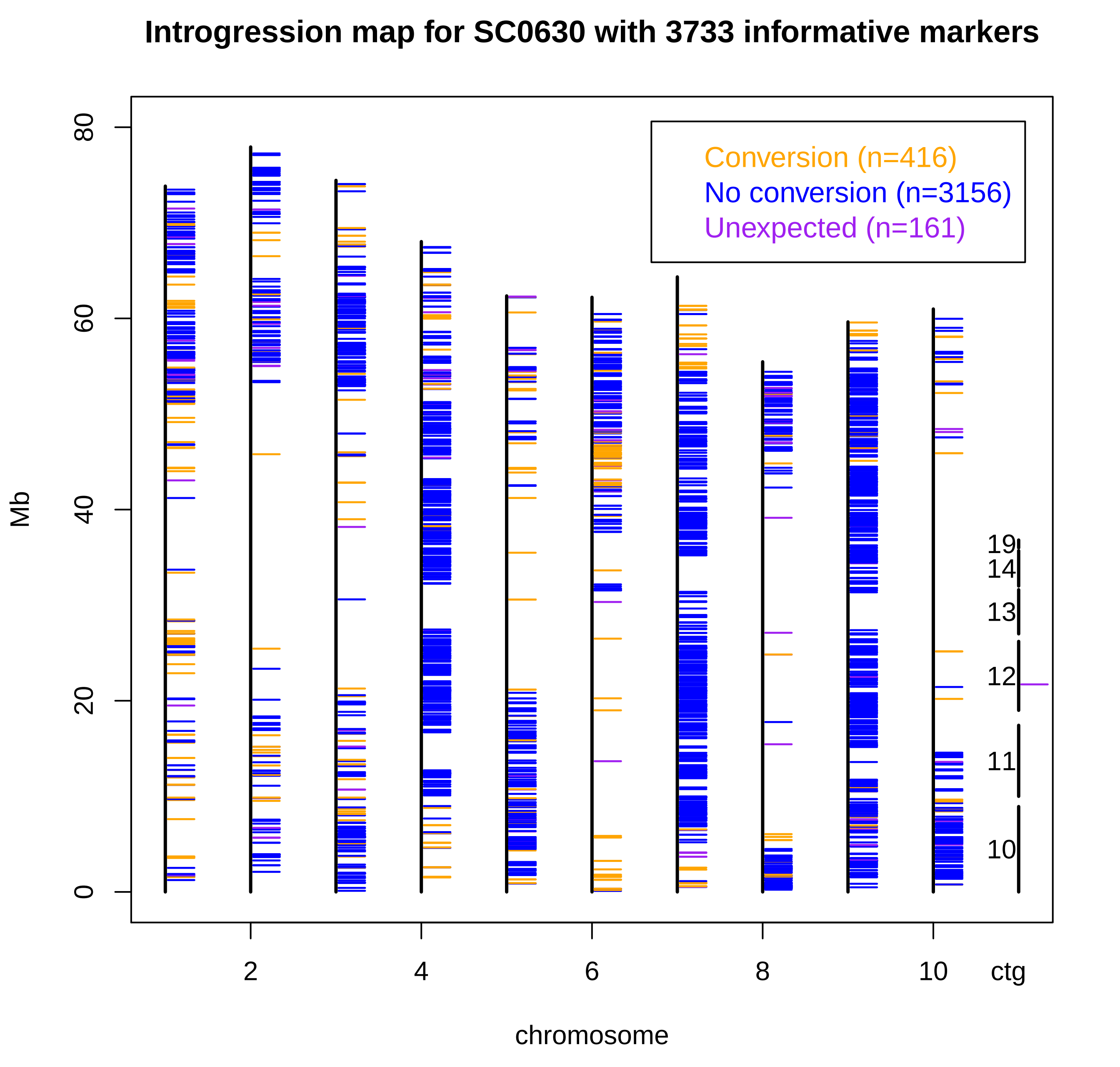

Introgression map for SC0630 with 3733 informative markers
Conversion (n=416)
No conversion (n=3156)
Unexpected (n=161)
Mb
0
20
40
60
80
2
4
6
8
10
ctg
19
14
13
12
11
10
chromosome

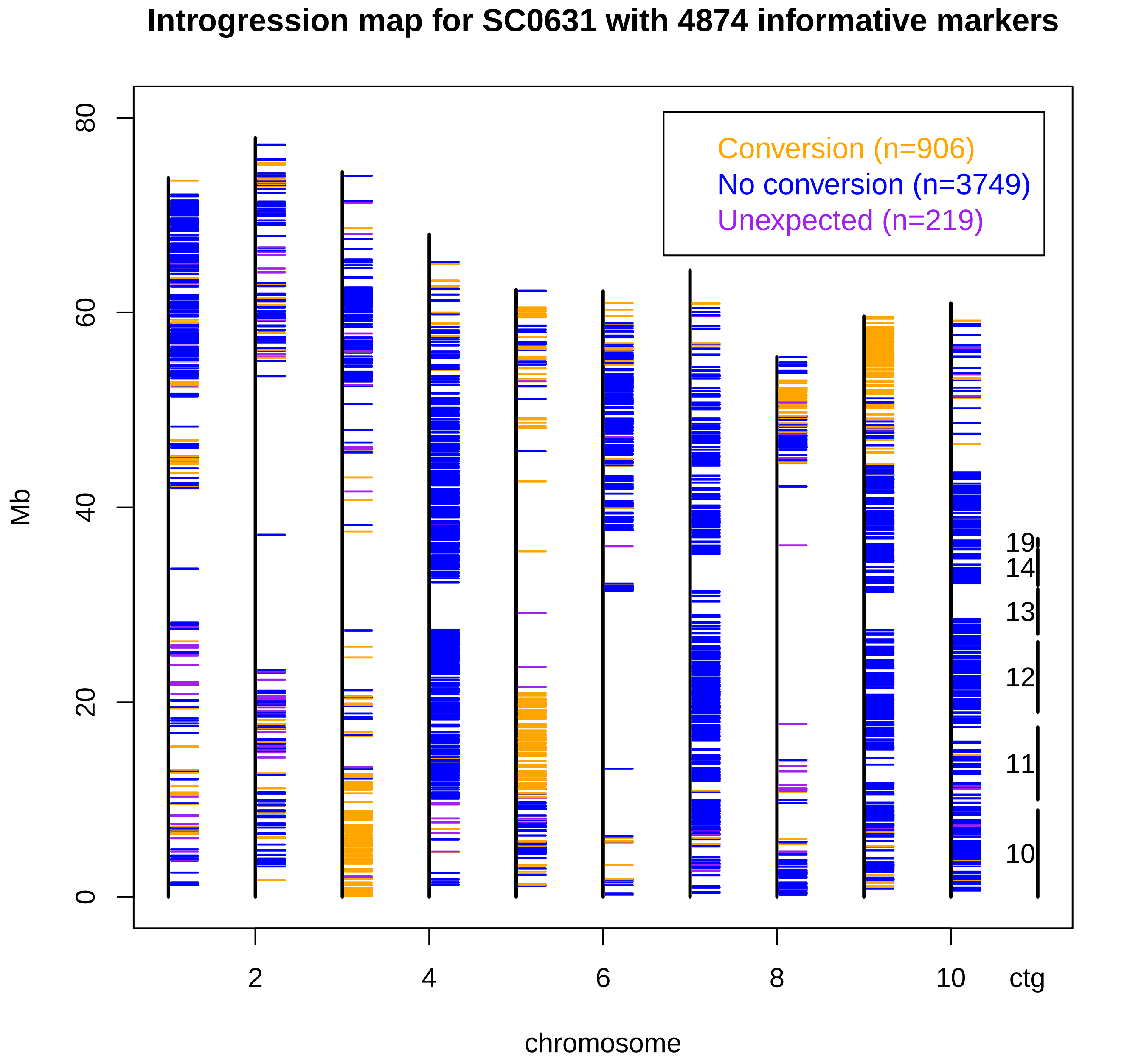

Introgression map for SC0631 with 4874 informative markers
Conversion (n=906)
No conversion (n=3749)
Unexpected (n=219)
Mb
0
20
40
60
80
2
4
6
8
10
ctg
chromosome
19
14
13
12
11
10

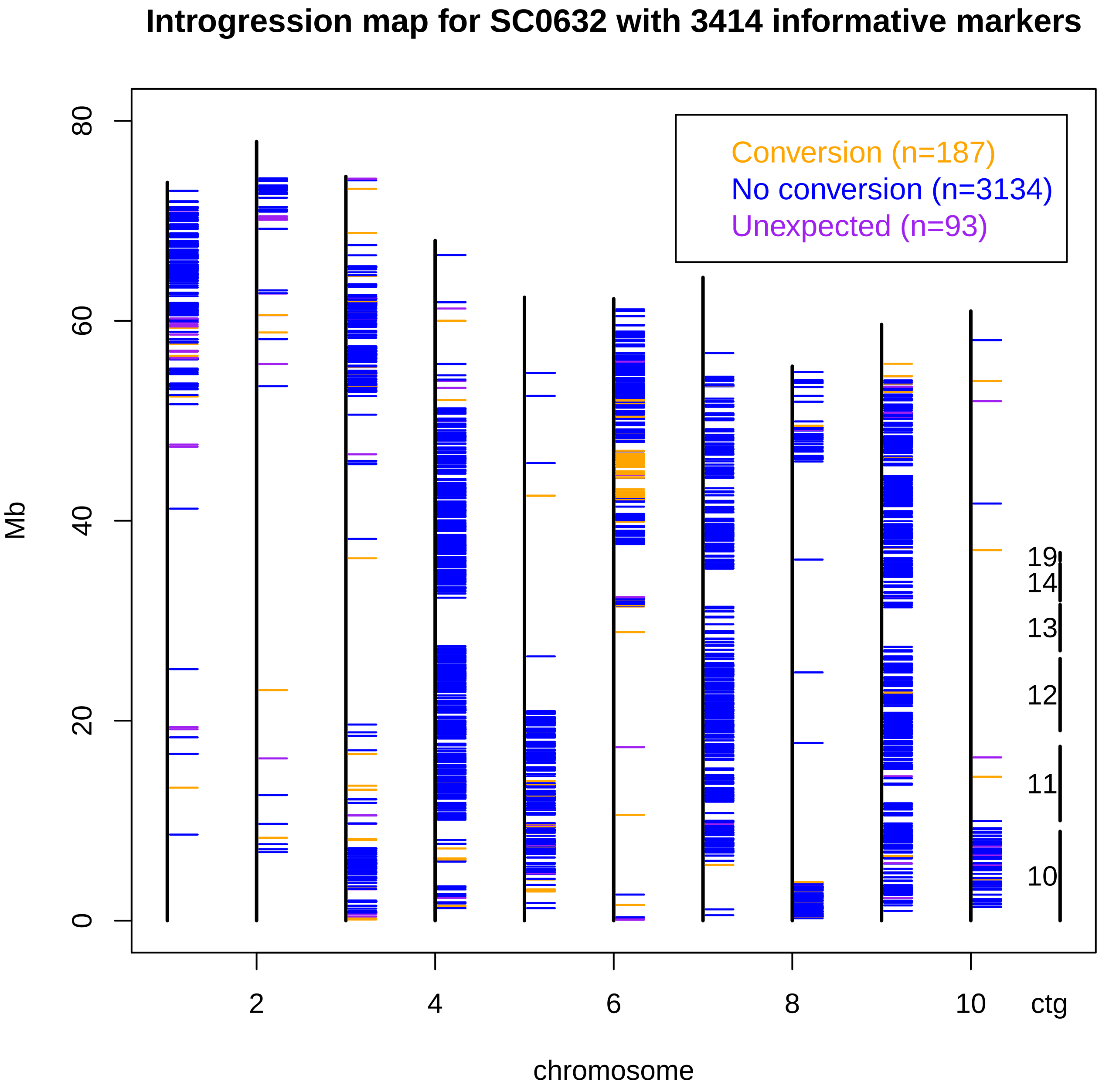

Introgression map for SC0632 with 3414 informative markers
Conversion (n=187)
No conversion (n=3134)
Unexpected (n=93)
Mb
0
20
40
60
80
2
4
6
8
10
ctg
19
14
13
12
11
10
chromosome

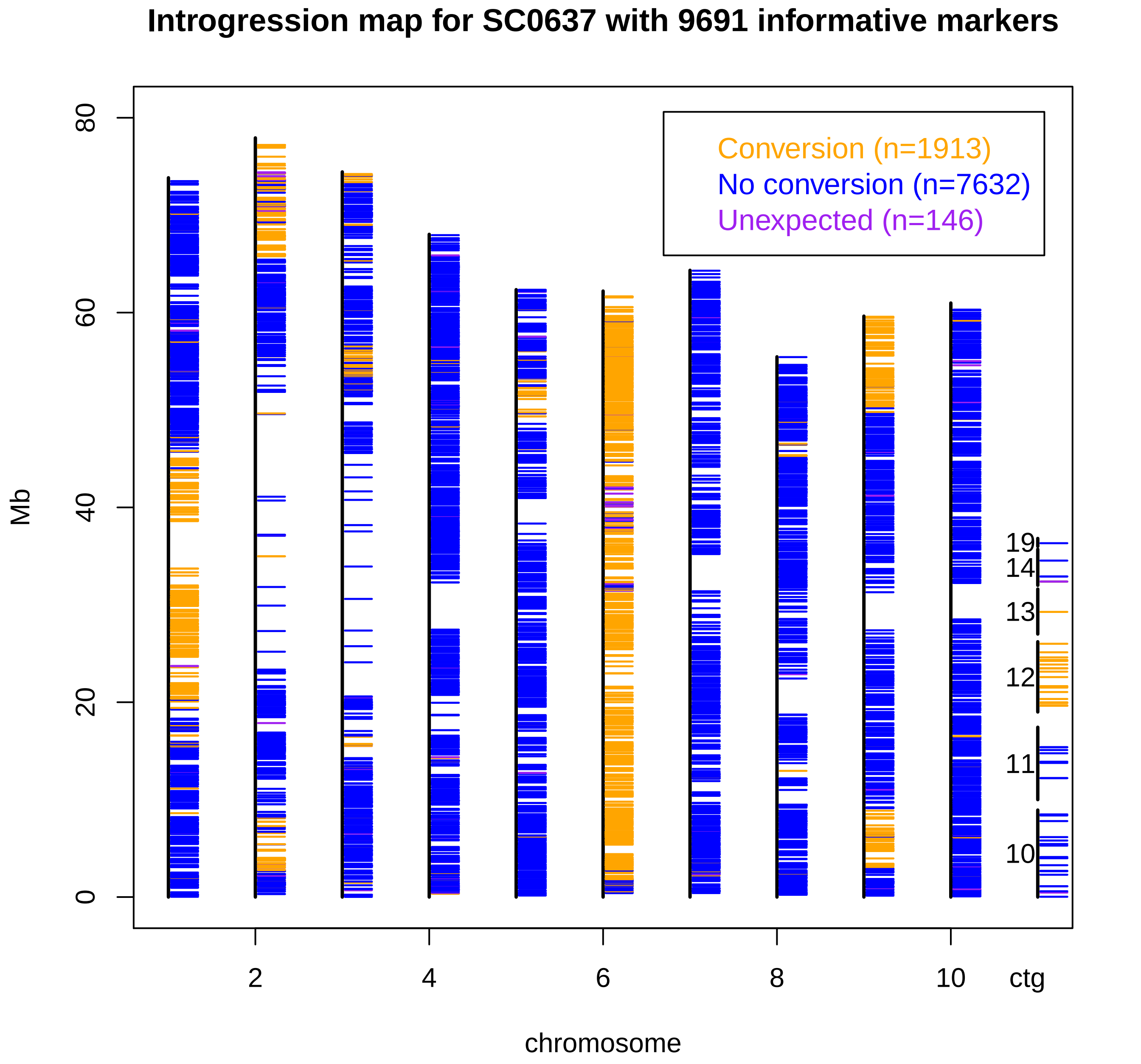
Introgression map for SC0637 with 9691 informative markers
Conversion (n=1913)
No conversion (n=7632)
Unexpected (n=146)
Mb
0
20
40
60
80
chromosome
2
4
6
8
10
ctg
19
14
13
12
11
10

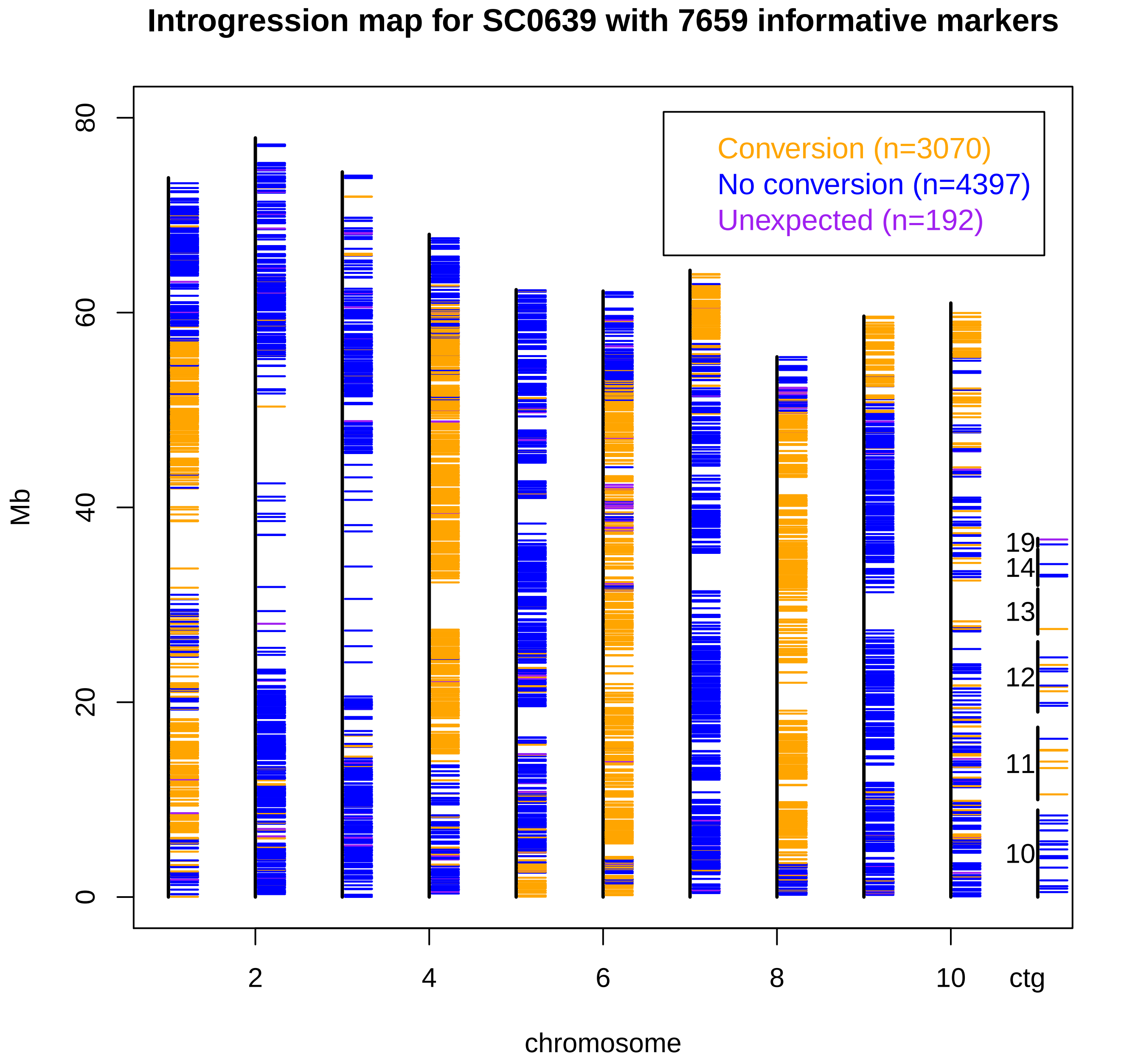

Introgression map for SC0639 with 7659 informative markers
Conversion (n=3070)
No conversion (n=4397)
Unexpected (n=192)
Mb
0
20
40
60
80
2
4
6
8
10
ctg
19
14
13
12
11
10
chromosome

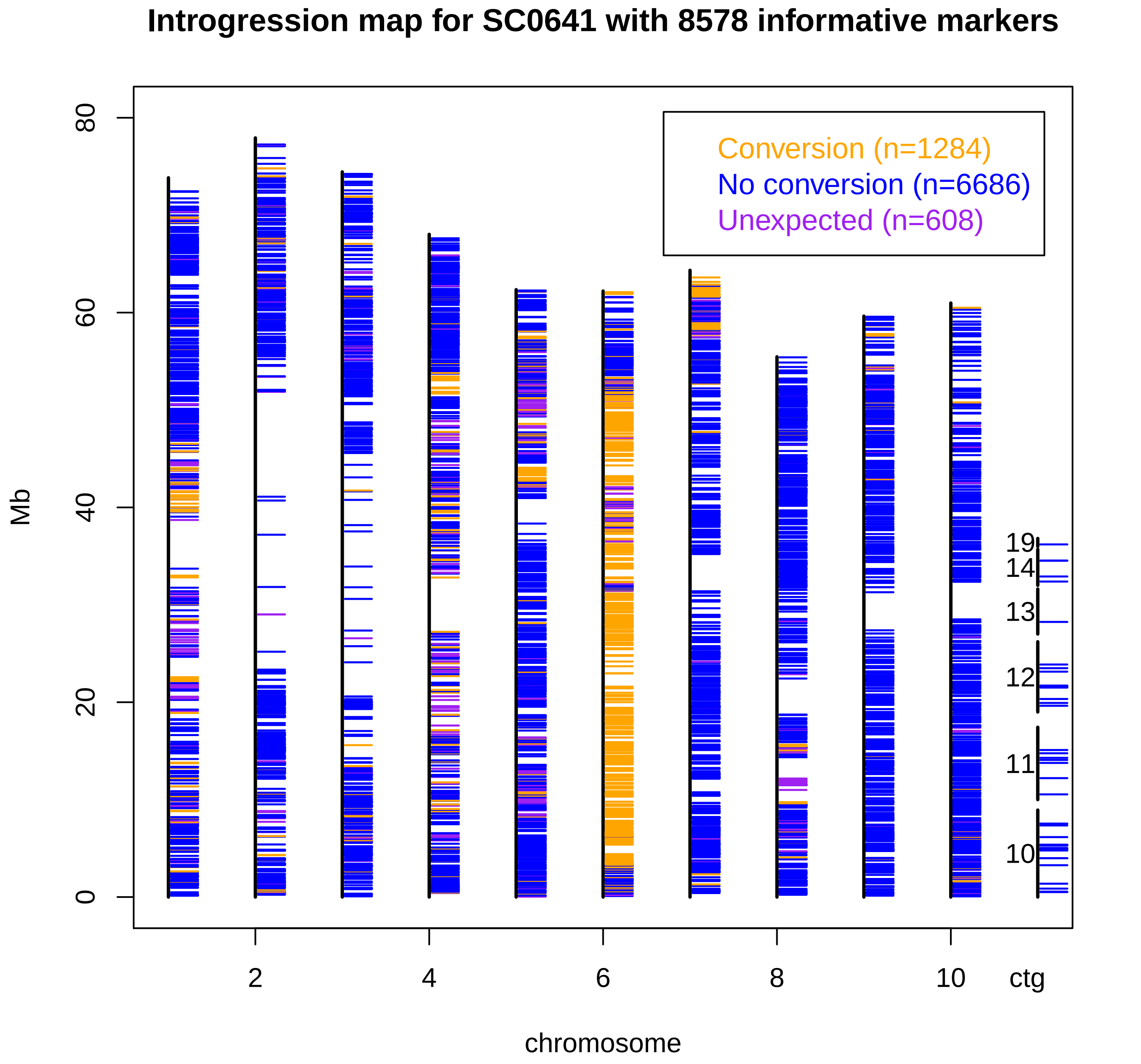
Introgression map for SC0641 with 8578 informative markers
Conversion (n=1284)
No conversion (n=6686)
Unexpected (n=608)
Mb
0
20
40
60
80
chromosome
2
4
6
8
10
ctg
19
14
13
12
11
10

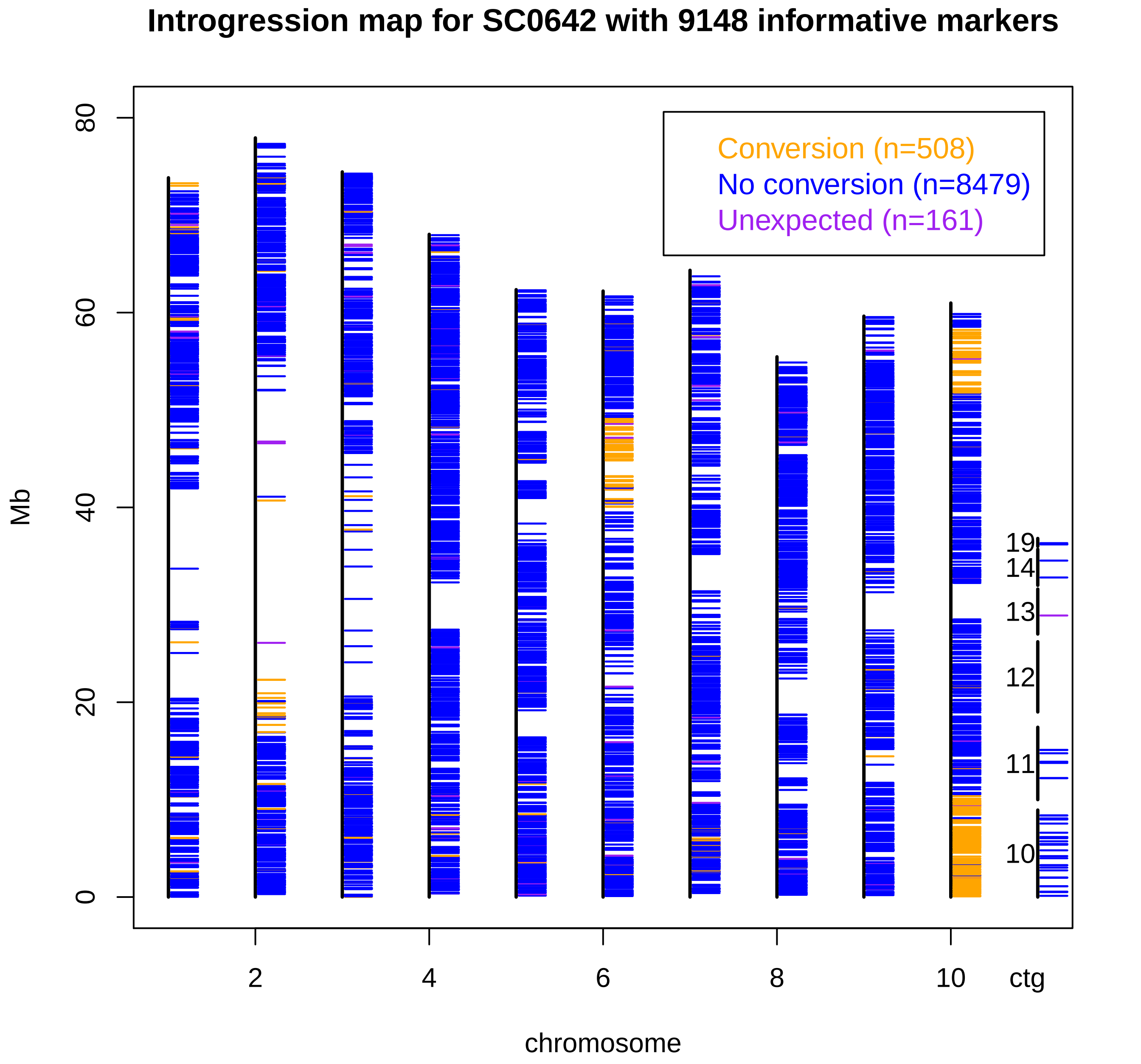
Introgression map for SC0642 with 9148 informative markers
Conversion (n=508)
No conversion (n=8479)
Unexpected (n=161)
Mb
0
20
40
60
80
chromosome
2
4
6
8
10
ctg
19
14
13
12
11
10

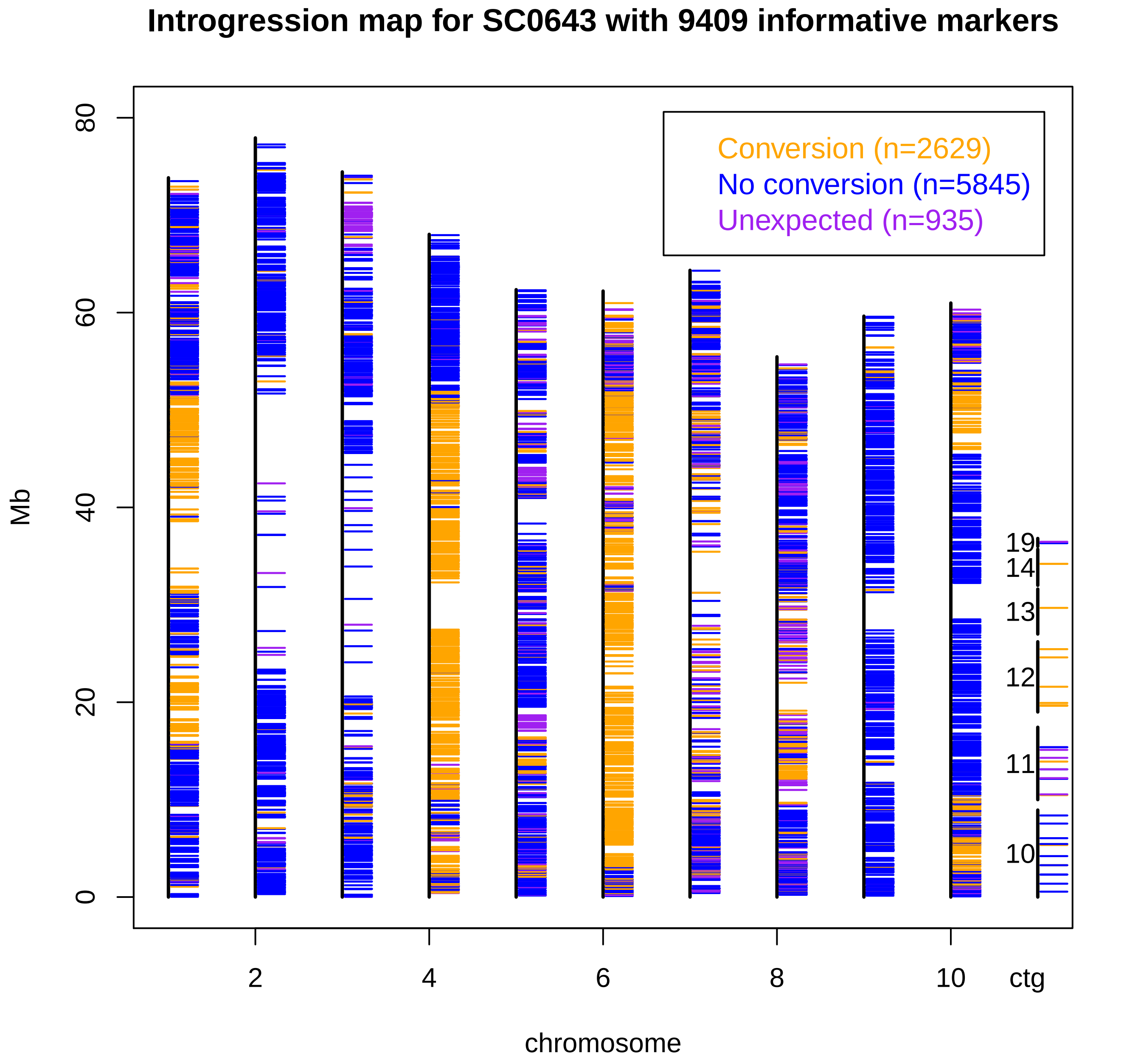

Introgression map for SC0643 with 9409 informative markers
Conversion (n=2629)
No conversion (n=5845)
Unexpected (n=935)
Mb
0
20
40
60
80
chromosome
2
4
6
8
10
ctg
19
14
13
12
11
10

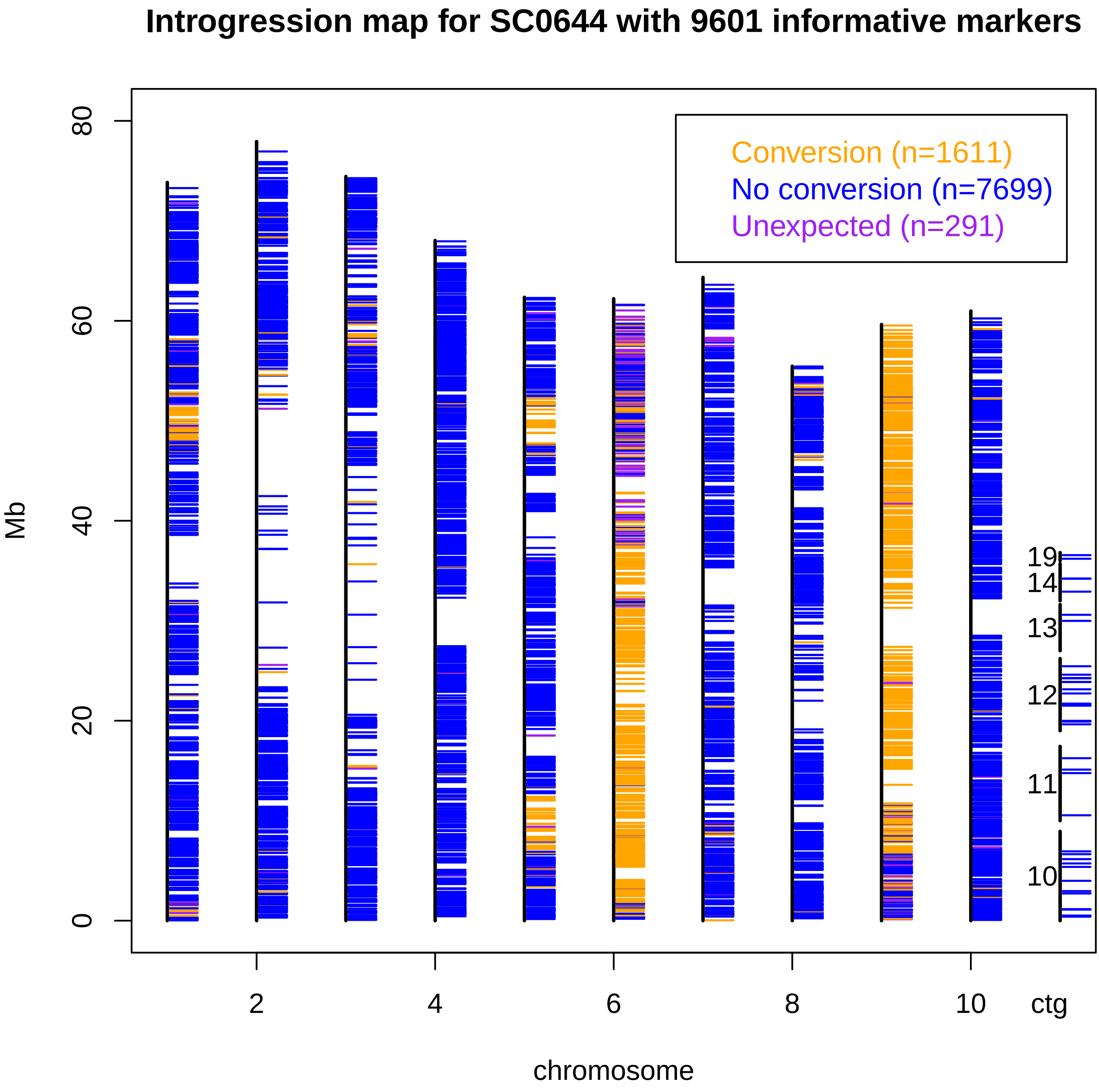

Introgression map for SC0644 with 9601 informative markers
Conversion (n=1611)
No conversion (n=7699)
Unexpected (n=291)
Mb
0
20
40
60
80
chromosome
2
4
6
8
10
ctg
19
14
13
12
11
10

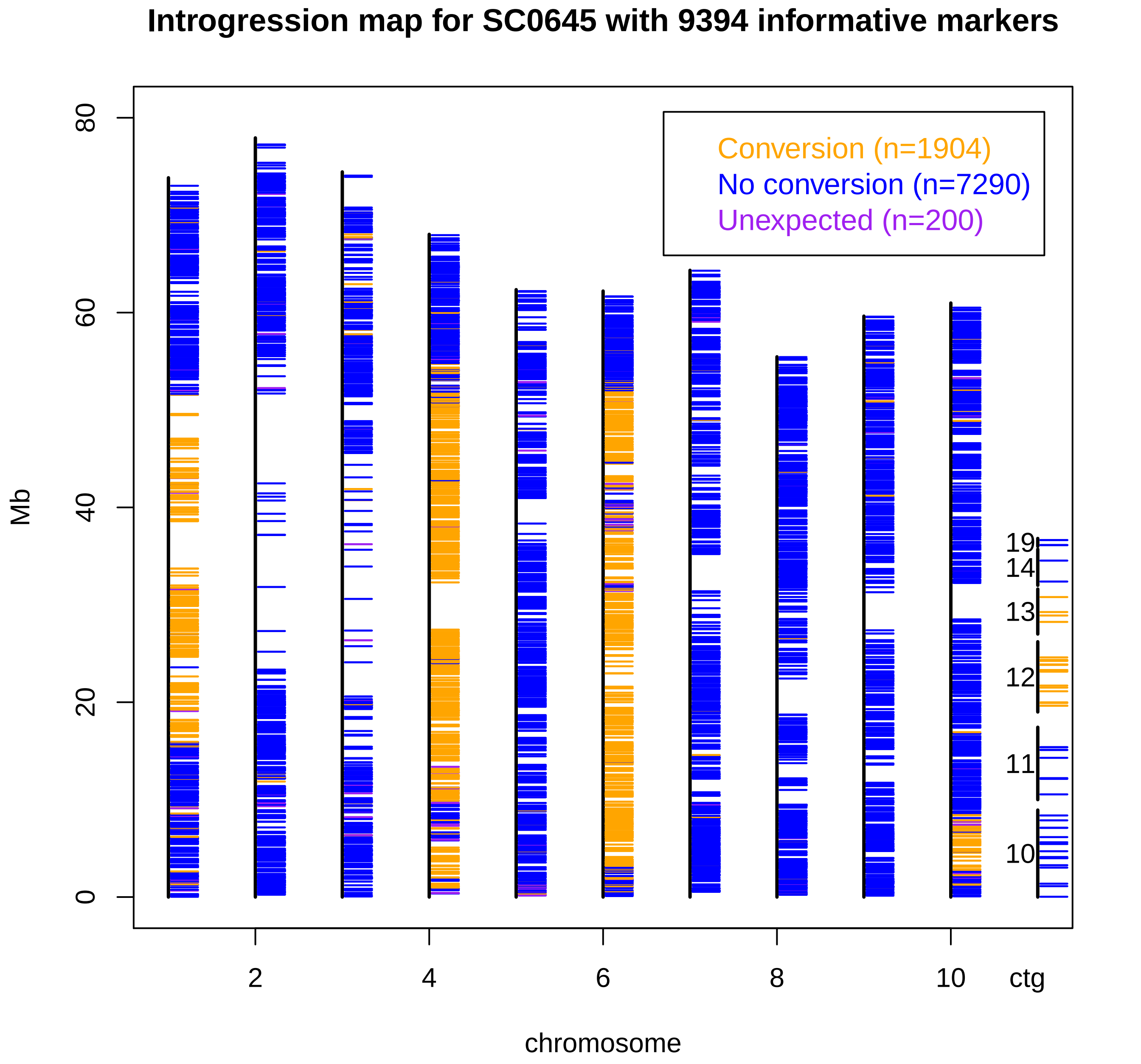

Introgression map for SC0645 with 9394 informative markers
Conversion (n=1904)
No conversion (n=7290)
Unexpected (n=200)
Mb
0
20
40
60
80
chromosome
2
4
6
8
10
ctg
19
14
13
12
11
10

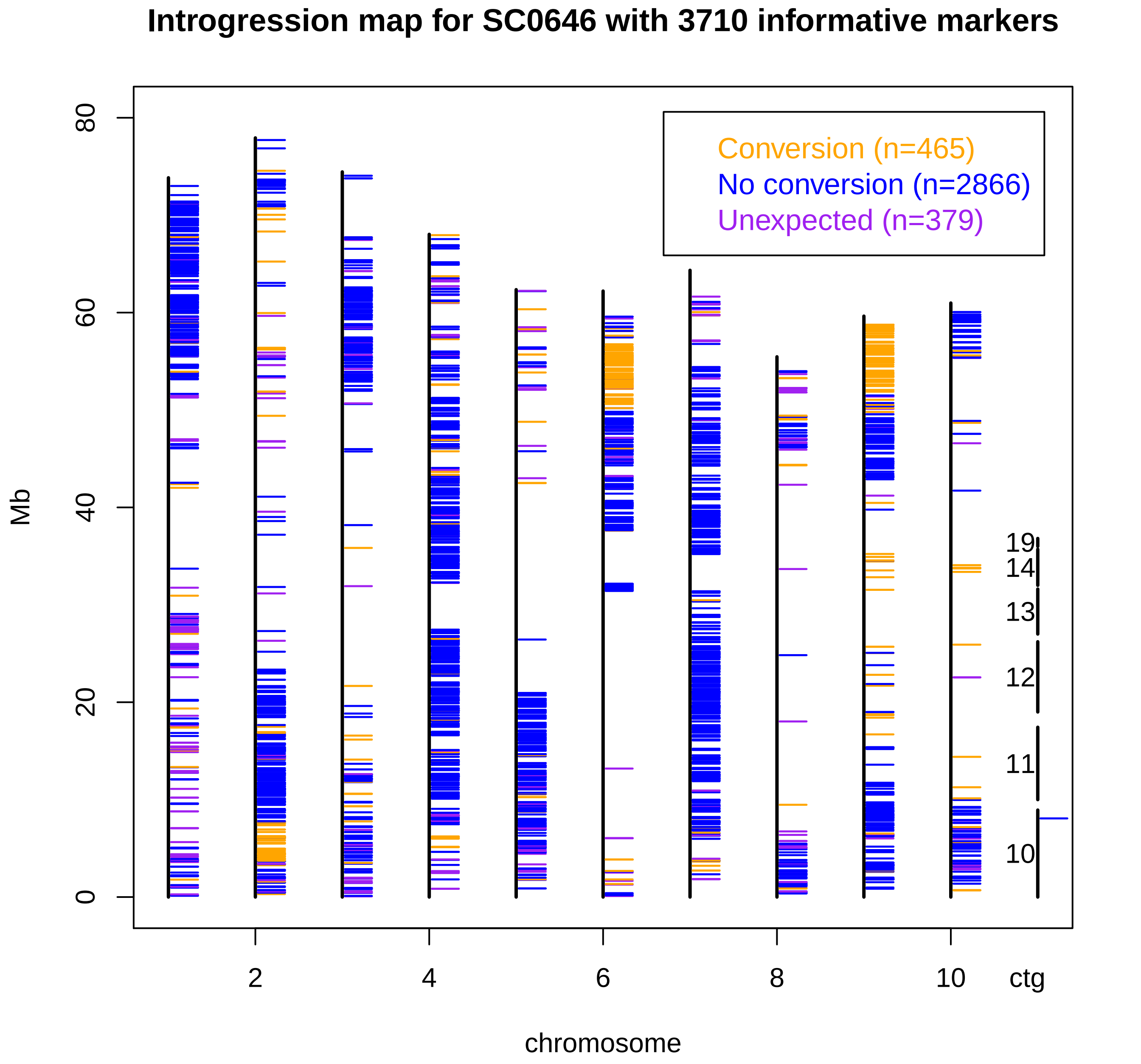
Introgression map for SC0646 with 3710 informative markers
Conversion (n=465)
No conversion (n=2866)
Unexpected (n=379)
Mb
0
20
40
60
80
2
4
6
8
10
ctg
chromosome
19
14
13
12
11
10

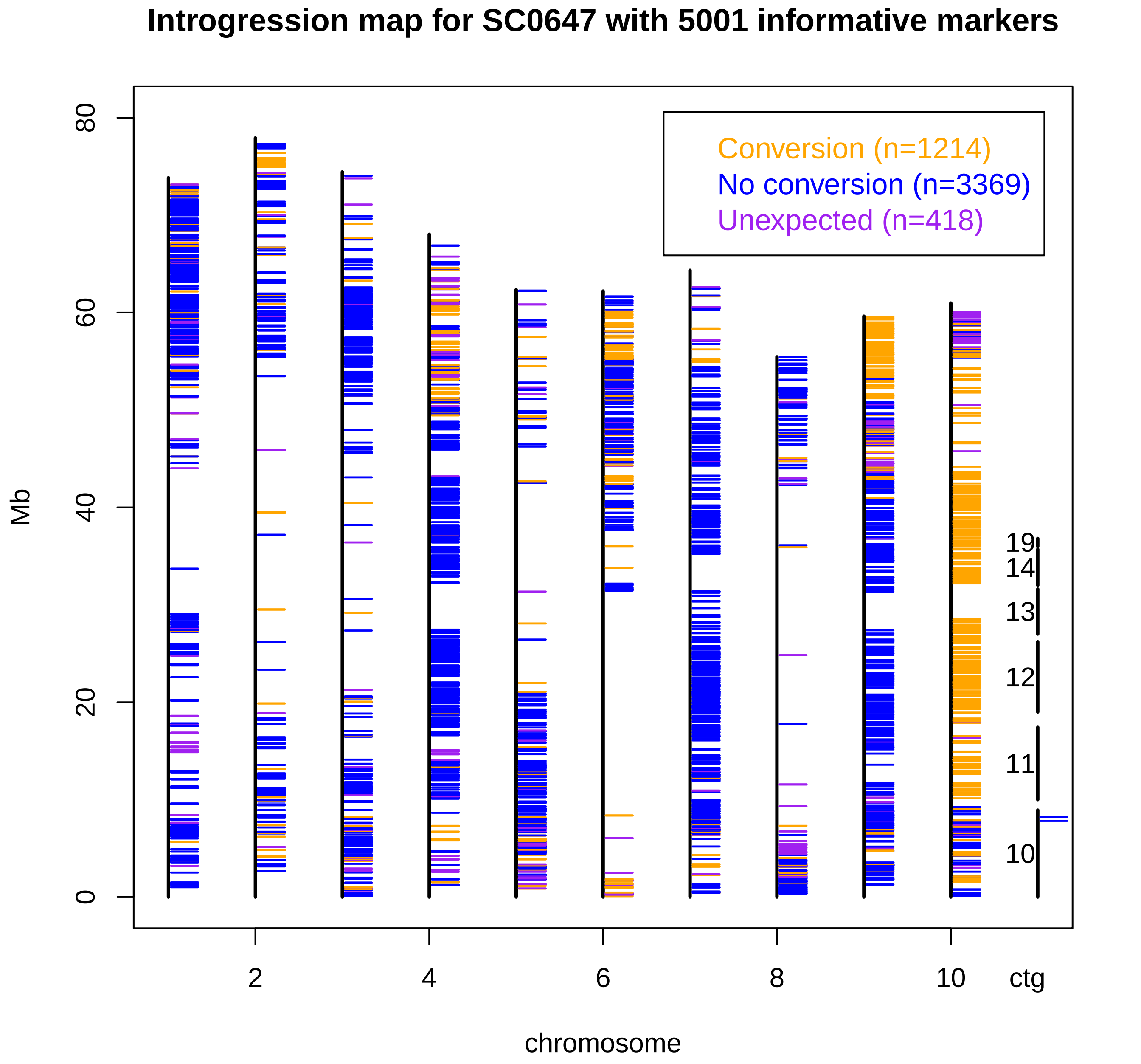

Introgression map for SC0647 with 5001 informative markers
Conversion (n=1214)
No conversion (n=3369)
Unexpected (n=418)
Mb
0
20
40
60
80
2
4
6
8
10
ctg
chromosome
19
14
13
12
11
10

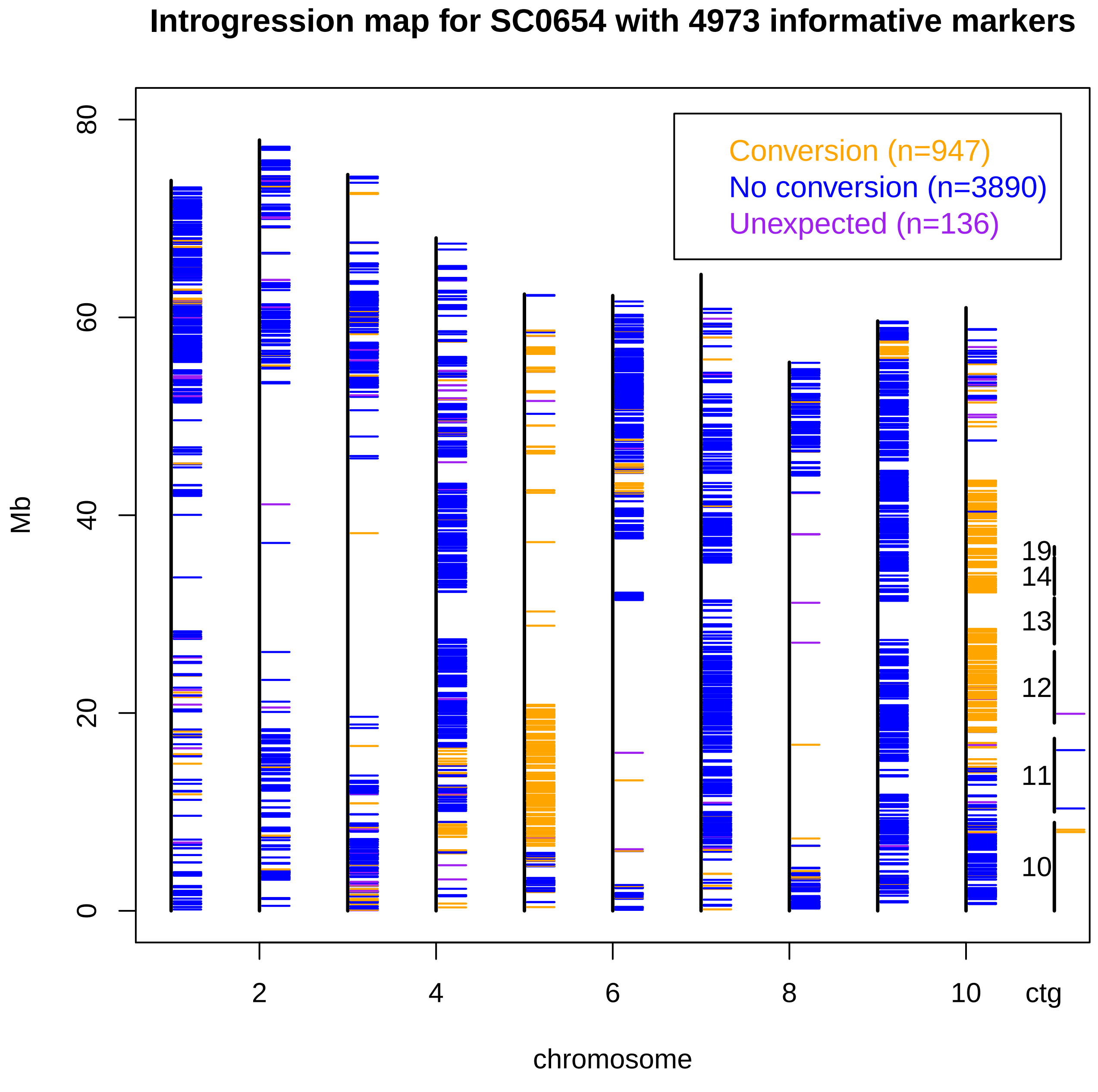

Introgression map for SC0654 with 4973 informative markers
Conversion (n=947)
No conversion (n=3890)
Unexpected (n=136)
Mb
0
20
40
60
80
2
4
6
8
10
ctg
chromosome
19
14
13
12
11
10

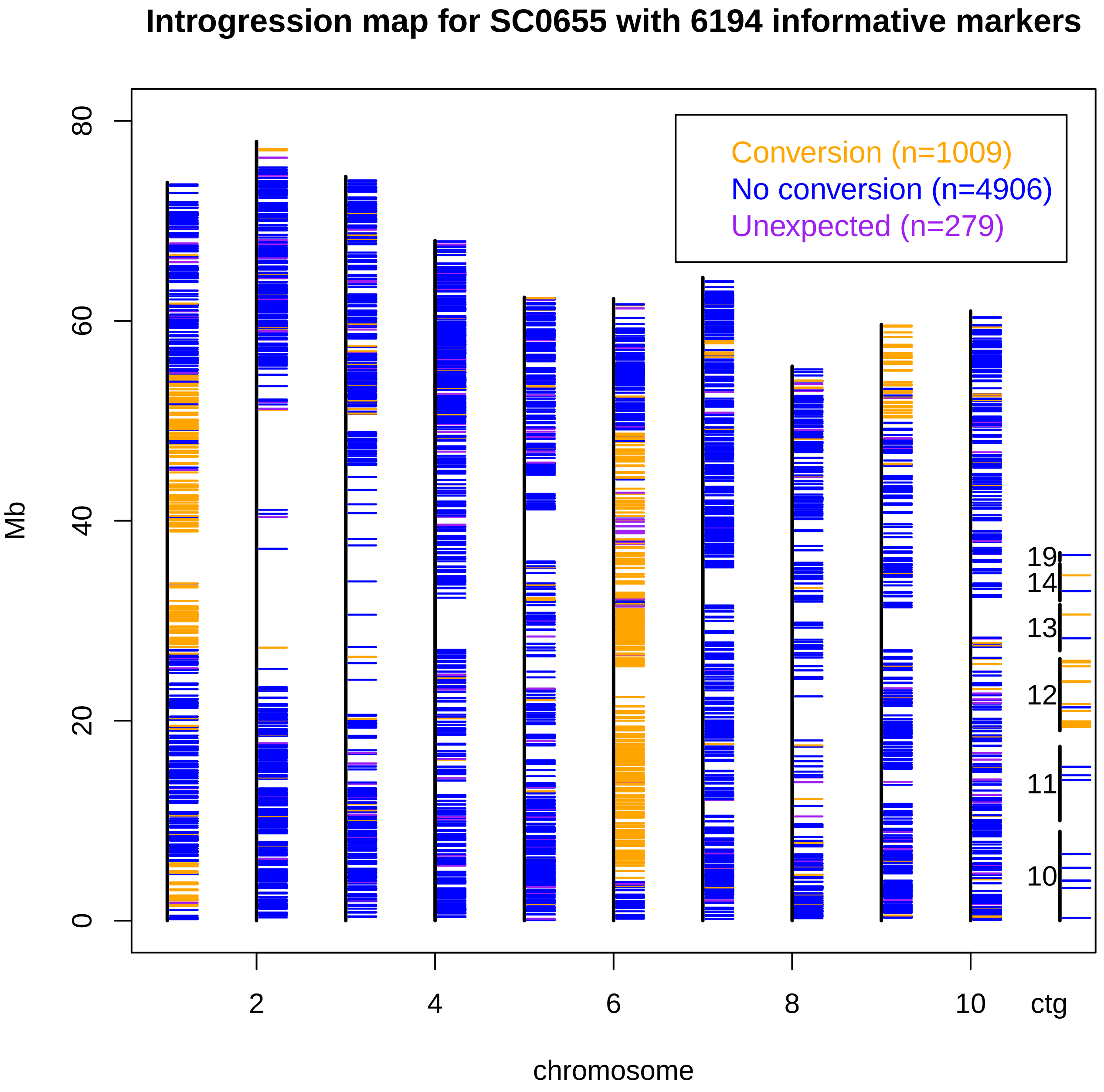

Introgression map for SC0655 with 6194 informative markers
Conversion (n=1009)
No conversion (n=4906)
Unexpected (n=279)
Mb
0
20
40
60
80
chromosome
2
4
6
8
10
ctg
19
14
13
12
11
10

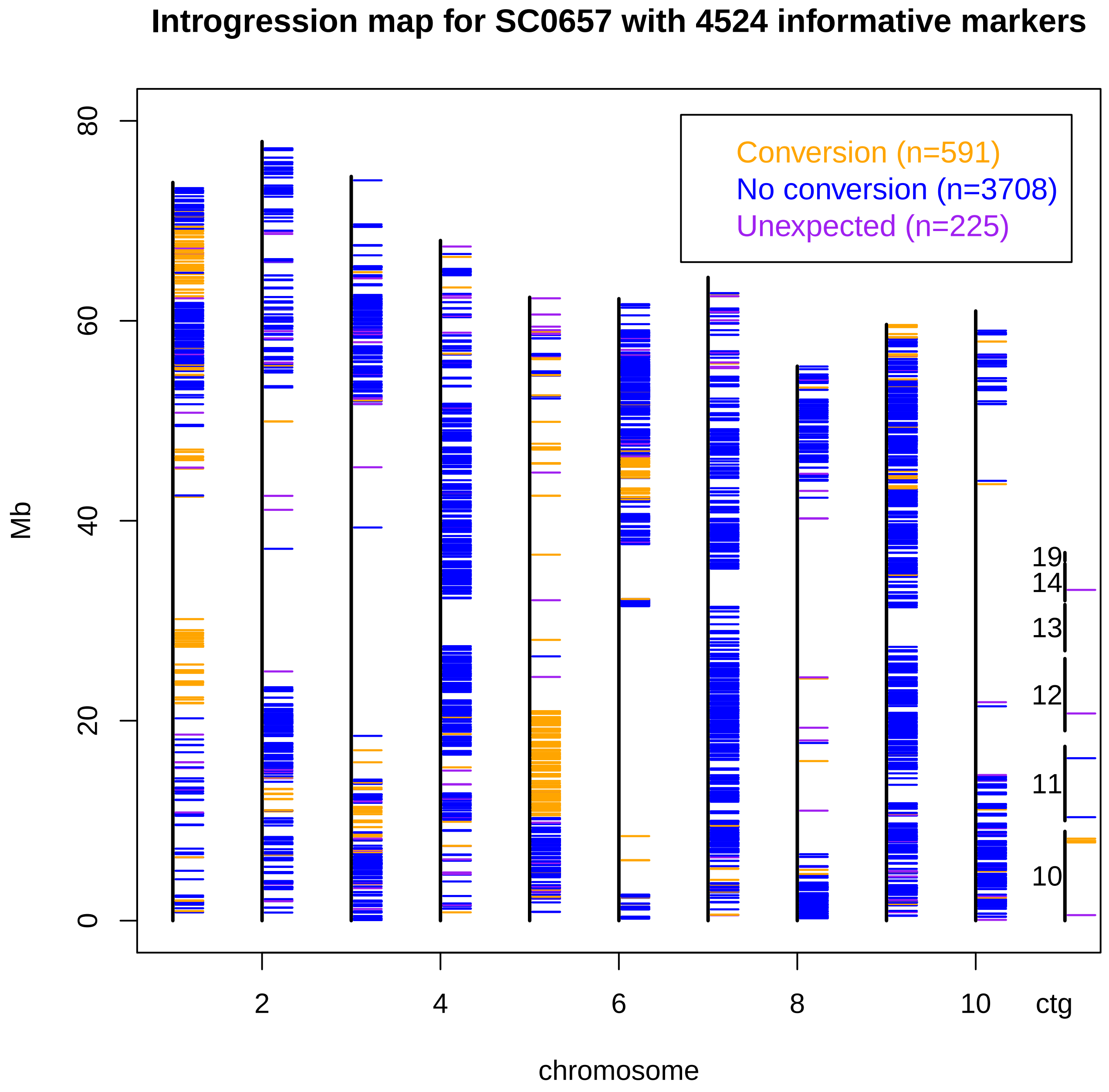

Introgression map for SC0657 with 4524 informative markers
Conversion (n=591)
No conversion (n=3708)
Unexpected (n=225)
Mb
0
20
40
60
80
chromosome
2
4
6
8
10
ctg
19
14
13
12
11
10

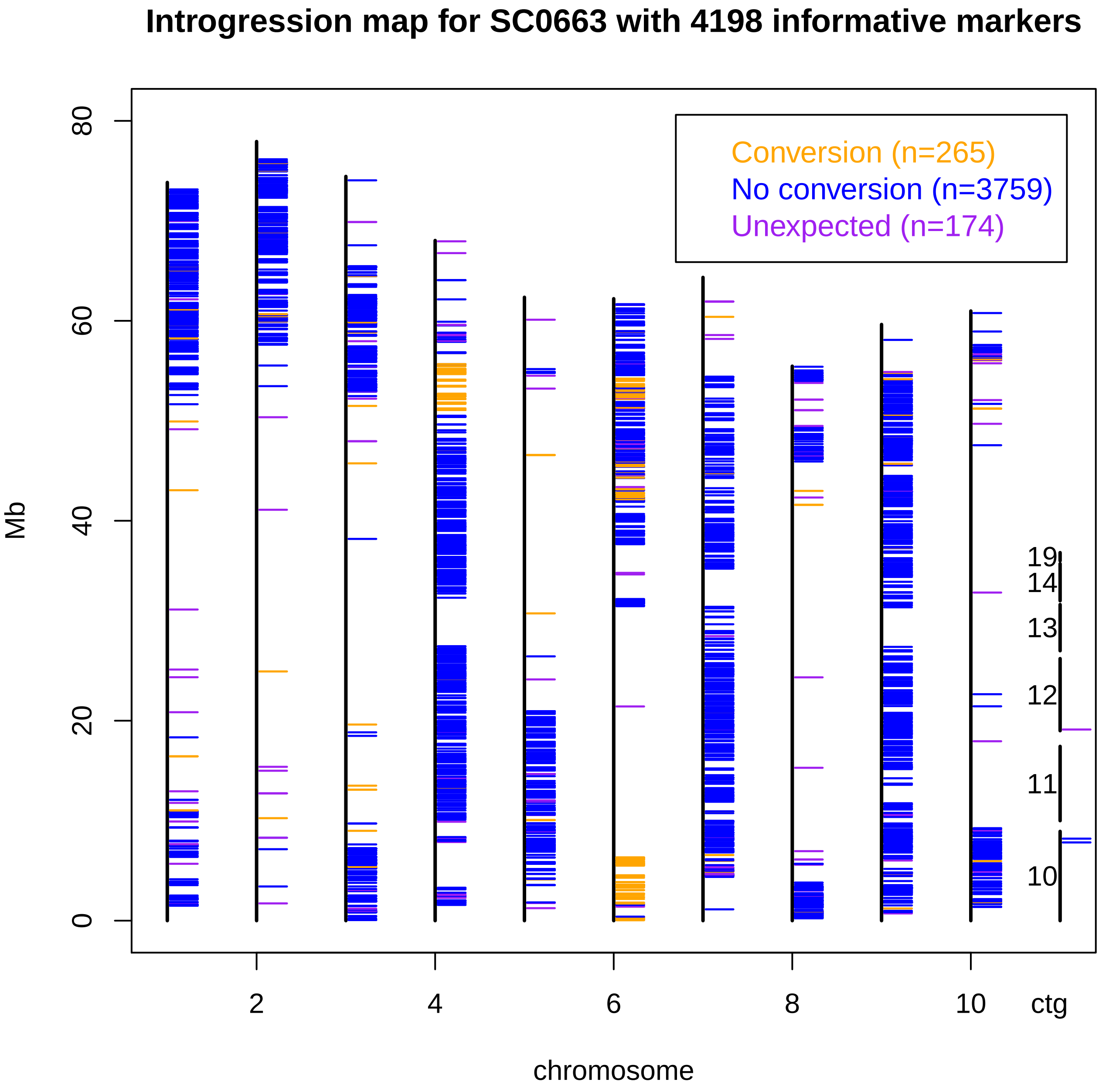

Introgression map for SC0663 with 4198 informative markers
Conversion (n=265)
No conversion (n=3759)
Unexpected (n=174)
Mb
0
20
40
60
80
chromosome
2
4
6
8
10
ctg
19
14
13
12
11
10

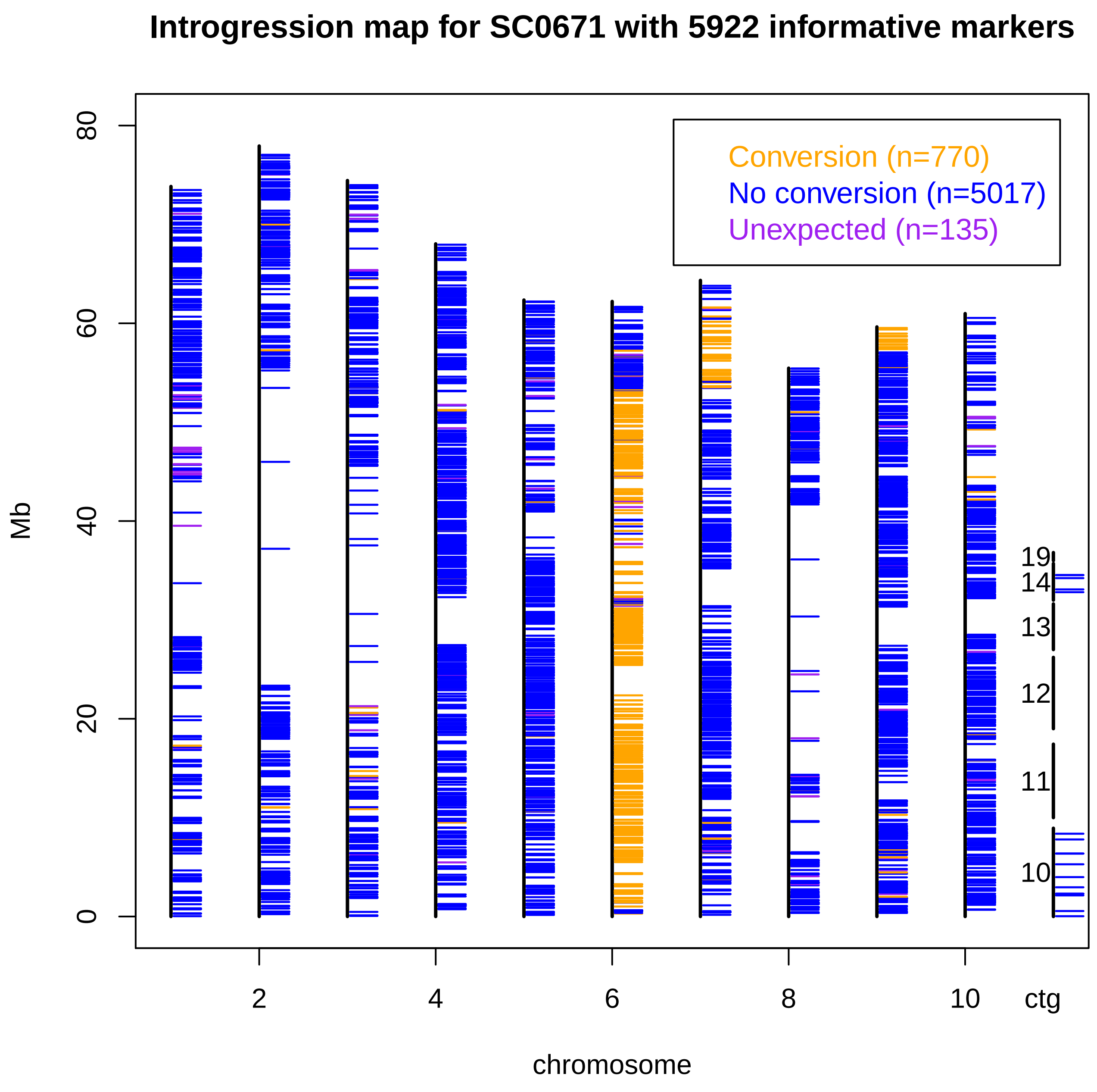
Introgression map for SC0671 with 5922 informative markers
Conversion (n=770)
No conversion (n=5017)
Unexpected (n=135)
Mb
0
20
40
60
80
2
4
6
8
10
ctg
19
14
13
12
11
10
chromosome

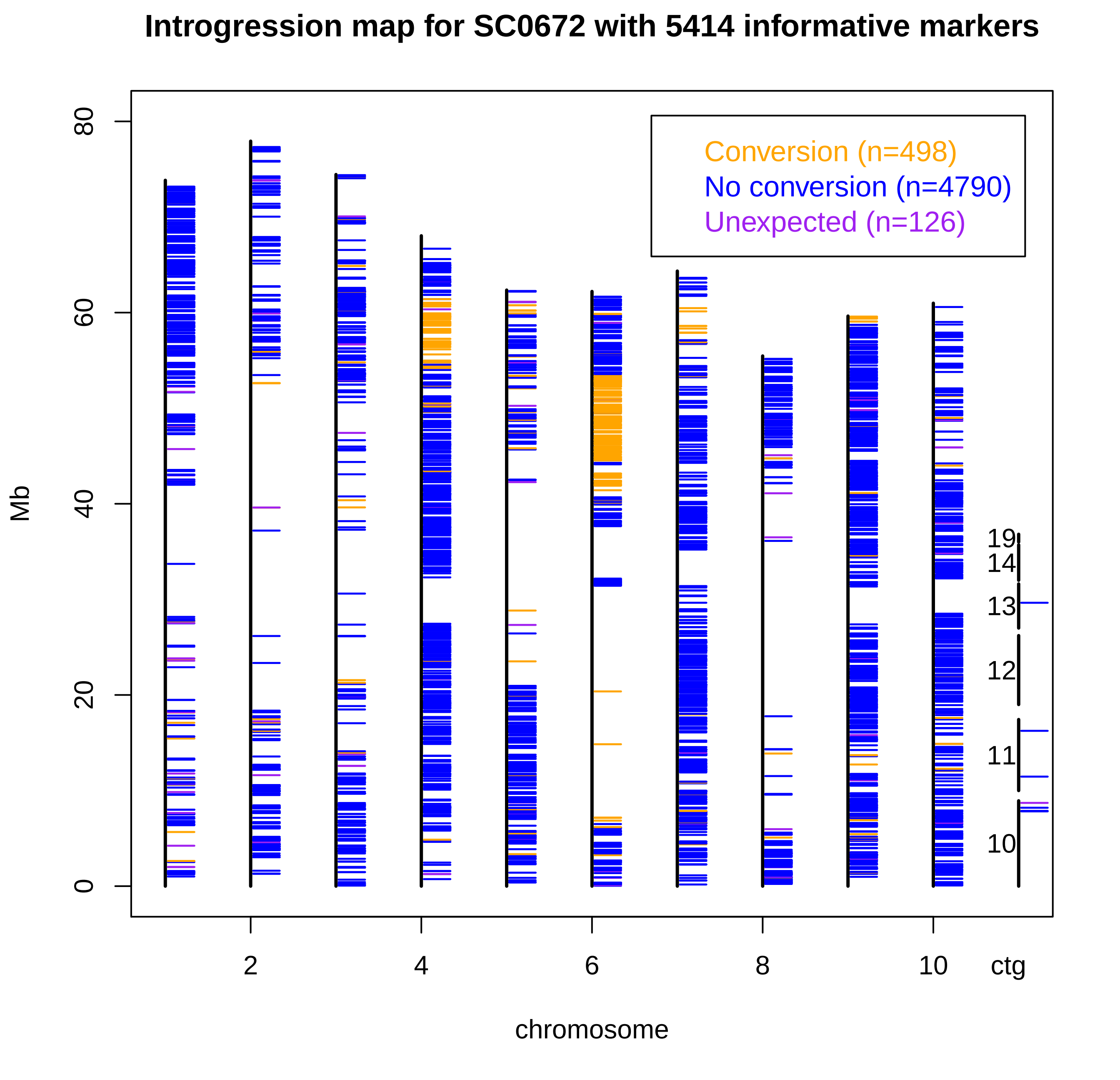
Introgression map for SC0672 with 5414 informative markers
Conversion (n=498)
No conversion (n=4790)
Unexpected (n=126)
Mb
0
20
40
60
80
chromosome
2
4
6
8
10
ctg
19
14
13
12
11
10

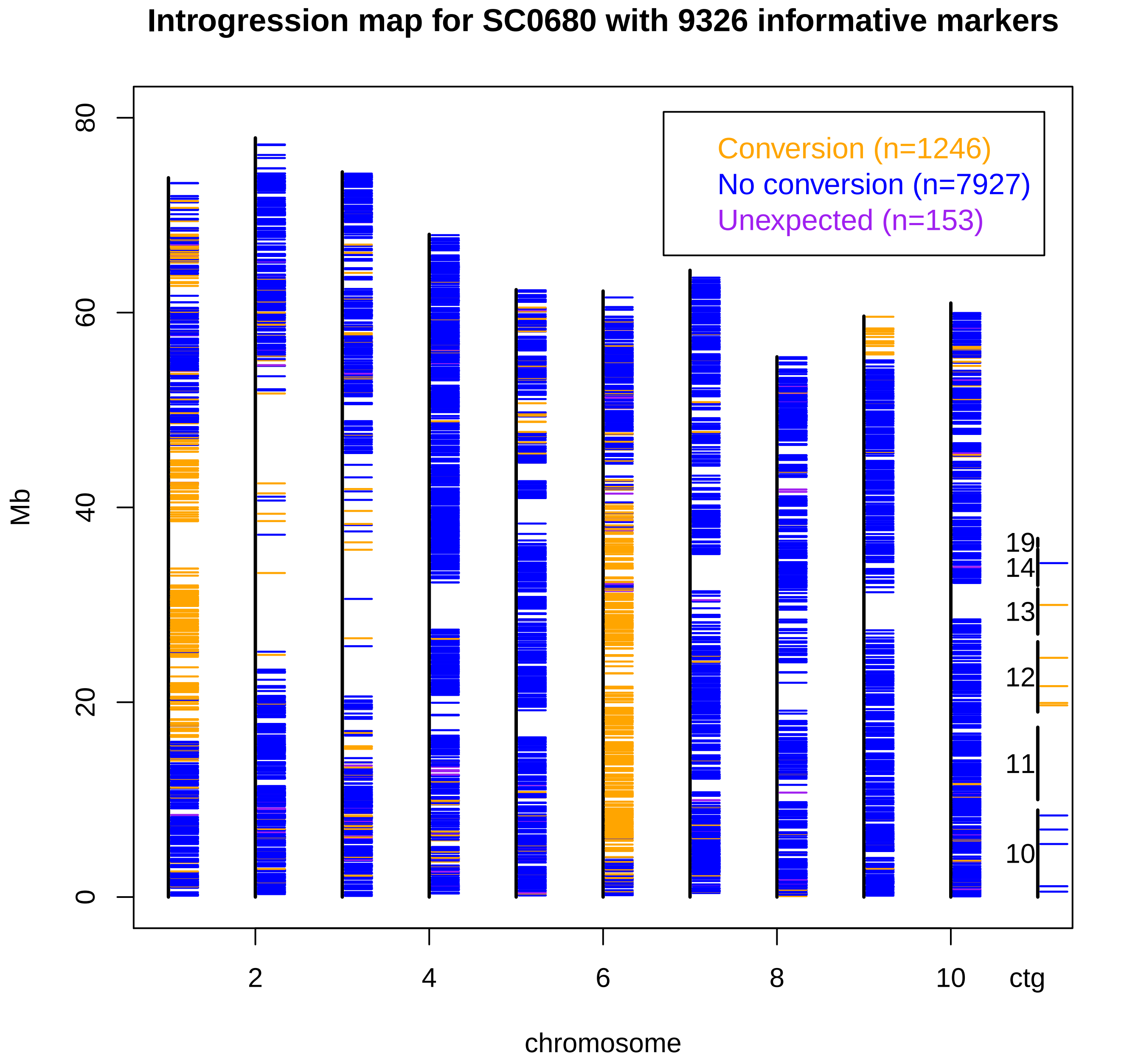

Introgression map for SC0680 with 9326 informative markers
Conversion (n=1246)
No conversion (n=7927)
Unexpected (n=153)
Mb
0
20
40
60
80
chromosome
2
4
6
8
10
ctg
19
14
13
12
11
10

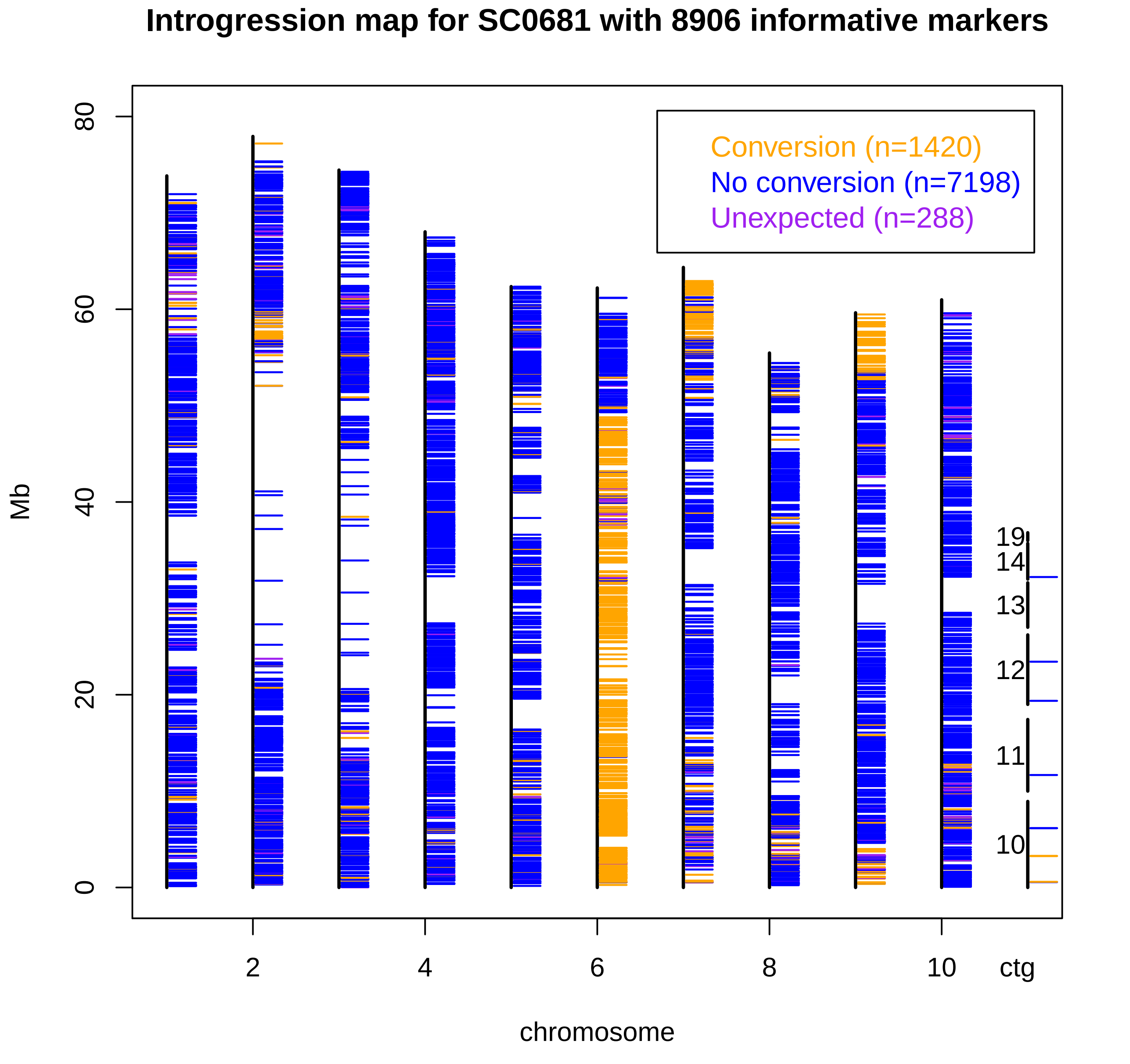

Introgression map for SC0681 with 8906 informative markers
Conversion (n=1420)
No conversion (n=7198)
Unexpected (n=288)
Mb
0
20
40
60
80
2
4
6
8
10
ctg
19
14
13
12
11
10
chromosome

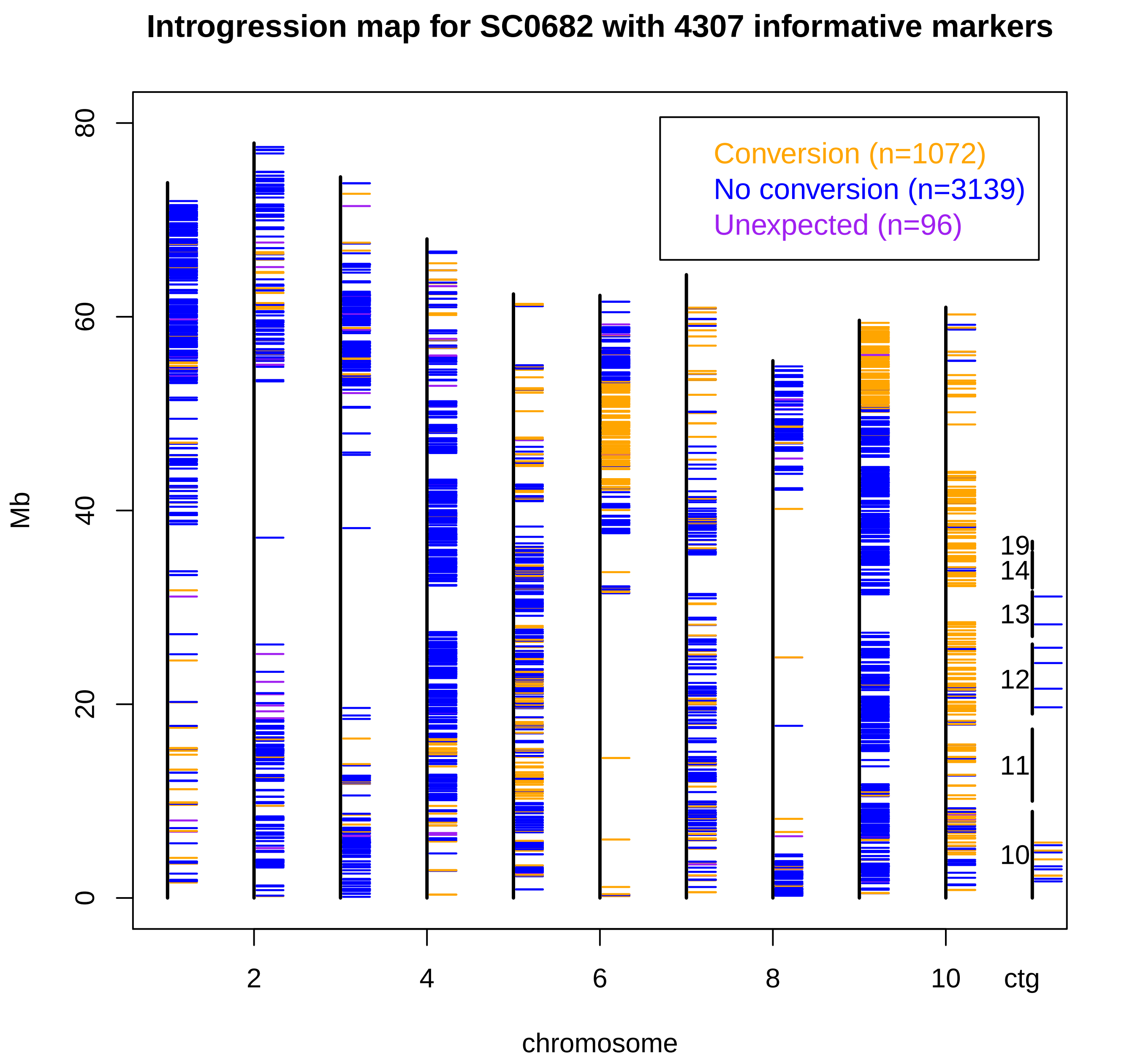

Introgression map for SC0682 with 4307 informative markers
Conversion (n=1072)
No conversion (n=3139)
Unexpected (n=96)
Mb
0
20
40
60
80
chromosome
2
4
6
8
10
ctg
19
14
13
12
11
10

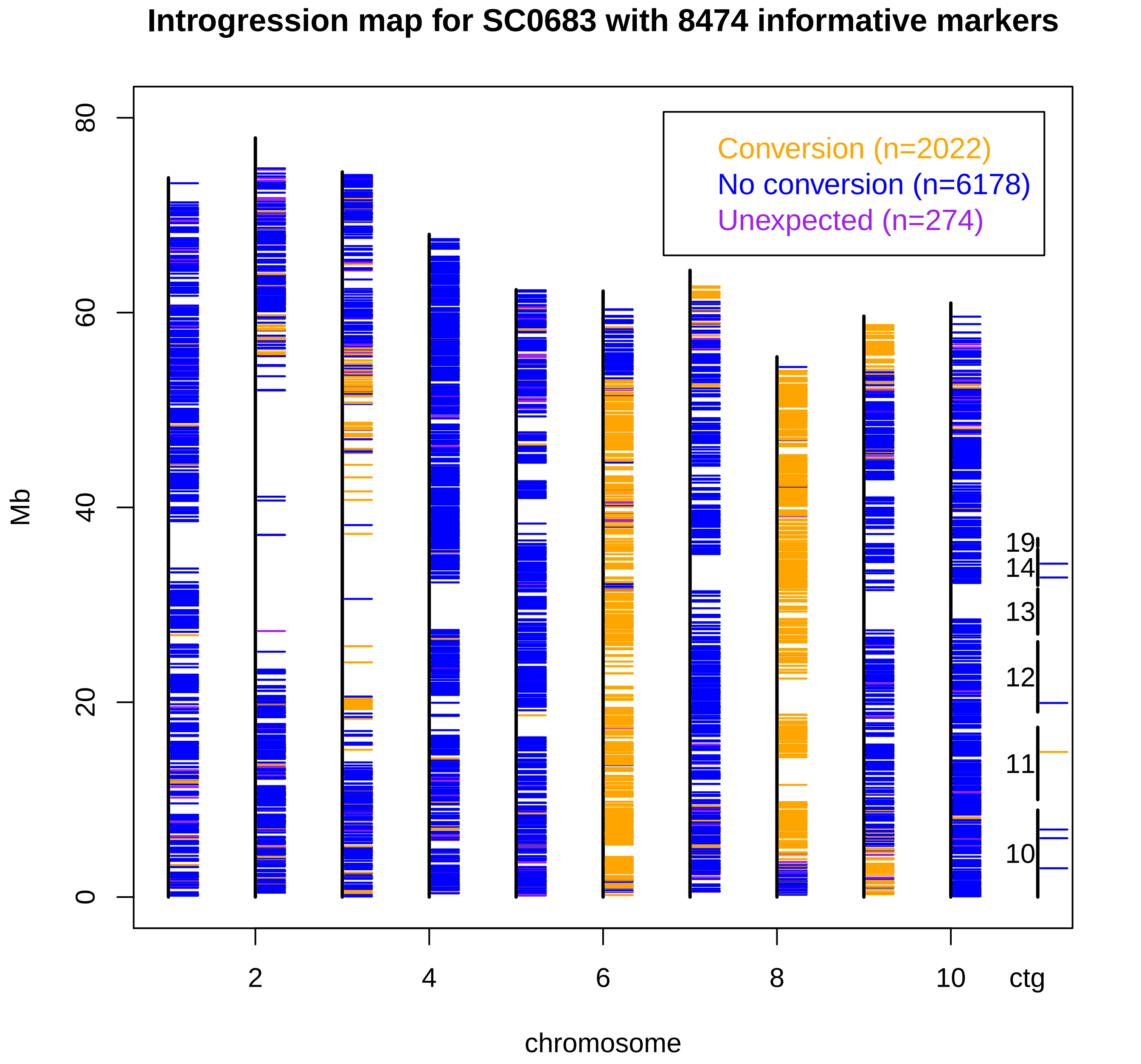
Introgression map for SC0683 with 8474 informative markers
Conversion (n=2022)
No conversion (n=6178)
Unexpected (n=274)
Mb
0
20
40
60
80
chromosome
2
4
6
8
10
ctg
19
14
13
12
11
10

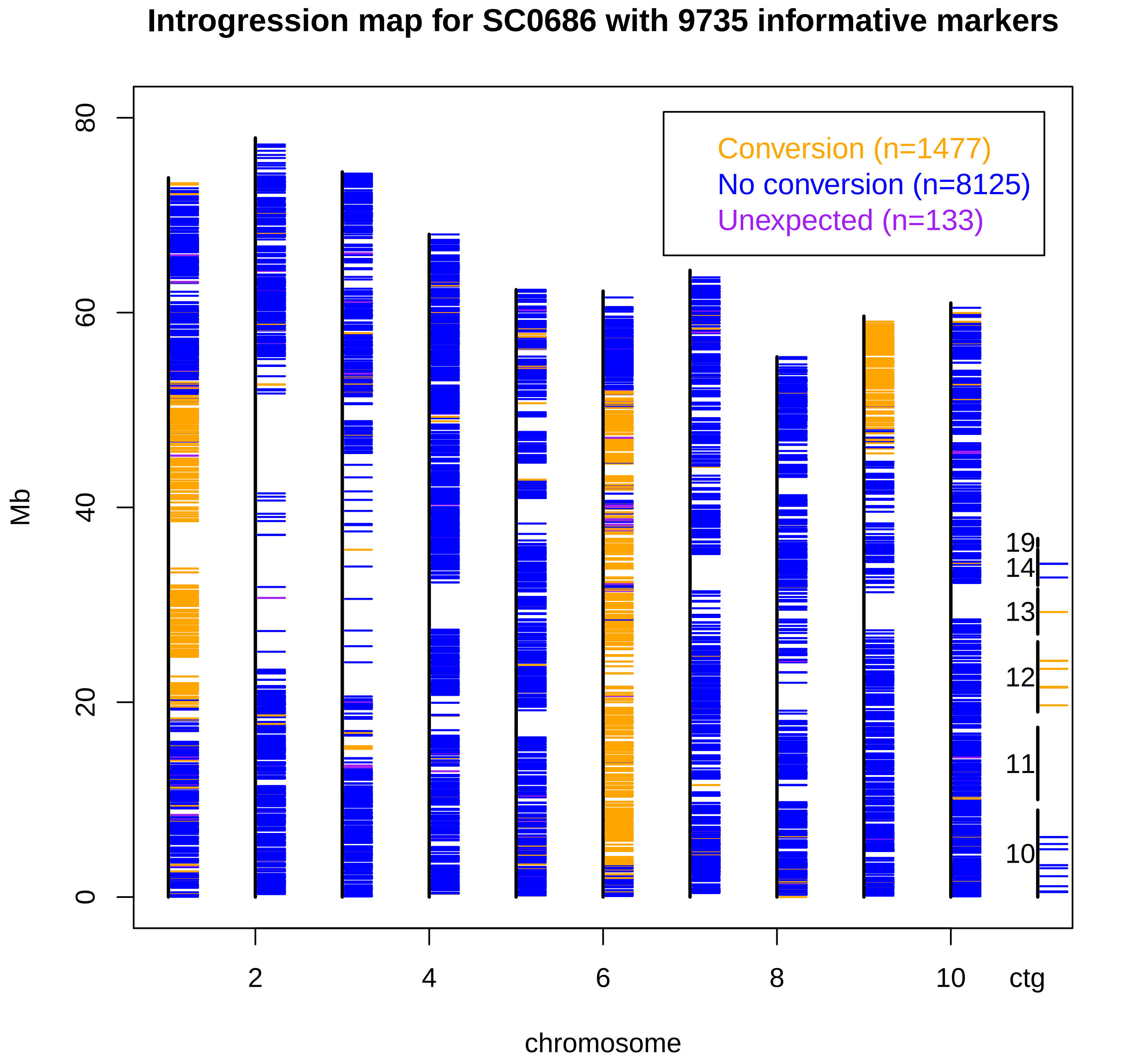
Introgression map for SC0686 with 9735 informative markers
Conversion (n=1477)
No conversion (n=8125)
Unexpected (n=133)
Mb
0
20
40
60
80
chromosome
2
4
6
8
10
ctg
19
14
13
12
11
10

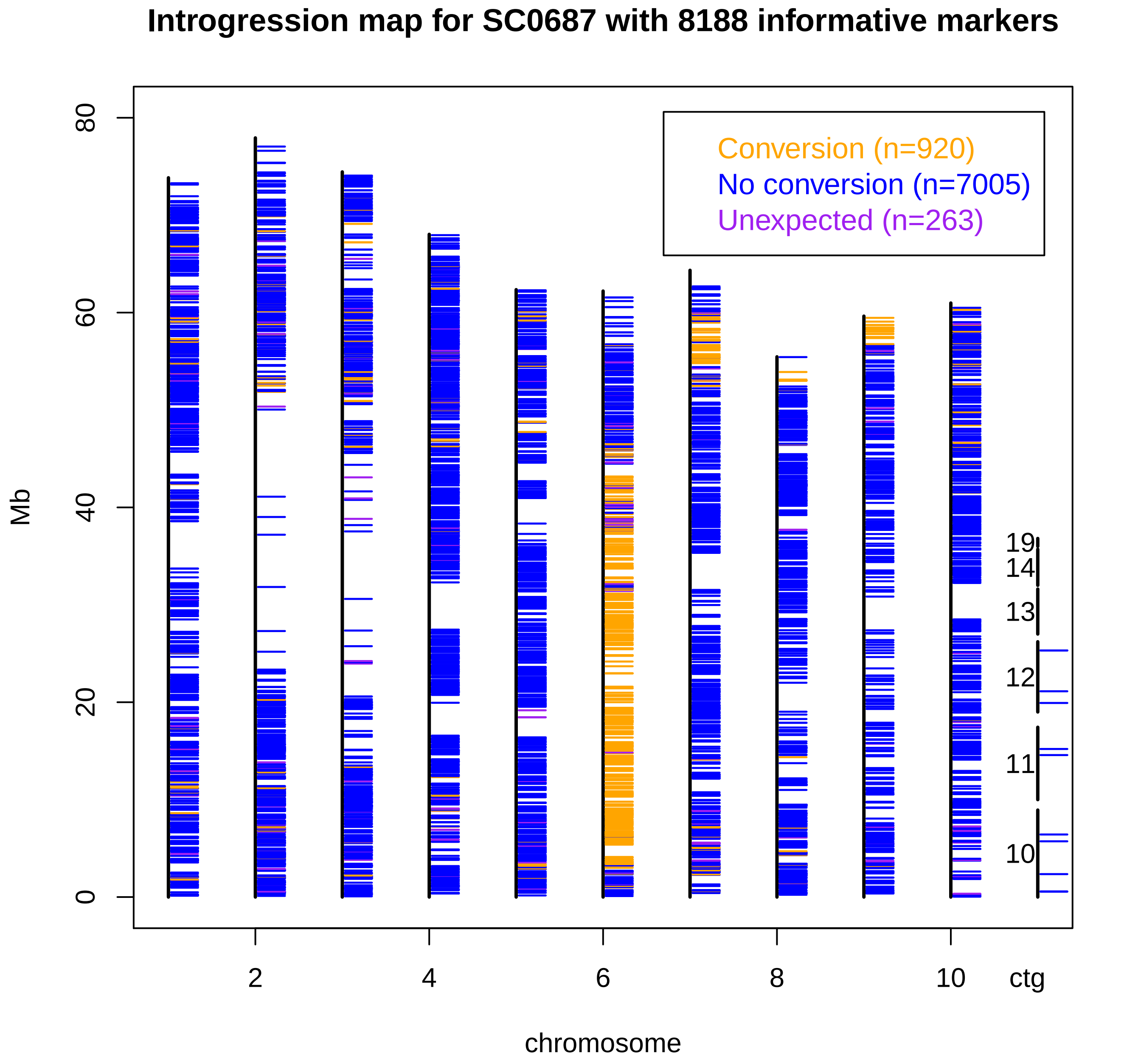
Introgression map for SC0687 with 8188 informative markers
Conversion (n=920)
No conversion (n=7005)
Unexpected (n=263)
Mb
0
20
40
60
80
2
4
6
8
10
ctg
19
14
13
12
11
10
chromosome

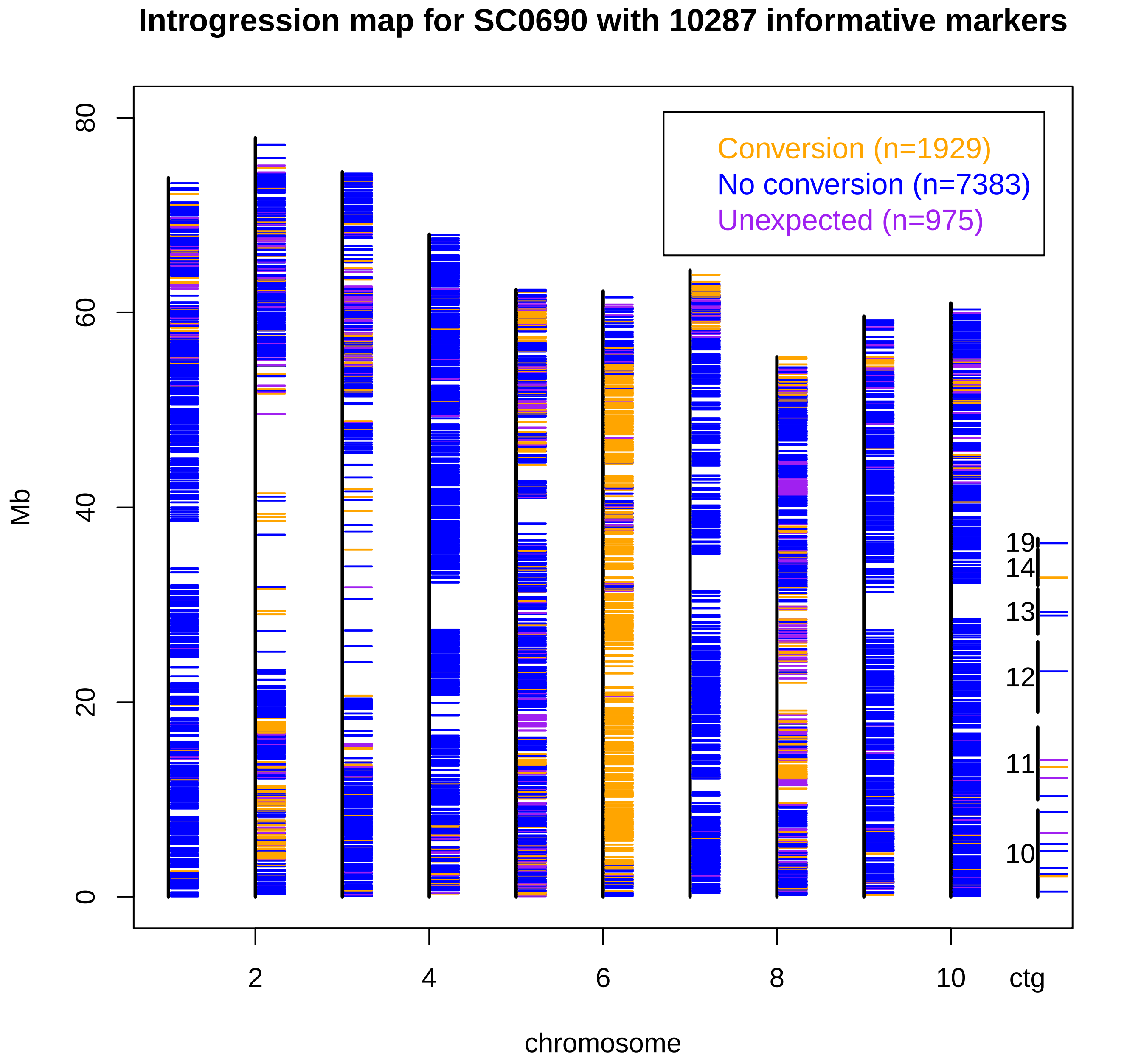

Introgression map for SC0690 with 10287 informative markers
Conversion (n=1929)
No conversion (n=7383)
Unexpected (n=975)
Mb
0
20
40
60
80
chromosome
2
4
6
8
10
ctg
19
14
13
12
11
10

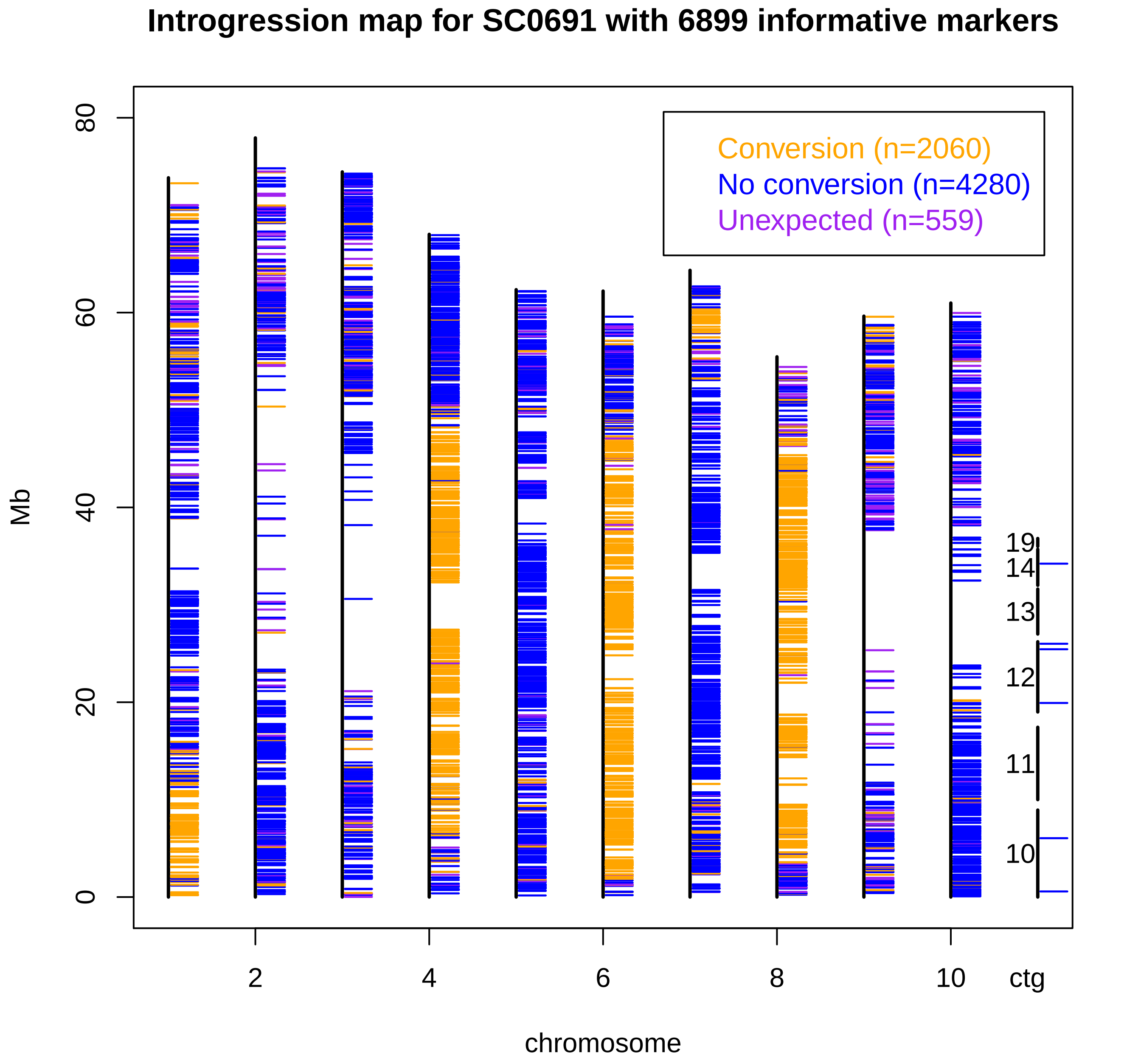

Introgression map for SC0691 with 6899 informative markers
Conversion (n=2060)
No conversion (n=4280)
Unexpected (n=559)
Mb
0
20
40
60
80
chromosome
2
4
6
8
10
ctg
19
14
13
12
11
10

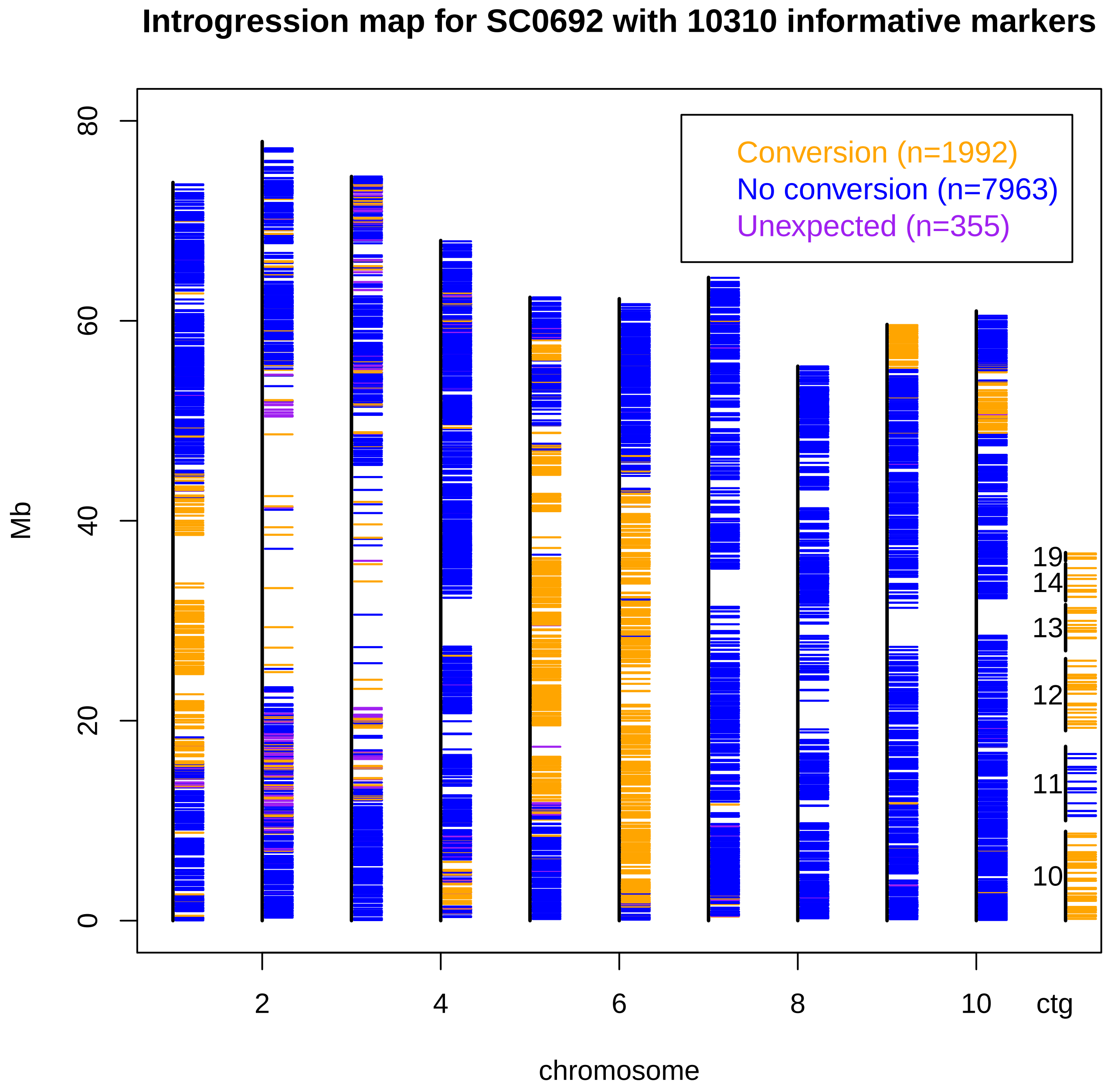

Introgression map for SC0692 with 10310 informative markers
Conversion (n=1992)
No conversion (n=7963)
Unexpected (n=355)
Mb
0
20
40
60
80
chromosome
2
4
6
8
10
ctg
19
14
13
12
11
10

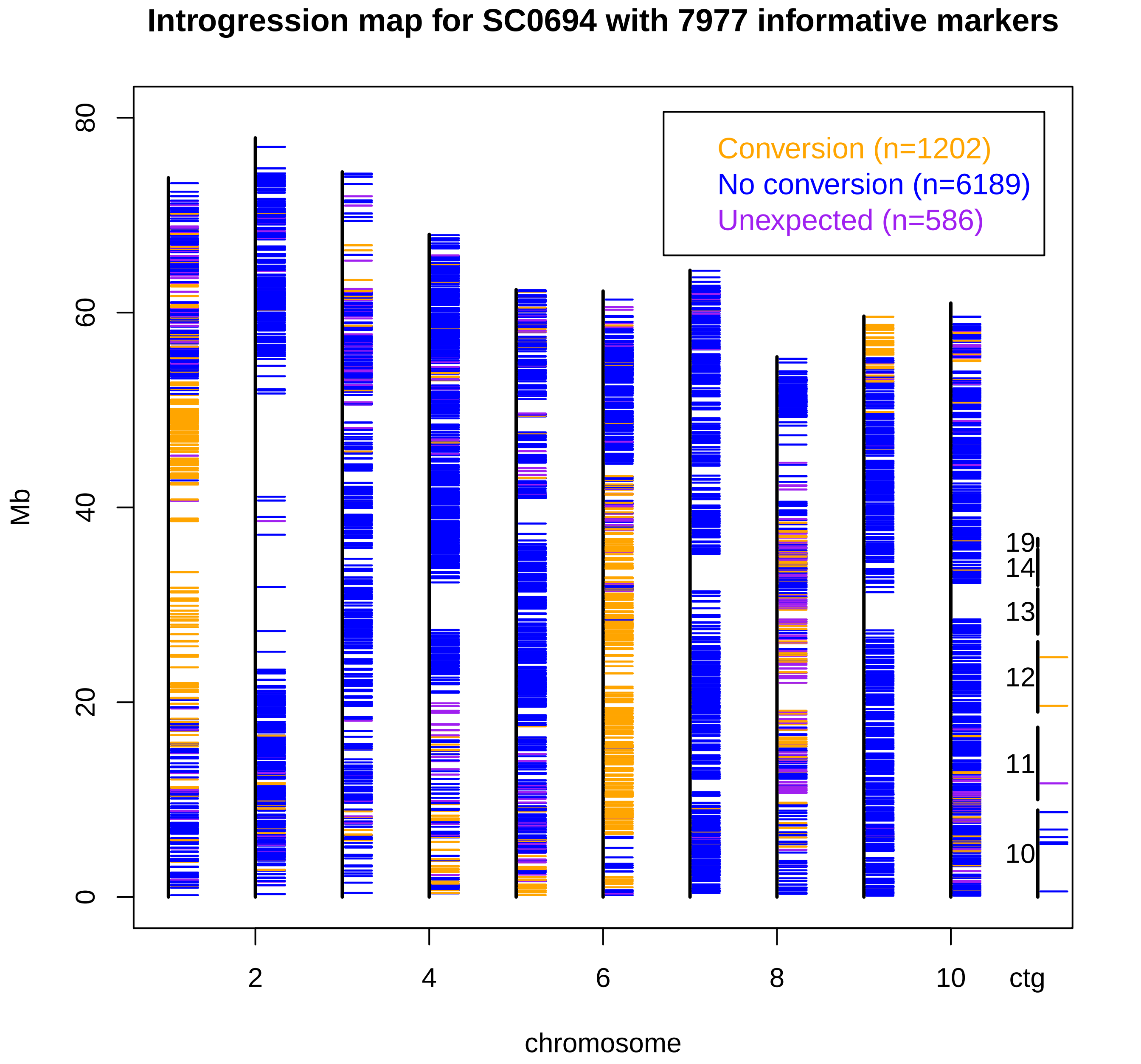
Introgression map for SC0694 with 7977 informative markers
Conversion (n=1202)
No conversion (n=6189)
Unexpected (n=586)
Mb
0
20
40
60
80
chromosome
2
4
6
8
10
ctg
19
14
13
12
11
10

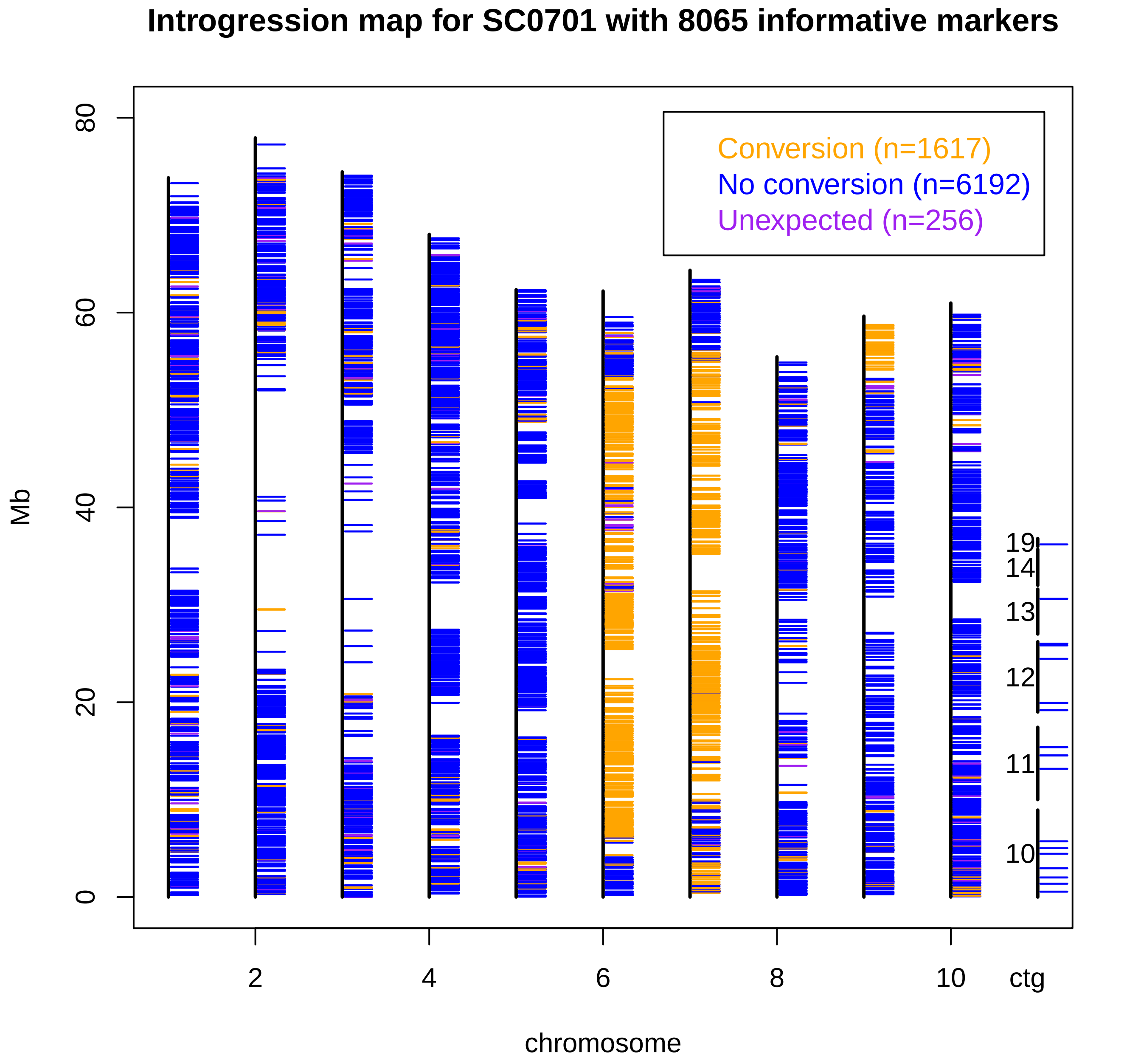

Introgression map for SC0701 with 8065 informative markers
Conversion (n=1617)
No conversion (n=6192)
Unexpected (n=256)
Mb
0
20
40
60
80
chromosome
2
4
6
8
10
ctg
19
14
13
12
11
10

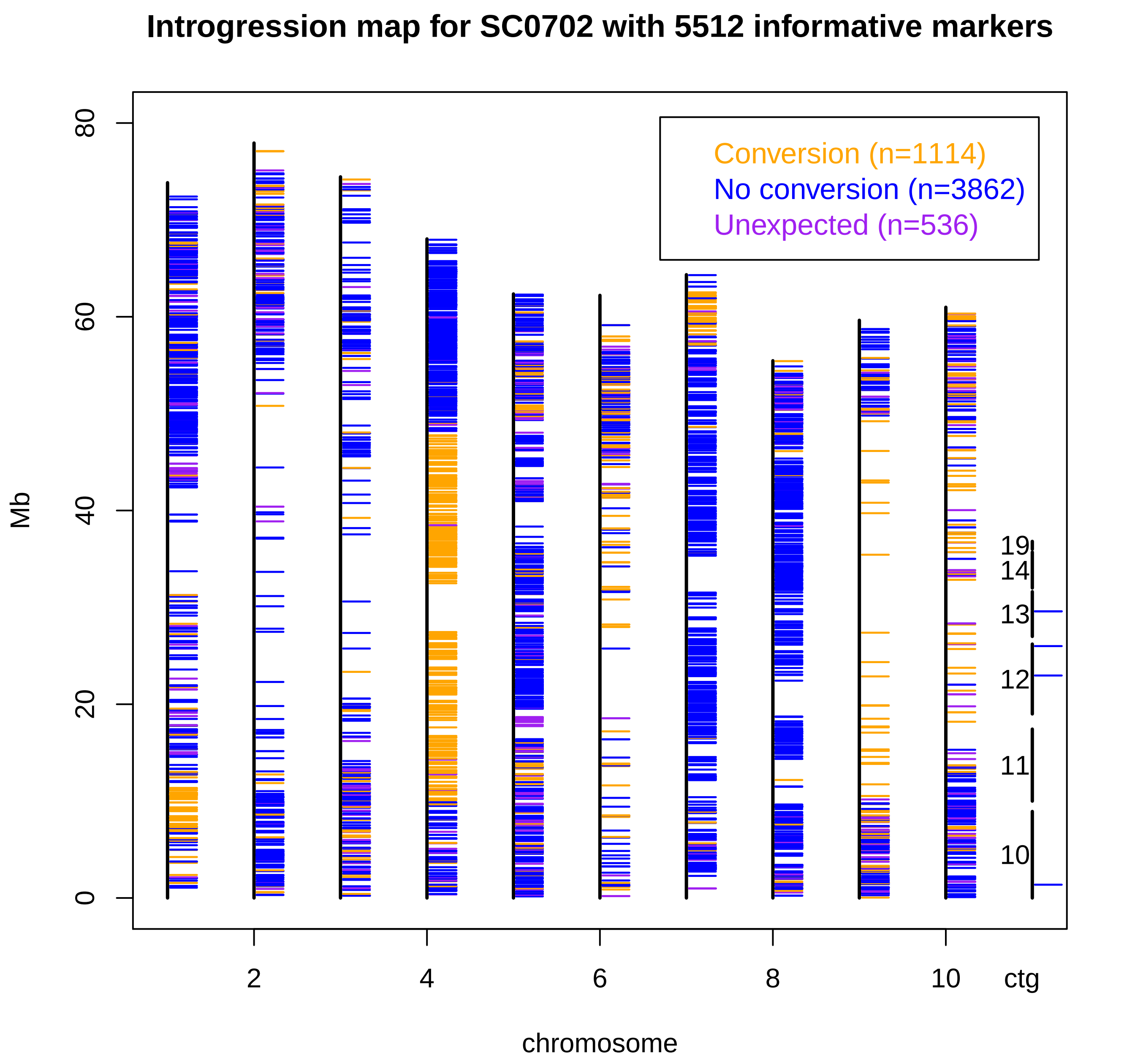

Introgression map for SC0702 with 5512 informative markers
Conversion (n=1114)
No conversion (n=3862)
Unexpected (n=536)
Mb
0
20
40
60
80
2
4
6
8
10
ctg
19
14
13
12
11
10
chromosome

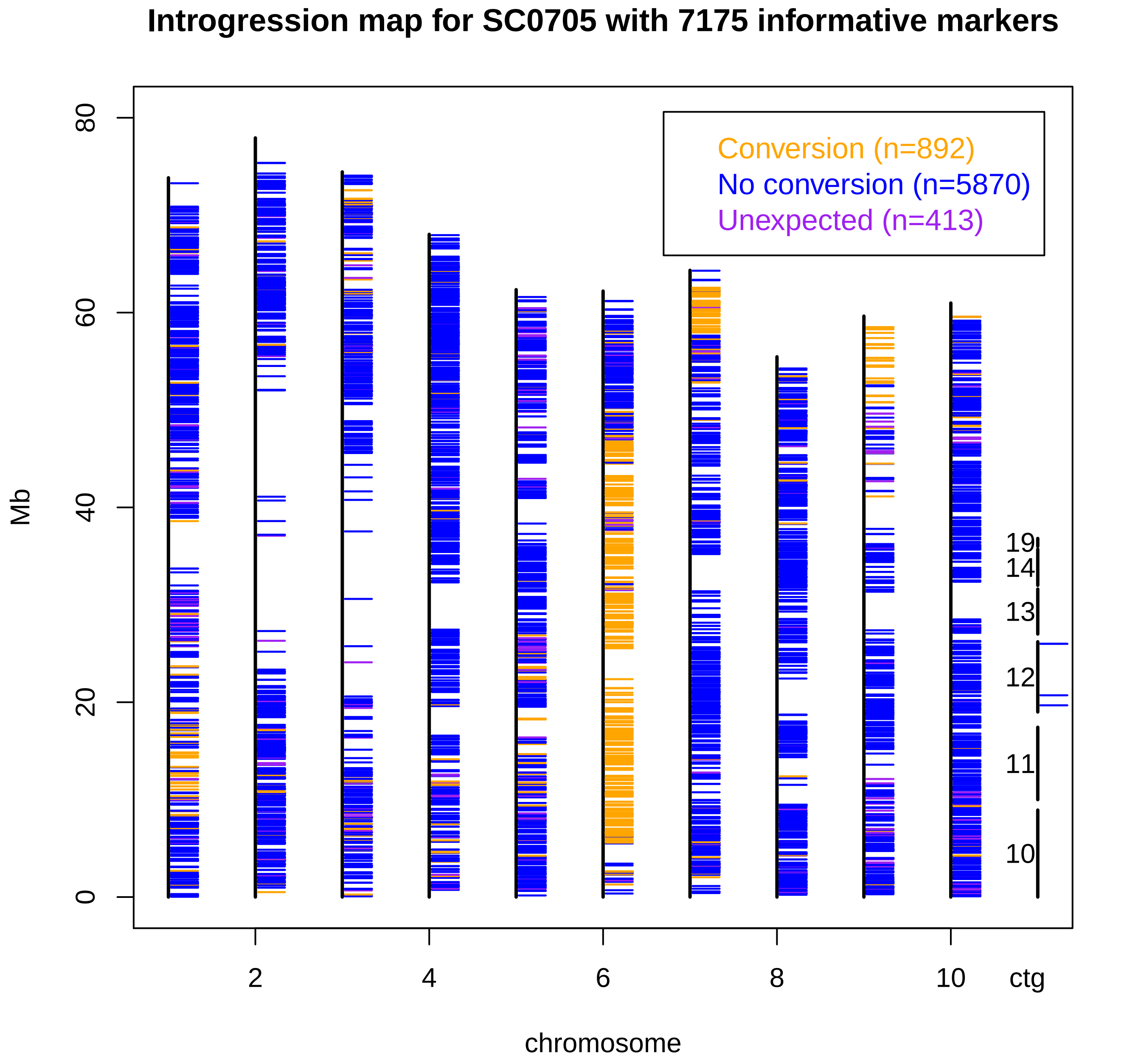

Introgression map for SC0705 with 7175 informative markers
Conversion (n=892)
No conversion (n=5870)
Unexpected (n=413)
Mb
0
20
40
60
80
2
4
6
8
10
ctg
chromosome
19
14
13
12
11
10

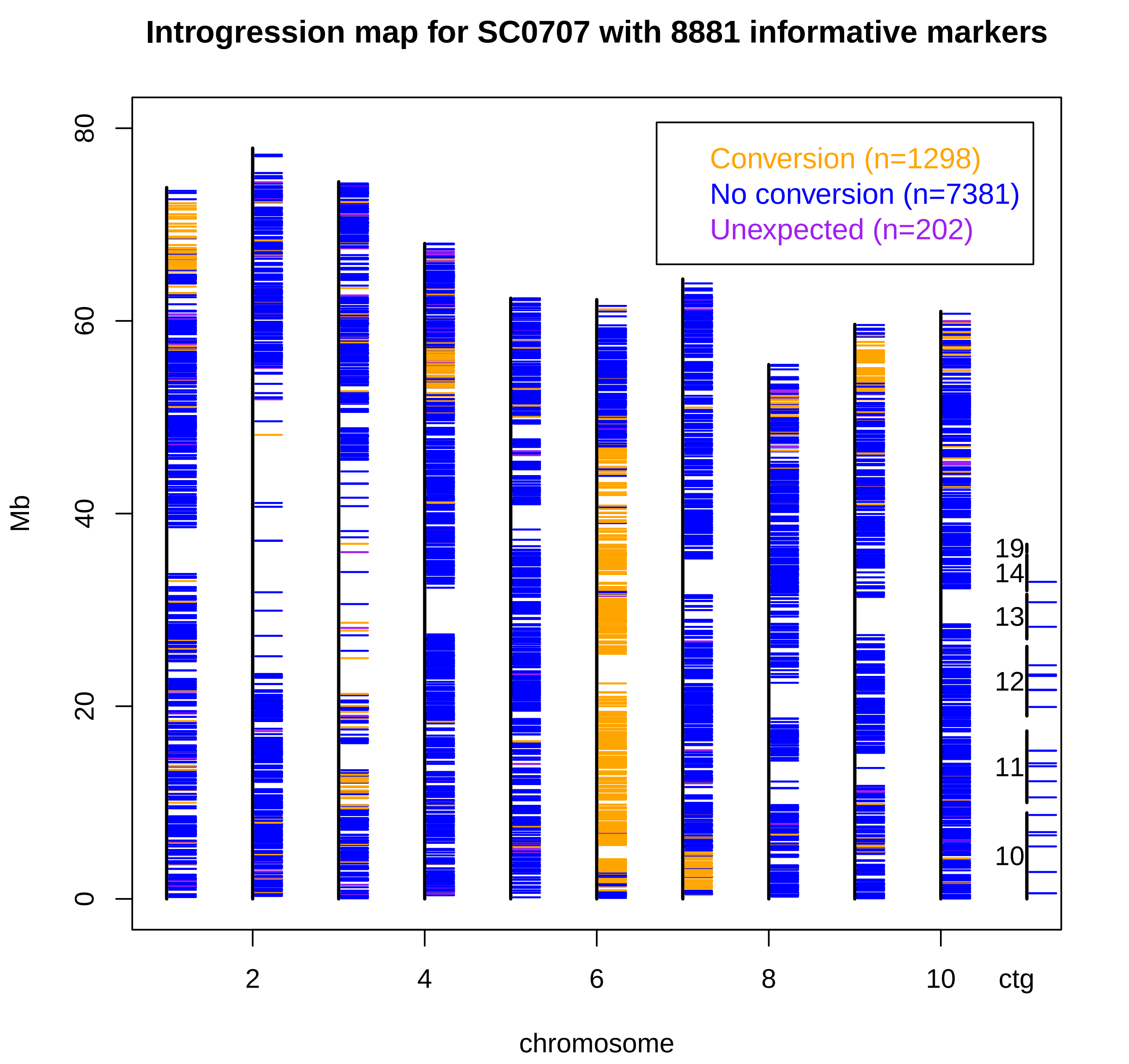

Introgression map for SC0707 with 8881 informative markers
Conversion (n=1298)
No conversion (n=7381)
Unexpected (n=202)
Mb
0
20
40
60
80
chromosome
2
4
6
8
10
ctg
19
14
13
12
11
10

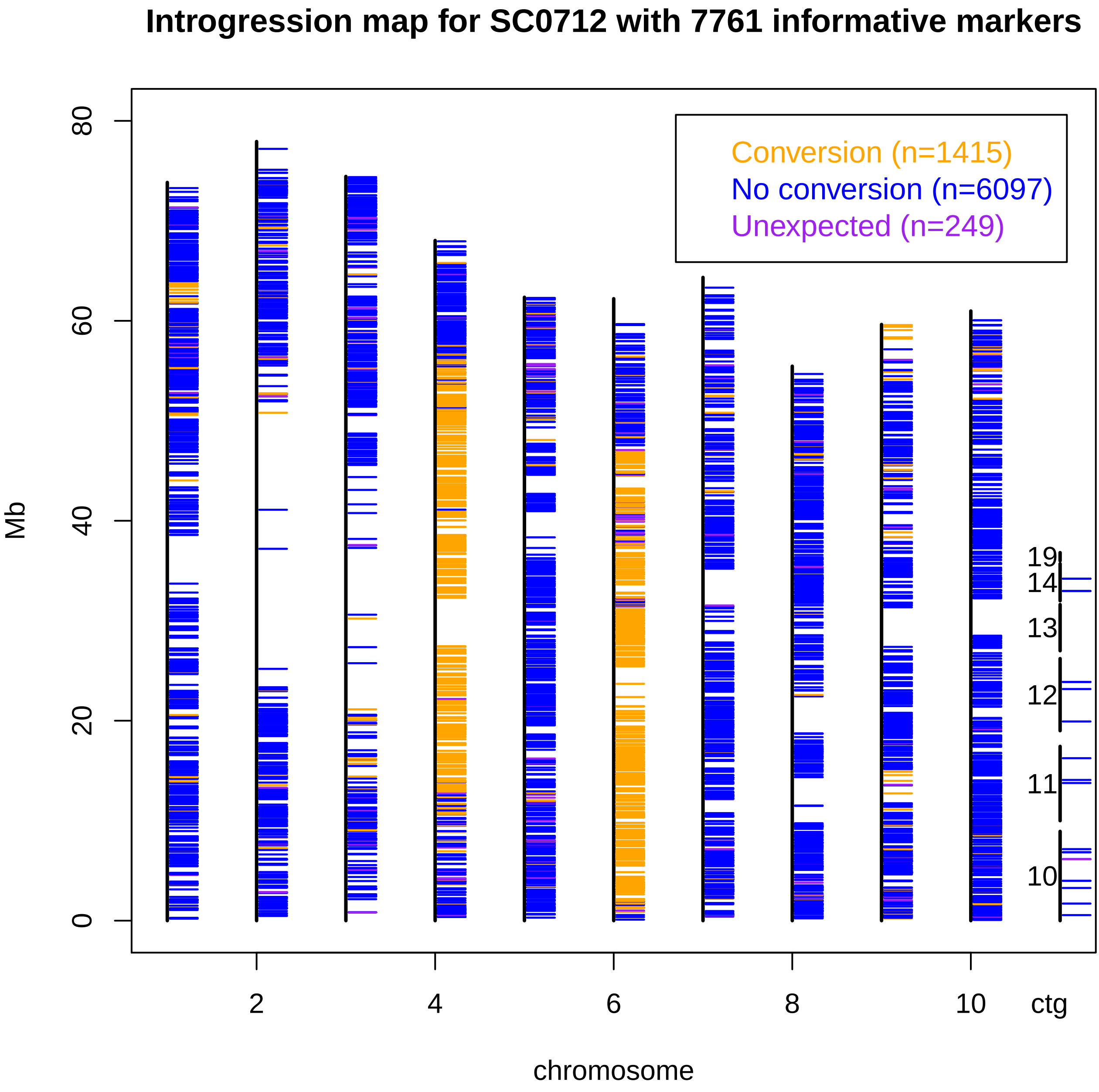

Introgression map for SC0712 with 7761 informative markers
Conversion (n=1415)
No conversion (n=6097)
Unexpected (n=249)
Mb
0
20
40
60
80
chromosome
2
4
6
8
10
ctg
19
14
13
12
11
10

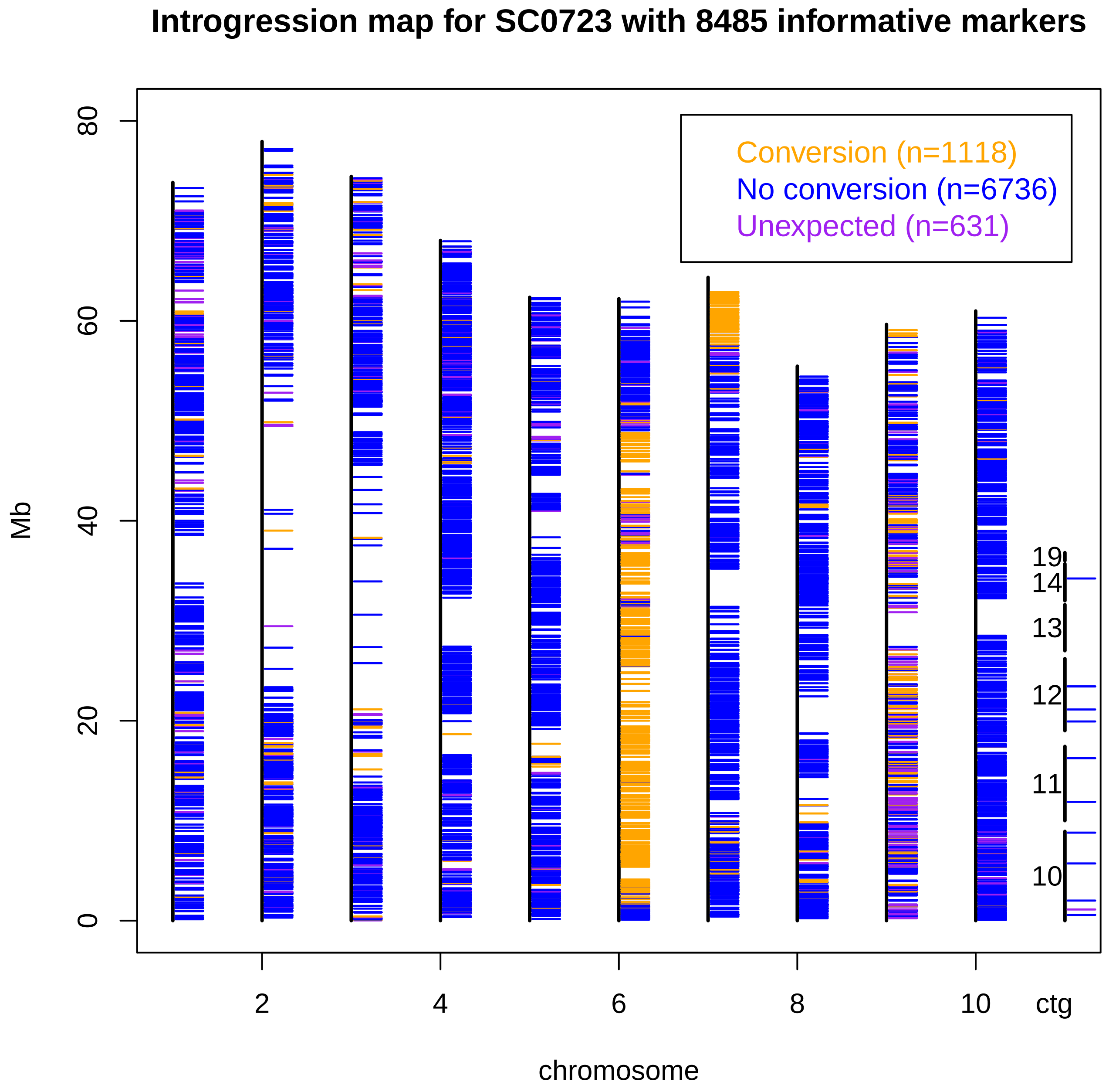

Introgression map for SC0723 with 8485 informative markers
Conversion (n=1118)
No conversion (n=6736)
Unexpected (n=631)
Mb
0
20
40
60
80
chromosome
2
4
6
8
10
ctg
19
14
13
12
11
10

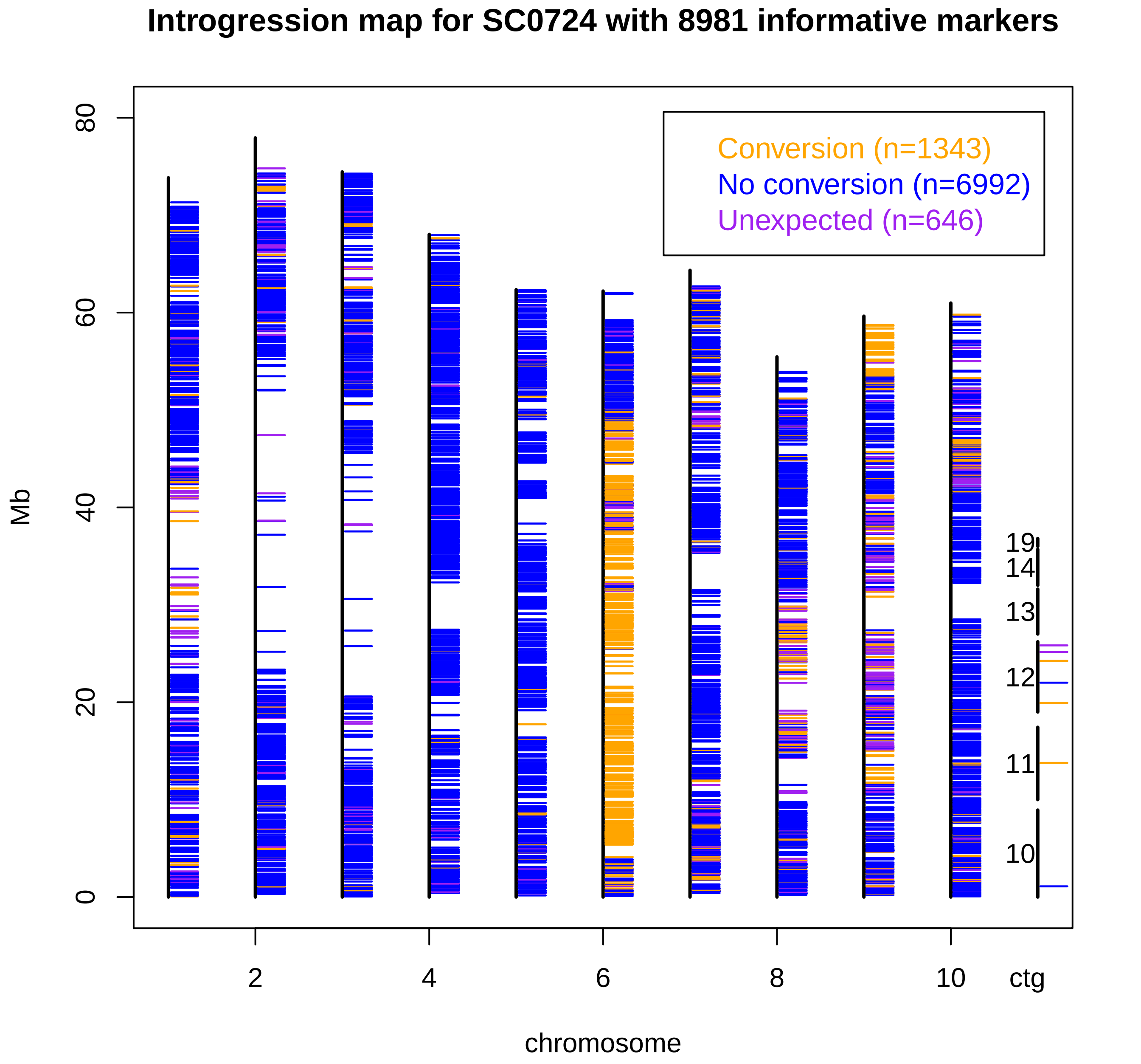

Introgression map for SC0724 with 8981 informative markers
Conversion (n=1343)
No conversion (n=6992)
Unexpected (n=646)
Mb
0
20
40
60
80
2
4
6
8
10
ctg
chromosome
19
14
13
12
11
10

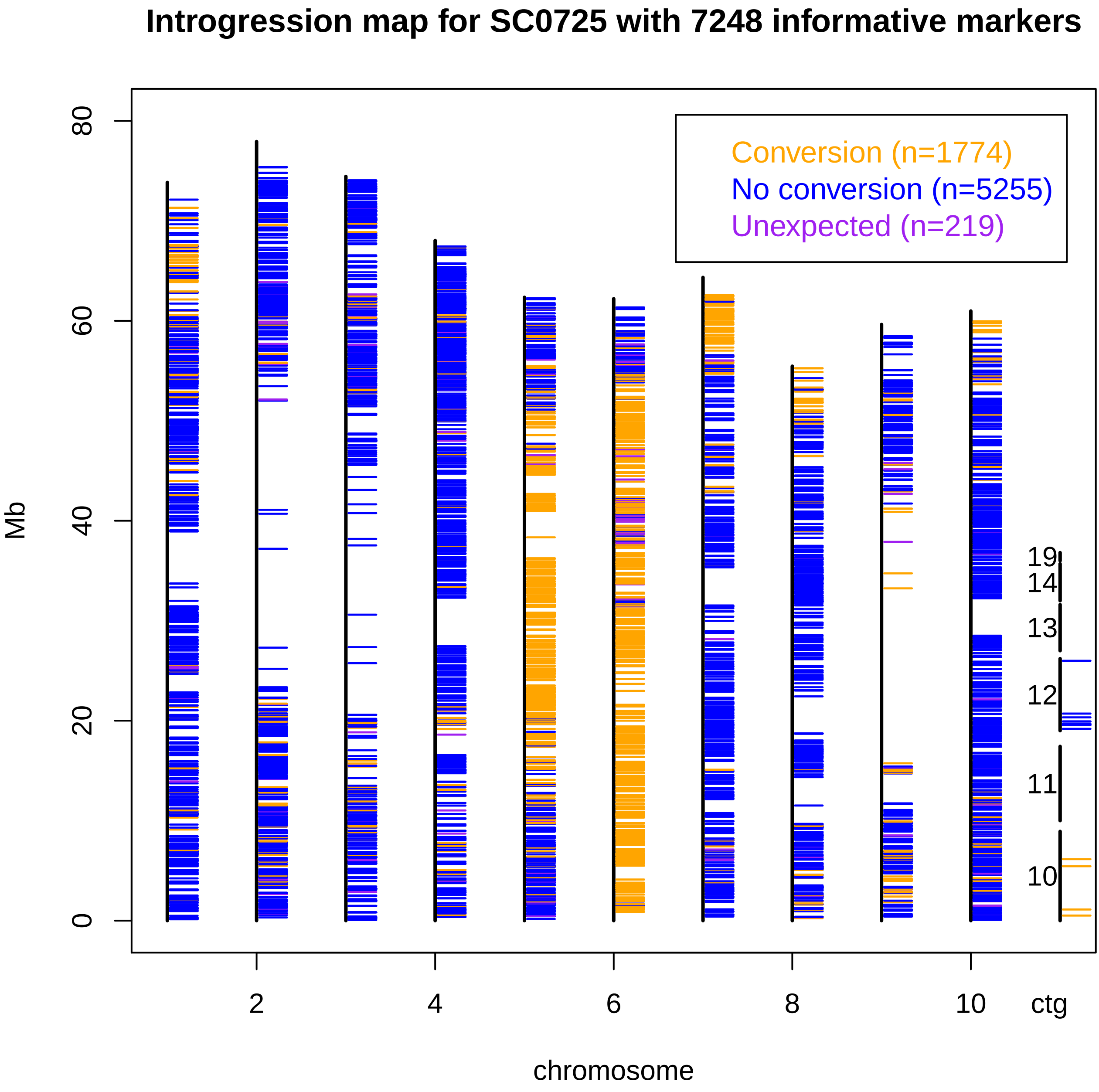

Introgression map for SC0725 with 7248 informative markers
Conversion (n=1774)
No conversion (n=5255)
Unexpected (n=219)
Mb
0
20
40
60
80
chromosome
2
4
6
8
10
ctg
19
14
13
12
11
10

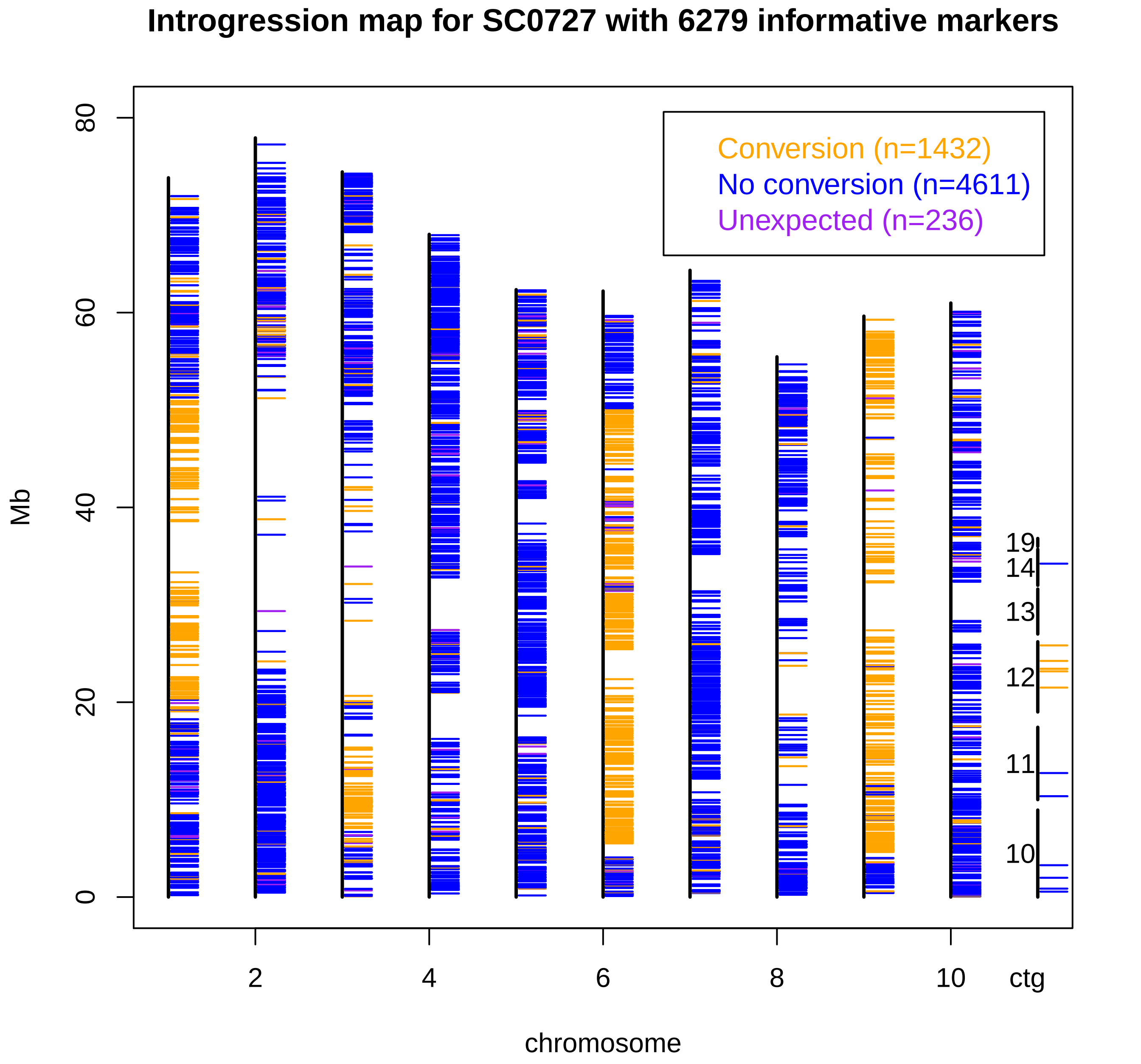

Introgression map for SC0727 with 6279 informative markers
Conversion (n=1432)
No conversion (n=4611)
Unexpected (n=236)
Mb
0
20
40
60
80
2
4
6
8
10
ctg
19
14
13
12
11
10
chromosome

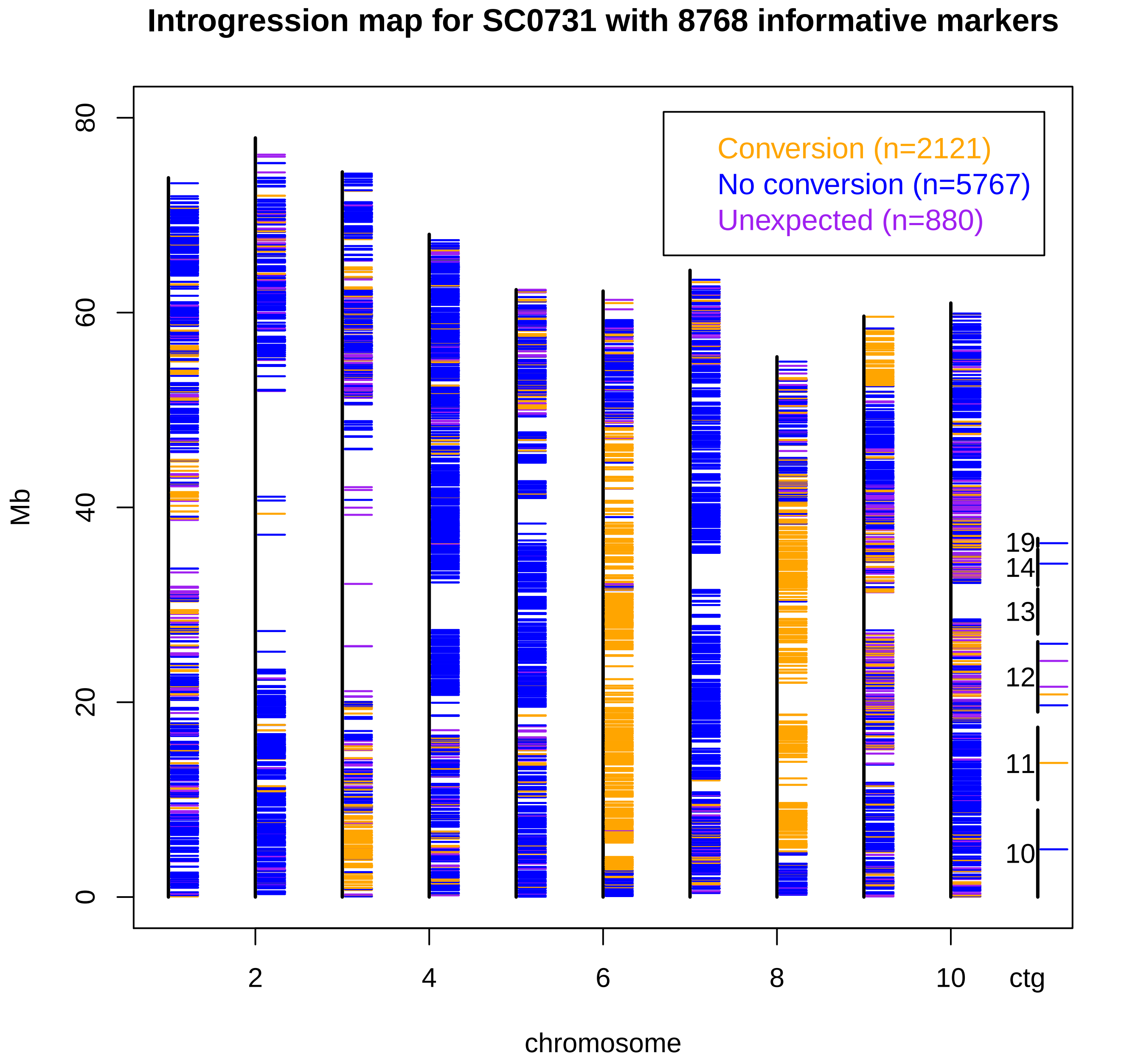

Introgression map for SC0731 with 8768 informative markers
Conversion (n=2121)
No conversion (n=5767)
Unexpected (n=880)
Mb
0
20
40
60
80
2
4
6
8
10
ctg
19
14
13
12
11
10
chromosome

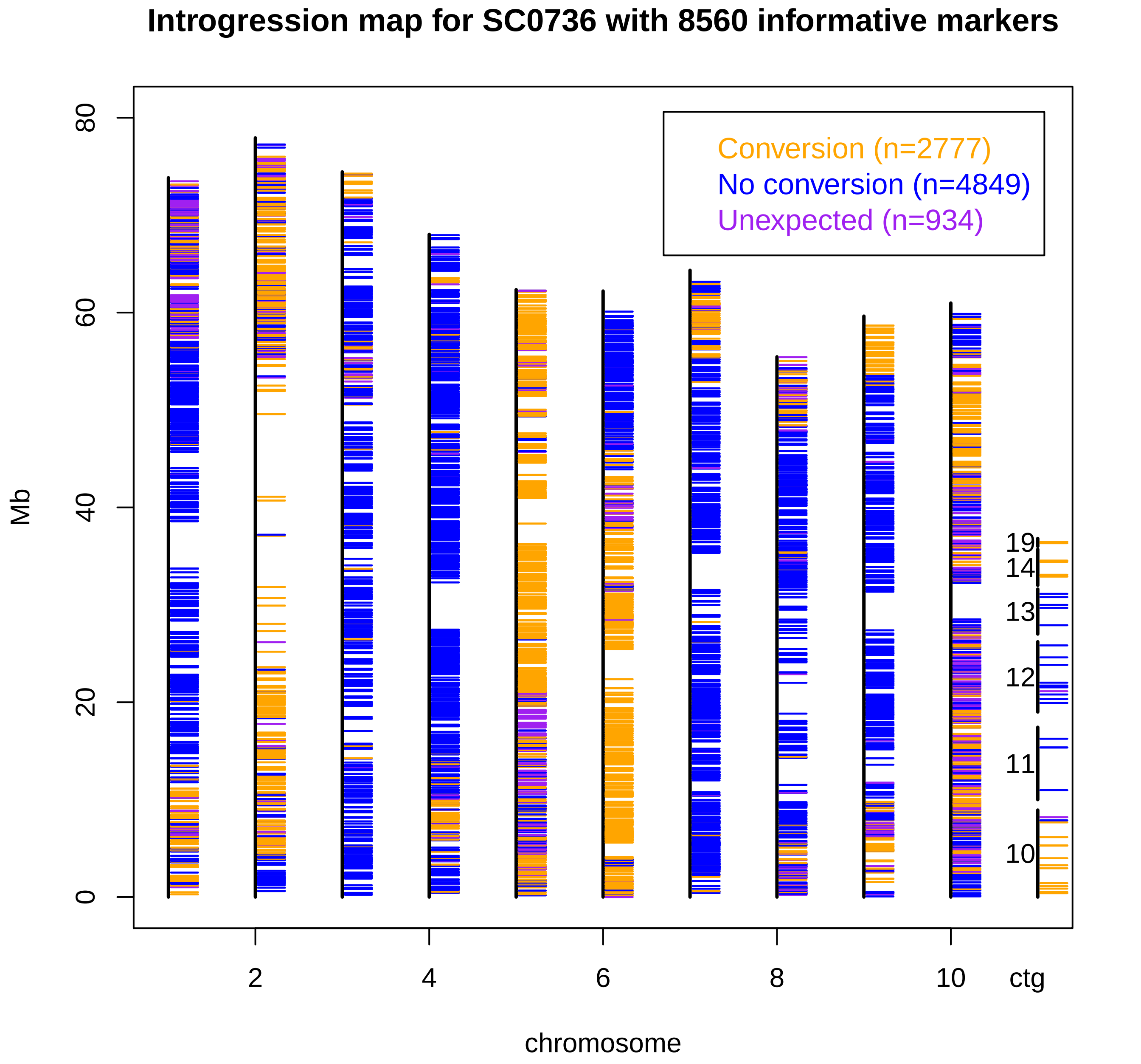
Introgression map for SC0736 with 8560 informative markers
Conversion (n=2777)
No conversion (n=4849)
Unexpected (n=934)
Mb
0
20
40
60
80
chromosome
2
4
6
8
10
ctg
19
14
13
12
11
10

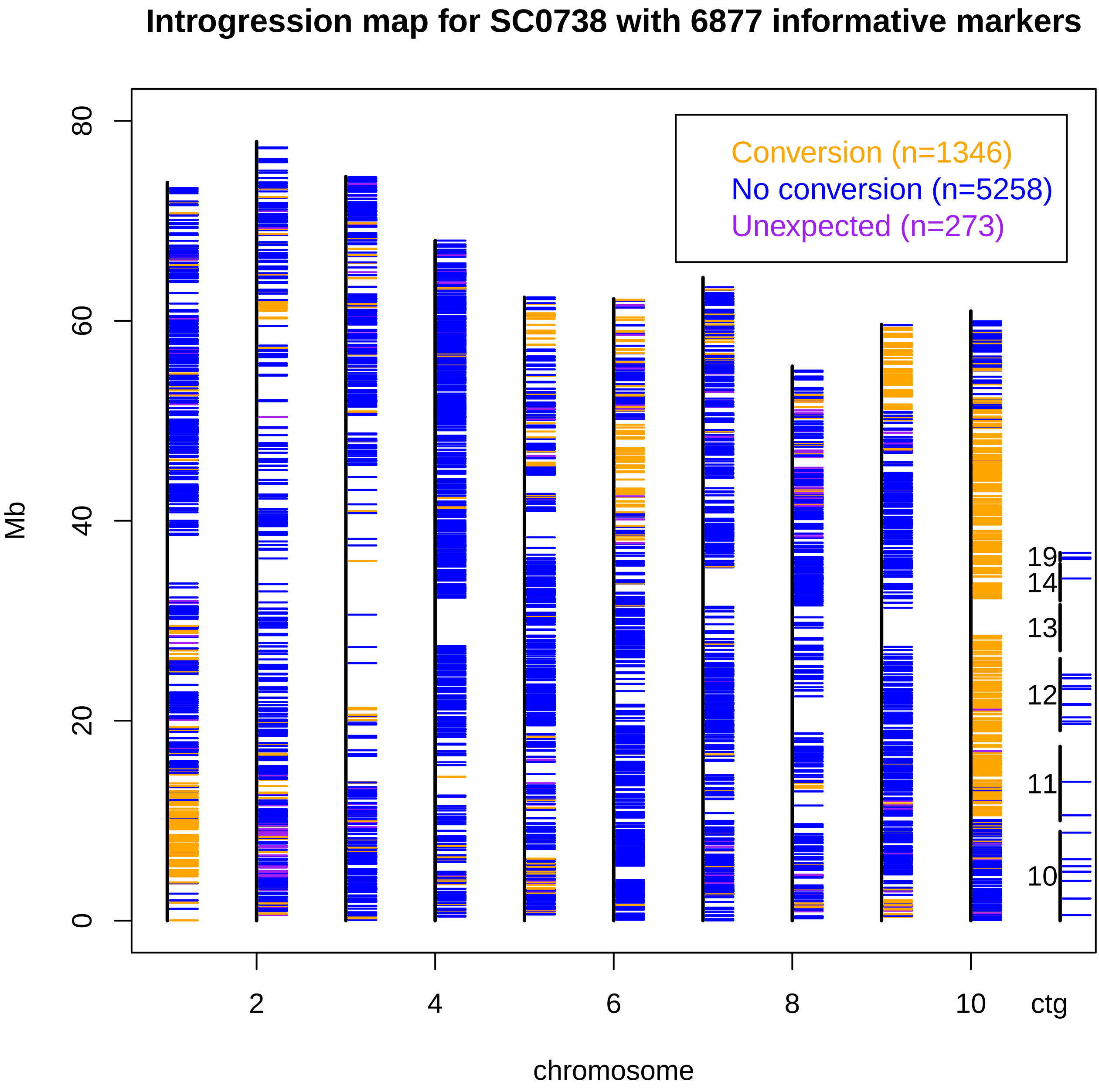
Introgression map for SC0738 with 6877 informative markers
Conversion (n=1346)
No conversion (n=5258)
Unexpected (n=273)
Mb
0
20
40
60
80
chromosome
2
4
6
8
10
ctg
19
14
13
12
11
10

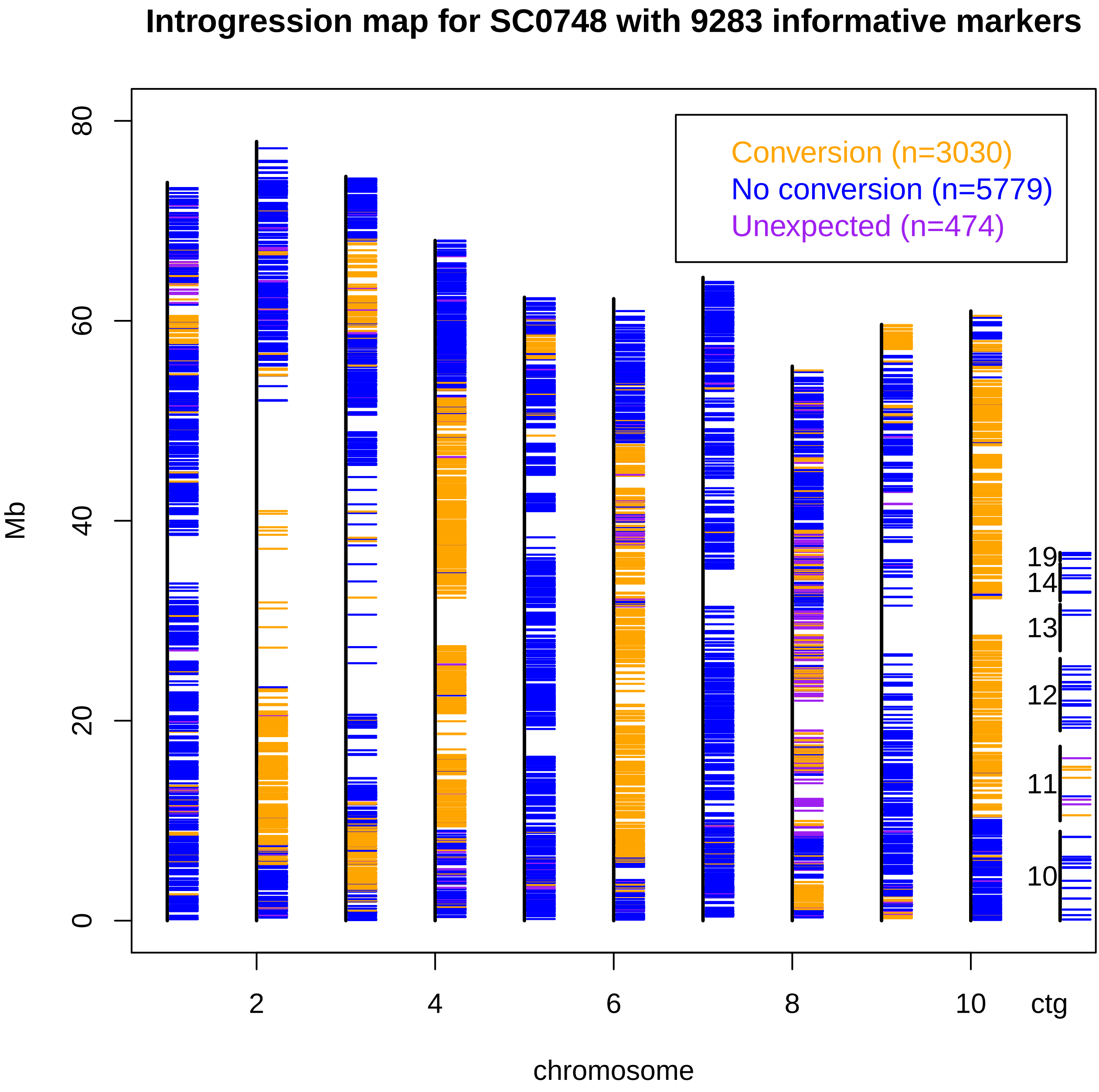
Introgression map for SC0748 with 9283 informative markers
Conversion (n=3030)
No conversion (n=5779)
Unexpected (n=474)
Mb
0
20
40
60
80
chromosome
2
4
6
8
10
ctg
19
14
13
12
11
10

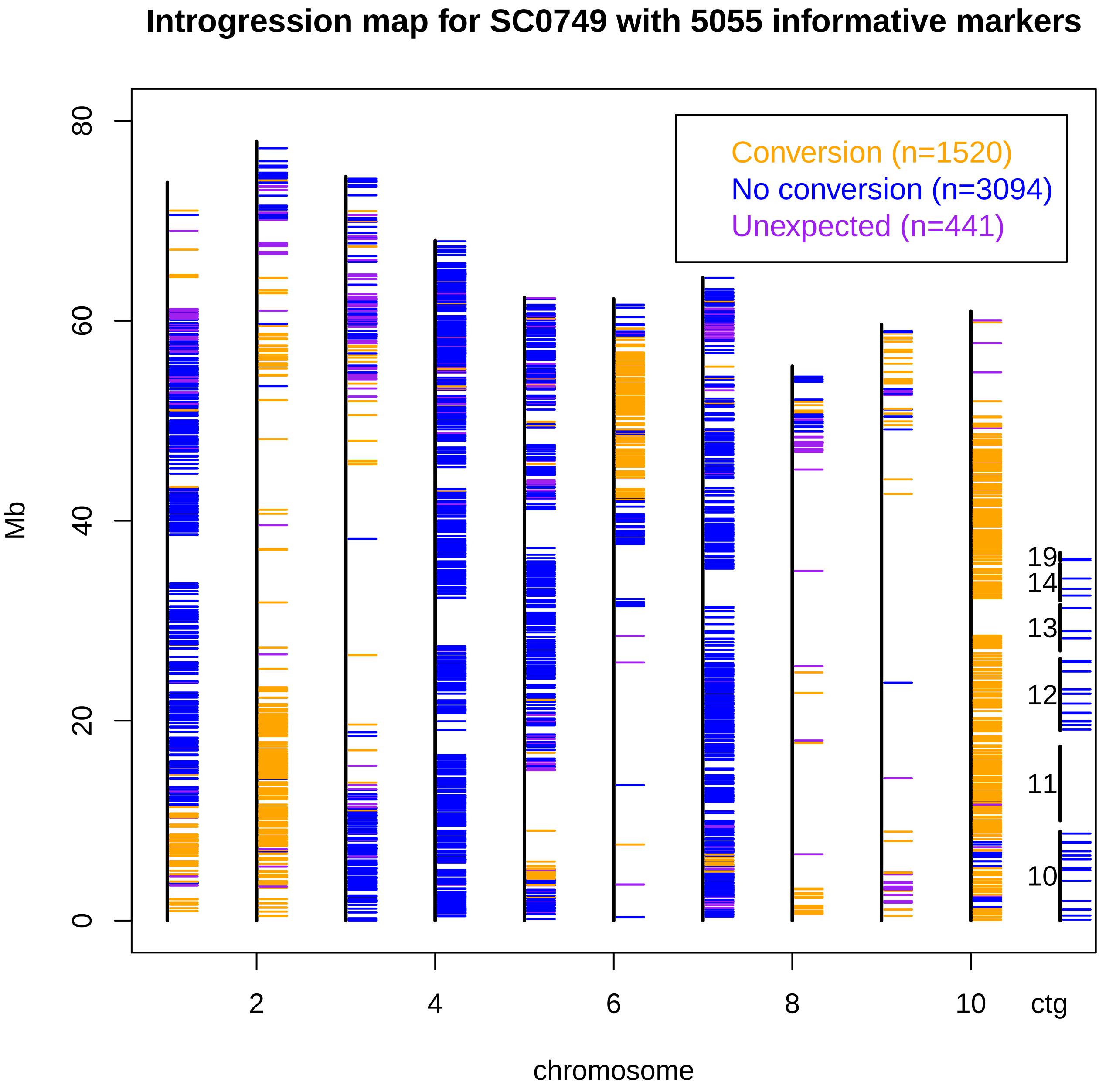

Introgression map for SC0749 with 5055 informative markers
Conversion (n=1520)
No conversion (n=3094)
Unexpected (n=441)
Mb
0
20
40
60
80
2
4
6
8
10
ctg
19
14
13
12
11
10
chromosome

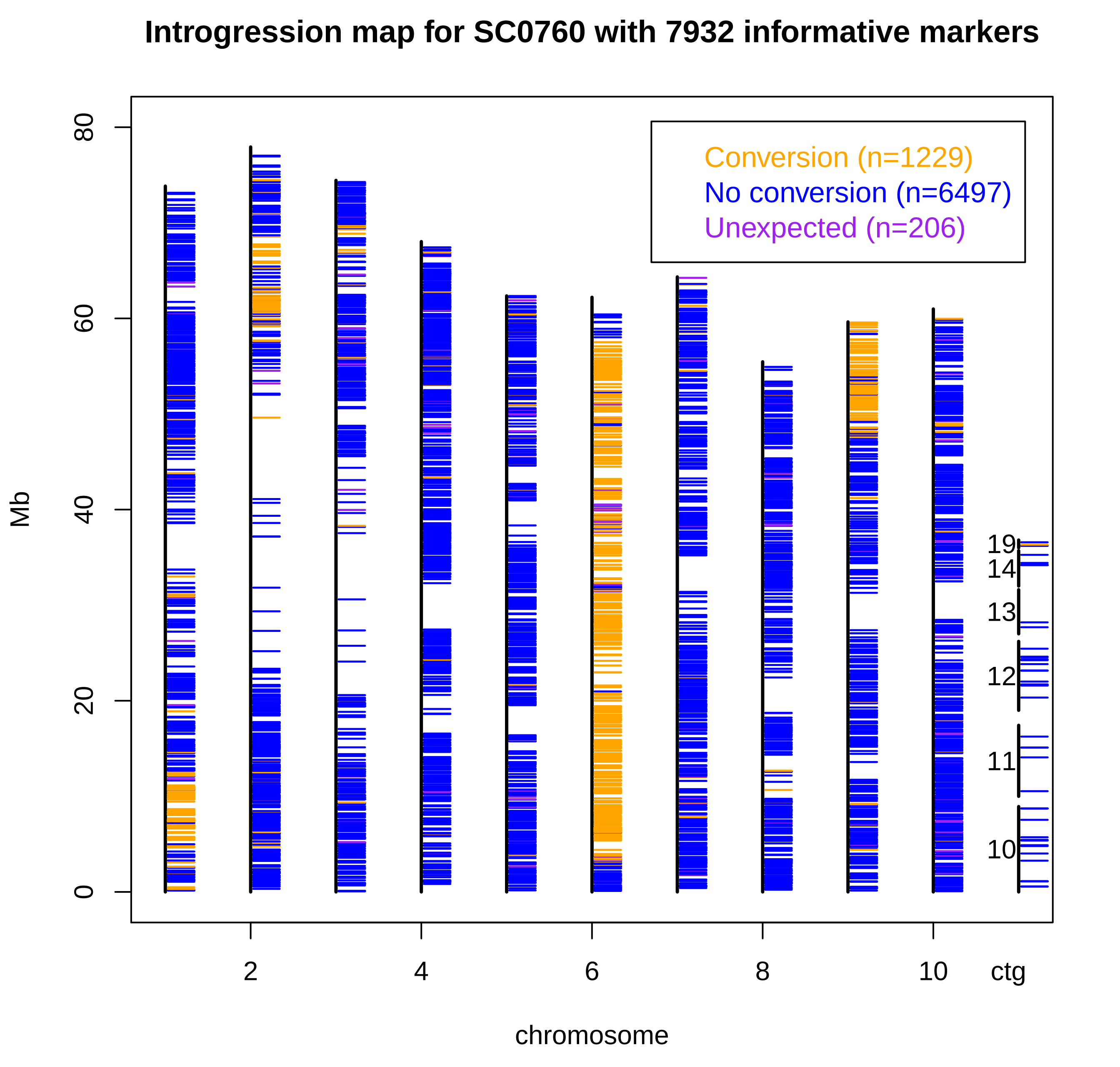

Introgression map for SC0760 with 7932 informative markers
Conversion (n=1229)
No conversion (n=6497)
Unexpected (n=206)
Mb
0
20
40
60
80
chromosome
2
4
6
8
10
ctg
19
14
13
12
11
10

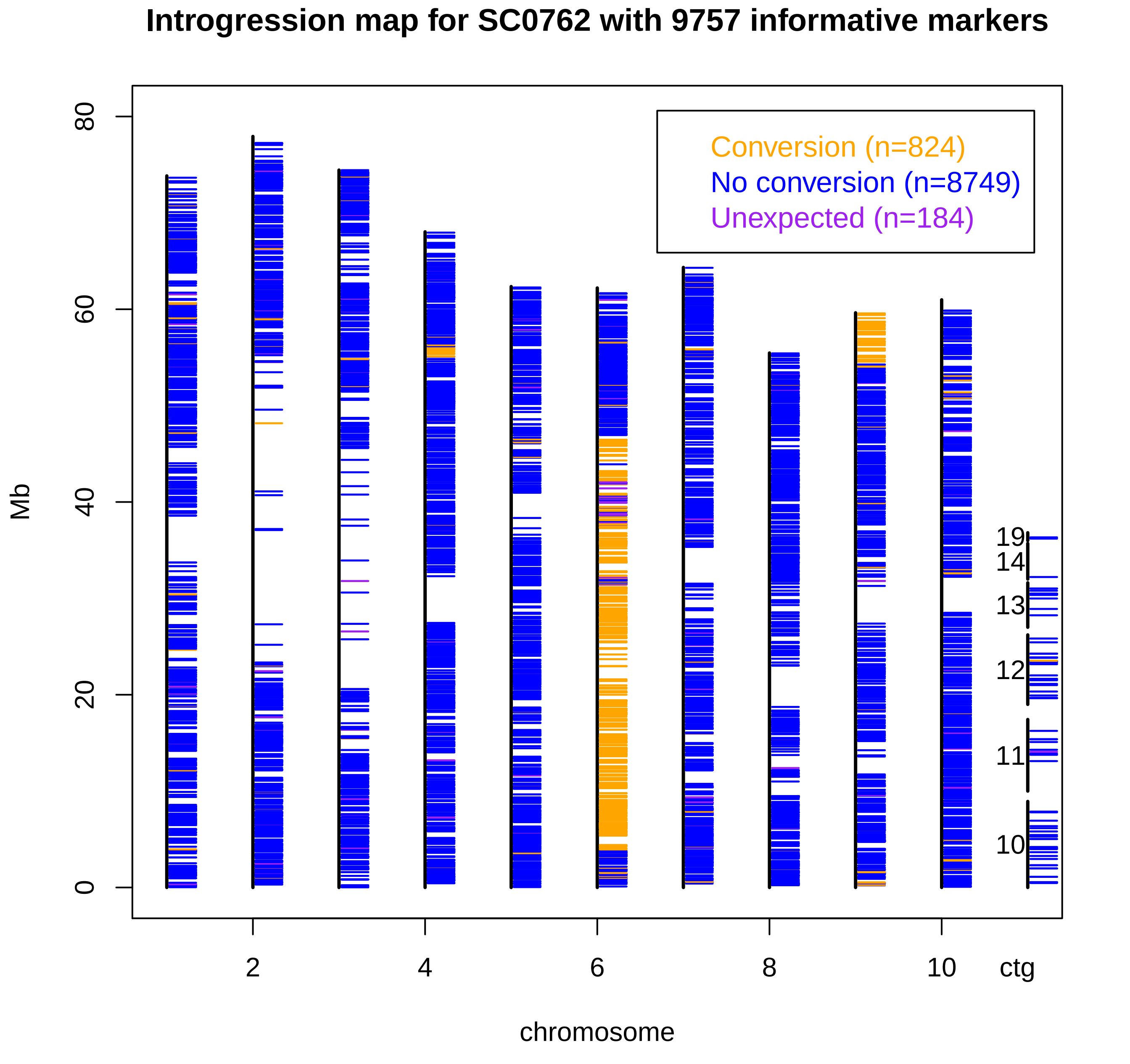

Introgression map for SC0762 with 9757 informative markers
Conversion (n=824)
No conversion (n=8749)
Unexpected (n=184)
Mb
0
20
40
60
80
chromosome
2
4
6
8
10
ctg
19
14
13
12
11
10

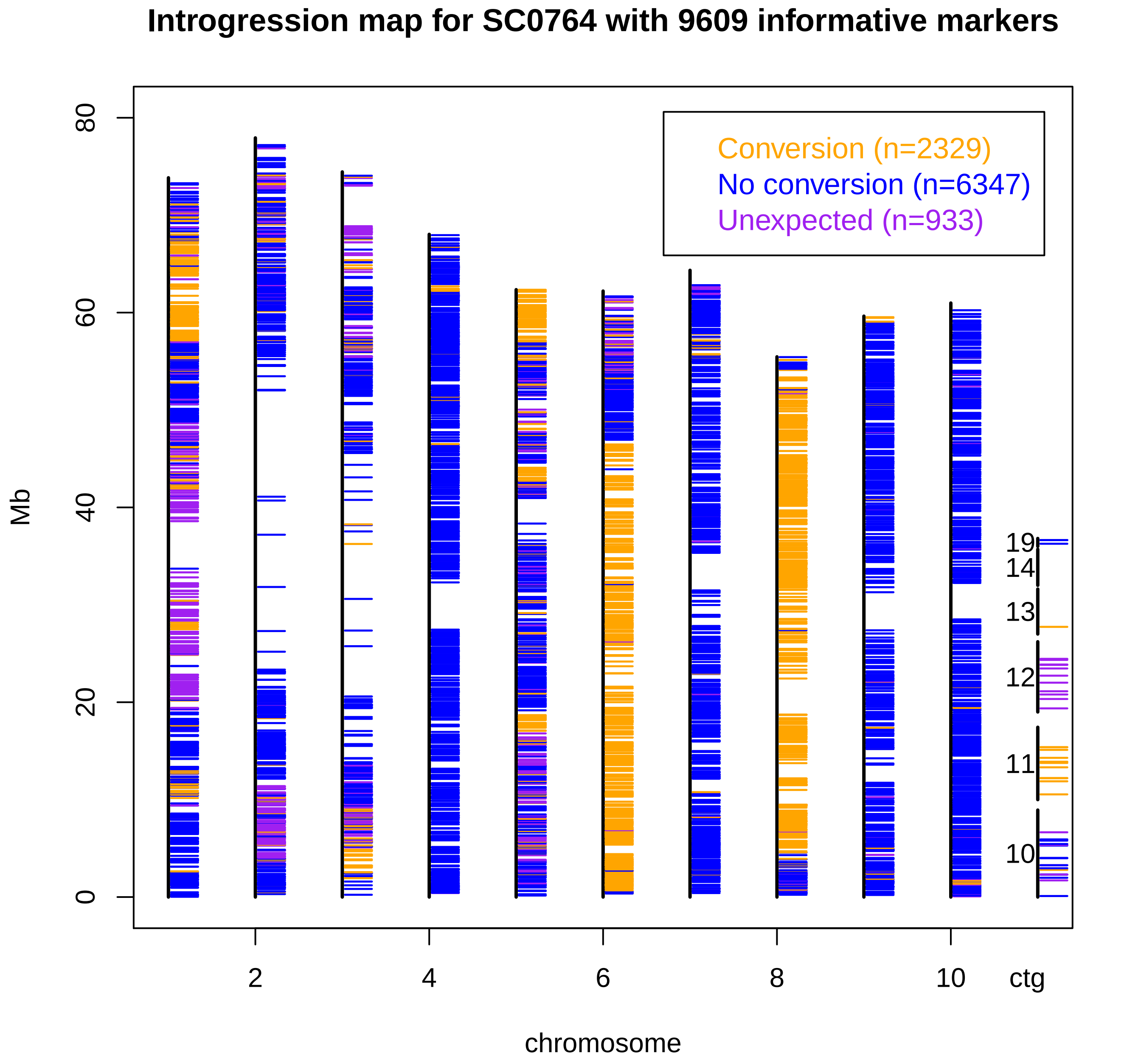
Introgression map for SC0764 with 9609 informative markers
Conversion (n=2329)
No conversion (n=6347)
Unexpected (n=933)
Mb
0
20
40
60
80
2
4
6
8
10
ctg
19
14
13
12
11
10
chromosome

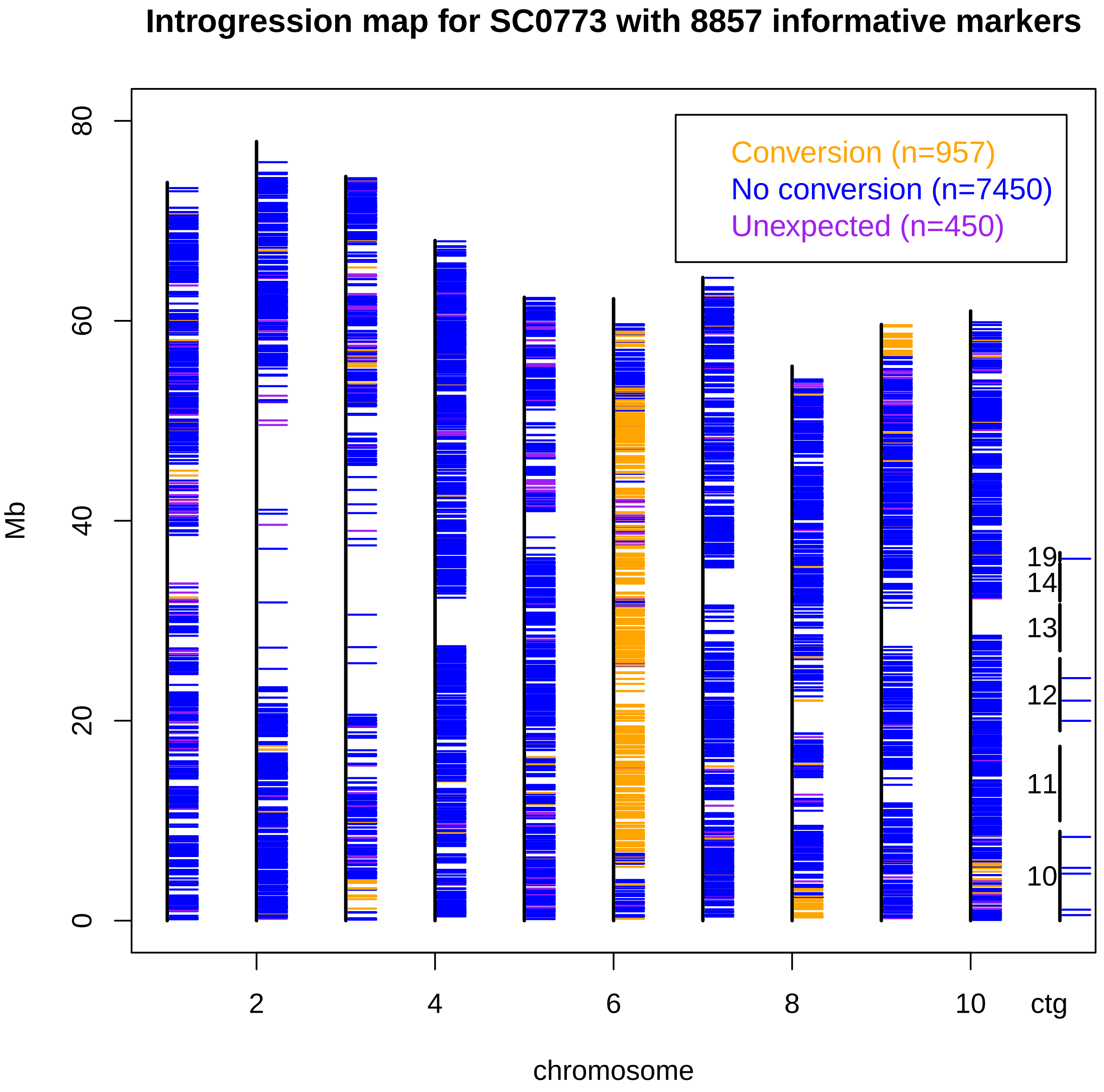
Introgression map for SC0773 with 8857 informative markers
Conversion (n=957)
No conversion (n=7450)
Unexpected (n=450)
Mb
0
20
40
60
80
2
4
6
8
10
ctg
19
14
13
12
11
10
chromosome

Introgression map for SC0774 with 8862 informative markers

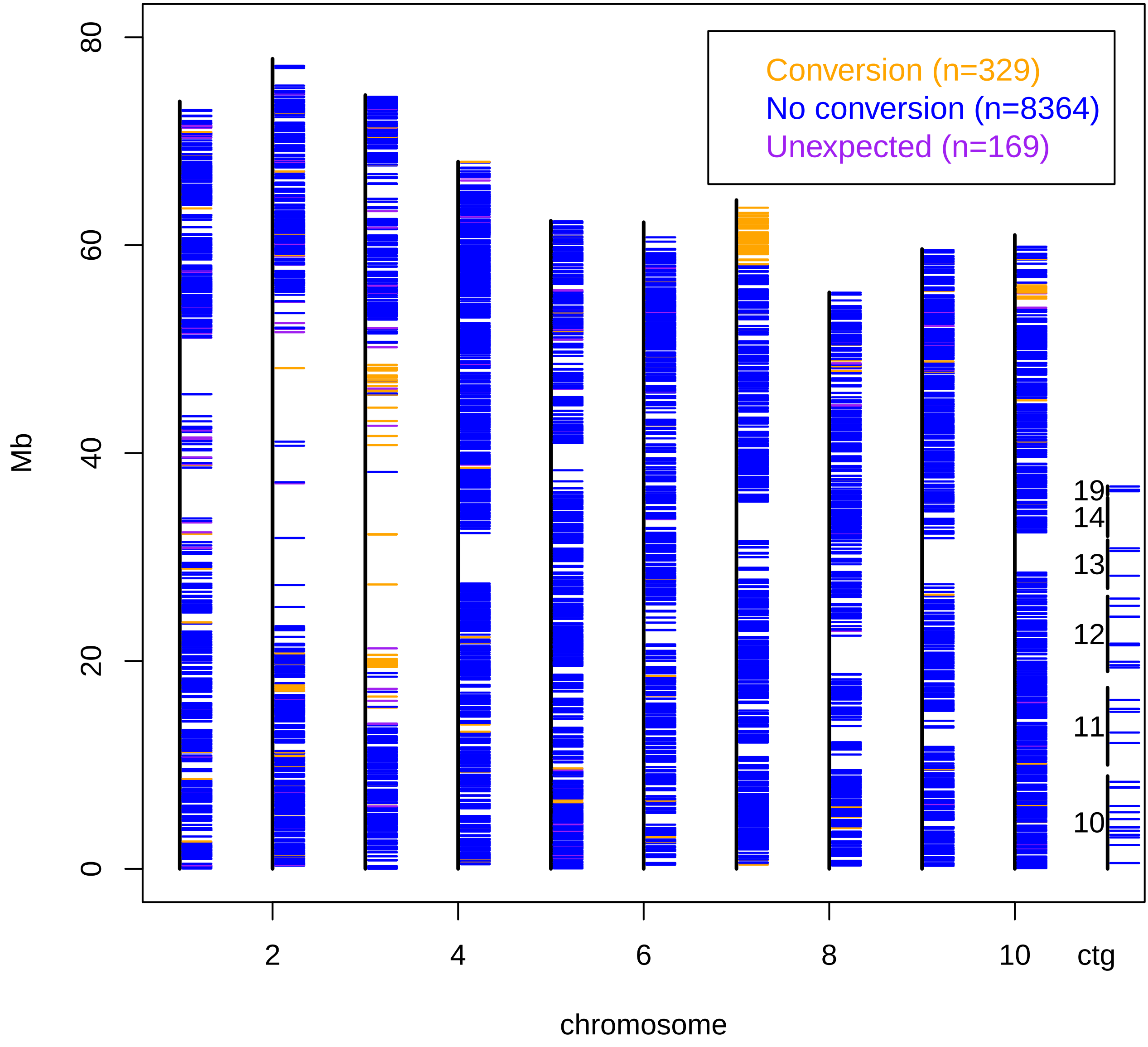

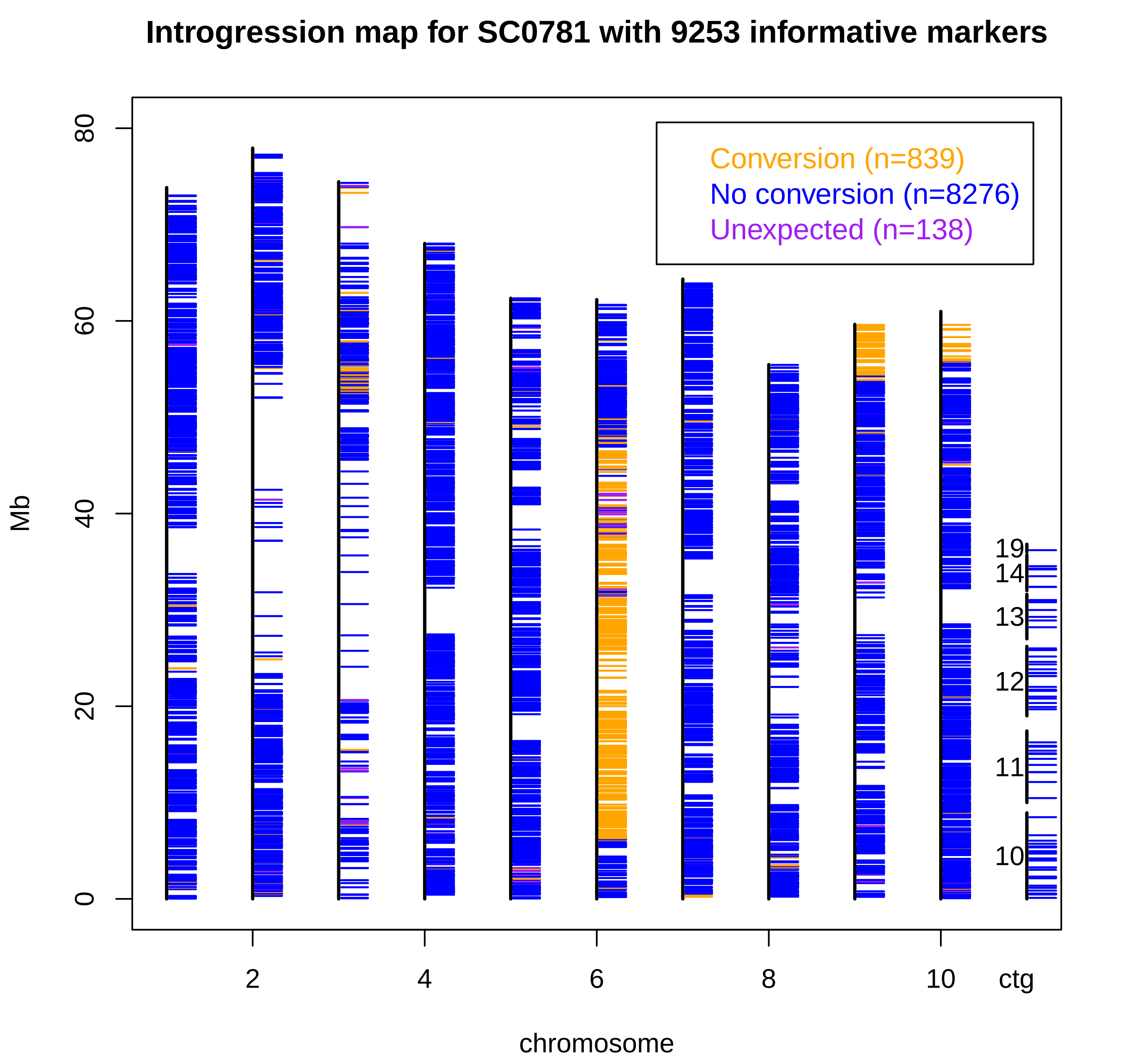
Introgression map for SC0781 with 9253 informative markers
Conversion (n=839)
No conversion (n=8276)
Unexpected (n=138)
Mb
0
20
40
60
80
chromosome
2
4
6
8
10
ctg
19
14
13
12
11
10

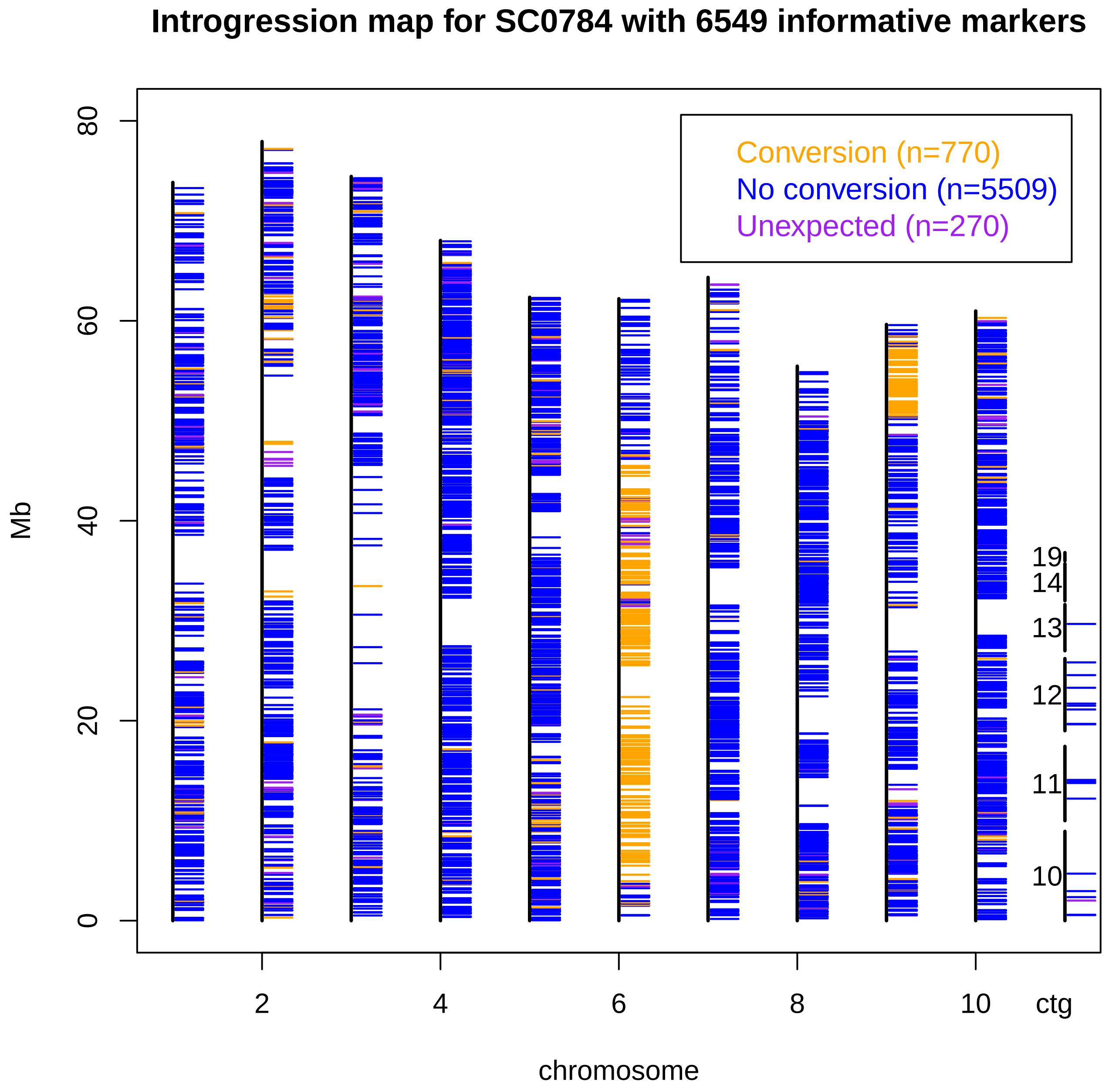

Introgression map for SC0784 with 6549 informative markers
Conversion (n=770)
No conversion (n=5509)
Unexpected (n=270)
Mb
0
20
40
60
80
2
4
6
8
10
ctg
19
14
13
12
11
10
chromosome

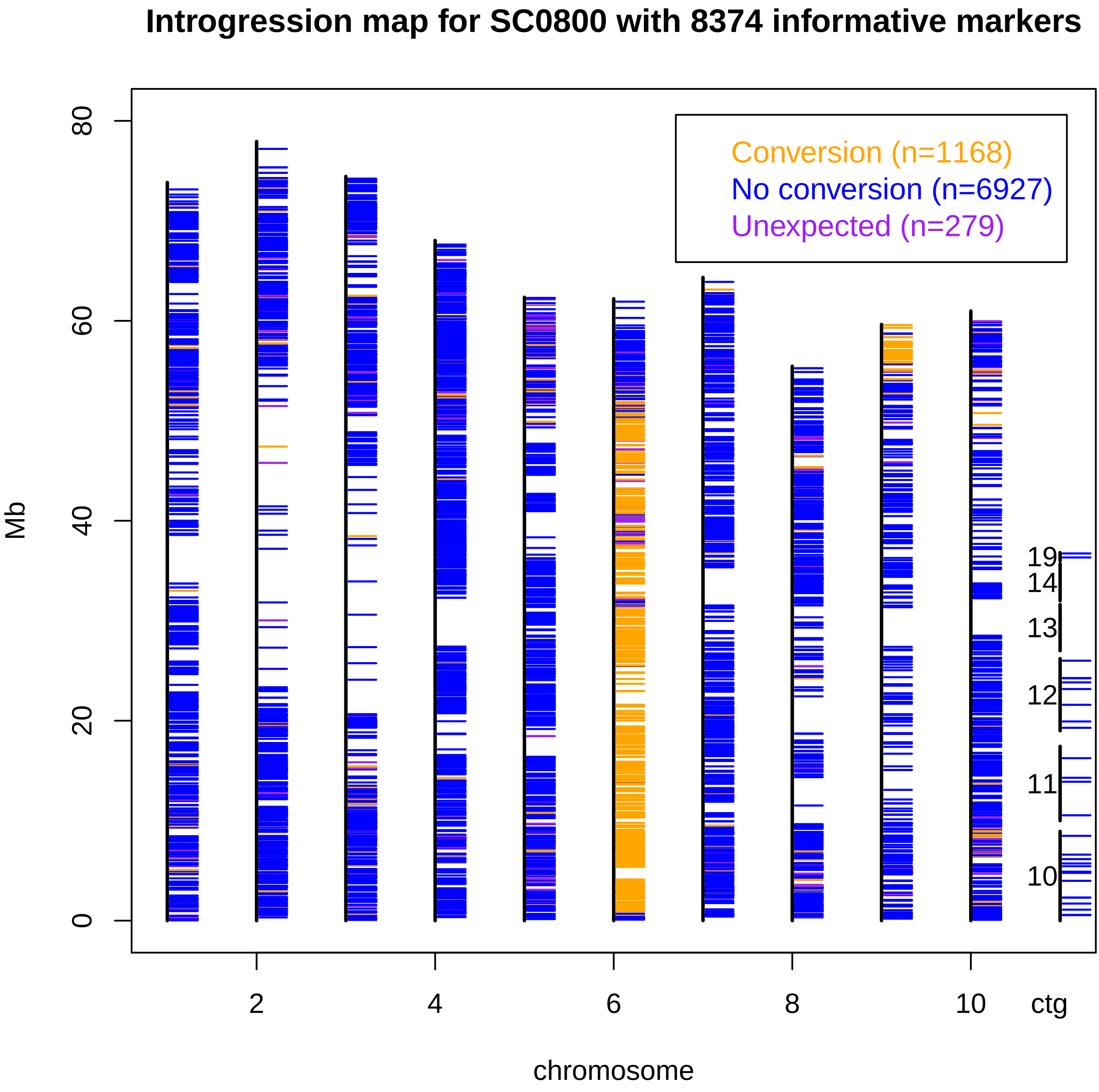
Introgression map for SC0800 with 8374 informative markers
Conversion (n=1168)
No conversion (n=6927)
Unexpected (n=279)
Mb
0
20
40
60
80
2
4
6
8
10
ctg
19
14
13
12
11
10
chromosome

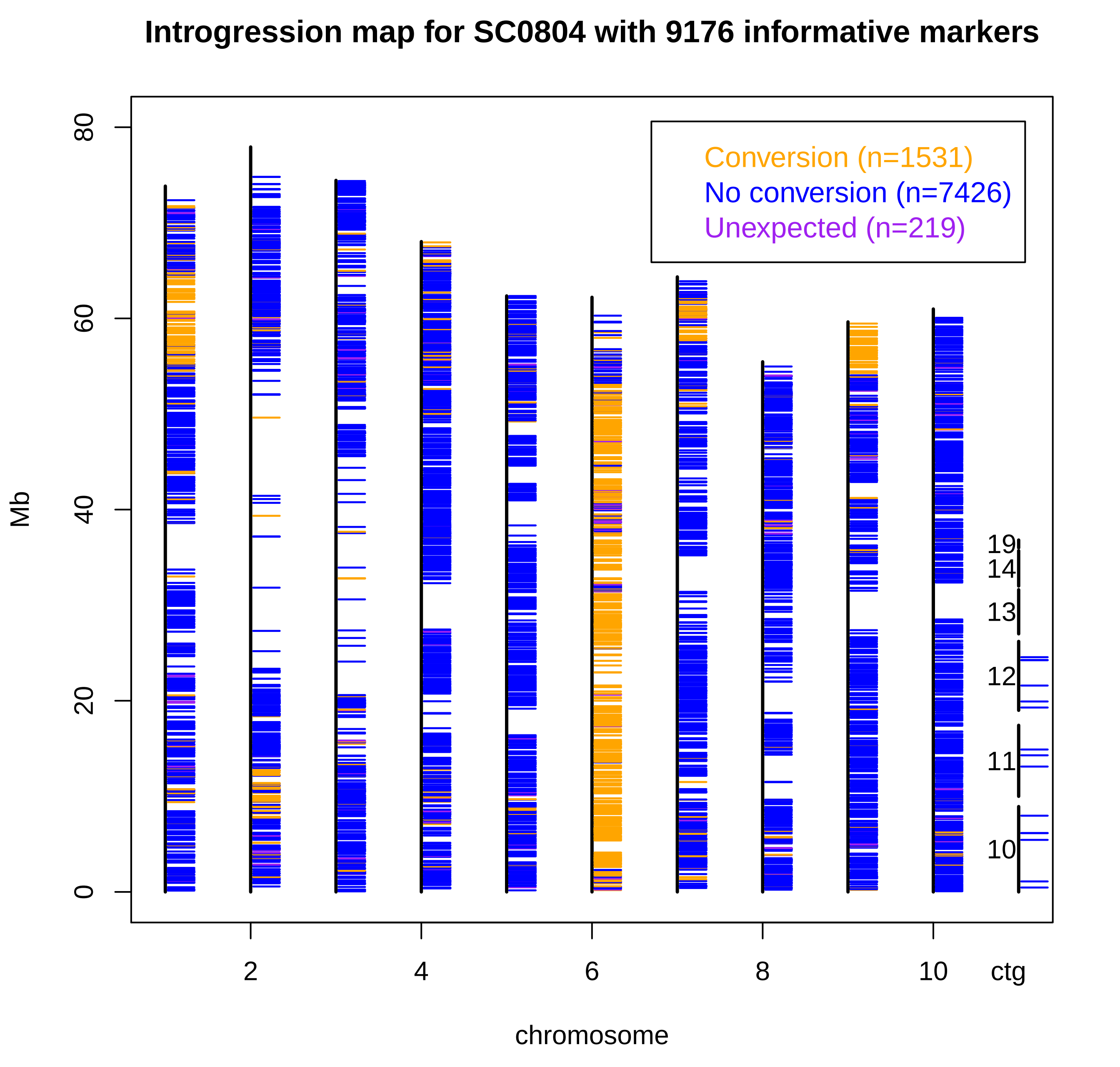

Introgression map for SC0804 with 9176 informative markers
Conversion (n=1531)
No conversion (n=7426)
Unexpected (n=219)
Mb
0
20
40
60
80
chromosome
2
4
6
8
10
ctg
19
14
13
12
11
10

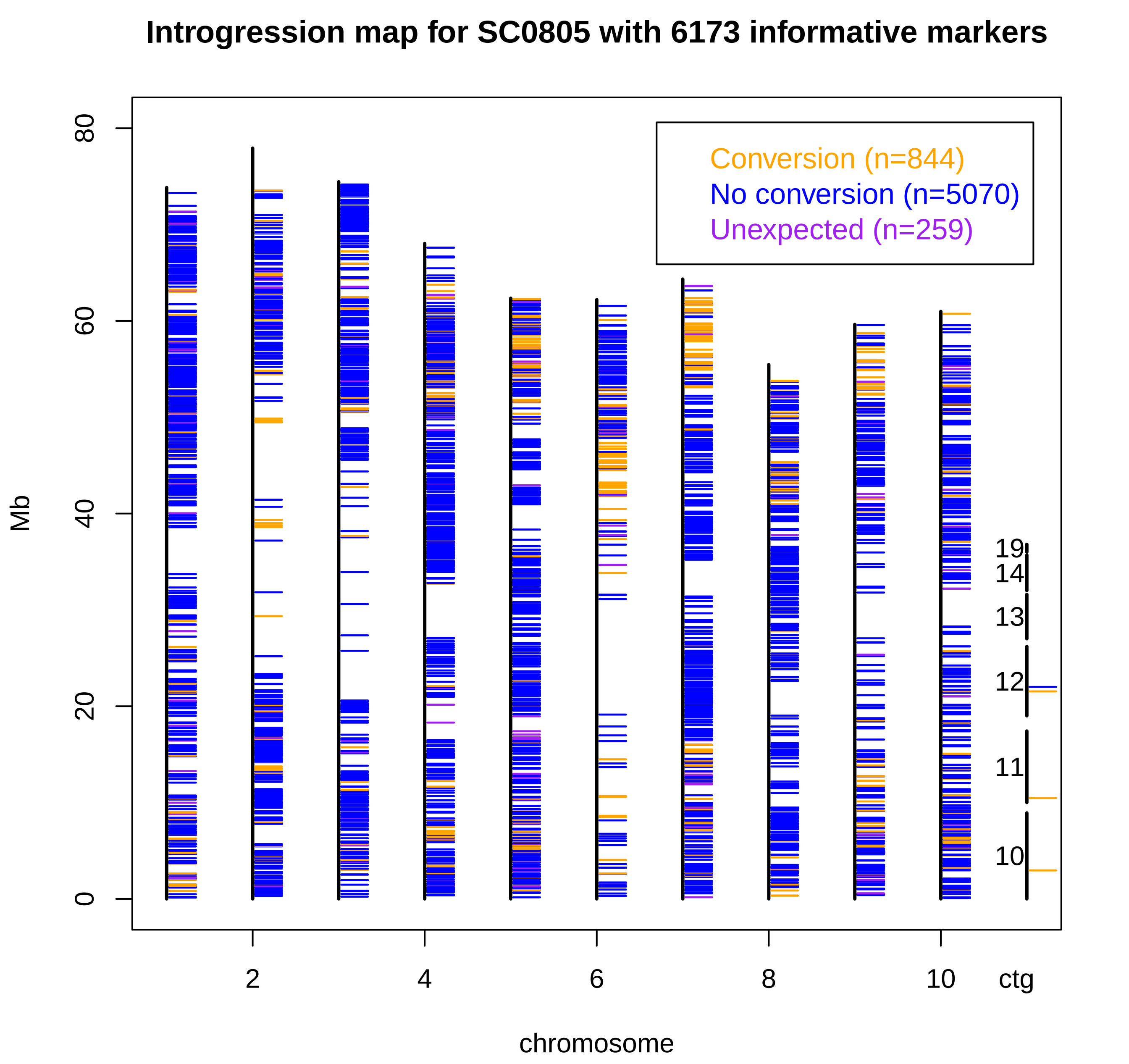

Introgression map for SC0805 with 6173 informative markers
Conversion (n=844)
No conversion (n=5070)
Unexpected (n=259)
Mb
0
20
40
60
80
2
4
6
8
10
ctg
19
14
13
12
11
10
chromosome

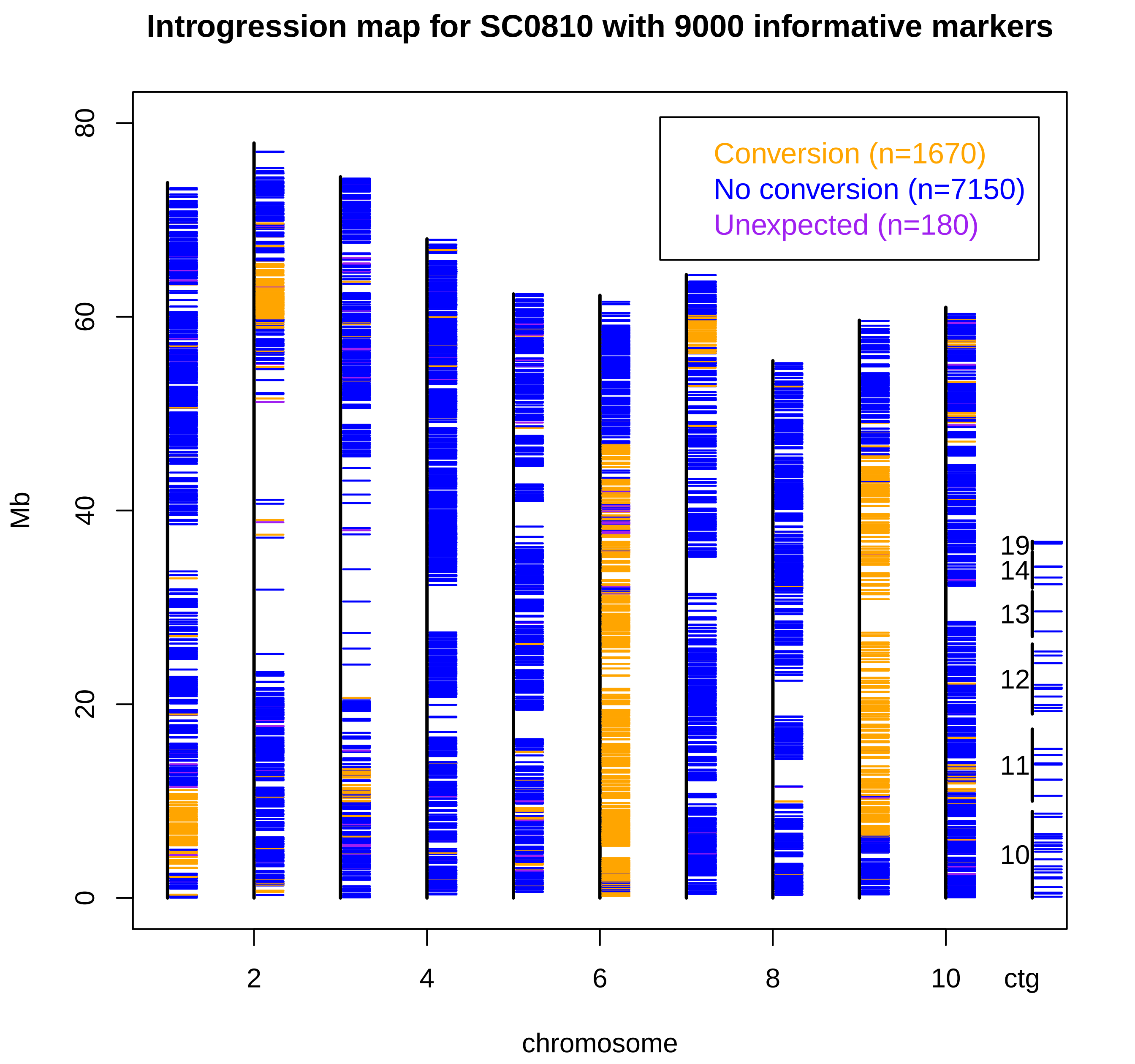
Introgression map for SC0810 with 9000 informative markers
Conversion (n=1670)
No conversion (n=7150)
Unexpected (n=180)
Mb
0
20
40
60
80
chromosome
2
4
6
8
10
ctg
19
14
13
12
11
10

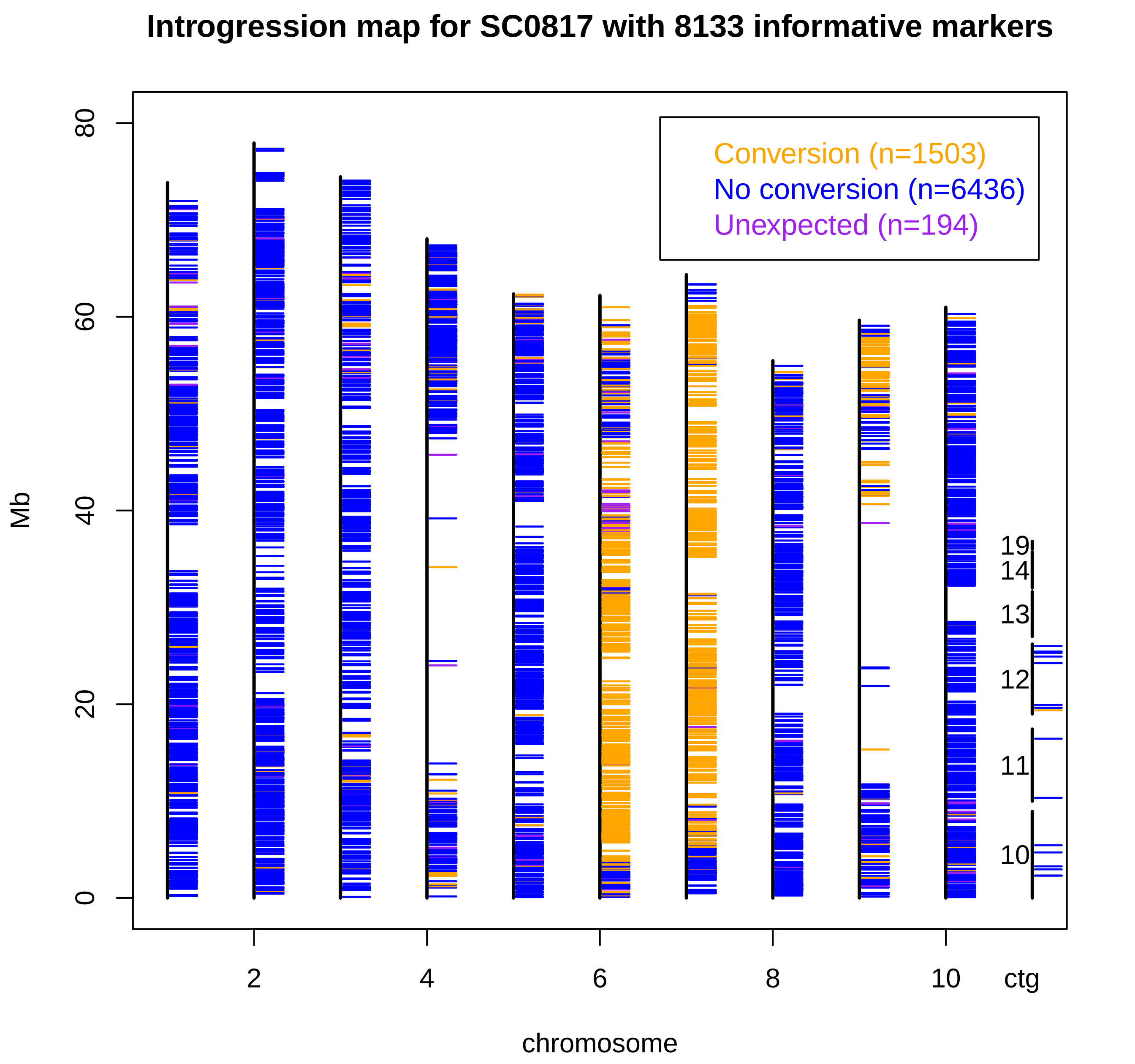
Introgression map for SC0817 with 8133 informative markers
Conversion (n=1503)
No conversion (n=6436)
Unexpected (n=194)
Mb
0
20
40
60
80
chromosome
2
4
6
8
10
ctg
19
14
13
12
11
10

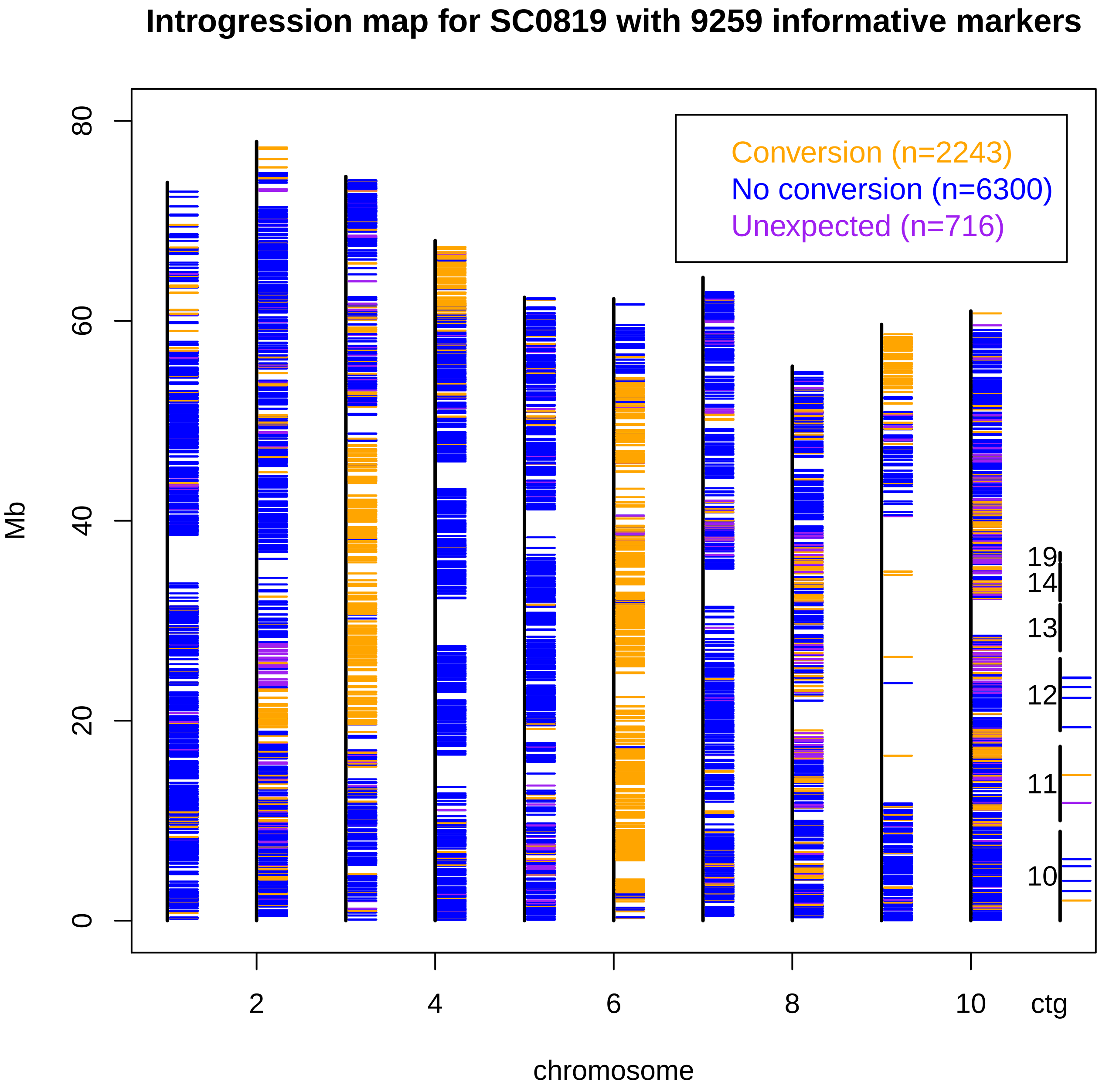
Introgression map for SC0819 with 9259 informative markers
Conversion (n=2243)
No conversion (n=6300)
Unexpected (n=716)
Mb
0
20
40
60
80
chromosome
2
4
6
8
10
ctg
19
14
13
12
11
10

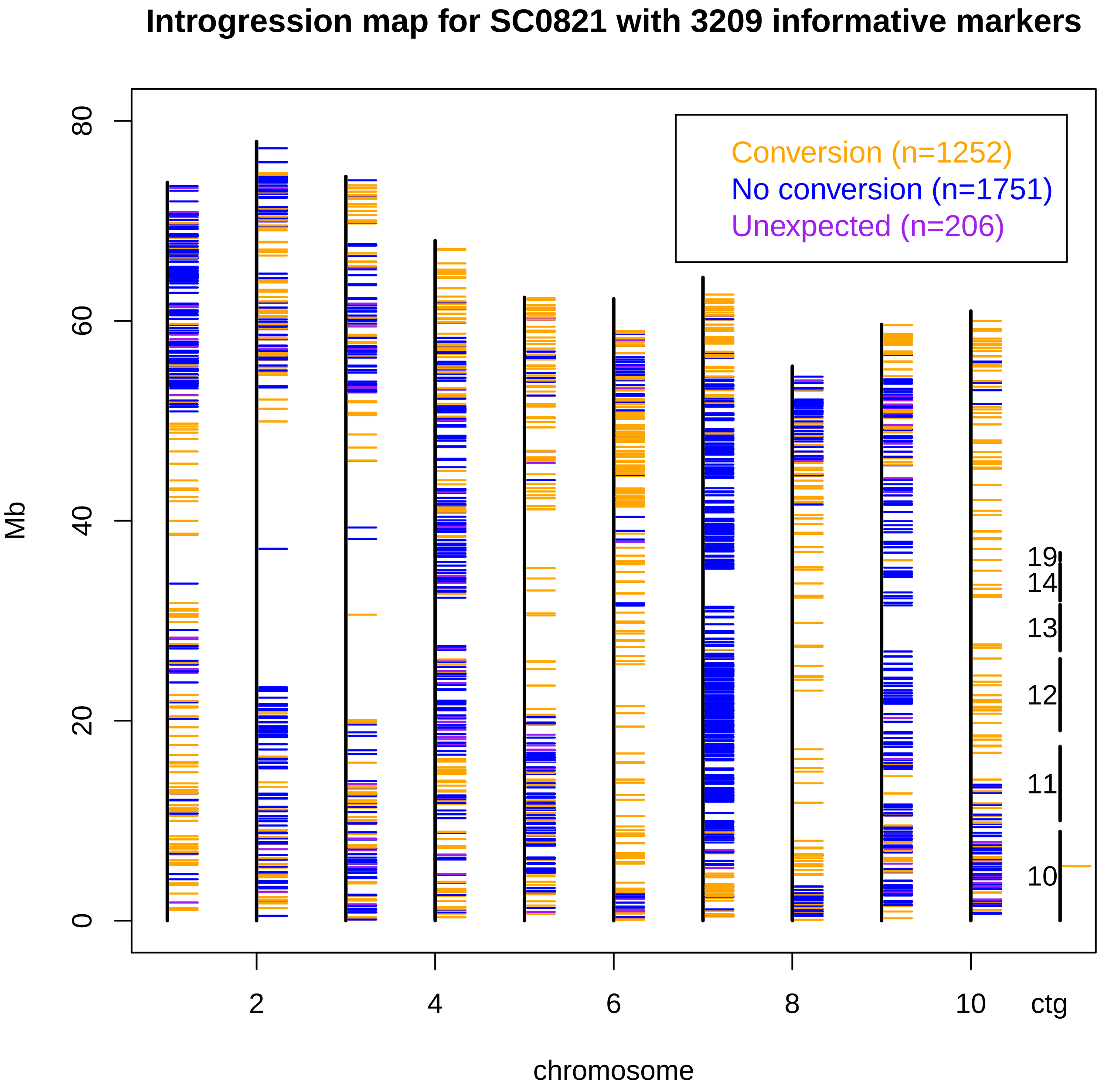
Introgression map for SC0821 with 3209 informative markers
Conversion (n=1252)
No conversion (n=1751)
Unexpected (n=206)
Mb
0
20
40
60
80
chromosome
2
4
6
8
10
ctg
19
14
13
12
11
10

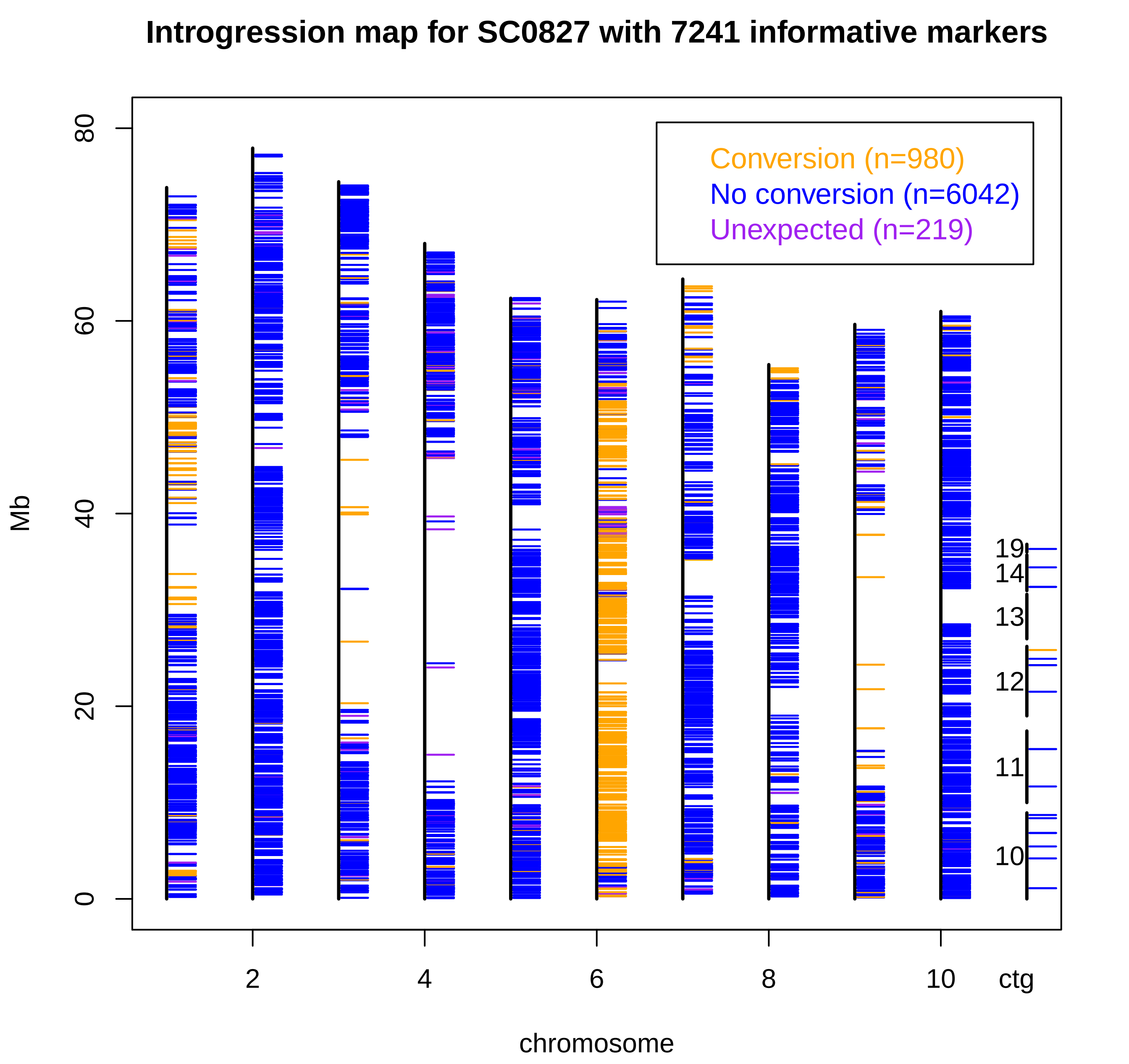
Introgression map for SC0827 with 7241 informative markers
Conversion (n=980)
No conversion (n=6042)
Unexpected (n=219)
Mb
0
20
40
60
80
2
4
6
8
10
ctg
19
14
13
12
11
10
chromosome

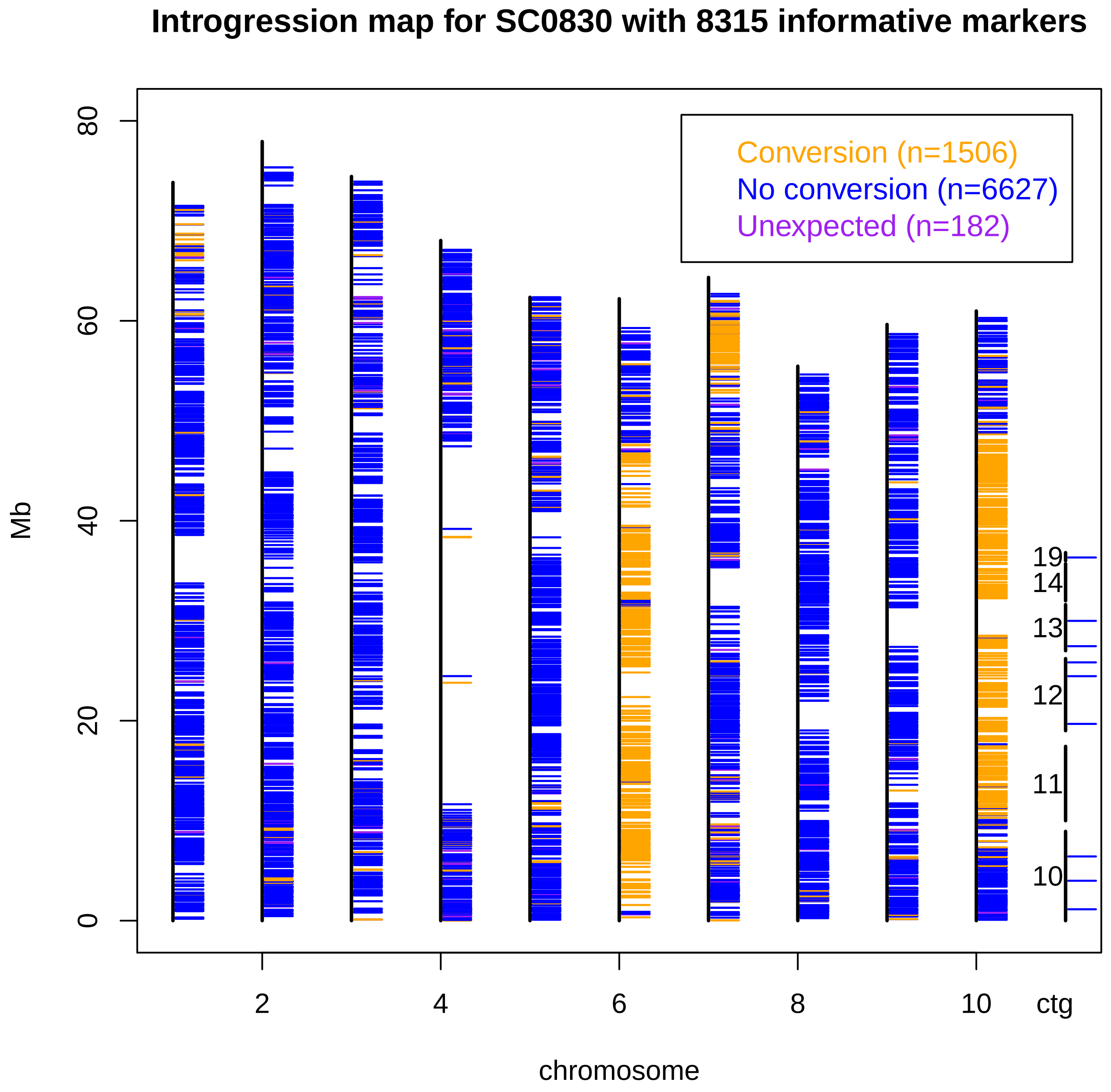

Introgression map for SC0830 with 8315 informative markers
Conversion (n=1506)
No conversion (n=6627)
Unexpected (n=182)
Mb
0
20
40
60
80
2
4
6
8
10
ctg
chromosome
19
14
13
12
11
10

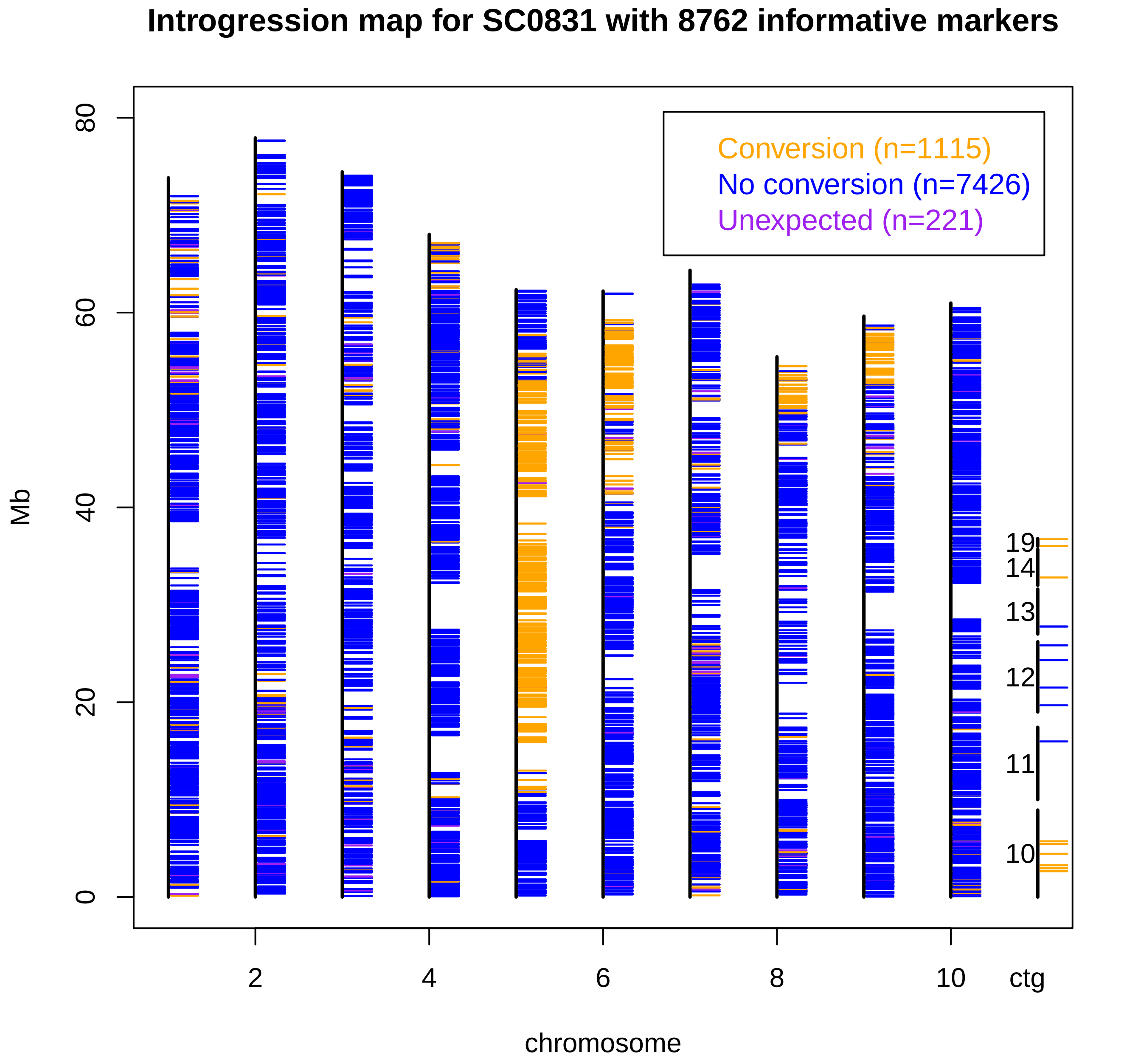

Introgression map for SC0831 with 8762 informative markers
Conversion (n=1115)
No conversion (n=7426)
Unexpected (n=221)
Mb
0
20
40
60
80
2
4
6
8
10
ctg
19
14
13
12
11
10
chromosome

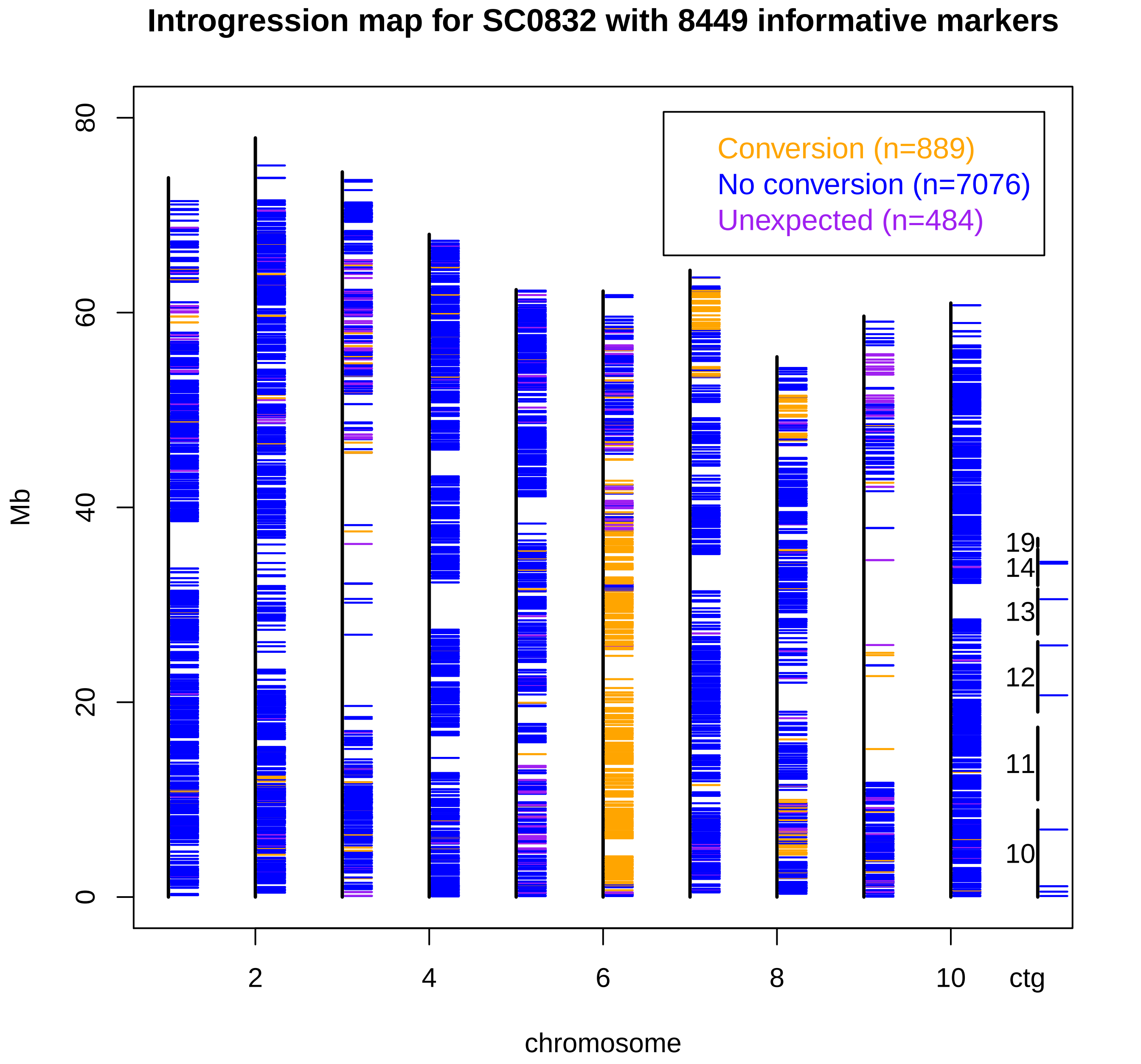
Introgression map for SC0832 with 8449 informative markers
Conversion (n=889)
No conversion (n=7076)
Unexpected (n=484)
Mb
0
20
40
60
80
chromosome
2
4
6
8
10
ctg
19
14
13
12
11
10

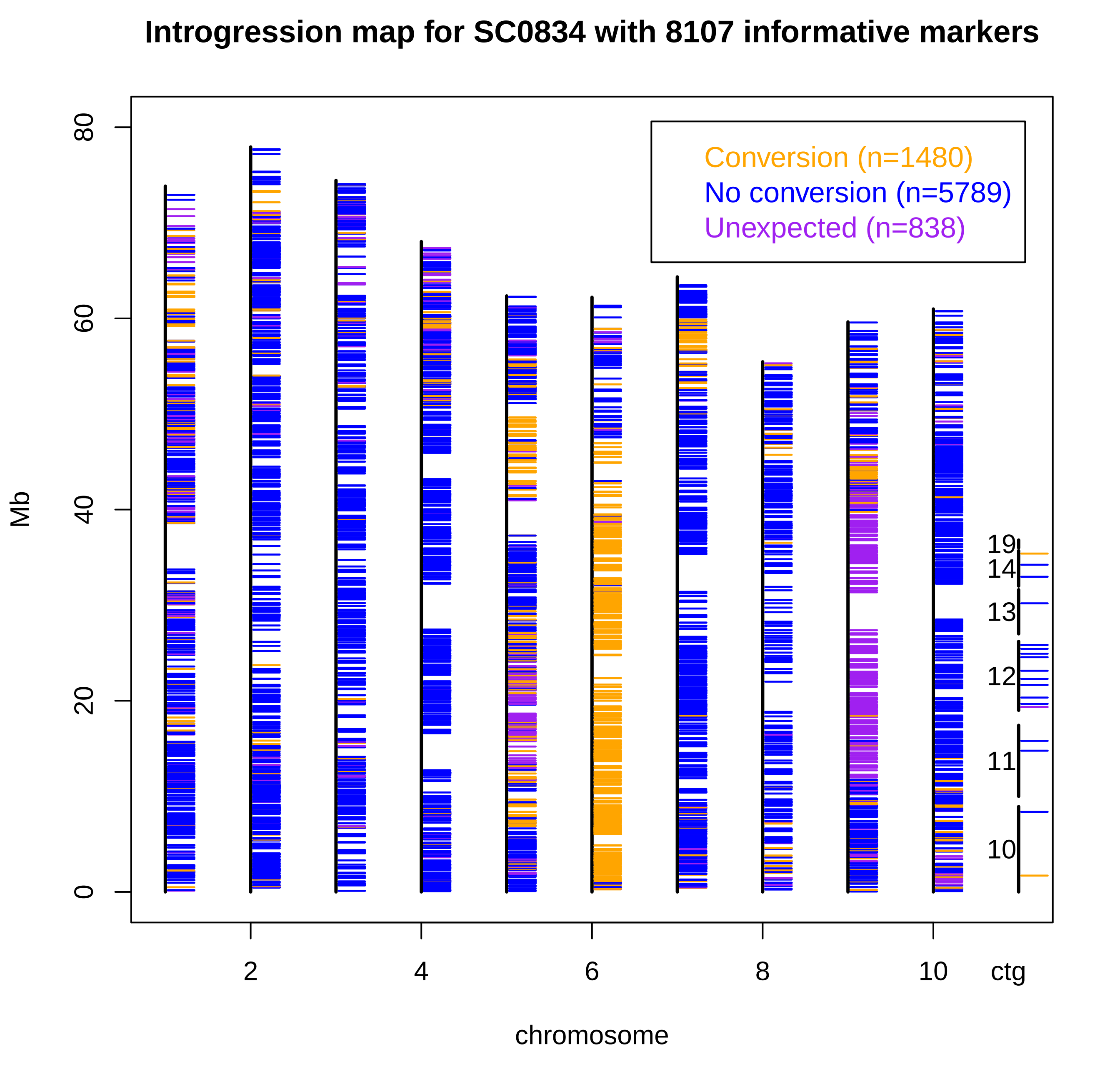
Introgression map for SC0834 with 8107 informative markers
Conversion (n=1480)
No conversion (n=5789)
Unexpected (n=838)
Mb
0
20
40
60
80
chromosome
2
4
6
8
10
ctg
19
14
13
12
11
10

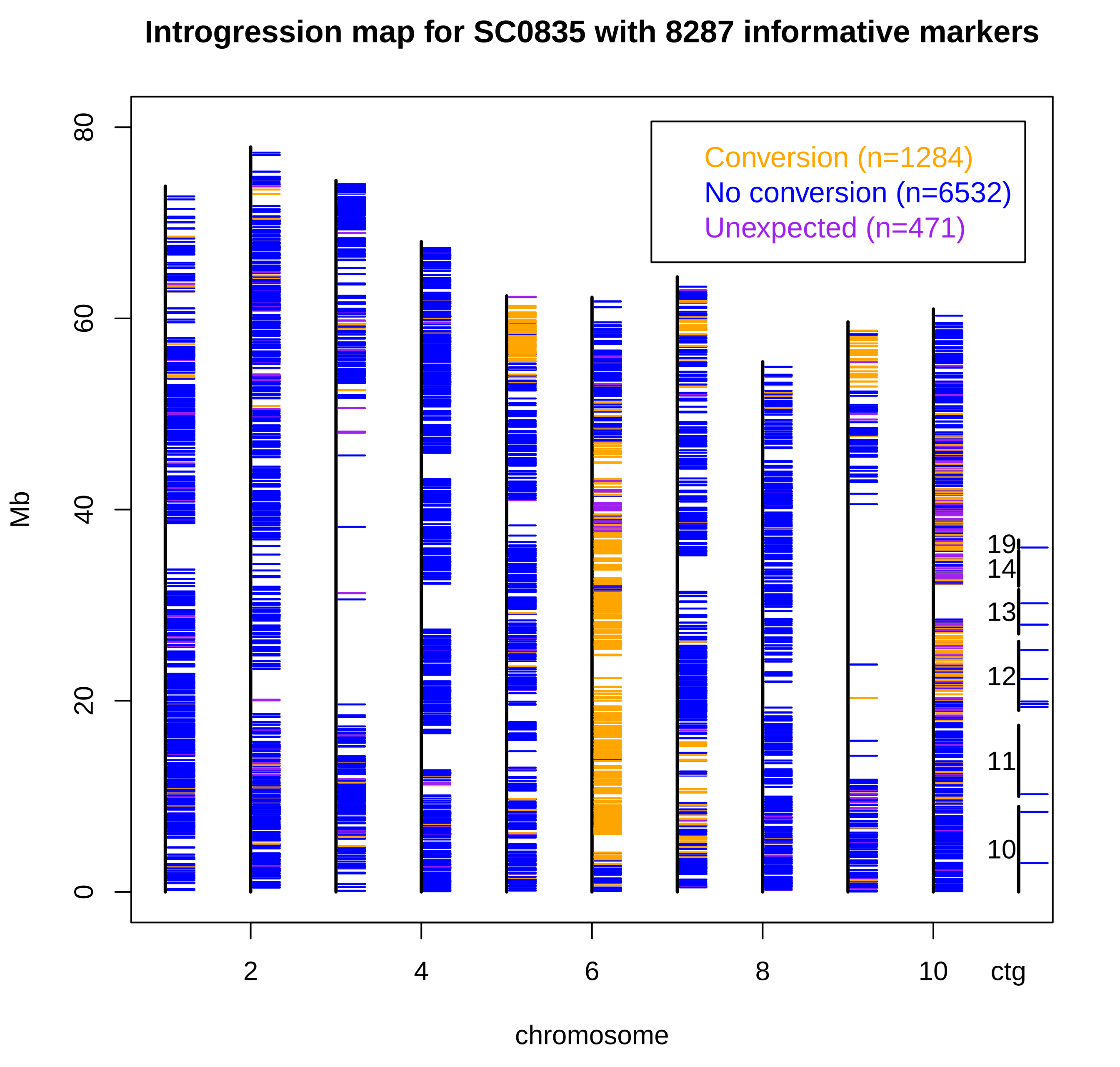

Introgression map for SC0835 with 8287 informative markers
Conversion (n=1284)
No conversion (n=6532)
Unexpected (n=471)
Mb
0
20
40
60
80
chromosome
2
4
6
8
10
ctg
19
14
13
12
11
10

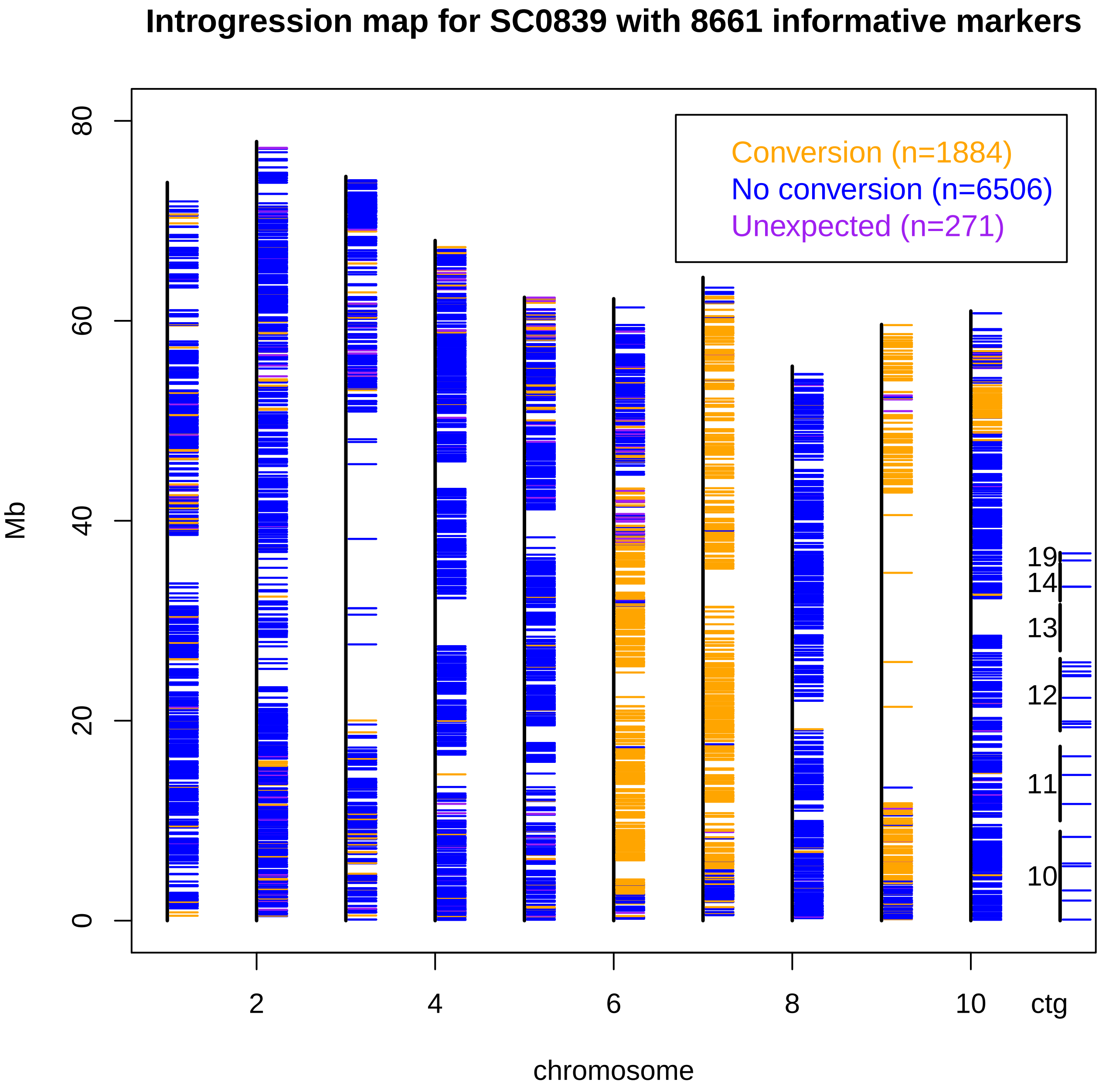
Introgression map for SC0839 with 8661 informative markers
Conversion (n=1884)
No conversion (n=6506)
Unexpected (n=271)
Mb
0
20
40
60
80
chromosome
2
4
6
8
10
ctg
19
14
13
12
11
10

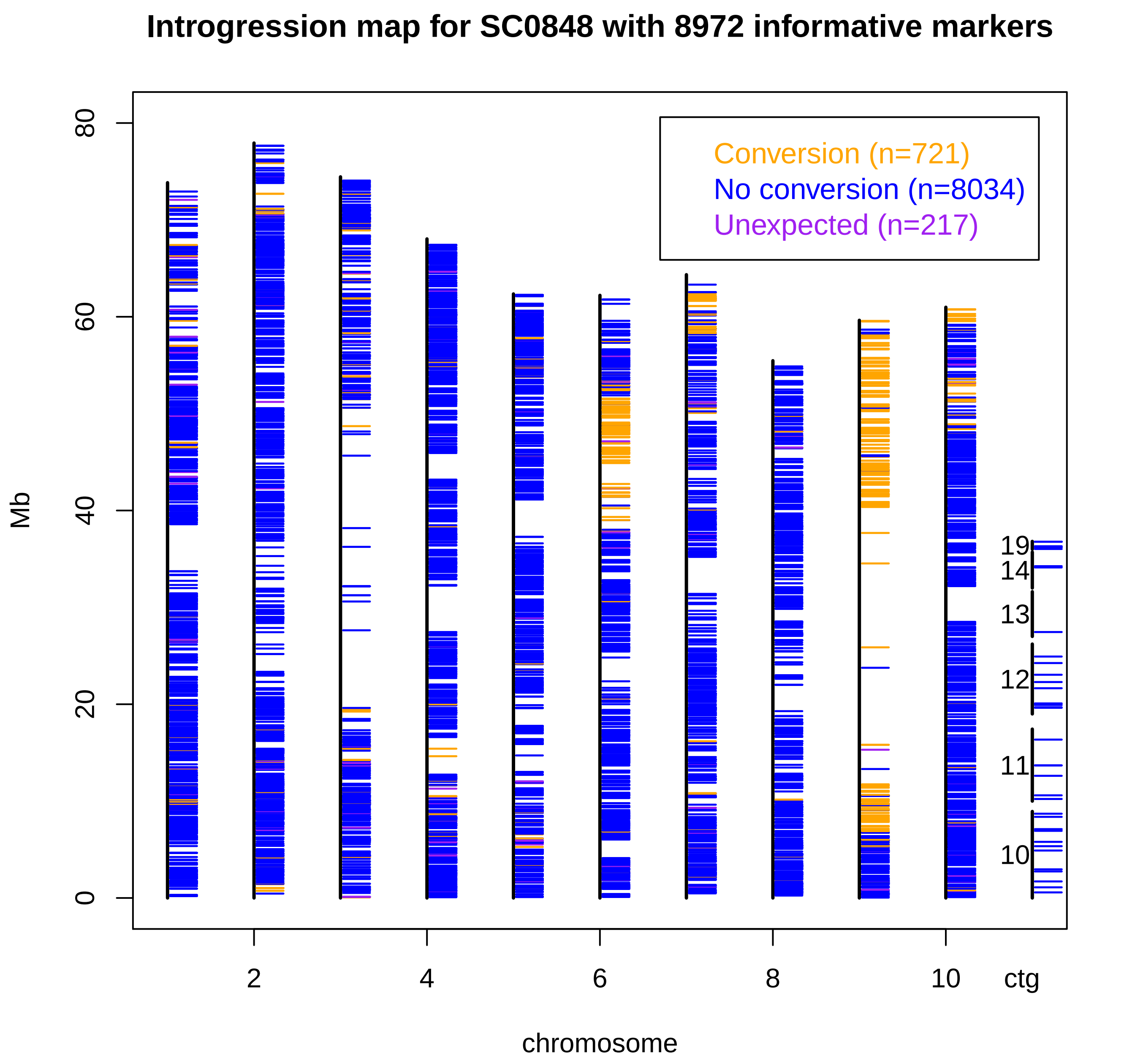
Introgression map for SC0848 with 8972 informative markers
Conversion (n=721)
No conversion (n=8034)
Unexpected (n=217)
Mb
0
20
40
60
80
chromosome
2
4
6
8
10
ctg
19
14
13
12
11
10

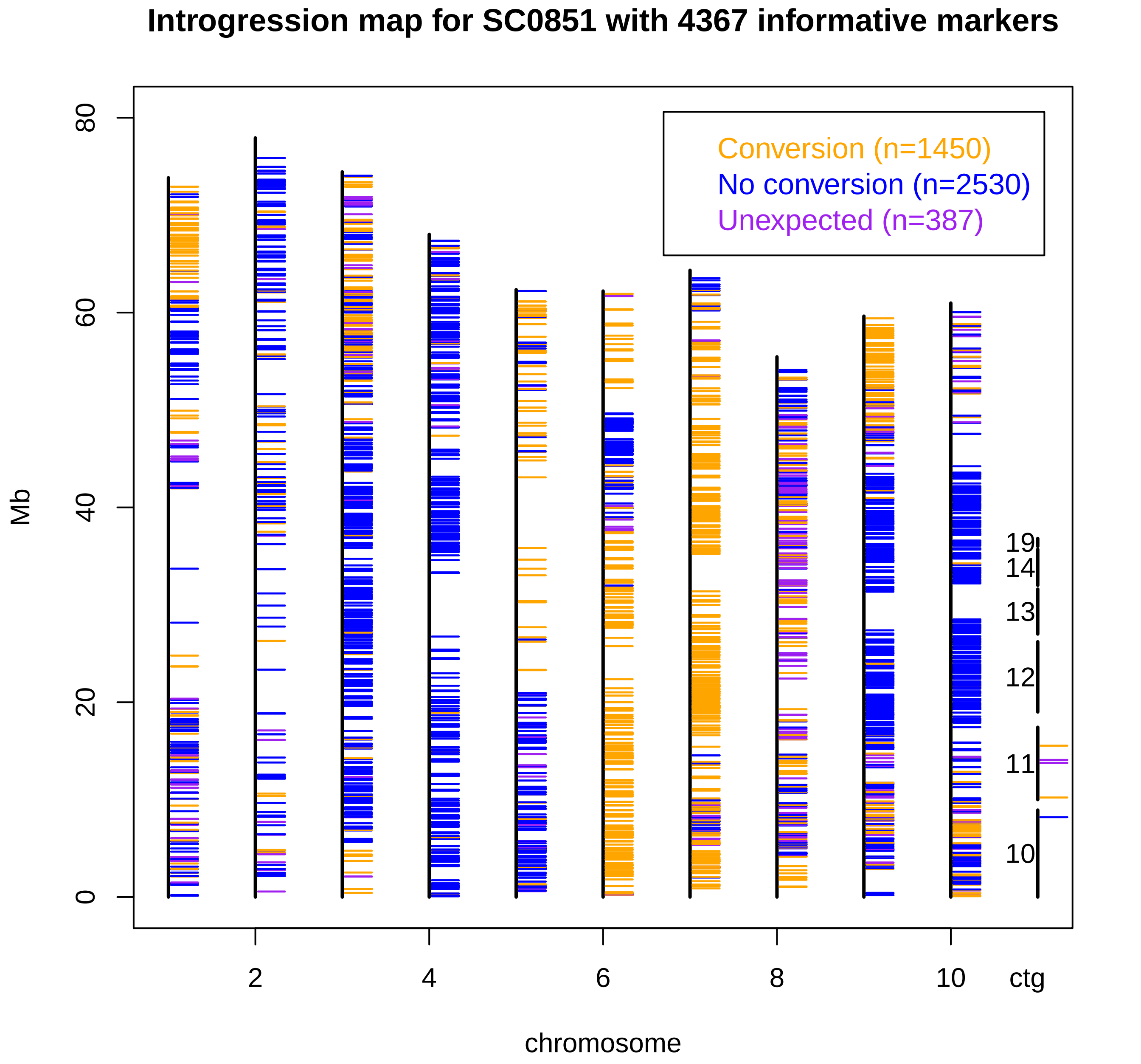
Introgression map for SC0851 with 4367 informative markers
Conversion (n=1450)
No conversion (n=2530)
Unexpected (n=387)
Mb
0
20
40
60
80
chromosome
2
4
6
8
10
ctg
19
14
13
12
11
10

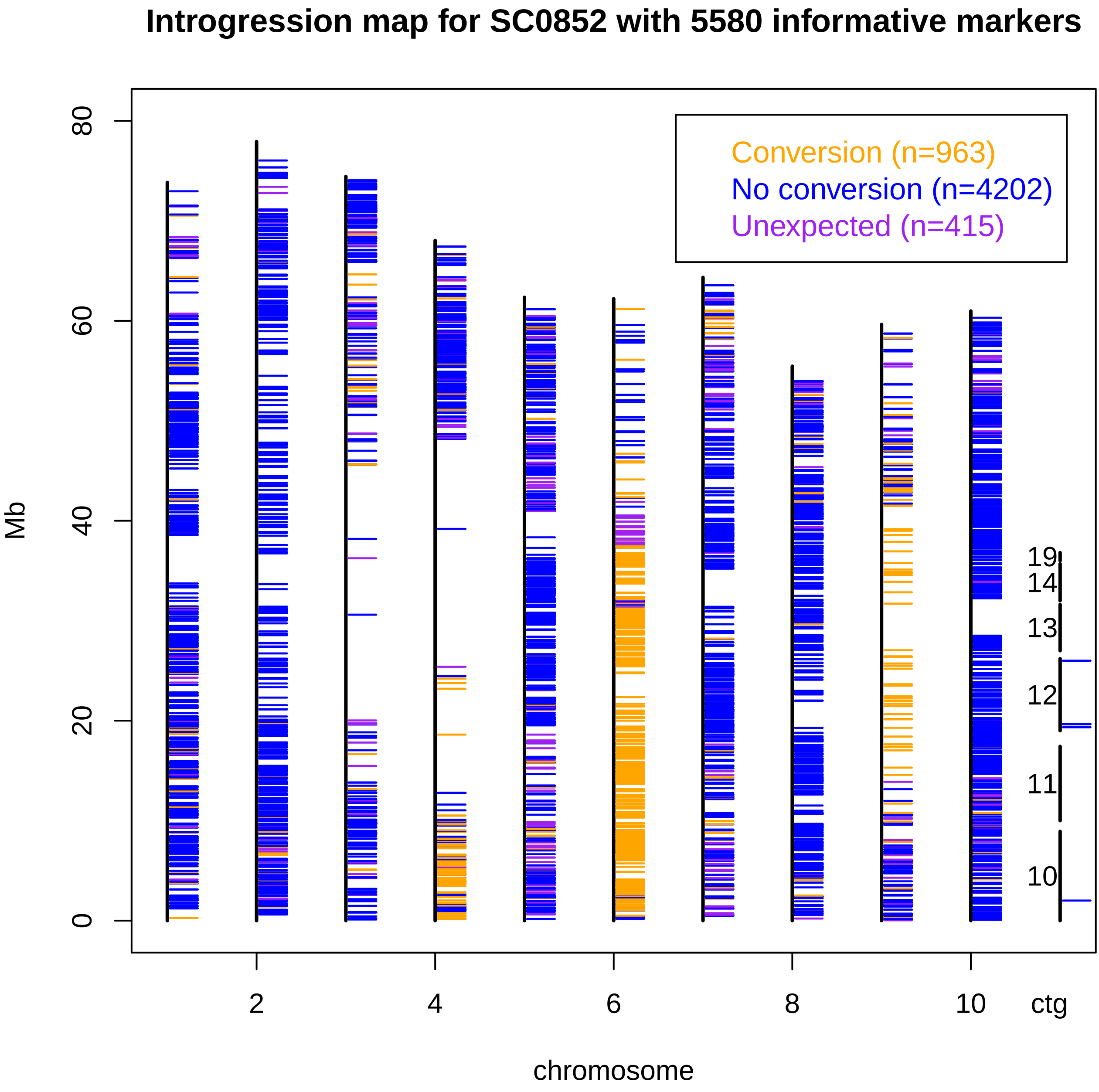

Introgression map for SC0852 with 5580 informative markers
Conversion (n=963)
No conversion (n=4202)
Unexpected (n=415)
Mb
0
20
40
60
80
chromosome
2
4
6
8
10
ctg
19
14
13
12
11
10

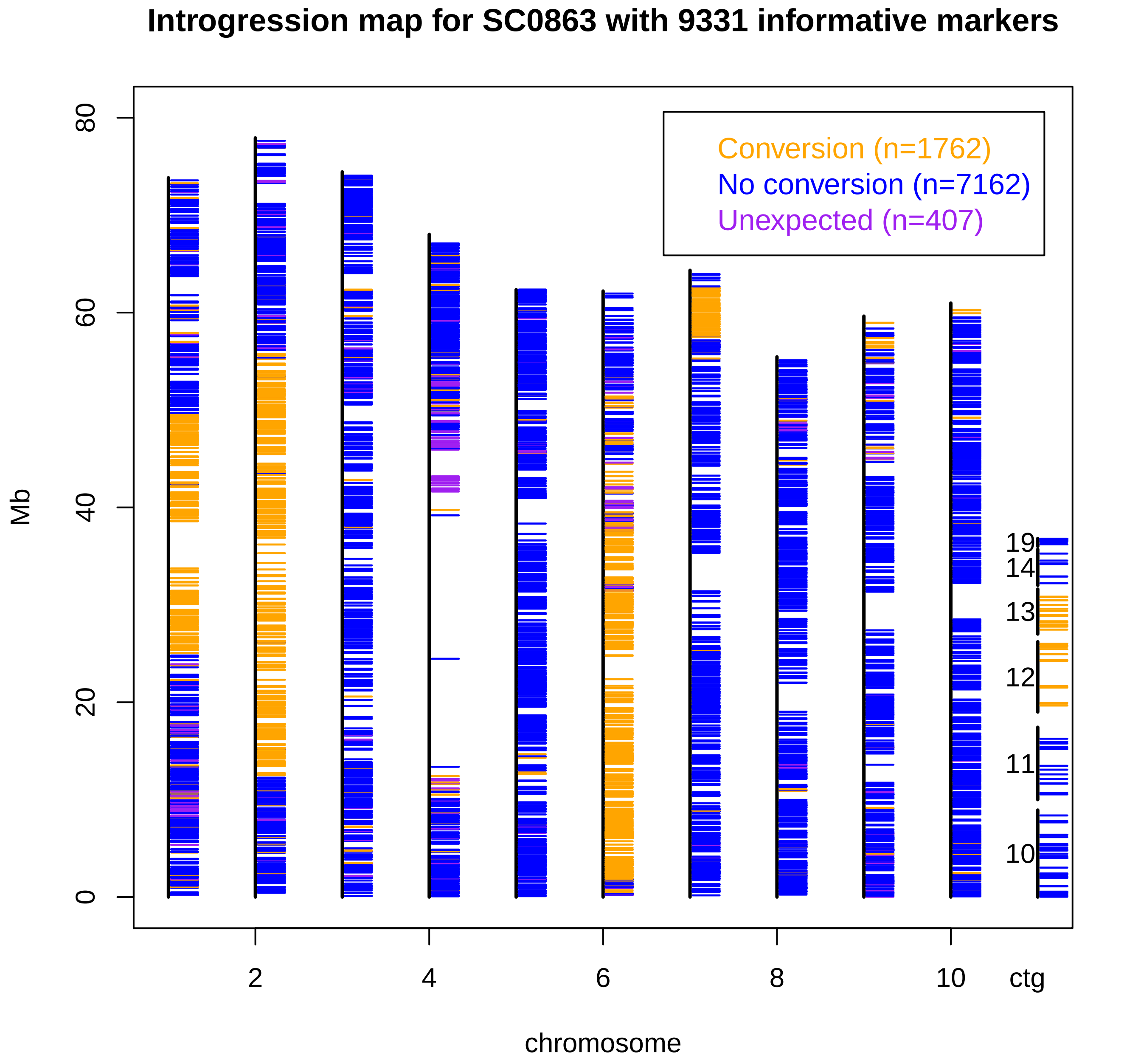

Introgression map for SC0863 with 9331 informative markers
Conversion (n=1762)
No conversion (n=7162)
Unexpected (n=407)
Mb
0
20
40
60
80
chromosome
2
4
6
8
10
ctg
19
14
13
12
11
10

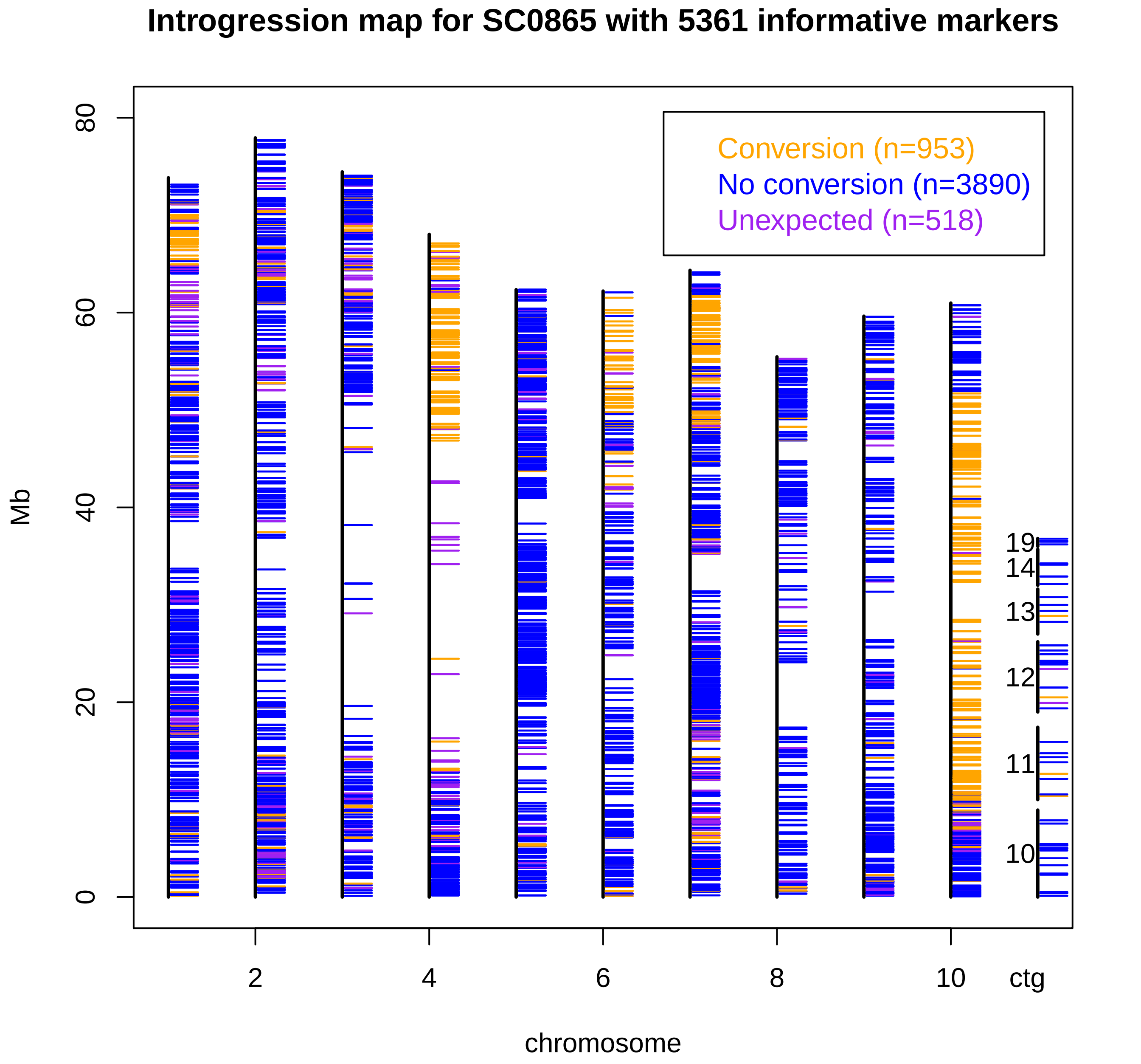

Introgression map for SC0865 with 5361 informative markers
Conversion (n=953)
No conversion (n=3890)
Unexpected (n=518)
Mb
0
20
40
60
80
chromosome
2
4
6
8
10
ctg
19
14
13
12
11
10

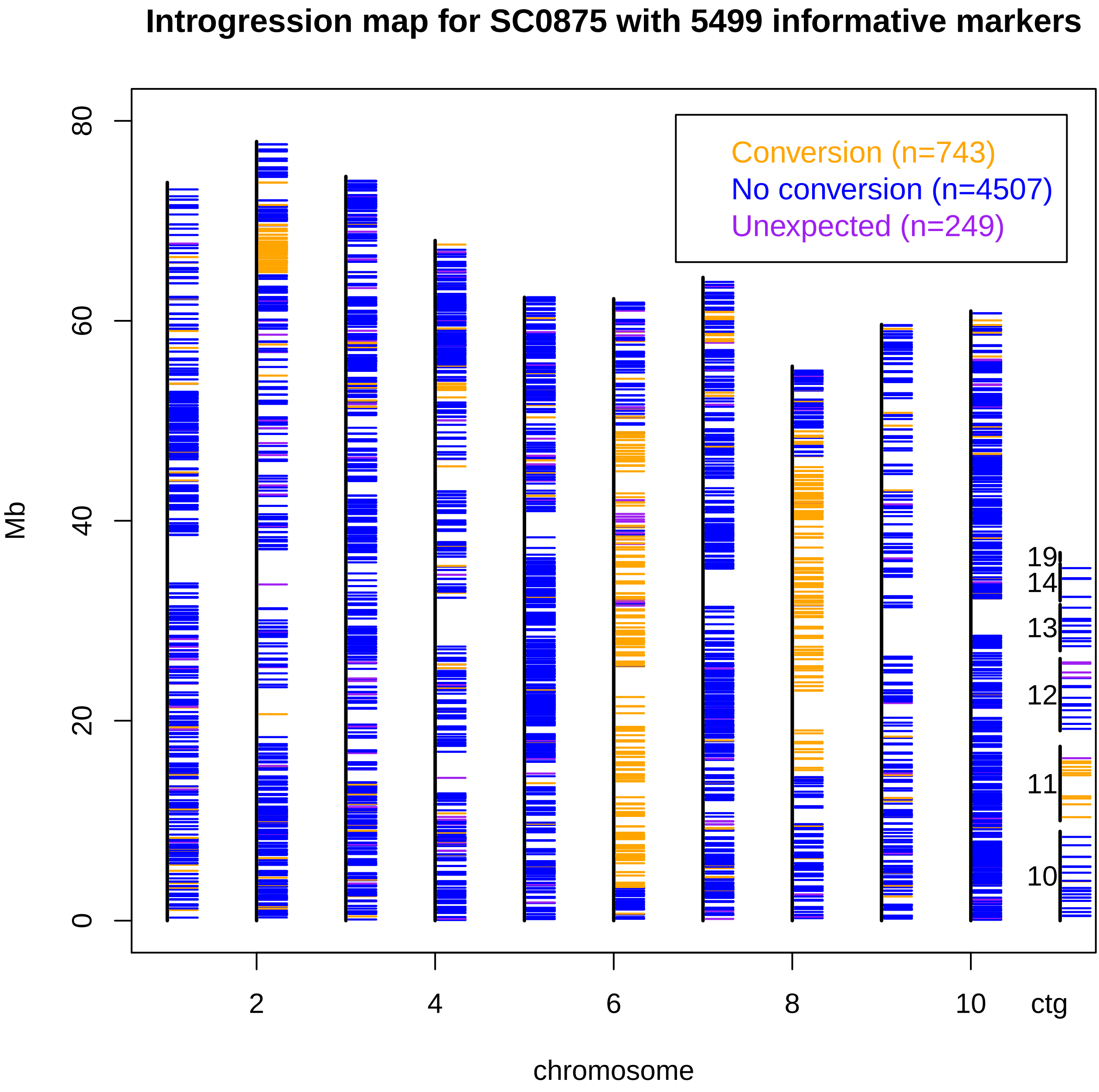
Introgression map for SC0875 with 5499 informative markers
Conversion (n=743)
No conversion (n=4507)
Unexpected (n=249)
Mb
0
20
40
60
80
chromosome
2
4
6
8
10
ctg
19
14
13
12
11
10

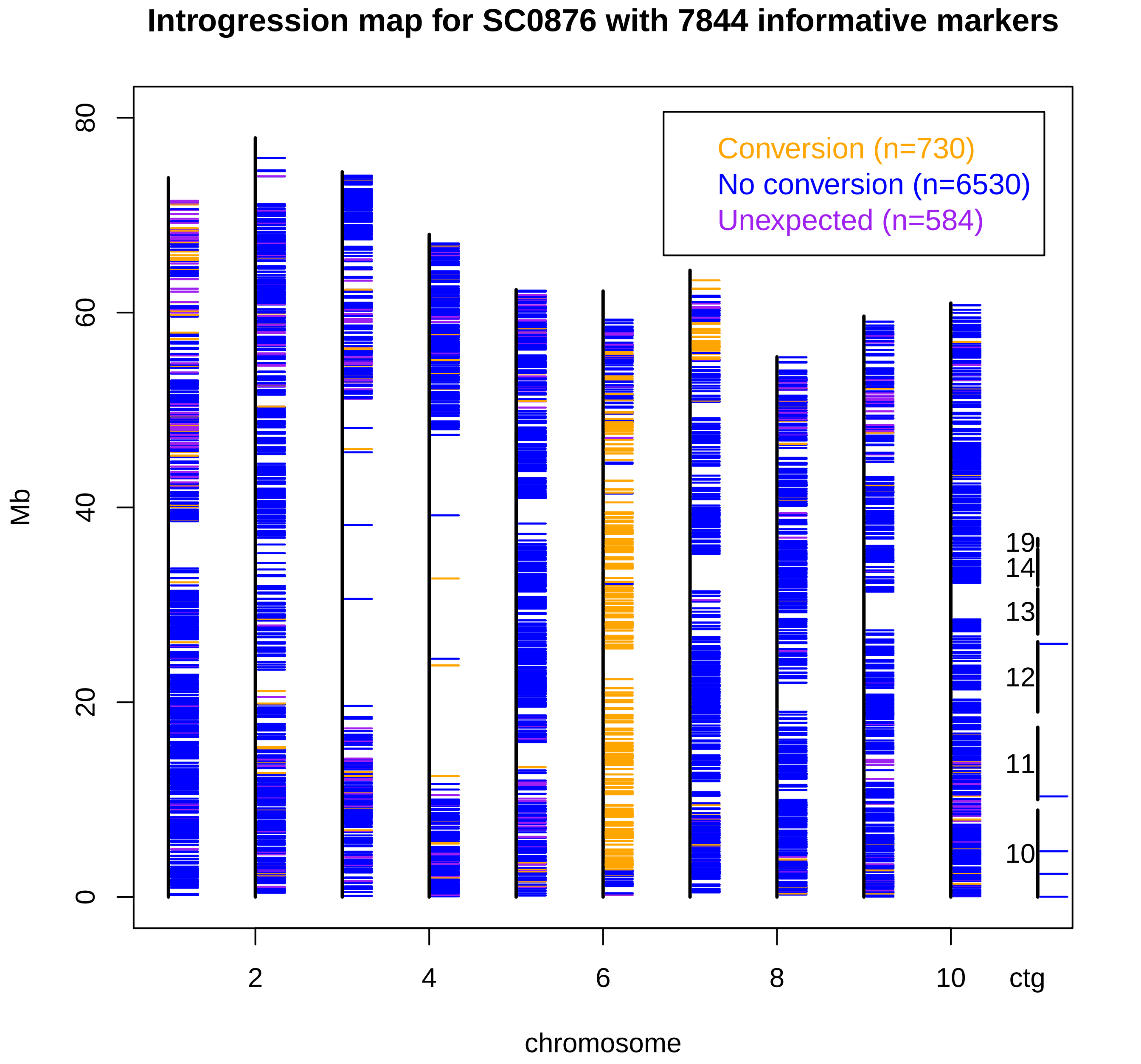
Introgression map for SC0876 with 7844 informative markers
Conversion (n=730)
No conversion (n=6530)
Unexpected (n=584)
Mb
0
20
40
60
80
2
4
6
8
10
ctg
19
14
13
12
11
10
chromosome

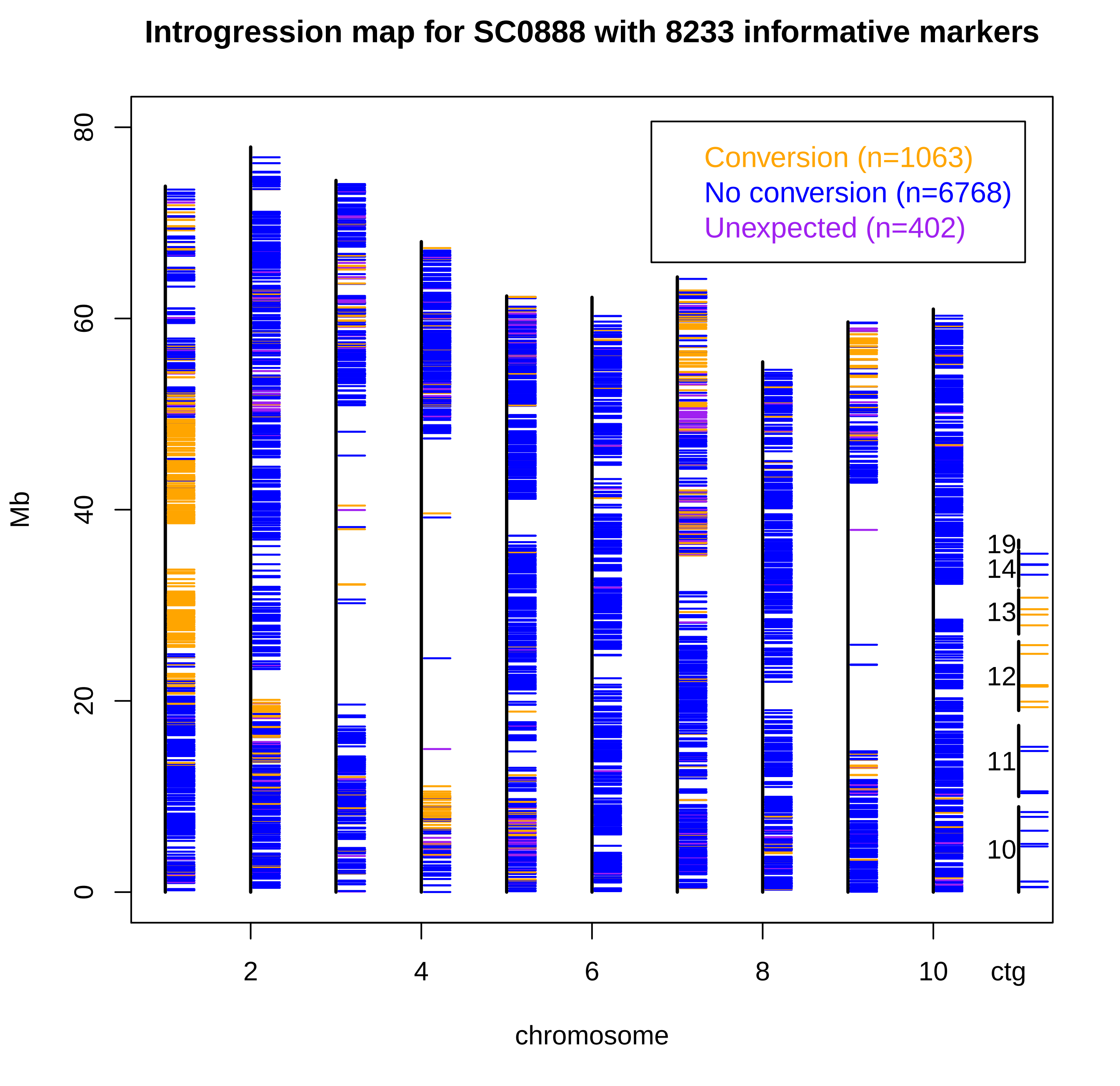
Introgression map for SC0888 with 8233 informative markers
Conversion (n=1063)
No conversion (n=6768)
Unexpected (n=402)
Mb
0
20
40
60
80
chromosome
2
4
6
8
10
ctg
19
14
13
12
11
10

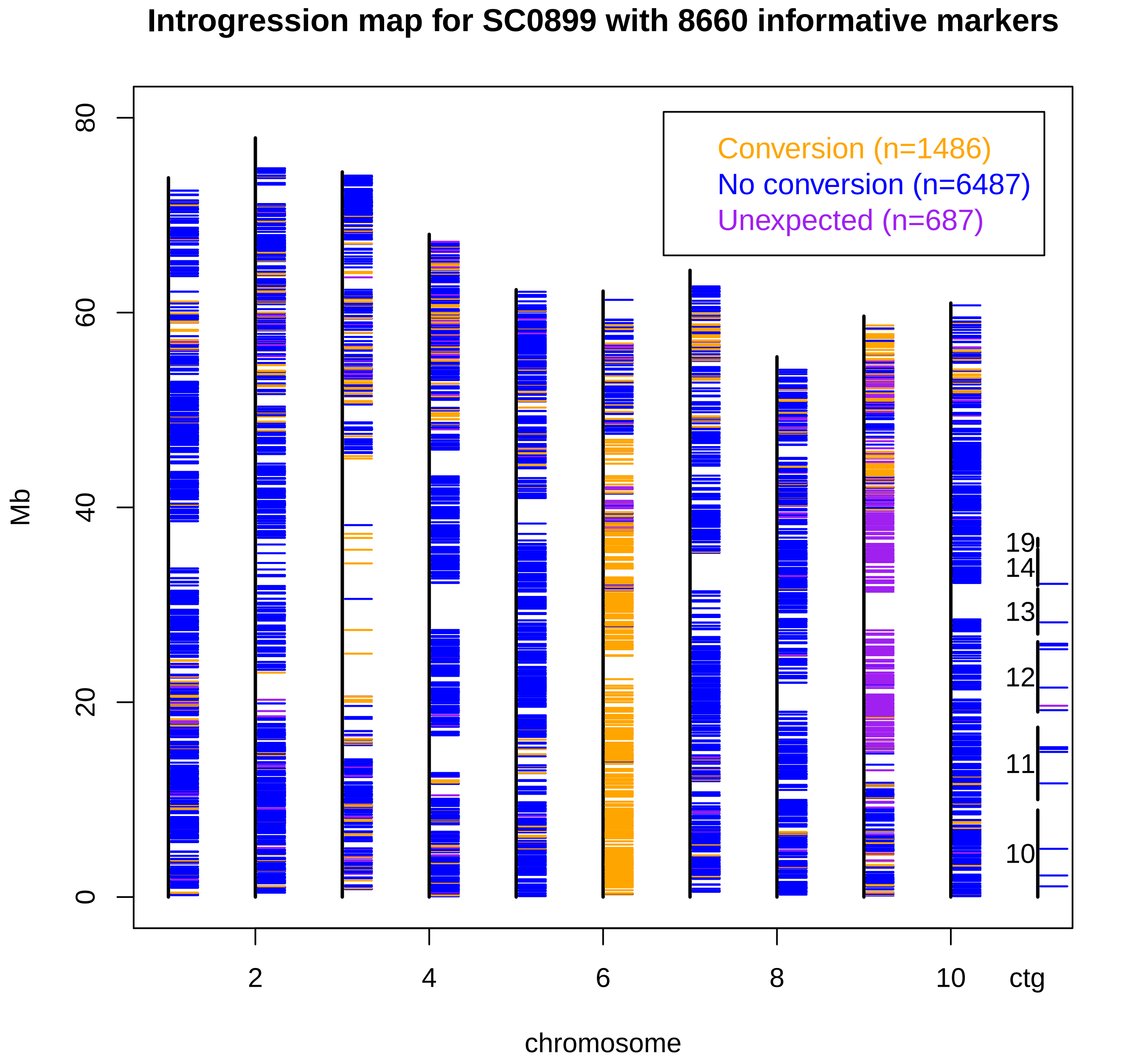
Introgression map for SC0899 with 8660 informative markers
Conversion (n=1486)
No conversion (n=6487)
Unexpected (n=687)
Mb
0
20
40
60
80
2
4
6
8
10
ctg
19
14
13
12
11
10
chromosome

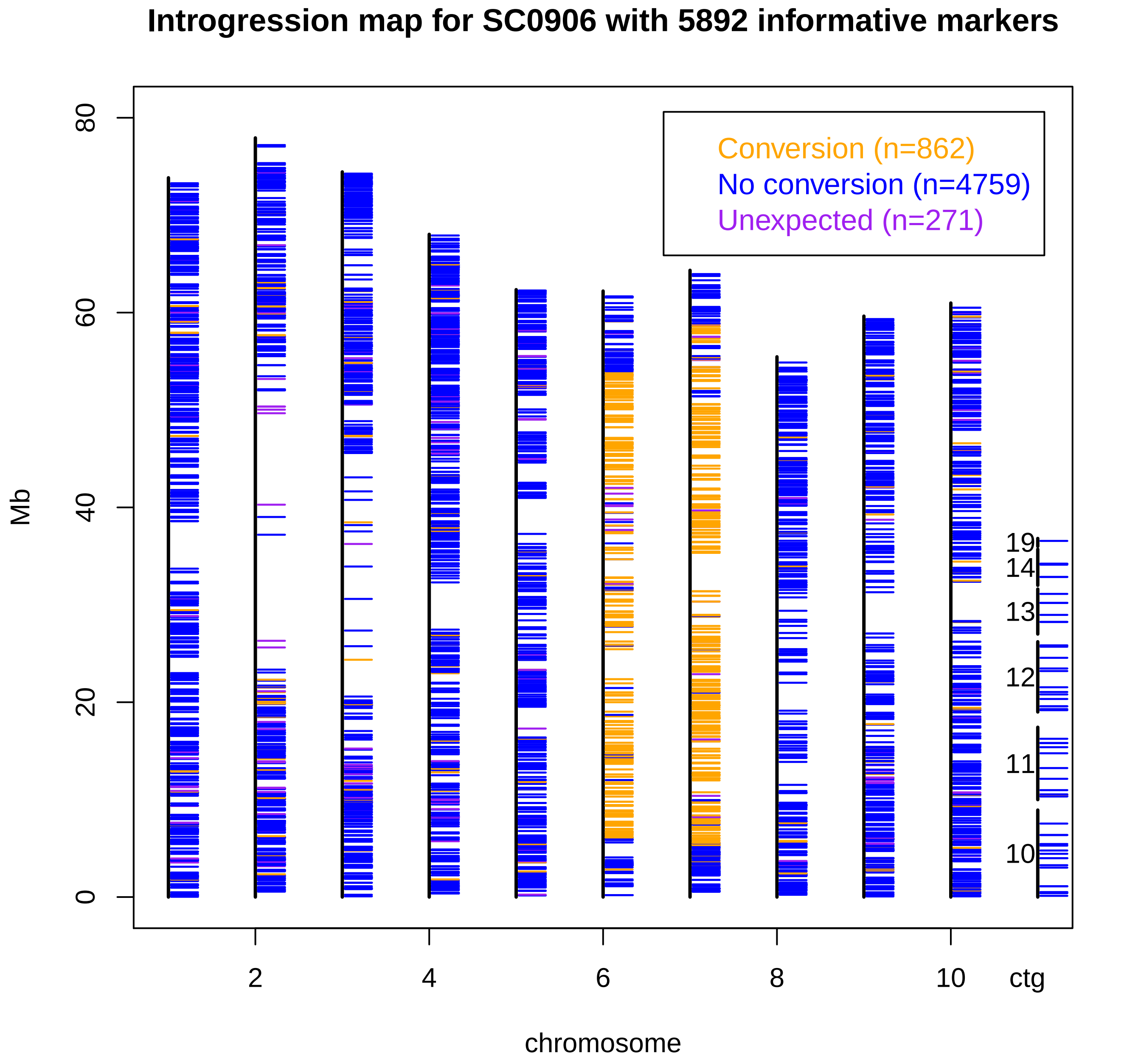
Introgression map for SC0906 with 5892 informative markers
Conversion (n=862)
No conversion (n=4759)
Unexpected (n=271)
Mb
0
20
40
60
80
chromosome
2
4
6
8
10
ctg
19
14
13
12
11
10

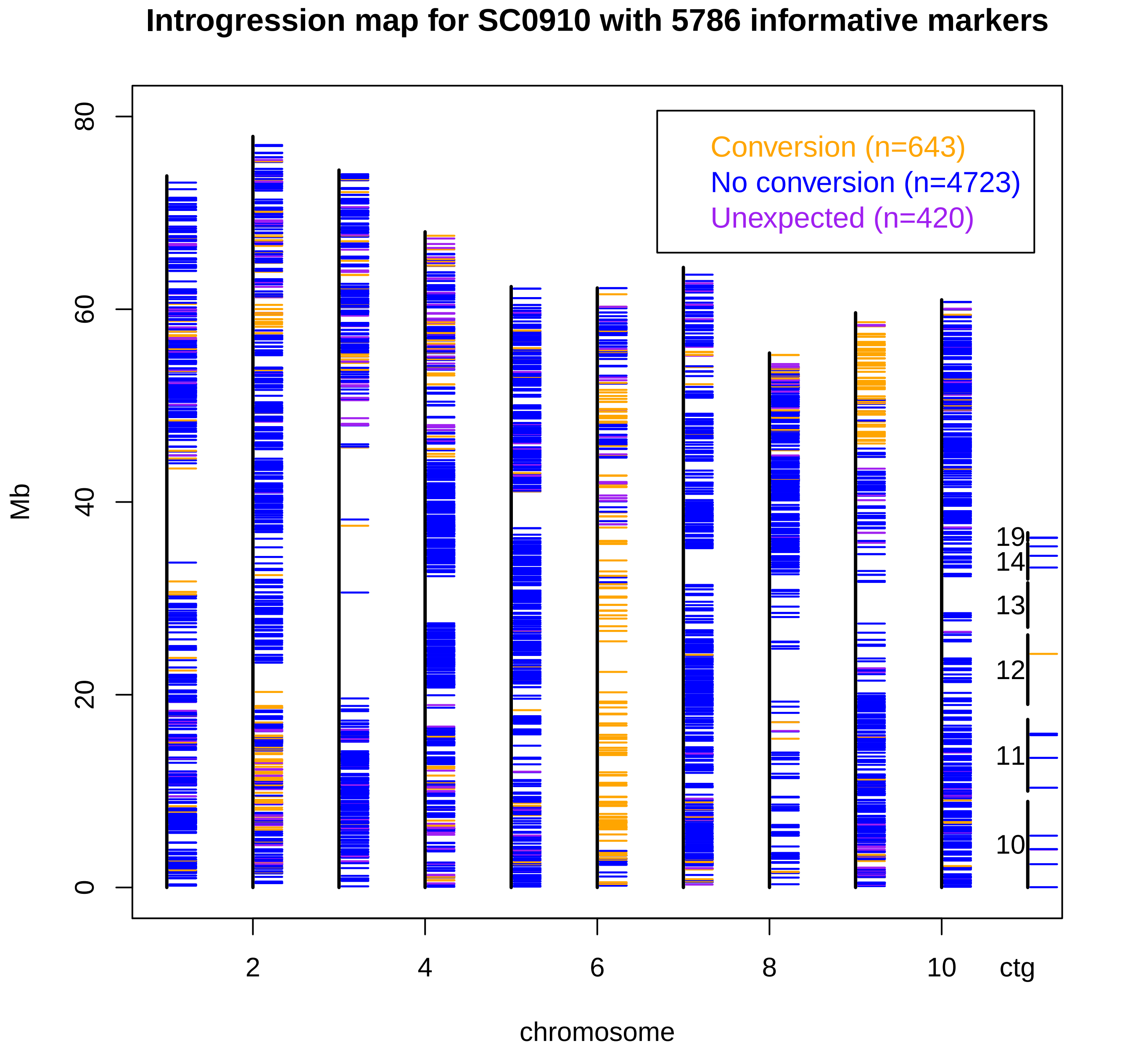
Introgression map for SC0910 with 5786 informative markers
Conversion (n=643)
No conversion (n=4723)
Unexpected (n=420)
Mb
0
20
40
60
80
2
4
6
8
10
ctg
19
14
13
12
11
10
chromosome

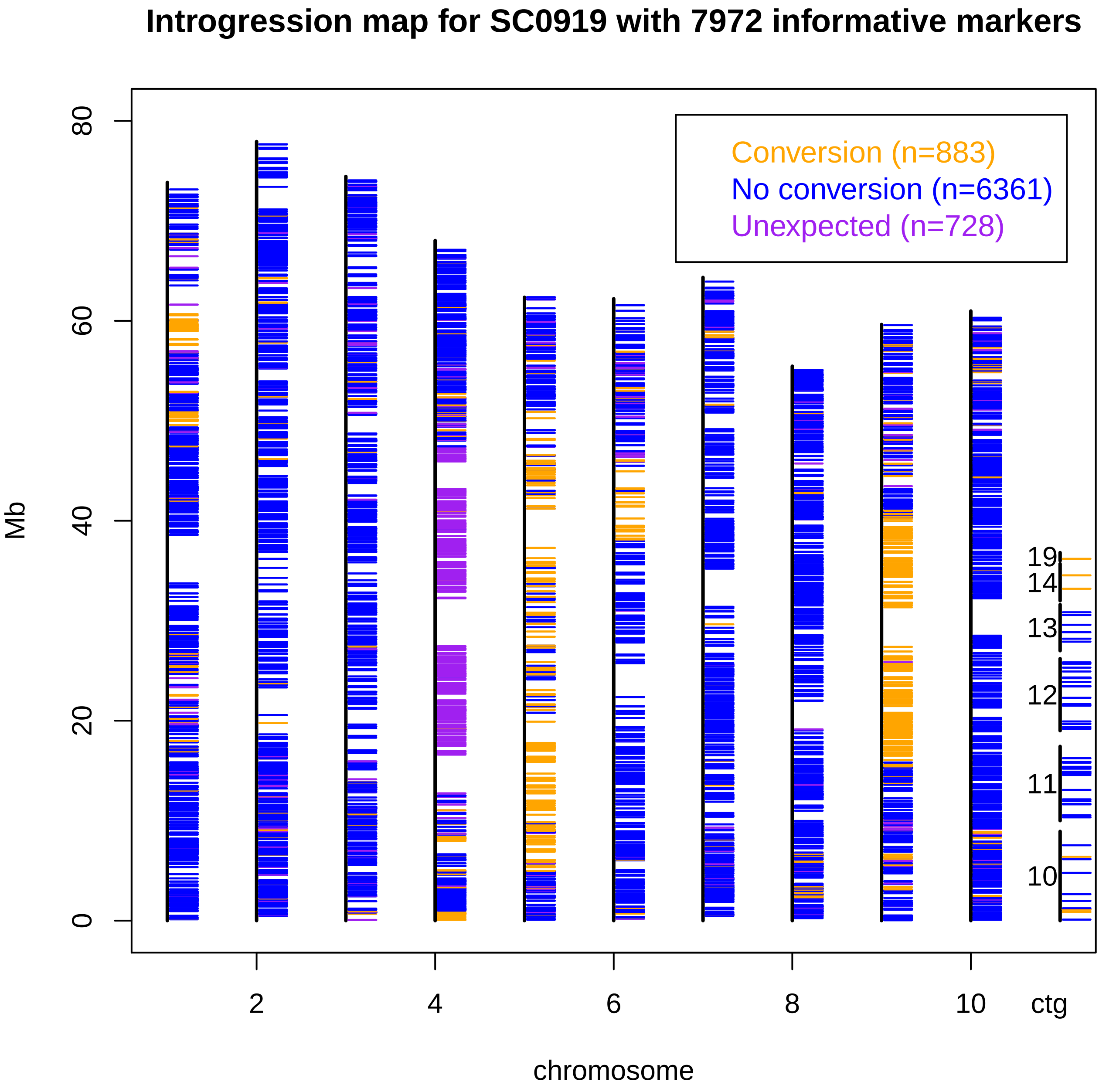
Introgression map for SC0919 with 7972 informative markers
Conversion (n=883)
No conversion (n=6361)
Unexpected (n=728)
Mb
0
20
40
60
80
chromosome
2
4
6
8
10
ctg
19
14
13
12
11
10

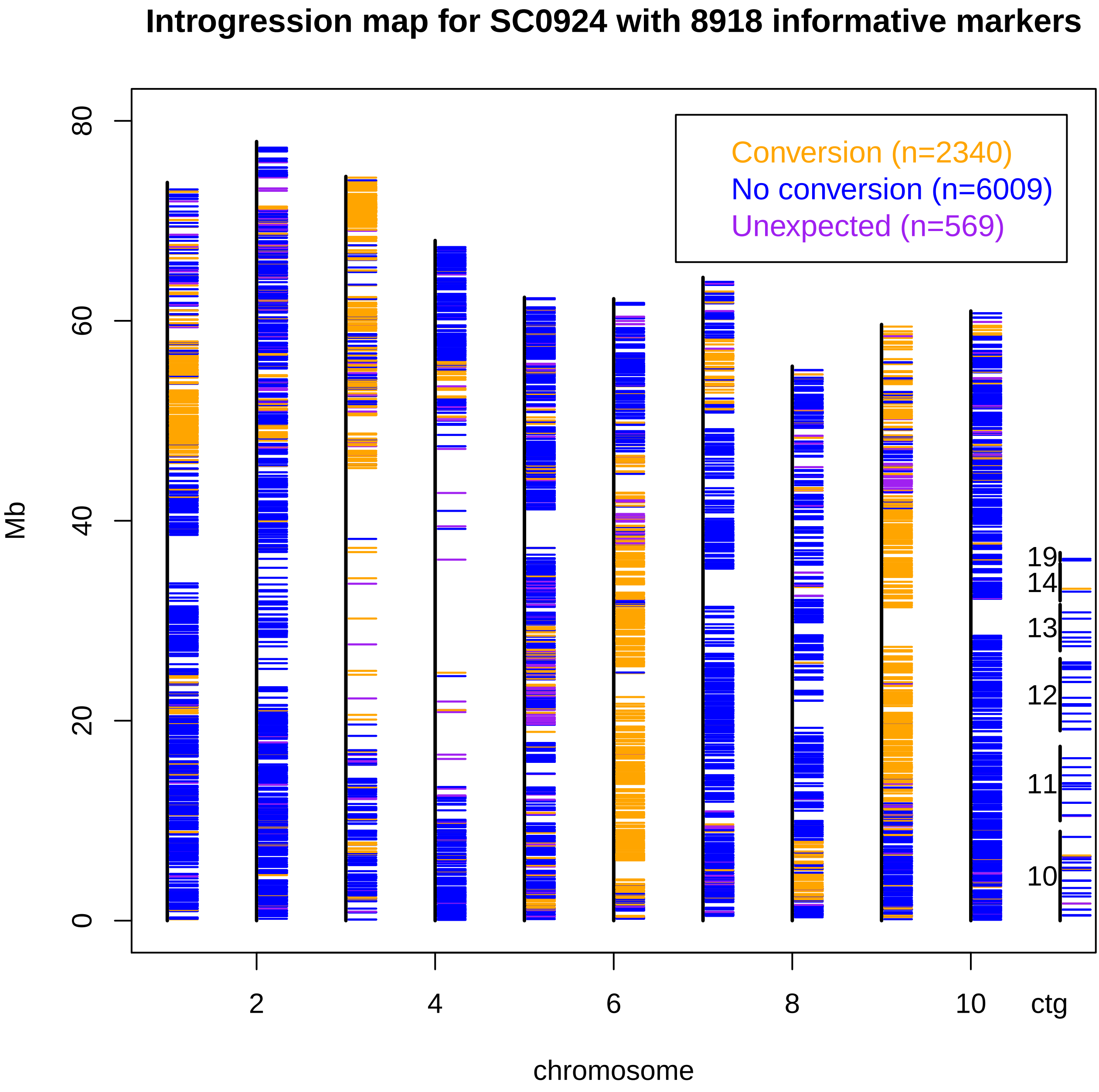

Introgression map for SC0924 with 8918 informative markers
Conversion (n=2340)
No conversion (n=6009)
Unexpected (n=569)
Mb
0
20
40
60
80
chromosome
2
4
6
8
10
ctg
19
14
13
12
11
10

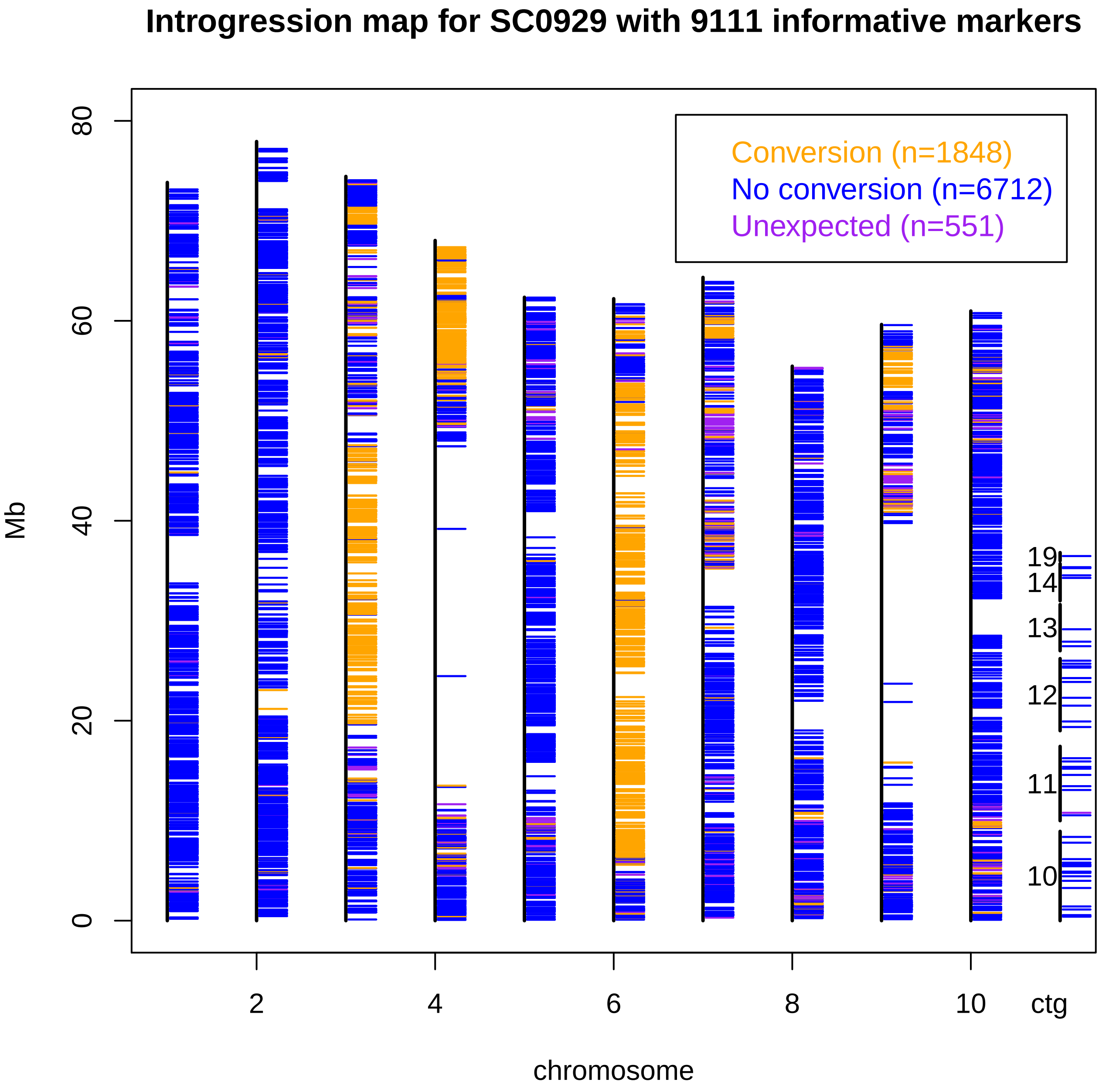

Introgression map for SC0929 with 9111 informative markers
Conversion (n=1848)
No conversion (n=6712)
Unexpected (n=551)
Mb
0
20
40
60
80
chromosome
2
4
6
8
10
ctg
19
14
13
12
11
10

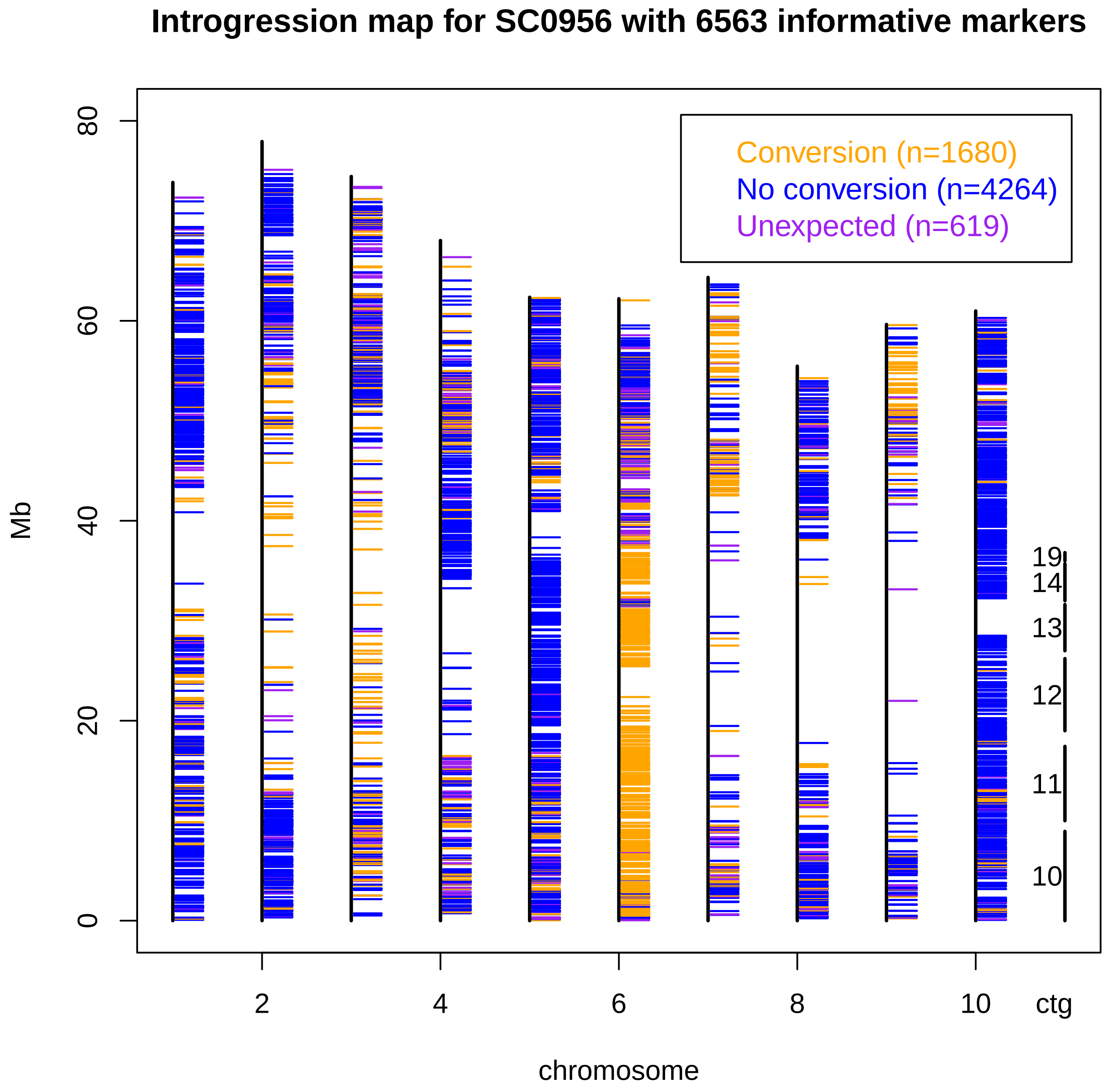

Introgression map for SC0956 with 6563 informative markers
Conversion (n=1680)
No conversion (n=4264)
Unexpected (n=619)
Mb
0
20
40
60
80
2
4
6
8
10
ctg
19
14
13
12
11
10
chromosome

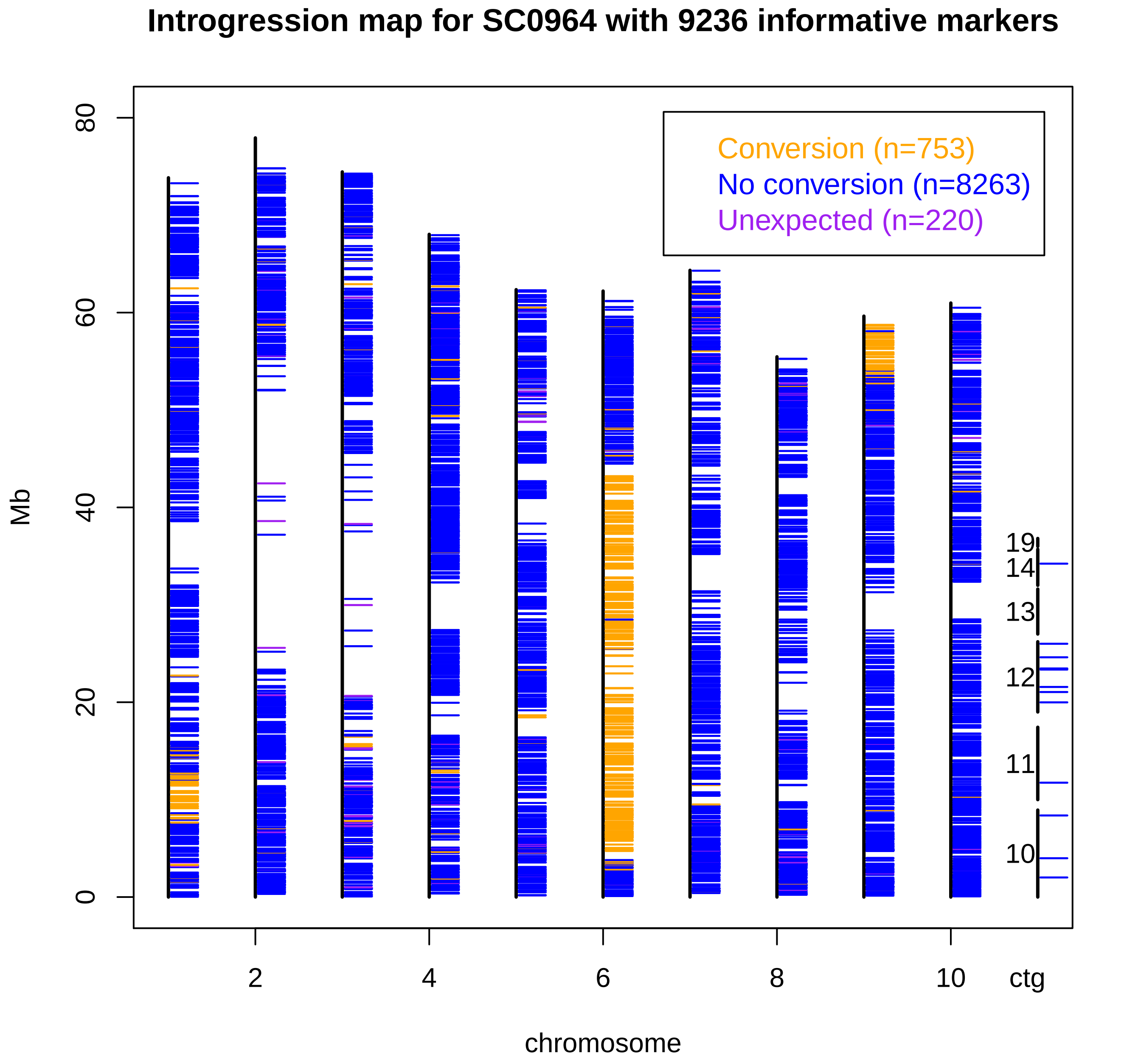

Introgression map for SC0964 with 9236 informative markers
Conversion (n=753)
No conversion (n=8263)
Unexpected (n=220)
Mb
0
20
40
60
80
2
4
6
8
10
ctg
19
14
13
12
11
10
chromosome

Introgression map for SC0970 with 7607 informative markers

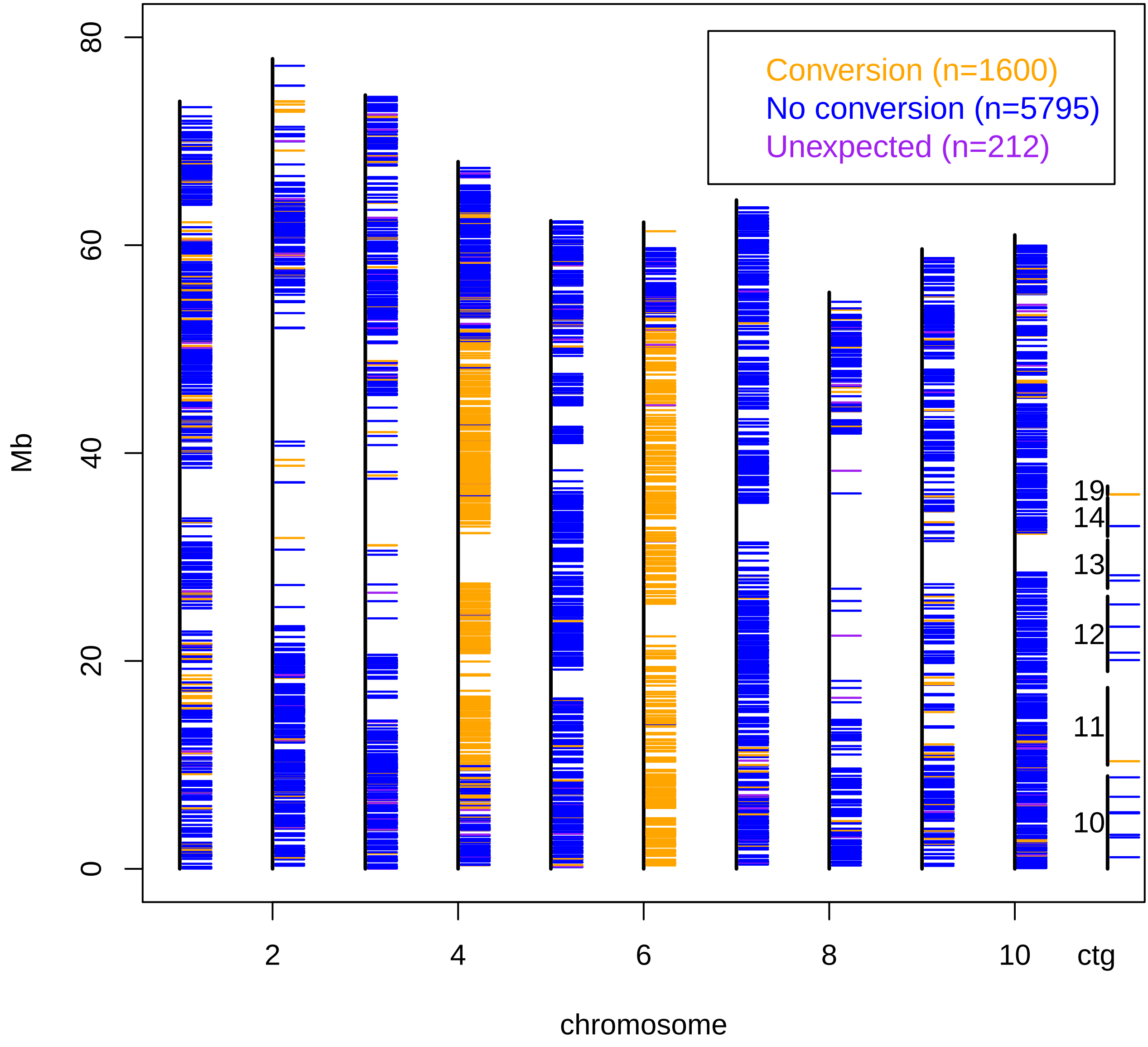

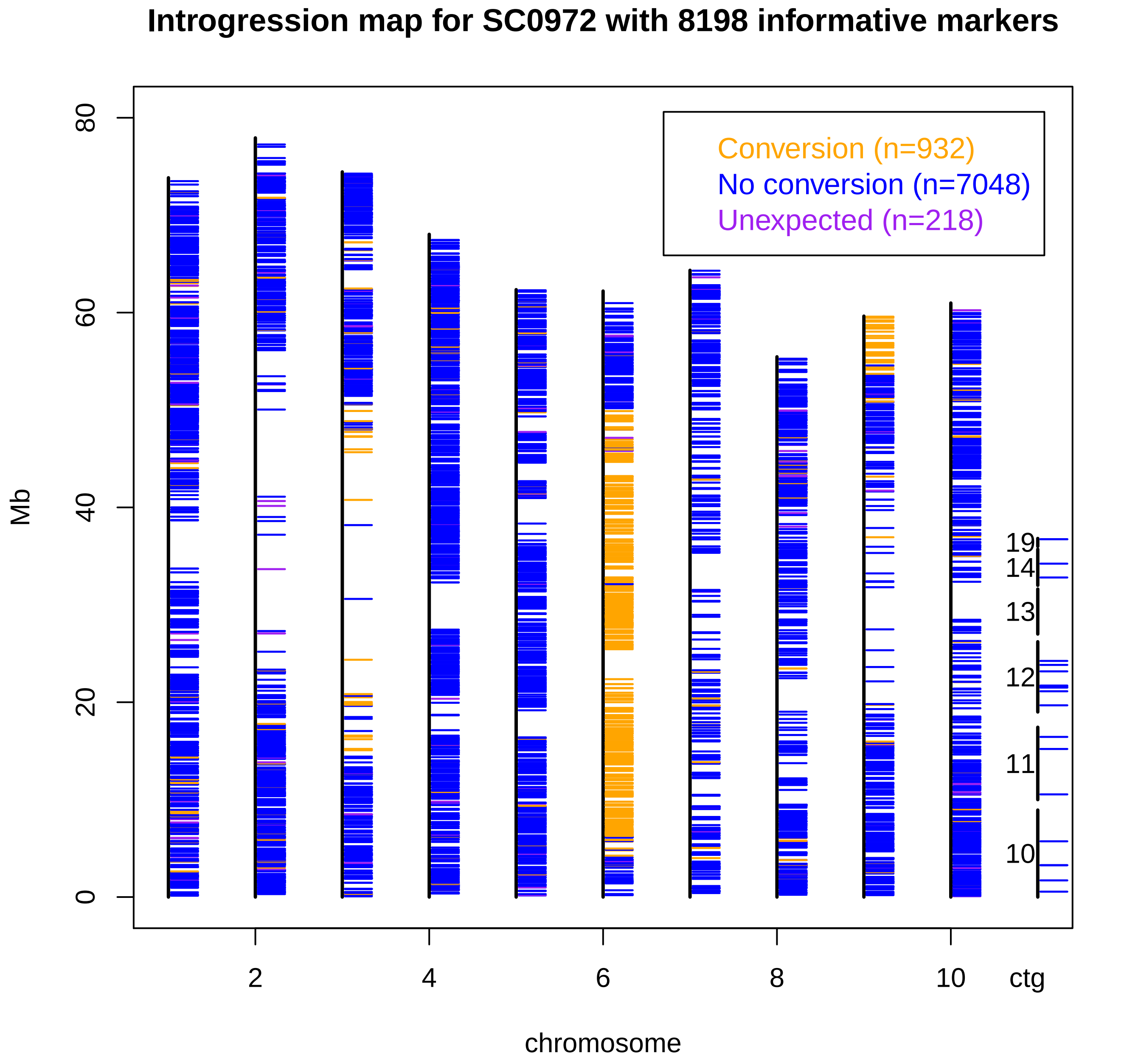
Introgression map for SC0972 with 8198 informative markers
Conversion (n=932)
No conversion (n=7048)
Unexpected (n=218)
Mb
0
20
40
60
80
chromosome
2
4
6
8
10
ctg
19
14
13
12
11
10

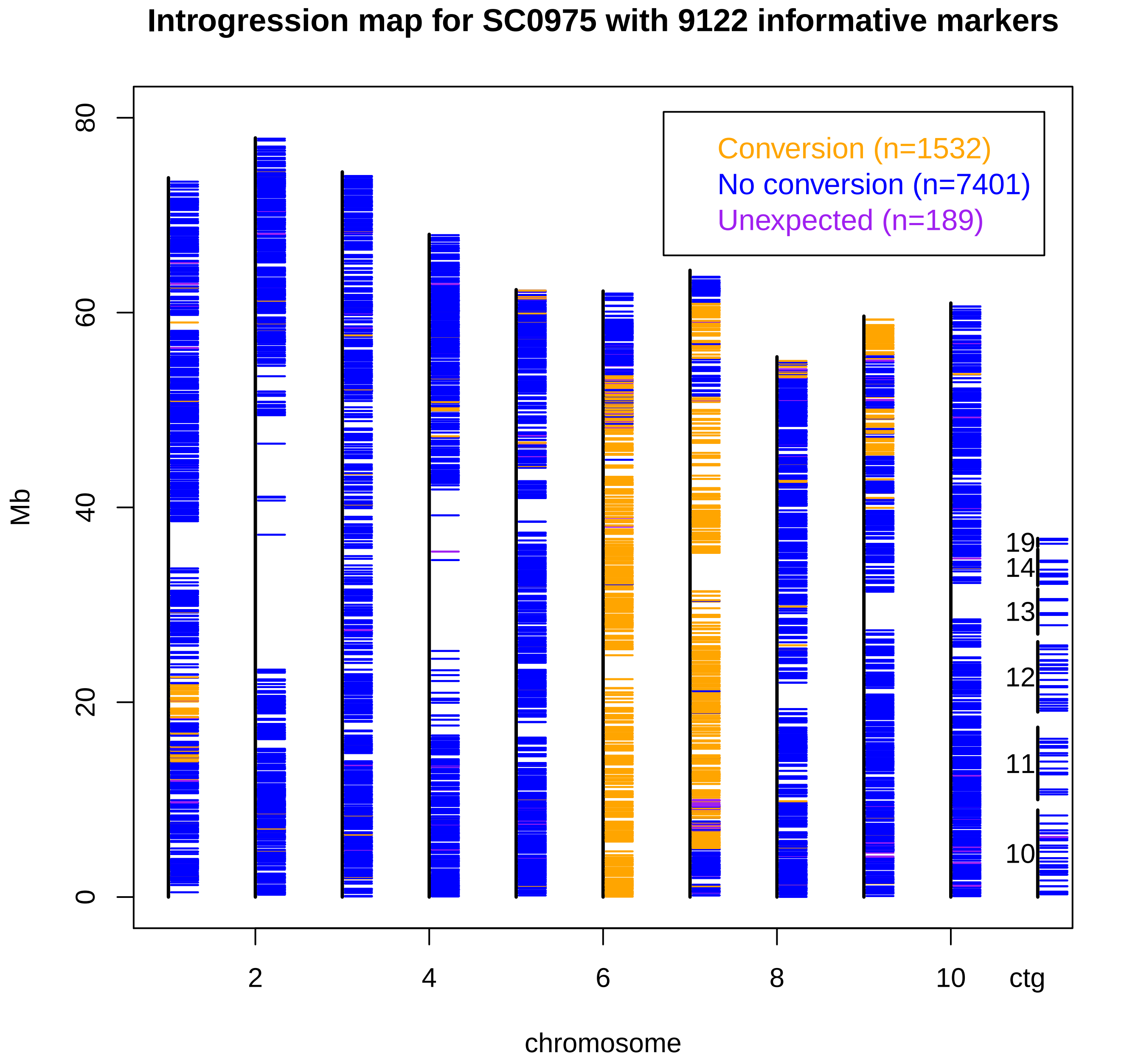
Introgression map for SC0975 with 9122 informative markers
Conversion (n=1532)
No conversion (n=7401)
Unexpected (n=189)
Mb
0
20
40
60
80
chromosome
2
4
6
8
10
ctg
19
14
13
12
11
10

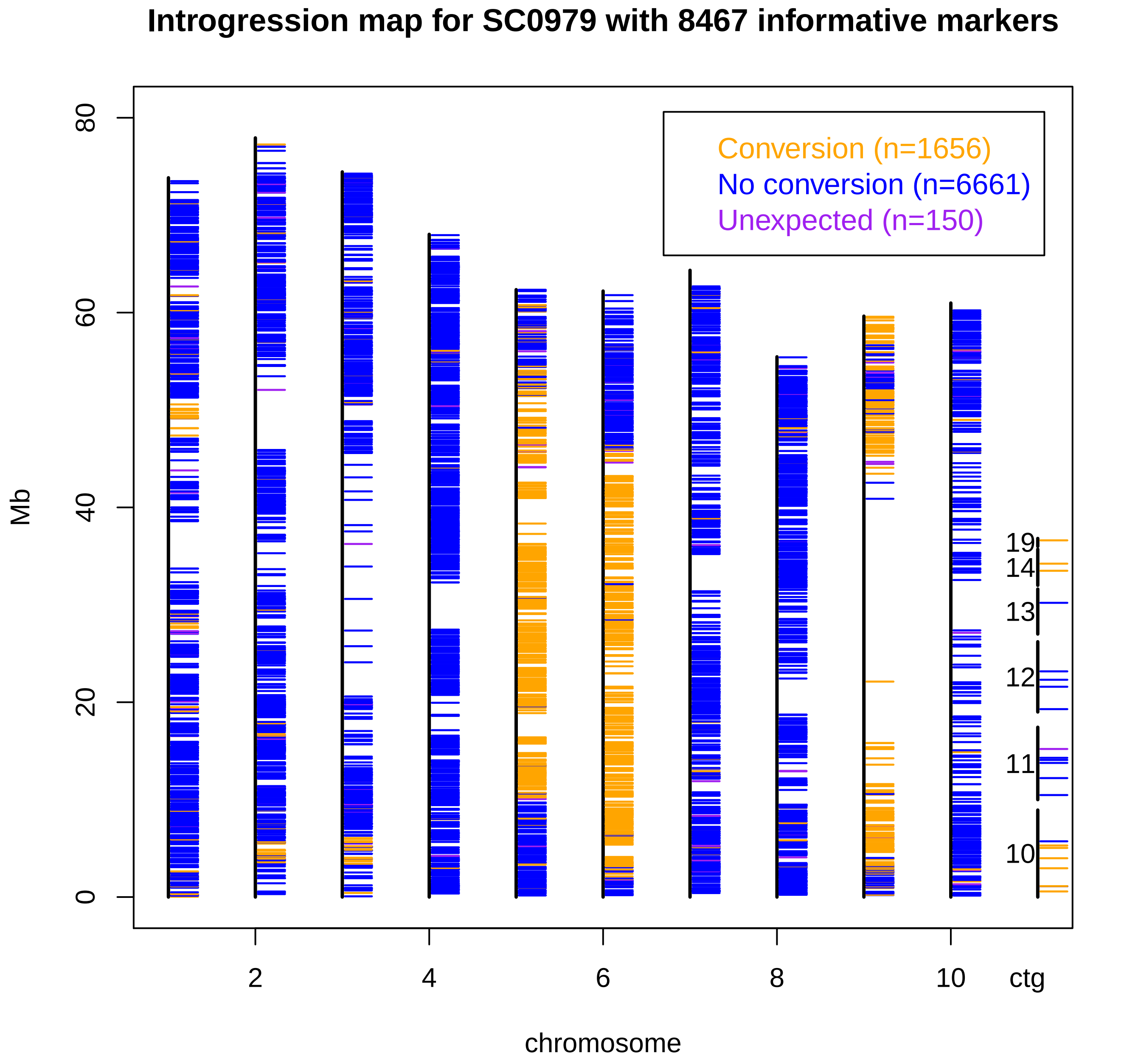
Introgression map for SC0979 with 8467 informative markers
Conversion (n=1656)
No conversion (n=6661)
Unexpected (n=150)
Mb
0
20
40
60
80
2
4
6
8
10
ctg
chromosome
19
14
13
12
11
10

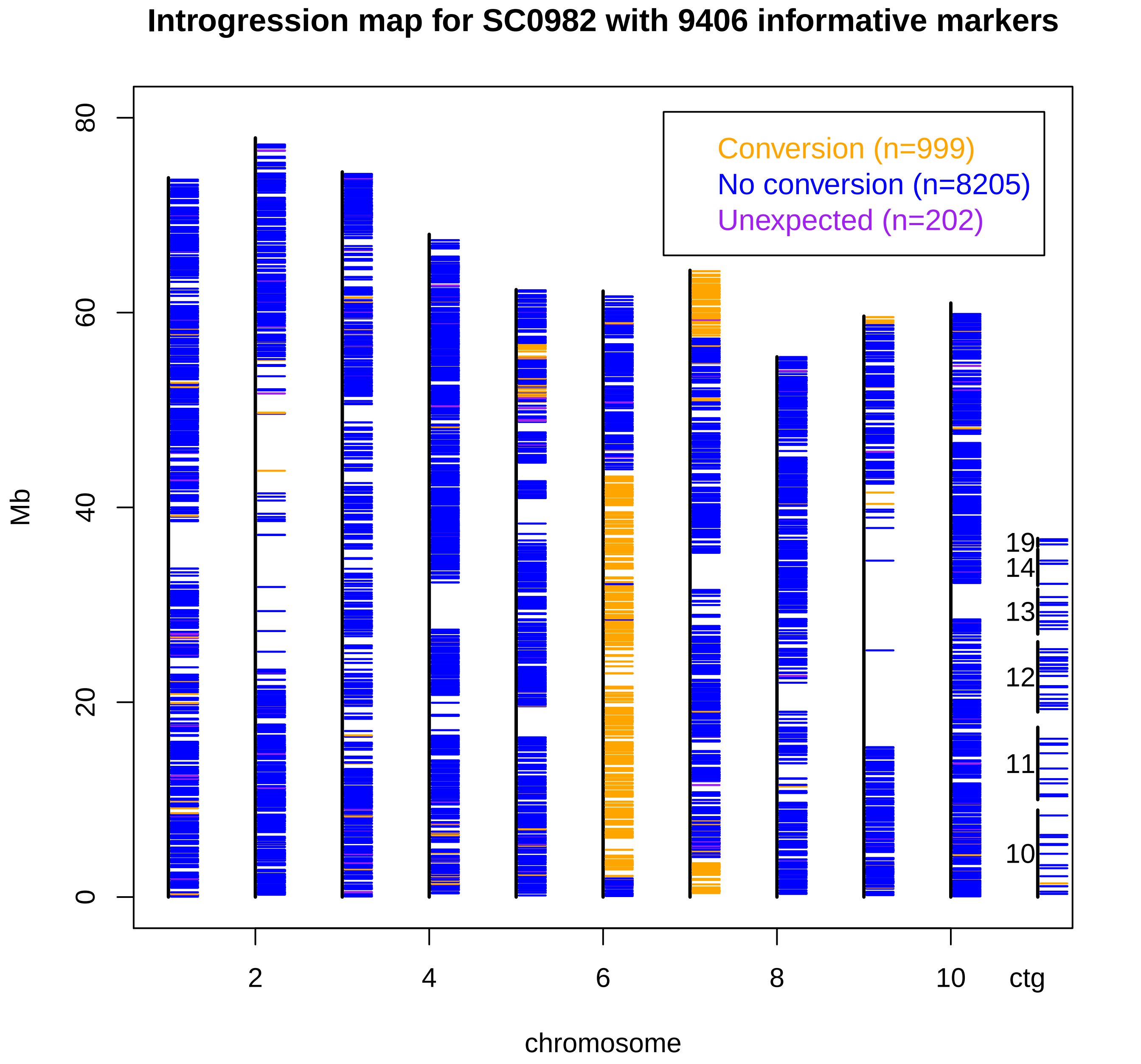

Introgression map for SC0982 with 9406 informative markers
Conversion (n=999)
No conversion (n=8205)
Unexpected (n=202)
Mb
0
20
40
60
80
chromosome
2
4
6
8
10
ctg
19
14
13
12
11
10

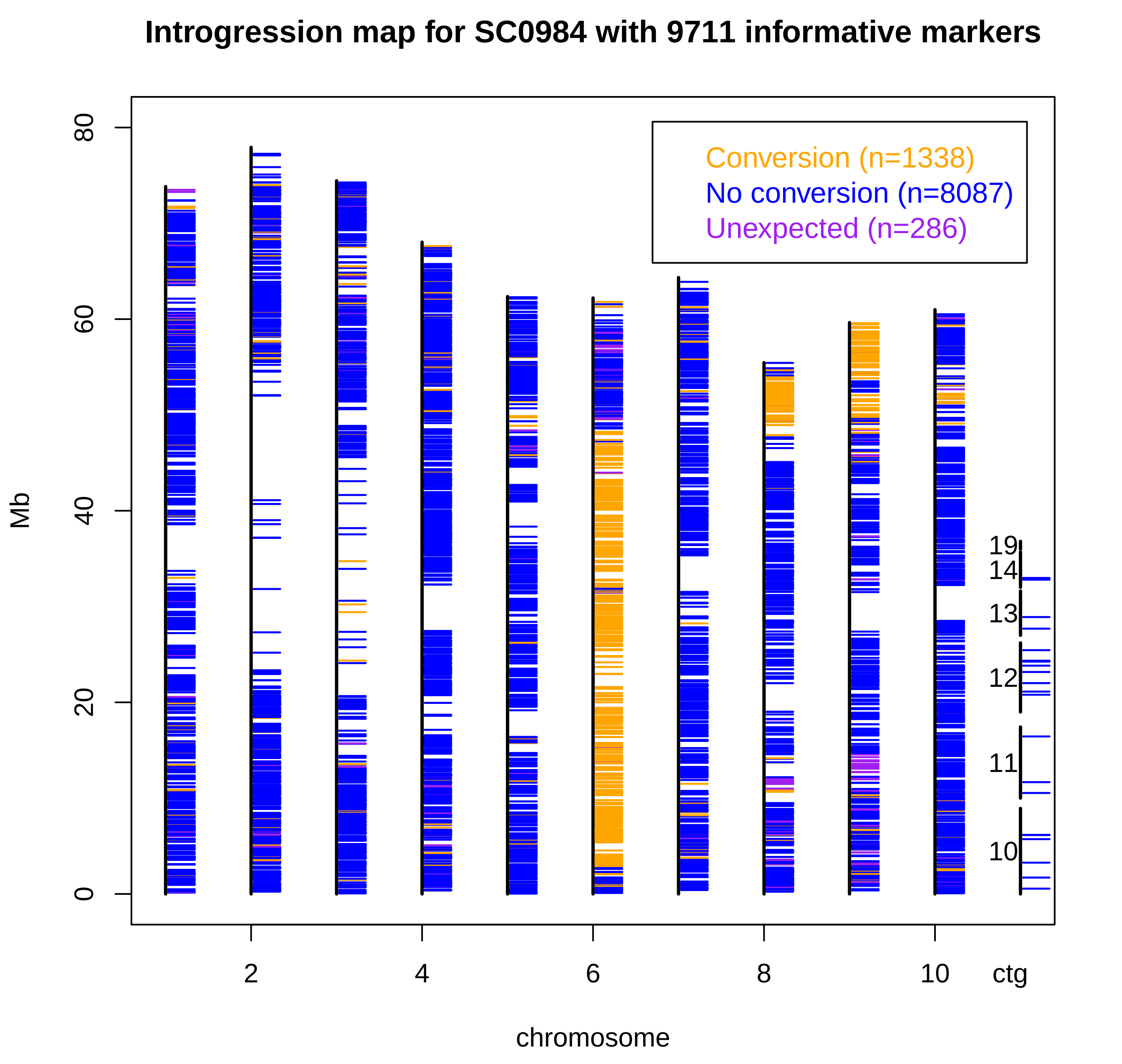

Introgression map for SC0984 with 9711 informative markers
Conversion (n=1338)
No conversion (n=8087)
Unexpected (n=286)
Mb
0
20
40
60
80
chromosome
2
4
6
8
10
ctg
19
14
13
12
11
10

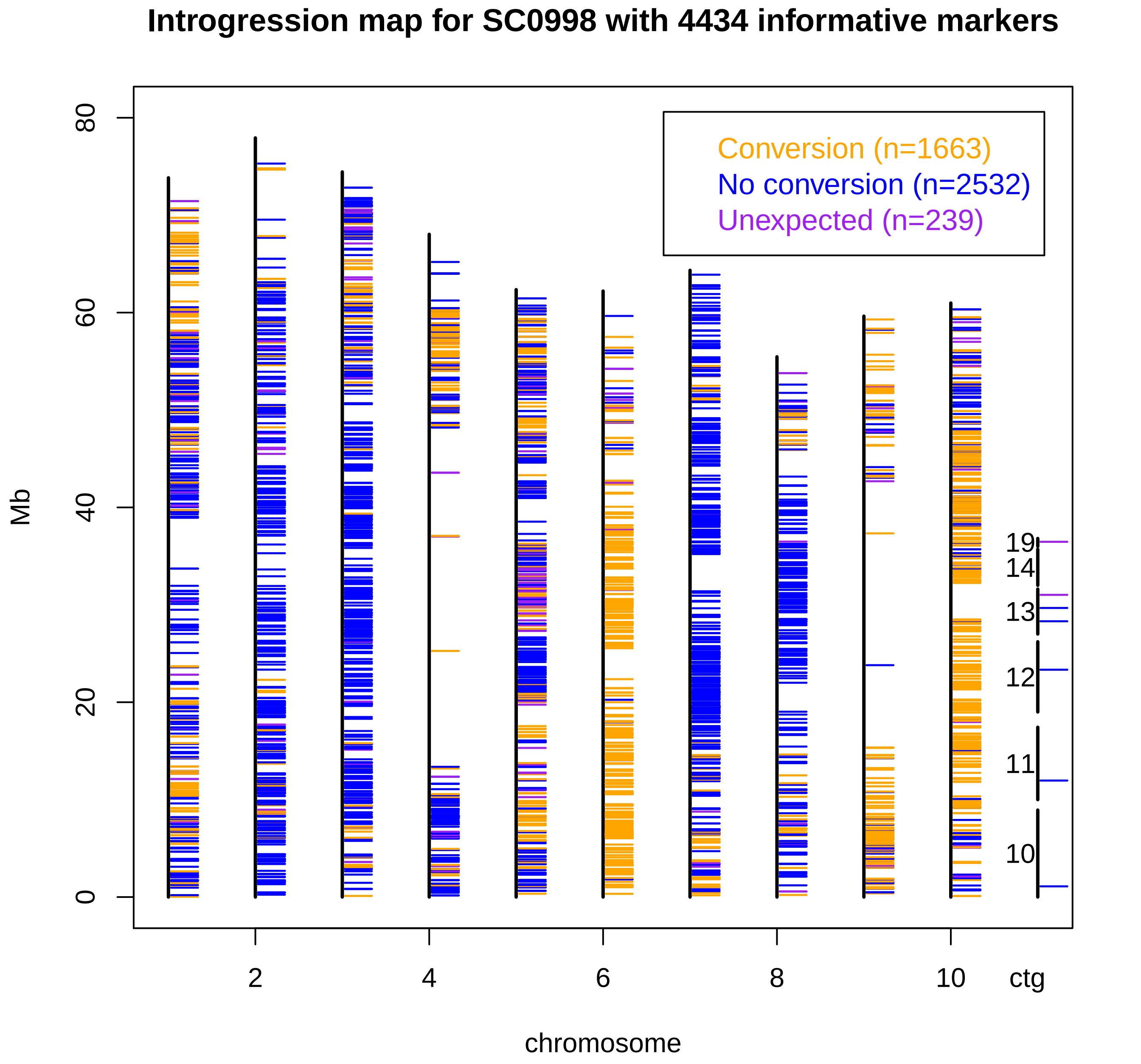

Introgression map for SC0998 with 4434 informative markers
Conversion (n=1663)
No conversion (n=2532)
Unexpected (n=239)
Mb
0
20
40
60
80
2
4
6
8
10
ctg
19
14
13
12
11
10
chromosome

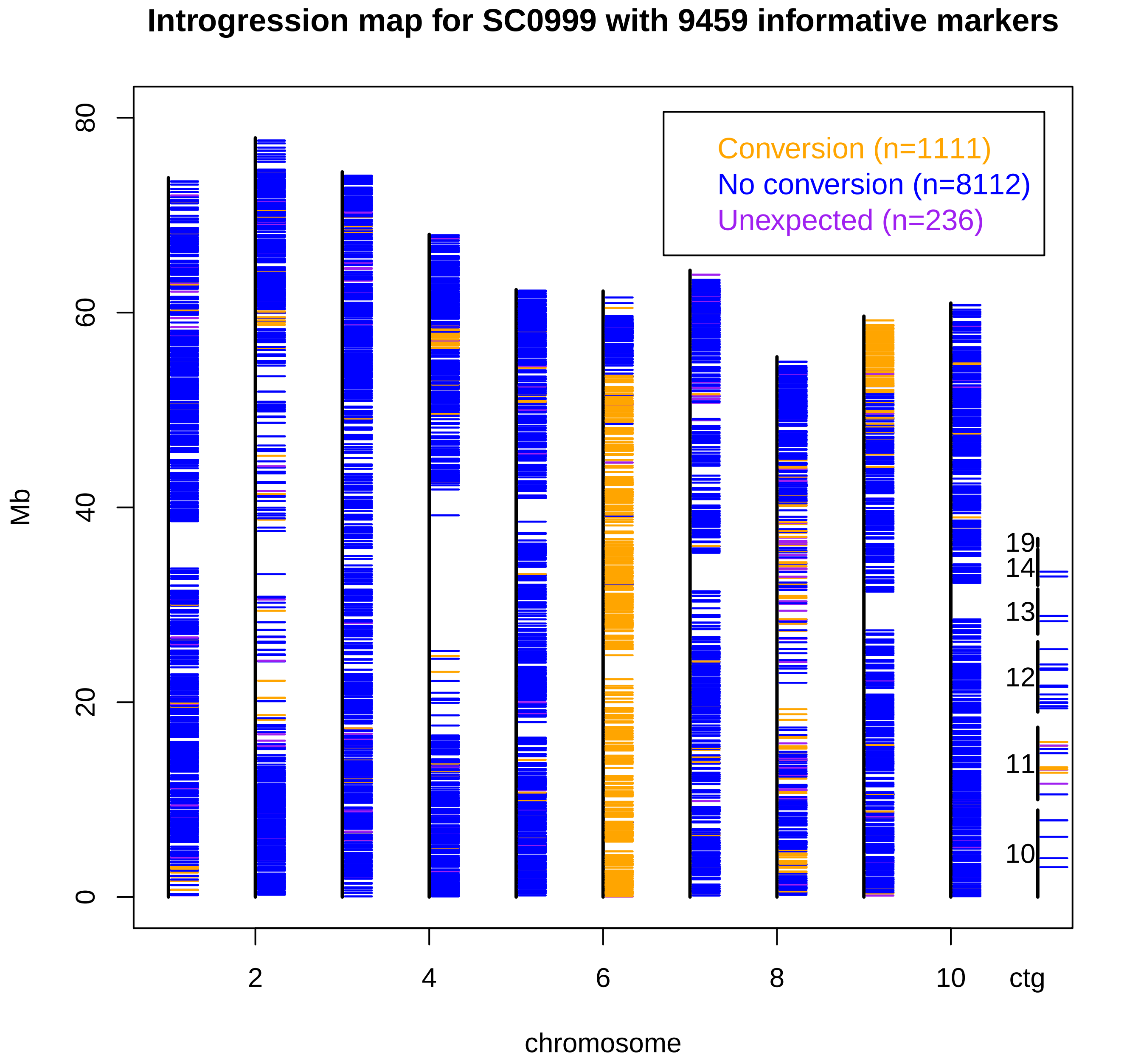

Introgression map for SC0999 with 9459 informative markers
Conversion (n=1111)
No conversion (n=8112)
Unexpected (n=236)
Mb
0
20
40
60
80
chromosome
2
4
6
8
10
ctg
19
14
13
12
11
10

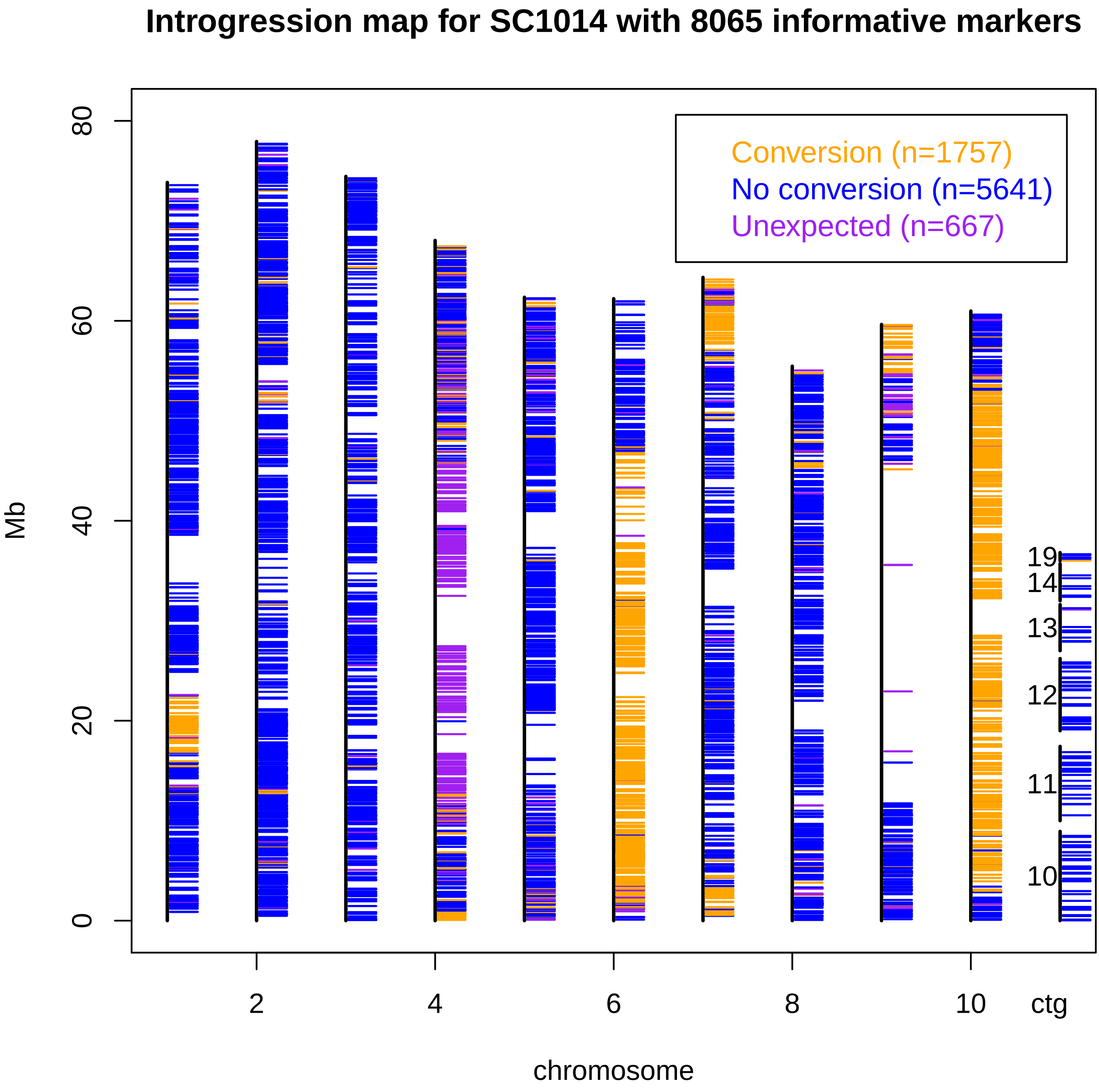
Introgression map for SC1014 with 8065 informative markers
Conversion (n=1757)
No conversion (n=5641)
Unexpected (n=667)
Mb
0
20
40
60
80
chromosome
2
4
6
8
10
ctg
19
14
13
12
11
10

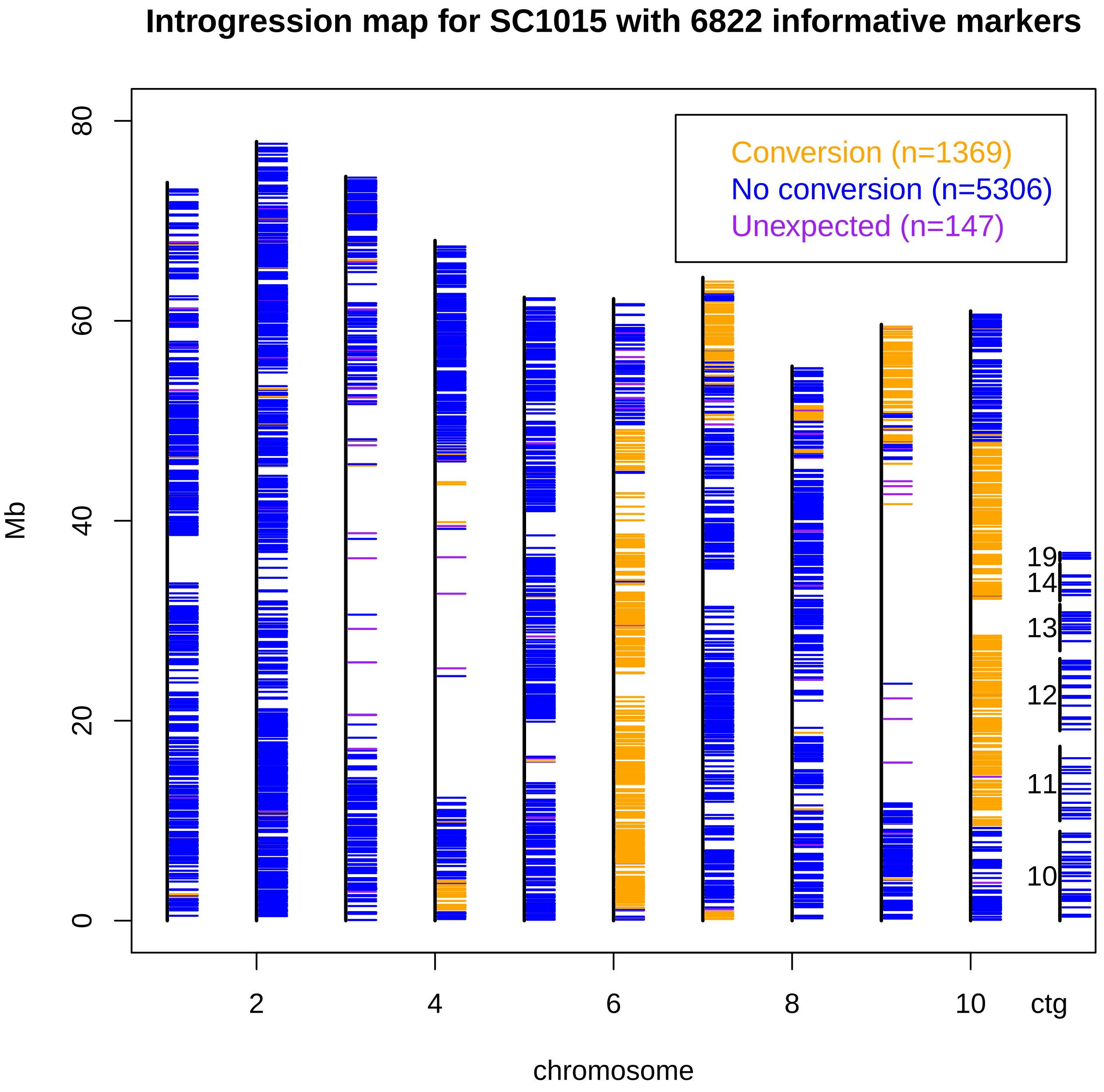
Introgression map for SC1015 with 6822 informative markers
Conversion (n=1369)
No conversion (n=5306)
Unexpected (n=147)
Mb
0
20
40
60
80
chromosome
2
4
6
8
10
ctg
19
14
13
12
11
10

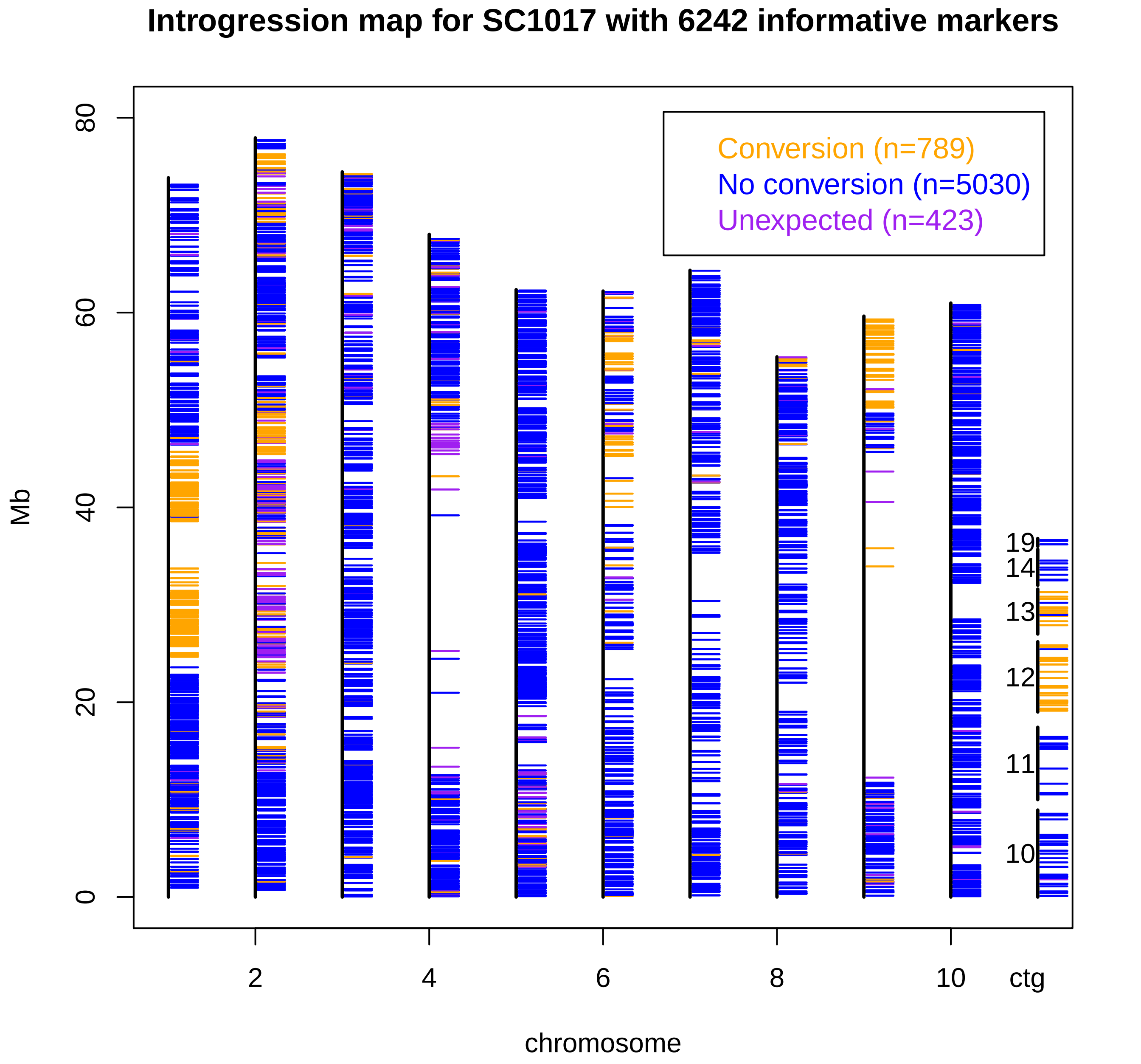
Introgression map for SC1017 with 6242 informative markers
Conversion (n=789)
No conversion (n=5030)
Unexpected (n=423)
Mb
0
20
40
60
80
chromosome
2
4
6
8
10
ctg
19
14
13
12
11
10

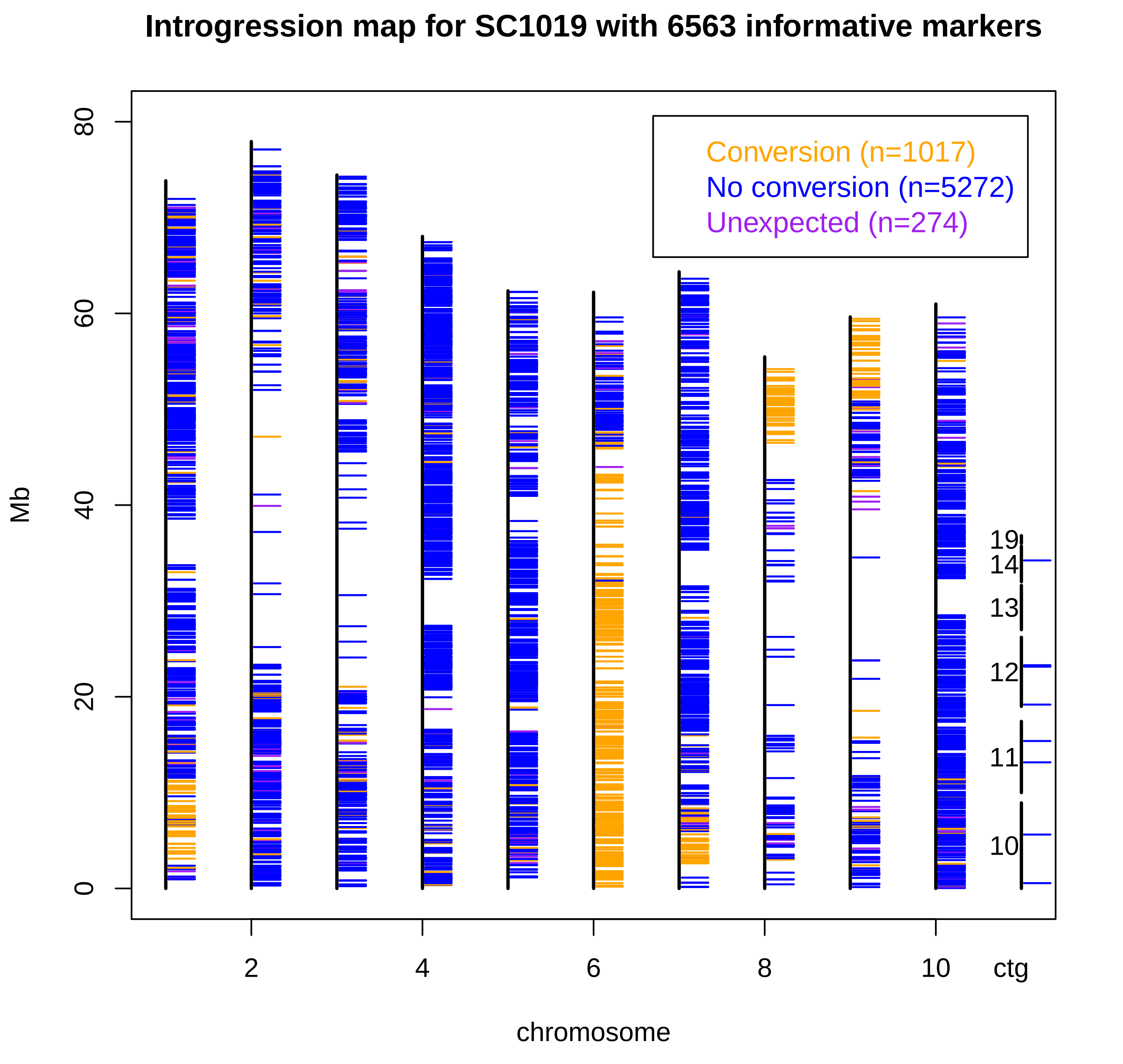
Introgression map for SC1019 with 6563 informative markers
Conversion (n=1017)
No conversion (n=5272)
Unexpected (n=274)
Mb
0
20
40
60
80
chromosome
2
4
6
8
10
ctg
19
14
13
12
11
10

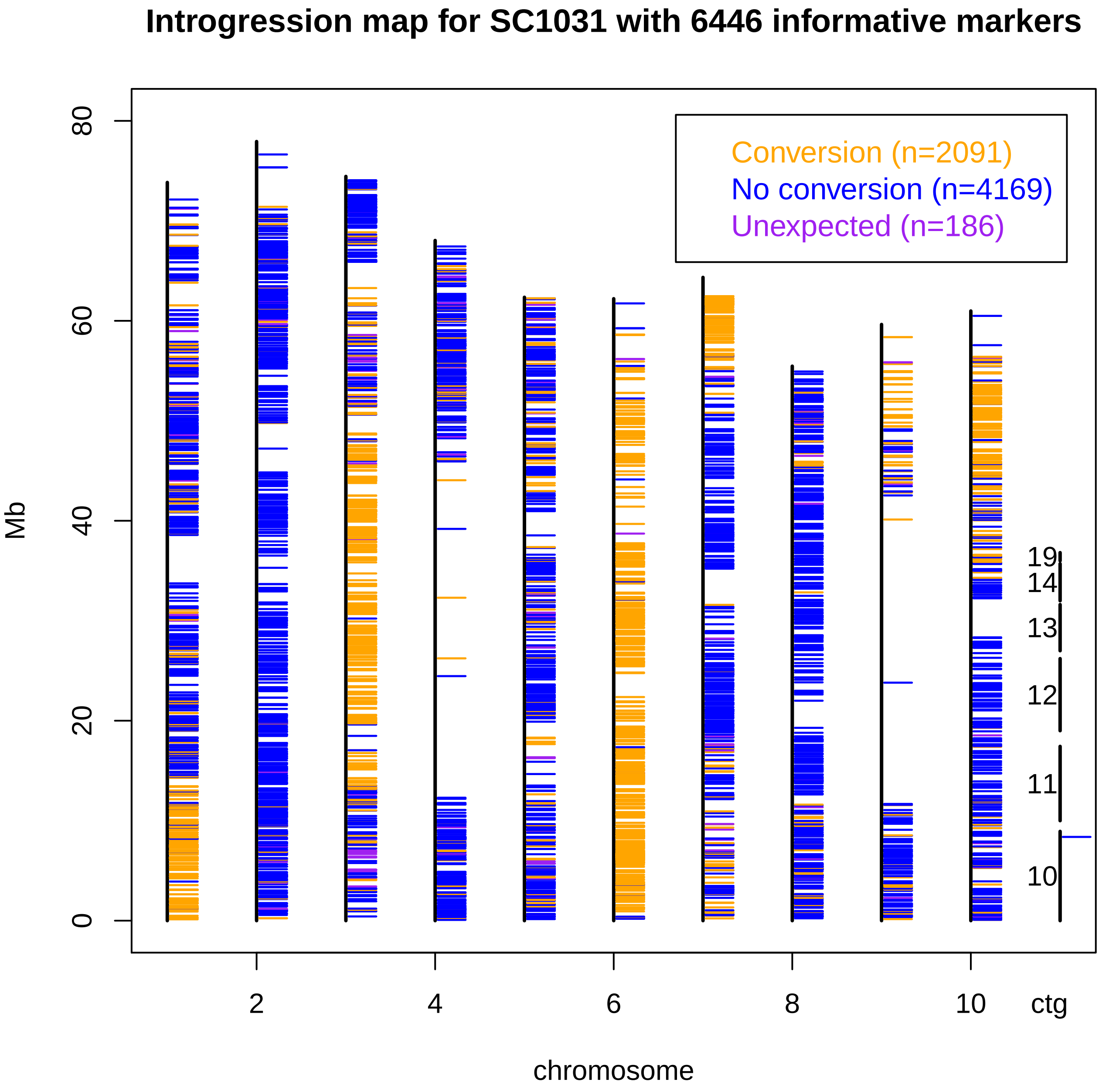

Introgression map for SC1031 with 6446 informative markers
Conversion (n=2091)
No conversion (n=4169)
Unexpected (n=186)
Mb
0
20
40
60
80
chromosome
2
4
6
8
10
ctg
19
14
13
12
11
10

Introgression map for SC1033 with 5107 informative markers

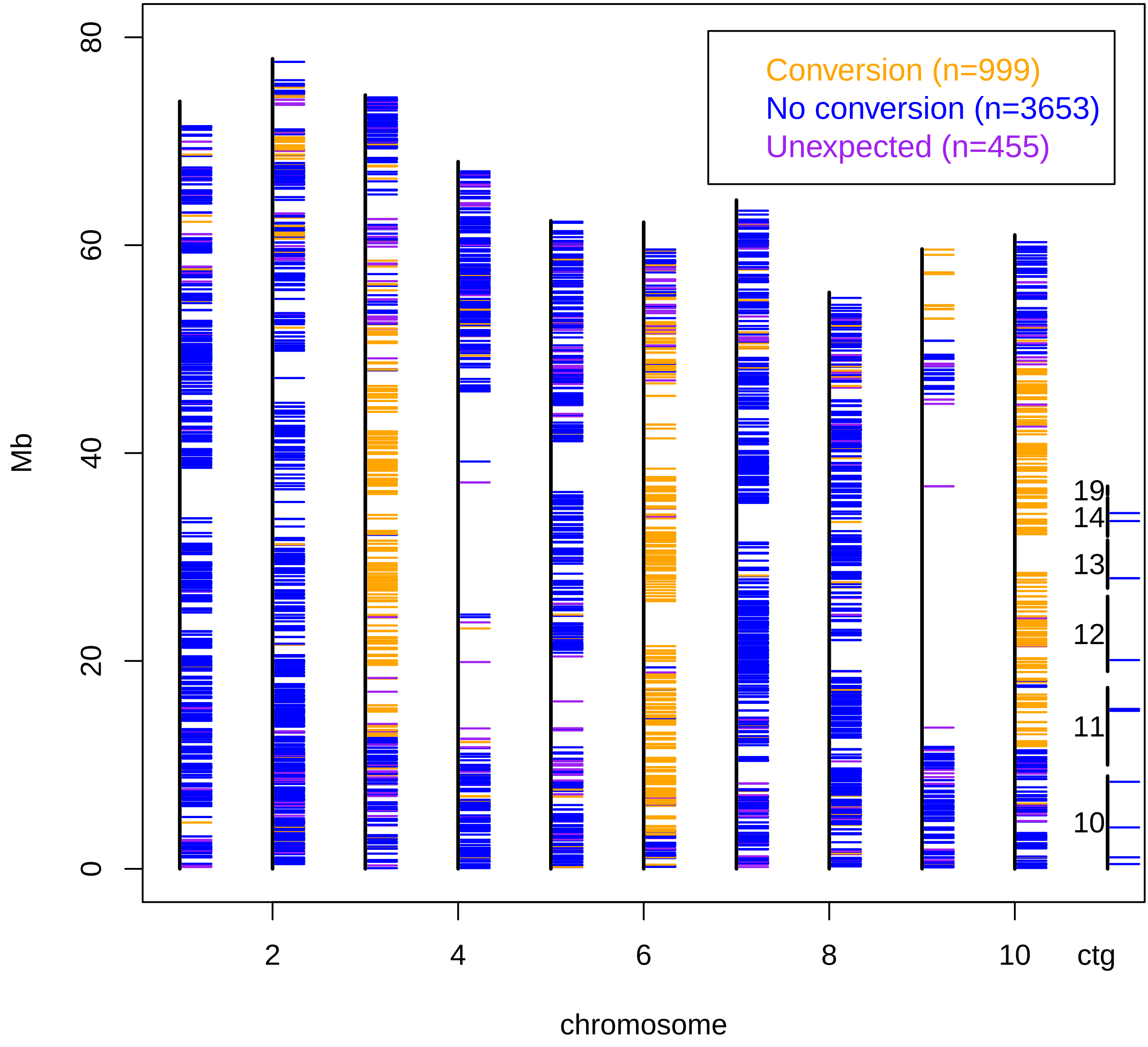

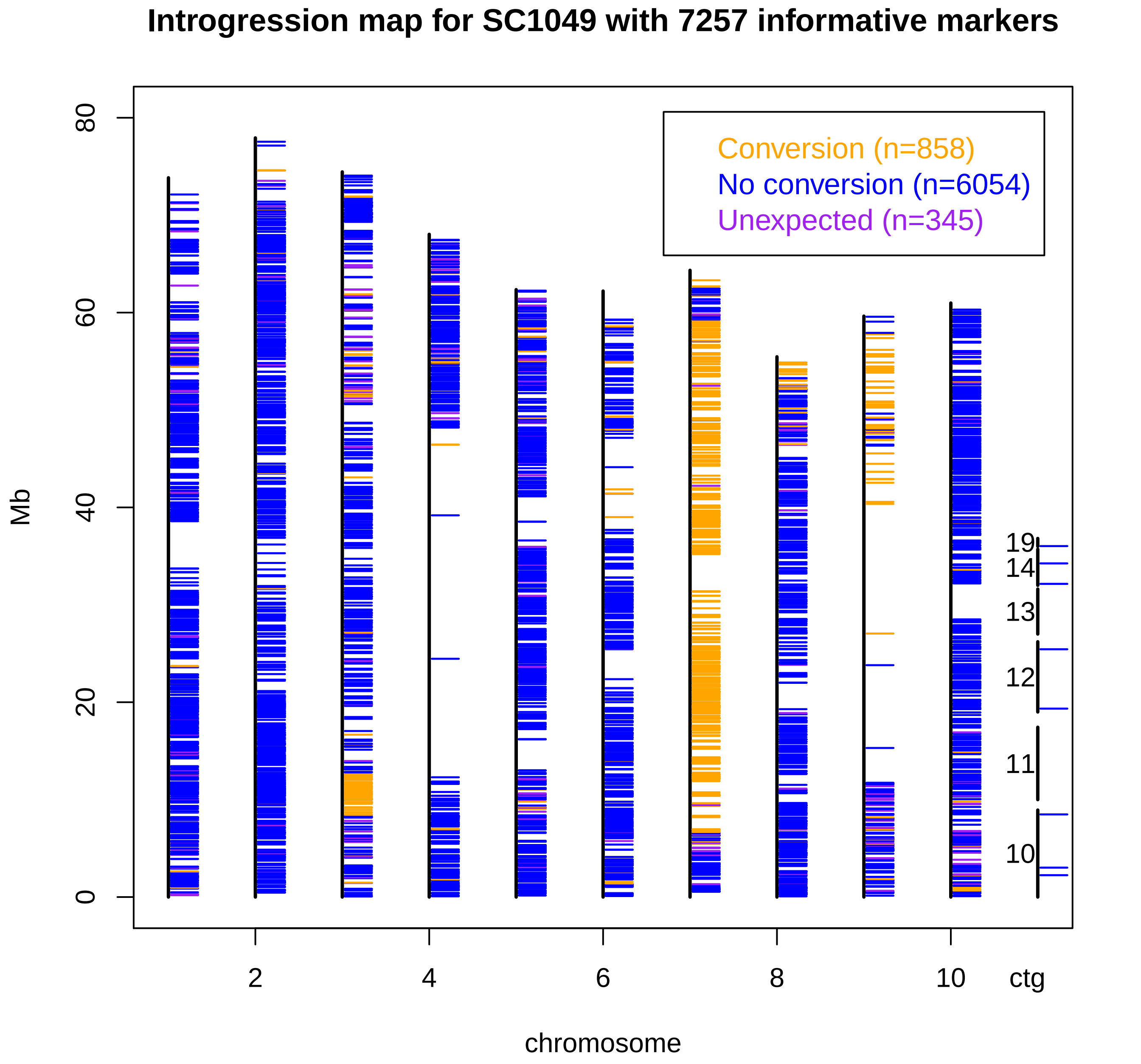
Introgression map for SC1049 with 7257 informative markers
Conversion (n=858)
No conversion (n=6054)
Unexpected (n=345)
Mb
0
20
40
60
80
chromosome
2
4
6
8
10
ctg
19
14
13
12
11
10

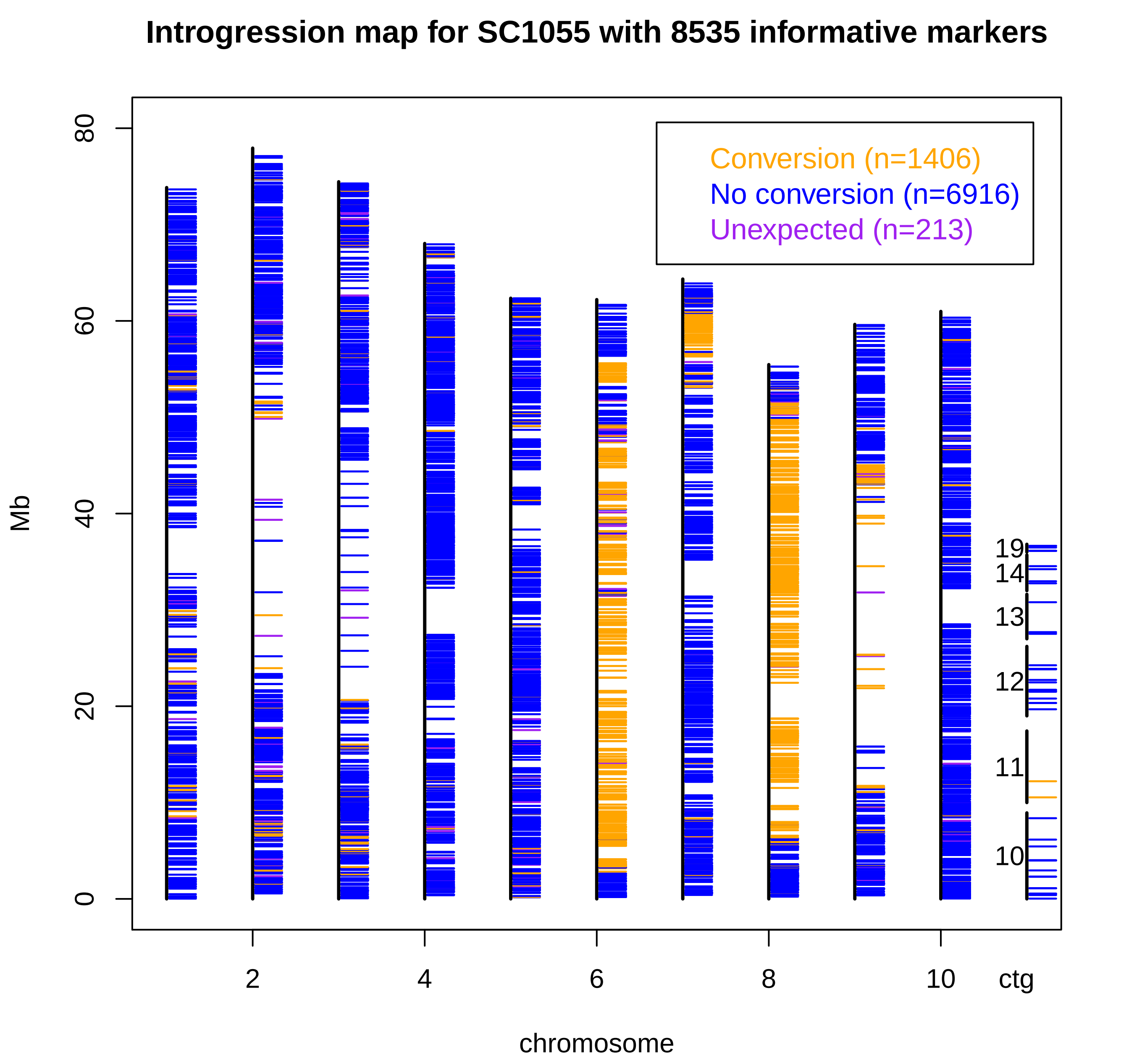
Introgression map for SC1055 with 8535 informative markers
Conversion (n=1406)
No conversion (n=6916)
Unexpected (n=213)
Mb
0
20
40
60
80
chromosome
2
4
6
8
10
ctg
19
14
13
12
11
10

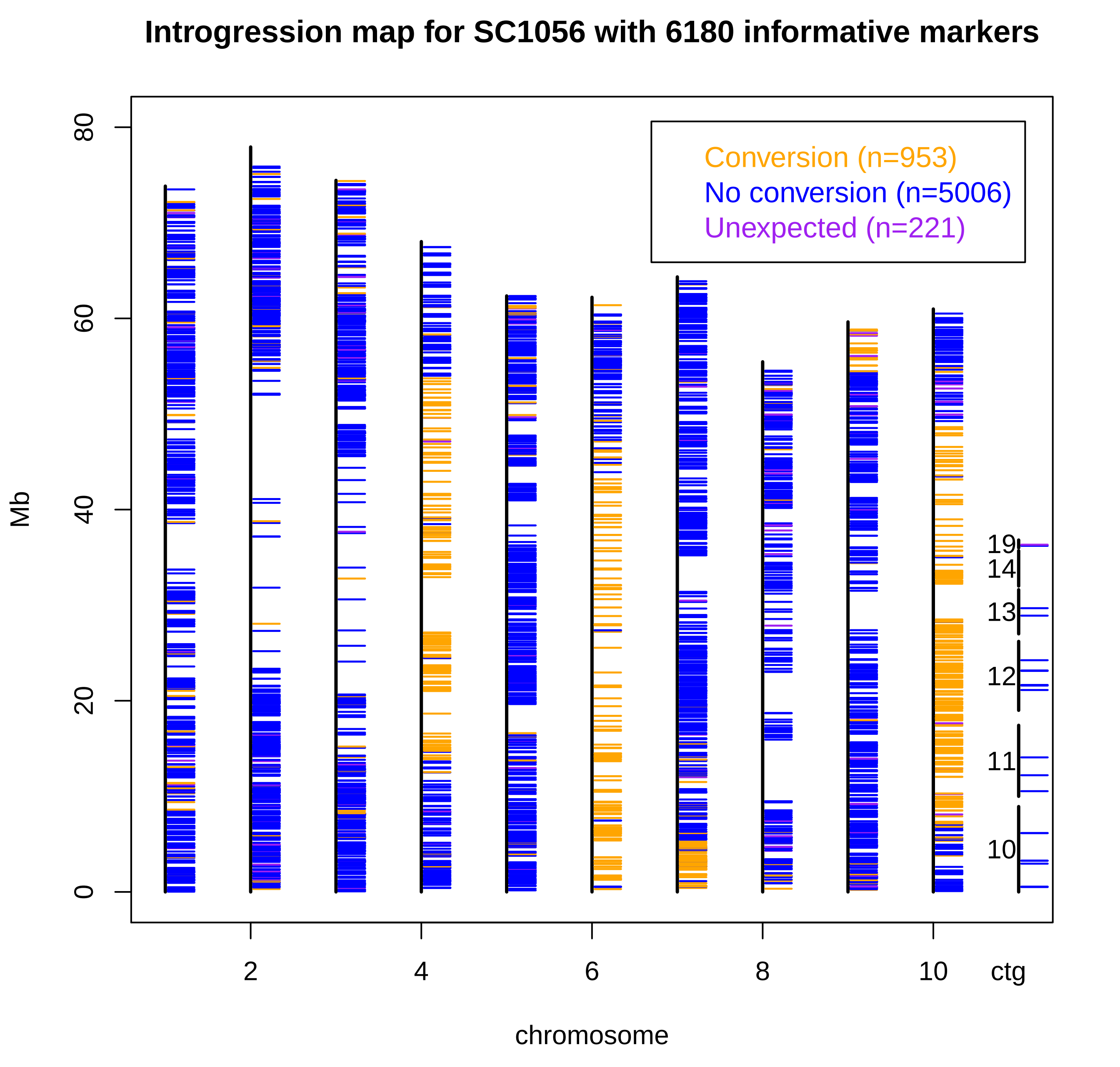

Introgression map for SC1056 with 6180 informative markers
Conversion (n=953)
No conversion (n=5006)
Unexpected (n=221)
Mb
0
20
40
60
80
chromosome
2
4
6
8
10
ctg
19
14
13
12
11
10

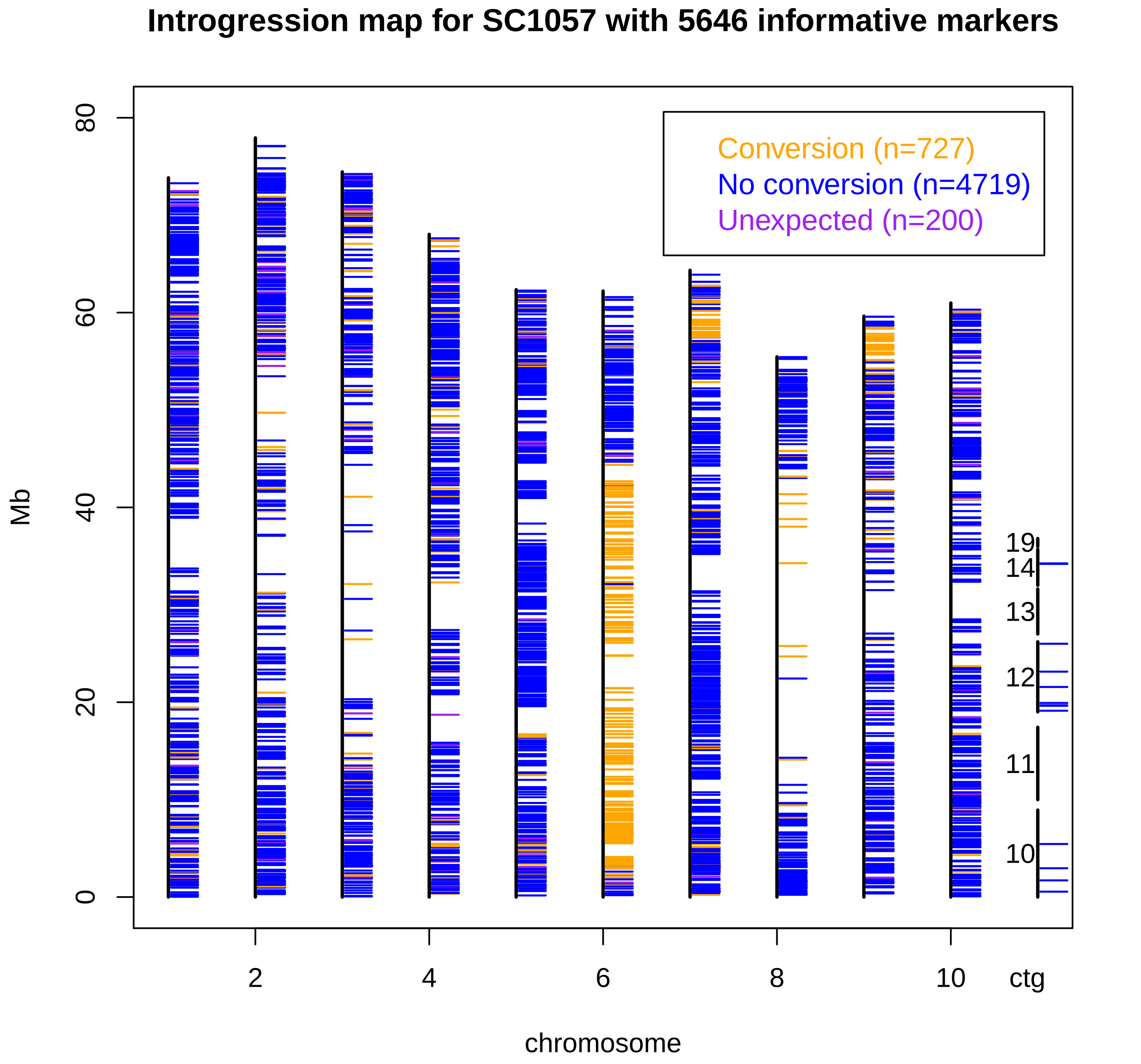
Introgression map for SC1057 with 5646 informative markers
Conversion (n=727)
No conversion (n=4719)
Unexpected (n=200)
Mb
0
20
40
60
80
2
4
6
8
10
ctg
19
14
13
12
11
10
chromosome

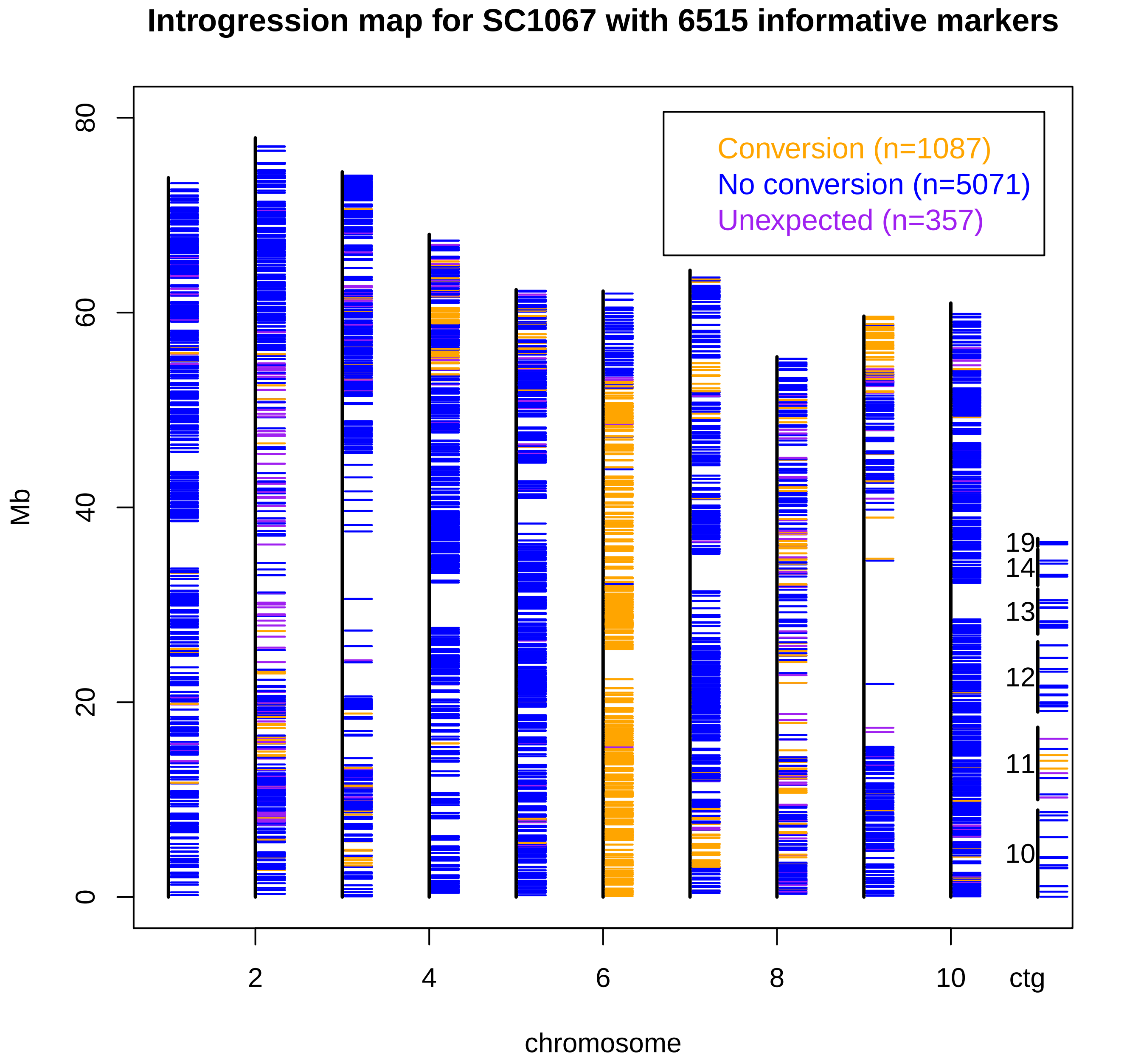

Introgression map for SC1067 with 6515 informative markers
Conversion (n=1087)
No conversion (n=5071)
Unexpected (n=357)
Mb
0
20
40
60
80
chromosome
2
4
6
8
10
ctg
19
14
13
12
11
10

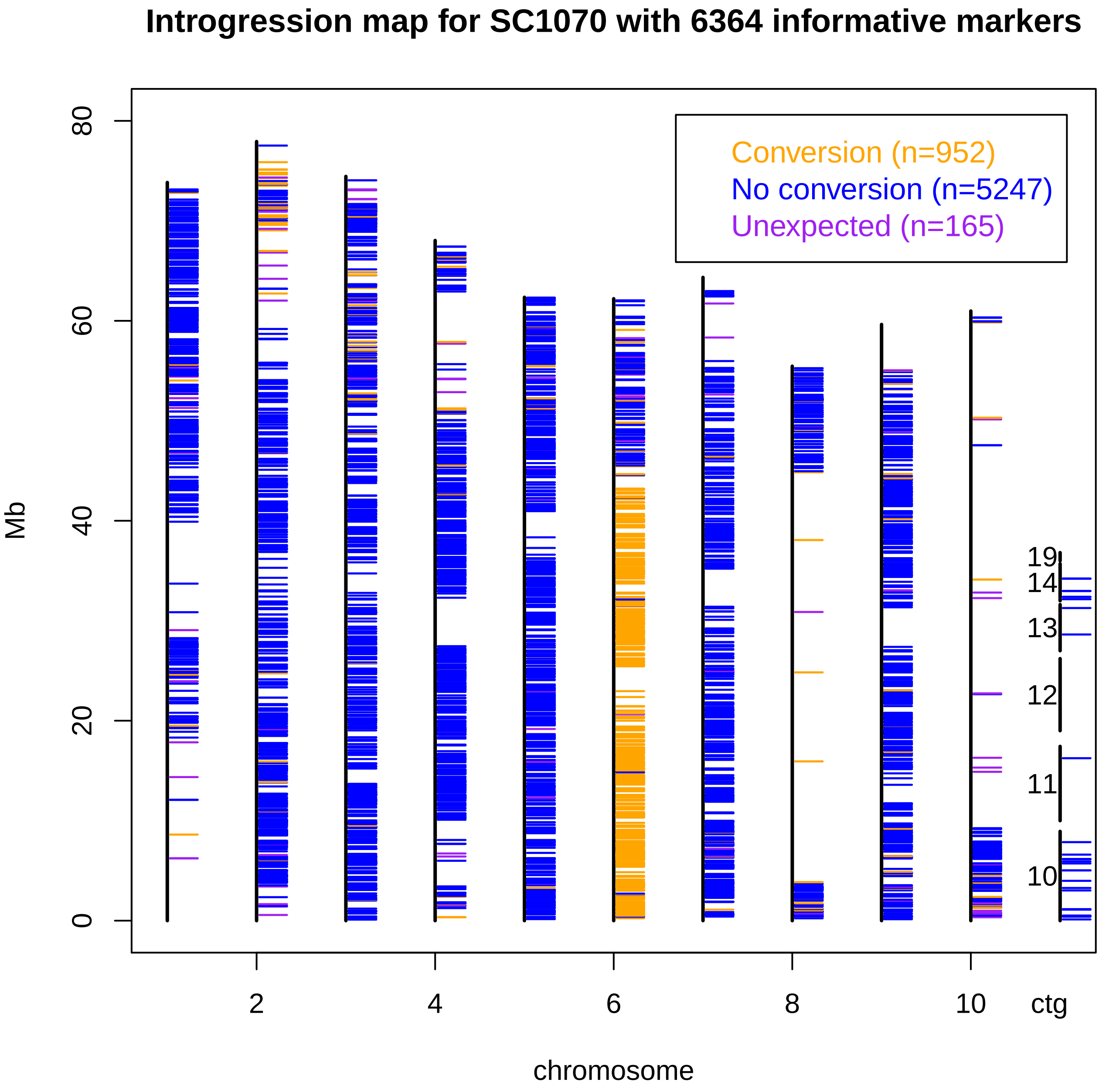

Introgression map for SC1070 with 6364 informative markers
Conversion (n=952)
No conversion (n=5247)
Unexpected (n=165)
Mb
0
20
40
60
80
chromosome
2
4
6
8
10
ctg
19
14
13
12
11
10

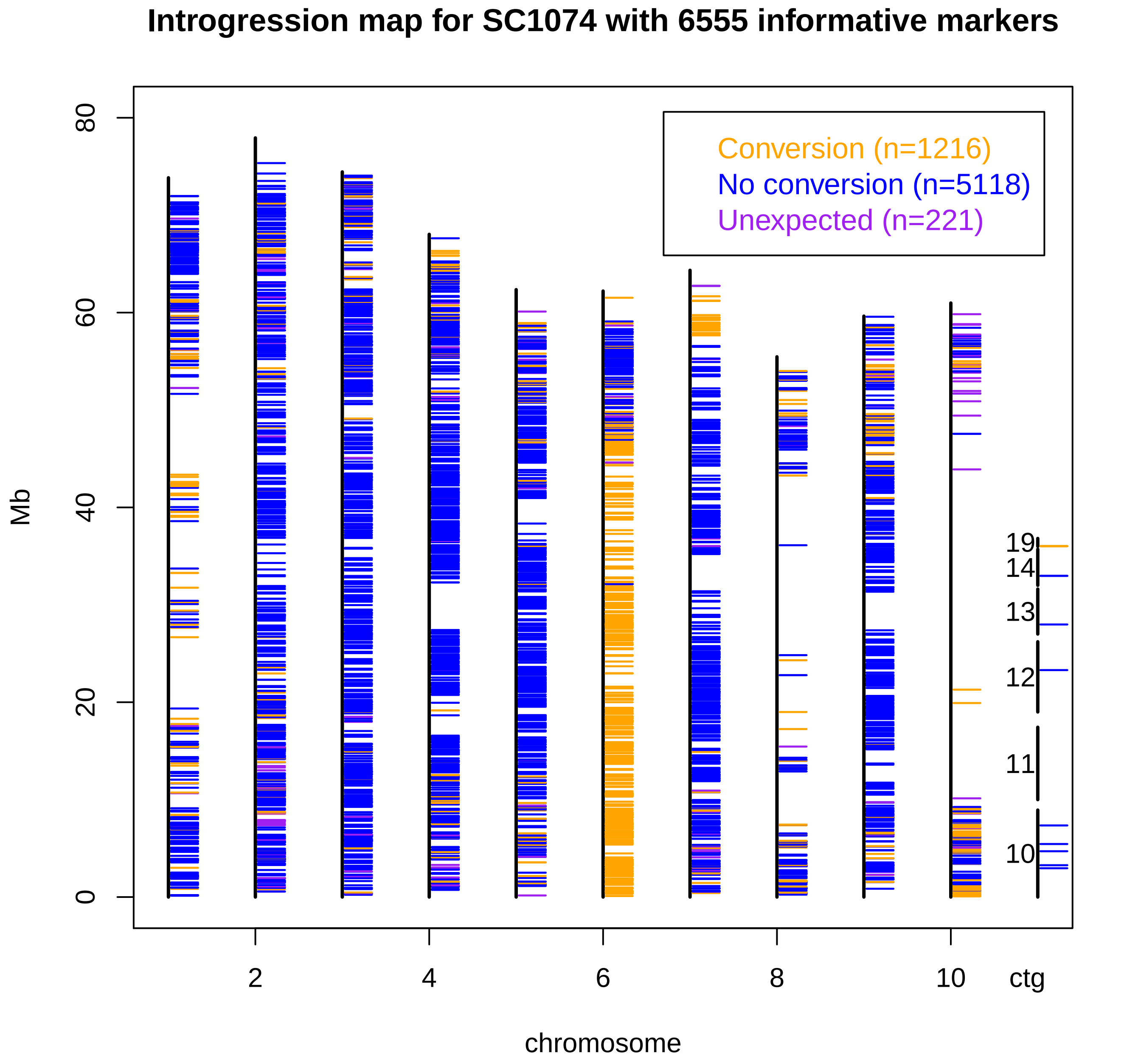
Introgression map for SC1074 with 6555 informative markers
Conversion (n=1216)
No conversion (n=5118)
Unexpected (n=221)
Mb
0
20
40
60
80
2
4
6
8
10
ctg
19
14
13
12
11
10
chromosome

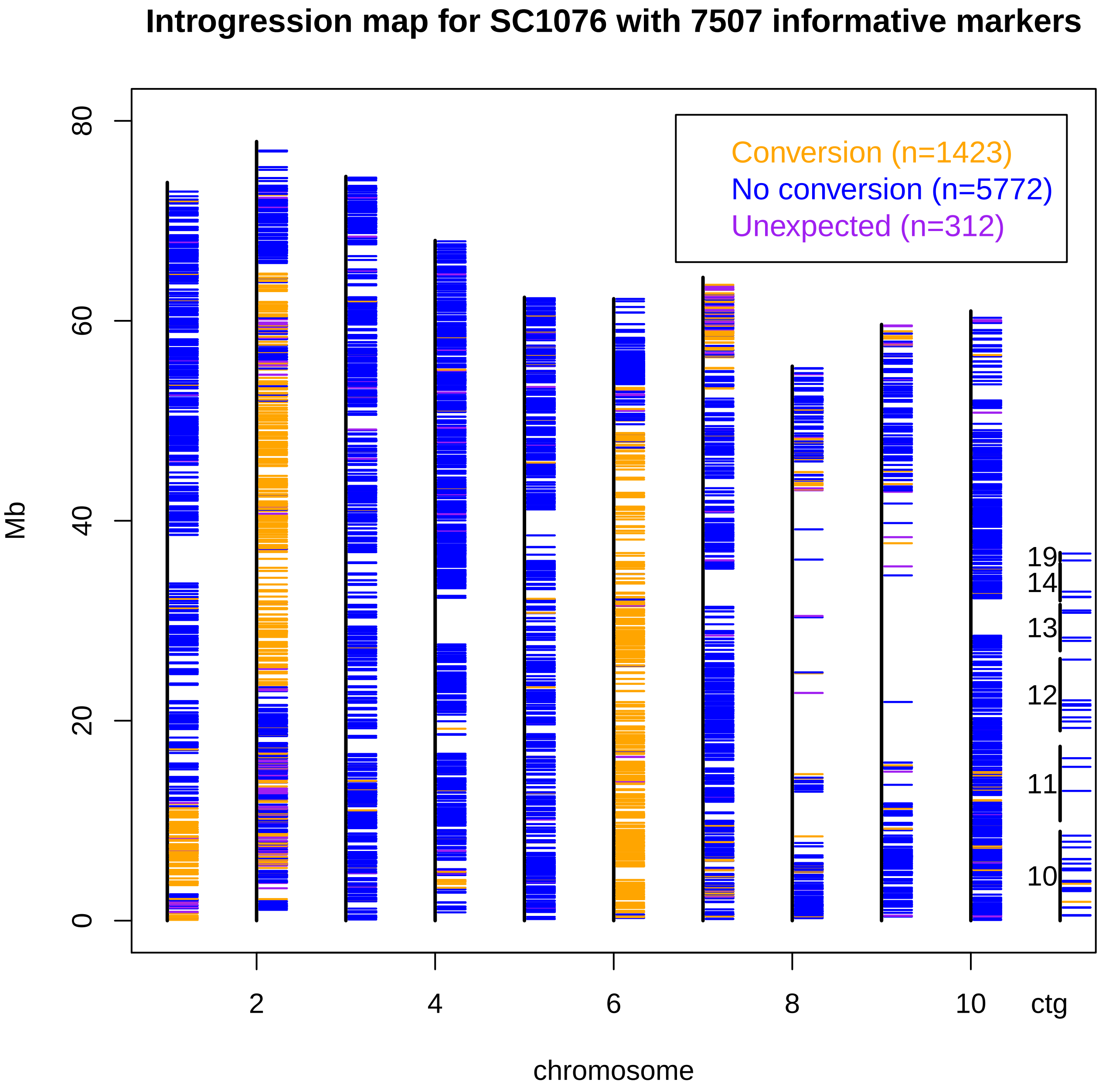

Introgression map for SC1076 with 7507 informative markers
Conversion (n=1423)
No conversion (n=5772)
Unexpected (n=312)
Mb
0
20
40
60
80
chromosome
2
4
6
8
10
ctg
19
14
13
12
11
10

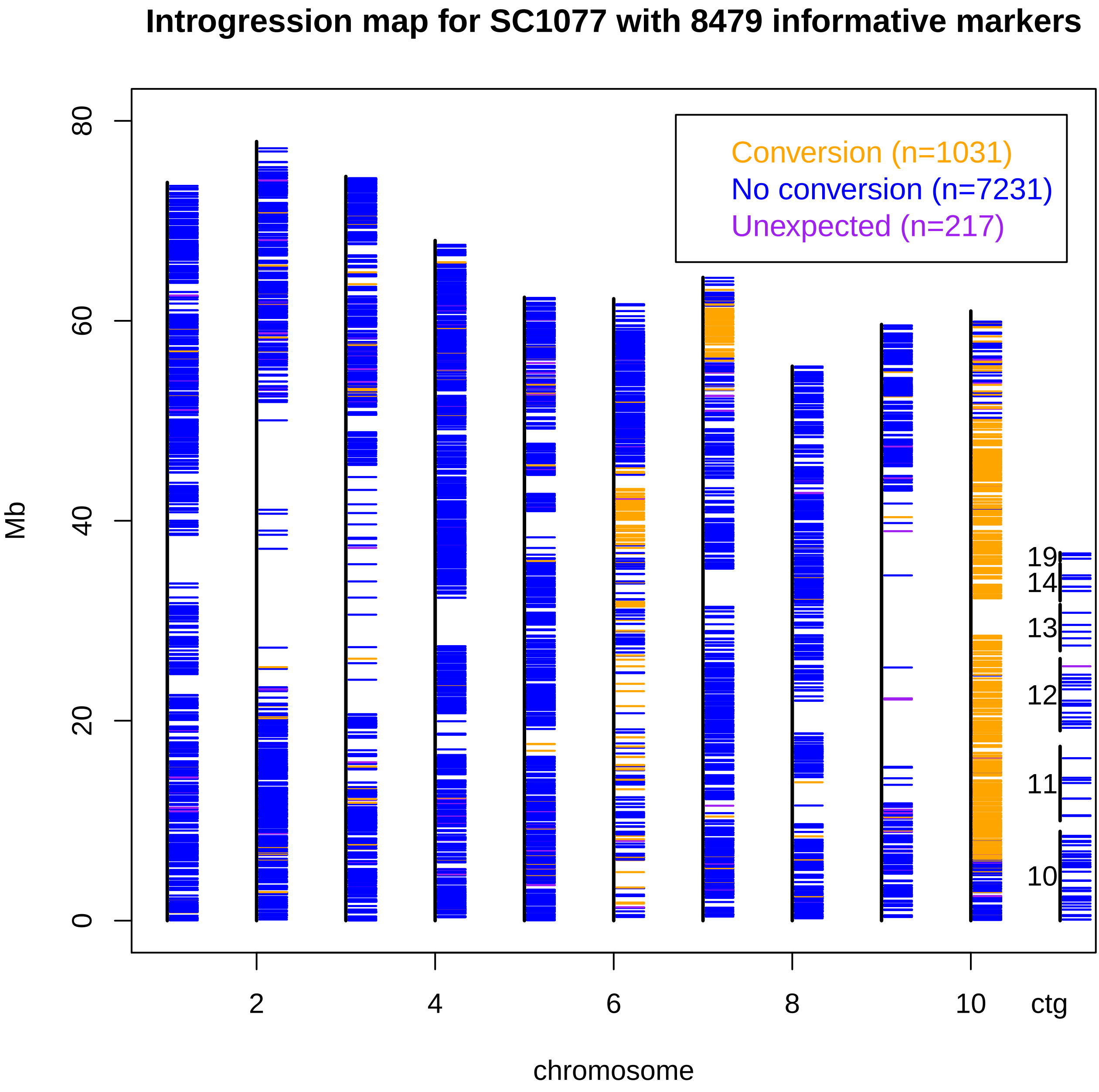
Introgression map for SC1077 with 8479 informative markers
Conversion (n=1031)
No conversion (n=7231)
Unexpected (n=217)
Mb
0
20
40
60
80
chromosome
2
4
6
8
10
ctg
19
14
13
12
11
10

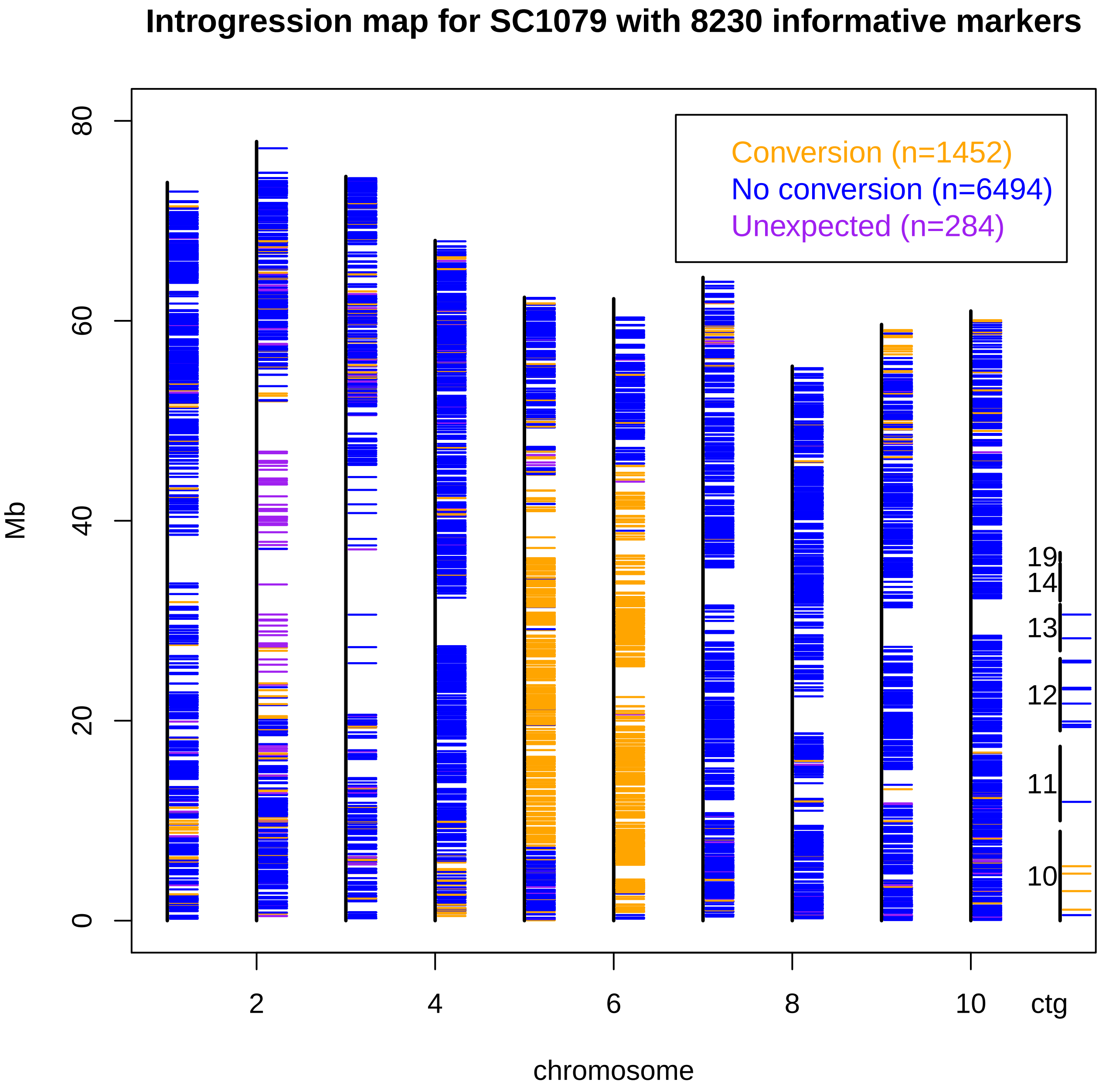
Introgression map for SC1079 with 8230 informative markers
Conversion (n=1452)
No conversion (n=6494)
Unexpected (n=284)
Mb
0
20
40
60
80
2
4
6
8
10
ctg
19
14
13
12
11
10
chromosome

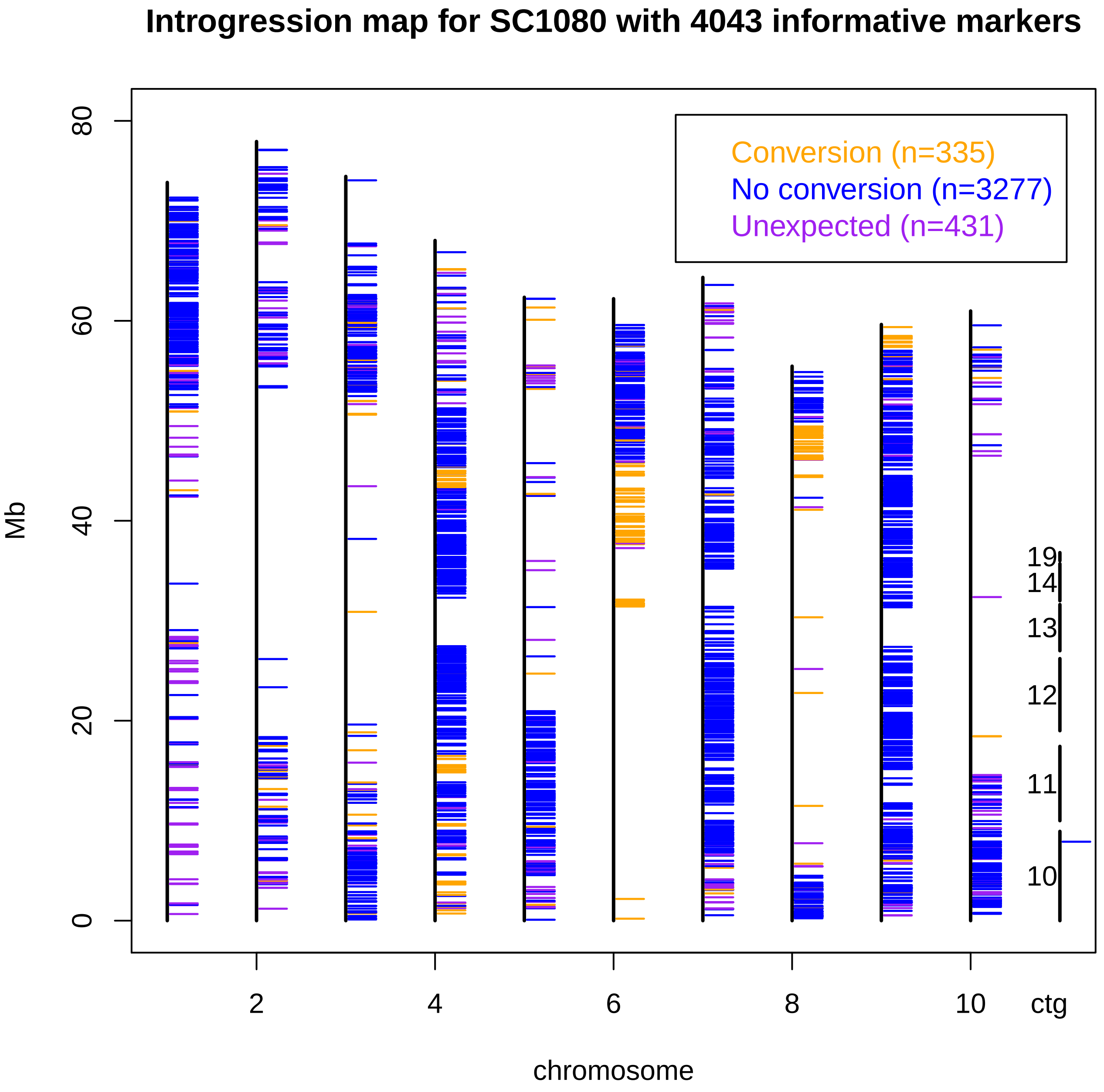
Introgression map for SC1080 with 4043 informative markers
Conversion (n=335)
No conversion (n=3277)
Unexpected (n=431)
Mb
0
20
40
60
80
chromosome
2
4
6
8
10
ctg
19
14
13
12
11
10

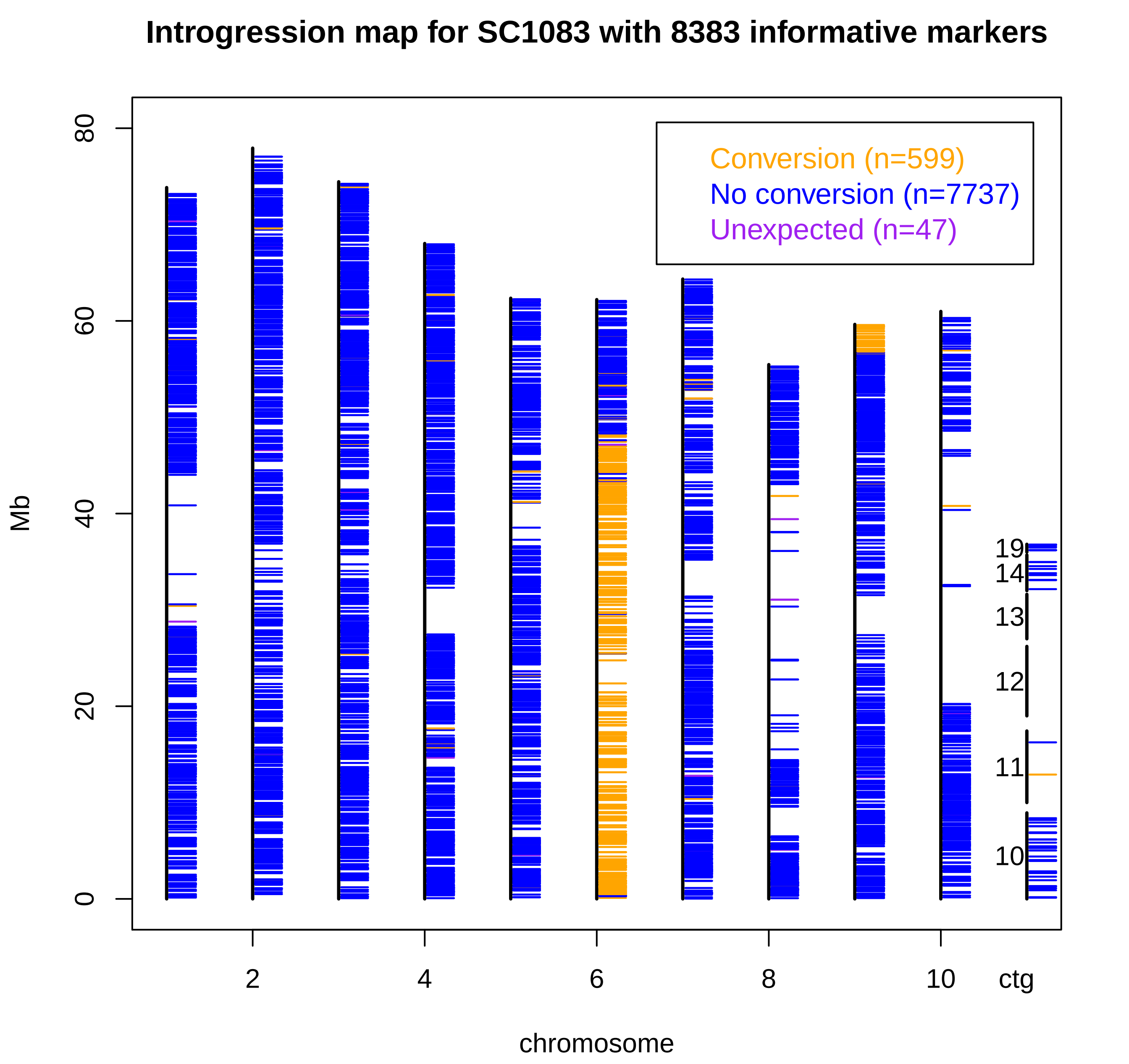

Introgression map for SC1083 with 8383 informative markers
Conversion (n=599)
No conversion (n=7737)
Unexpected (n=47)
Mb
0
20
40
60
80
chromosome
2
4
6
8
10
ctg
19
14
13
12
11
10

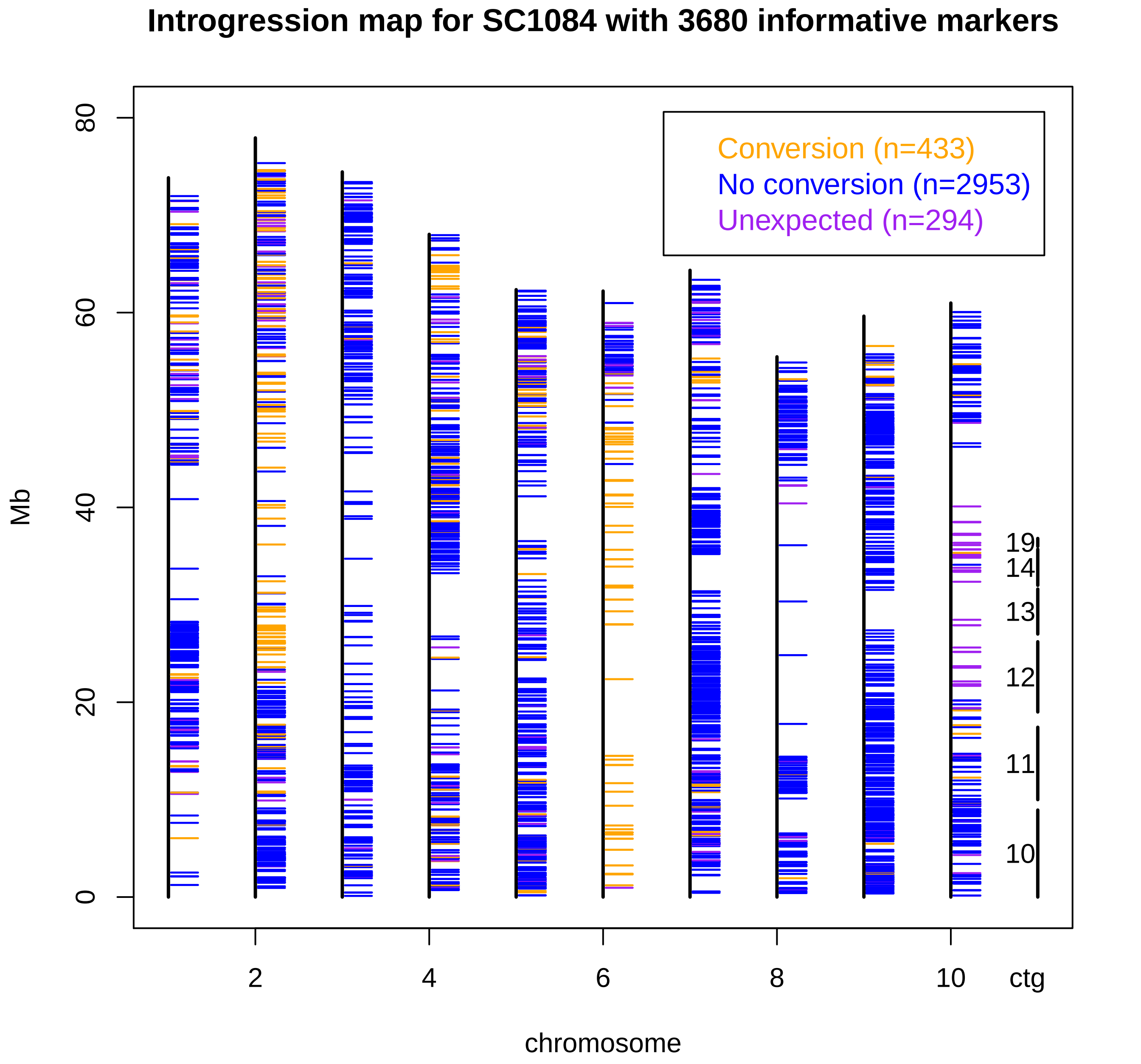

Introgression map for SC1084 with 3680 informative markers
Conversion (n=433)
No conversion (n=2953)
Unexpected (n=294)
Mb
0
20
40
60
80
chromosome
2
4
6
8
10
ctg
19
14
13
12
11
10

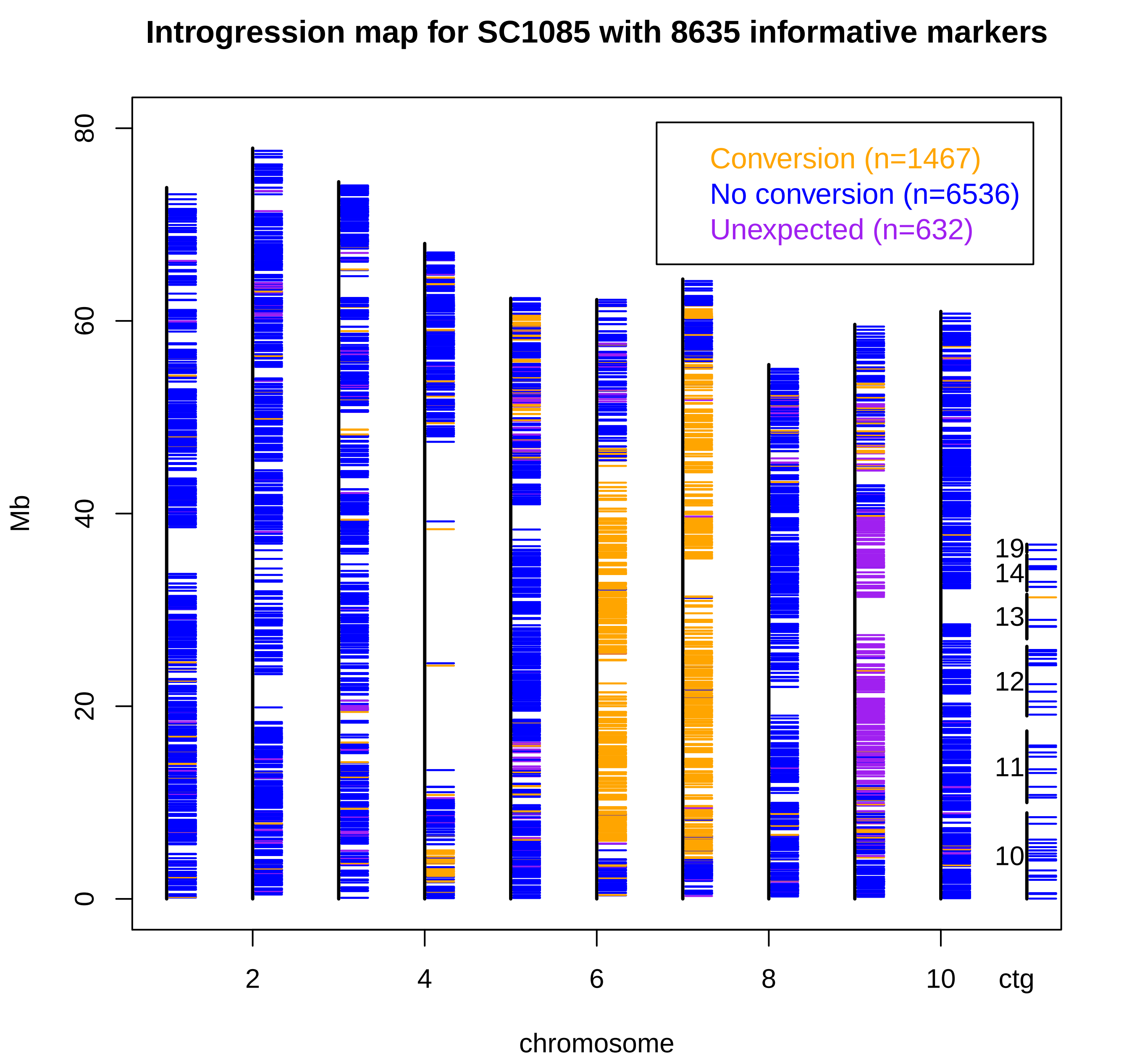

Introgression map for SC1085 with 8635 informative markers
Conversion (n=1467)
No conversion (n=6536)
Unexpected (n=632)
Mb
0
20
40
60
80
chromosome
2
4
6
8
10
ctg
19
14
13
12
11
10

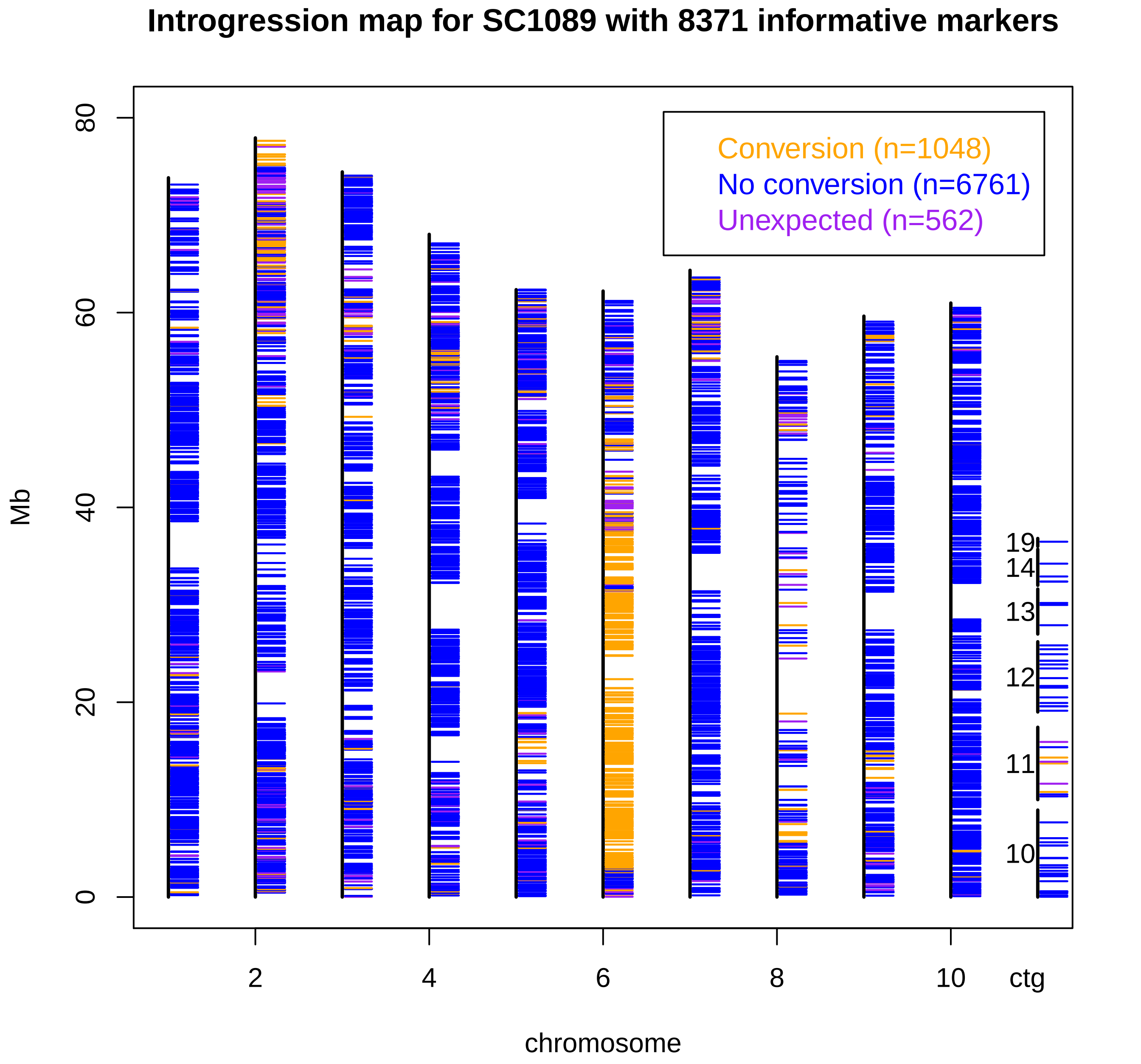

Introgression map for SC1089 with 8371 informative markers
Conversion (n=1048)
No conversion (n=6761)
Unexpected (n=562)
Mb
0
20
40
60
80
2
4
6
8
10
ctg
19
14
13
12
11
10
chromosome

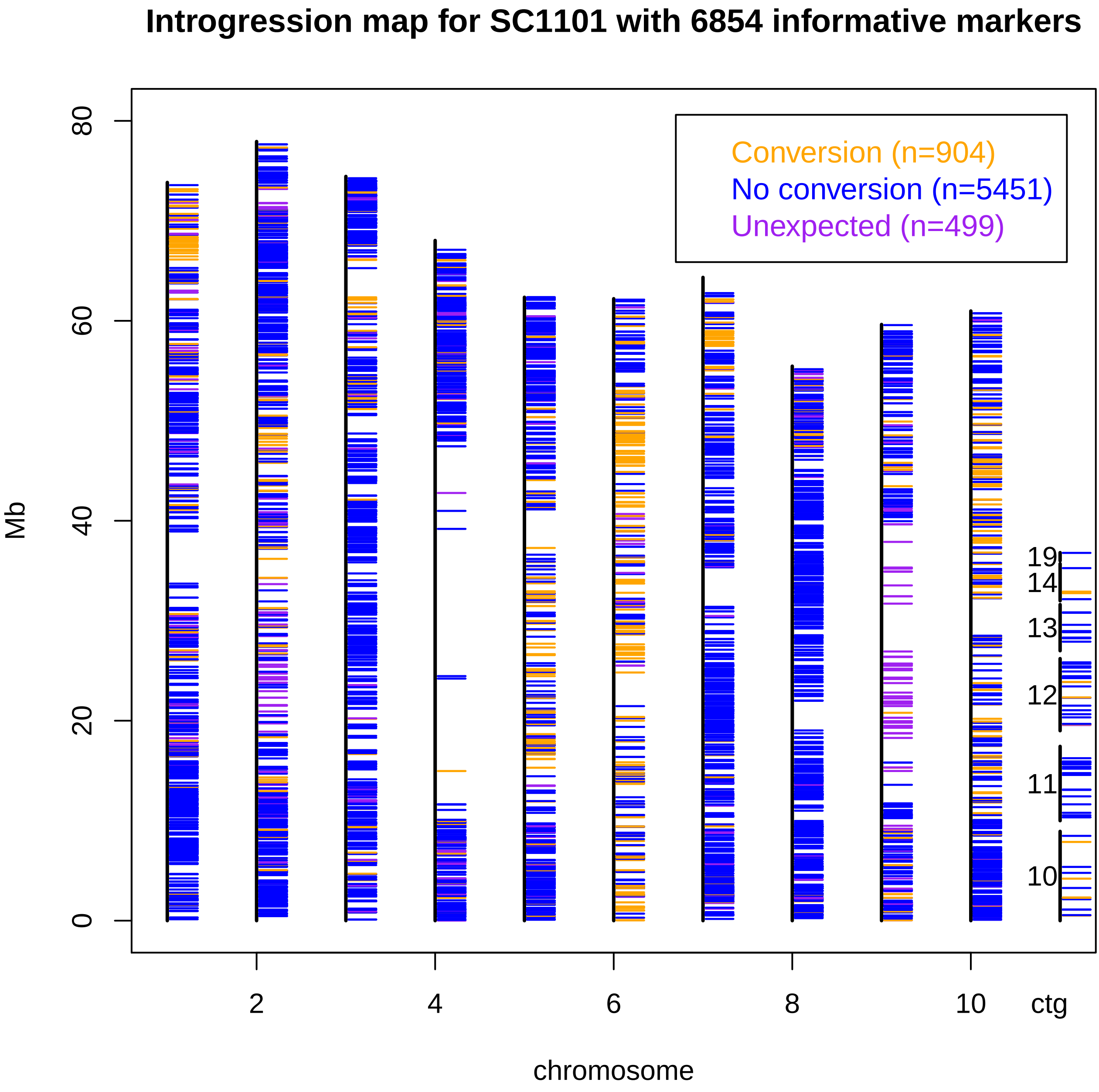

Introgression map for SC1101 with 6854 informative markers
Conversion (n=904)
No conversion (n=5451)
Unexpected (n=499)
Mb
0
20
40
60
80
chromosome
2
4
6
8
10
ctg
19
14
13
12
11
10

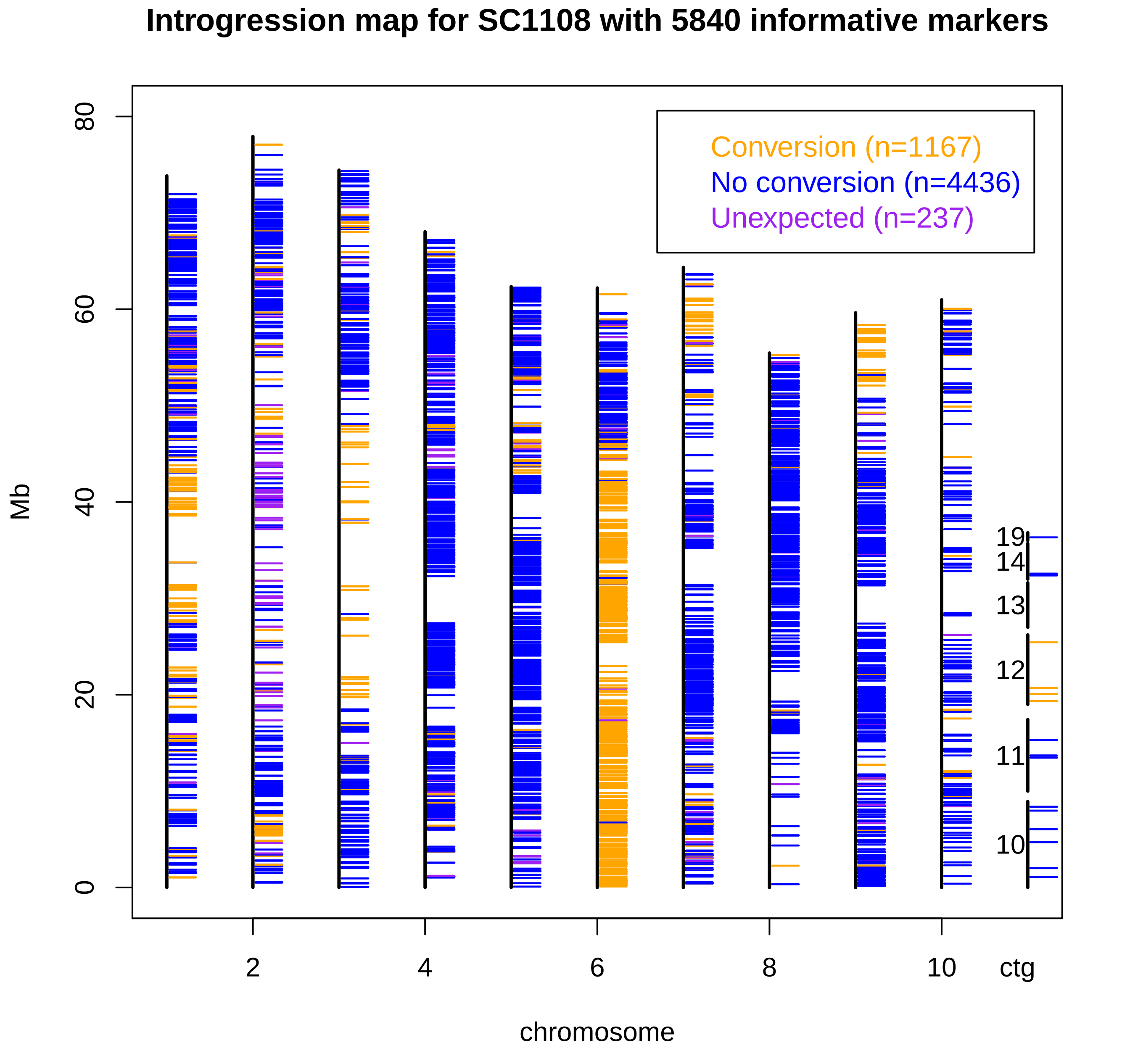

Introgression map for SC1108 with 5840 informative markers
Conversion (n=1167)
No conversion (n=4436)
Unexpected (n=237)
Mb
0
20
40
60
80
chromosome
2
4
6
8
10
ctg
19
14
13
12
11
10

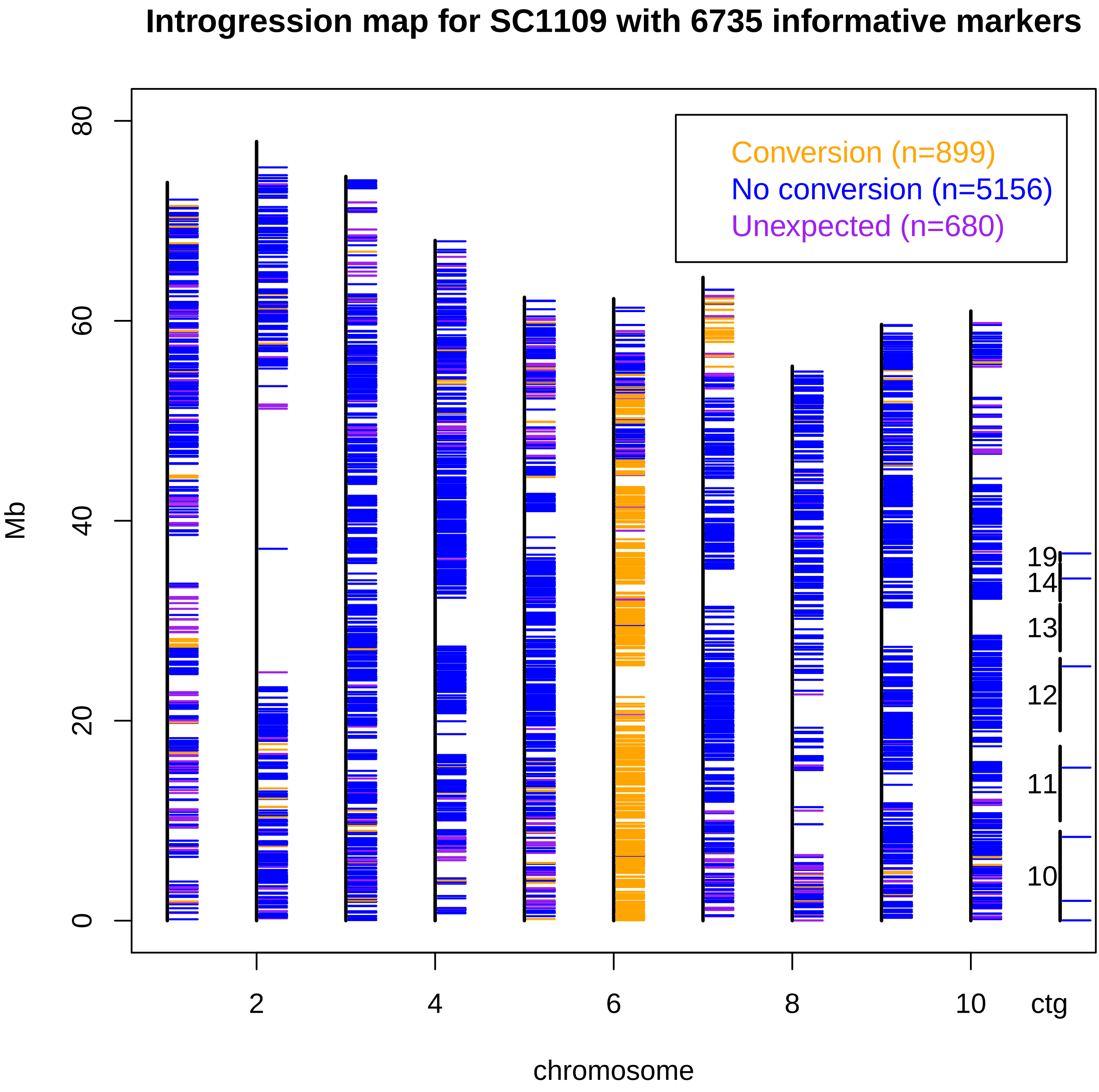

Introgression map for SC1109 with 6735 informative markers
Conversion (n=899)
No conversion (n=5156)
Unexpected (n=680)
Mb
0
20
40
60
80
chromosome
2
4
6
8
10
ctg
19
14
13
12
11
10

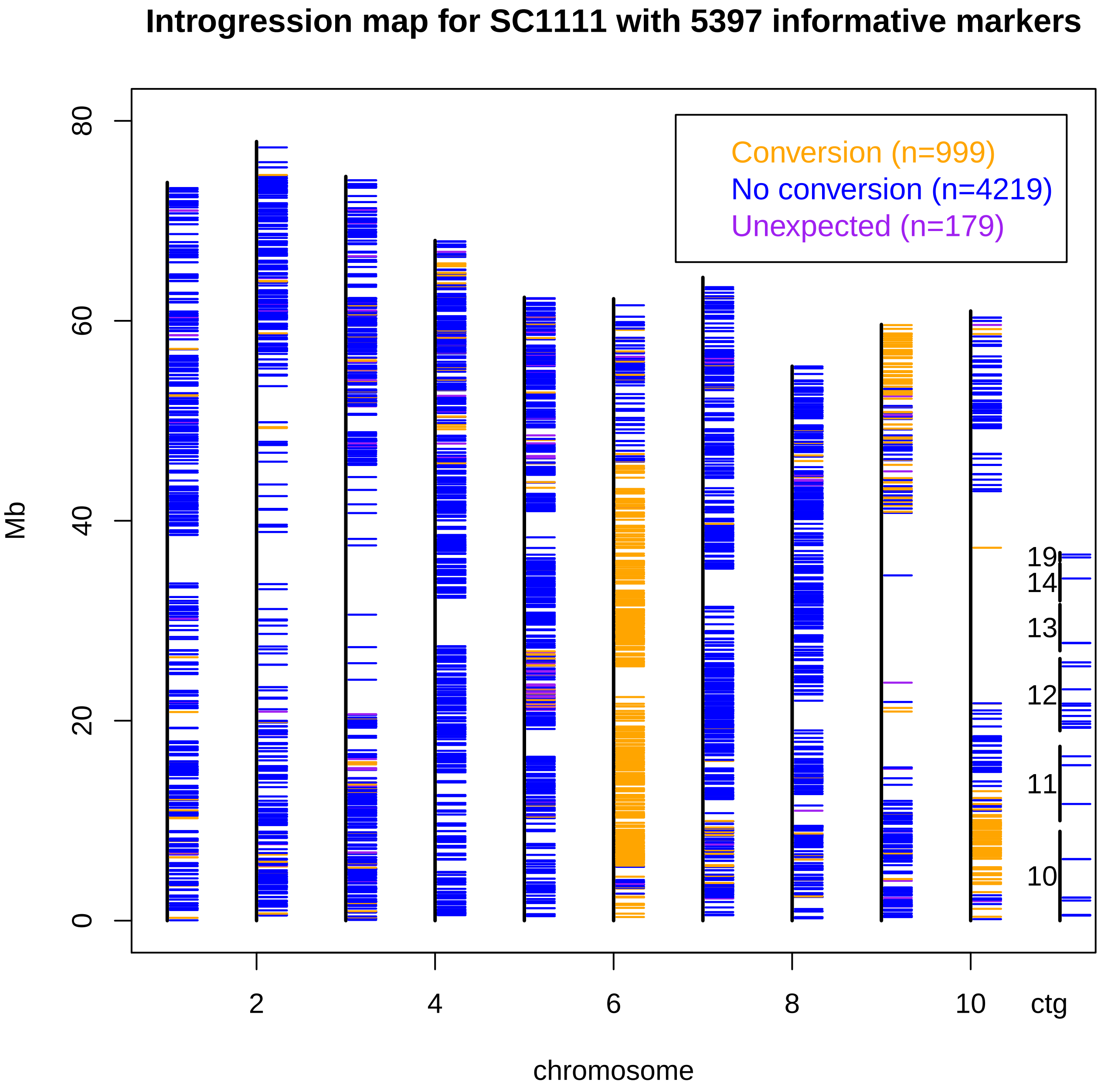
Introgression map for SC1111 with 5397 informative markers
Conversion (n=999)
No conversion (n=4219)
Unexpected (n=179)
Mb
0
20
40
60
80
chromosome
2
4
6
8
10
ctg
19
14
13
12
11
10

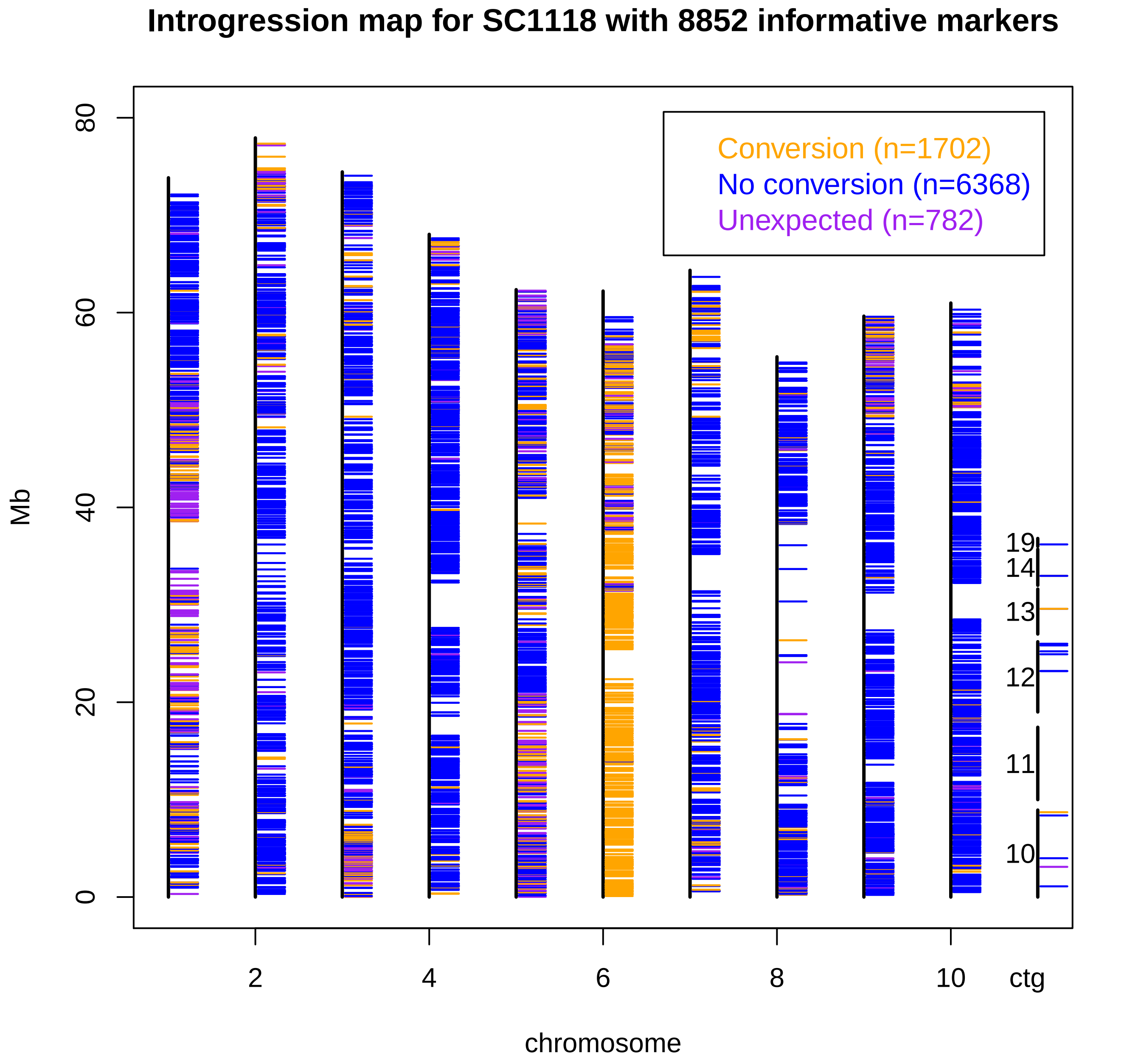

Introgression map for SC1118 with 8852 informative markers
Conversion (n=1702)
No conversion (n=6368)
Unexpected (n=782)
Mb
0
20
40
60
80
chromosome
2
4
6
8
10
ctg
19
14
13
12
11
10

Introgression map for SC1124 with 5838 informative markers

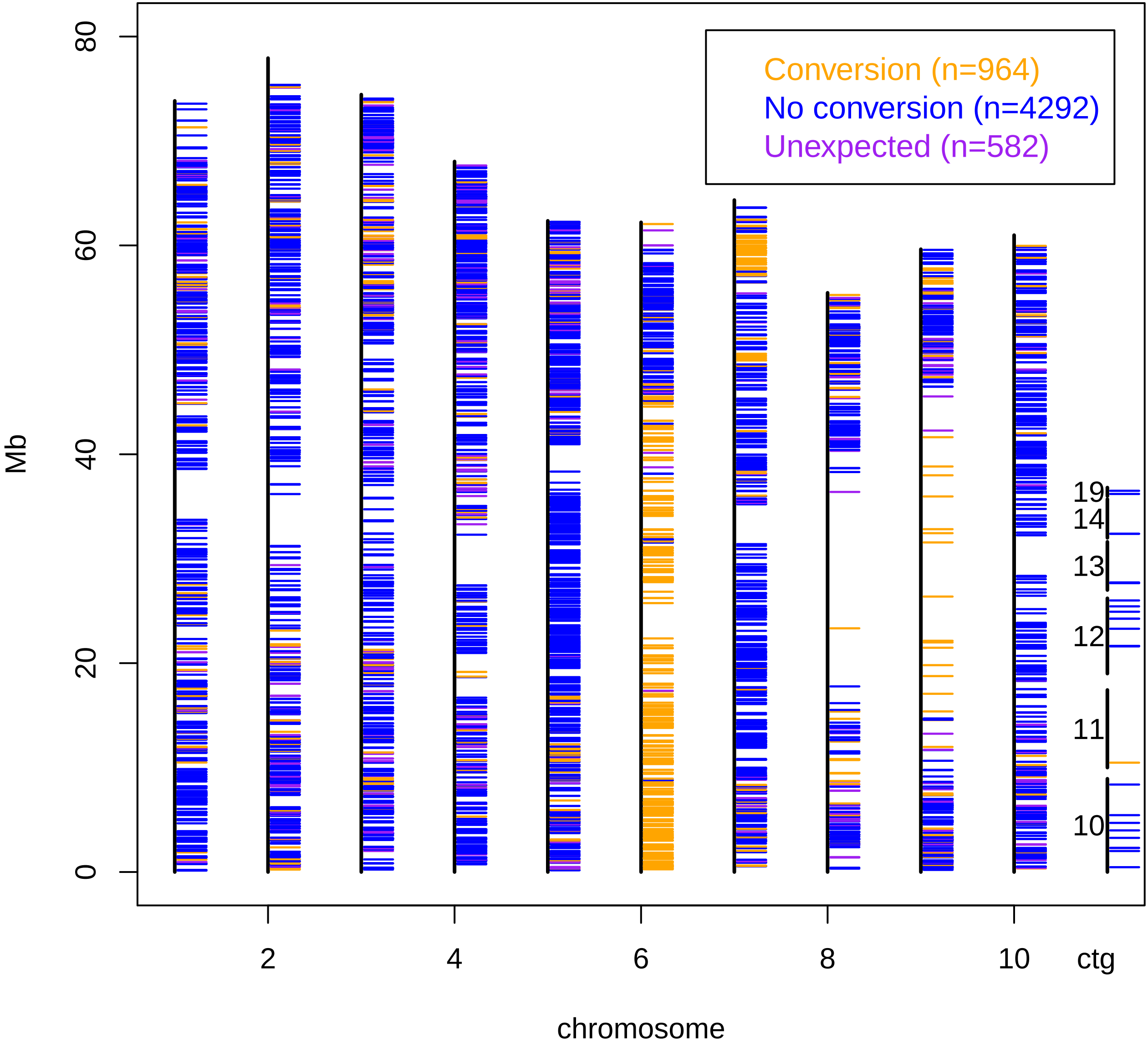

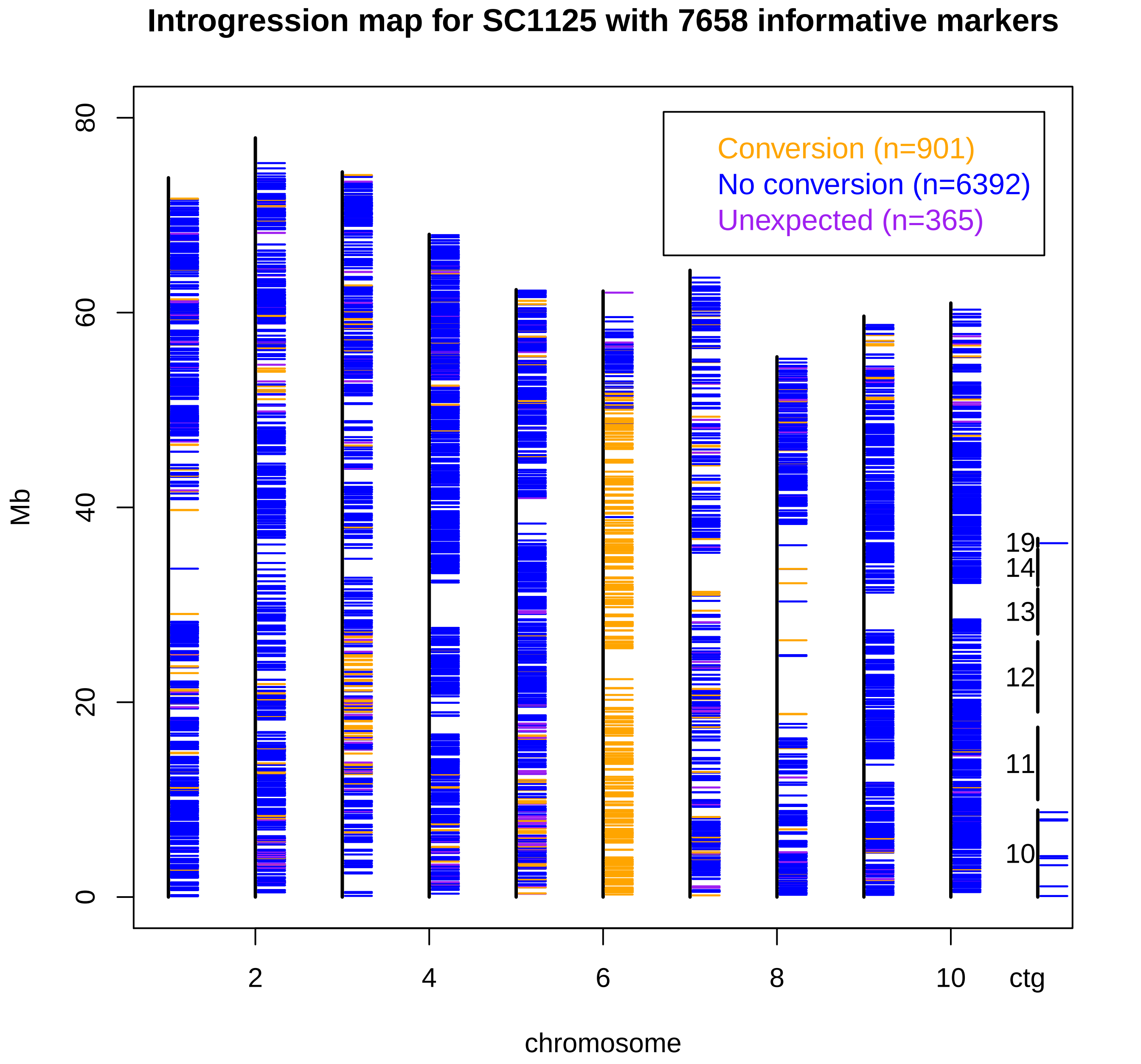

Introgression map for SC1125 with 7658 informative markers
Conversion (n=901)
No conversion (n=6392)
Unexpected (n=365)
Mb
0
20
40
60
80
chromosome
2
4
6
8
10
ctg
19
14
13
12
11
10

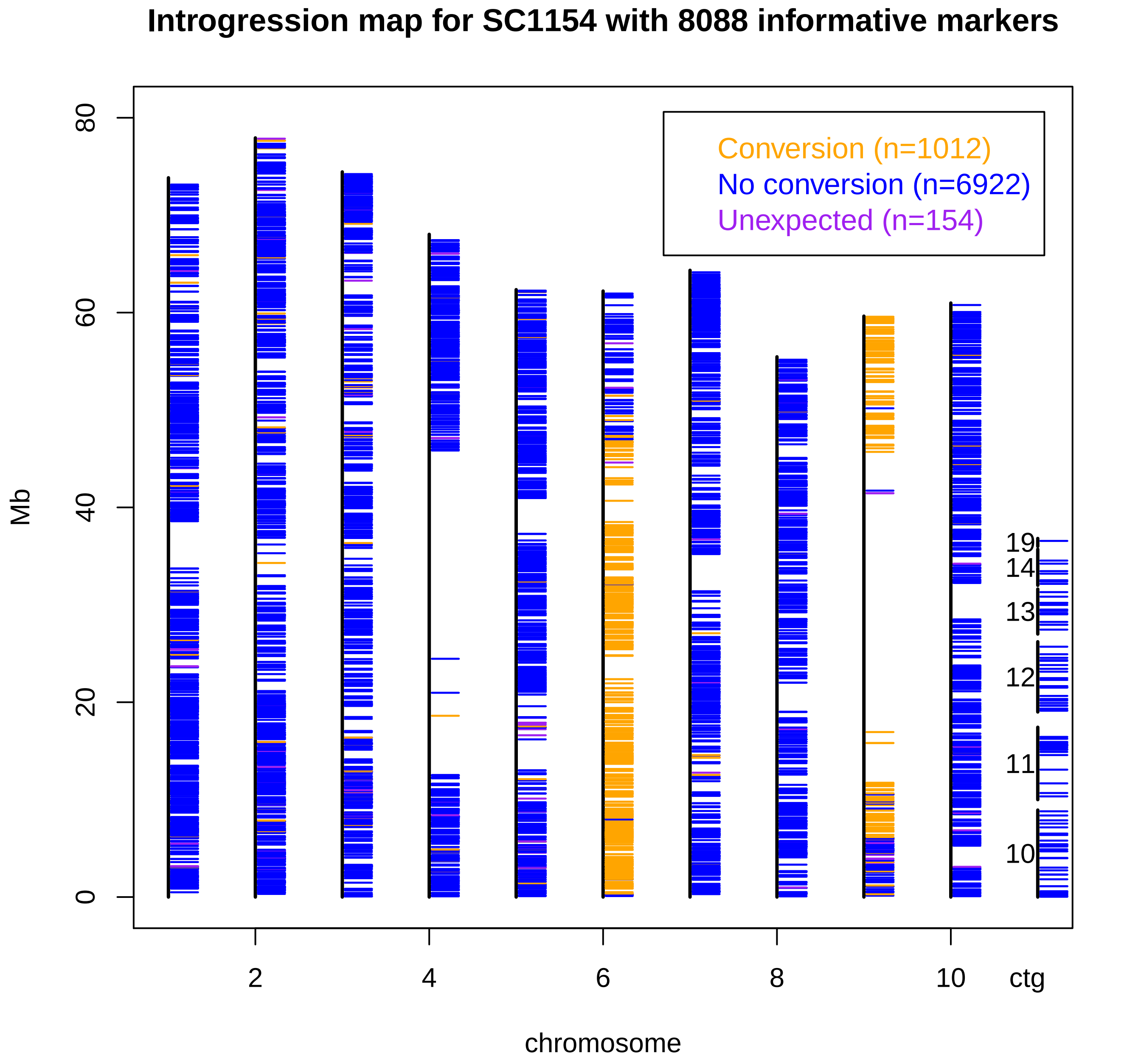

Introgression map for SC1154 with 8088 informative markers
Conversion (n=1012)
No conversion (n=6922)
Unexpected (n=154)
Mb
0
20
40
60
80
chromosome
2
4
6
8
10
ctg
19
14
13
12
11
10

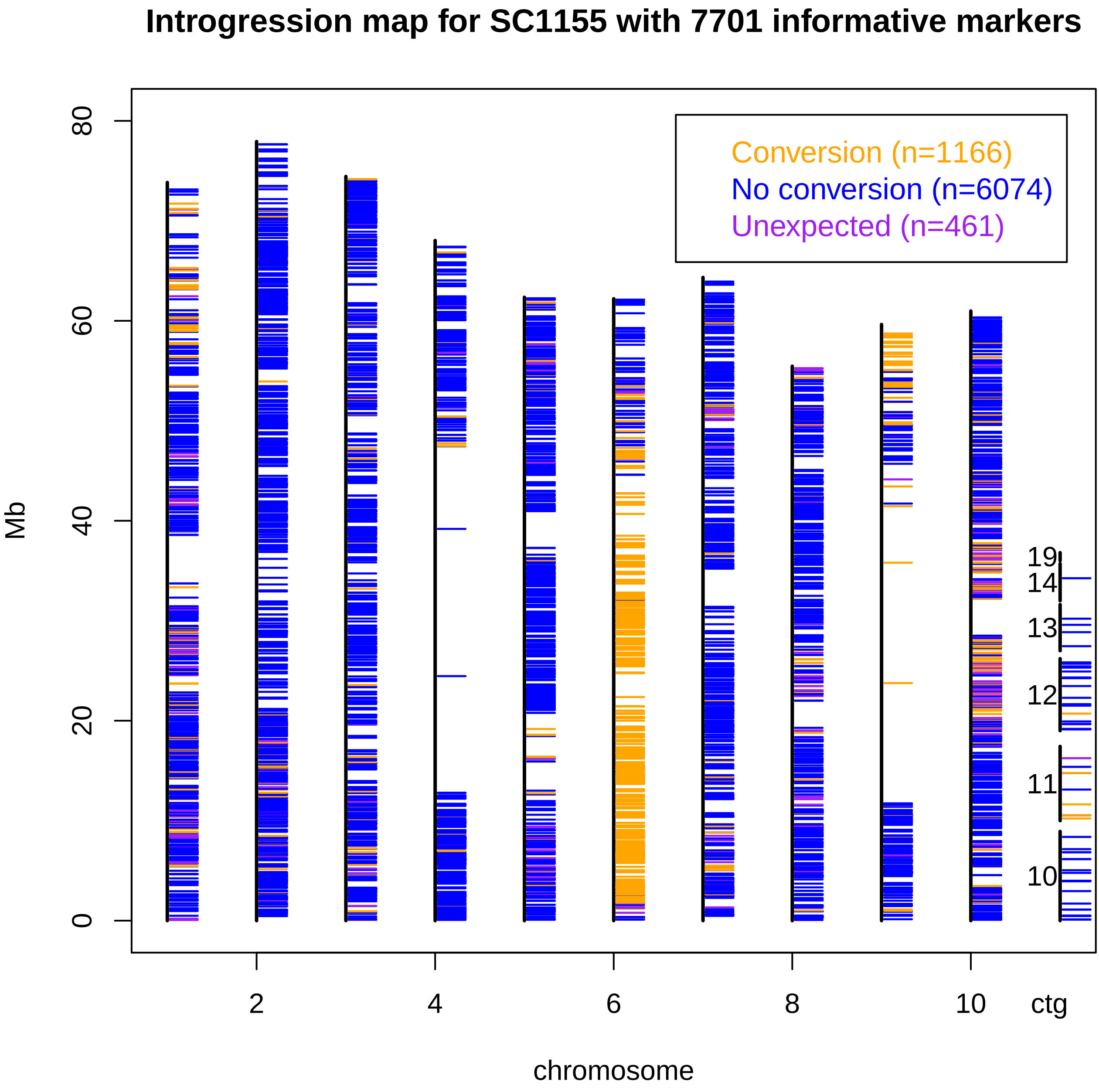
Introgression map for SC1155 with 7701 informative markers
Conversion (n=1166)
No conversion (n=6074)
Unexpected (n=461)
Mb
0
20
40
60
80
chromosome
2
4
6
8
10
ctg
19
14
13
12
11
10

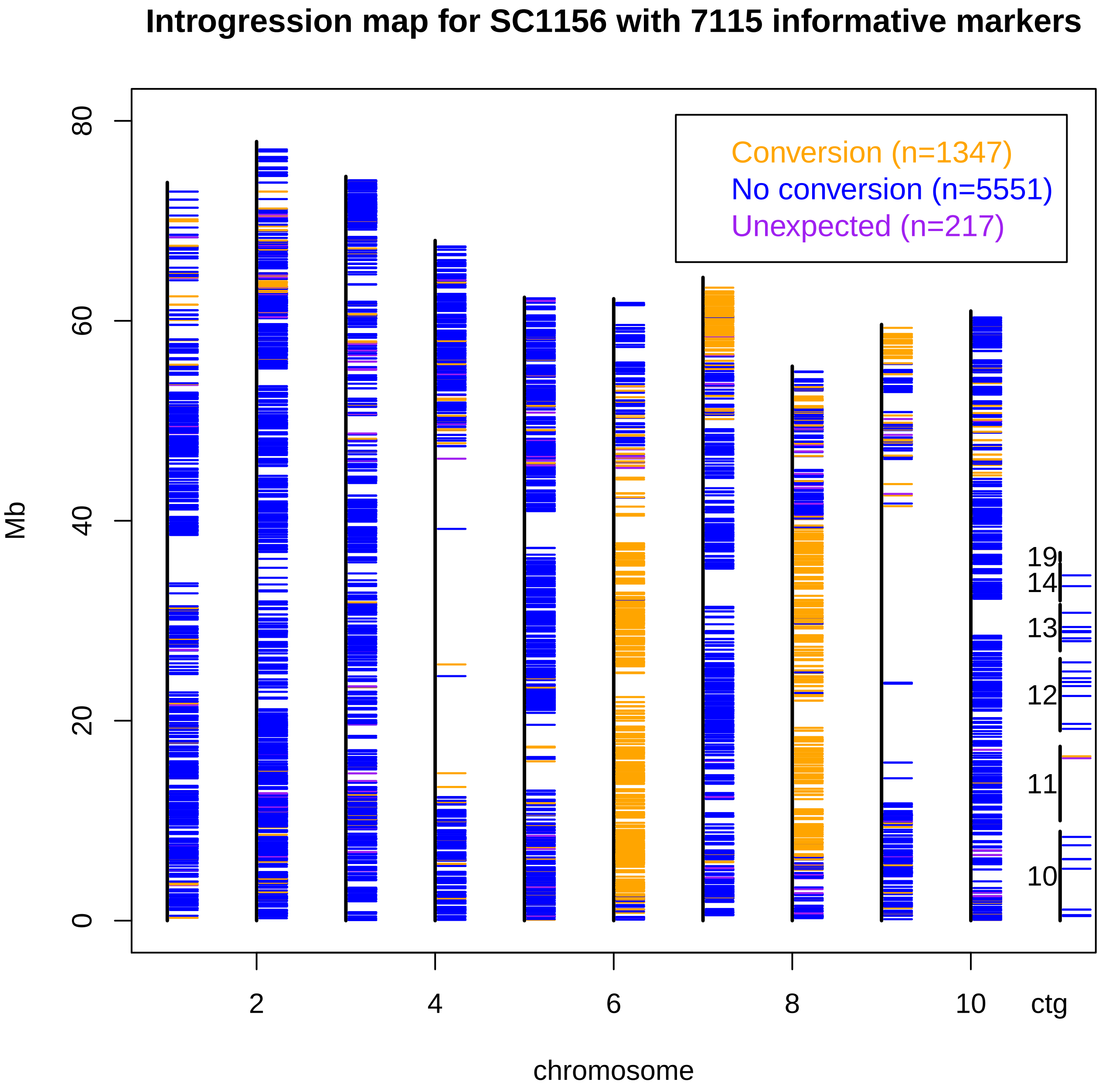

Introgression map for SC1156 with 7115 informative markers
Conversion (n=1347)
No conversion (n=5551)
Unexpected (n=217)
Mb
0
20
40
60
80
chromosome
2
4
6
8
10
ctg
19
14
13
12
11
10

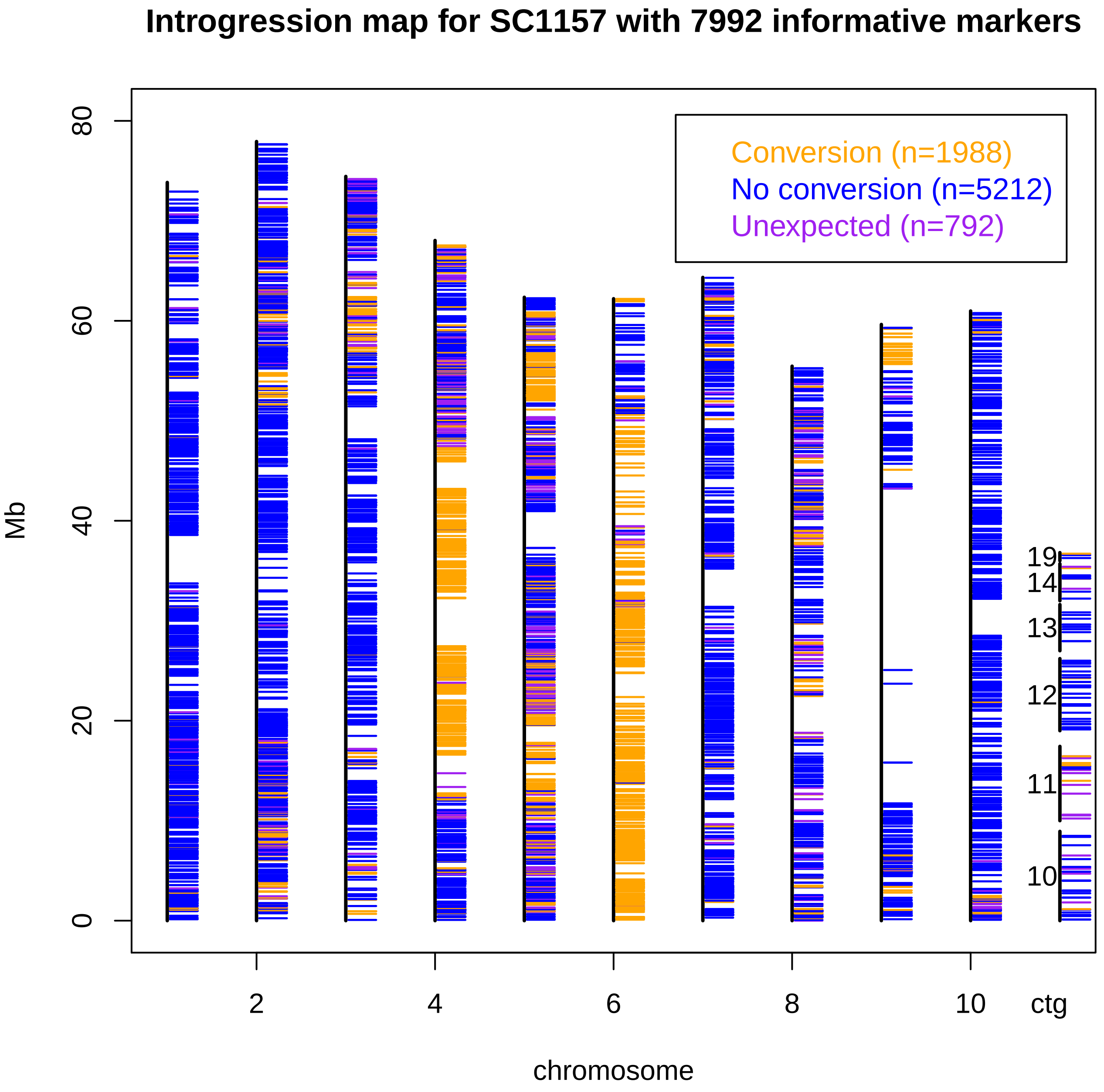
Introgression map for SC1157 with 7992 informative markers
Conversion (n=1988)
No conversion (n=5212)
Unexpected (n=792)
Mb
0
20
40
60
80
chromosome
2
4
6
8
10
ctg
19
14
13
12
11
10

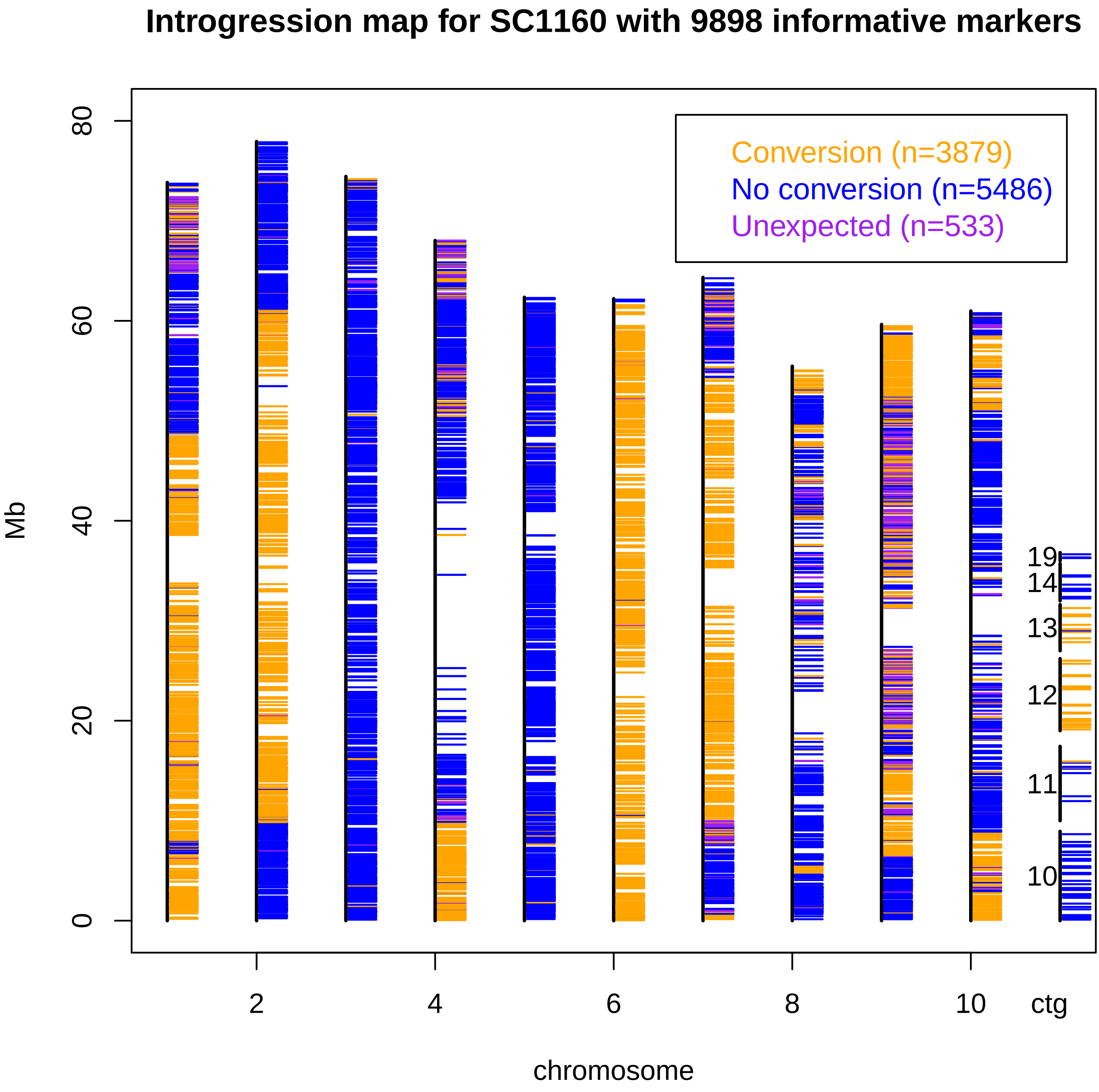

Introgression map for SC1160 with 9898 informative markers
Conversion (n=3879)
No conversion (n=5486)
Unexpected (n=533)
Mb
0
20
40
60
80
chromosome
2
4
6
8
10
ctg
19
14
13
12
11
10

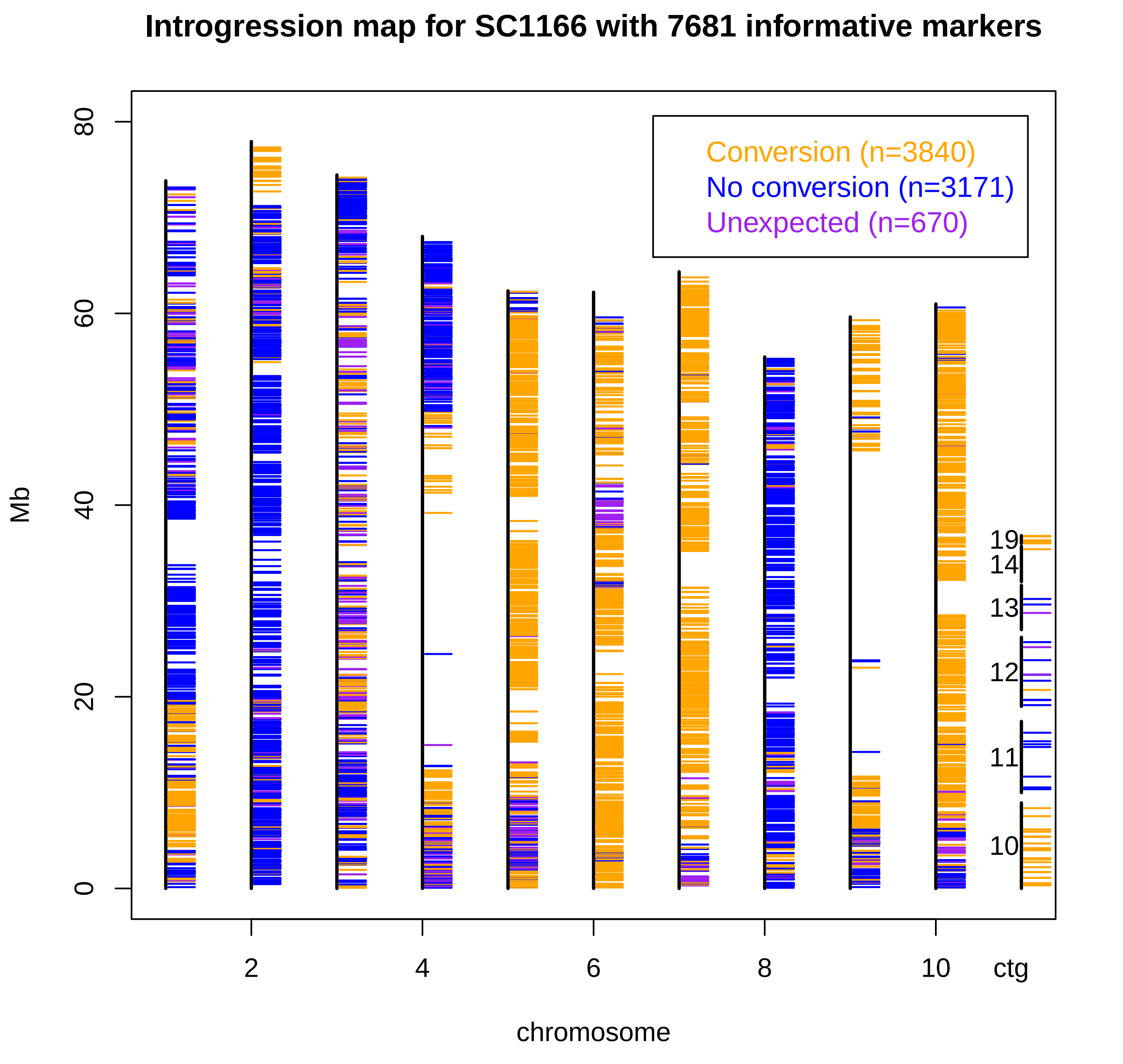
Introgression map for SC1166 with 7681 informative markers
Conversion (n=3840)
No conversion (n=3171)
Unexpected (n=670)
Mb
0
20
40
60
80
2
4
6
8
10
ctg
19
14
13
12
11
10
chromosome

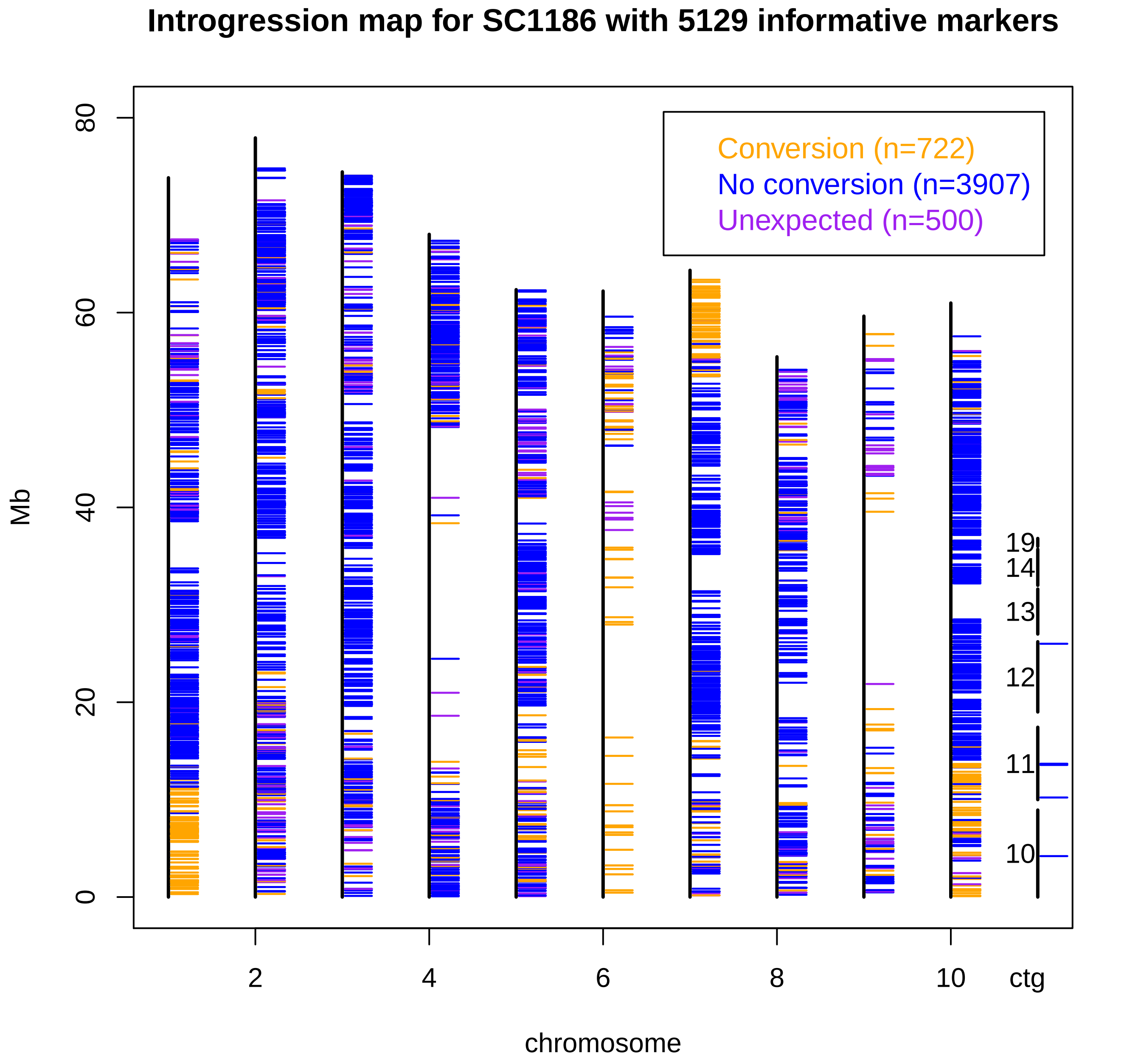

Introgression map for SC1186 with 5129 informative markers
Conversion (n=722)
No conversion (n=3907)
Unexpected (n=500)
Mb
0
20
40
60
80
chromosome
2
4
6
8
10
ctg
19
14
13
12
11
10

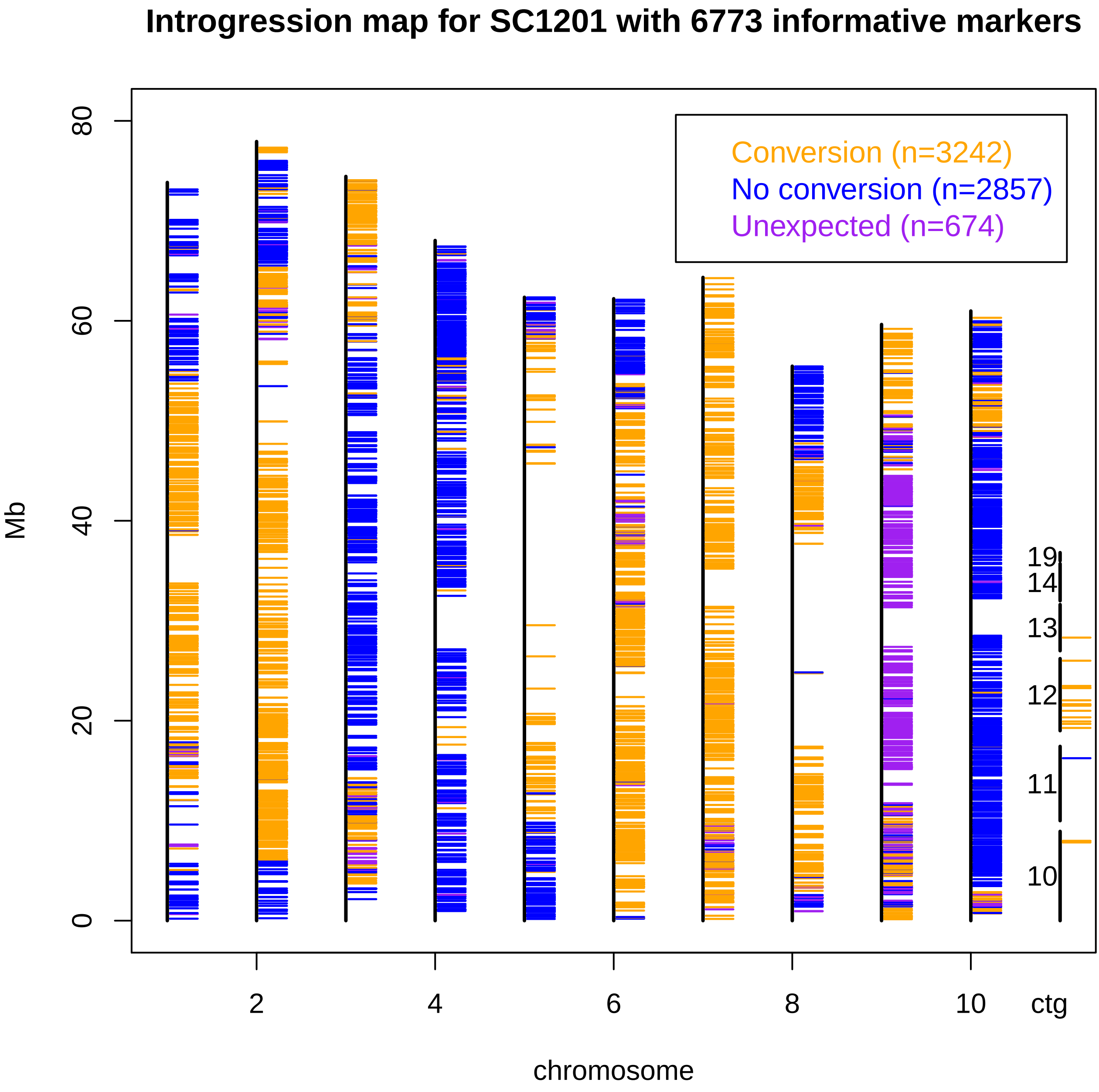
Introgression map for SC1201 with 6773 informative markers
Conversion (n=3242)
No conversion (n=2857)
Unexpected (n=674)
Mb
0
20
40
60
80
2
4
6
8
10
ctg
chromosome
19
14
13
12
11
10

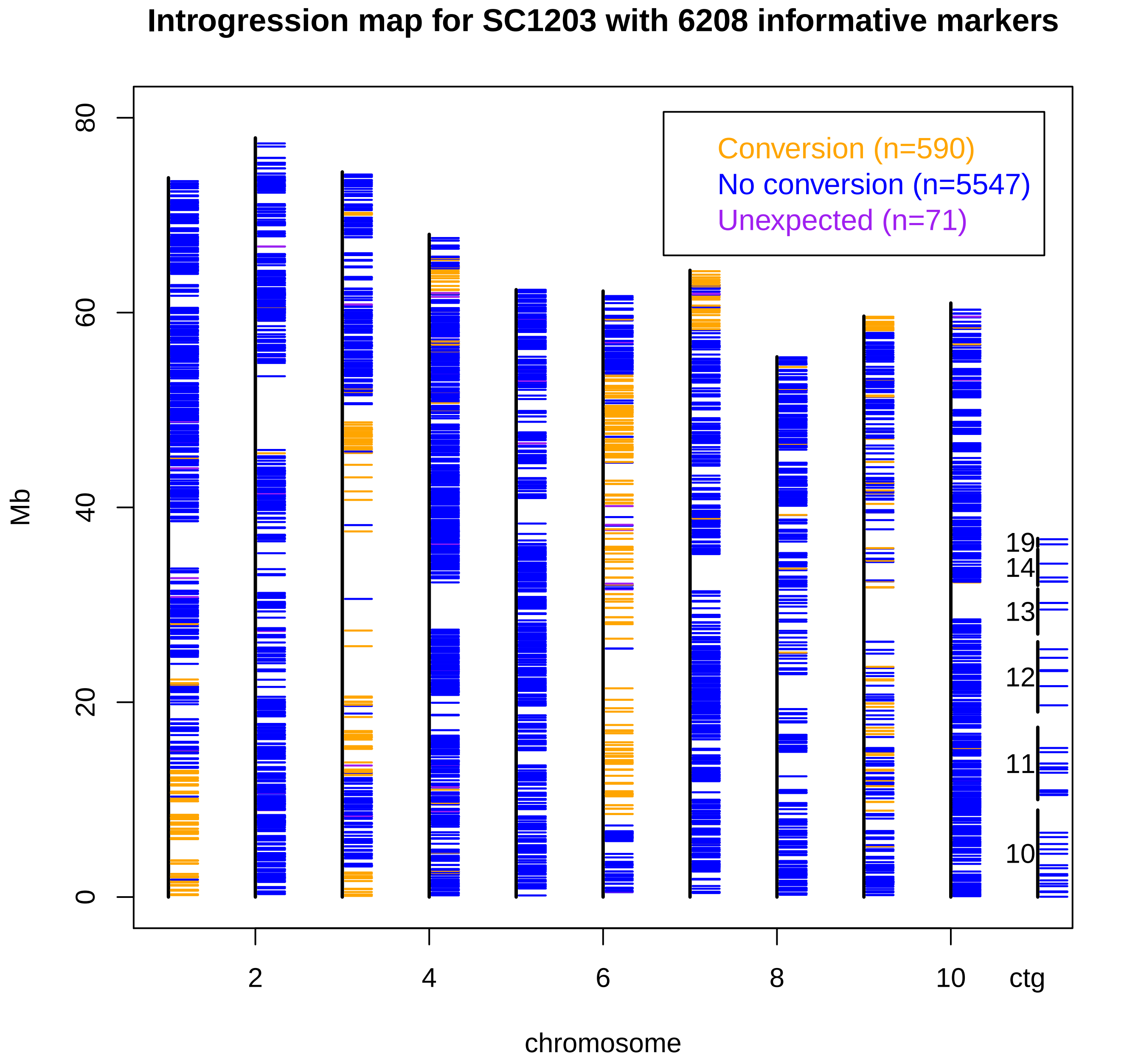
Introgression map for SC1203 with 6208 informative markers
Conversion (n=590)
No conversion (n=5547)
Unexpected (n=71)
Mb
0
20
40
60
80
chromosome
2
4
6
8
10
ctg
19
14
13
12
11
10

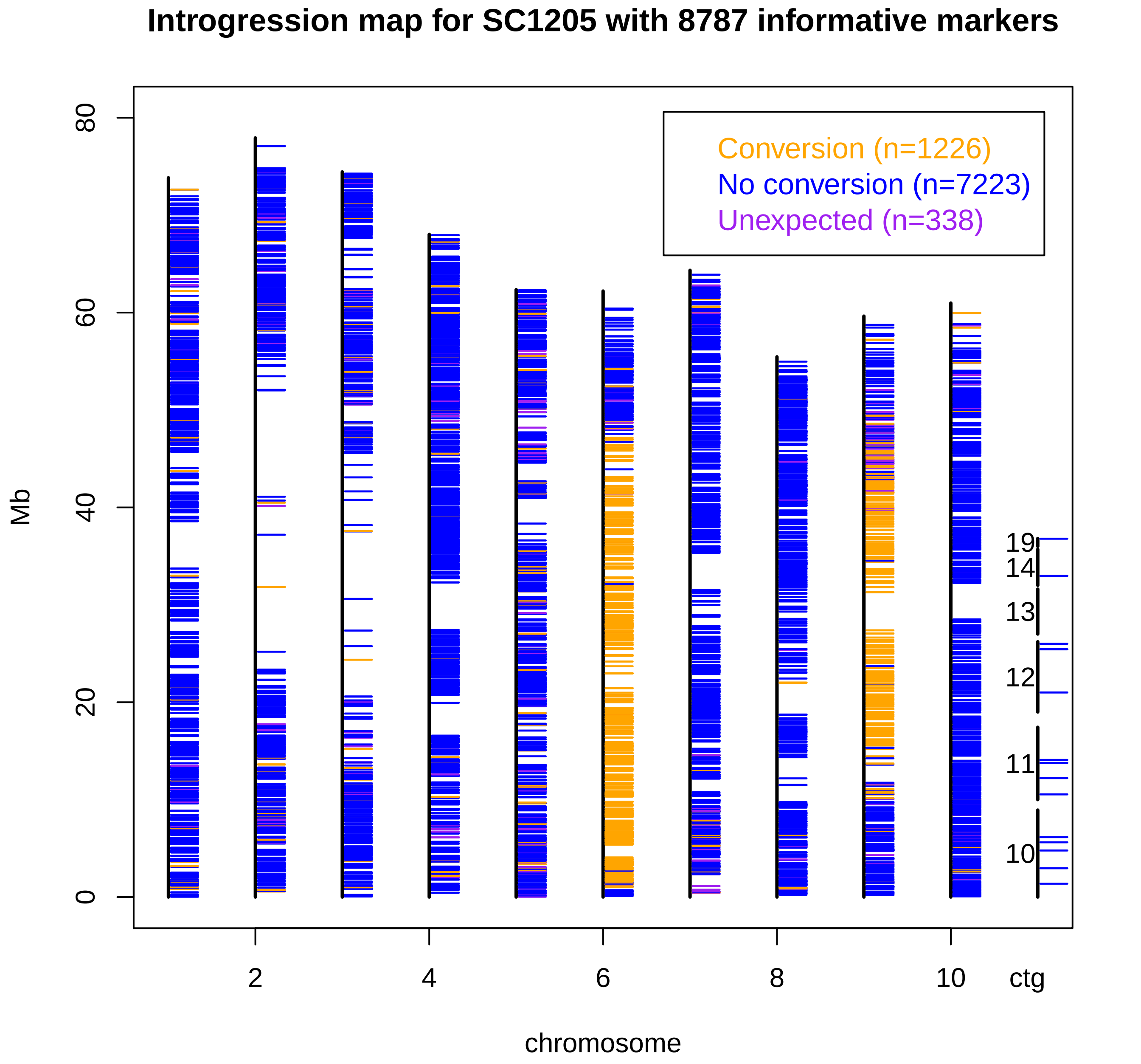

Introgression map for SC1205 with 8787 informative markers
Conversion (n=1226)
No conversion (n=7223)
Unexpected (n=338)
Mb
0
20
40
60
80
chromosome
2
4
6
8
10
ctg
19
14
13
12
11
10

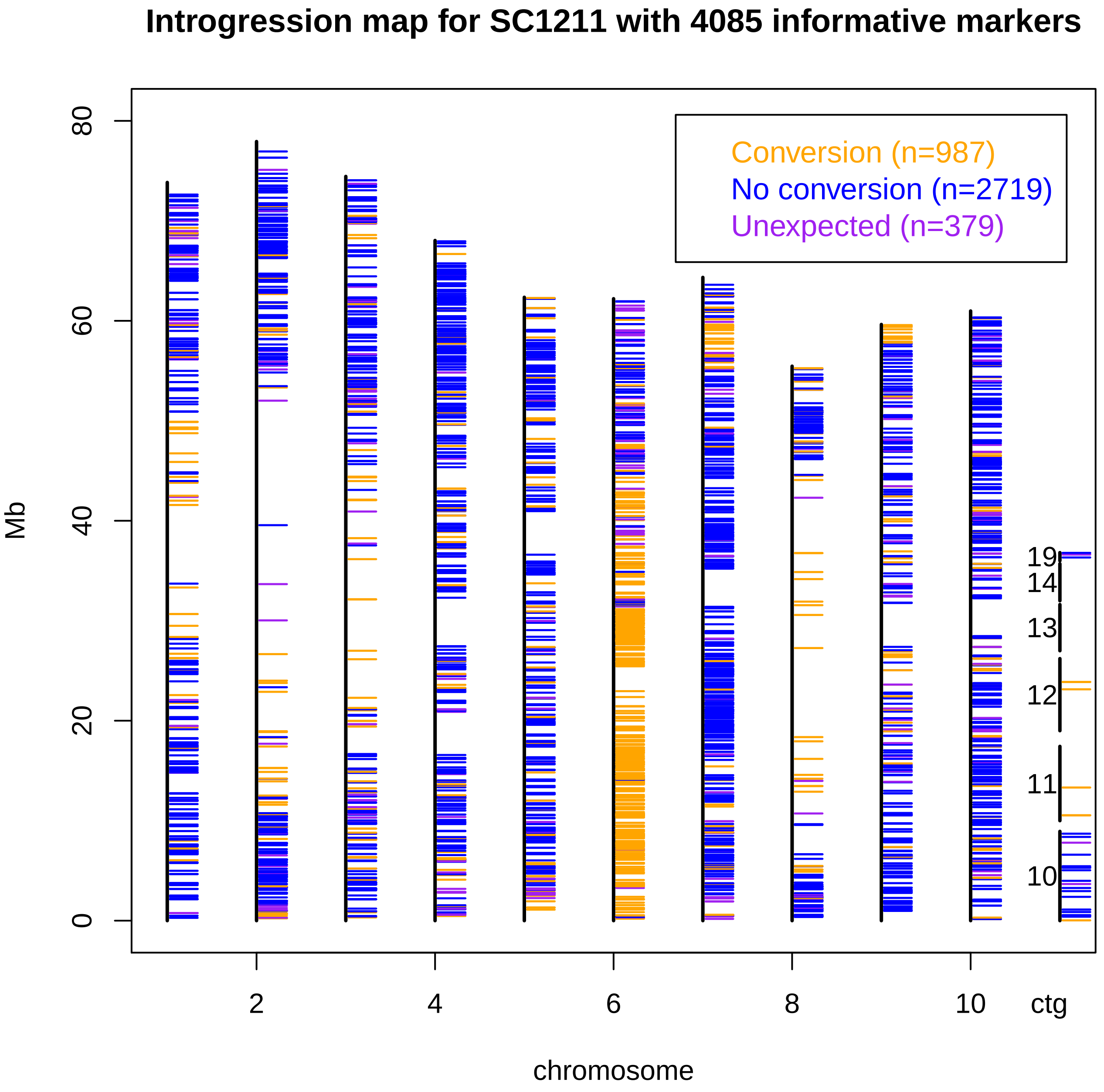

Introgression map for SC1211 with 4085 informative markers
Conversion (n=987)
No conversion (n=2719)
Unexpected (n=379)
Mb
0
20
40
60
80
chromosome
2
4
6
8
10
ctg
19
14
13
12
11
10

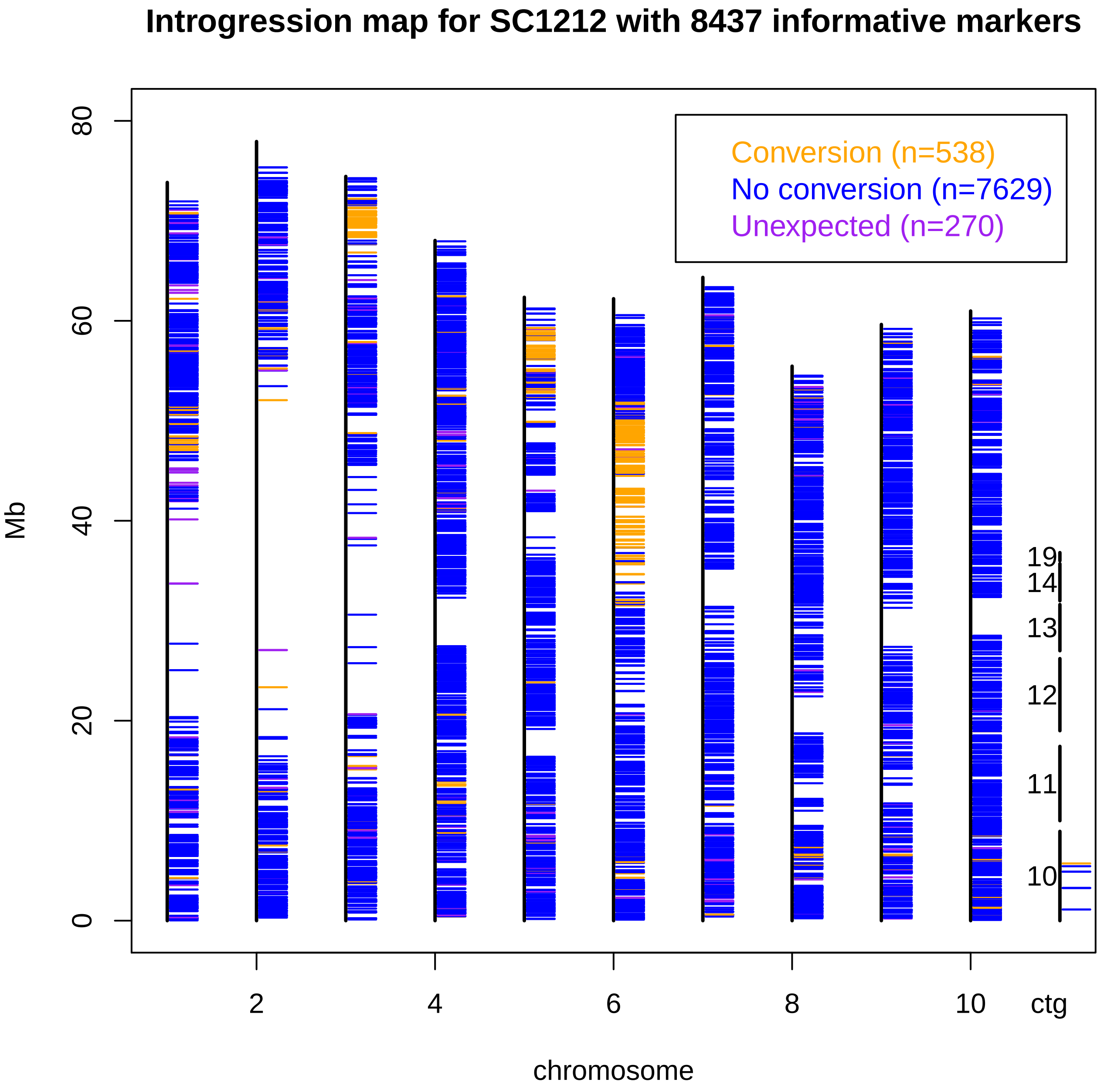

Introgression map for SC1212 with 8437 informative markers
Conversion (n=538)
No conversion (n=7629)
Unexpected (n=270)
Mb
0
20
40
60
80
2
4
6
8
10
ctg
19
14
13
12
11
10
chromosome

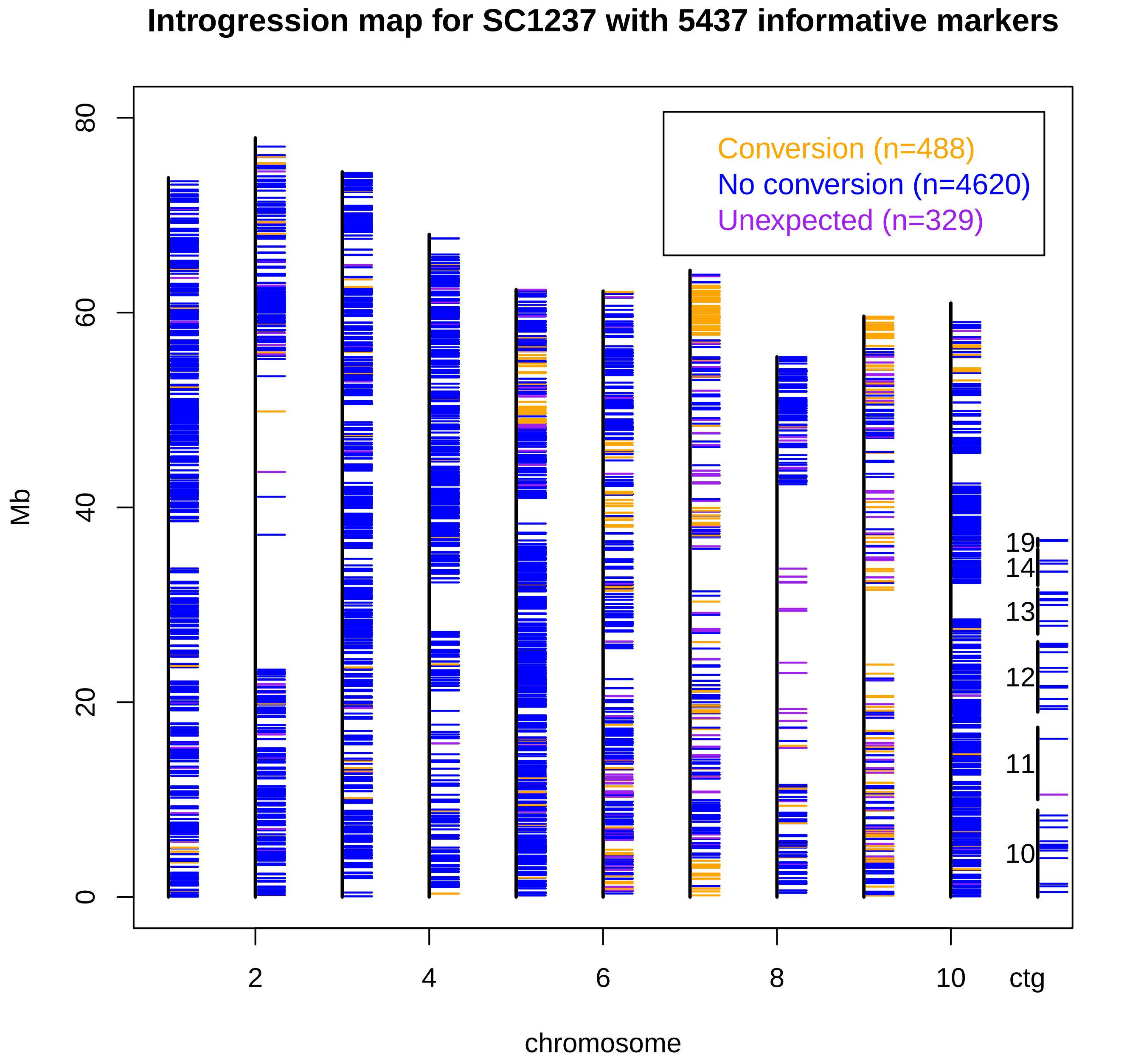
Introgression map for SC1237 with 5437 informative markers
Conversion (n=488)
No conversion (n=4620)
Unexpected (n=329)
Mb
0
20
40
60
80
chromosome
2
4
6
8
10
ctg
19
14
13
12
11
10

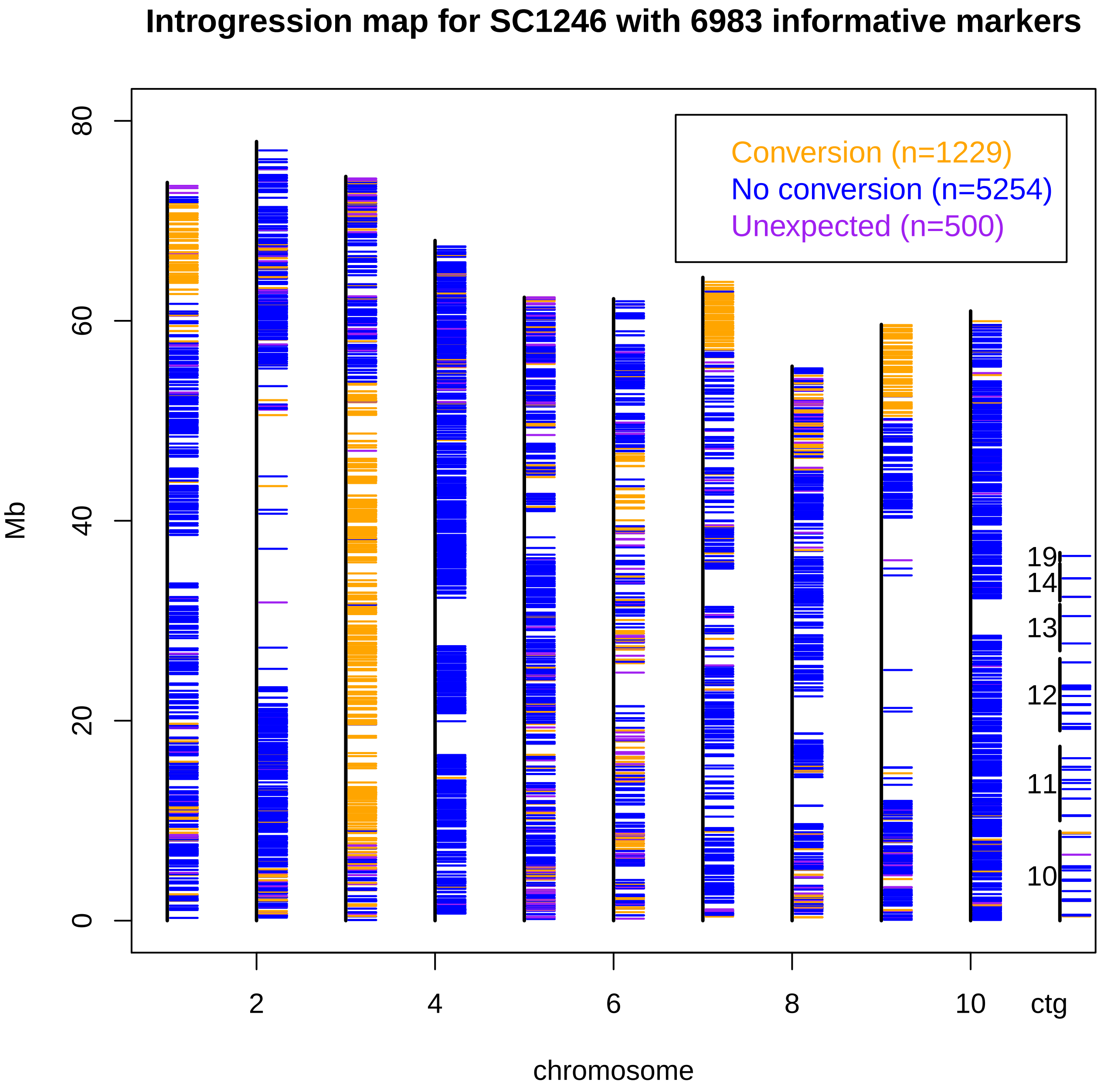

Introgression map for SC1246 with 6983 informative markers
Conversion (n=1229)
No conversion (n=5254)
Unexpected (n=500)
Mb
0
20
40
60
80
chromosome
2
4
6
8
10
ctg
19
14
13
12
11
10

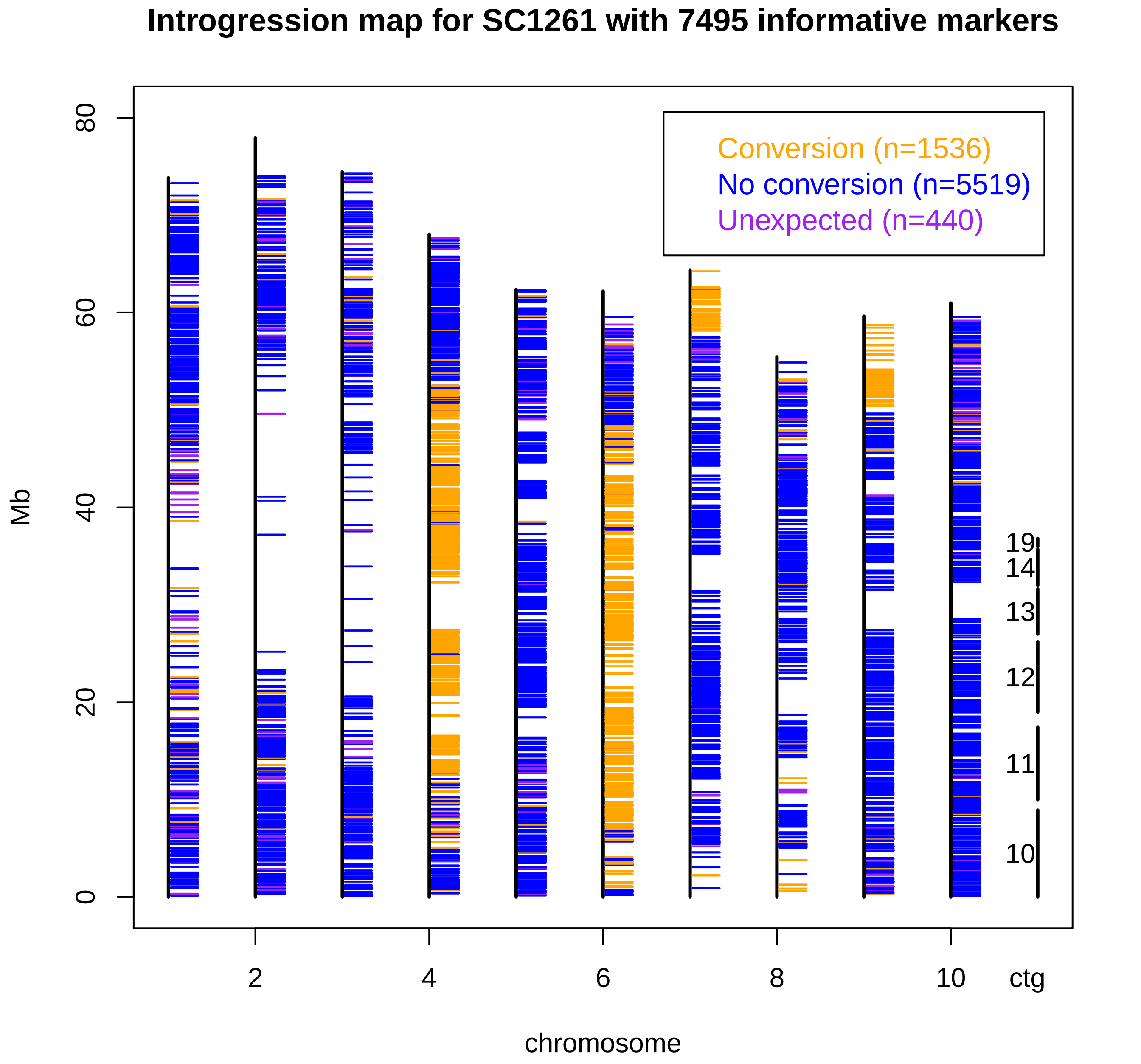
Introgression map for SC1261 with 7495 informative markers
Conversion (n=1536)
No conversion (n=5519)
Unexpected (n=440)
Mb
0
20
40
60
80
chromosome
2
4
6
8
10
ctg
19
14
13
12
11
10

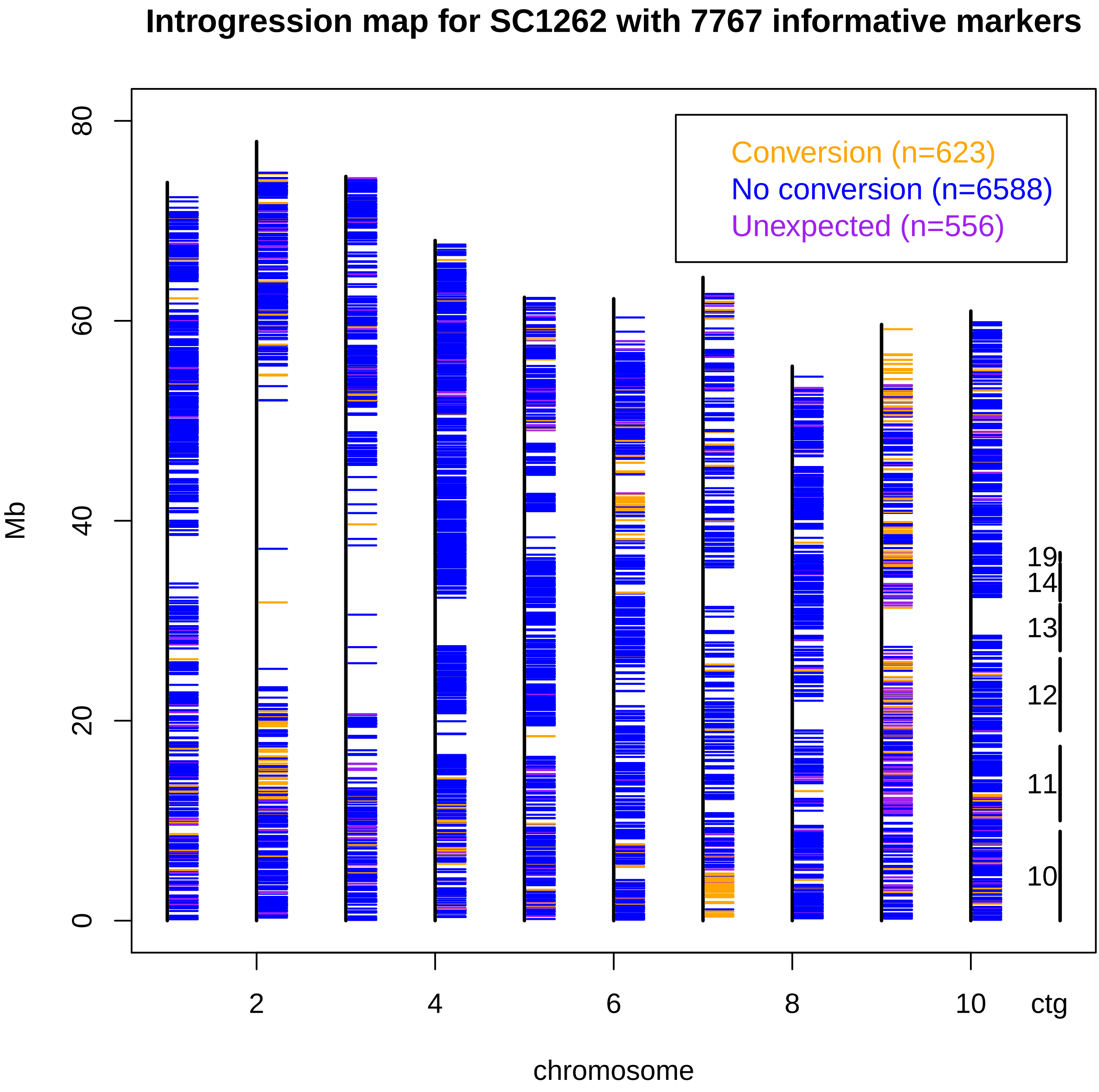
Introgression map for SC1262 with 7767 informative markers
Conversion (n=623)
No conversion (n=6588)
Unexpected (n=556)
Mb
0
20
40
60
80
chromosome
2
4
6
8
10
ctg
19
14
13
12
11
10

Introgression map for SC1271 with 8386 informative markers

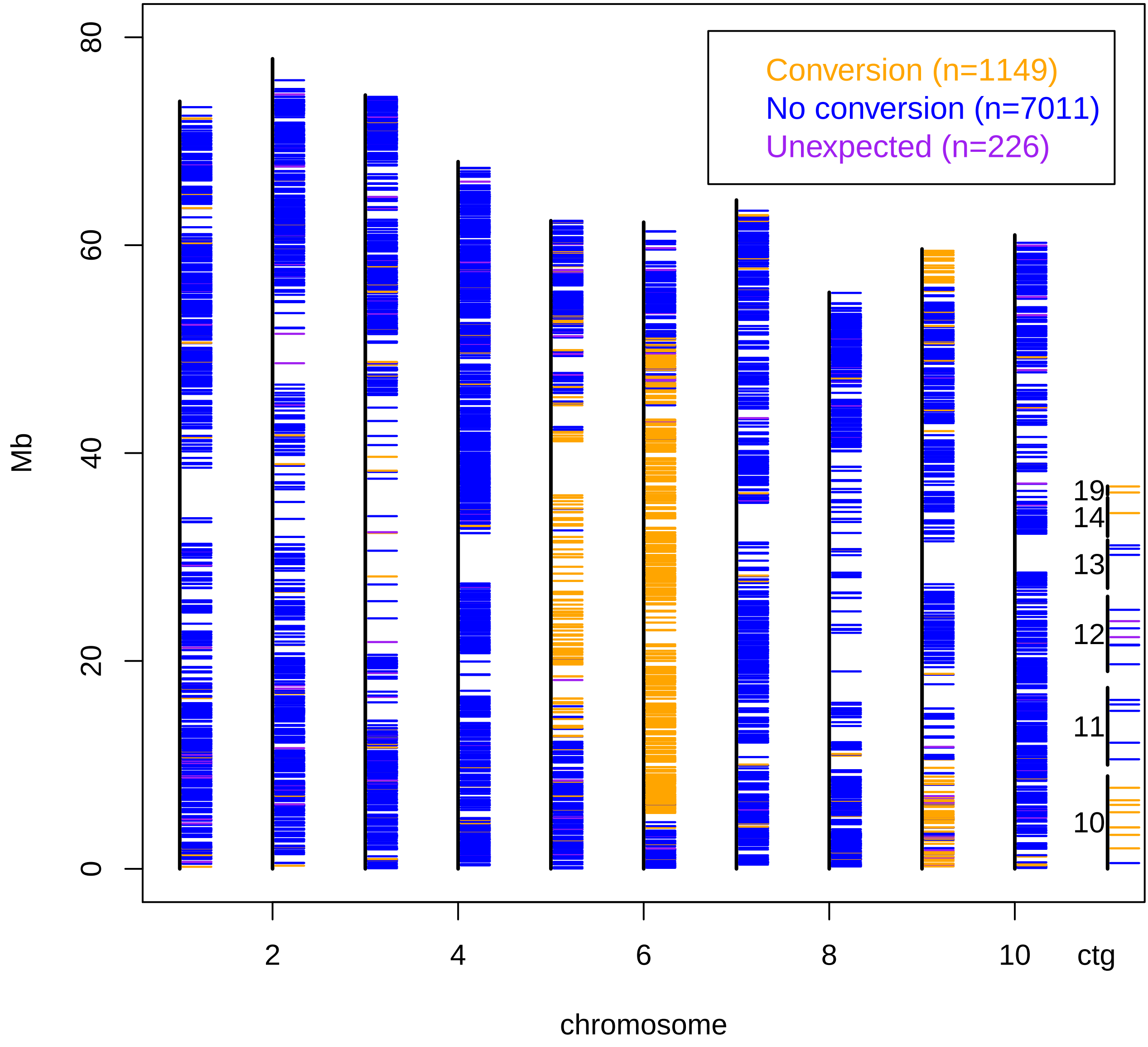

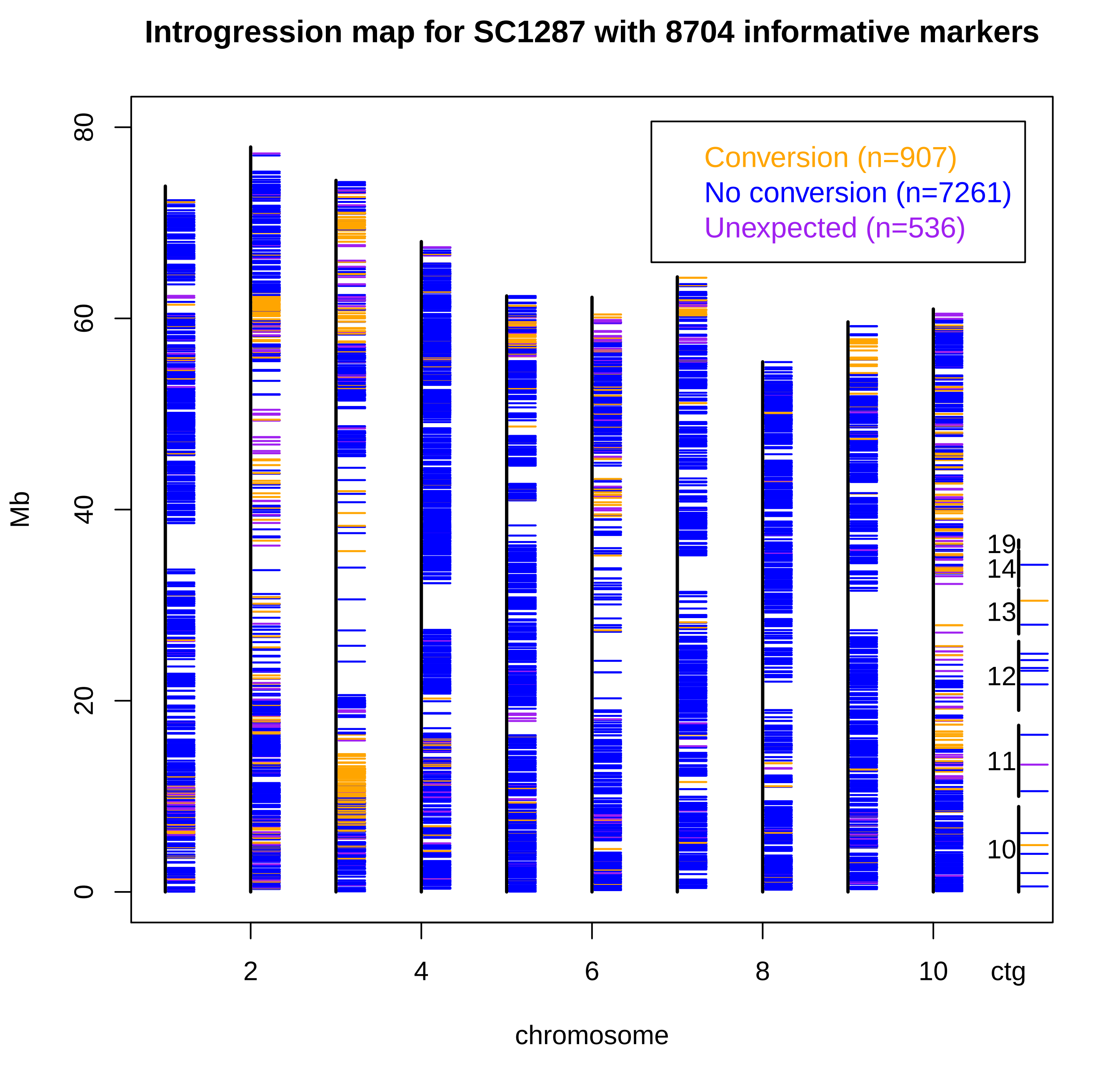

Introgression map for SC1287 with 8704 informative markers
Conversion (n=907)
No conversion (n=7261)
Unexpected (n=536)
Mb
0
20
40
60
80
chromosome
2
4
6
8
10
ctg
19
14
13
12
11
10

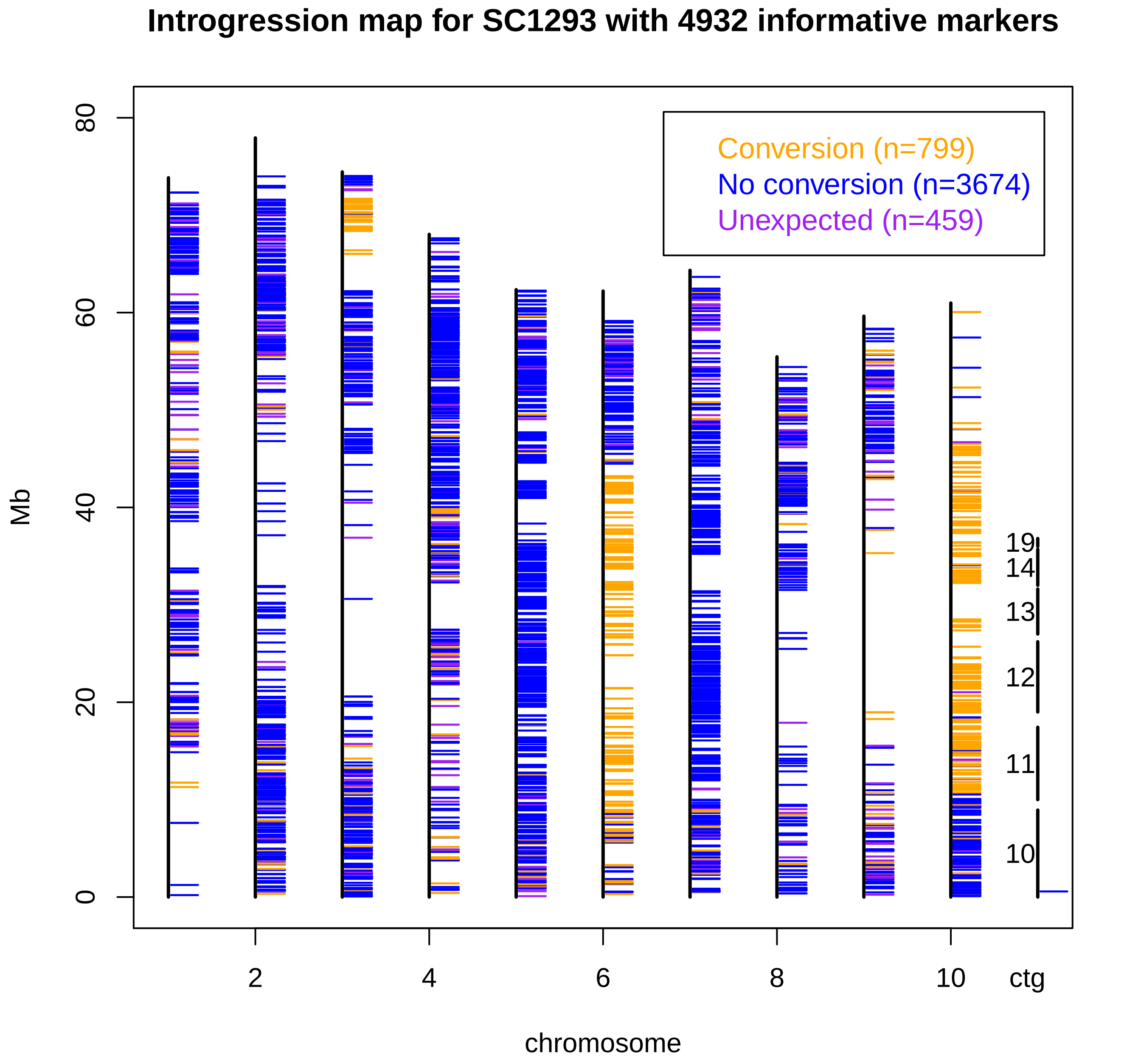

Introgression map for SC1293 with 4932 informative markers
Conversion (n=799)
No conversion (n=3674)
Unexpected (n=459)
Mb
0
20
40
60
80
2
4
6
8
10
ctg
19
14
13
12
11
10
chromosome

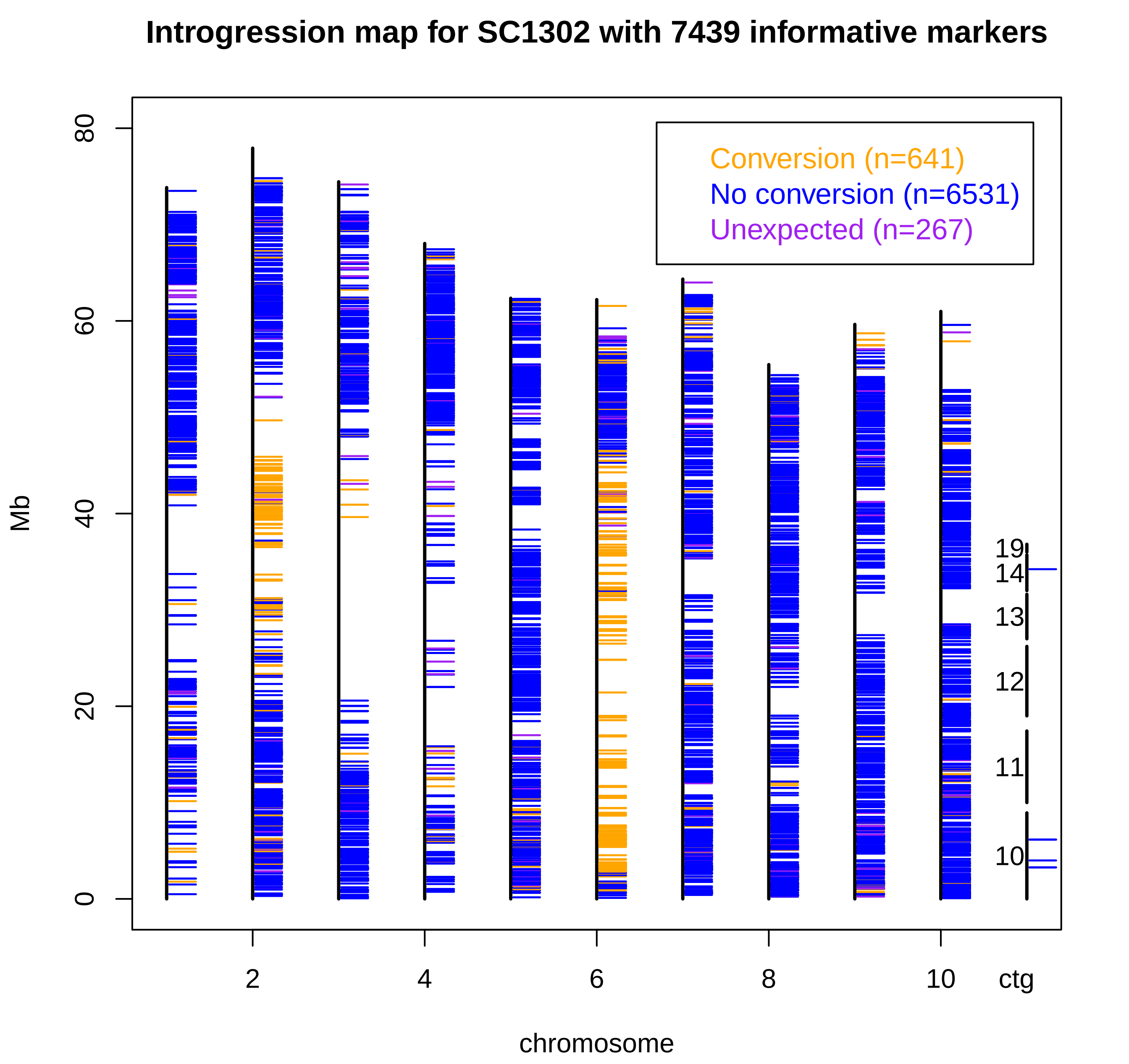
Introgression map for SC1302 with 7439 informative markers
Conversion (n=641)
No conversion (n=6531)
Unexpected (n=267)
Mb
0
20
40
60
80
chromosome
2
4
6
8
10
ctg
19
14
13
12
11
10

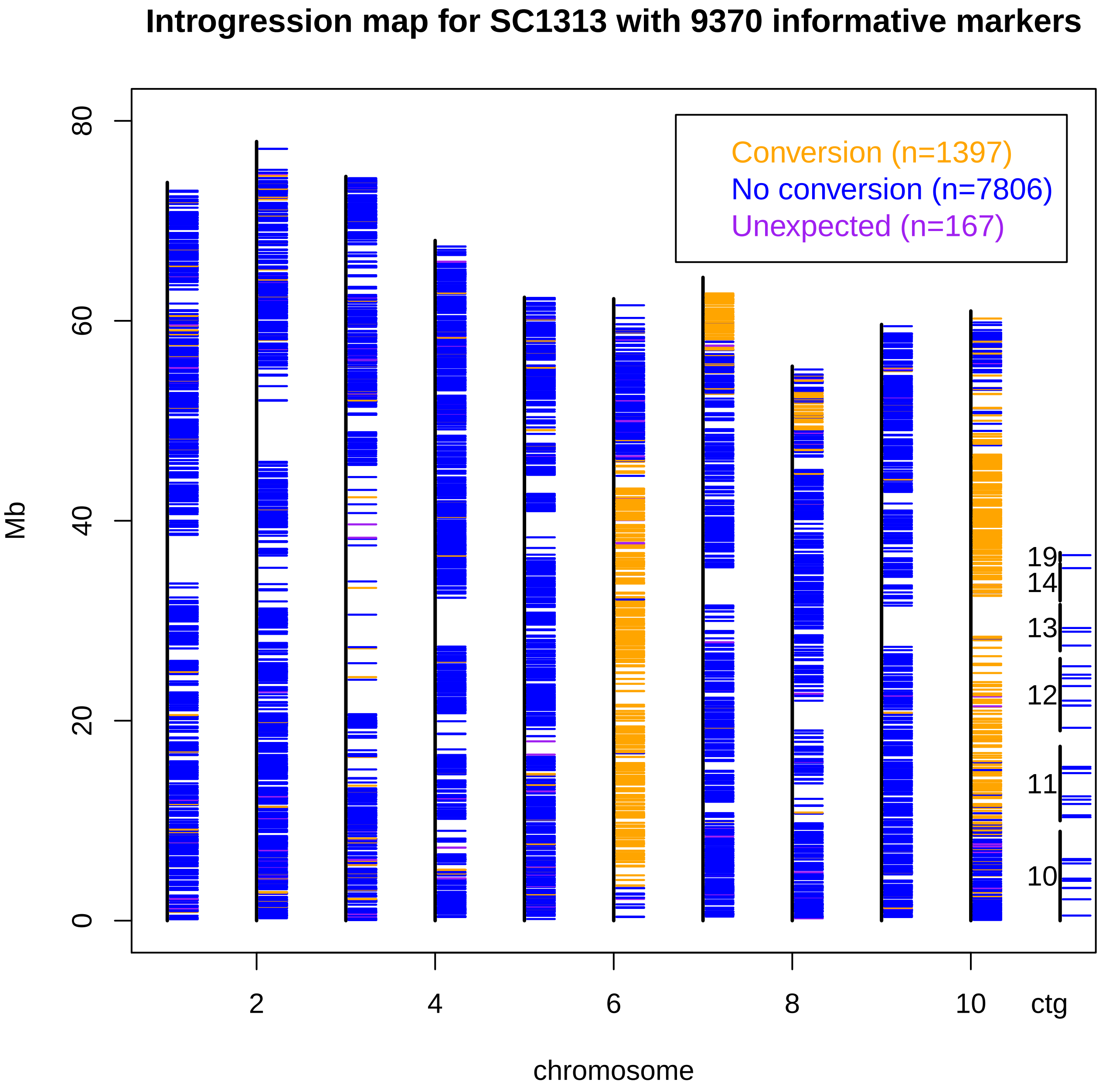

Introgression map for SC1313 with 9370 informative markers
Conversion (n=1397)
No conversion (n=7806)
Unexpected (n=167)
Mb
0
20
40
60
80
chromosome
2
4
6
8
10
ctg
19
14
13
12
11
10

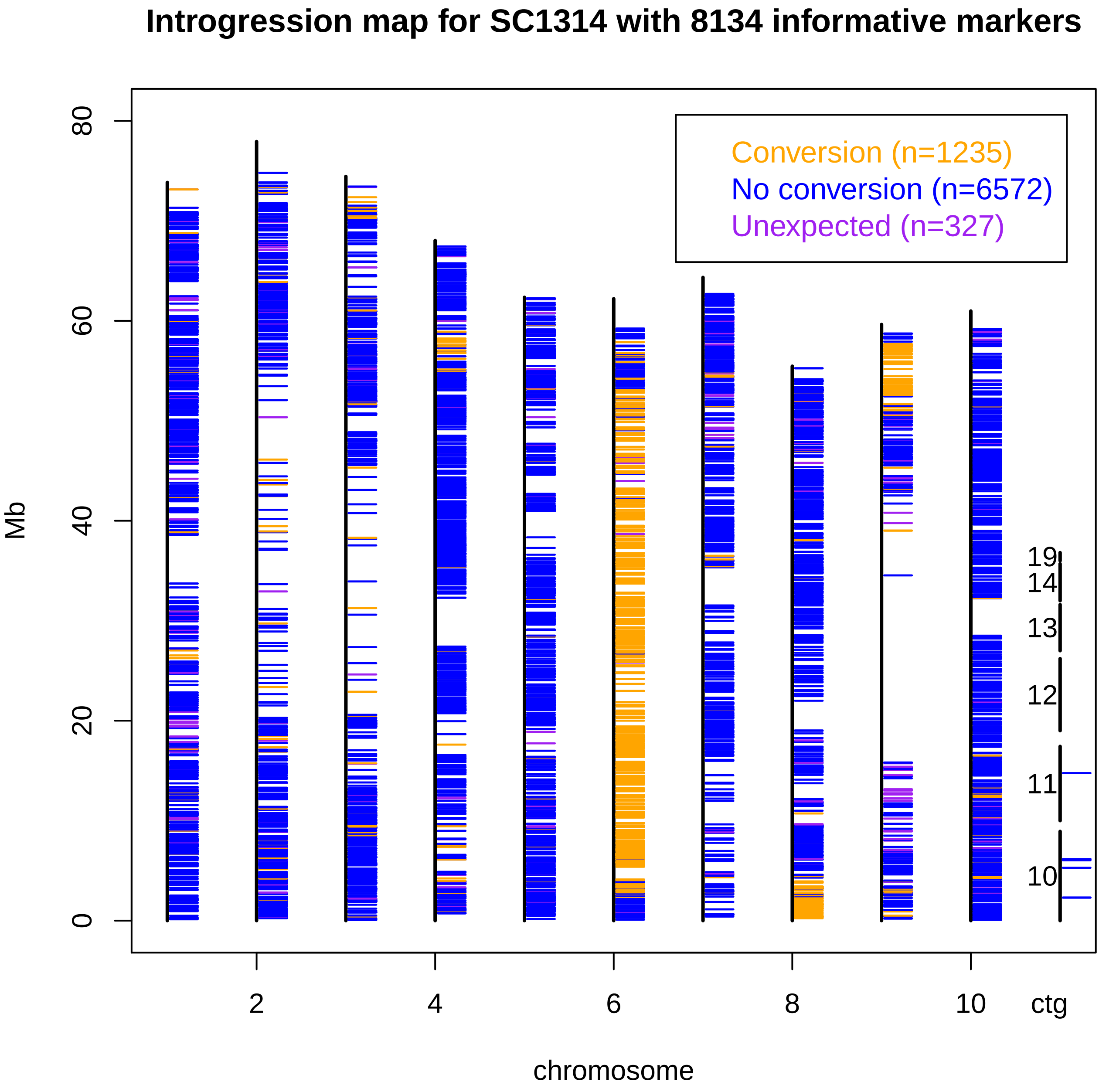

Introgression map for SC1314 with 8134 informative markers
Conversion (n=1235)
No conversion (n=6572)
Unexpected (n=327)
Mb
0
20
40
60
80
chromosome
2
4
6
8
10
ctg
19
14
13
12
11
10

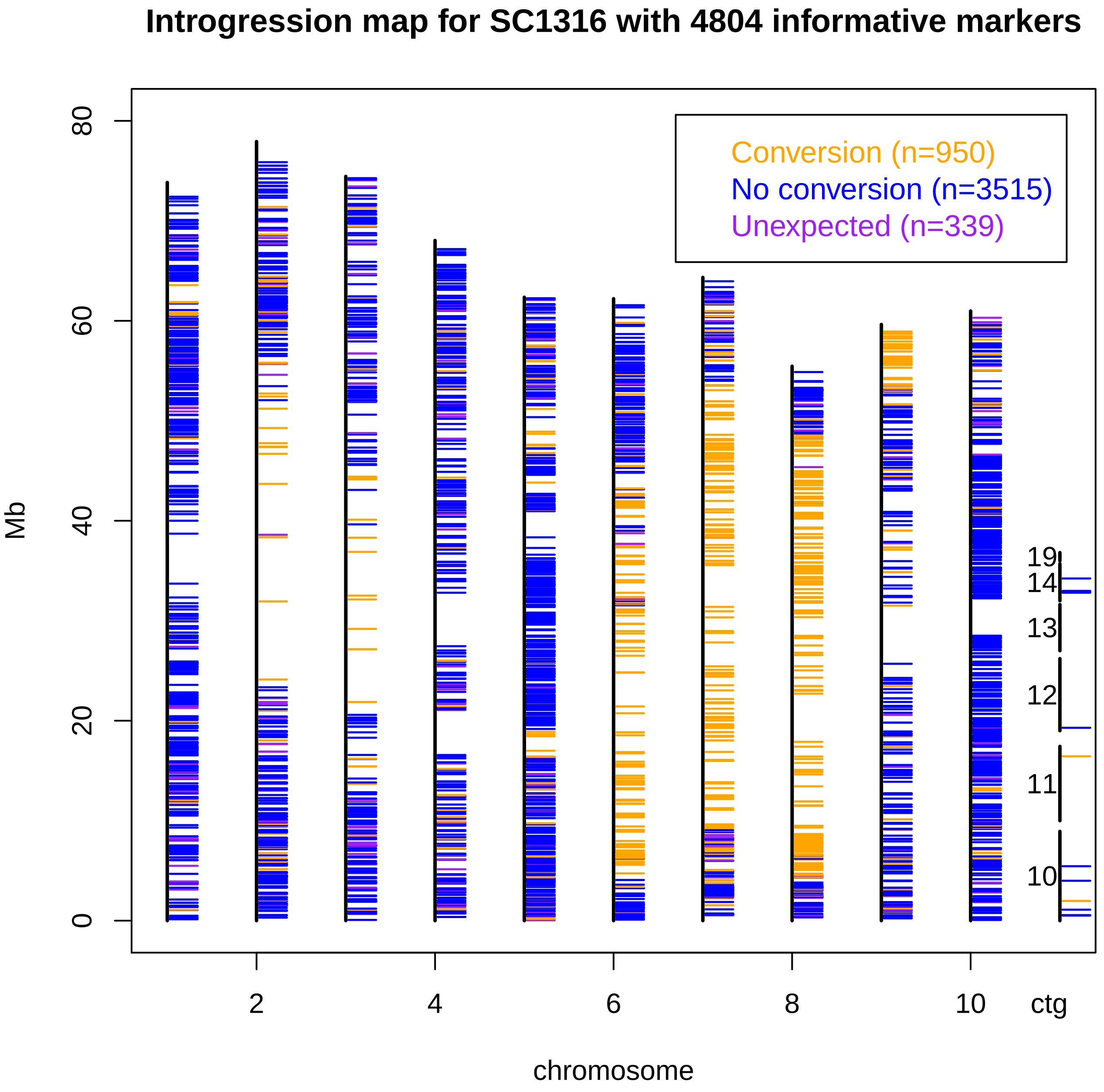
Introgression map for SC1316 with 4804 informative markers
Conversion (n=950)
No conversion (n=3515)
Unexpected (n=339)
Mb
0
20
40
60
80
chromosome
2
4
6
8
10
ctg
19
14
13
12
11
10

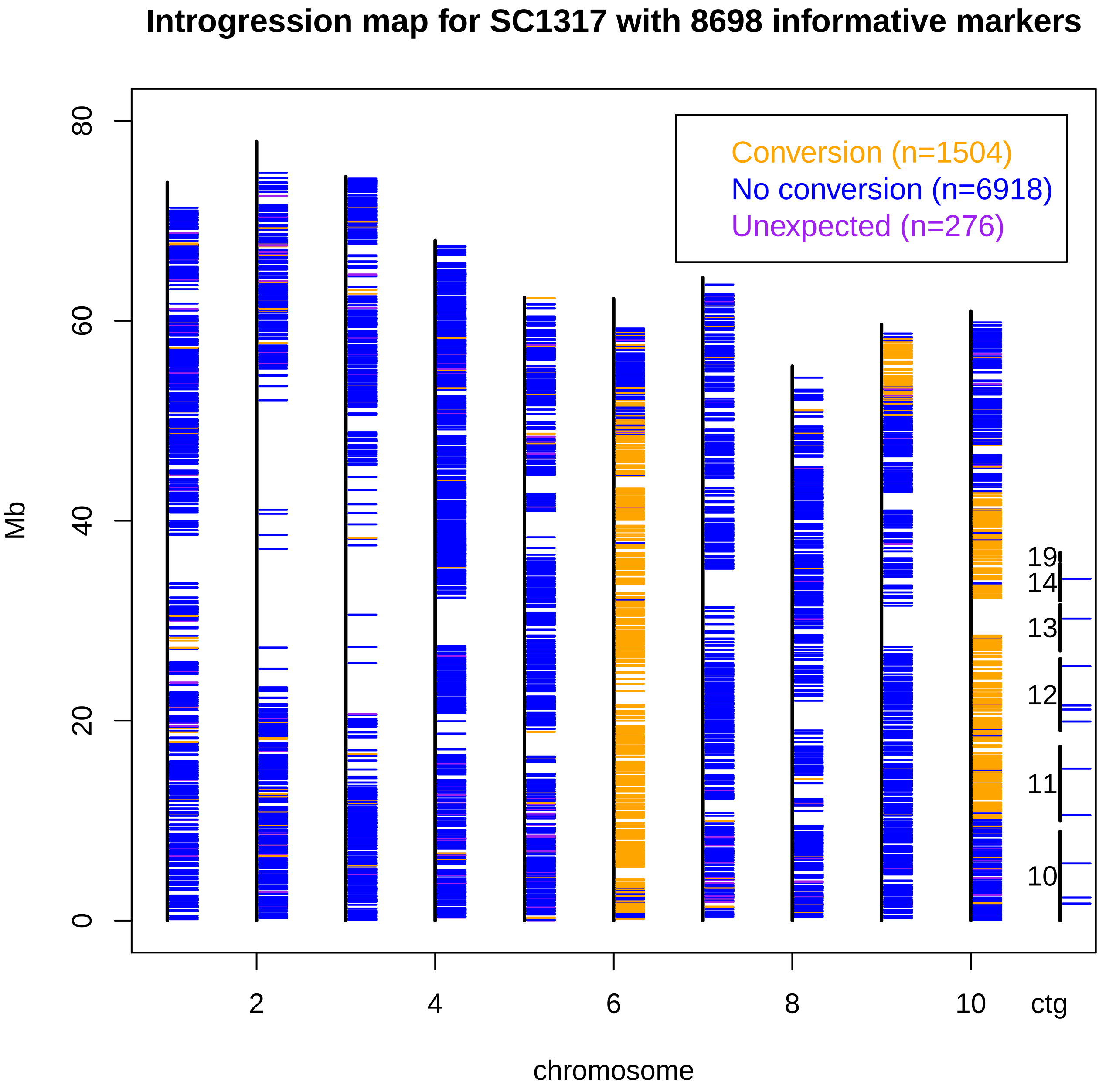

Introgression map for SC1317 with 8698 informative markers
Conversion (n=1504)
No conversion (n=6918)
Unexpected (n=276)
Mb
0
20
40
60
80
2
4
6
8
10
ctg
19
14
13
12
11
10
chromosome

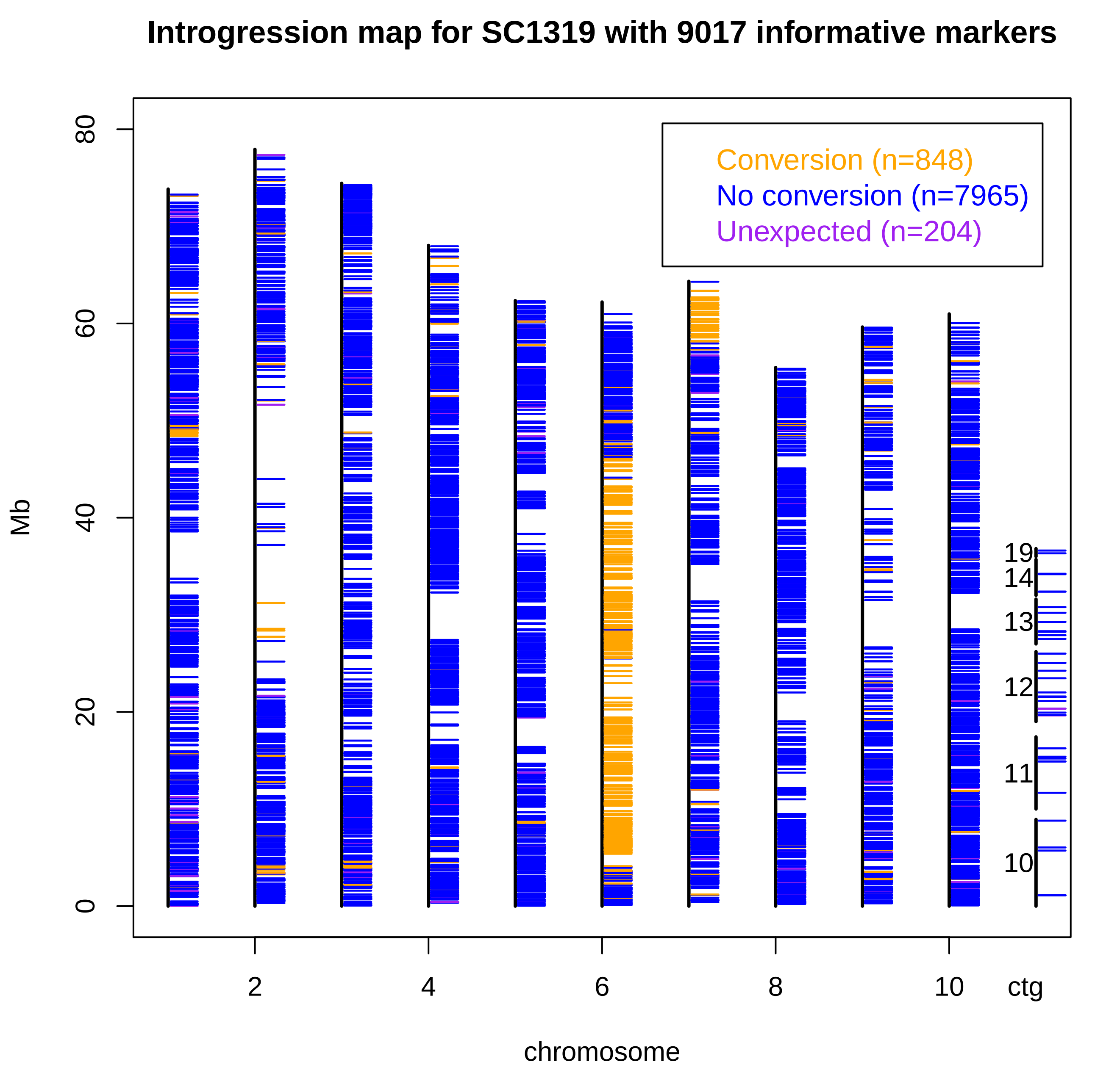

Introgression map for SC1319 with 9017 informative markers
Conversion (n=848)
No conversion (n=7965)
Unexpected (n=204)
Mb
0
20
40
60
80
chromosome
2
4
6
8
10
ctg
19
14
13
12
11
10

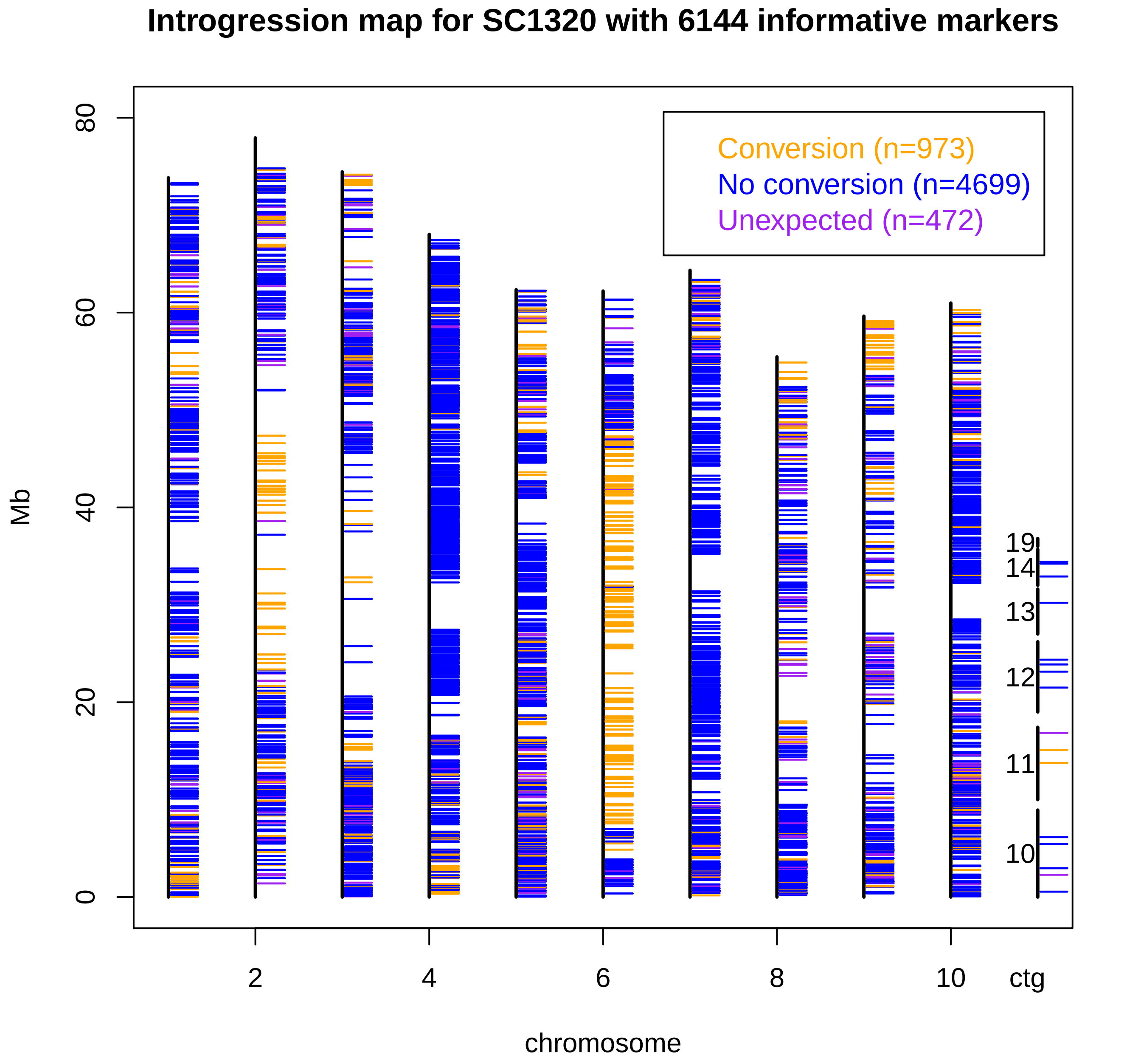
Introgression map for SC1320 with 6144 informative markers
Conversion (n=973)
No conversion (n=4699)
Unexpected (n=472)
Mb
0
20
40
60
80
chromosome
2
4
6
8
10
ctg
19
14
13
12
11
10

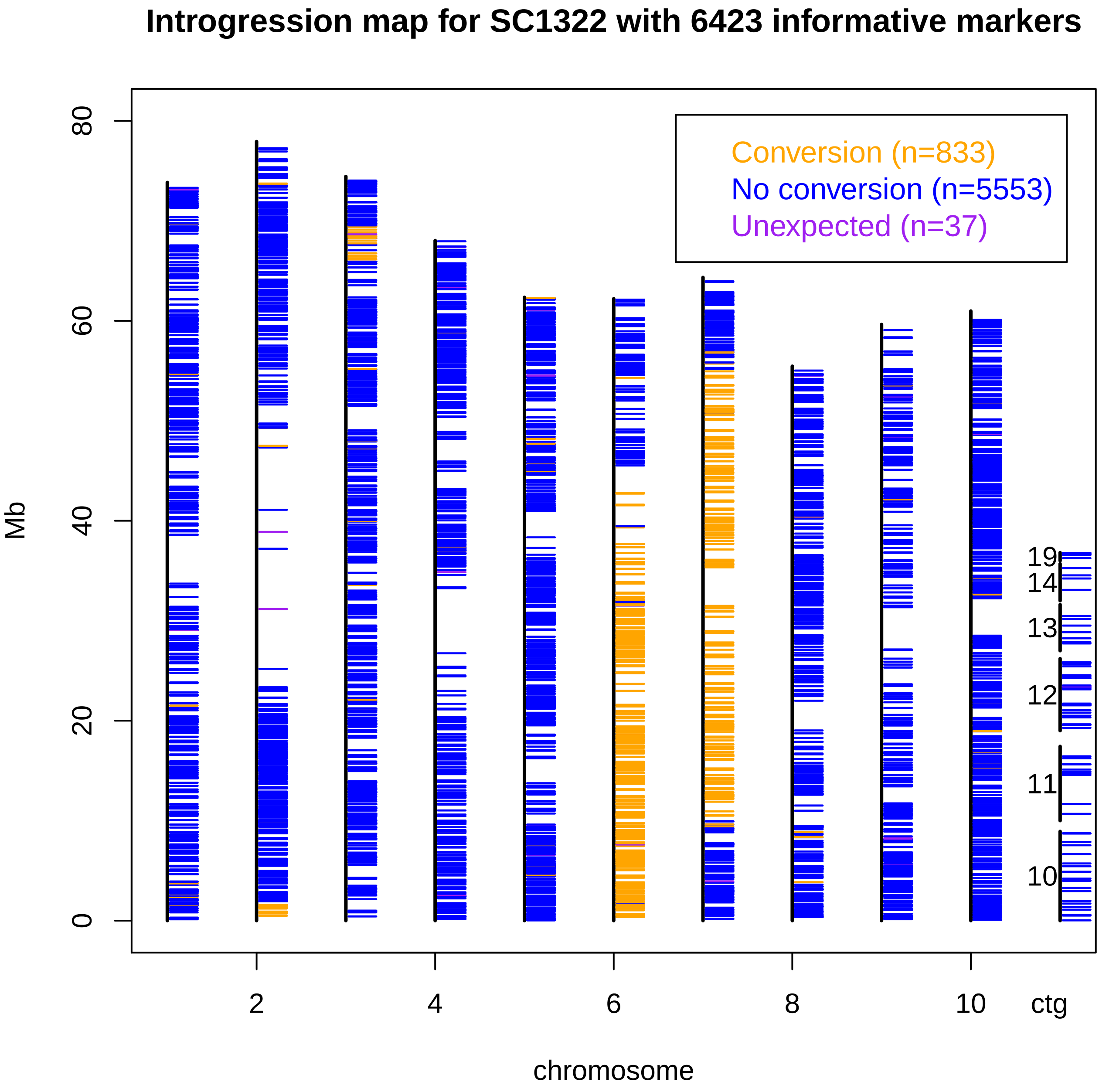
Introgression map for SC1322 with 6423 informative markers
Conversion (n=833)
No conversion (n=5553)
Unexpected (n=37)
Mb
0
20
40
60
80
chromosome
2
4
6
8
10
ctg
19
14
13
12
11
10

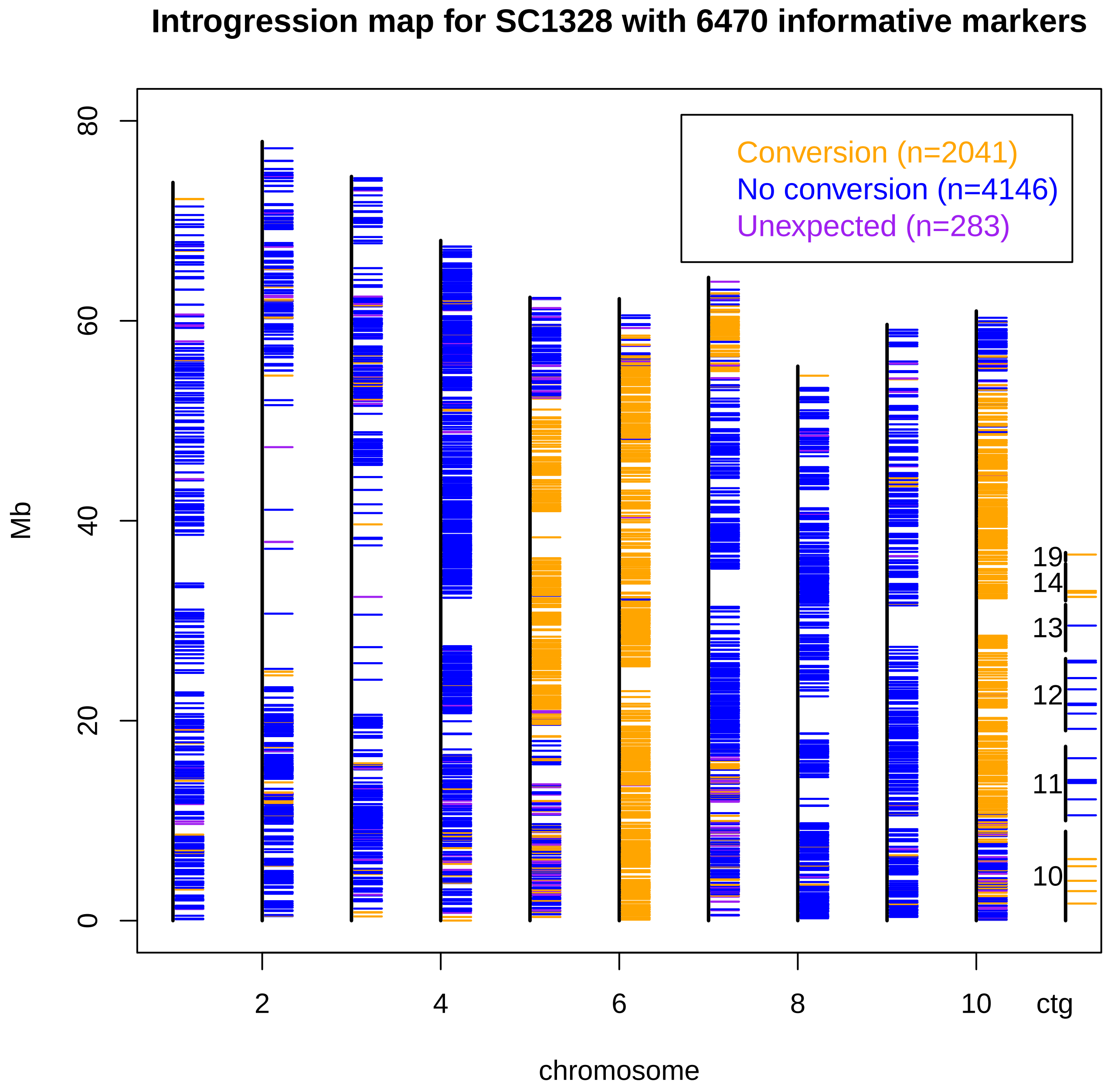
Introgression map for SC1328 with 6470 informative markers
Conversion (n=2041)
No conversion (n=4146)
Unexpected (n=283)
Mb
0
20
40
60
80
2
4
6
8
10
ctg
chromosome
19
14
13
12
11
10

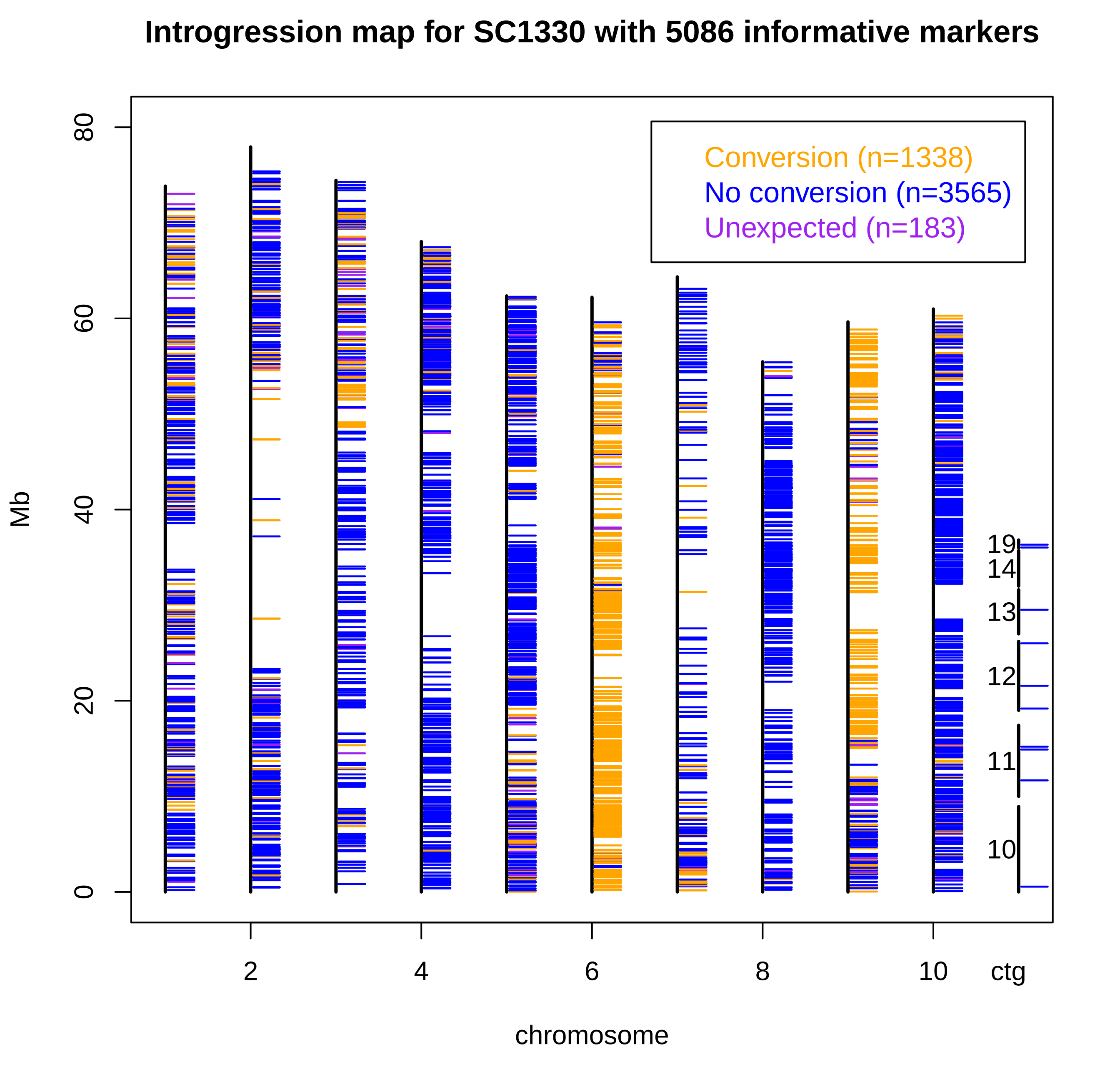

Introgression map for SC1330 with 5086 informative markers
Conversion (n=1338)
No conversion (n=3565)
Unexpected (n=183)
Mb
0
20
40
60
80
2
4
6
8
10
ctg
19
14
13
12
11
10
chromosome

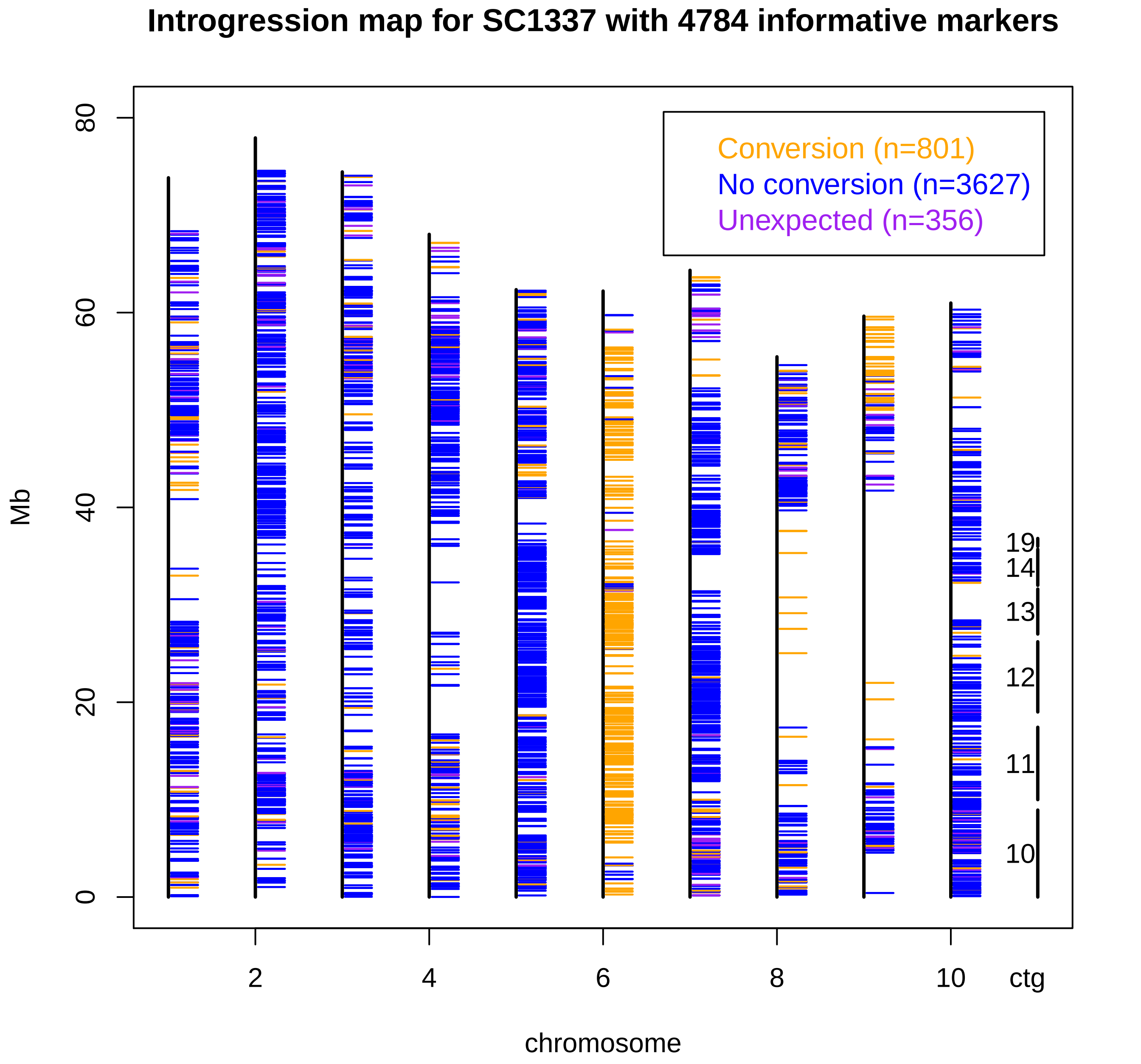
Introgression map for SC1337 with 4784 informative markers
Conversion (n=801)
No conversion (n=3627)
Unexpected (n=356)
Mb
0
20
40
60
80
chromosome
2
4
6
8
10
ctg
19
14
13
12
11
10

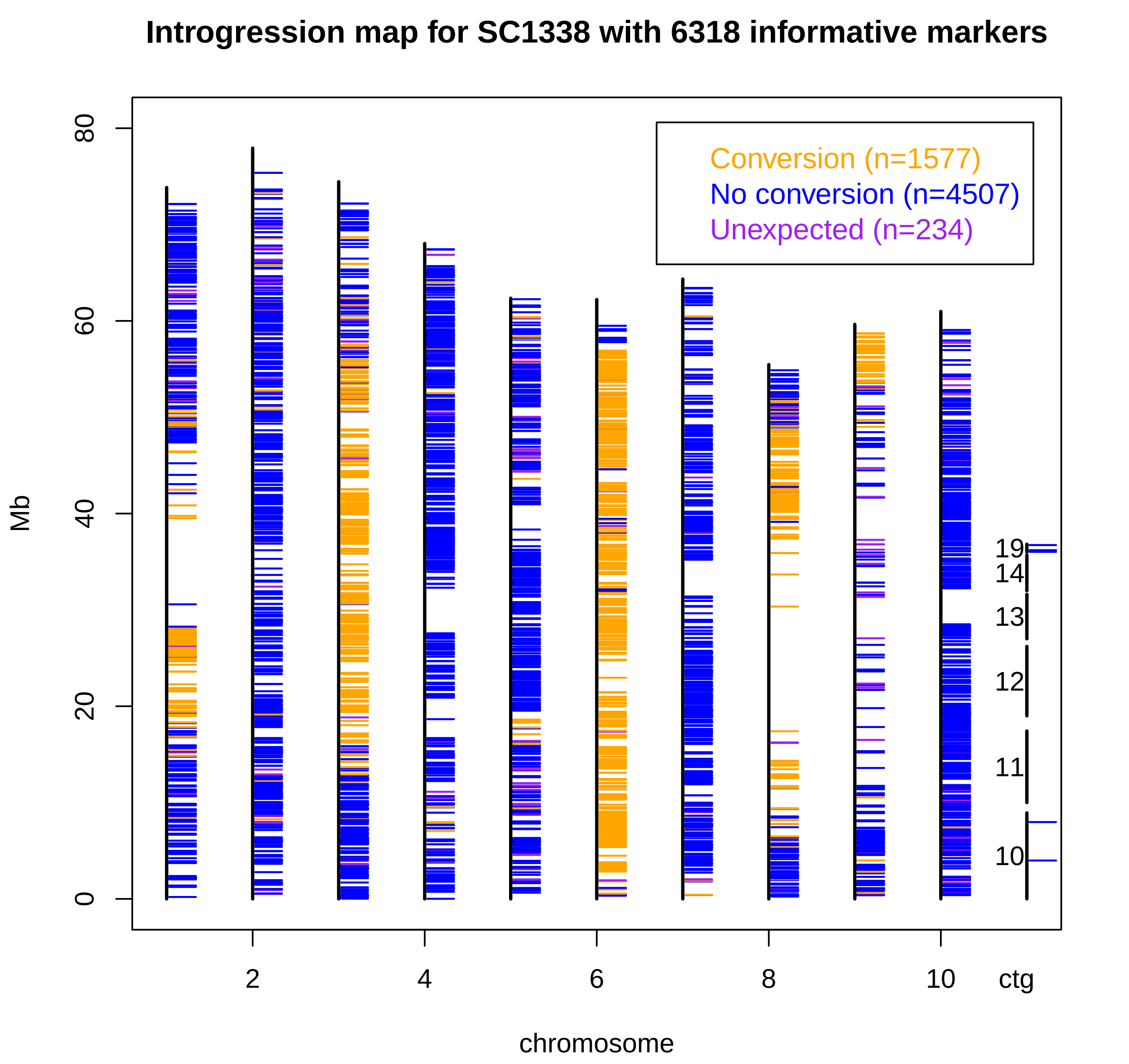
Introgression map for SC1338 with 6318 informative markers
Conversion (n=1577)
No conversion (n=4507)
Unexpected (n=234)
Mb
0
20
40
60
80
chromosome
2
4
6
8
10
ctg
19
14
13
12
11
10

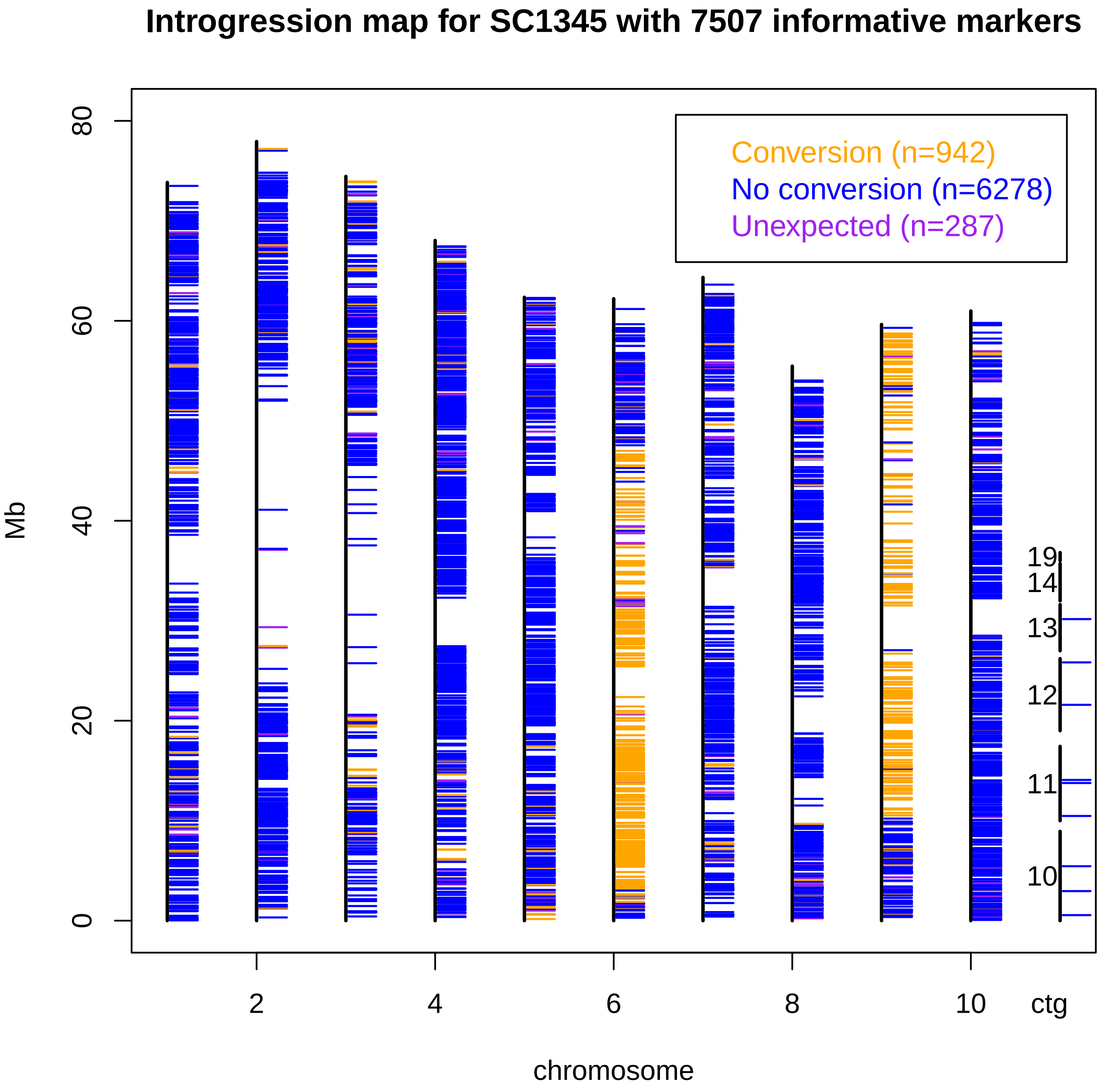

Introgression map for SC1345 with 7507 informative markers
Conversion (n=942)
No conversion (n=6278)
Unexpected (n=287)
Mb
0
20
40
60
80
2
4
6
8
10
ctg
chromosome
19
14
13
12
11
10

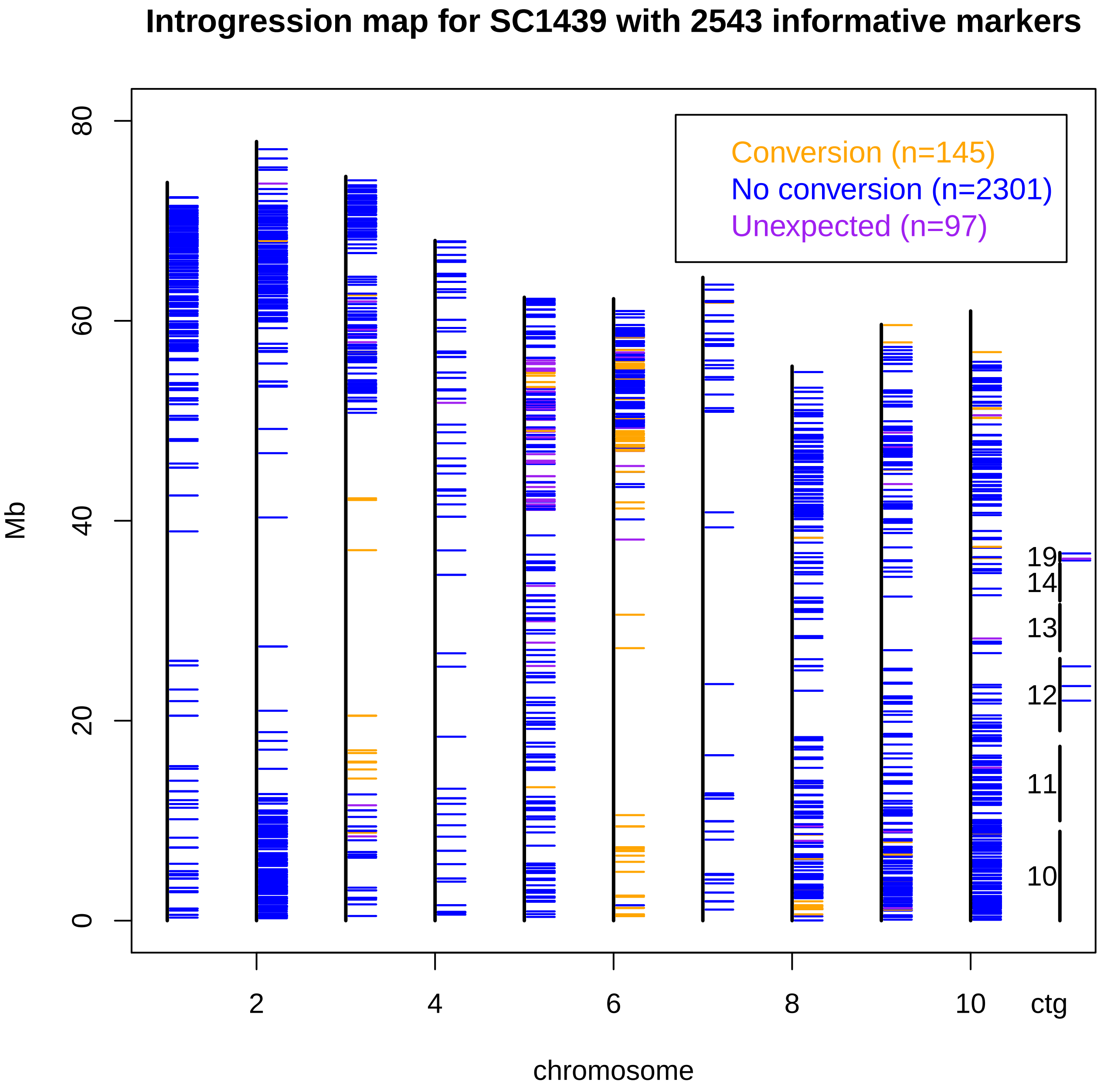
Introgression map for SC1439 with 2543 informative markers
Conversion (n=145)
No conversion (n=2301)
Unexpected (n=97)
Mb
0
20
40
60
80
chromosome
2
4
6
8
10
ctg
19
14
13
12
11
10

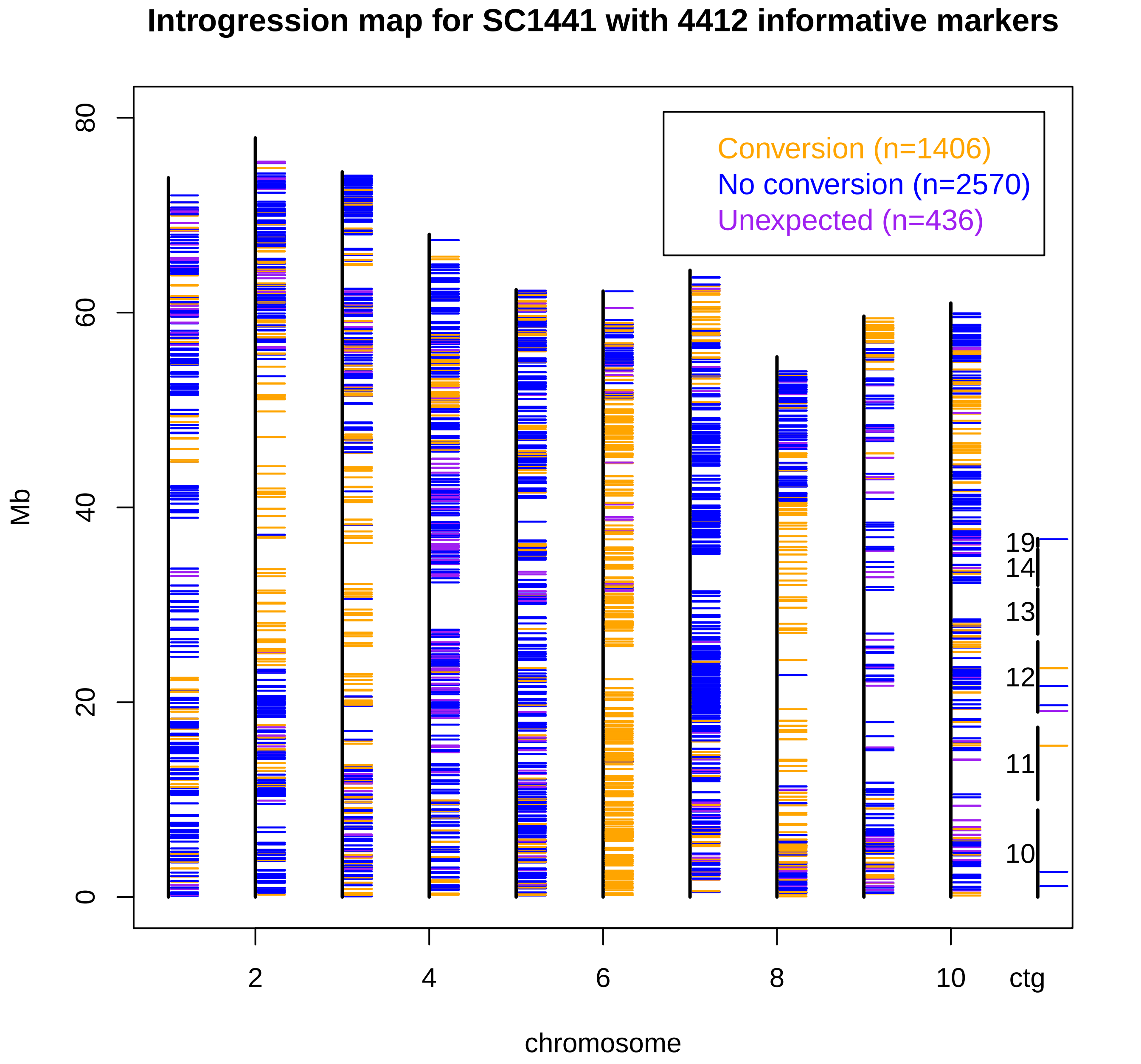

Introgression map for SC1441 with 4412 informative markers
Conversion (n=1406)
No conversion (n=2570)
Unexpected (n=436)
Mb
0
20
40
60
80
chromosome
2
4
6
8
10
ctg
19
14
13
12
11
10

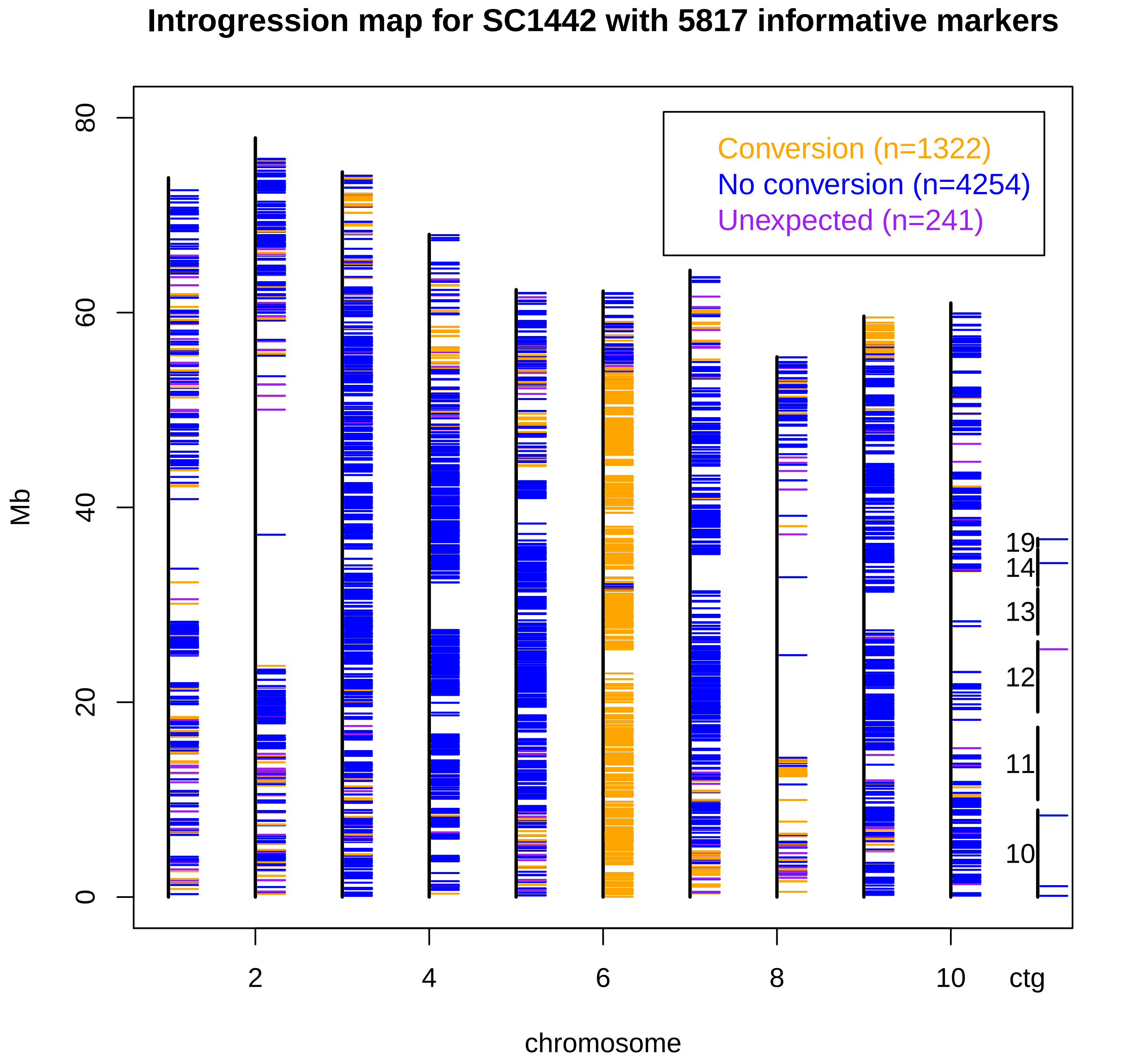

Introgression map for SC1442 with 5817 informative markers
Conversion (n=1322)
No conversion (n=4254)
Unexpected (n=241)
Mb
0
20
40
60
80
2
4
6
8
10
ctg
19
14
13
12
11
10
chromosome

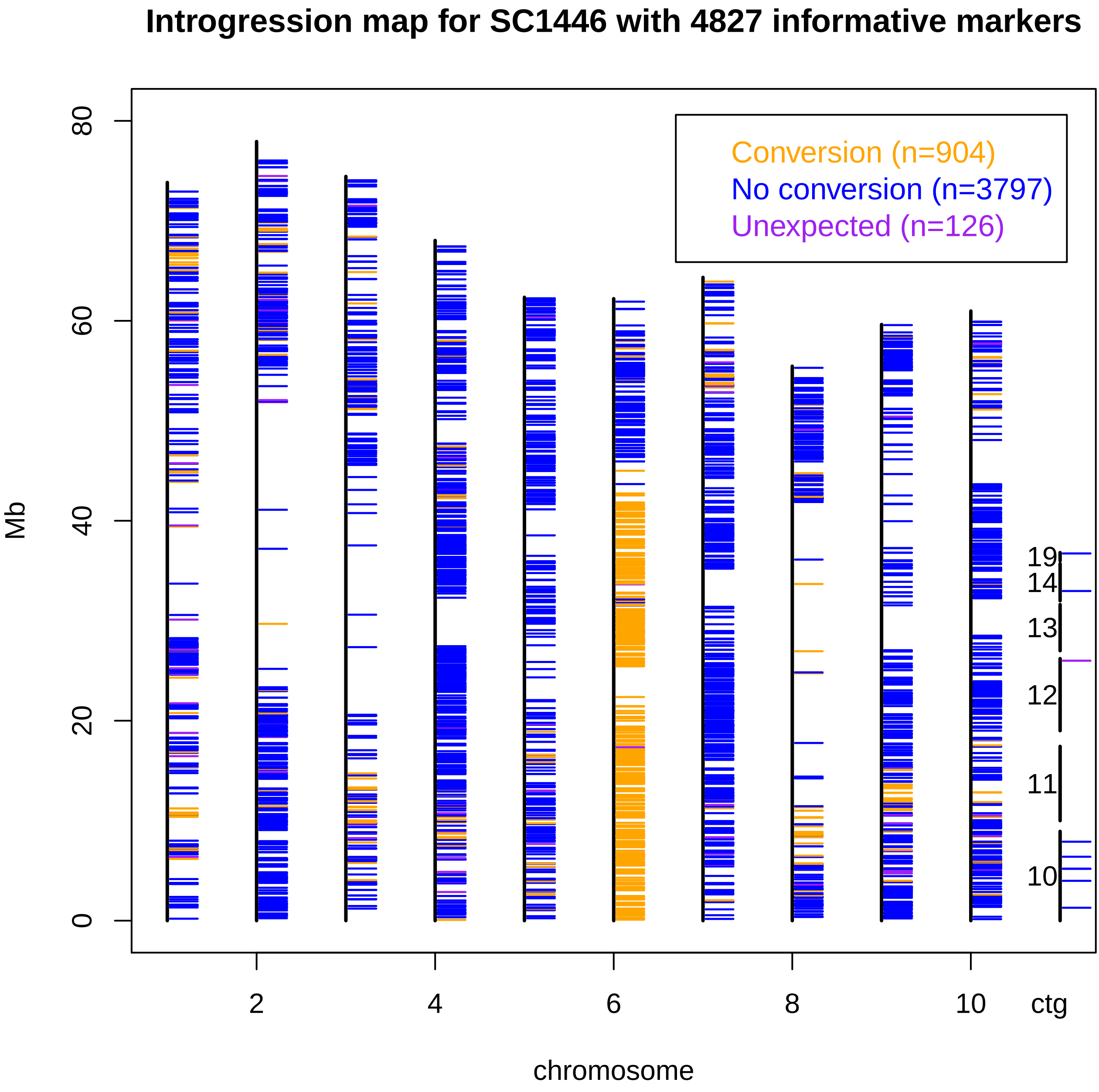

Introgression map for SC1446 with 4827 informative markers
Conversion (n=904)
No conversion (n=3797)
Unexpected (n=126)
Mb
0
20
40
60
80
chromosome
2
4
6
8
10
ctg
19
14
13
12
11
10

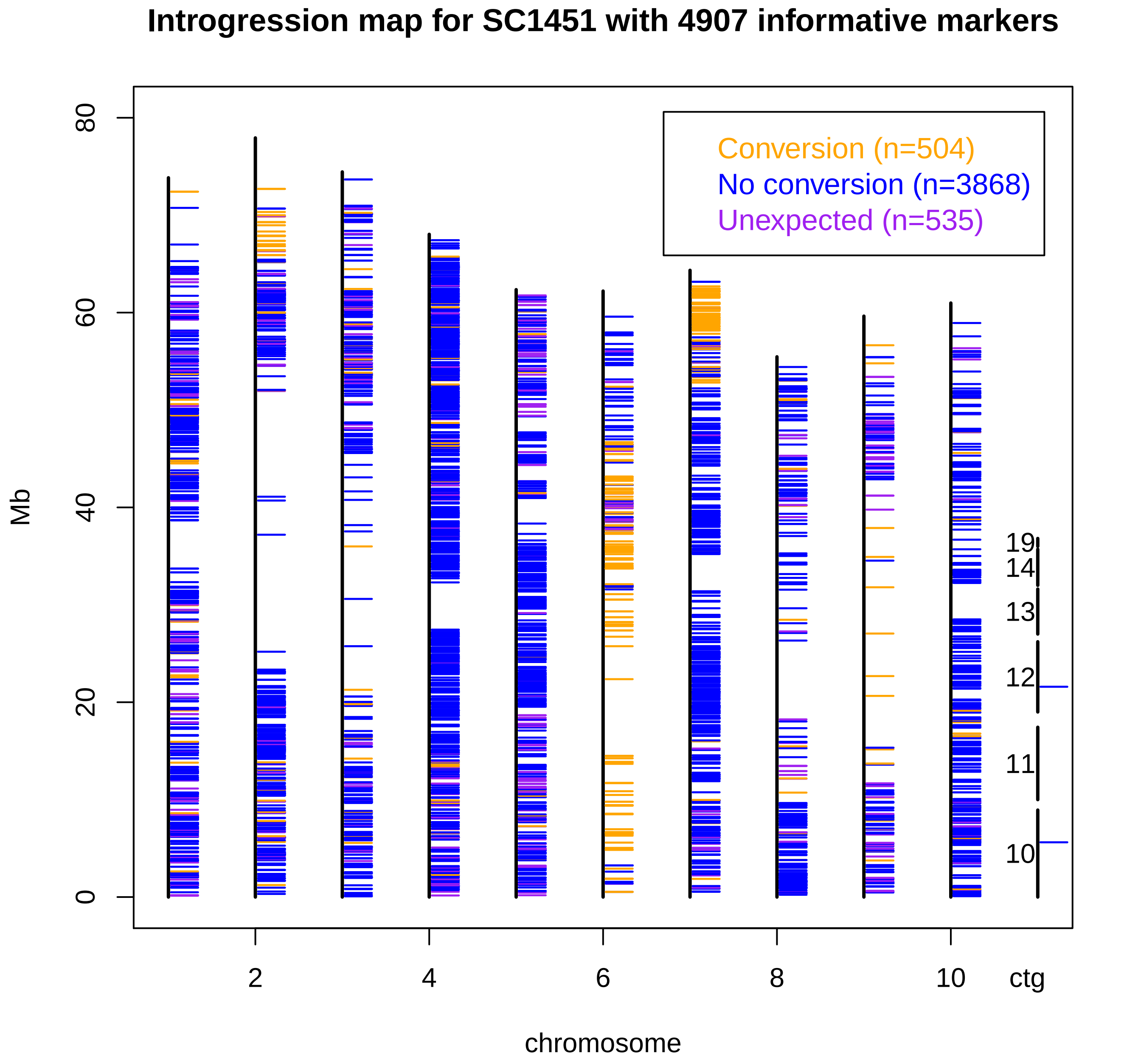

Introgression map for SC1451 with 4907 informative markers
Conversion (n=504)
No conversion (n=3868)
Unexpected (n=535)
Mb
0
20
40
60
80
2
4
6
8
10
ctg
19
14
13
12
11
10
chromosome

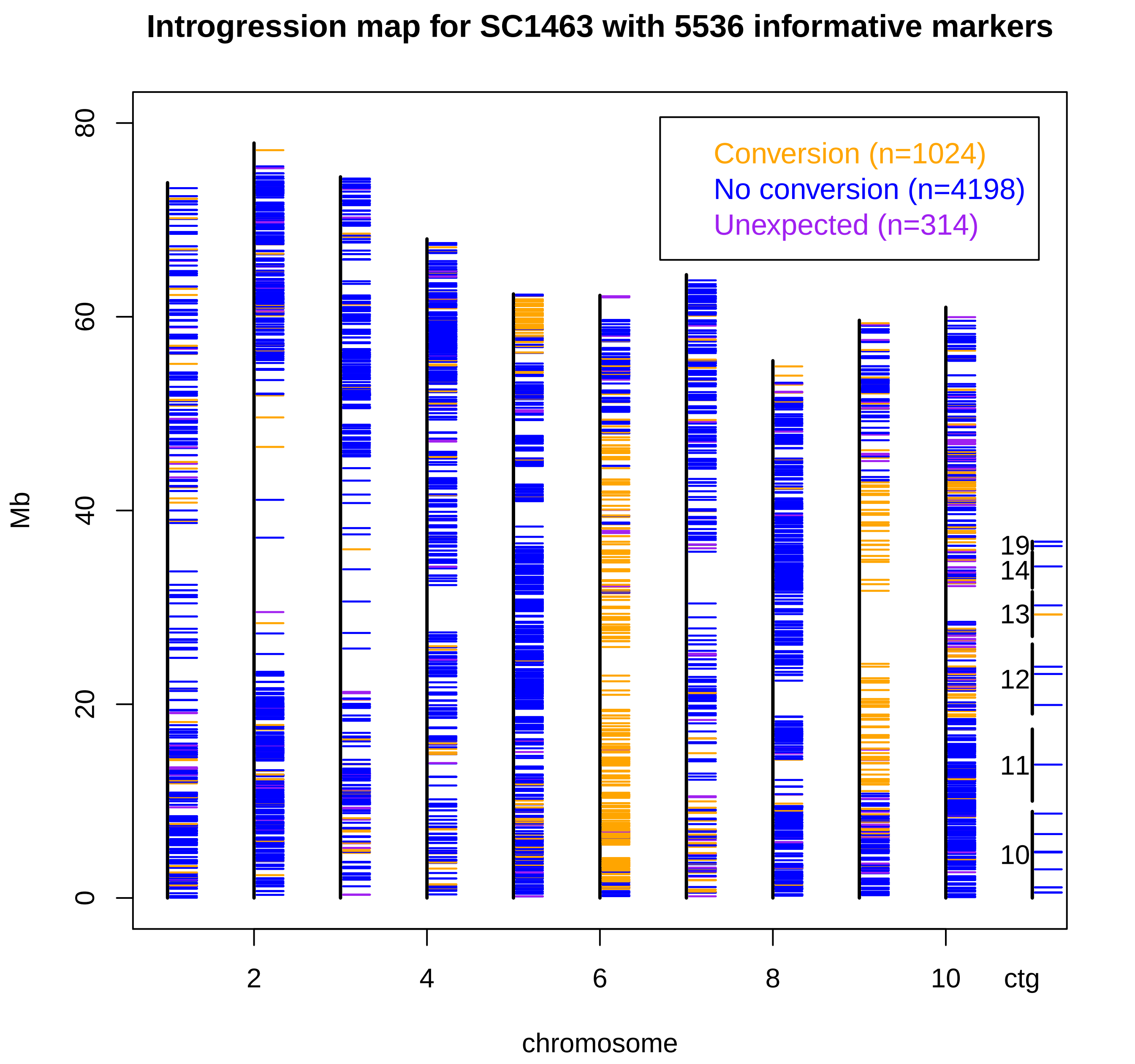
Introgression map for SC1463 with 5536 informative markers
Conversion (n=1024)
No conversion (n=4198)
Unexpected (n=314)
Mb
0
20
40
60
80
chromosome
2
4
6
8
10
ctg
19
14
13
12
11
10

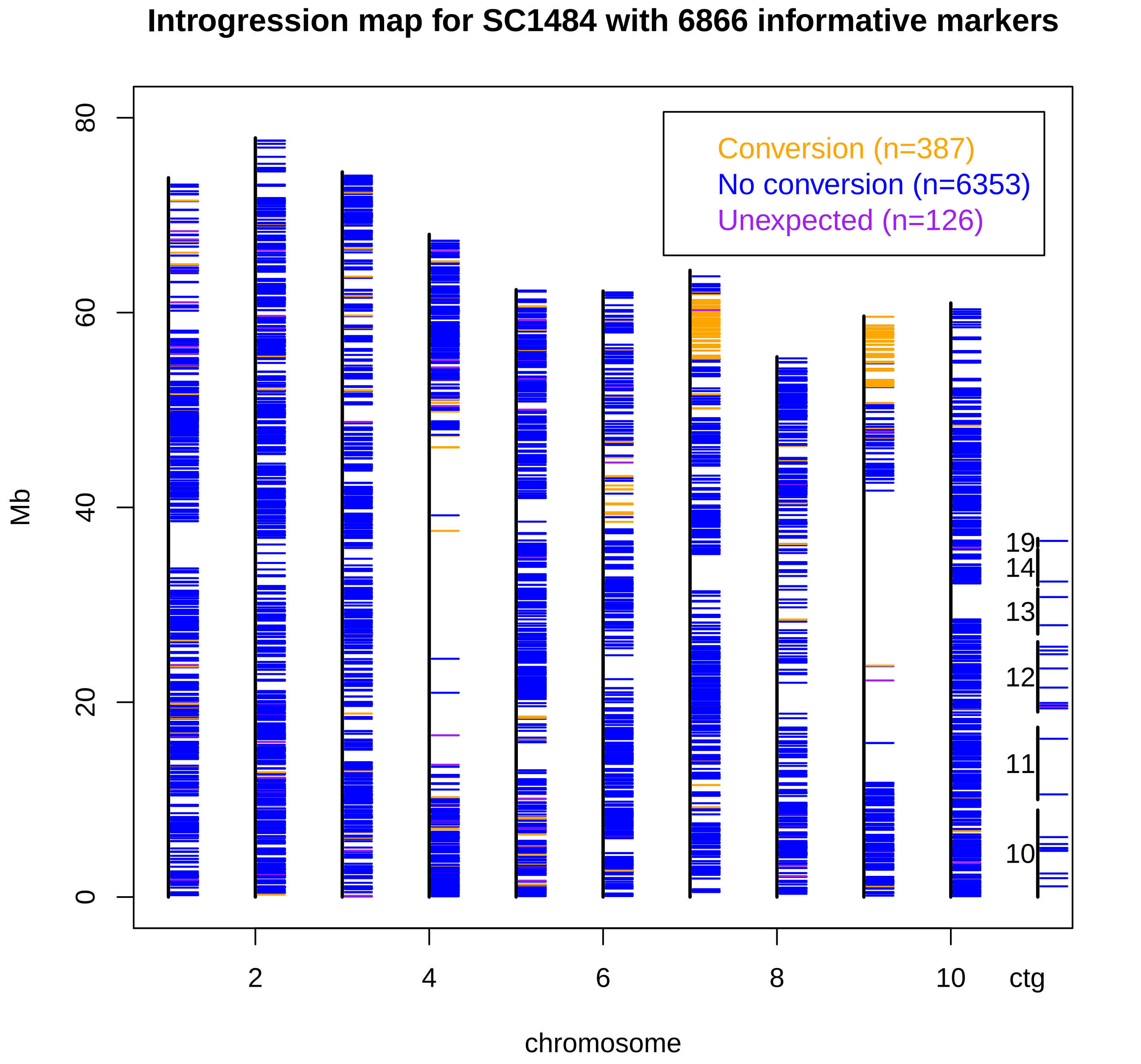
Introgression map for SC1484 with 6866 informative markers
Conversion (n=387)
No conversion (n=6353)
Unexpected (n=126)
Mb
0
20
40
60
80
chromosome
2
4
6
8
10
ctg
19
14
13
12
11
10
