# Supplementary material for: Retrospective genomic analysis of sorghum adaptation to temperate-zone grain production
Source: Genome Biol. 2013 Jun 26;14(6):R68. doi: 10.1186/gb-2013-14-6-r68 (PMC3706989; doi:10.1186/gb-2013-14-6-r68)
Supplement: Additional File 9 — Figure S6. Subpopulation-specific introgression frequencies. [file gb-2013-14-6-r68-S9.PDF]

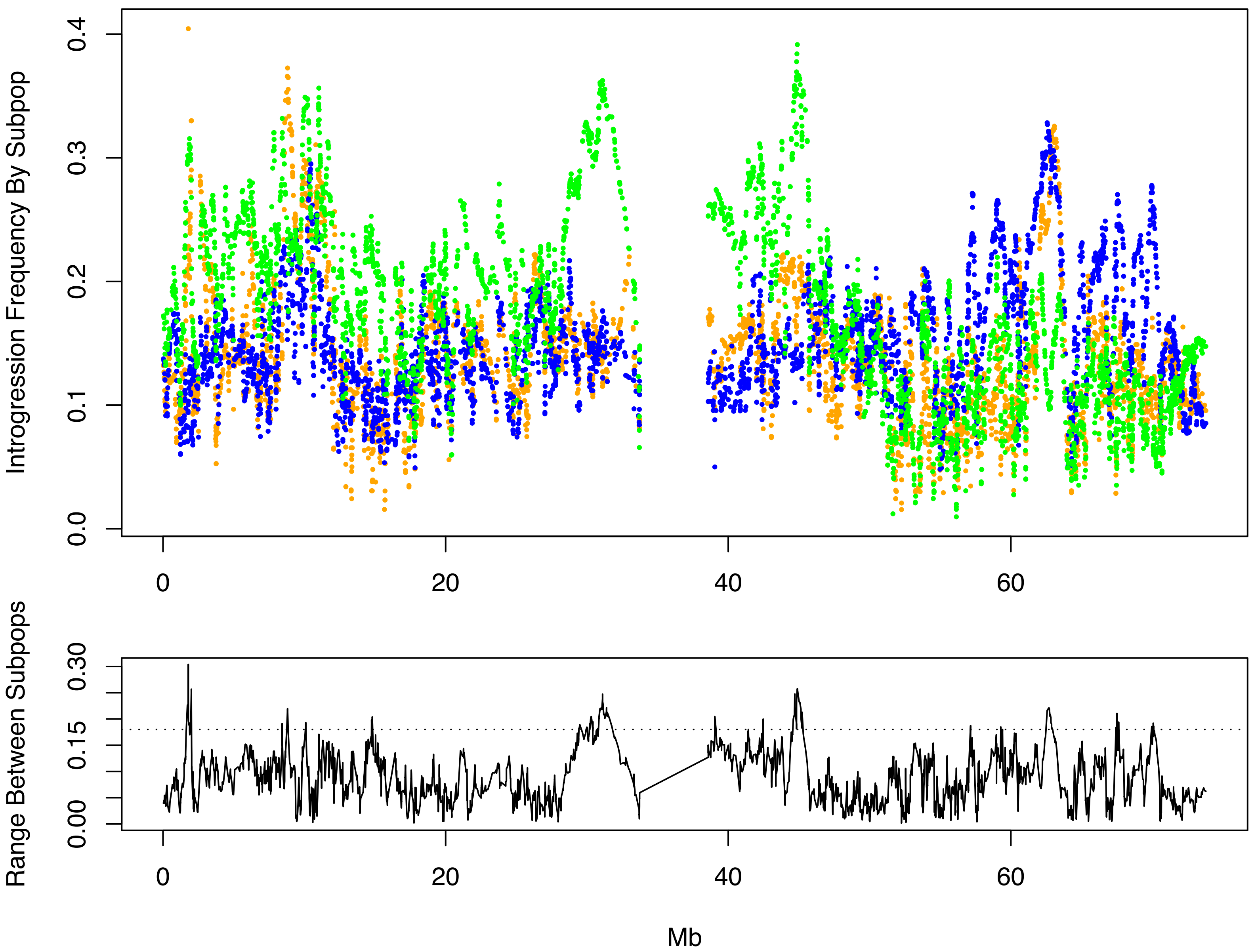

Introgression Frequency By Subpop
0.0
0.1
0.2
0.3
0.4
0
20
40
60
Range Between Subpops
0.00
0.15
0.30
0
20
40
60
Mb

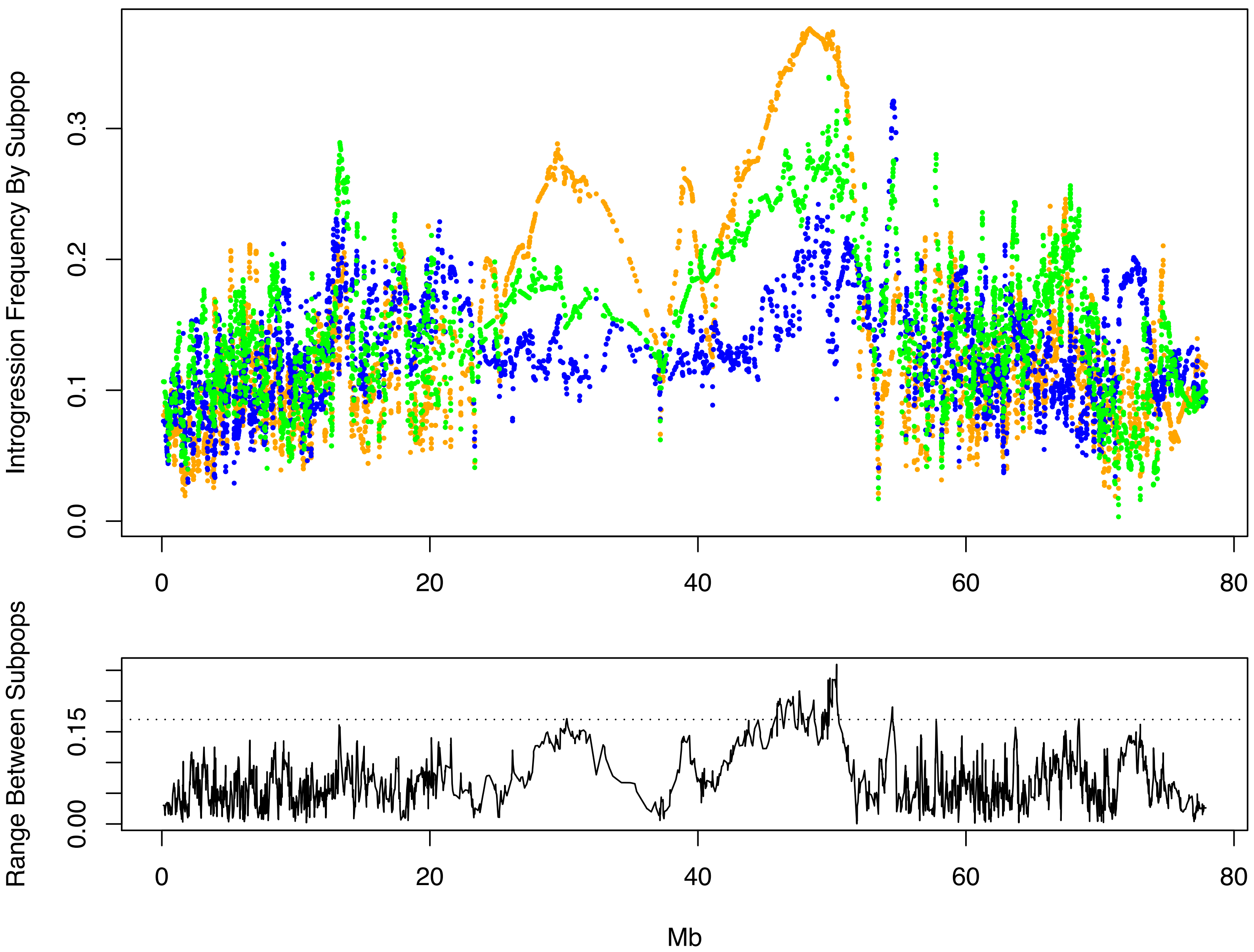

Introgression Frequency By Subpop
0.0
0.1
0.2
0.3
0
20
40
60
80
Range Between Subpops
0.00
0.15
0
20
40
60
80
Mb

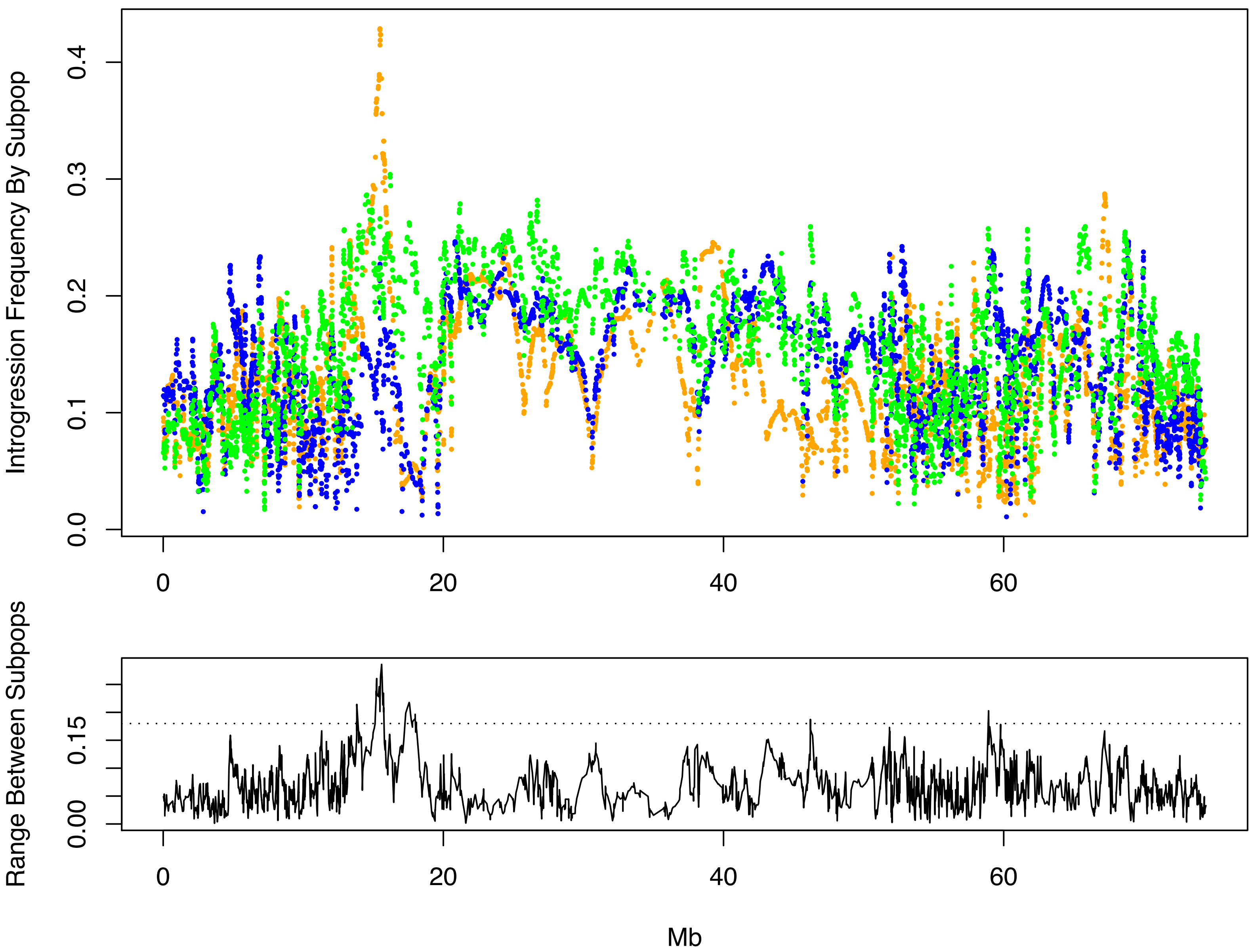

Introgression Frequency By Subpop
0.0
0.1
0.2
0.3
0.4
0
20
40
60
Range Between Subpops
0.00
0.15
0
20
40
60
Mb

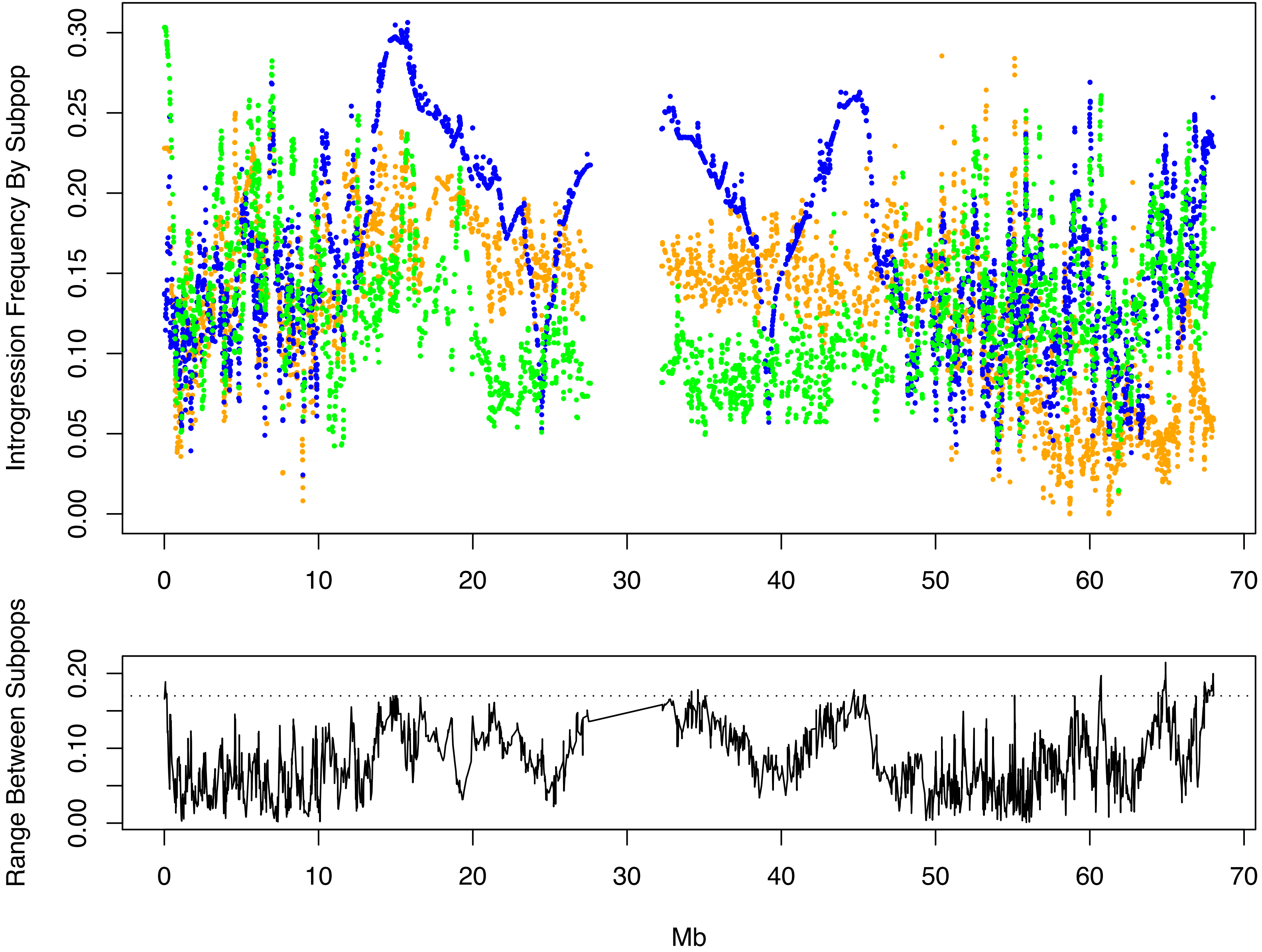
Introgression Frequency By Subpop
0.00
0.05
0.10
0.15
0.20
0.25
0.30
0
10
20
30
40
50
60
70
Range Between Subpops
0.00
0.10
0.20
Mb

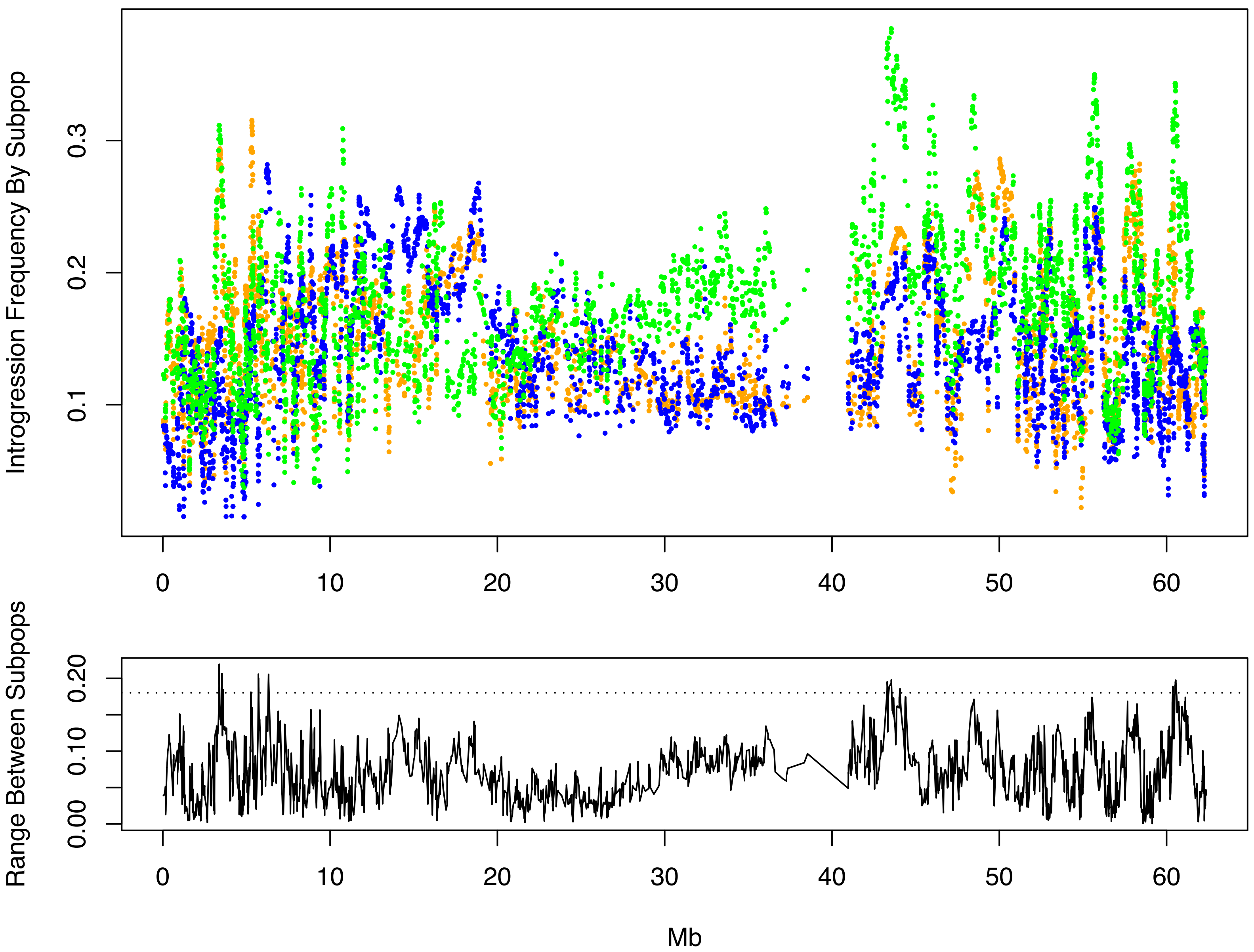
Introgression Frequency By Subpop
0.1
0.2
0.3
0
10
20
30
40
50
60
Range Between Subpops
0.00
0.10
0.20
Mb

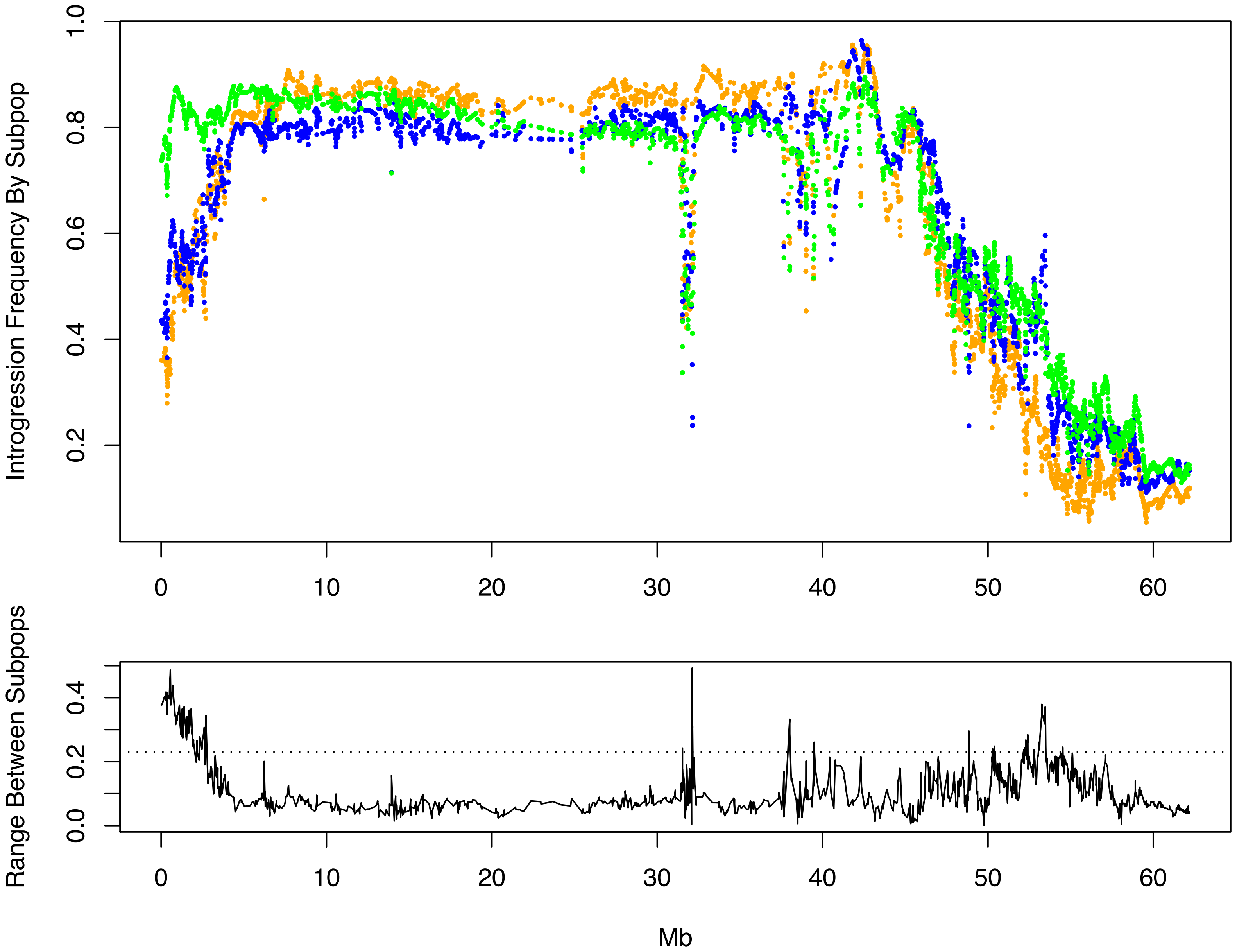

Introgression Frequency By Subpop
1.0
0.8
0.6
0.4
0.2
0
10
20
30
40
50
60
Range Between Subpops
0.4
0.2
0.0
0
10
20
30
40
50
60
Mb

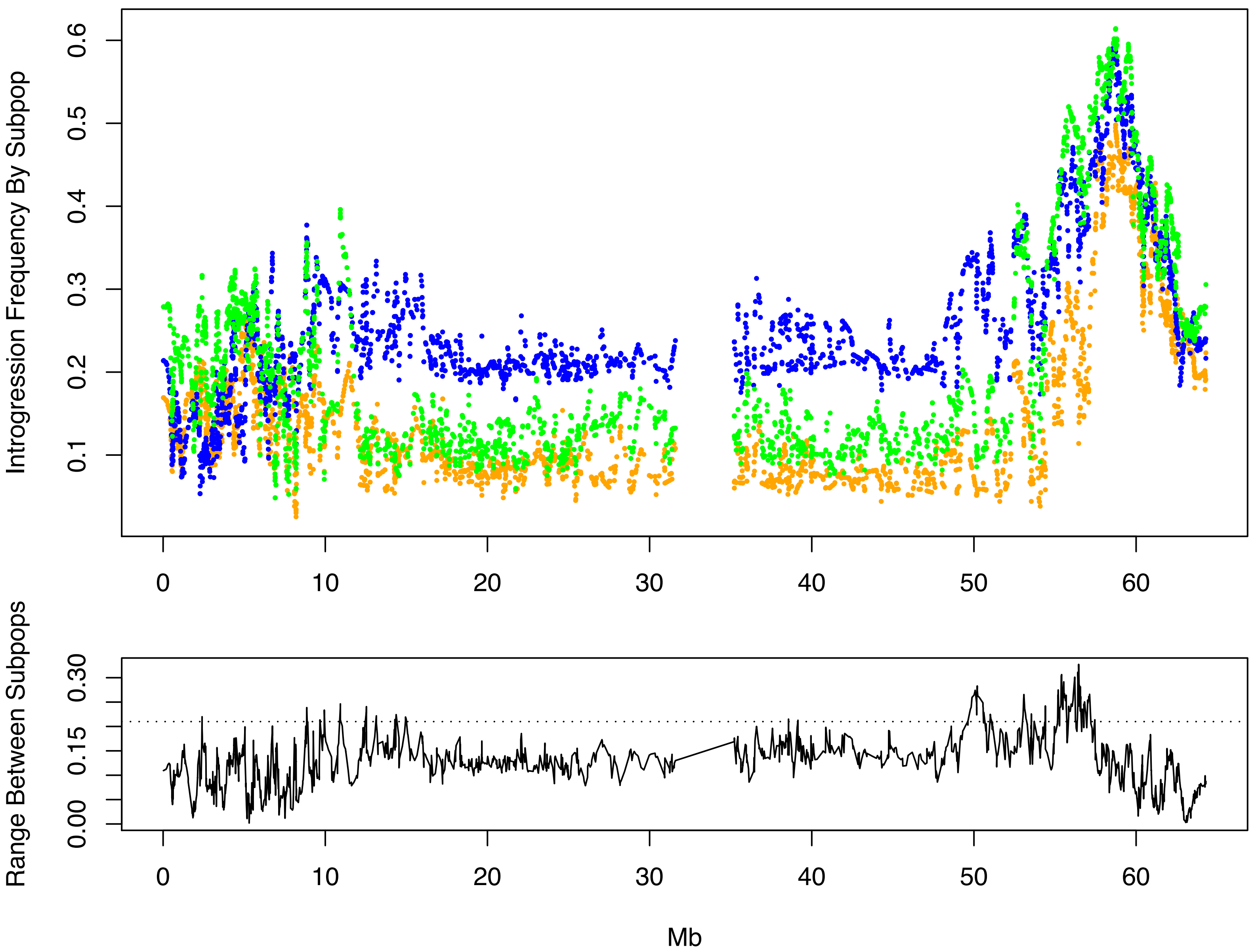

Introgression Frequency By Subpop
0.1
0.2
0.3
0.4
0.5
0.6
0
10
20
30
40
50
60
Range Between Subpops
0.00
0.15
0.30
0
10
20
30
40
50
60
Mb

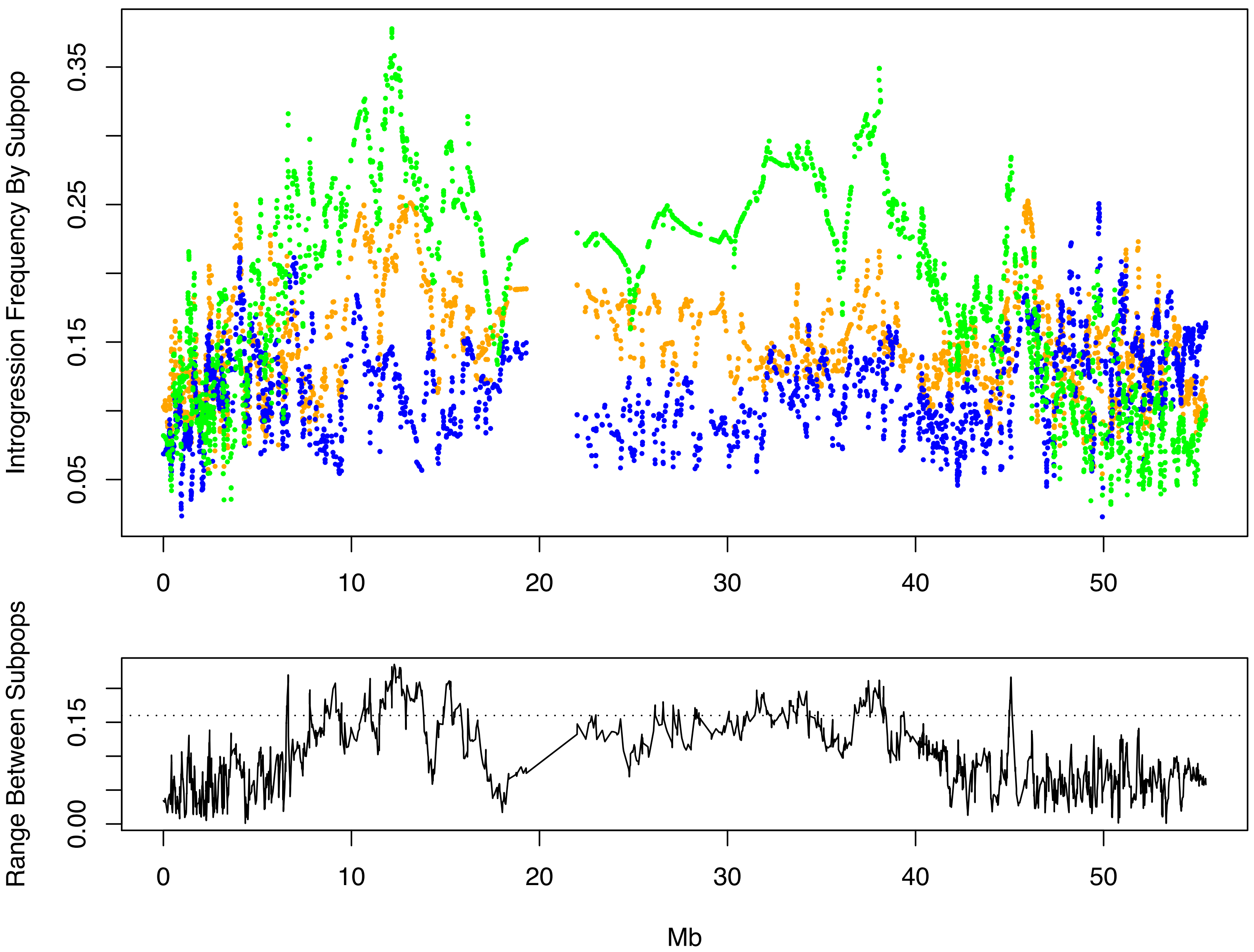

Introgression Frequency By Subpop
0.05
0.15
0.25
0.35
0
10
20
30
40
50
Range Between Subpops
0.00
0.15
0
10
20
30
40
50
Mb

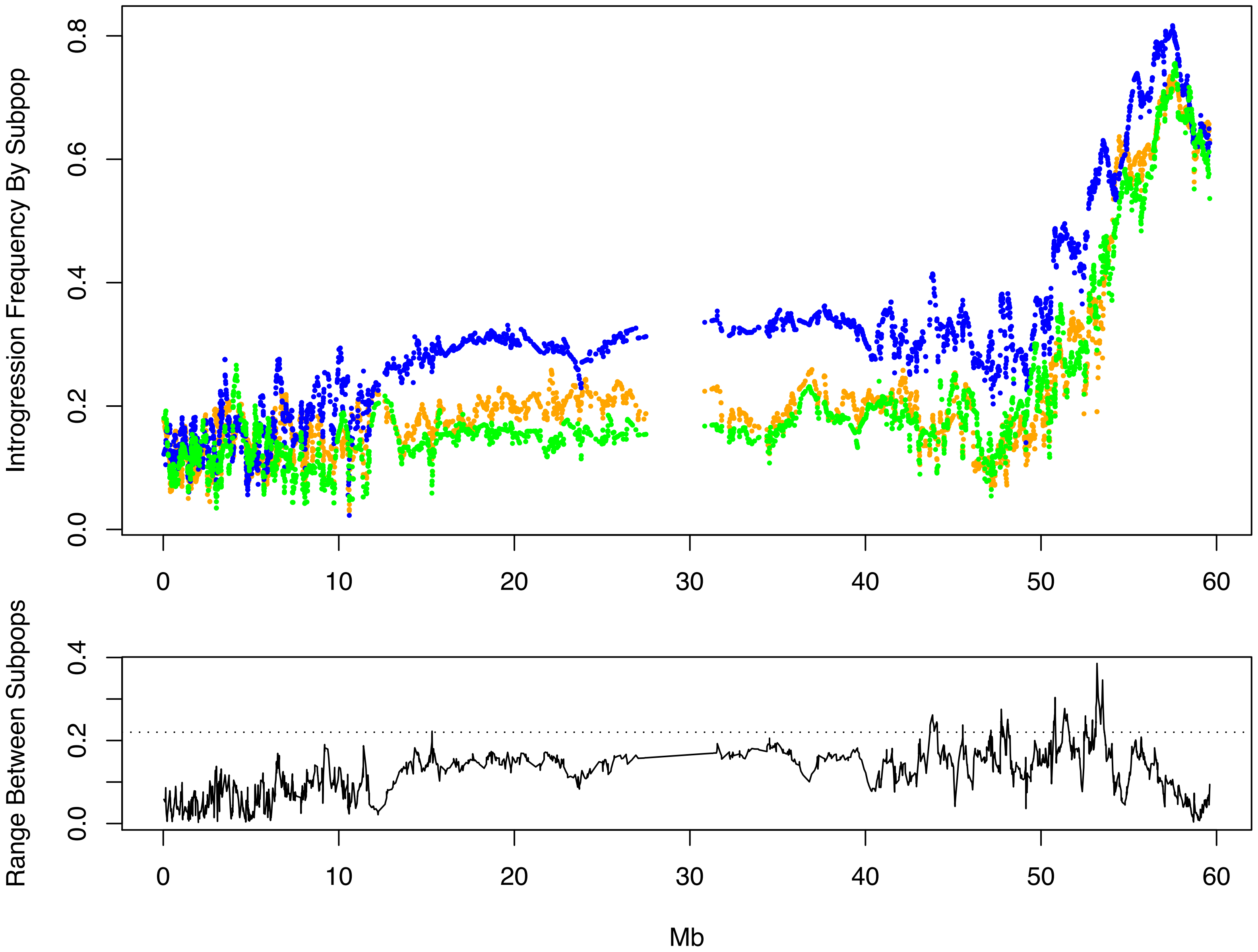
Introgression Frequency By Subpop
0.0
0.2
0.4
0.6
0.8
0
10
20
30
40
50
60
Range Between Subpops
0.0
0.2
0.4
0
10
20
30
40
50
60
Mb

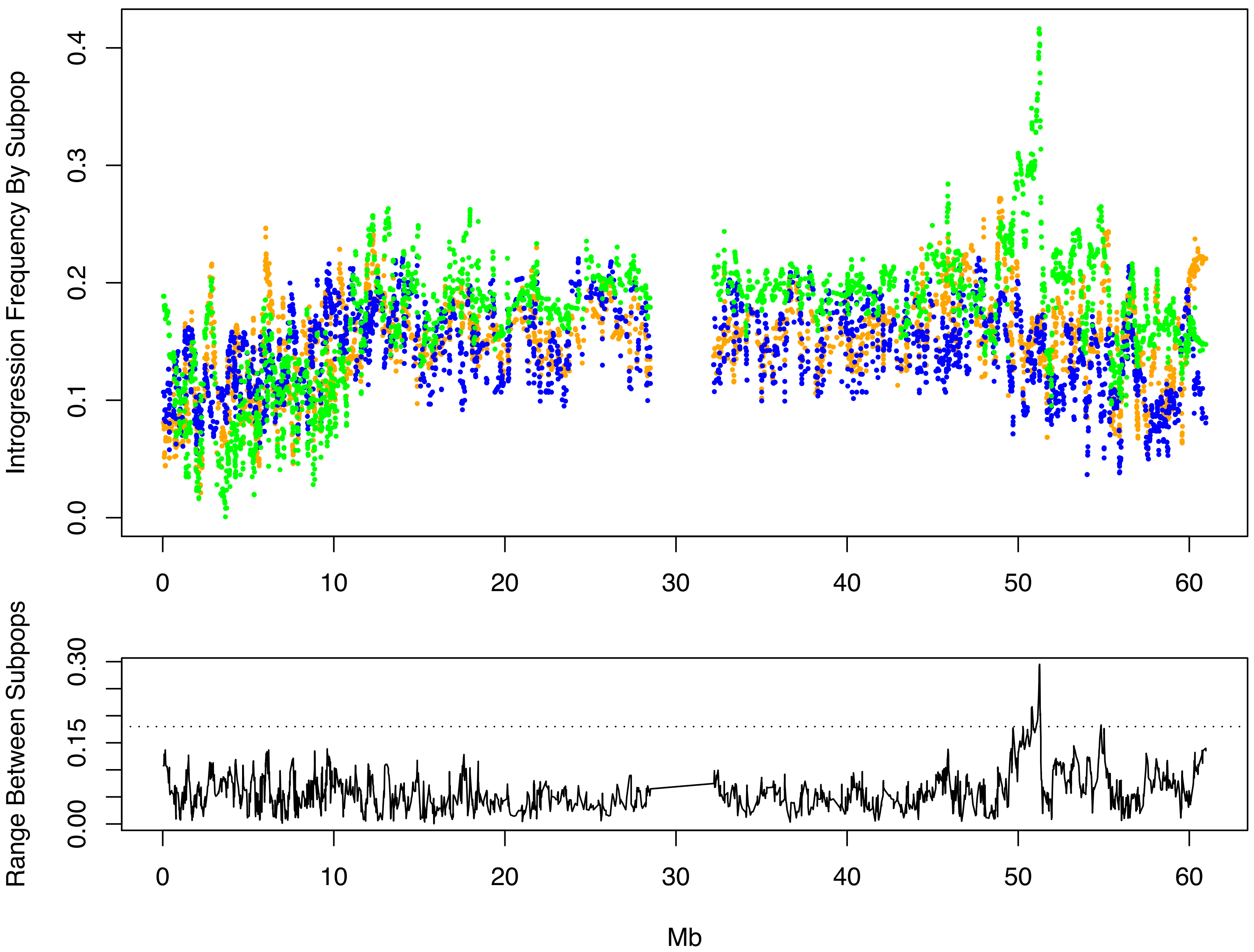

Introgression Frequency By Subpop
0.0
0.1
0.2
0.3
0.4
0
10
20
30
40
50
60
Range Between Subpops
0.00
0.15
0.30
0
10
20
30
40
50
60
Mb
